# Unified Synthesis Platform for 1,2,3-Trisubstituted Cyclopentadienyl Ligands Decouples Sterics from Electronics

**DOI:** 10.1021/jacs.5c20631

**Published:** 2026-01-09

**Authors:** Bram Van Den Bossche, Nicolai Cramer

**Affiliations:** Laboratory of Asymmetric Catalysis and Synthesis (LCSA), Institute of Chemical Sciences and Engineering, 27218 Ecole Polytechnique Fédérale de Lausanne (EPFL), 1015 Lausanne, Switzerland

## Abstract

Cyclopentadienyl (Cp) ligands are a cornerstone of coordination chemistry and transition-metal catalysis. Their tuning profoundly influences the chemical and biological reactivity, the induced selectivity, and the stability of the corresponding metal complexes. However, compared to phosphines for instance, the accessible chemical space of Cps is rather narrow, exhibiting major limitations regarding the nature, pattern, and number of Cp substituents. A unified synthetic strategy toward partially substituted Cps bearing diverse functionalities and closing gaps in chemical space is highly desirable. Herein, we report a streamlined general strategy to prepare 1,2,3-trisubstituted cyclopentadienes (1,2,3-Cps) from a central inexpensive precursor. Operationally straightforward reactions and purifications ensure scalable sequences. The robust and versatile synthesis platform opens access to underexplored Cp substitution patterns − including flexible incorporation of diverse alkyls, aryls, and previously elusive or rare functionalities such as halogens, chalcogens, and alkynes − with a profound ligand tunability and decoupling of sterics from electronics. The complexation ability with a selection of catalytically relevant early and late transition metals was demonstrated, and parametrization of the Cps with respect to their stereoelectronic environment was performed via the corresponding Cp rhodium phosphite species. In exemplary selected benchmark catalytic transformations, the cobalt and rhodium complexes directly outperformed classical Cp ligands with respect to individual reactivity, regioselectivity, and catalyst loading. Regarding catalytic turnover, a 1,2,3-Cp cobalt complex achieved an attractive turnover number (TON) of 180 for a benchmark C–H annulation.

## Graphical abstract

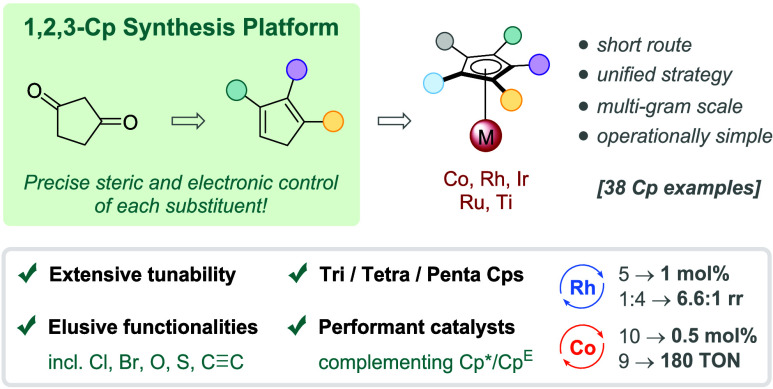

## Introduction

Cyclopentadienyl (Cp) ligands embody one of the cornerstones of organometallic chemistry. The wide variety of cyclopentadienyl metal complexes have numerous applications in homogeneous catalysis,^1^ drug discovery,^2^ and materials science.^3^ In particular, Cps have been prominently featured as excellent ancillary ligands for catalytic C–H functionalization reactions, especially with regard to group 9 transition metals.^4^ The main focus has been on expanding the scope of such transformations involving a key C–H bond cleavage event, including asymmetric ones,^1a,5^ thereby cementing it as a valuable and effective strategy for new bond formations. However, further advances are slowed down by the bottleneck of Cp ligand diversity, to which considerably less effort has been dedicated.^6^ Yet, careful Cp modulation can drastically affect the performance of the designed function of Cp metal complexes (Figure 1a). While Cp*Rh is a privileged C–H activation catalyst, diester-bearing **Cp** ^**E**^ **Rh** with its highly electron-deficient character emerged as a promising alternative rhodium­(III) catalyst, exhibiting a complementary reactivity profile.^7^ Compared to its unsubstituted analog (C_5_H_5_), the key pentamethylcyclopentadienyl (Cp*) ligand prompts an increased stabilization of the complex because of its strong electron-donating ability and steric encumbrance.^8^ As such, contrary to its noble congener [CpRhI_2_]_2_,^9^ cobalt­(III) complex **CpCo** is rarely used in catalysis due to the limited lifetime of the active species.^10^ In contrast, fully substituted Cp*Co is a stable and competent catalyst with widespread applications in C–H functionalizations.^11^ As an example for group 4 metallocenes, zirconium­(IV) complex **Cp** ^**Bu**^ **Zr** was employed as a dimerization catalyst in the total synthesis of cyctetryptomycin A and B.^12^ Lanthanide complex **Cp** ^**ttt**^ **Lu** with two stabilizing bulky tris­(*tert*-butyl)­cyclopentadienyl ligands was used to perform H_2_ splitting as well as reduction of N_2_.^13^ Among various Cp-bearing metal complexes investigated as anticancer agents, the tailored Cp ligand of iridium­(III) complex **Cp** ^**Ph2**^ **Ir** majorly improved its potency.^14^ Overall, multiple reports have illustrated how subtle steric and electronic modifications of achiral Cp ligands can distinctly influence the reaction outcome in terms of reactivity (inherent ability,^9^ catalyst turnover,^15^ or even a switch in mechanism)^16^ and regio-,^17^ diastereo-,^18^ enantio-,^19^ chemo-,^20^ or site-selectivity.^21^ Regarding chiral cyclopentadienyl (Cp^x^) ligands, the introduction of additional alkyl substituents on the Cp^x^ ring has enabled both improved yields and enantioselectivities in various rhodium-^22^ and cobalt-catalyzed^23^ asymmetric C–H functionalizations.

**Figure 1:**
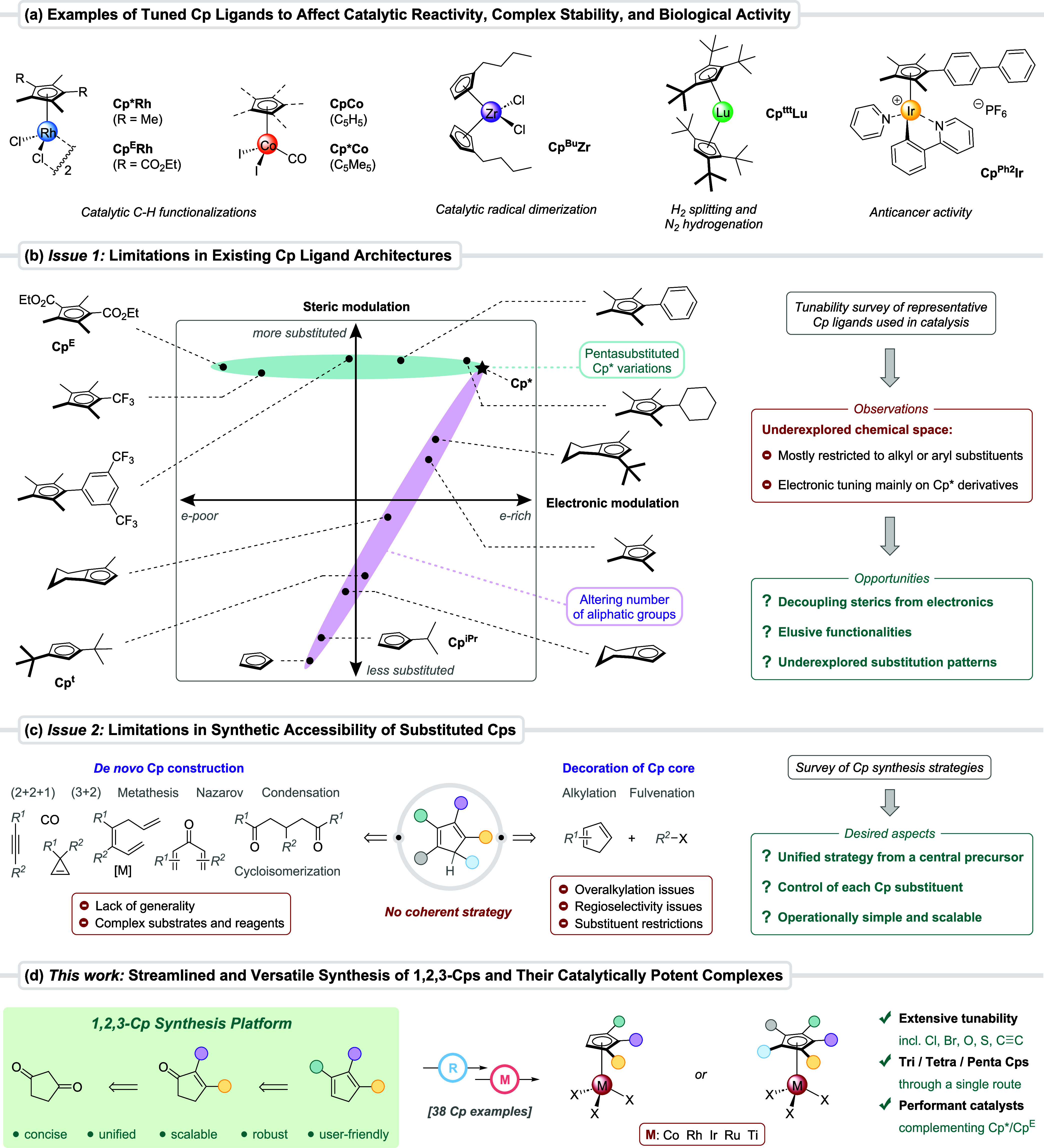
(a) Examples of substituent tuning to increase the chemical or biological performance of Cp metal complexes. (b) Visualization of the current limitations in steric and electronic tunability of catalytically relevant achiral Cp ligands and opportunities to expand the chemical space. (c) Reported approaches to construct substituted Cp cores, their key limitations, and desired aspects for a general synthetic strategy. (d) This work: development of a unified and diversity-oriented synthesis platform toward highly tunable 1,2,3-trifunctionalized Cps, tetra- or pentasubstituted derivatives, and their catalytically performant metal complexes.

Upon visualization of representative achiral Cp architectures in catalysis across a steric and electronic dimension, the underpopulated chemical space becomes evident (Figure 1b). With Cp* as an anchor point, the chemical space has been traversed in two distinct directions. The first (green) axis comprises an exchange of one or multiple methyl groups. Some steric modulation can be achieved by installing different alkyl or aryl groups,^20,24^ whereas highly electron-deficient Cp* variants contain CF_3_ or ester functionalities.^7a,25^ Electronically different Cps can be accessed, but the approach is restricted to sterically encumbered pentasubstituted ligands. Consequently, these are limited to implementation in transformations tolerant of such bulk on the catalyst. The second (purple) axis moves along the degree of substitution on the Cp ring. Only limited examples are available of these partially substituted Cps, and the few variations usually bear simple alkyl or phenyl groups.^17,26^ Although a decrease in substitution goes hand in hand with a less electron-rich nature, no comprehensive electronic modulation has been investigated thus far. A desirable decoupling of sterics from electronics in Cp synthesis (i.e., an independent choice of the attached functionalities and the degree of Cp substitution) remains an unresolved issue. To address this gap and populate the vacant central zone of the Cp chemical space, we aimed to construct 1,2,3-trisubstituted Cps (1,2,3-Cps) bearing a wide variety of substituents.

At the outset of our studies, we surveyed reported strategies to access substituted cyclopentadienes (Figure 1c).^27^ The majority of Cp syntheses rely on the *de novo* construction of the five-membered ring. In addition to some specific examples,^28^ these comprise (2 + 2 + 1)^29^ or (3 + 2)^30^ annulations, Nazarov-type cyclizations,^25,31^ cycloisomerizations,^32^ and intramolecular metatheses^33^ or condensations.^34^ The recurring limitation is a lack of generality due to severe scope constraints regarding the nature, pattern, and number of Cp substituents. Additional drawbacks stem from a high step count, problematic purifications, and the use of expensive reagents or tailored substrates, overall hindering widespread application of new Cp ligand architectures in catalysis. The second main approach involves decoration of preexisting cyclopentadiene cores, such as direct alkylations^35^ or through fulvenation reactions.^22a^ Generality as well as regioselectivity and overalkylation issues are key limitations. An alternative is a tandem nucleophilic addition–elimination sequence with a cyclic enone. For instance, tetramethylcyclopentenone in combination with organolithium reagents furnishes Cp* derivatives.^36^ However, for Cps with lower degrees of substitution, this approach remains largely underexplored,^29a,29b,37^ and a coherent strategy to construct 1,2,3-Cps with a flexible substituent choice is absent. Reliance on a different synthesis route for each ligand is time- and resource-intensive and hampers their application in catalyst screenings. *Therefore*, *a unified strategy for constructing partially substituted Cps*, *able to span the electronic spectrum and independently offer steric tuning options*, *is highly desirable*.

Herein, we report a general strategy and unified synthesis platform to access the underexplored substitution pattern of 1,2,3-trisubstituted Cps and accommodate previously elusive functionalities (Figure 1d). It features a short and robust synthetic route employing cyclopentanedione as an inexpensive central feedstock precursor. Well-understood and operationally straightforward reactions ensure scalable sequences. The developed strategy effectively decouples sterics from electronics, thereby considerably expanding the known chemical space of Cp ligands. We demonstrate complexation of the obtained cyclopentadienes to a selection of transition metals and collect characteristic parameters. Exemplarily benchmarking these complexes in challenging C–H functionalization reactions showcases their superior catalytic performance.

## Results and Discussion

### Design and Construction of a 1,2,3-Cp Synthesis Platform

To meet our objective of easily accessible and structurally diverse 1,2,3-Cps, we envisioned the tandem addition–elimination of enones as the foundation of a robust, simple, and unified synthetic route. The required 2,3-disubstituted cyclopentenones ultimately trace back to inexpensive 1,3-cyclopentanedione **1** (Scheme 1a). We employed straightforward chemistry to install the central 2-substituent, whereas separate nucleophilic addition events (through enol ether and enone intermediates) with commercial organometallic reagents provided the 1- and 3-substituents. First, cyclopentanedione **1** was alkylated via a reductive Knoevenagel condensation to afford **2a**–**b** bearing secondary alkyl groups.^38^ The *tert*-butyl group of **2c** was installed via S_N_1 chemistry with *t*BuOH in aqueous medium in 42% yield, thereby substantially improving on the reported 7-step method (5% overall yield).^39^ The resulting 2-alkyldiones **2** were converted into enol ethers **3** (60–84% yield). In turn, these afforded enones **4**–**5** in good to excellent yields upon treatment with an organolithium or Grignard reagent and acidic workup. Noteworthily, both steps delivered intermediates **3** and **4**–**5** in sufficient purity and did not require purification. When 2-aryl, -alkynyl, or -heteroatom substitution was required, we switched the order of steps, and the central 2-substituent was attached after obtaining the enone core (Scheme 1a, bottom). Tandem dihalogenation−elimination of inexpensive 3-methylcyclopentenone **6** furnished bromo- and iodo-bearing products **7**.^40^ Next, a large array of 2-aryl-substituted enones **8** could be obtained from **7a** by Suzuki–Miyaura cross-coupling (69–99% yield). In addition, Sonogashira cross-coupling of **7b** provided alkynyl-bearing enones **9** in 74–94% yield. Lastly, heteroatom-substituted enones **11** were accessed through base-catalyzed epoxidation of **6** and telescoped treatment of intermediate **10** with cerium chloride^41^ or (thio)­phenol nucleophiles.^42^ The sulfide group of **11c** was oxidized using oxone to afford sulfone **11d** in an 84% yield.

**Scheme 1:**
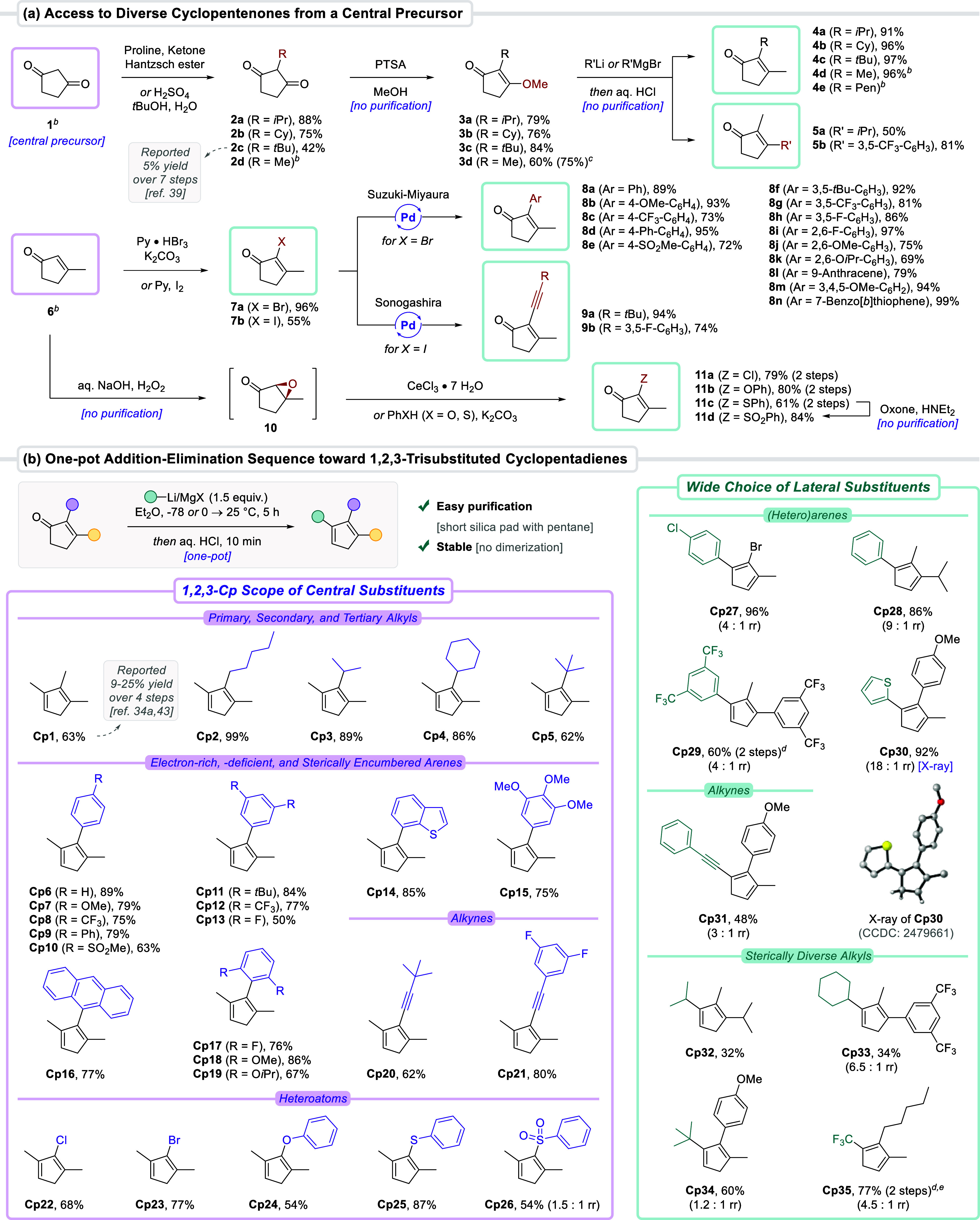
Unified and Diversity-Oriented Synthesis Platform for 1,2,3-Trisubstituted Cyclopentadienes^a^

a Isolated yields. For detailed conditions, see the Supporting Information. In the case of double bond isomers: major isomer as depicted, rr = regioisomeric ratio. The solid-state X-ray structure of **Cp30** shows 50% probability thermal ellipsoids; hydrogen atoms (except for those on the Cp ring) and disorder are omitted for clarity.
b Commercial compounds.
c Yield for ethyl enol ether.
d For elimination: PTSA, PhMe, 80 °C.
e For trifluoromethylation: TBAF, TMSCF_3_, and then aq. HCl.

The scope of our 1,2,3-Cp synthesis platform was established following a one-pot addition–elimination sequence (Scheme 1b). Volatile trimethylcyclopentadiene **Cp1** previously required a laborious 4-step procedure, including a poorly reproducible oxidative homocoupling of 2-butanone using toxic PbO_2_, which resulted in a low overall yield (reported 9–25%).^34a,43^ Furthermore, mixtures of isomers were formed at each step, thus complicating the analysis. Here, **Cp1** was directly prepared from enone **4d** in 63% yield upon consecutive treatment with MeLi·LiBr and aqueous HCl. Other primary, secondary, and tertiary 2-alkyl-substituted Cps were equally accessible in good to excellent yields (**Cp2–5**, 62–99%). The presence of both electron-rich and electron-poor arene substituents was tolerated (**Cp6–15**). Hindered *ortho*-functionalized 2-arylenones were also converted without issues into cyclopentadienes **Cp16–19** (67–86% yield). Alkynyl-bearing **Cp20–21** could be accessed, and importantly, the triple bond did not result in any undesired dimerization through intermolecular [4 + 2] cycloaddition. In the case of chloro- and bromo-substituted **Cp22–23**, using MeMgBr instead of MeLi·LiBr circumvented parasitic halogen–lithium exchanges. Furthermore, we were pleased to isolate oxygen- and sulfur-bearing **Cp24–26** without stability issues that could originate from their electronically strongly biased nature. The many commercially available organolithium and Grignard reagents offer a wide choice of lateral 1,2,3-Cp substituents to install (Scheme 1b, right). Addition of (hetero)­arenes (**Cp27–30**), alkynyl (**Cp31**) and sterically diverse alkyl groups (**Cp32–34**) was all tolerated, including the use of bulky *tert*-butyllithium in 60% yield. Competitive deprotonation was responsible for the occasionally lower yields observed. However, in such a case, the enolized substrate was recovered afterward, and switching organometallic reagents could aid in favoring nucleophilic over basic behavior. Lastly, the combination of TBAF with the Ruppert–Prakash reagent enabled selective 1,2-CF_3_ addition, resulting in trisubstituted trifluoromethyl-bearing cyclopentadiene **Cp35**. In this case, dehydration of the cyclopentenol intermediate did not occur upon acidic workup, and heating in the presence of PTSA was necessary.

Lowering the number of substituents on a cyclopentadiene ring usually decreases its stability.^44^ Steric bulk is required to dampen decomposition pathways such as self-Diels–Alder or polymerizations. Importantly, the 1,2,3-Cps in this work are stable to be isolated, purified, and handled under ambient conditions without precautions, and they can be stored in a freezer for many months without decomposition. Although distillation is possible, the apolar character of most 1,2,3-Cps allowed simple and fast purification through a short pad of silica, usually with just pentane. Note that, despite the prevalent tendency of [1,5]-H sigmatropic shifts in many substituted cyclopentadienes,^45^ the majority of our 1,2,3-Cps were obtained as a single double bond isomer, thus simplifying analysis. Thiophene-bearing **Cp30** is one of the few solid 1,2,3-Cps at 25 °C, of which a single crystal allowed X-ray-mediated structural confirmation. In the case of unsymmetrically substituted Cps, the major isomer was always the thermodynamically most stable one. To exemplify the scalability of our synthesis platform (Scheme 2a), a multigram run of the route toward isopropyl-bearing **Cp3** was carried out in very good yields (73–91%), ultimately affording 3.93 g (28.9 mmol) of 1,2,3-Cp product.

**Scheme 2:**
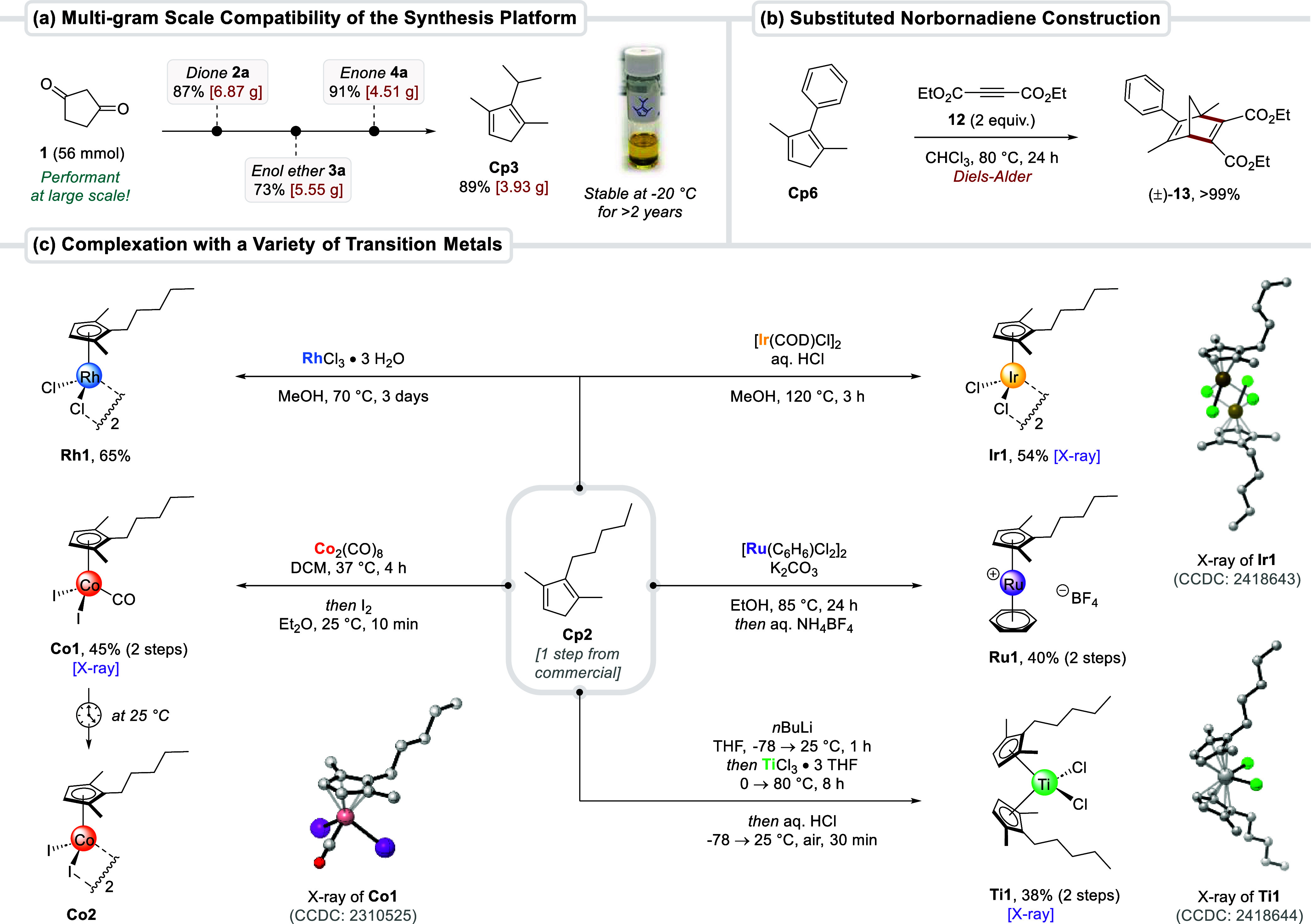
Multigram-Scale Synthesis, Diels–Alder Cycloaddition, and Transition-Metal Complexation of 1,2,3-Cps^a^

a Isolated yields. The solid-state X-ray structures of **Co1**, **Ir1**, and **Ti1** show 50% probability thermal ellipsoids; hydrogen atoms, solvent molecules, and disorder are omitted for clarity.

### Derivatization of 1,2,3-Cps and Their Complexation to Transition Metals

Aside from their predominant application as ligands in coordination chemistry, the cyclopentadiene motif can also serve as a very useful handle for Diels–Alder reactions.^44^ To demonstrate such [4 + 2] cycloaddition competence, **Cp6** was treated with alkyne **12** at an elevated temperature of 80 °C to afford substituted norbornadiene **13** in quantitative yield (Scheme 2b). A resolution of racemic diene **13** would open possibilities for its evaluation as a chiral diene ligand in asymmetric catalysis.^46^ The rich metalation chemistry of cyclopentadienes was highlighted by the complexation of **Cp2** to five different transition metals (Scheme 2c). Simple prolonged exposure to rhodium trichloride at 70 °C provided the dimeric complex **Rh1** in 65% yield. An Ir­(I) precursor in the presence of aqueous HCl afforded iridium­(III) analog **Ir1**. Dicobalt octacarbonyl paired with one-pot oxidation using iodine furnished cobalt­(III) complex **Co1** in 45% yield. Interestingly, the monomeric piano-stool configuration of **Co1** does not persist at ambient temperature. The CO ligand turned out to be rather labile and got slowly released over time (even in the solid state), converting complex **Co1** into dimeric species **Co2**. In contrast, Cp*Co­(CO)­I_2_ is stable at 25 °C and decarbonylation to [Cp*CoI_2_]_2_ occurs only above 100 °C.^10^ This difference illustrates how changing the degree of Cp substitution directly influences the dissociation rates of other ligands and the relative strength with which they are bound to the metal − both exploitable features for catalysis. In the case of ruthenium (group 8), a thallium-free complexation with [Ru­(C_6_H_6_)­Cl_2_]_2_ and subsequent anion exchange provided **Ru1** in 40% overall yield. For titanium (group 4), deprotonation of **Cp2** with BuLi and addition of titanium trichloride gave a Ti­(III) intermediate, which oxidized upon exposure to air and one-pot addition of aqueous HCl to deliver bent metallocene **Ti1**. The solid-state structures of **Ir1**, **Co1**, and **Ti1** were unambiguously confirmed through X-ray crystallography.

Next, a set of 1,2,3-Cps with uncommon or elusive substituents were complexed to rhodium through a telescoped Rh­(I)-complexation/oxidation sequence (Scheme 3a). The process is general, and all major variations in the 1,2,3-Cp substituents’ electronic nature, size, and pattern were tolerated and provided the corresponding rhodium­(III) complexes **Rh2–16** in overall good yields. Particularly, heteroatom-bearing Cp ligands are very rare,^47^ and catalytic potentials of the corresponding metal complexes remain underexplored. In fact, to the best of our knowledge, only two reports involve a C–H functionalization reaction using an oxygen-bearing CpRh catalyst.^47d,47e^ To fill this gap, chloro-, bromo-, phenoxy-, sulfide-, and sulfone-bearing cyclopentadienyl rhodium complexes (**Rh3–4** and **Rh6–8**) were prepared. To the best of our knowledge, **Rh12–13** are the first examples of alkynyl-substituted CpRh complexes. Complexes **Rh14–15** further illustrate the sterically diverse and unique substitution patterns that are obtainable. Metalating unsymmetrically substituted 1,2,3-Cps results in planar chiral complexes. As such, resolution of racemic complexes, **Rh13–15** for example, opens possibilities for future applications in enantioselective catalysis.

**Scheme 3:**
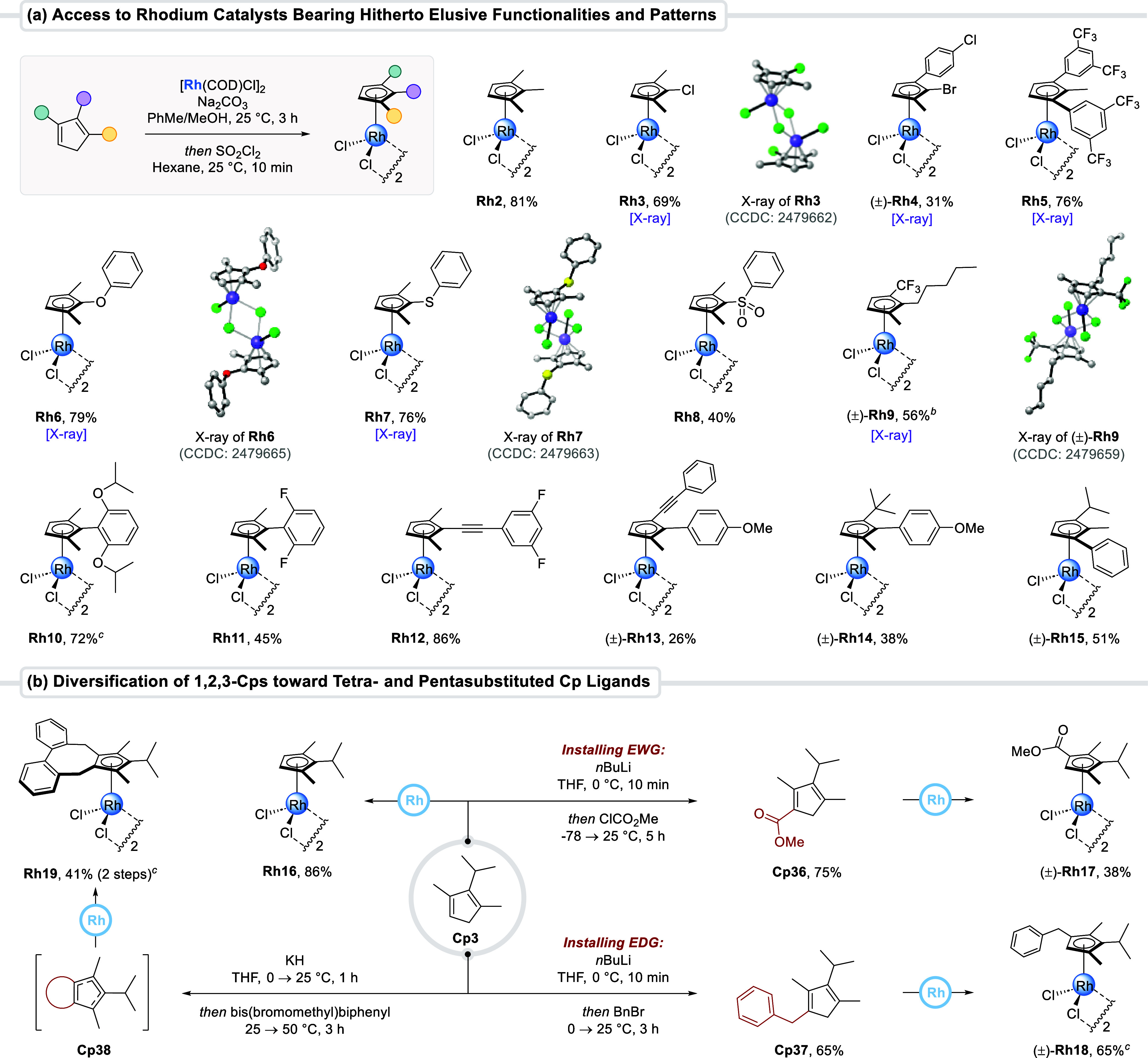
Assembly of 1,2,3-Cp Rhodium Catalysts with Elusive Functionalities and Substitution Patterns and Diversification of 1,2,3-Cps toward Tetra- and Pentasubstituted Cp Ligands^a^

a Isolated yields. The solid-state X-ray structures of **Rh3**, **Rh6**, **Rh7**, and **Rh9** show 50% probability thermal ellipsoids; hydrogen atoms, solvent molecules, and disorder are omitted for clarity. EWG = electron-withdrawing group. EDG = electron-donating group.
b With KOAc at 70 °C instead of Na_2_CO_3_ at 25 °C.
c Altered conditions: RhCl_3_·3 H_2_O, *i*PrOH, 90 °C, 18 h.

To cover additional chemical space, we increased the degree of Cp substitution (Scheme 3b). In this respect, deprotonation of the 1,2,3-Cps, followed by reaction with an alkyl or acyl electrophile, enables the introduction of a respectively electron-rich or electron-deficient fourth substituent. For instance, deprotonation of **Cp3** with BuLi and subsequent quench with methyl chloroformate provided tetrasubstituted **Cp36** bearing an ester functionality in 75% yield and was converted to rhodium complex **Rh17**. In a similar fashion, **Cp3** was alkylated with benzyl bromide, giving **Cp37**. Subsequent η^5^-coordination to rhodium­(III) provided complex **Rh18** in 65% yield. Finally, the transformation of 1,2,3-Cps into pentasubstituted cyclopentadienyl ligands was demonstrated. A *bis*-electrophile harboring two bromide leaving groups was selected for the double alkylation. The resulting ligand **Cp38** was telescoped to the metalation step, allowing isolation of rhodium complex **Rh19** in 41% overall yield. In analogy, the use of chiral *bis*-electrophiles would provide access to structurally diverse pentasubstituted Cp^x^ ligands in a single step.^48^ Efforts in this direction and subsequent applications in asymmetric catalysis are currently underway in our group.

### Electronic Assessment of 1,2,3-Cp Rhodium­(III) Complexes by ^31^P NMR

The stereoelectronic environment of the Cp ligands was subsequently evaluated (Figure 2). Ligand and catalyst parametrization plays a pivotal role in providing essential training data for machine learning models and aids in the development of predictive models for *in silico* catalyst optimization.^6a^ The [CpRhCl_2_]_2_ complexes were converted with triethyl phosphite in CD_2_Cl_2_ to CpRhCl_2_P­(OEt)_3_ adducts **Rh**′. ^31^P NMR analysis provided the chemical shift of the phosphorus nucleus (δ_P_) and its coupling with ^103^Rh (*J*_Rh–P_). These parameters are both related to the (de)­shielding of the ^31^P nucleus, which is affected by the electron-withdrawing character of the bound metal and, consequently, by the electron-richness of its Cp ligand. As such, more electron-deficient Cps result in lower δ_P_ and *J*_Rh–P_ values.^15,49^ Previously, these parameters were measured for simple classical Cp ligands by Rovis,^6a^ thus allowing comparison with the complexes described herein. Overall, the coupling constant *J*_Rh–P_ appears to be a more robust proxy for the Cp electronics than the chemical shift δ_P_. For instance, the small δ_P_ decrease from pentasubstituted Cp*Rh′ (114.2 ppm) to monosubstituted **Cp** ^**iPr**^ **Rh**′ (110.5 ppm) does not account for the established electron-deficient nature of the latter, whereas the significant difference in *J*_Rh–P_ does (215.4 vs 201.6 Hz). When comparing pentasubstituted **Rh19**′, its coupling constant (*J*_Rh–P_ = 215.9 Hz) closely resembles the Cp* ligand, despite having a similar chemical shift (δ_P_ = 110.2 ppm) to **Cp** ^**iPr**^ **Rh**′.

**Figure 2:**
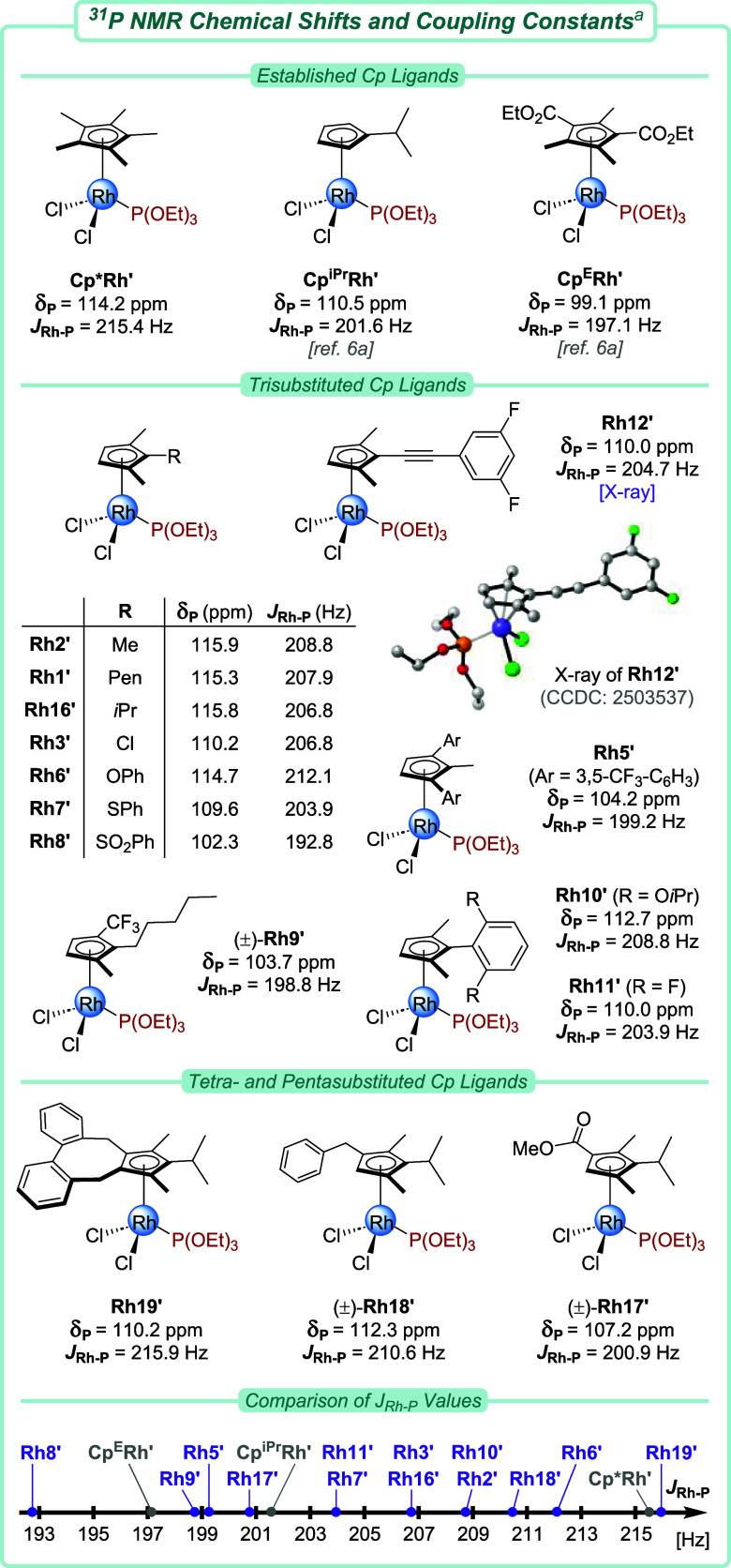
Evaluation of the stereoelectronic environment of the Rh­(III) center. The solid-state X-ray structure of **Rh12‘** shows 50% probability thermal ellipsoids; hydrogen atoms and disorder are omitted for clarity. ^*a*^ Measured in CD_2_Cl_2_.

The substitution degree of 1,2,3-trisubstituted Cps lies exactly between penta- and monoalkylated Cps. Notably, the electronic nature of 1,2,3-Cps was found to behave accordingly. For instance, the coupling constant of trimethyl-bearing **Rh2′** (*J*_Rh–P_ = 208.8 Hz) almost matches the averaged value of Cp*Rh′ and **Cp** ^**iPr**^ **Rh**′ (*avg*. 208.5 Hz). As expected, swapping a methyl group for a chloro (**Rh3**′, *J*_Rh–P_ = 206.8 Hz) or alkynyl group (**Rh12**′, *J*_Rh–P_ = 204.7 Hz) rendered the ligand more electron-poor. For aryl-substituted 1,2,3-Cps, the electron-donating or -withdrawing influence of, respectively, alkoxy (**Rh10**′, *J*_Rh–P_ = 208.8 Hz) and fluoro substituents (**Rh11**′, *J*_Rh–P_ = 203.9 Hz) on the phenyl ring was demonstrated. Ligands bearing two electron-deficient aromatic rings (**Rh5**′) or a CF_3_ group (**Rh9**′) were found to be highly electron-deficient, displaying coupling constants *J*_Rh–P_ almost as low as that of diester-functionalized **Cp** ^**E**^ **Rh**′. For chalcogen substituents, both inductive (−*I*) and mesomeric (+*M*) effects may influence the overall electronic outcome. The +*M* effect was found to dominate in the case of phenoxy-substituted complex **Rh6**′ (*J*_Rh–P_ = 212.1 Hz) in contrary to electron-poor sulfide-bearing complex **Rh7**′ (*J*_Rh–P_ = 203.9 Hz). Interestingly, the strongly electron-withdrawing sulfone substituent in **Rh8**′ displayed an extremely low value of *J*_Rh–P_ = 192.8 Hz, outcompeting **Cp** ^**E**^ **Rh**′ (197.1 Hz). Lastly, trialkylated complex **Rh16**′ was compared to its tetrasubstituted derivatives **Rh17–18**′. The increased *J*_Rh–P_ value of benzylated **Rh18**′ suggests a more electron-rich ligand, whereas the electron-withdrawing ester group of **Rh17′** resulted in a smaller coupling constant, underscoring the electronic tuning of these Cps. Notably, the electronic character of **Rh17**′ (*J*_Rh–P_ = 200.9 Hz) is similar to that of monosubstituted complex **Cp** ^**iPr**^ **Rh**′ (*J*_Rh–P_ = 201.6 Hz) despite their very different steric encumbrance and degree of substitution. Overall, the described Cp substitutions and functionalities result in a widely different electronic nature of the resulting metal complexes, as evidenced by the broad range of δ_P_ (102.3–115.9 ppm) and *J*_Rh–P_ (192.8–215.9 Hz) values. Paired with their steric differences, the 1,2,3-Cp platform covers a broad chemical space along both dimensions, and importantly, it allows the decoupling of sterics from electronics in Cp ligand synthesis.

### Catalytic Performance of 1,2,3-Cp Metal Complexes

The demonstrated tunability as well as the occupancy of a central zone of the chemical space depicted in Scheme 1b likely make 1,2,3-Cps an attractive starting point in catalyst tailoring for new transformations. Their balanced steric and electronic properties lying between un- and pentasubstituted Cp ligands suggest an increased likelihood of achieving the desired catalytic reactivity. To support this hypothesis, the catalytic ability of trisubstituted complexes **Ir1** and **Ti1** was tested on two transformations that were reported to work either with a penta- or monosubstituted Cp ligand, respectively (Scheme 4).^50,51^ **Ir1** catalyzed the C­(sp^3^)-H amidation of oxime **14** with tosyl azide, providing product **16** in 57% yield and thus proceeding with an efficiency similar to Cp*. The reductive Ti­(III)-catalyzed acyloin-type coupling of ketone **17** with nitrile **18** dominantly prefers complexes with a low degree of Cp substitution. Monosubstituted C_5_H_4_Et was reported as the best ligand, and under similar conditions, trisubstituted **Ti1** delivered α-hydroxyketone **19** in 52% yield.

**Scheme 4:**
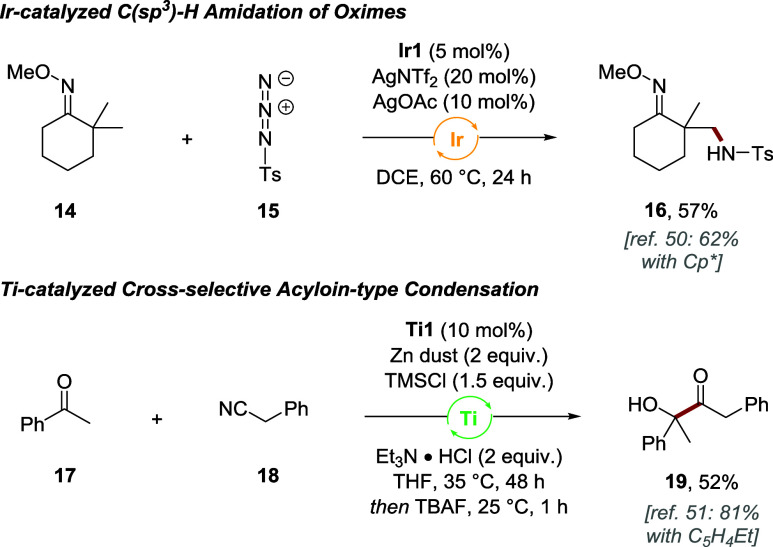
Catalytic Ability of 1,2,3-Cp Iridium and Titanium Complexes in Transformations Previously Limited to Penta- or Monosubstituted Cp Ligands

To further gauge the catalytic behavior of complexes bearing the 1,2,3-substitution pattern and the newly accessible functionalities, we chose among the plethora of potential catalytic transformations a selection of challenging C–H functionalization reactions that could benefit from improved catalytic performance with respect to reactivity, regioselectivity, and turnover number (TON) among others. Importantly, our goal was to rapidly showcase the utility and future potential of these Cp ligands. Without resorting to exhaustive screening rounds, we performed spot-checks using a restricted but diverse set of complexes to illustrate a clear response in the reaction outcome to a change in Cp substituents. First, we investigated the formation of 2-substituted indoline **22** (Table 1).^7b^ This transformation heavily relies on the electron-deficient **Cp** ^**E**^ **Rh** catalyst to achieve a good yield (Entry 1). Classical Cp*Rh provided under identical conditions only 40% yield (Entry 2). We found that lowering the Cp substitution degree substantially improved reactivity, as trialkyl-substituted catalyst **Rh1** restored the yield to 82%, thereby matching the TON of **Cp** ^**E**^ **Rh** (Entry 3). Placing an electron-withdrawing chloro (**Rh3**) or CF_3_ group (**Rh9**) on the Cp resulted in a further yield increase (Entries 4 and 5). The unique sulfide-bearing Cp complex **Rh7** performed even better, furnishing the indoline product in 92% yield and allowing a catalyst loading reduction to 3 mol % without impact (Entries 6 and 7). Interestingly, other catalysts bearing newly accessible substituents, such as bromo (**Rh4**) and alkynyl (**Rh12**) groups, also provided an excellent yield at this reduced loading (Entries 8 and 9). Aryl-substituted (**Rh5** and **Rh10–11**) and ester-bearing (**Rh17**) complexes proved to be less efficient (Figure S8). The effect of a chalcogen substituent on the Cp ring was further probed at 2 mol % of rhodium (Entries 10–12). Complex **Rh8** with its severely electron-withdrawing sulfone group did not perform better than **Rh7** (18% vs 52% yield). However, installing a phenoxy functionality (**Rh6**) provided a 67% yield with the highest TON of 33. These results indicate that a catalyst’s performance in this transformation cannot simply be explained by the (non)­presence of an electron-withdrawing group on the Cp ring. While electronic parameters, such as the *J*_Rh–P_ values, do give valuable insights, other effects may lie at the basis of the distinct but different outcomes that the various heteroatom substituents provide. Bearing in mind how the synthesis of electron-deficient **Cp** ^**E**^ **Rh** unlocked complementary reactivity to Cp*Rh,^7^ our results point to a broader potential for heteroatom-bearing 1,2,3-Cp complexes in the discovery and optimization of new catalytic applications.

**Table 1:**
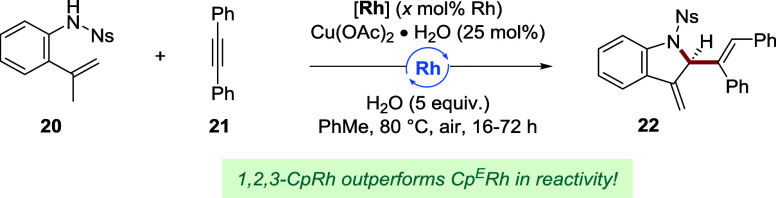
Catalytic Efficiency of Different Cp Ligands in a Rh­(III)-Catalyzed C–H Annulation for 2-Substituted Indolines

Entry	[Rh]	mol % Rh^a^	*t* (h)	% yield^b^	TON
1	**Cp** ^**E**^ **Rh**	6	16	84^c^	14
2	Cp*Rh	6	20	40	7
3	**Rh1**	6	20	82	14
4	**Rh3**	6	20	88	15
5	**Rh9**	6	20	90	15
6	**Rh7**	6	20	92	15
7	**Rh7**	3	24	88^d^	29
8	**Rh4**	3	24	90	30
9	**Rh12**	3	24	89	30
10	**Rh7**	2	72	52	26
11	**Rh8**	2	72	18	9
12	**Rh6**	2	72	67	33

a Loading refers to monomeric CpRh.

b Determined by qNMR with 1,3,5-trimethoxybenzene as an internal standard.

c As reported.^7b^

d Isolated yield.

Next, we investigated the construction of substituted pyridines from α,β-unsaturated oxime **23** (Table 2).^52^ A judicious choice of Cp ligand is essential to control the regioselective migratory insertion of unsymmetrical alkyne coupling partner **24**. Previously, pyridine isomers **25a** and **25b** were obtained in a 1:4 regioisomeric ratio (rr) with [**Cp** ^**t**^RhCl_2_]_2_ as the catalyst (**Cp** ^**t**^ **Rh**, Entry 1). Cp*Rh delivered a lower yield, but favoring opposite regioisomer **25a** in a ratio of 1.4:1 (Entry 2). As such, this transformation provides a proofing ground for the tunability of the reaction outcome (i.e., reactivity and selectivity) as a response to different Cp substituents. Lowering the degree of Cp substitution improved both the yield and regioselectivity (Entries 3 and 4), particularly for the trisubstituted catalyst **Rh1** (89%, 5.6:1 rr). Exchanging its pentyl group for different functionalities had a pronounced effect (Entries 5–9). For instance, **Rh10** with an electron-rich *ortho*-disubstituted arene attached furnished **25a** in 96% yield and 4.9:1 rr. An isopropyl group on the Cp (**Rh16**) improved the selectivity to 6.1:1 rr, whereas a smaller methyl group lowered it (**Rh2**, 4.9:1 rr). Chloro- (**Rh3**) and sulfide-substituted (**Rh7**) complexes gave inferior yields, contrasting to their excellent performance for the synthesis of indoline **22** and illustrating the necessity of a diverse 1,2,3-Cp ligand set to cater different applications. Electron-deficient complex **Rh5** likewise resulted in diminished reactivity (Figure S9). The different steric profiles of **Rh14** (1:1 rr) and pentasubstituted **Rh19** (1:1.4 rr) were reflected by their distinct selectivity values (Entries 10 and 11). With **Rh16** as the best-performer of the selection, using cesium carbonate as a base provided C–H annulation product **25a** in 91% isolated yield and a regioselectivity of 6.6:1 (Entry 12). The catalyst loading could be reduced to 1 mol % of rhodium (Entries 13 and 14), showing that **Rh16** not only provides good complementary regioselectivity compared to **Cp** ^**t**^ **Rh**, but additionally improves the TON from 17 to 58.

**Table 2:**
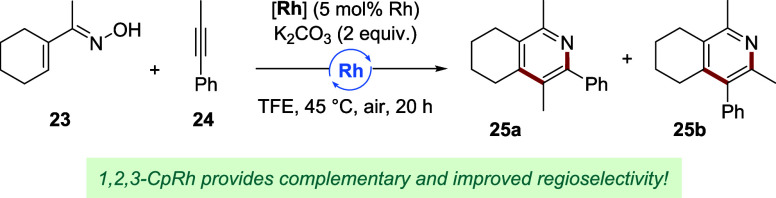
Ligand-Controlled Regioselectivity for Pyridine Synthesis

Entry	[Rh]	deviation^a^	% yield^b^	**25a**/**25b** ^b^
1	**Cp** ^**t**^ **Rh**	−​	87^c^	1:4
2	Cp*Rh	−​	75	1.4:1
3	**Rh18**	−​	72	2.2:1
4	**Rh1**	−​	89	5.6:1
5	**Rh10**	−​	96	4.9:1
6	**Rh16**	−​	87	6.1:1
7	**Rh2**	−​	91	4.9:1
8	**Rh3**	−​	13	6.1:1
9	**Rh7**	−​	22	3.5:1
10	**Rh14**	−​	93	1:1
11	**Rh19**	−​	45	1:1.4
12	**Rh16**	Cs_2_CO_3_	91^d^	6.6:1
13	**Rh16**	Cs_2_CO_3_, 0.2 M, 2 mol % Rh, 72 h	85	6.3:1
14	**Rh16**	Cs_2_CO_3_, 0.2 M, 1 mol % Rh, 72 h	58	6.6:1

a Loading refers to monomeric CpRh. TFE = trifluoroethanol. Concentration is 0.1 M.

b Determined by qNMR with 1,3,5-trimethoxybenzene as an internal standard.

c As reported.^52^

d Isolated yield.

In general, Cp cobalt­(III)-catalyzed reactions still suffer from low TONs. Typically, the majority of transformations using CpCo complexes proceed with catalyst loadings of 10 mol %,^11b^ and examples below 5 mol % are relatively rare.^23f,53^ Current sustainability efforts aim to trade the use of precious noble metals for their earth-abundant 3d-congeners, such as cobalt. In this regard, one of the necessary developments, aside from mimicking and expanding rhodium’s reactivity patterns, lies in matching its efficiency in terms of achieving high catalyst turnover. Therefore, we benchmarked our 1,2,3-Cp ligand scaffold for the cobalt-catalyzed C–H annulation process of *N*-chlorobenzamide **26** with styrene (Table 3).^54^ The synthesis of dihydroisoquinolone **28** was reported with Cp*Co in high yield but with a meager TON of 9 (Entry 1). We developed an enantioselective version of this reaction employing a chiral Cp^x^ cobalt complex and were able to lower the catalyst loading to 5 mol %.^23a^ Applying these conditions, Cp*Co at 10 mol % loading performed poorly, furnishing **28** in only 28% yield (Entry 2). In contrast, trisubstituted complex **Co2** provided a 77% yield with only 5 mol % of cobalt (Entry 3). While decreasing the catalyst loading to 2 mol %, a good yield was maintained (Entry 4). A further decrease of the loading to 1 mol % still led to a higher TON of 46, although a yield drop occurred (Entry 5). The reason lies in competitive consumption of **26** through a Hofmann rearrangement leading to substantial amounts of side product **29**. Switching to nonafluoro-*tert*-butanol (NFTB) as the solvent restored productive conversion toward dihydroisoquinolone **28** (79%, Entry 6). In this solvent, even at an impressive 0.5 mol % of **Co2** monomer, no erosion in reactivity occurred and the product was isolated in 74% yield, corresponding to a TON of 148 (Entry 7). Upon an additional 5-fold decrease in loading to 0.1 mol %, we observed the current catalytic limits, yet an improved TON of 180 was reached (Entry 8). In sum, we were able to substantially improve the catalytic efficiency in this transformation with a 20-fold increase in TON by changing the catalyst from Cp*Co to **Co2**. It sets an example for earth-abundant metal catalysis, showing that with the right ligand, attractive turnover numbers can be realized.

**Table 3:**
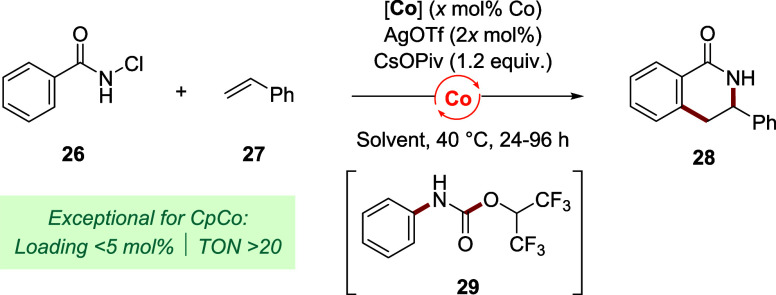
Catalytic Efficiency of a 1,2,3-Cp Ligand in a Co­(III)-Catalyzed C–H Annulation: Access to High TONs

Entry	[Co]	mol % Co^a^	solvent^b^,^c^	% yield^d^	TON
1	Cp*Co	10	TFE	88^e^	9
2	Cp*Co	10	HFIP	28^f^	3
3	**Co2**	5	HFIP	77^f^	15
4	**Co2**	2	HFIP	73^f^	37
5	**Co2**	1	HFIP	46^f^	46
6	**Co2**	1	NFTB	79	79
7	**Co2**	0.5	NFTB	74^g^	148
8	**Co2**	0.1	NFTB	18	180

a Loading refers to monomeric CpCo.

b Concentration is 0.1 M (Entries 2–4), 0.2 M (Entries 1, 5–6), or 0.4 M (Entries 7 and 8). HFIP = hexafluoroisopropanol.

c Time is 24 h (Entries 2–4), 36 h (Entry 1), 48 h (Entries 5–7), or 96 h (Entry 8).

d Determined by qNMR with ethylene carbonate as an internal standard.

e As reported,^54^ using KOAc at 25 °C.

f Parasitic tandem Hofmann rearrangement—HFIP addition of **26** afforded side product **29** in 21% (Entry 2), 5% (Entry 3), 7% (Entry 4), or 39% (Entry 5) yield.

g Isolated yield.

## Conclusion

In conclusion, we have developed a robust, concise, and diversity-oriented synthesis platform to access 1,2,3-trifunctionalized cyclopentadienes, allowing for precise control of each Cp substituent. It uses readily available feedstock materials, is scalable, and comprises operationally straightforward reactions and purifications. The versatility of this unified strategy was amply demonstrated with the flexible incorporation of diverse alkyl and aryl substituents as well as previously elusive or uncommon functionalities, including alkyne, halogen, oxygen, sulfide, and sulfoxide groups. Additional degrees of substitution in the form of tetra- and pentasubstituted derivatives were obtained by installation of either electron-donating or -deficient motifs. The platform acts as a gateway, unlocking a wide chemical space of Cps and allows the free traversal along both steric and electronic axes in a disconnected way.

Complexation of the 1,2,3-Cp scaffold with a selection of catalytically relevant early and late transition metals was demonstrated. The individual Cps were parametrized with respect to their stereoelectronic environment via the corresponding CpRh­(III) phosphite species. In exemplary benchmark catalytic transformations, the cobalt and rhodium complexes outperformed existing Cp ligands with respect to their individual reactivity, regioselectivity, and catalyst loading. Regarding catalytic turnover, an example is set for earth-abundant 3d-metal-catalyzed C–H annulations, with a 1,2,3-Cp cobalt complex achieving a TON of 180. We believe that the outlined synthesis platform and, in consequence, the enabled highly tunable Cp-bearing complexes will spur the development of new catalytic transformations showcasing reactivity and selectivity levels that outperform current catalysts. Application of the 1,2,3-trisubstituted Cp architecture for a modular construction of chiral pentasubstituted Cp^x^ ligands is ongoing.

## Supplementary Material



## Notes

Experimental procedures, characterization data for all new compounds, crystallographic details, and copies of the NMR spectra (PDF)
