# Supplementary material for: Unified Synthesis Platform for 1,2,3-Trisubstituted Cyclopentadienyl Ligands Decouples Sterics from Electronics
Source: J Am Chem Soc. 2026 Jan 9;148(3):3775–87. doi: 10.1021/jacs.5c20631 (PMC12856903; doi:10.1021/jacs.5c20631)

Supporting Information

Unified Synthesis Platform for 1,2,3-Trisubstituted Cyclopentadienyl Ligands Decouples Sterics from Electronics

Bram Van Den Bossche,[a] *and Nicolai Cramer*[a]*

[a] Laboratory of Asymmetric Catalysis and Synthesis (LCSA),
Instititute of Chemical Sciences and Engineering,
École Polytechnique Fédérale de Lausanne (EPFL),
BCH 4305, Avenue F.-A. Forel 2, 1015 Lausanne, Switzerland.

* Corresponding author. E-mail: nicolai.cramer@epfl.ch
Homepage: https://www.epfl.ch/labs/lcsa/

Table of Contents

1. General Methods S3

2. Synthesis of Cyclopentenones S5

3. Synthesis of 1,2,3-Trisubstituted Cps S26

4. Diversification of 1,2,3-Cps........ S46

4.1 Diels-Alder Cycloaddition towards Norbornadienes S46

4.2 Synthesis of Tetrasubstituted Cp Derivatives........ S47

5. Synthesis of Cp Metal Complexes S49

5.1 Cp Cobalt, Iridium, Ruthenium, and Titanium Complexes S49

5.2 Cp Rhodium Complexes........ S53

5.3 Cp Rhodium Phosphite Adducts S63

6. Benchmark Catalytic Transformations........ S67

6.1 Ir-catalyzed C(sp^3)-H Amidation of Oximes S67

6.2 Ti-catalyzed Cross-selective Acyloin-type Condensation S67

6.3 Rh-catalyzed C-H Annulation for 2-Substituted Indolines........ S68

6.4 Rh-catalyzed Regioselective Pyridine Synthesis S69

6.5 Co-catalyzed C-H Annulation for Dihydroisoquinolones S71

7. X-ray Crystallographic Data S72

8. References S84

9. NMR spectra S86

1. General Methods

Experimental procedures, solvents, and reagents

All reactions were carried out under an atmosphere of nitrogen in flame-dried or oven-dried glassware with magnetic stirring, and using Schlenk techniques or inside a MBraun nitrogen-filled glovebox, unless otherwise indicated. The following solvents were purified by an Innovative Technology Solvent Delivery System: dichloromethane (DCM), diethyl ether, acetonitrile, toluene, and tetrahydrofuran (THF). Solvents, that were used e.g. for cobalt complexation, were additionally degassed by freeze-pump-thaw technique (3 x) before being stored under 4 Å molecular sieves in a nitrogen-filled glovebox. Solvents, that were used e.g. in palladium-catalyzed cross-couplings, were degassed by bubbling nitrogen for at least 15 minutes (and at least 2 hours for water) under stirring. Chemicals, and other solvents than the ones mentioned *in supra*, were used as obtained from the suppliers, unless otherwise indicated.

Thin-Layer Chromatography (TLC)

Analytical thin-layer chromatography was performed with commercial glass plates coated with 0.25 mm silica gel (E. Merck, Kieselgel 60 F_{254}). Compounds were either visualized under UV-light at 254 nm, or by dipping the plates in an aqueous potassium permanganate solution, an ethanolic phosphomolybdic acid (PMA) stain, an ethanolic vanillin stain, or an aqueous ceric ammonium molybdate (CAM) stain; afterwards all these stains were developed by heating. Retention factors (R_f) were determined and are reported with indication of the used solvent mixture. Preparative thin-layer chromatography (Prep. TLC) for purification of samples up to 20 mg per plate was performed using the exact set-up as for analytical TLC. Compounds were visualized under UV-light at 254 nm.

Flash Column Chromatography (FCC)

Flash column chromatography was performed with SiliCycle *SiliaFlash P60* silica gel (40-63 µm) or neutral alumina (aluminum oxide, activated, neutral, Brockmann I activity), and using pressurised air during elution. The crude products were loaded onto the column either as coated on silica (dry loading, by evaporating *in vacuo* a DCM solution with suspended silica gel) or as solution in a minimal emount of eluent (wet loading).

Nuclear Magnetic Resonance Spectroscopy (NMR)

Proton nuclear magnetic resonance (^{1}H NMR) data were acquired on a Bruker *AVANCEIII-400* (400 MHz), Bruker *AVANCENEO-500* (500 MHz), Bruker *AVANCEIIIHD-600* (600 MHz), or Bruker *AVANCEII-800* (800 MHz) spectrometer at 298 K, unless otherwise indicated. Chemical shifts (δ) are reported in parts per million (ppm) relative to incompletely deuterated $CDCl_3$ (s, 7.26 ppm), C_6D_6 (s, 7.16 ppm), CD_2Cl_2 (t, 5.32 ppm, J = 1.1 Hz), or MeOD (p, 3.31 ppm, J = 1.7 Hz). Splitting patterns are designated as s, singlet; d, doublet; t, triplet; q, quartet; p, pentet; h, hextet; hept, heptet; dd, doublet of doublets; m, multiplet; br, broad; app, apparent; or combinations thereof. For quantitative ^{1}H NMR (qNMR) experiments, the spectra were recorded with a relaxation delay (d_1) of 10 seconds. TraceCERT® internal standards (1,3,5-trimethoxybenzene, ethylene carbonate, dimethylsulfone) were purchased from Sigma-Aldrich and used as obained. Proton-decoupled carbon-13 nuclear magnetic resonance (^{13}C{^{1}H} NMR) data were acquired on a Bruker *AVANCEIII-400* (101 MHz), Bruker *AVANCENEO-500* (126 MHz), Bruker

AVANCEIIIHD-600 (151 MHz), or Bruker *AVANCEII-800* (201 MHz) spectrometer at 298 K, unless otherwise indicated. Chemical shifts are reported in ppm relative to $CDCl_3$ (77.16 ppm), C_6D_6 (128.06 ppm), or CD_2Cl_2 (53.84 ppm). If symmetric carbons are present, they are listed as one signal. If an apparent multiplicity is observed due to coupling with other nuclei (e.g. ^{19}F, ^{31}P, ^{103}Rh), the splitting pattern and coupling constant are provided. Proton-decoupled fluorine-19 nuclear magnetic resonance ($^{19}F\{^1H\}$ NMR) data were acquired on a Bruker *AVANCEIII-400* (376 MHz) spectrometer at 298 K, unless otherwise indicated. Proton-decoupled phosphorus-31 nuclear magnetic resonance ($^{31}P\{^1H\}$ NMR) data were acquired on a Bruker *AVANCEIII-400* (162 MHz) spectrometer at 298 K, unless otherwise indicated.

Infrared Spectroscopy (IR)

Infrared (IR) data were recorded on a Bruker *Alpha-P* FT-IR spectrometer. Absorbance frequencies are reported in reciprocal centimeters (cm^{-1}). Compounds were applied in solution and, after evaporation of the volatile solvent, measured as a thin film. Relative signal intensities are designated as s, strong; m, medium; w, weak.

High Resolution Mass Spectrometry (HRMS)

HRMS measurements were performed on an Agilent LC-MS TOF (multimode: ESI + APCI), Waters Xevo G2-S QTOF (ESI, APPI, or APCI), Exploris 240 (Sicrit plasma), or Thermo Orbitrap Elite (nanochip-ESI) mass spectrometer. High resolution mass values of the indicated ion are given in *m/z*. For the ion is provided a bruto formula, as well as an indication of the added/removed ions or molecules compared to the parent molecule M.

Melting point determination (M.p.)

Melting points were measured on a Büchi *B-540* apparatus and are uncorrected. They are represented as a range, indicating the temperatures at which the melting proces started and ended. Melting points were determined for solids only, and not for foams.

X-ray Crystallographic Analysis (XRD)

Single crystal X-ray analysis was performed by Dr. F. Fadaei-Tirani at the EPFL in Lausanne. The employed apparatus and applied experimental methodology is described for each crystal at the end of the Supporting Information, together with the obtained crystal data. For each structure, the crystallographic data has been deposited at the Cambridge Crystallographic Data Center (CCDC). Using the CCDC reference numbers provided, copies of the data can be obtained online and free of charge.

Author Contributions

BVDB designed and performed all the experiments and analyzed the results. Both authors conceived and conceptualized the project, interpreted the results, and performed the writing and revision of the manuscript. NC provided funding and resources.

No generative AI tools (ChatGPT, Grammarly, etc.) were used at any stage during the preparation or revision of the main manuscript or the Supporting Information.

2. Synthesis of Cyclopentenones

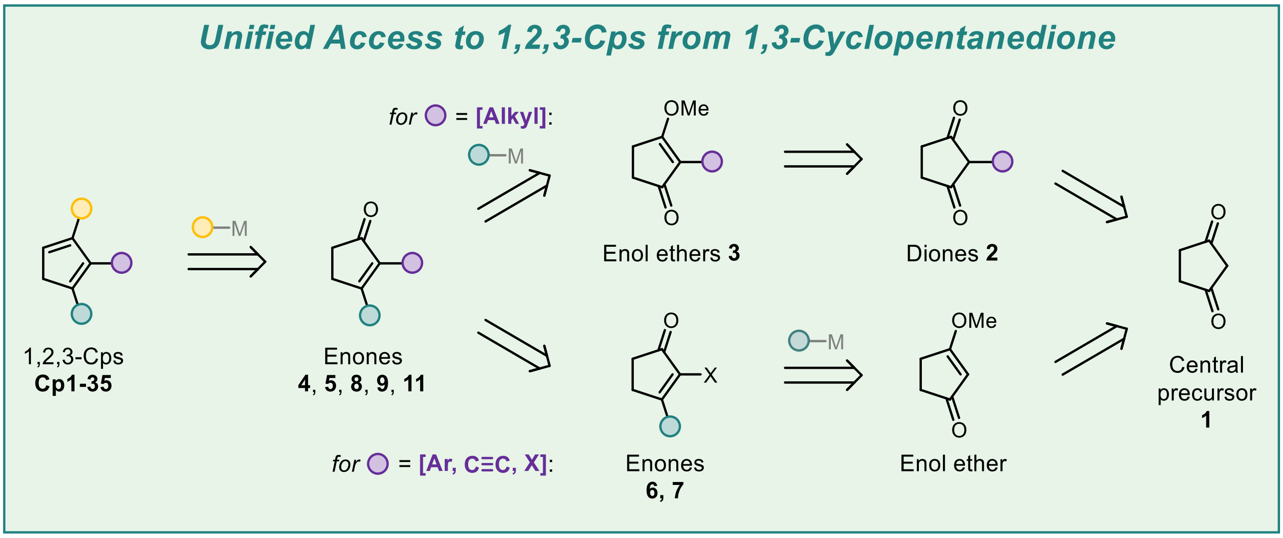

Scheme S1. General overview of the unified synthetic access to diverse 1,2,3-trisubstituted cyclopentadienes.

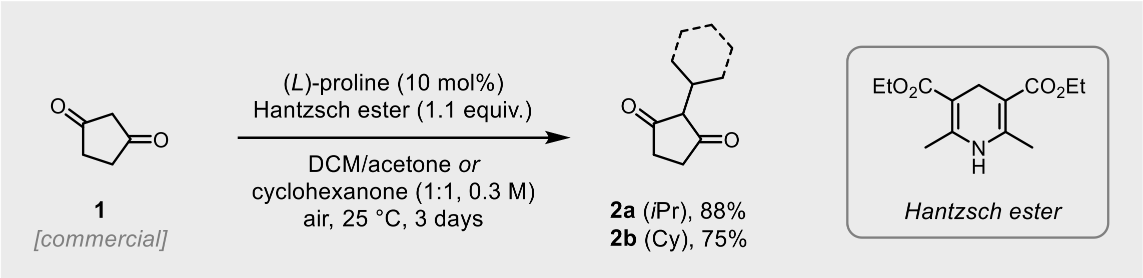

Scheme S2. Organocatalyzed reductive Knoevenagel condensation of 1,3-cyclopentanedione.

2-Isopropylcyclopentane-1,3-dione (**2a**)

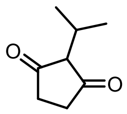

According to the following adaptatation of a reported procedure (**Scheme S2**),[1] 2-isopropylcyclopentane-1,3-dione **2a** was obtained with the characterization data matching those reported.

A round-bottom flask was charged with commercial cyclopentane-1,3-dione **1** (981 mg, 10.00 mmol, 1.0 equiv.) and diethyl 2,6-dimethyl-1,4-dihydropyridine-3,5-dicarboxylate (Hantzsch ester, 2843 mg, 1.1 equiv.). The solids were dissolved in DCM (1.5 mL/mmol), after which acetone was added (1.5 mL/mmol). Next, the (*L*)-proline organocatalyst (116 mg, 10 mol%) was added, the flask was sealed, and the mixture was stirred under air at room temperature (25 °C) for 3 days. The crude reaction mixture was directly purified by flash column chromatography on silica gel (dry loading, 10 cm column height, gradient: pentane/EtOAc = 50:50 → 0:100), affording 2-isopropylcyclopentane-1,3-dione **2a** (1236 mg, 8.81 mmol, 88% yield) as a pale yellow solid.

Notably, the protocol was repeated successfully on a larger scale (56.00 mmol), affording 6869 mg (49.00 mmol, 87% yield) of **2a**.

^{1}H NMR (400 MHz, $CDCl_3$ + 5 drops MeOD) δ = 2.66 (hept, *J* = 6.9 Hz, 1H), 2.35 (s, 4H), 1.08 (d, *J* = 7.0 Hz, 7H) ppm; **R_f** (EtOAc) = 0.36.

2-Cyclohexylcyclopentane-1,3-dione (**2b**)

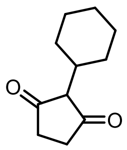

According to the procedure described for compound **2a** (**Scheme S2**), but using cyclohexanone (1.5 mL/mmol) instead of acetone, 2-cyclohexylcyclopentane-1,3-dione **2b** (1347 mg, 7.47 mmol, 75% yield) was obtained as a pale orange solid with the characterization data matching those previously reported.[1] Purification was performed by flash column chromatography on silica gel (dry loading, 15 cm column height, gradient: pentane/EtOAc = 50:50 → 0:100).

^{1}H NMR (400 MHz, MeOD) δ = 2.43 (s, 4H), 2.43 – 2.30 (m, 1H), 1.81 – 1.62 (m, 5H), 1.51 – 1.41 (m, 2H), 1.36 – 1.19 (m, 3H) ppm; **R_f** (EtOAc) = 0.40.

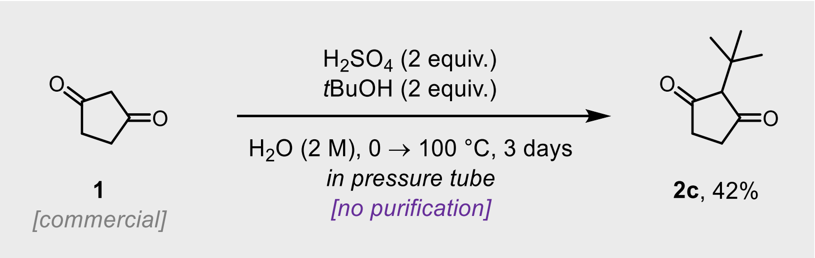

Scheme S3. Installation of a *tert*-butyl group on 1,3-cyclopentanedione *via* S_N1 reaction.

2-(*tert*-Butyl)cyclopentane-1,3-dione (**2c**)

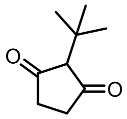

According to the following procedure (**Scheme S3**), 2-(*tert*-butyl)cyclopentane-1,3-dione **2c** was obtained with the characterization data matching those previously reported.[2] Notably, it allows an expedient synthesis of **2c** (1 step, 42% yield) compared to the literature protocol (7 steps, 5% overall yield) that starts from commercial 1-(trimethylsiloxy)cyclopentene.[2,3]

A screw-cap pressure tube was charged with commercial cyclopentane-1,3-dione **1** (981 mg, 10.00 mmol, 1.0 equiv.). Water (0.5 mL/mmol) was added, and the brown slurry was cooled to 0 °C under stirring. Then, concentrated sulfuric acid (1.13 mL, 2.0 equiv.) was slowly added, after which *tert*-butanol (1.91 mL, 2.0 equiv.) was added. The ice bath was removed, and the tube was closed by screw-cap after flushing it with argon. The dark brown solution was stirred in a heating block at 100 °C for 3 days. After cooling to room temperature (25 °C), the black reaction mixture was transferred to a separation funnel, diluted with water, and the resulting black-green emulsion was extracted with EtOAc (3 x). The combined organic layers were washed with water and then with sat. aq. $NaHCO_3$ (3x), which removed most colour from the organic phase. After a final wash with brine, the light-pink organic phase was dried over $MgSO_4$ and passed through a short pad of silica gel (10 cm height) with EtOAc as eluent. The solvent was removed *in vacuo*, affording 2-(*tert*-butyl)cyclopentane-1,3-dione **2c** (651 mg, 4.22 mmol, 42% yield) as a white crystalline solid, and in sufficient purity to be used in the next step without further purification.

Notably, the protocol was repeated successfully on a larger scale (30.00 mmol), affording 1767 mg (11.46 mmol, 38% yield) of **2c**.

^{1}H NMR (400 MHz, MeOD) δ = 2.38 (s, 4H), 1.24 (s, 9H) ppm; **R$_f$** (EtOAc) = 0.34.

<u>General Procedure 1</u> – Acid-catalyzed Conversion of Diones into Enol Ethers.

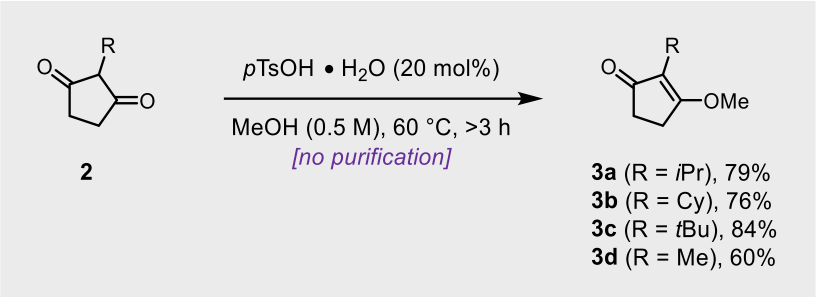

Scheme S4. Acid-catalyzed conversion of diones into enol ethers.

An oven-dried microwave vial was charged with dione **2** (1.0 equiv.) and 4-toluenesulfonic acid monohydrate (PTSA, 20 mol%), capped, and placed under an atmosphere of nitrogen by Schlenk technique. Anhydrous methanol (2 mL/mmol) was added, and the reaction mixture was stirred in a heating block at 60 °C for 3 hours (but can be left overnight). After cooling to room temperature (25 °C), the mixture was diluted with EtOAc (20 mL/mmol) and washed with consecutively aq. NaOH solution (1 M, 10 mL/mmol), sat. aq. $NaHCO_3$ solution (10 mL/mmol), and brine. The organic phase was dried over $MgSO_4$, filtered, and concentrated under reduced pressure, affording enol ether **3** in sufficient purity to be used in the next step without further purification.

<u>2-Isopropyl-3-methoxycyclopent-2-en-1-one</u> (**3a**)

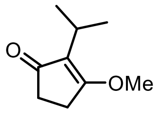

According to *General Procedure 1*, starting from dione **2a** (4.22 mmol), enol ether **3a** (1067 mg, 6.92 mmol, 79% yield) was obtained as a yellow oil with the characterization data matching those previously reported,[4] and in sufficient purity to be used in the next step without further purification.

Notably, the protocol was repeated successfully on a larger scale (49.00 mmol), affording 5548 mg (35.98 mmol, 73% yield) of **3a**.

^{1}H NMR (400 MHz, $CDCl_3$) δ = 3.91 (s, 3H), 2.74 (hept, *J* = 7.1 Hz, 1H), 2.64 – 2.59 (m, 2H), 2.42 – 2.36 (m, 2H), 1.10 (d, *J* = 7.0 Hz, 6H) ppm; **R$_f$** (EtOAc) = 0.48.

2-Cyclohexyl-3-methoxycyclopent-2-en-1-one (**3b**)

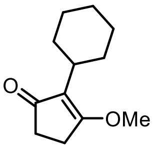

According to *General Procedure 1*, starting from dione **2b** (7.40 mmol), enol ether **3b** (1094 mg, 5.63 mmol, 76% yield) was obtained as a light-orange solid, and in sufficient purity to be used in the next step without further purification.

^{1}H NMR (400 MHz, $CDCl_3$) δ = 3.90 (s, 3H), 2.65 – 2.58 (m, 2H), 2.44 – 2.32 (m, 3H), 1.76 – 1.58 (m, 5H), 1.51 – 1.41 (m, 2H), 1.32 – 1.16 (m, 3H) ppm; **^{13}C{^{1}H} NMR** (101 MHz, $CDCl_3$) δ = 204.7, 184.4, 125.3, 56.3, 33.6, 33.1, 29.9, 26.9, 26.1, 24.2 ppm; **IR** (ATR) ṽ = 2922 (m), 2850 (w), 1680 (m), 1609 (s), 1448 (w), 1381 (w), 1368 (w), 1342 (s), 1301 (w), 1275 (m), 1251 (m), 1240 (m), 1166 (w), 1111 (w), 1052 (m), 1028 (w), 982 (w), 934 (w), 588 (w) cm^{-1}; **HRMS** (ESI/APCI) *m/z* = calcd. for $[C_{12}H_{19}O_2]^+$, $[M+H]^+$: 195.1380, found: 195.1381; **R_f** (EtOAc) = 0.57; **M.p.** = 79-80 °C.

2-(*tert*-Butyl)-3-methoxycyclopent-2-en-1-one (**3c**)

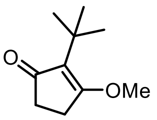

According to *General Procedure 1*, starting from dione **2c** (7.13 mmol), enol ether **3c** (1010 mg, 6.00 mmol, 84% yield) was obtained as a light-orange oil, and in sufficient purity to be used in the next step without further purification.

^{1}H NMR (400 MHz, $CDCl_3$) δ = 3.88 (s, 3H), 2.61 – 2.56 (m, 2H), 2.37 – 2.31 (m, 2H), 1.23 (s, 9H) ppm; **^{13}C{^{1}H} NMR** (101 MHz, $CDCl_3$) δ = 205.0, 183.5, 126.2, 55.9, 34.0, 32.3, 29.2, 24.2 ; **IR** (ATR) ṽ = 2950 (w), 2925 (w), 2861 (w), 1679 (m), 1595 (s), 1482 (w), 1459 (w), 1391 (w), 1341 (s), 1301 (m), 1244 (m), 1165 (w), 1069 (m), 1029 (w), 982 (w), 925 (w), 619 (w), 583 (w) cm^{-1}; **HRMS** (ESI/APCI) *m/z* = calcd. for $[C_{10}H_{17}O_2]^+$, $[M+H]^+$: 169.1223, found: 169.1225; **R_f** (EtOAc) = 0.47.

3-Methoxy-2-methylcyclopent-2-en-1-one (**3d**)

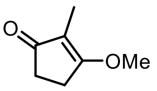

According to *General Procedure 1*, starting from commercial dione **2d** (45.36 mmol), enol ether **3d** (3433 mg, 27.21 mmol, 60% yield) was obtained as a white solid with the characterization data matching those previously reported,[5] and in sufficient purity to be used in the next step without further purification. Notably, the conversion was incomplete, even after multiple days of heating and with the addition of drying agents, such as molecular sieves (4 Å) or anhydrous $MgSO_4$. The ethyl enol ether **3d'** was synthesized with higher conversion.

^{1}H NMR (400 MHz, $CDCl_3$) δ = 3.95 (s, 3H), 2.67 – 2.60 (m, 2H), 2.47 – 2.41 (m, 2H), 1.63 (t, *J* = 1.7 Hz, 3H) ppm; **R_f** (EtOAc) = 0.37.

3-Ethoxy-2-methylcyclopent-2-en-1-one (**3d'**)

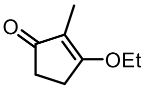

According to the following procedure, enol ether **3d'** was obtained with the characterization data matching those previously reported.[6]

A flame-dried round-bottom flask was charged with commercial dione **2d** (9145 mg, 81.56 mmol, 1.0 equiv.) and 4-toluenesulfonic acid monohydrate (PTSA, 20 mol%), closed with a septum, and placed under an atmosphere of nitrogen by Schlenk technique. Ethanol (1 mL/mmol) was added, and the

reaction mixture was stirred in a heating block at 80 °C for 20 hours. After cooling to room temperature (25 °C), the mixture was diluted with EtOAc (20 mL/mmol) and washed with consecutively aq. NaOH solution (1 M, 10 mL/mmol), sat. aq. $NaHCO_3$ solution (10 mL/mmol), and brine. The organic phase was dried over $MgSO_4$, filtered, and concentrated under reduced pressure, affording enol ether **3d'** (8620 mg, 61.49 mmol, 75% yield) as a white solid, and in sufficient purity to be used in the next step without further purification. Long-term storage was done under nitrogen atmosphere in a freezer at -40 °C.

^{1}H NMR (400 MHz, $CDCl_3$) δ = 4.23 (q, *J* = 7.1 Hz, 2H), 2.66 – 2.61 (m, 2H), 2.46 – 2.42 (m, 2H), 1.64 (t, *J* = 1.7 Hz, 3H), 1.40 (t, *J* = 7.1 Hz, 3H) ppm.

General Procedure 2 ***– Nucleophilic Addition on Enol Ethers towards Enones.***

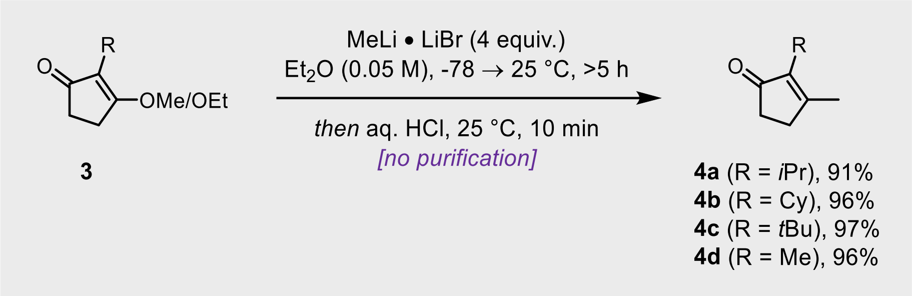

Scheme S5. Nucleophilic addition on enol ethers towards enones.

At -78 °C (acetone/dry ice bath) and under an atmosphere of nitrogen, a methyllithium lithium bromide complex solution (1.5 M in Et_2O, 4.0 equiv.) was added dropwise to a solution of enol ether **3** (1.0 equiv.) in anhydrous Et_2O (20 mL/mmol). The reaction mixture was allowed to slowly warm up in the cold bath to room temperature (25 °C) under stirring for 5 hours (but can be left overnight). It was quenched with sat. aq. NH_4Cl solution, then aq. HCl solution (1 M, 3 mL/mmol) was added, and the mixture was stirred vigorously for another 10 min, after which it was extracted with Et_2O (2x). The combined organic layers were sequentially washed with sat. aq. $NaHCO_3$ solution and brine, dried over $MgSO_4$, and filtered. The solvent was removed *in vacuo*, affording enone **4** in sufficient purity to be used in the next step without further purification.

2-Isopropyl-3-methylcyclopent-2-en-1-one (**4a**)

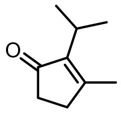

According to *General Procedure 2*, starting from enol ether **3a** (35.97 mmol), cyclopentenone **4a** (4507 mg, 32.61 mmol, 91% yield) was obtained as a light-yellow oil with the characterization data matching those previously reported,[7] and in sufficient purity to be used in the next step without further purification.

^{1}H NMR (400 MHz, $CDCl_3$) δ = 2.79 (hept, *J* = 7.1 Hz, 1H), 2.48 – 2.41 (m, 2H), 2.34 – 2.28 (m, 2H), 2.06 (s, 3H), 1.16 (d, *J* = 7.1 Hz, 6H) ppm; **R_f** (pentane/EtOAc, 1:1) = 0.69.

2-Cyclohexyl-3-methylcyclopent-2-en-1-one (**4b**)

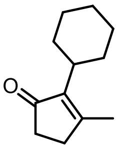

According to *General Procedure 2*, starting from enol ether **3b** (5.57 mmol), cyclopentenone **4b** (949 mg, 5.32 mmol, 96% yield) was obtained as a light-yellow solid.

^{1}H NMR (400 MHz, $CDCl_3$) δ = 2.47 – 2.36 (m, 3H), 2.33 – 2.27 (m, 2H), 2.07 (s, 3H), 1.81 – 1.62 (m, 5H), 1.49 – 1.40 (m, 2H), 1.30 – 1.19 (m, 3H) ppm; **^{13}C{^{1}H} NMR** (101 MHz, $CDCl_3$) δ = 209.7, 169.4, 144.1, 35.4, 34.7, 31.9, 30.1, 26.9, 26.1, 17.8 ppm; **IR** (ATR) ṽ = 2921 (m), 2850 (m), 1687 (s), 1633 (m), 1444 (w), 1408 (w), 1382 (w), 1331 (w), 1295 (w), 1274 (w), 1230 (w), 1169 (w), 1076 (w), 972 (w), 889 (w), 806 (w), 576 (w), 526 (w) cm^{-1}; **HRMS** (ESI/APCI) *m/z* = calcd. for $[C_{12}H_{19}O]^+$, $[M+H]^+$: 179.1430, found: 179.1431; **R_f** (pentane/EtOAc, 9:1) = 0.31; **M.p.** = 47-48 °C.

2-(*tert*-Butyl)-3-methylcyclopent-2-en-1-one (**4c**)

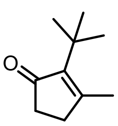

According to *General Procedure 2*, starting from enol ether **3c** (8.11 mmol), cyclopentenone **4c** (1204 mg, 7.91 mmol, 97% yield) was obtained as a light-orange oil with the characterization data matching those previously reported.[8]

^{1}H NMR (400 MHz, $CDCl_3$) δ = 2.43 – 2.39 (m, 2H), 2.29 – 2.25 (m, 2H), 2.19 (s, 3H), 1.27 (s, 9H) ppm; **R_f** (pentane/EtOAc, 9:1) = 0.29.

2,3-Dimethylcyclopent-2-en-1-one (**4d**)

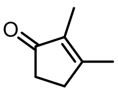

According to *General Procedure 2*, starting from enol ether **3d'** (27.15 mmol), cyclopentenone **4d** (2876 mg, 26.11 mmol, 96% yield) was obtained as a light-yellow oil with the characterization data matching those previously reported.[9] Notably, this compound is also commercial (CAS 1121-05-7).

^{1}H NMR (400 MHz, $CDCl_3$) δ = 2.52 – 2.45 (m, 2H), 2.41 – 2.33 (m, 2H), 2.07 – 2.01 (m, 3H), 1.72 – 1.65 (m, 3H) ppm; **R_f** (pentane/EtOAc, 3:1) = 0.34.

3-Isopropyl-2-methylcyclopent-2-en-1-one (**5a**)

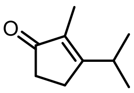

According to the following procedure, cyclopentenone **5a** was obtained with the characterization data matching those previously reported (although incomplete,[10] and therefore a full set of data is provided below).

At -78 °C (acetone/dry ice bath) and under an atmosphere of nitrogen, an isopropyllithium solution (0.7 M in pentane, 9.91 mL, 3.0 equiv.) was added dropwise to a solution of enol ether **3d'** (324 mg, 2.31 mmol, 1.0 equiv.) in anhydrous Et_2O (20 mL/mmol). The reaction mixture was allowed to slowly warm up in the cold bath to room temperature (25 °C) under stirring for 14 hours. It was quenched with sat. aq. NH_4Cl solution, then aq. HCl solution (1 M, 3 mL/mmol) was added, and the mixture was stirred vigorously for another 10 min, after which it was extracted with Et_2O (2x). The combined organic layers were sequentially washed with sat. aq. $NaHCO_3$ solution and brine, dried over $MgSO_4$, filtered, and concentrated under reduced pressure. The residue was purified by flash column chromatography on

silica gel (wet loading with pentane/DCM = 20:1, 18 cm column height, isocratic: pentane/EtOAc = 5:1), affording cyclopentenone **5a** (161 mg, 1.16 mmol, 50% yield) as a light-yellow liquid. The moderate yield is due to competitive enolization of **3d'** rather than addition by the organolithium reagent.

^{1}H NMR (400 MHz, $CDCl_3$) δ = 3.03 (hept, *J* = 6.9 Hz, 1H), 2.51 – 2.46 (m, 2H), 2.38 – 2.33 (m, 2H), 1.70 (t, *J* = 2.0 Hz, 3H), 1.12 (d, *J* = 6.9 Hz, 6H) ppm; **^{13}C{^{1}H} NMR** (101 MHz, $CDCl_3$) δ = 210.7, 178.8, 134.8, 34.0, 29.5, 24.9, 20.4, 8.0 ppm; **IR** (ATR) ṽ = 2964 (w), 2923 (w), 1698 (s), 1640 (m), 1468 (w), 1446 (w), 1385 (w), 1342 (w), 1300 (w), 1201 (w), 1075 (w), 1032 (w), 578 (w) cm^{-1}; **HRMS** (ESI/QTOF) *m/z* = calcd. for $[C_9H_{15}O]^+$, $[M+H]^+$: 139.1117, found: 139.1112; **R_f** (pentane/EtOAc, 6:1) = 0.28.

<u>3-(3,5-Bis(trifluoromethyl)phenyl)-2-methylcyclopent-2-en-1-one</u> (**5b**)

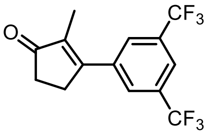

According to the following procedure, cyclopentenone **5b** was obtained.

At 0 °C (ice bath) and under an atmosphere of nitrogen, a (3,5-bis(trifluoromethyl)-phenyl)magnesium bromide solution (0.5 M in THF, 16.80 mL, 3.0 equiv.) was added dropwise to a solution of enol ether **3d'** (391 mg, 2.79 mmol, 1.0 equiv.) in anhydrous Et_2O (20 mL/mmol). The ice bath was removed, and the reaction was stirred at room temperature (25 °C) for 24 hours. It was quenched with sat. aq. NH_4Cl solution, then aq. HCl solution (1 M, 5 mL/mmol) was added, and the mixture was stirred vigorously for another 10 min, after which it was extracted with Et_2O (2x). The combined organic layers were sequentially washed with sat. aq. $NaHCO_3$ solution and brine, dried over $MgSO_4$, filtered, and concentrated under reduced pressure. The residue was purified by flash column chromatography on silica gel (dry loading, 18 cm column height, gradient: pentane/EtOAc = 9:1 → 5:1), affording cyclopentenone **5b** (700 mg, 2.27 mmol, 81% yield) as a brown crystalline solid.

^{1}H NMR (400 MHz, $CDCl_3$) δ = 7.92 (s, 3H), 2.99 – 2.94 (m, 2H), 2.63 – 2.60 (m, 2H), 1.97 (t, *J* = 2.1 Hz, 3H) ppm; **^{13}C{^{1}H} NMR** (101 MHz, $CDCl_3$) δ = 208.8, 162.4, 139.4, 138.7, 132.4 (q, *J* = 33.5 Hz), 127.6 (q, *J* = 3.6 Hz), 123.2 (q, *J* = 272.9 Hz), 123.0 (hept, *J* = 3.9 Hz), 34.1, 29.4, 9.9 ppm; **^{19}F{^{1}H} NMR** (376 MHz, $CDCl_3$) δ = -62.95 ppm; **IR** (ATR) ṽ = 1706 (m), 1375 (w), 1320 (m), 1279 (s), 1175 (m), 1132 (s), 1092 (w), 1014 (w), 900 (w), 871 (w), 845 (w), 704 (w), 682 (w) cm^{-1}; **HRMS** (Sicrit plasma/LTQ-Orbitrap) *m/z* = calcd. for $[C_{14}H_{11}F_6O]^+$, $[M+H]^+$: 309.0709, found: 309.0708; **R_f** (pentane/EtOAc, 6:1) = 0.25; **M.p.** = 50-51 °C.

<u>3-((3,5-Difluorophenyl)ethynyl)-2-methylcyclopent-2-en-1-one</u> (**5c**)

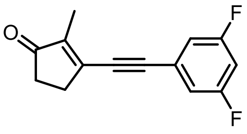

According to the following procedure, cyclopentenone **5c** was obtained.

At -78 °C (acetone/dry ice bath) and under an atmosphere of nitrogen, *n*-butyllithium solution (1.6 M in hexanes, 4.62 mL, 3.0 equiv.) was added dropwise to a solution of 1-ethynyl-3,5-difluorobenzene (1021 mg, 3.0 equiv.) in anhydrous Et_2O (10 mL/mmol). The cold bath was removed, and the reaction mixture was stirred at room temperature (25 °C) for 15 min. In another flask, at -78 °C (acetone/dry ice bath) and under an atmosphere of nitrogen, the resulting alkynyllithium solution was added dropwise to a solution of enol ether **3d'** (345 mg, 2.46 mmol, 1.0 equiv.) in anhydrous Et_2O (10 mL/mmol). The reaction mixture was allowed to slowly warm up in the

cold bath to room temperature (25 °C) under stirring for 18 hours. It was quenched with sat. aq. NH_4Cl solution, then aq. HCl solution (1 M, 5 mL/mmol) was added, and the mixture was stirred vigorously for another 10 min, after which it was extracted with Et_2O (2x). The combined organic layers were sequentially washed with sat. aq. $NaHCO_3$ solution and brine, dried over $MgSO_4$, filtered, and concentrated under reduced pressure. The residue was purified by flash column chromatography on silica gel (dry loading, 22 cm column height, gradient: pentane/EtOAc = 10:1 → 7:1), affording cyclopentenone **5c** (262 mg, 1.13 mmol, 46% yield) as a yellow solid.

^{1}H NMR (400 MHz, $CDCl_3$) δ = 7.08 – 7.01 (m, 2H), 6.87 (tt, *J* = 8.9, 2.4 Hz, 1H), 2.75 – 2.70 (m, 2H), 2.50 – 2.46 (m, 2H), 1.92 (t, *J* = 2.1 Hz, 3H) ppm; **^{13}C{^{1}H} NMR** (101 MHz, $CDCl_3$) δ = 208.8, 162.9 (dd, *J* = 249.9, 13.3 Hz), 148.7, 146.1, 124.8 (t, *J* = 11.7 Hz), 114.9 (dd, *J* = 19.5, 7.7 Hz), 105.7 (t, *J* = 25.2 Hz), 102.3 (t, *J* = 4.1 Hz), 86.6, 34.1, 30.0, 10.0 ppm; **^{19}F{^{1}H} NMR** (376 MHz, $CDCl_3$) δ = -108.90 ppm; **IR** (ATR) ṽ = 3050 (w), 2204 (w), 1689 (s), 1620 (m), 1587 (m), 1444 (w), 1432 (m), 1315 (m), 1128 (w), 1108 (w), 1084 (w), 991 (m), 866 (w), 852 (m), 667 (w) cm^{-1}; **HRMS** (ESI/QTOF) *m/z* = calcd. for $[C_{14}H_{11}F_2O]^+$, $[M+H]^+$: 233.0772, found: 233.0774; **R_f** (pentane/EtOAc, 9:1) = 0.23; **M.p.** = 127-128 °C.

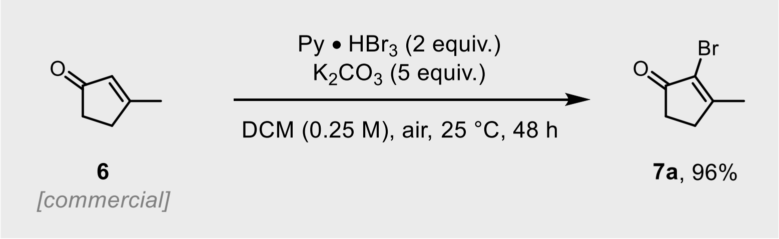

Scheme S6. Tandem dibromination-elimination towards a 2-bromo-substituted enone.

2-Bromo-3-methylcyclopent-2-en-1-one (**7a**)

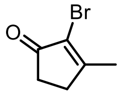

According to the following adaptation of a reported procedure (**Scheme S6**),[11] cyclopentenone **7a** was obtained with the characterization data matching those reported.

Under air, commercial 3-methylcyclopent-2-en-1-one **6** (971 mg, 1.00 mL, 10.10 mmol, 1.0 equiv.) was dissolved in anhydrous DCM (4 mL/mmol). Next, potassium carbonate (6980 mg, 5.0 equiv.) and pyridinium perbromide (technical grade, 90% purity, 7179 mg, 2.0 equiv.) were added sequentially. The suspension was stirred under air at room temperature (25 °C) for 2 days. The reaction mixture was carefully quenched with sat. aq. $NaHCO_3$ solution and then filtered through a pad of celite (3 cm) with DCM as eluent. After dilution with water, the mixture was extracted with DCM (3x). The combined orange organic layers were sequentially washed with water and brine, dried over $MgSO_4$, and concentrated under reduced pressure. The residue was purified by flash column chromatography on silica gel (dry loading, 15 cm column height, isocratic: pentane/EtOAc = 4:1), affording 2-bromo-3-methylcyclopent-2-en-1-one **7a** (1702 mg, 9.72 mmol, 96% yield) as a brown solid. Notably, the protocol was repeated successfully on a larger scale (57.58 mmol), affording 9.42 g (53.83 mmol, 93% yield) of **7a**.

^{1}H NMR (400 MHz, $CDCl_3$) δ = 2.69 – 2.63 (m, 2H), 2.58 – 2.52 (m, 2H), 2.19 (s, 3H) ppm; **R_f** (pentane/EtOAc, 5:1) = 0.21.

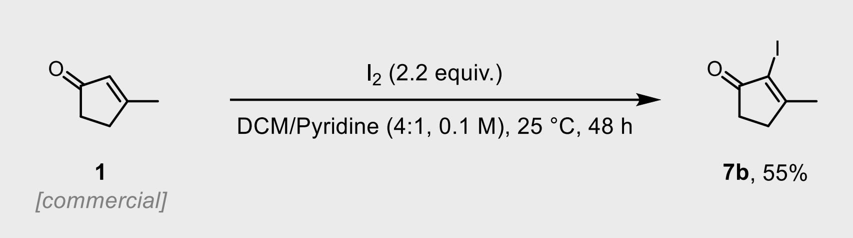

Scheme S7. Tandem diiodination-elimination towards a 2-iodo-substituted enone.

2-Iodo-3-methylcyclopent-2-en-1-one (**7b**)

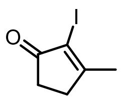

According to the following adaptation of a reported procedure (**Scheme S7**),[12] cyclopentenone **7b** was obtained with the characterization data matching those previously reported.[13]

In a flame-dried two-necked round-bottom flask was made a solution of commercial 3-methylcyclopent-2-en-1-one **6** (4612 mg, 5.00 mL, 47.98 mmol, 1.0 equiv.) in anhydrous DCM (8 mL/mmol) under an atmosphere of nitrogen. Next, pyridine (2 mL/mmol) and iodine (26.79 g, 2.2 equiv.) were added sequentially and under a counterflow of nitrogen. The flask was sealed and stirred at room temperature (25 °C) for 2 days. The reaction mixture was quenched with sat. aq. $Na_2S_2O_3$ solution (5 mL/mmol), and extracted with DCM (2x). The combined organic layers were washed with consecutively aq. HCl solution (3 M, 10 mL/mmol), and water (5 mL/mmol). After a final wash with brine, the organic phase was dried over $MgSO_4$, and concentrated under reduced pressure. The residue was purified by flash column chromatography on silica gel (dry loading, 18 cm column height, isocratic: DCM), affording 2-iodo-3-methylcyclopent-2-en-1-one **7b** (5838 mg, 26.30 mmol, 55% yield) as a white solid.

^{1}H NMR (400 MHz, $CDCl_3$) δ = 2.78 – 2.74 (m, 2H), 2.61 – 2.57 (m, 2H), 2.23 (s, 3H) ppm; **R_f** (DCM) = 0.28.

***General Procedure 3* – Suzuki-Miyaura Cross-coupling with a Bromo-substituted Enone.**

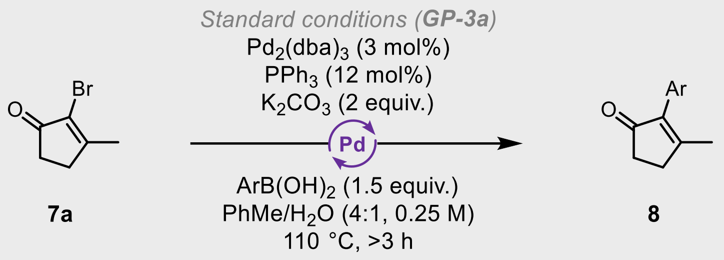

GP	Type of $ArB(OH)_2$	[Pd], ligand	Base	Solvent, *T*
GP-3a	General (ideally electron-rich)	$Pd_2(dba)_3$, PPh_3	K_2CO_3	$PhMe/H_2O$, 110 °C
GP-3b	Hindered (*ortho*-substituted)	$Pd_2(dba)_3$, SPhos	K_3PO_4	Dioxane/H_2O, 80 °C
GP-3c	Protodeboration-prone	XPhos-Pd-G2	K_3PO_4	THF/H_2O, 25 °C

8a (Ar = Ph), 89% *via GP-3a*
8b (Ar = 4-OMe-C_6H_4), 93% *via GP-3a*
8c (Ar = 4-CF_3-C_6H_4), 73% *via GP-3a*
8d (Ar = 4-Ph-C_6H_4), 95% *via GP-3a*
8e (Ar = 4-SO_2Me-C_6H_4), 72% *via GP-3a*
8f (Ar = 3,5-*t*Bu-C_6H_3), 92% *via GP-3a*
8g (Ar = 3,5-CF_3-C_6H_3), 81% *via GP-3a*
8h (Ar = 3,5-F-C_6H_3), 86% *via GP-3a*
8i (Ar = 2,6-F-C_6H_3), 97% *via GP-3c*
8j (Ar = 2,6-OMe-C_6H_3), 75% *via GP-3b*
8k (Ar = 2,6-O*i*Pr-C_6H_3), 69% *via GP-3b*
8l (Ar = 9-Anthracene), 79% *via GP-3b*
8m (Ar = 3,4,5-OMe-C_6H_2), 94% *via GP-3a*
8n (Ar = 7-Benzo[*b*]thiophene), 99% *via GP-3b*
8o (Ar = 4-NO_2-C_6H_4), 83% *via GP-3a*
8p (Ar = 8-Quinoline), 96% *via GP-3b*

Scheme S8. Suzuki-Miyaura cross-coupling with a bromo-substituted enone.

General Procedure 3a: A microwave vial was charged with 2-bromo-3-methylcyclopent-2-en-1-one **7a** (1.0 equiv.), tris(dibenzylideneacetone)dipalladium (3 mol%), triphenylphosphine (12 mol%), potassium carbonate (2.0 equiv.), and the desired boronic acid (1.5 equiv.). After capping the vial and placing it under an atmosphere of nitrogen by Schlenk technique, degassed toluene (3 mL/mmol) and degassed water (0.75 mL/mmol) were added. The reaction mixture was stirred vigorously (to ensure mixing of both phases) in a heating block at 110 °C for 3-24 hours. After cooling to room temperature (25 °C), the mixture was diluted with EtOAc, filtered through celite (2 cm), and washed with consecutively aq. K_3PO_4 solution (2x, *ca.* 0.3 M, 1.5 mL/mmol), water, and brine. The combined organic layers were dried over $MgSO_4$, filtered, and concentrated under reduced pressure. The residue was purified by flash column chromatography on silica gel, affording the corresponding aryl-substituted cyclopentenone **8**.

General Procedure 3b: A microwave vial was charged with 2-bromo-3-methylcyclopent-2-en-1-one **7a** (1.0 equiv.), tris(dibenzylideneacetone)dipalladium (3 mol%), dicyclohexyl(2′,6′-dimethoxy[1,1′-biphenyl]-2-yl)phosphane (SPhos, 12 mol%), potassium phosphate (3.0 equiv.), and the desired sterically hindered boronic acid (1.5 equiv.). After capping the vial and placing it under an atmosphere of nitrogen by Schlenk technique, degassed dioxane (3 mL/mmol) and degassed water (0.75 mL/mmol) were added sequentially. The reaction mixture was stirred vigorously in a heating block at 80 °C for 10-24 hours. After cooling to room temperature (25 °C), the mixture was diluted with EtOAc, filtered through celite (2 cm), and washed with consecutively aq. K_3PO_4 solution (2x, *ca.* 0.3 M), and water. The combined aqueous phases were back-extracted with EtOAc. Next, the combined organic layers were washed with

brine, dried over $MgSO_4$, filtered, and concentrated under reduced pressure. The residue was purified by flash column chromatography on silica gel, affording the corresponding cyclopentenone **8**.

General Procedure 3c: A round-bottom flask was charged with 2-bromo-3-methylcyclopent-2-en-1-one **7a** (1.0 equiv.), chloro(2-dicyclohexylphosphino-2′,4′,6′-triisopropyl-1,1′-biphenyl)[2-(2′-amino-1,1′-biphenyl)]palladium(II) precatalyst (XPhos-Pd-G2, 5 mol%), and the desired protodeboration-prone boronic acid (3.0 equiv.). After sealing the flask and placing it under an atmosphere of nitrogen by Schlenk technique, degassed THF (2 mL/mmol) and a degassed solution of potassium phosphate (2.0 equiv.) in water (4 mL/mmol) were added sequentially. The reaction mixture was stirred vigorously at room temperature (25 °C) for 2 hours. Afterwards, the mixture was diluted with EtOAc, filtered through celite (2 cm), and washed consecutively with water (2 x) and brine. The combined organic layers were dried over $MgSO_4$, filtered, and concentrated under reduced pressure. The residue was purified by flash column chromatography on silica gel, affording the corresponding cyclopentenone **8**.

3-Methyl-2-phenylcyclopent-2-en-1-one (**8a**)

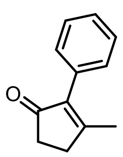

According to *General Procedure 3a*, starting from enone **7a** (3.00 mmol) with commercial phenylboronic acid as the coupling partner and 18 h reaction time, cyclopentenone **8a** (460 mg, 2.67 mmol, 89% yield) was obtained as a yellow oil with the characterization data matching those previously reported.[14] Purification was performed by flash column chromatography on silica gel (dry loading, 15 cm column height, gradient: pentane/EtOAc = 4:1 → 3:1).

^{1}H NMR (400 MHz, $CDCl_3$) δ = 7.45 – 7.37 (m, 2H), 7.36 – 7.25 (m, 3H), 2.70 – 2.62 (m, 2H), 2.60 – 2.52 (m, 2H), 2.22 – 2.16 (m, 3H) ppm; **R_f** (pentane/EtOAc, 3:1) = 0.30.

2-(4-Methoxyphenyl)-3-methylcyclopent-2-en-1-one (**8b**)

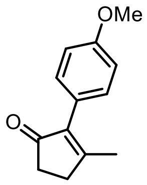

According to *General Procedure 3a*, starting from enone **7a** (2.89 mmol) with commercial (4-methoxyphenyl)boronic acid as the coupling partner and 18 h reaction time, cyclopentenone **8b** (546 mg, 2.70 mmol, 93% yield) was obtained as an orange solid with the characterization data matching those previously reported.[15] Purification was performed by flash column chromatography on silica gel (dry loading, 20 cm column height, gradient: pentane/EtOAc = 5:1 → 3:1).

^{1}H NMR (400 MHz, $CDCl_3$) δ = 7.28 – 7.22 (m, 2H), 6.98 – 6.93 (m, 2H), 3.83 (s, 3H), 2.68 – 2.62 (m, 2H), 2.57 – 2.52 (m, 2H), 2.19 (s, 3H) ppm; **R_f** (pentane/EtOAc, 3:1) = 0.27.

3-Methyl-2-(4-(trifluoromethyl)phenyl)cyclopent-2-en-1-one (**8c**)

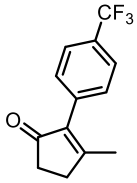

According to *General Procedure 3a*, starting from enone **7a** (3.00 mmol) with commercial (4-(trifluoromethyl)phenyl)boronic acid as the coupling partner and 18 h reaction time, cyclopentenone **8c** (524 mg, 2.18 mmol, 73% yield) was obtained as a yellow solid with the characterization data matching those previously reported.[16] Purification was performed

by flash column chromatography on silica gel (dry loading, 15 cm column height, gradient: pentane/EtOAc = 4:1 → 3:1).

^{1}H NMR (400 MHz, $CDCl_3$) δ = 7.70 – 7.63 (m, 2H), 7.44 – 7.38 (m, 2H), 2.74 – 2.65 (m, 2H), 2.64 – 2.55 (m, 2H), 2.23 – 2.18 (m, 3H) ppm; **R_f** (pentane/EtOAc, 3:1) = 0.22.

2-([1,1'-Biphenyl]-4-yl)-3-methylcyclopent-2-en-1-one (**8d**)

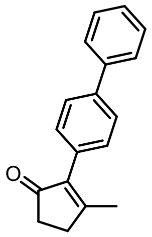

According to *General Procedure 3a*, starting from enone **7a** (2.89 mmol) with commercial [1,1'-biphenyl]-4-ylboronic acid as the coupling partner and 7 h reaction time, cyclopentenone **8d** (680 mg, 2.74 mmol, 95% yield) was obtained as a yellow solid. Purification was performed by flash column chromatography on silica gel (dry loading, 18 cm column height, gradient: pentane/EtOAc = 5:1 → 3:1).

^{1}H NMR (500 MHz, $CDCl_3$) δ = 7.66 – 7.60 (m, 4H), 7.47 – 7.43 (m, 2H), 7.40 – 7.33 (m, 3H), 2.71 – 2.67 (m, 2H), 2.60 – 2.56 (m, 2H), 2.24 (s, 3H) ppm; **^{13}C{^{1}H} NMR** (126 MHz, $CDCl_3$) δ = 207.8, 172.0, 141.0, 140.6, 140.1, 130.9, 129.6, 128.9, 127.5, 127.24, 127.19, 35.0, 32.0, 18.6 ppm; **IR** (ATR) ṽ = 2910 (w), 1698 (s), 1635 (w), 1600 (w), 1487 (m), 1436 (w), 1405 (w), 1379 (m), 1341 (m), 1297 (w), 1134 (m), 937 (w), 840 (w), 767 (m), 732 (m), 698 (m), 650 (w) cm^{-1}; **HRMS** (nanochip-ESI/LTQ-Orbitrap) *m/z* = calcd. for $[C_{18}H_{17}O]^+$, $[M+H]^+$: 249.1274, found: 249.1272; **R_f** (pentane/EtOAc, 3:1) = 0.26; **M.p.** = 165-166 °C.

3-Methyl-2-(4-(methylsulfonyl)phenyl)cyclopent-2-en-1-one (**8e**)

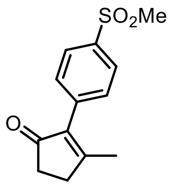

According to *General Procedure 3a*, starting from enone **7a** (2.88 mmol) with commercial (4-(methylsulfonyl)phenyl)boronic acid as the coupling partner and 24 h reaction time, cyclopentenone **8e** (519 mg, 2.07 mmol, 72% yield) was obtained as a light-brown solid. Purification was performed by flash column chromatography on silica gel (dry loading, 18 cm column height, gradient: pentane/EtOAc = 2:1 → 1:2).

^{1}H NMR (500 MHz, $CDCl_3$) δ = 8.00 – 7.96 (m, 2H), 7.51 – 7.47 (m, 2H), 3.06 (s, 3H), 2.75 – 2.71 (m, 2H), 2.61 – 2.58 (m, 2H), 2.21 (s, 3H) ppm; **^{13}C{^{1}H} NMR** (126 MHz, $CDCl_3$) δ = 206.9, 174.1, 139.6, 139.1, 137.8, 130.2, 127.6, 44.7, 35.0, 32.2, 18.6 ppm; **IR** (ATR) ṽ = 2924 (w), 1696 (s), 1637 (w), 1380 (w), 1341 (w), 1307 (s), 1151 (s), 1090 (w), 958 (w), 776 (m), 600 (w), 547 (w), 524 (w) cm^{-1}; **HRMS** (nanochip-ESI/LTQ-Orbitrap) *m/z* = calcd. for $[C_{13}H_{15}O_3S]^+$, $[M+H]^+$: 251.0736, found: 251.0732; **R_f** (pentane/EtOAc, 1:2) = 0.29; **M.p.** = 137-138 °C.

2-(3,5-Di-*tert*-butylphenyl)-3-methylcyclopent-2-en-1-one (**8f**)

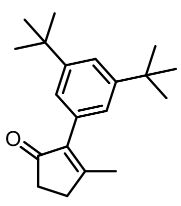

According to *General Procedure 3a*, starting from enone **7a** (3.86 mmol) with commercial (3,5-di-*tert*-butylphenyl)boronic acid as the coupling partner and 6 h reaction time, cyclopentenone **8f** (1016 mg, 3.57 mmol, 92% yield) was obtained as a white solid. Purification was performed by flash column chromatography on silica gel (dry loading, 15 cm column height, isocratic: pentane/EtOAc = 9:1).

^{1}H NMR (400 MHz, $CDCl_3$) δ = 7.37 (t, *J* = 1.9 Hz, 1H), 7.11 (d, *J* = 1.9 Hz, 2H), 2.69 – 2.61 (m, 2H), 2.58 – 2.51 (m, 2H), 2.21 – 2.17 (m, 3H), 1.33 (s, 18H) ppm; **^{13}C{^{1}H} NMR** (101 MHz, $CDCl_3$) δ = 207.9, 171.3, 150.4, 141.4, 131.0, 123.6, 121.8, 35.0, 31.9, 31.6, 18.6 ppm; **IR** (ATR) ṽ = 2961 (m), 2906 (w), 2866 (w), 1698 (s), 1637 (w), 1594 (w), 1475 (w), 1439 (w), 1379 (w), 1362 (w), 1343 (w), 1248 (w), 1201 (w), 1142 (w), 714 (w) cm^{-1}; **HRMS** (ESI/APCI) *m/z* = calcd. for $[C_{20}H_{29}O]^+$, $[M+H]^+$: 285.2213, found: 285.2212; **R_f** (pentane/EtOAc, 9:1) = 0.28; **M.p.** = 116-117 °C.

2-(3,5-Bis(trifluoromethyl)phenyl)-3-methylcyclopent-2-en-1-one (**8g**)

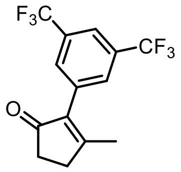

According to *General Procedure 3a*, starting from enone **7a** (2.89 mmol) with commercial (3,5-bis(trifluoromethyl)phenyl)boronic acid as the coupling partner and 12 h reaction time, cyclopentenone **8g** (724 mg, 2.35 mmol, 81% yield) was obtained as a light-yellow solid. Purification was performed by flash column chromatography on silica gel (dry loading, 18 cm column height, gradient: pentane/EtOAc = 7:1 → 5:1).

^{1}H NMR (400 MHz, $CDCl_3$) δ = 7.85 – 7.82 (m, 1H), 7.77 – 7.74 (m, 2H), 2.76 – 2.72 (m, 2H), 2.62 – 2.58 (m, 2H), 2.24 – 2.21 (m, 3H) ppm; **^{13}C{^{1}H} NMR** (101 MHz, $CDCl_3$) δ = 206.3, 174.1, 138.2, 134.1, 131.8 (q, *J* = 33.4 Hz), 129.6 – 129.3 (m), 123.4 (q, *J* = 272.8 Hz), 121.6 (hept, *J* = 3.9 Hz), 34.9, 32.2, 18.5 ppm; **^{19}F{^{1}H} NMR** (376 MHz, $CDCl_3$) δ = -62.84 ppm; **IR** (ATR) ṽ = 1701 (m), 1645 (w), 1469 (w), 1438 (w), 1392 (m), 1375 (w), 1325 (w), 1278 (s), 1174 (m), 1129 (s), 895 (w), 845 (w), 707 (w), 684 (w) cm^{-1}; **HRMS** (ESI/QTOF) *m/z* = calcd. for $[C_{14}H_{11}F_6O]^+$, $[M+H]^+$: 309.0709, found: 309.0711; **R_f** (pentane/EtOAc, 5:1) = 0.29; **M.p.** = 89-90 °C.

2-(3,5-Difluorophenyl)-3-methylcyclopent-2-en-1-one (**8h**)

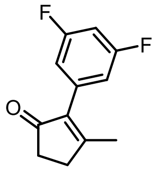

According to *General Procedure 3a*, starting from enone **7a** (2.92 mmol) with commercial (3,5-difluorophenyl)boronic acid as the coupling partner and 24 h reaction time, cyclopentenone **8h** (524 mg, 2.52 mmol, 86% yield) was obtained as a beige solid. Purification was performed by flash column chromatography on silica gel (dry loading, 18 cm column height, gradient: pentane/EtOAc = 6:1 → 3:1).

^{1}H NMR (400 MHz, $CDCl_3$) δ = 6.87 – 6.80 (m, 2H), 6.77 (tt, *J* = 9.0, 2.4 Hz, 1H), 2.71 – 2.65 (m, 2H), 2.60 – 2.53 (m, 2H), 2.20 (s, 3H) ppm; **^{13}C{^{1}H} NMR** (101 MHz, $CDCl_3$) δ = 206.6, 173.3, 163.0 (dd, *J* = 247.9, 13.1 Hz), 138.7 (t, *J* = 2.4 Hz), 135.0 (t, *J* = 10.1 Hz), 112.2 (dd, *J* = 18.7, 7.1 Hz), 103.3 (t, *J* = 25.3 Hz), 34.8, 32.1, 18.5 ppm; **^{19}F{^{1}H} NMR** (376 MHz, $CDCl_3$) δ = -110.03 ppm; **IR** (ATR) ṽ = 3080 (w), 2929 (w), 1688 (s), 1639 (w), 1617 (w), 1589 (m), 1443 (w), 1385 (w), 1351 (w), 1273 (m), 1126 (w), 1112 (m), 980 (w), 874 (w), 828 (w), 701 (w) cm^{-1}; **HRMS** (Sicrit plasma/LTQ-Orbitrap) *m/z* = calcd. for $[C_{12}H_{11}F_2O]^+$, $[M+H]^+$: 209.0772, found: 209.0770; **R_f** (pentane/EtOAc, 4:1) = 0.26; **M.p.** = 94-95 °C.

2-(2,6-Difluorophenyl)-3-methylcyclopent-2-en-1-one (**8i**)

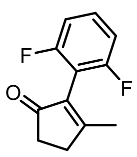

According to *General Procedure 3c*, starting from enone **7a** (3.43 mmol) with commercial (2,6-difluorophenyl)boronic acid as the coupling partner and 2 h reaction time, cyclopentenone **8i** (692 mg, 3.33 mmol, 97% yield) was obtained as a light-orange solid. Purification was performed by flash column chromatography on silica gel (dry loading, 18 cm column height, gradient: pentane/EtOAc = 5:1 → 4:1).

^{1}H NMR (400 MHz, CD_2Cl_2) δ = 7.39 – 7.30 (m, 1H), 7.01 – 6.93 (m, 2H), 2.76 – 2.72 (m, 2H), 2.56 – 2.52 (m, 2H), 2.06 (s, 3H) ppm; **^{13}C{^{1}H} NMR** (101 MHz, CD_2Cl_2) δ = 205.7, 176.9, 160.9 (dd, *J* = 248.6, 7.9 Hz), 131.1, 130.5 (t, *J* = 10.2 Hz), 111.7 (dd, *J* = 19.4, 6.3 Hz), 109.9 (t, *J* = 21.3 Hz), 35.2, 32.7, 18.6 (t, *J* = 1.8 Hz) ppm; **^{19}F{^{1}H} NMR** (376 MHz, CD_2Cl_2) δ = -111.19 ppm; **IR** (ATR) ṽ = 1706 (s), 1653 (w), 1618 (m), 1585 (w), 1464 (s), 1433 (w), 1381 (w), 1337 (w), 1297 (w), 1271 (w), 1235 (w), 1135 (w), 1001 (m), 786 (m), 560 (w) cm^{-1}; **HRMS** (nanochip-ESI/LTQ-Orbitrap) *m/z* = calcd. for $[C_{12}H_{10}F_2NaO]^+$, $[M+Na]^+$: 231.0592, found: 231.0587; **R$_f$** (pentane/EtOAc, 4:1) = 0.32; **M.p.** = 34-35 °C.

2-(2,6-Dimethoxyphenyl)-3-methylcyclopent-2-en-1-one (**8j**)

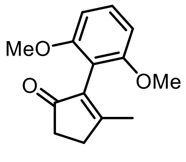

According to *General Procedure 3b*, starting from enone **7a** (3.17 mmol) with commercial (2,6-dimethoxyphenyl)boronic acid as the coupling partner and 12 h reaction time, cyclopentenone **8j** (552 mg, 2.38 mmol, 75% yield) was obtained as a pale orange solid. Purification was performed by flash column chromatography on silica gel (dry loading, 18 cm column height, gradient: pentane/EtOAc = 3:1 → 2:1).

^{1}H NMR (400 MHz, CD_2Cl_2) δ = 7.28 (t, *J* = 8.4 Hz, 1H), 6.61 (d, *J* = 8.4 Hz, 2H), 3.72 (s, 6H), 2.70 – 2.64 (m, 2H), 2.50 – 2.44 (m, 2H), 1.92 – 1.89 (m, 3H) ppm; **^{13}C{^{1}H} NMR** (101 MHz, CD_2Cl_2) δ = 207.3, 174.1, 158.6, 135.4, 129.9, 110.5, 104.3, 56.1, 35.3, 32.3, 18.4 ppm; **IR** (ATR) ṽ = 2938 (w), 2837 (w), 1698 (m), 1649 (w), 1590 (w), 1471 (m), 1433 (w), 1379 (w), 1335 (w), 1300 (w), 1285 (w), 1250 (m), 1133 (w), 1109 (s), 937 (w), 780 (w) cm^{-1}; **HRMS** (ESI/QTOF) *m/z* = calcd. for $[C_{14}H_{17}O_3]^+$, $[M+H]^+$: 233.1172, found: 233.1181; **R$_f$** (pentane/EtOAc, 2:1) = 0.21; **M.p.** = 111-112 °C.

2-(2,6-Diisopropoxyphenyl)-3-methylcyclopent-2-en-1-one (**8k**)

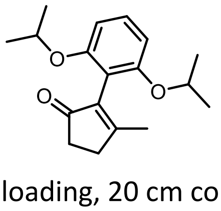

According to *General Procedure 3b*, starting from enone **7a** (3.30 mmol) with (2,6-diisopropoxyphenyl)boronic acid **SI2** as the coupling partner and 24 h reaction time, cyclopentenone **8k** (655 mg, 2.27 mmol, 69% yield) was obtained as a light-yellow oil. Purification was performed by flash column chromatography on silica gel (dry loading, 20 cm column height, gradient: pentane/EtOAc = 8:1 → 5:1).

^{1}H NMR (400 MHz, $CDCl_3$) δ = 7.17 (t, *J* = 8.3 Hz, 1H), 6.54 (d, *J* = 8.3 Hz, 2H), 4.40 (hept, *J* = 6.1 Hz, 2H), 2.68 – 2.63 (m, 2H), 2.54 – 2.49 (m, 2H), 1.95 – 1.93 (m, 3H), 1.23 (d, *J* = 6.1 Hz, 6H), 1.20 (d, *J* = 6.1 Hz, 6H) ppm; **^{13}C{^{1}H} NMR** (101 MHz, $CDCl_3$) δ = 207.5, 172.9, 157.1, 136.1, 129.2, 112.8, 106.8, 70.8, 35.2, 32.1, 22.4, 22.1, 18.9 ppm; **IR** (ATR) ṽ = 2976 (w), 2928 (w), 1703 (s), 1651 (w), 1588 (m), 1459 (s), 1381

(w), 1333 (w), 1295 (w), 1249 (m), 1115 (s), 1067 (m), 778 (w) cm^{-1}; **HRMS** (ESI/QTOF) *m/z* = calcd. for $[C_{18}H_{24}NaO_3]^+$, $[M+Na]^+$: 311.1618, found: 311.1615; **R_f** (pentane/EtOAc, 4:1) = 0.38.

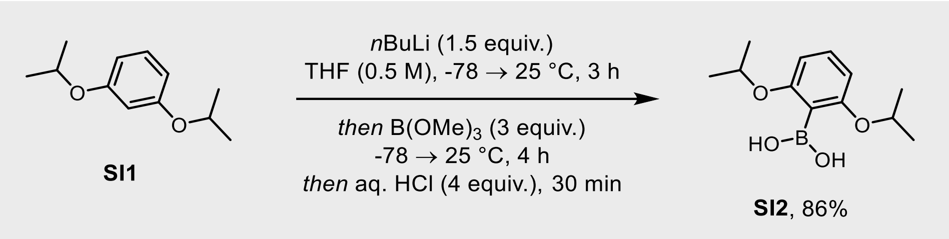

Scheme S9. Preparation of (2,6-diisopropoxyphenyl)boronic acid.

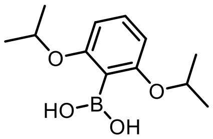

The required (2,6-diisopropoxyphenyl)boronic acid **SI2** was prepared according to the following procedure (**Scheme S9**) with the characterization data matching those previously reported.[17]

At -78 °C (acetone/dry ice bath) and under an atmosphere of nitrogen, *n*-butyllithium solution (1.6 M in hexanes, 23.5 mL, 1.5 equiv.) was added dropwise to a solution of 1,3-di(propan-2-yloxy)benzene **SI1** (4862 mg, 25.03 mmol, 1.0 equiv.) in anhydrous THF (2 mL/mmol). The cold bath was removed, and the reaction mixture was stirred at room temperature (25 °C) for 3 hours. Next, it was cooled back to -78 °C, and trimethyl borate (8.53 mL, 3.0 equiv.) was added dropwise. The reaction mixture was stirred at -78 °C for 1 hour, followed by removal of the cold bath and stirring at room temperature (25 °C) for 3 hours. After addition of aq. HCl solution (1 M, 4 equiv.), the mixture was stirred vigorously for another 30 min, after which it was diluted with Et_2O. The organic phase was sequentially washed with water, sat. aq. $NaHCO_3$ solution, and brine, then dried over $MgSO_4$, filtered, and concentrated under reduced pressure. The residue was purified by flash column chromatography on silica gel (wet loading with pentane/EtOAc = 15:1, 18 cm column height, gradient: pentane/EtOAc = 15:1 → 4:1), affording (2,6-diisopropoxy-phenyl)boronic acid **SI2** (5105 mg, 21.44 mmol, 86% yield) as a pale yellow liquid.

^{1}H NMR (400 MHz, $CDCl_3$) δ = 7.49 (s, 2H), 7.31 (t, *J* = 8.3 Hz, 1H), 6.58 (d, *J* = 8.4 Hz, 2H), 4.68 (hept, *J* = 6.1 Hz, 2H), 1.39 (d, *J* = 6.1 Hz, 12H) ppm; **R_f** (pentane/EtOAc, 4:1) = 0.37.

2-(Anthracen-9-yl)-3-methylcyclopent-2-en-1-one (**8l**)

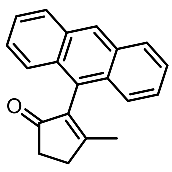

According to *General Procedure 3b*, starting from enone **7a** (2.32 mmol) with commercial anthracen-9-ylboronic acid as the coupling partner and 10 h reaction time, cyclopentenone **8l** (497 mg, 1.83 mmol, 79% yield) was obtained as a yellow solid. Purification was performed by flash column chromatography on silica gel (dry loading, 18 cm column height, gradient: pentane/EtOAc = 5:1 → 2:1).

^{1}H NMR (400 MHz, CD_2Cl_2) δ = 8.51 (s, 1H), 8.09 – 8.05 (m, 2H), 7.69 – 7.65 (m, 2H), 7.52 – 7.42 (m, 4H), 3.01 – 2.97 (m, 2H), 2.80 – 2.77 (m, 2H), 1.86 (s, 3H) ppm; **$^{13}C\{^1H\}$ NMR** (101 MHz, CD_2Cl_2) δ = 207.9, 176.5, 139.5, 131.8, 130.4, 129.2, 127.69, 127.66, 126.1, 126.0, 125.6, 35.7, 32.6, 18.5 ppm; **IR** (ATR) ṽ = 3050 (w), 1693 (s), 1642 (m), 1623 (w), 1435 (w), 1412 (w), 1381 (m), 1267 (w), 1179 (w), 1152 (w), 1070

(w), 889 (w), 845 (w), 786 (w), 738 (m), 504 (w) cm^{-1}; **HRMS** (Sicrit plasma/LTQ-Orbitrap) *m/z* = calcd. for $[C_{20}H_{17}O]^+$, $[M+H]^+$: 273.1274, found: 273.1273; **R$_f$** (pentane/EtOAc, 2:1) = 0.37; **M.p.** = 227-228 °C.

3-Methyl-2-(3,4,5-trimethoxyphenyl)cyclopent-2-en-1-one (**8m**)

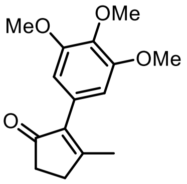

According to *General Procedure 3a*, starting from enone **7a** (3.00 mmol) with commercial (3,4,5-trimethoxyphenyl)boronic acid as the coupling partner and 3 h reaction time, cyclopentenone **8m** (741 mg, 2.82 mmol, 94% yield) was obtained as a brown solid. Purification was performed by flash column chromatography on silica gel (dry loading, 15 cm column height, gradient: pentane/EtOAc = 2:1 → 1:2).

^{1}H NMR (400 MHz, $CDCl_3$) δ = 6.49 (s, 2H), 3.86 (s, 9H), 2.70 – 2.62 (m, 2H), 2.59 – 2.51 (m, 2H), 2.22 – 2.17 (m, 3H) ppm; **^{13}C{^{1}H} NMR** (101 MHz, $CDCl_3$) δ = 207.7, 171.9, 153.3, 140.6, 137.8, 127.5, 106.5, 61.0, 56.3, 35.0, 31.9, 18.6 ppm; **IR** (ATR) ṽ = 2939 (w), 1693 (m), 1639 (w), 1580 (m), 1505 (w), 1453 (w), 1413 (w), 1381 (w), 1355 (w), 1279 (w), 1237 (w), 1170 (w), 1126 (s), 1007 (w), 826 (w) cm^{-1}; **HRMS** (ESI/QTOF) *m/z* = calcd. for $[C_{15}H_{18}NaO_4]^+$, $[M+Na]^+$: 285.1097, found: 285.1111; **R$_f$** (pentane/EtOAc, 1:1) = 0.20; **M.p.** = 123-124 °C.

2-(Benzo[*b*]thiophen-7-yl)-3-methylcyclopent-2-en-1-one (**8n**)

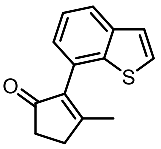

According to *General Procedure 3b*, starting from enone **7a** (2.96 mmol) with commercial benzo[b]thiophen-7-ylboronic acid as the coupling partner and 18 h reaction time, cyclopentenone **8n** (672 mg, 2.94 mmol, 99% yield) was obtained as a gray solid. Purification was performed by flash column chromatography on silica gel (dry loading, 15 cm column height, gradient: pentane/EtOAc = 5:1 → 2:1).

^{1}H NMR (400 MHz, $CDCl_3$) δ = 7.81 (d, *J* = 8.0 Hz, 1H), 7.45 – 7.34 (m, 3H), 7.14 (d, *J* = 7.1 Hz, 1H), 2.80 – 2.74 (m, 2H), 2.69 – 2.62 (m, 2H), 2.08 (s, 3H) ppm; **^{13}C{^{1}H} NMR** (101 MHz, $CDCl_3$) δ = 206.9, 174.2, 140.6, 140.1, 139.7, 127.1, 126.3, 125.5, 124.5, 124.3, 123.4, 35.1, 32.1, 19.0 ppm; **IR** (ATR) ṽ = 1697 (s), 1641 (w), 1459 (w), 1432 (w), 1377 (w), 1351 (w), 1325 (w), 1297 (w), 1144 (w), 1086 (w), 871 (w), 797 (w), 706 (w) cm^{-1}; **HRMS** (ESI/QTOF) *m/z* = calcd. for $[C_{14}H_{13}OS]^+$, $[M+H]^+$: 229.0682, found: 229.0680; **R$_f$** (pentane/EtOAc, 2:1) = 0.42; **M.p.** = 91-92 °C.

3-Methyl-2-(4-nitrophenyl)cyclopent-2-en-1-one (**8o**)

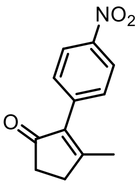

According to *General Procedure 3a*, starting from enone **7a** (3.04 mmol) with commercial (4-nitrophenyl)boronic acid as the coupling partner and 6 h reaction time, cyclopentenone **8o** (550 mg, 2.53 mmol, 83% yield) was obtained as a dark orange solid. Purification was performed by flash column chromatography on silica gel (dry loading, 18 cm column height, gradient: pentane/EtOAc = 3:1 → 1:1).

^{1}H NMR (400 MHz, $CDCl_3$) δ = 8.29 – 8.25 (m, 2H), 7.51 – 7.46 (m, 2H), 2.75 – 2.72 (m, 2H), 2.62 – 2.58 (m, 2H), 2.23 (s, 3H) ppm; **^{13}C{^{1}H} NMR** (101 MHz, $CDCl_3$) δ = 206.6, 174.1, 147.2, 138.82, 138.79, 130.2, 123.6, 35.0, 32.3, 18.6 ppm; **IR** (ATR) ṽ = 1697 (s), 1635 (w), 1597 (m), 1515 (s), 1493 (w), 1436 (w), 1406 (w), 1379 (w), 1347 (s), 1299 (w), 1133 (m), 1108 (w), 939 (w), 852 (m), 756 (w), 701 (m) cm^{-1}; **HRMS**

(ESI/QTOF) *m/z* = calcd. for $[C_{12}H_{12}NO_3]^+$, $[M+H]^+$: 218.0812, found: 218.0817; **R$_f$** (pentane/EtOAc, 1:1) = 0.35; **M.p.** = 101-102 °C.

3-Methyl-2-(quinolin-8-yl)cyclopent-2-en-1-one (**8p**)

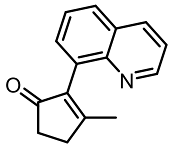

According to *General Procedure 3b*, starting from enone **7a** (3.35 mmol) with commercial quinolin-8-ylboronic acid as the coupling partner and 8 h reaction time, cyclopentenone **8p** (720 mg, 3.22 mmol, 96% yield) was obtained as an orange solid. Purification was performed by flash column chromatography on silica gel (dry loading, 18 cm column height, gradient: pentane/EtOAc = 1:1 → 1:2 → 0:1, *then* EtOAc/MeOH = 20:1 → 9:1).

^{1}H NMR (400 MHz, $CDCl_3$) δ = 8.89 (dd, *J* = 4.2, 1.8 Hz, 1H), 8.17 (dd, *J* = 8.3, 1.8 Hz, 1H), 7.83 (dd, *J* = 8.0, 1.6 Hz, 1H), 7.62 – 7.56 (m, 1H), 7.55 – 7.51 (m, 1H), 7.39 (dd, *J* = 8.3, 4.2 Hz, 1H), 2.99 – 2.55 (m, 4H), 2.01 (s, 3H) ppm; **^{13}C{^{1}H} NMR** (101 MHz, $CDCl_3$) δ = 207.9, 174.4, 150.0, 146.4, 139.8, 136.6, 131.9, 131.2, 128.8, 128.3, 126.3, 121.2, 35.4, 32.3, 19.1 ppm; **IR** (ATR) ṽ = 2921 (w), 1695 (s), 1647 (w), 1594 (w), 1496 (w), 1470 (w), 1434 (w), 1388 (w), 1333 (w), 1294 (w), 1147 (w), 933 (w), 837 (w), 795 (m), 587 (w) cm^{-1}; **HRMS** (ESI/QTOF) *m/z* = calcd. for $[C_{15}H_{14}NO]^+$, $[M+H]^+$: 224.1070, found: 224.1076; **R$_f$** (EtOAc/MeOH, 9:1) = 0.42; **M.p.** = 118-119 °C.

***General Procedure 4** – Sonogashira Cross-coupling with a Iodo-substituted Enone.*[18]

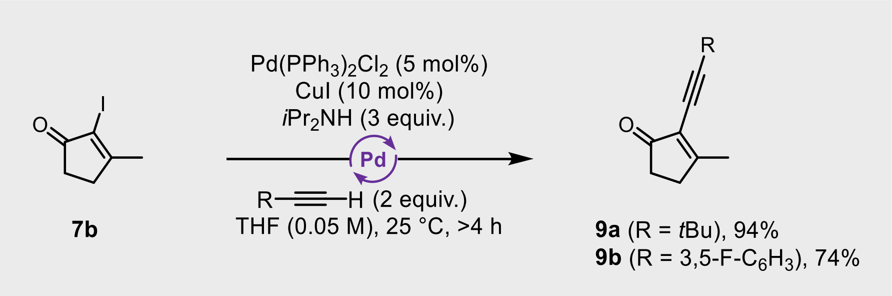

Scheme S10. Sonogashira cross-coupling with a iodo-substituted enone.

A round-bottom flask was charged with 2-iodo-3-methylcyclopent-2-en-1-one **7b** (1.0 equiv.), bis(triphenylphosphine)palladium(II) dichloride (5 mol%), and copper(I) iodide (10 mol%). After sealing the flask and placing it under an atmosphere of nitrogen by Schlenk technique, anhydrous THF (20 mL/mmol) was added, and the resulting solution was degassed by bubbling nitrogen for 10 minutes under stirring. Next, the desired terminal alkyne (2.0 equiv.) was added, and the mixture was cooled to 0 °C under stirring. Then, diisopropylamine (3.0 equiv.) was added dropwise. The ice bath was removed, and the reaction mixture was stirred at room temperature (25 °C) for 4-18 hours. Afterwards, the mixture was diluted with EtOAc, filtered through celite (2 cm), and washed with consecutively sat. aq. NH_4Cl solution, and aq. HCl solution (1 M). The combined aqueous phases were back-extracted with EtOAc. Next, the combined organic layers were washed with brine, dried over $MgSO_4$, filtered, and concentrated under reduced pressure. The residue was purified by flash column chromatography on silica gel, affording the corresponding alkynyl-substituted cyclopentenone **9**.

2-(3,3-Dimethylbut-1-yn-1-yl)-3-methylcyclopent-2-en-1-one (**9a**)

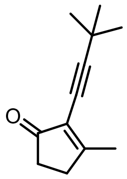

According to *General Procedure 4*, starting from enone **7b** (2.31 mmol) with commercial 3,3-dimethylbut-1-yne as the coupling partner and 4 h reaction time, cyclopentenone **9a** (381 mg, 2.16 mmol, 94% yield) was obtained as an off-white solid. Purification was performed by flash column chromatography on silica gel (dry loading, 18 cm column height, gradient: pentane/EtOAc = 8:1 → 5:1).

^{1}H NMR (500 MHz, $CDCl_3$) δ = 2.61 – 2.57 (m, 2H), 2.45 – 2.41 (m, 2H), 2.19 (s, 3H), 1.30 (s, 9H) ppm; **^{13}C{^{1}H} NMR** (126 MHz, $CDCl_3$) δ = 206.0, 178.5, 126.6, 107.5, 69.4, 34.8, 31.8, 31.2, 28.4, 18.9 ppm; **IR** (ATR) ṽ = 2967 (m), 2928 (w), 2911 (w), 1742 (s), 1620 (w), 1455 (w), 1437 (w), 1378 (m), 1364 (w), 1337 (w), 1254 (m), 1183 (w), 921 (w), 725 (w) cm^{-1}; **HRMS** (nanochip-ESI/LTQ-Orbitrap) *m/z* = calcd. for $[C_{12}H_{16}NaO]^+$, $[M+Na]^+$: 199.1093, found: 199.1092; **R_f** (pentane/EtOAc, 5:1) = 0.30; **M.p.** = 91-92 °C.

2-((3,5-Difluorophenyl)ethynyl)-3-methylcyclopent-2-en-1-one (**9b**)

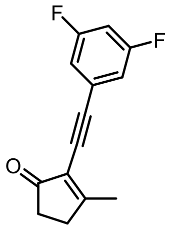

According to *General Procedure 4*, starting from enone **7b** (1.16 mmol) with commercial 1-ethynyl-3,5-difluorobenzene as the coupling partner and 18 h reaction time, cyclopentenone **9b** (200 mg, 0.86 mmol, 74% yield) was obtained as a brown solid. Purification was performed by flash column chromatography on silica gel (dry loading, 18 cm column height, gradient: pentane/EtOAc = 9:1 → 7:3).

^{1}H NMR (400 MHz, $CDCl_3$) δ = 7.06 – 6.99 (m, 2H), 6.79 (tt, *J* = 9.0, 2.4 Hz, 1H), 2.73 – 2.68 (m, 2H), 2.56 – 2.51 (m, 2H), 2.31 (s, 3H) ppm; **^{13}C{^{1}H} NMR** (101 MHz, $CDCl_3$) δ = 205.1, 181.0, 162.8 (dd, *J* = 248.9, 13.3 Hz), 125.65, 125.61 (t, *J* = 11.8 Hz), 114.9 (dd, *J* = 19.5, 7.6 Hz), 104.9 (t, *J* = 25.4 Hz), 95.3 (t, *J* = 4.0 Hz), 81.8, 35.0, 32.3, 19.3 ppm; **^{19}F{^{1}H} NMR** (376 MHz, $CDCl_3$) δ = -109.67 ppm; **IR** (ATR) ṽ = 2904 (w), 2215 (w), 1702 (s), 1621 (w), 1610 (m), 1587 (s), 1424 (m), 1385 (m), 1319 (w), 1201 (w), 1140 (w), 1120 (s), 990 (m), 865 (w), 836 (w), 672 (w) cm^{-1}; **HRMS** (ESI/QTOF) *m/z* = calcd. for $[C_{14}H_{11}F_2O]^+$, $[M+H]^+$: 233.0772, found: 233.0775.; **R_f** (pentane/EtOAc, 7:3) = 0.29; **M.p.** = 136-137 °C.

***General Procedure 5* – *Synthesis of Chloro- or Chalcogen-substituted Enones.*[19,20]**

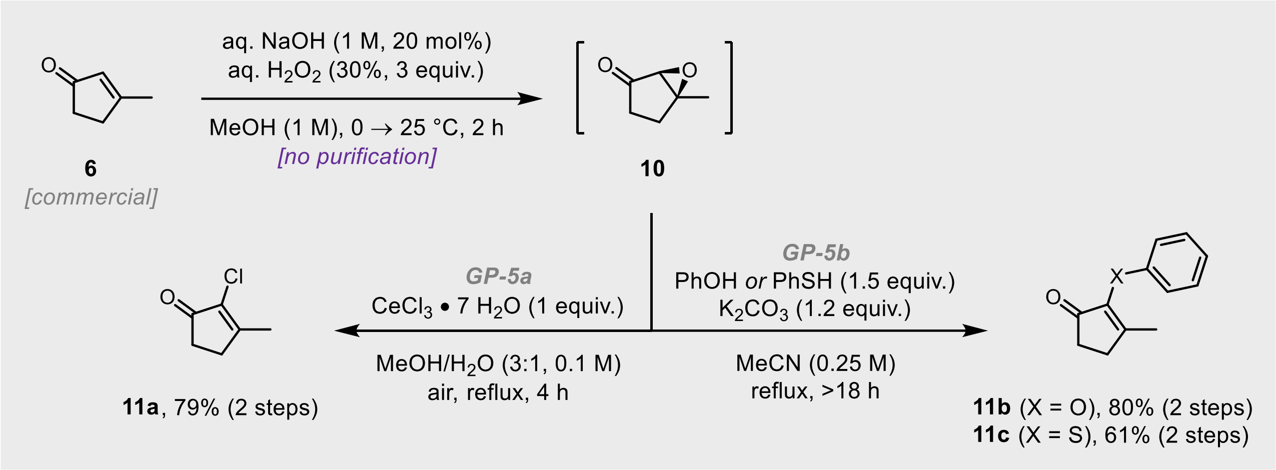

Scheme S11. Synthesis of chloro- or chalcogen-substituted enones.

General Procedure 5a: **Part 1.** At 0 °C (ice bath) and under an atmosphere of nitrogen, an aqueous hydrogen peroxide solution (30%, 3 equiv.) was added to a solution of commercial 3-methylcyclopent-2-en-1-one **6** (1.0 equiv.) in methanol (1 mL/mmol). Then, an aqueous sodium hydroxide solution (1 M, 20 mol%) was added dropwise, and the reaction was stirred at 0 °C for 1 hour. The ice bath was removed, and the reaction was stirred at room temperature (25 °C) for 1 hour, after which it was poored into a mixture of ice and brine, and was extracted with DCM (3x). The combined organic layers were washed with brine, dried over $MgSO_4$, filtered through silica (2 cm), and concentrated under reduced pressure, affording epoxide **10** as a transparent liquid, and in sufficient purity to be used in the next step without further purification.
Part 2. Crude epoxide **10** was dissolved in methanol (7.5 mL/mmol) and water (2.5 mL/mmol). Next, cerium trichloride heptahydrate (1.0 equiv.) was added and the resulting light-yellow solution was stirred at reflux in an oil bath (90 °C) under air for 4 hours. After cooling to room temperature (25 °C), the obtained white suspension was filtered over celite (2 cm) and the filtrate was extracted with DCM (3x). The combined organic layers were washed with brine, dried over $MgSO_4$, filtered, and concentrated under reduced pressure. The residue was purified by flash column chromatography on silica gel, affording 2-chloro-substituted cyclopentenone **11a**.

General Procedure 5b: **Part 1.** Intermediate epoxide **10** was synthesized from commercial 3-methylcyclopent-2-en-1-one **6** (1.0 equiv.) according to *General Procedure 5a*. **Part 2.** In a two-necked round-bottom flask with reflux condenser attached was made a solution of crude epoxide **10** in anhydrous acetonitrile (4 mL/mmol) under an atmosphere of nitrogen. Next, potassium carbonate (1.2 equiv.) and (thio)phenol (1.5 equiv.) were added sequentially and under a counterflow of nitrogen. The reaction was stirred at reflux in an oil bath (100 °C) for 18-24 hours. After cooling to room temperature (25 °C), the reaction mixture was poored into aqueous sodium hydroxide (1 M, 10 mL/mmol) and extracted with Et_2O (3x). The combined organic layers were washed with brine, dried over $MgSO_4$, filtered, and concentrated under reduced pressure. The residue was purified by flash column chromatography on silica gel, affording 2-chalcogen-substituted cyclopentenones **11b-c**.

2-Chloro-3-methylcyclopent-2-en-1-one (**11a**)

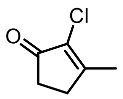

According to *General Procedure 5a*, starting from enone **6** (19.84 mmol), cyclopentenone **11a** (2054 mg, 15.73 mmol, 79% yield over 2 steps) was obtained as a white solid with the characterization data matching those previously reported.[21] Purification was performed by by flash column chromatography on silica gel (wet loading with DCM, 15 cm column height, isocratic: DCM).

^{1}H NMR (400 MHz, $CDCl_3$) δ = 2.66 – 2.61 (m, 2H), 2.55 – 2.50 (m, 2H), 2.18 – 2.16 (m, 3H) ppm; **R_f** (DCM) = 0.24.

3-Methyl-2-phenoxycyclopent-2-en-1-one (**11b**)

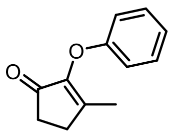

According to *General Procedure 5b*, starting from enone **6** (17.10 mmol) with phenol as the nucleophile and 18 h reaction time, cyclopentenone **11b** (2573 mg, 13.67 mmol, 80% yield over 2 steps) was obtained as a white crystalline solid with the characterization data matching those previously reported.[19] Purification was performed by flash column chromatography on silica gel (dry loading, 15 cm column height, gradient: pentane/EtOAc = 5:1 → 3:1).

^{1}H NMR (400 MHz, $CDCl_3$) δ = 7.29 – 7.23 (m, 2H), 7.03 – 6.98 (m, 1H), 6.90 – 6.85 (m, 2H), 2.63 – 2.58 (m, 2H), 2.52 – 2.47 (m, 2H), 2.01 (s, 3H) ppm; **R_f** (pentane/EtOAc, 3:1) = 0.34.

3-Methyl-2-(phenylthio)cyclopent-2-en-1-one (**11c**)

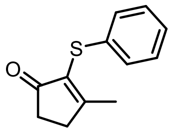

According to *General Procedure 5b*, starting from enone **6** (15.91 mmol) with thiophenol as the nucleophile and 24 h reaction time, cyclopentenone **11c** (1976 mg, 9.67 mmol, 61% yield over 2 steps) was obtained as a beige solid with the characterization data matching those previously reported (although incomplete,[22] and therefore a full set of data is provided below). Purification was performed by flash column chromatography on silica gel (dry loading, 18 cm column height, gradient: pentane/EtOAc = 4:1 → 2:1).

^{1}H NMR (400 MHz, $CDCl_3$) δ = 7.25 – 7.18 (m, 4H), 7.16 – 7.11 (m, 1H), 2.76 – 2.72 (m, 2H), 2.56 – 2.52 (m, 2H), 2.26 (s, 3H) ppm; **$^{13}C\{^1H\}$ NMR** (101 MHz, $CDCl_3$) δ = 205.2, 181.9, 134.7, 133.3, 129.0, 128.6, 126.2, 34.3, 32.6, 19.1 ppm; **IR** (ATR) ṽ = 1708 (s), 1600 (m), 1582 (w), 1478 (w), 1439 (w), 1405 (w), 1374 (w), 1265 (w), 1156 (w), 1085 (w), 1024 (w), 958 (w), 741 (m), 690 (w) cm^{-1}; **HRMS** (Sicrit plasma/LTQ-Orbitrap) *m/z* = calcd. for $[C_{12}H_{13}OS]^+$, $[M+H]^+$: 205.0682, found: 205.0680; **R_f** (pentane/EtOAc, 2:1) = 0.36; **M.p.** = 53-54 °C.

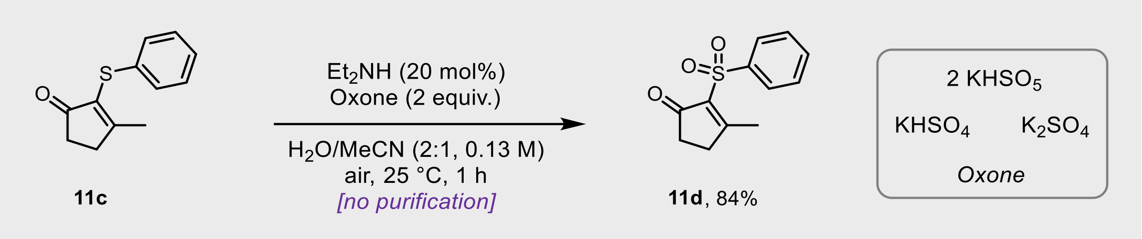

Scheme S12. Oxone-mediated oxidation towards a sulfone-substituted cyclopentenone.

3-Methyl-2-(phenylsulfonyl)cyclopent-2-en-1-one (**11d**)

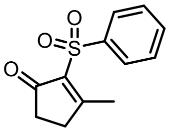

According to the following adaptatation of a reported procedure (**Scheme S12**),[23] cyclopentenone **11d** was obtained.

In a round-bottom flask was made a solution of sulfide-substituted enone **11c** (1127 mg, 5.52 mmol, 1.0 equiv.) in acetonitrile (2.5 mL/mmol), after which diethylamine (114 µL, 20 mol%) was added. Next, a solution of oxone (6783 mg, 2 equiv.) in water (5 mL/mmol) was added to the yellow solution, which immediately induced the precipitation of white solids, and the reaction mixture was stirred under air at room temperature (25 °C) for 1 hour. Afterwards, the suspension was filtered on a glass sinter funnel and the white filter cake was washed with water, then recovered by dissolving it in DCM. The organic phase was sequentially washed with sat. aq. $NaHCO_3$ solution and brine, then dried over $MgSO_4$, filtered, and concentrated under reduced pressure, affording cyclopentenone **11d** (1101 mg, 4.66 mmol, 84% yield) as a white solid, and in sufficient purity to be used in the next step without further purification.

^{1}H NMR (400 MHz, $CDCl_3$) δ = 8.08 – 8.04 (m, 2H), 7.64 – 7.59 (m, 1H), 7.56 – 7.50 (m, 2H), 2.75 – 2.70 (m, 2H), 2.64 (s, 3H), 2.44 – 2.40 (m, 2H) ppm; **^{13}C{^{1}H} NMR** (101 MHz, $CDCl_3$) δ = 199.9, 185.5, 140.5, 139.4, 133.9, 129.0, 128.4, 34.5, 33.9, 19.1 ppm; **IR** (ATR) ṽ = 1709 (s), 1602 (m), 1447 (w), 1428 (w), 1319 (m), 1308 (m), 1267 (w), 1150 (s), 1088 (w), 760 (m), 727 (w), 688 (w), 630 (m), 613 (w), 570 (m), 545 (w) cm^{-1}; **HRMS** (APCI/QTOF) *m/z* = calcd. for $[C_{12}H_{13}O_3S]^+$, $[M+H]^+$: 237.0580, found: 237.0576; **R_f** (pentane/acetone, 2:1) = 0.28; **M.p.** = 154-155 °C.

3. Synthesis of 1,2,3-Trisubstituted Cps

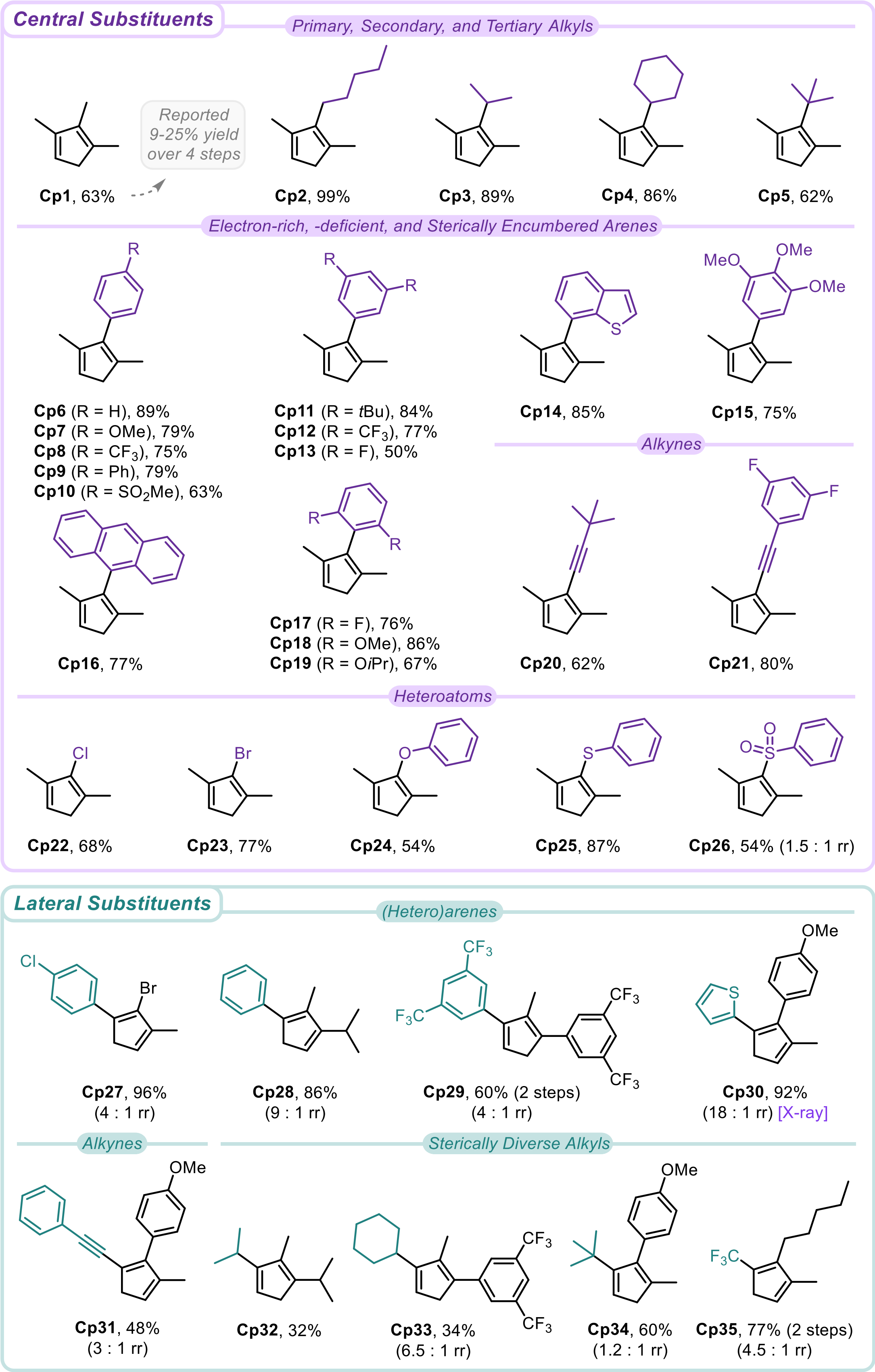

Figure S1. Overview of the prepared 1,2,3-trisubstituted cyclopentadienes.

General Procedure 6 – One-pot Addition-Elimination of Cyclopentenones towards 1,2,3-Cps.

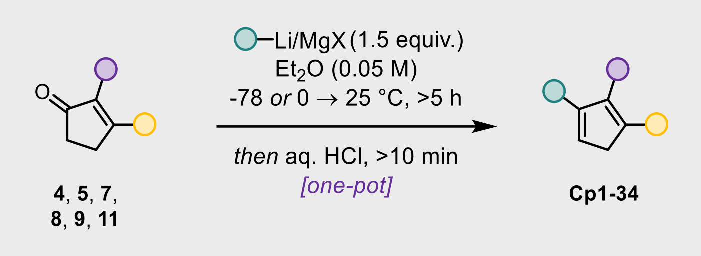

Scheme S13. One-pot addition-elimination of cyclopentenones towards 1,2,3-Cps.

At -78 °C (acetone/dry ice bath) and under an atmosphere of nitrogen, the desired organolithium reagent solution (1.5 equiv.) was added dropwise to a solution of the desired cyclopentenone (1.0 equiv.) in anhydrous Et_2O (20 mL/mmol). The reaction mixture was allowed to slowly warm up in the cold bath to room temperature (25 °C) under stirring for >5 hours (but can be left overnight). After dropwise quenching with sat. aq. NH_4Cl solution, aq. HCl solution (1 M, 3 mL/mmol) was added to affect elimination on the cyclopentenol intermediate, and the biphasic mixture was stirred vigorously (to ensure effective mixing of both phases) for >10 min, after which it was extracted with Et_2O (2x). The combined organic layers were sequentially washed with sat. aq. $NaHCO_3$ solution and brine, dried over $MgSO_4$, filtered, and concentrated under reduced pressure (min. 500 mbar at 40 °C). The residue was purified by flash column chromatography on silica gel. The solvent was carefully removed under reduced pressure (min. 200 mbar at 30 °C), affording the (often quite volatile) 1,2,3-trisubstituted cyclopentadienes (**Figure S1**). Long-term storage was done in a freezer at -40 °C.

Note 1: in the case of Grignard reagents, the addition was performed at 0 °C (ice bath), after which the reaction mixture was stirred at room temperature (25 °C) for >10 min. The elimination step was usually slower (compared to when organilithium reagents are used), requiring >3 hours of vigorous stirring. Generally, the elimination step was slower for cyclopentenols with electron-deficient substituents.

Note 2: most 1,2,3-Cps can be easily visualized during TLC analysis with a vanillin stain and subsequent heating, generating a strong blue-purple spot.

Note 3: occasionally lower yields observed are due to partial enolization of the substrate competing with the nucleophilic addition. This was observed for some bulky reagents and for some electron-deficient enones. The deprotonated substrate can however be easily recovered afterwards, and switching metals aided in favouring nucleophilic over basic behaviour.

Note 4: in the case of **Cp29** and **Cp35**, aq. HCl did not affect the elimination, but exposure of the cyclopentenol intermediates to PTSA in toluene at 80 °C did result in dehydration towards the 1,2,3-Cps.

Note 5: the 1,2,3-Cps were usually obtained as a single double bond isomer, but sometimes mixtures were observed (**Figure S2**). In such case, the major isomer was usually the thermodynamically favoured one (i.e. with most substituted olefinic bonds and most extended π-system), and over time the ratio shifted in its favour (even when stored in the freezer). The initial regioisomeric ratio (rr) measured after column chromatography is given. 2D NOESY experiments confirmed the assignments of the isomers.

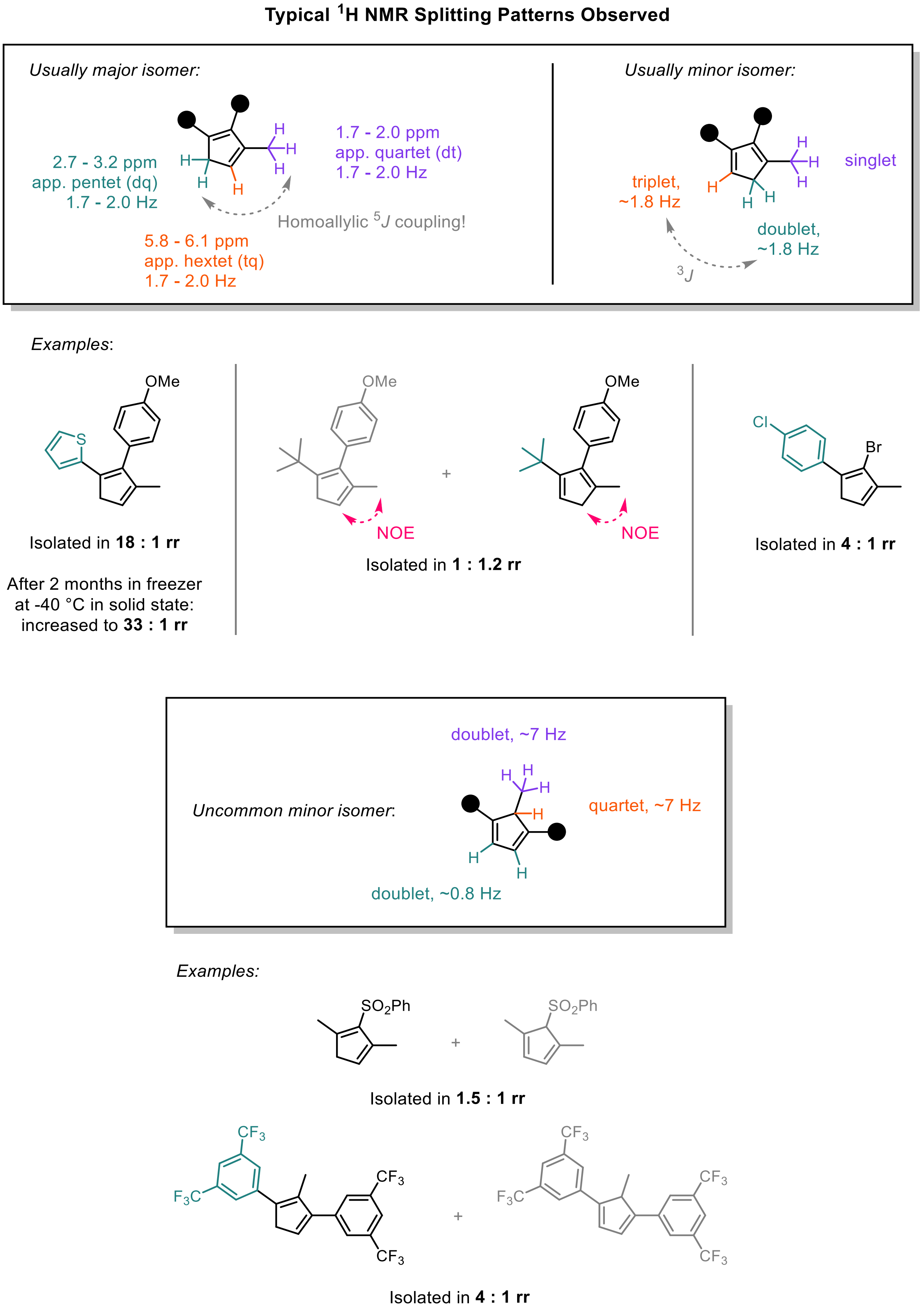

Figure S2. Overview of the observed 1,2,3-Cp double bond isomers, and their characteristic ^{1}H NMR signals (in $CDCl_3$).

1,2,3-Trimethylcyclopenta-1,3-diene (**Cp1**)

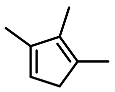

According to *General Procedure 6*, starting from enone **4d** (26.10 mmol) with MeLi·LiBr (1.5 M in Et_2O, 1.5 equiv.), cyclopentadiene **Cp1** (1779 mg, 16.44 mmol, 63% yield) was obtained as a light-yellow liquid with the characterization data matching those previously reported.[24] Notably, it allows a more efficient, expedient, reproducible, and safer synthesis of **4d** compared to the literature protocols (4 steps, 9-25% overall yield) that involve a poorly reproducible oxidative homocoupling of 2-butanone using toxic PbO_2.[24,25]

After work-up, extra care should be taken during evaporation (200 mbar at 20 °C) due to the volatility of this cyclopentadiene. Purification was performed by either flash column chromatography on silica gel (wet loading with pentane, 10 cm column height, isocratic: pentane) or simple distillation (under nitrogen, short path, oil bath at 200 °C, atmospheric pressure, receiving flask in cold bath at -78 °C).

^{1}H NMR (400 MHz, $CDCl_3$) δ = 5.85 – 5.80 (m, 1H), 2.78 – 2.74 (m, 2H), 1.94 – 1.93 (m, 3H), 1.92 – 1.90 (m, 3H), 1.82 – 1.80 (m, 3H) ppm; **R_f** (pentane) = 0.60.

1,3-Dimethyl-2-pentylcyclopenta-1,3-diene (**Cp2**)

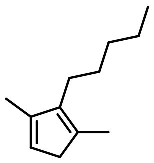

According to *General Procedure 6*, starting from commercial dihydrojasmone **4e** (17.66 mmol) with MeLi·LiBr (1.5 M in Et_2O, 1.5 equiv.), cyclopentadiene **Cp2** (2877 mg, 17.51 mmol, 99% yield) was obtained as a light-yellow liquid. Purification was performed by flash column chromatography on silica gel (wet loading with pentane, 15 cm column height, isocratic: pentane).

^{1}H NMR (400 MHz, $CDCl_3$) δ = 5.85 – 5.79 (m, 1H), 2.80 – 2.74 (m, 2H), 2.23 (t, *J* = 7.5 Hz, 2H), 1.96 – 1.90 (m, 6H), 1.46 – 1.22 (m, 6H), 0.90 (t, *J* = 6.8 Hz, 3H) ppm; **^{13}C{^{1}H} NMR** (101 MHz, $CDCl_3$) δ = 144.0, 140.6, 137.1, 123.7, 43.9, 32.1, 29.6, 25.8, 22.8, 14.4, 14.2, 13.9 ppm; **IR** (ATR) ṽ = 2955 (m), 2927 (s), 2857 (m), 1440 (w), 1378 (w), 1331 (w), 1091 (w), 1002 (w), 942 (w), 843 (w), 725 (w) cm^{-1}; **HRMS** (ESI/APCI) *m/z* = calcd. for $[C_{12}H_{21}]^+$, $[M+H]^+$: 165.1638, found: 165.1637; **R_f** (pentane) = 0.74.

2-Isopropyl-1,3-dimethylcyclopenta-1,3-diene (**Cp3**)

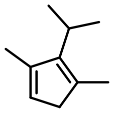

According to *General Procedure 6*, starting from enone **4a** (32.57 mmol) with MeLi·LiBr (1.5 M in Et_2O, 1.5 equiv.), cyclopentadiene **Cp3** (3931 mg, 28.86 mmol, 89% yield) was obtained as a light-yellow liquid with the characterization data matching those previously reported.[26] Purification was performed by flash column chromatography on silica gel (wet loading with pentane, 10 cm column height, isocratic: pentane).

^{1}H NMR (400 MHz, $CDCl_3$) δ = 5.84 – 5.78 (m, 1H), 2.86 (hept, *J* = 7.2 Hz, 1H), 2.76 – 2.71 (m, 2H), 2.02 – 1.99 (m, 3H), 1.98 (s, 3H), 1.19 (d, *J* = 7.2 Hz, 6H) ppm; **R_f** (pentane) = 0.69.

(2,5-Dimethylcyclopenta-1,4-dien-1-yl)cyclohexane (**Cp4**)

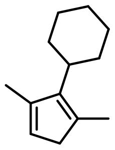

According to *General Procedure 6*, starting from enone **4b** (5.16 mmol) with MeLi·LiBr (1.5 M in Et_2O, 1.5 equiv.), cyclopentadiene **Cp4** (783 mg, 4.44 mmol, 86% yield) was obtained as a light-yellow liquid. Purification was performed by flash column chromatography on silica gel (wet loading with pentane, 10 cm column height, isocratic: pentane).

^{1}H NMR (400 MHz, $CDCl_3$) δ = 5.83 – 5.77 (m, 1H), 2.78 – 2.71 (m, 2H), 2.48 – 2.38 (m, 1H), 2.03 – 1.97 (m, 6H), 1.86 – 1.53 (m, 7H), 1.38 – 1.16 (m, 3H) ppm; **^{13}C{^{1}H} NMR** (101 MHz, $CDCl_3$) δ = 144.4, 144.0, 136.6, 124.8, 44.5, 38.1, 31.8, 27.4, 26.5, 16.2, 14.7 ppm; **IR** (ATR) ṽ = 2927 (s), 2852 (m), 1708 (w), 1673 (w), 1446 (m), 1370 (w), 1332 (w), 1095 (w), 1007 (w), 989 (w), 889 (w), 855 (w) cm^{-1}; **HRMS** (ESI/APCI) *m/z* = calcd. for $[C_{13}H_{21}]^+$, $[M+H]^+$: 177.1638, found: 177.1639; **R_f** (pentane) = 0.64.

2-(*tert*-Butyl)-1,3-dimethylcyclopenta-1,3-diene (**Cp5**)

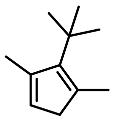

According to *General Procedure 6*, starting from enone **4c** (5.79 mmol) with MeLi·LiBr (1.5 M in Et_2O, 2.0 equiv.), cyclopentadiene **Cp5** (543 mg, 3.62 mmol, 62% yield) was obtained as a light-yellow liquid with the characterization data matching those previously reported.[8] Purification was performed by flash column chromatography on silica gel (wet loading with pentane, 10 cm column height, isocratic: pentane).

^{1}H NMR (400 MHz, $CDCl_3$) δ = 5.86 – 5.83 (m, 1H), 2.77 – 2.73 (m, 2H), 2.14 – 2.12 (m, 3H), 2.12 (s, 3H), 1.31 (s, 9H) ppm; **R_f** (pentane) = 0.65.

(2,5-Dimethylcyclopenta-1,4-dien-1-yl)benzene (**Cp6**)

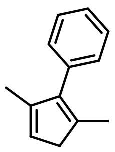

According to *General Procedure 6*, starting from enone **8a** (2.65 mmol) with MeLi·LiBr (1.5 M in Et_2O, 1.5 equiv.), cyclopentadiene **Cp6** (400 mg, 2.35 mmol, 89% yield) was obtained as a colourless liquid with the characterization data matching those previously reported.[27] Purification was performed by flash column chromatography on silica gel (wet loading with pentane, 10 cm column height, isocratic: pentane).

^{1}H NMR (400 MHz, $CDCl_3$) δ = 7.42 – 7.35 (m, 2H), 7.31 – 7.26 (m, 1H), 7.23 – 7.19 (m, 2H), 5.98 – 5.92 (m, 1H), 2.99 – 2.94 (m, 2H), 1.99 (s, 3H), 1.90 – 1.86 (m, 3H) ppm; **R_f** (pentane) = 0.53.

1-(2,5-Dimethylcyclopenta-1,4-dien-1-yl)-4-methoxybenzene (**Cp7**)

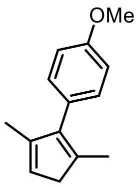

According to *General Procedure 6*, starting from enone **8b** (2.70 mmol) with MeLi·LiBr (1.5 M in Et_2O, 1.5 equiv.), cyclopentadiene **Cp7** (425 mg, 2.12 mmol, 79% yield) was obtained as a light-yellow liquid. Purification was performed by flash column chromatography on silica gel (wet loading with pentane, 10 cm column height, isocratic: pentane/DCM = 4:1).

^{1}H NMR (400 MHz, $CDCl_3$) δ = 7.17 – 7.11 (m, 2H), 6.96 – 6.91 (m, 2H), 5.97 – 5.91 (m, 1H), 3.84 (s, 3H), 2.97 – 2.91 (m, 2H), 1.98 (s, 3H), 1.90 – 1.86 (m, 3H) ppm; **^{13}C{^{1}H} NMR** (101 MHz, $CDCl_3$) δ = 158.3, 143.6, 141.8, 139.6, 130.5, 129.3, 124.2, 113.6, 55.3, 44.3, 15.3, 14.8 ppm; **IR** (ATR) ṽ = 2908 (w), 2833

(w), 1607 (w), 1508 (s), 1463 (w), 1440 (w), 1377 (w), 1284 (w), 1241 (s), 1172 (m), 1106 (w), 1036 (m), 1003 (w), 976 (w), 951 (w), 859 (w), 830 (m), 794 (w), 737 (w), 618 (w), 558 (w), 544 (w) cm^{-1}; **HRMS** (ESI/APCI) *m/z* = calcd. for $[C_{14}H_{17}O]^+$, $[M+H]^+$: 201.1274, found: 201.1270; $\mathbf{R_f}$ (pentane/DCM, 9:1) = 0.44.

1-(2,5-Dimethylcyclopenta-1,4-dien-1-yl)-4-(trifluoromethyl)benzene (**Cp8**)

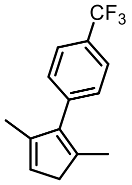

According to *General Procedure 6*, starting from enone **8c** (2.17 mmol) with MeLi·LiBr (1.5 M in Et_2O, 1.5 equiv.), cyclopentadiene **Cp8** (388 mg, 1.63 mmol, 75% yield) was obtained as a light-yellow liquid. Purification was performed by flash column chromatography on silica gel (wet loading with pentane, 10 cm column height, isocratic: pentane).

^{1}H NMR (400 MHz, $CDCl_3$) δ = 7.66 – 7.62 (m, 2H), 7.34 – 7.30 (m, 2H), 5.99 – 5.95 (m, 1H), 3.00 – 2.97 (m, 2H), 1.98 (s, 3H), 1.88 – 1.85 (m, 3H) ppm; **^{13}C{^{1}H} NMR** (101 MHz, $CDCl_3$) δ = 142.7, 141.5, 141.2, 140.8 (q, *J* = 1.3 Hz), 129.7, 128.7 (q, *J* = 32.0 Hz), 125.1 (q, *J* = 3.8 Hz), 125.0, 124.5 (q, *J* = 271.8 Hz), 44.7, 15.2, 14.7 ppm; **^{19}F{^{1}H} NMR** (376 MHz, $CDCl_3$) δ = -62.38 ppm; **IR** (ATR) ṽ = 2916 (w), 1616 (w), 1446 (w), 1406 (w), 1380 (w), 1325 (s), 1164 (m), 1125 (m), 1108 (m), 1068 (m), 1020 (w), 843 (w), 736 (w), 605 (w) cm^{-1}; **HRMS** (Sicrit plasma/LTQ-Orbitrap) *m/z* = calcd. for $[C_{14}H_{14}F_3]^+$, $[M+H]^+$: 239.1042, found: 239.1040; $\mathbf{R_f}$ (pentane) = 0.56.

4-(2,5-Dimethylcyclopenta-1,4-dien-1-yl)-1,1'-biphenyl (**Cp9**)

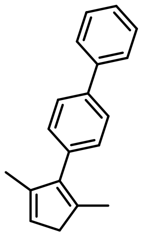

According to *General Procedure 6*, starting from enone **8d** (1.22 mmol) with MeLi·LiBr (1.5 M in Et_2O, 1.5 equiv.) and in THF instead of Et_2O, cyclopentadiene **Cp9** (240 mg, 0.97 mmol, 79% yield) was obtained as a beige solid. Purification was performed by flash column chromatography on silica gel (wet loading with pentane/Et_2O = 20:1, 10 cm column height, gradient: pentane → pentane/Et_2O = 20:1).

^{1}H NMR (400 MHz, $CDCl_3$) δ = 7.67 – 7.61 (m, 4H), 7.48 – 7.43 (m, 2H), 7.38 – 7.32 (m, 1H), 7.32 – 7.27 (m, 2H), 5.99 – 5.96 (m, 1H), 3.00 – 2.97 (m, 2H), 2.04 (s, 3H), 1.95 – 1.92 (m, 3H) ppm; **^{13}C{^{1}H} NMR** (101 MHz, $CDCl_3$) δ = 143.4, 141.8, 141.2, 140.4, 139.2, 136.0, 129.8, 128.9, 127.3, 127.2, 126.9, 124.5, 44.6, 15.4, 14.9 ppm; **IR** (ATR) ṽ = 3057 (w), 3028 (w), 2969 (w), 2911 (w), 2869 (w), 1600 (w), 1486 (m), 1445 (w), 1378 (w), 1111 (w), 1007 (w), 977 (w), 951 (w), 841 (m), 767 (s), 736 (s), 697 (s), 567 (w) cm^{-1}; **HRMS** (Sicrit plasma/LTQ-Orbitrap) *m/z* = calcd. for $[C_{19}H_{19}]^+$, $[M+H]^+$: 247.1481, found: 247.1481; $\mathbf{R_f}$ (pentane/Et_2O, 20:1) = 0.66; **M.p.** = 81-82 °C.

1-(2,5-Dimethylcyclopenta-1,4-dien-1-yl)-4-(methylsulfonyl)benzene (**Cp10**)

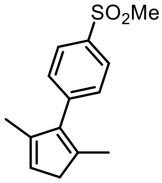

According to *General Procedure 6*, starting from enone **8e** (0.91 mmol) with MeLi·LiBr (1.5 M in Et_2O, 1.5 equiv.) and in THF instead of Et_2O, cyclopentadiene **Cp10** (144 mg, 0.58 mmol, 63% yield) was obtained as a light-yellow oil. Purification was performed by flash column chromatography on silica gel (wet loading with Et_2O, 10 cm column height, isocratic: Et_2O). Remaining cyclopentenone starting material **8e**, that had undergone competitive enolization due to deprotonation rather than addition by the organolithium reagent, could be recovered as well.

^{1}H NMR (400 MHz, $CDCl_3$) δ = 7.97 – 7.93 (m, 2H), 7.42 – 7.38 (m, 2H), 6.00 – 5.97 (m, 1H), 3.10 (s, 3H), 3.01 – 2.98 (m, 2H), 1.99 (s, 3H), 1.88 – 1.85 (m, 3H) ppm; **^{13}C{^{1}H} NMR** (101 MHz, $CDCl_3$) δ = 143.1, 142.4, 142.3, 140.8, 138.5, 130.2, 127.3, 125.3, 44.8, 44.7, 15.2, 14.8 ppm; **IR** (ATR) ṽ = 2925 (w), 1596 (w), 1380 (w), 1310 (s), 1151 (s), 1089 (w), 955 (w), 842 (w), 776 (m), 738 (w), 725 (w), 582 (w), 558 (w), 543 (m), 541 (w) cm^{-1}; **HRMS** (ESI/QTOF) *m/z* = calcd. for $[C_{14}H_{17}O_2S]^+$, $[M+H]^+$: 249.0944, found: 249.0948; **R_f** (Et_2O) = 0.71.

<u>1,3-Di-*tert*-butyl-5-(2,5-dimethylcyclopenta-1,4-dien-1-yl)benzene</u> (**Cp11**)

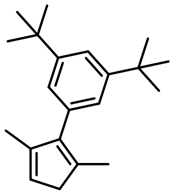

According to *General Procedure 6*, starting from enone **8f** (2.60 mmol) with MeLi·LiBr (1.5 M in Et_2O, 1.5 equiv.), cyclopentadiene **Cp11** (614 mg, 2.17 mmol, 84% yield) was obtained as a colourless liquid. Purification was performed by flash column chromatography on silica gel (wet loading with pentane, 10 cm column height, isocratic: pentane).

^{1}H NMR (400 MHz, $CDCl_3$) δ = 7.32 (t, *J* = 1.9 Hz, 1H), 7.06 (d, *J* = 1.9 Hz, 2H), 5.99 – 5.95 (m, 1H), 3.00 – 2.94 (m, 2H), 2.03 (s, 3H), 1.94 – 1.90 (m, 3H), 1.35 (s, 18H) ppm; **^{13}C{^{1}H} NMR** (101 MHz, $CDCl_3$) δ = 150.1, 143.8, 143.1, 139.7, 135.7, 124.5, 123.7, 120.2, 44.5, 35.0, 31.7, 15.5, 15.0 ppm; **IR** (ATR) ṽ = 2960 (s), 2905 (w), 2867 (w), 1591 (w), 1475 (w), 1459 (w), 1438 (w), 1391 (w), 1377 (w), 1361 (m), 1247 (m), 1202 (w), 1175 (w), 1002 (w), 900 (w), 875 (m), 859, 837 (w), 757 (w), 731 (m), 720 (m), 697 (w) cm^{-1}; **HRMS** (ESI/APCI) *m/z* = calcd. for $[C_{21}H_{31}]^+$, $[M+H]^+$: 283.2420, found: 283.2424; **R_f** (pentane) = 0.64.

<u>1-(2,5-Dimethylcyclopenta-1,4-dien-1-yl)-3,5-bis(trifluoromethyl)benzene</u> (**Cp12**)

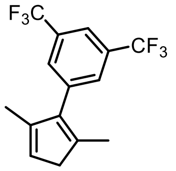

According to *General Procedure 6*, starting from enone **8g** (1.20 mmol) with MeLi·LiBr (1.5 M in Et_2O, 1.5 equiv.), cyclopentadiene **Cp12** (282 mg, 0.92 mmol, 77% yield) was obtained as a colourless liquid. Purification was performed by flash column chromatography on silica gel (wet loading with pentane, 10 cm column height, isocratic: pentane).

^{1}H NMR (400 MHz, $CDCl_3$) δ = 7.82 – 7.78 (m, 1H), 7.67 – 7.63 (m, 2H), 6.02 – 5.98 (m, 1H), 3.02 – 2.98 (m, 2H), 2.00 (s, 3H), 1.88 – 1.84 (m, 3H) ppm; **^{13}C{^{1}H} NMR** (101 MHz, $CDCl_3$) δ = 142.8, 141.9, 139.9, 139.1, 131.5 (q, *J* = 33.0 Hz), 129.4 (q, *J* = 3.6 Hz), 125.6, 123.6 (q, *J* = 272.8 Hz), 120.4 (hept, *J* = 3.7 Hz), 44.8, 15.0, 14.7 ppm; **^{19}F{^{1}H} NMR** (376 MHz, $CDCl_3$) δ = -62.85 ppm; **IR** (ATR) ṽ = 2918 (w), 1393 (w), 1379 (w), 1327 (w), 1275 (s), 1242 (w), 1168 (m), 1128 (s), 1107 (m), 1040 (w), 895 (m), 845 (w), 708 (w), 693 (w), 677 (w) cm^{-1}; **HRMS** (Sicrit plasma/LTQ-Orbitrap) *m/z* = calcd. for $[C_{15}H_{12}F_6]^+$, $[M]^+$: 306.0838, found: 306.0838; **R_f** (pentane) = 0.59.

<u>1-(2,5-Dimethylcyclopenta-1,4-dien-1-yl)-3,5-difluorobenzene</u> (**Cp13**)

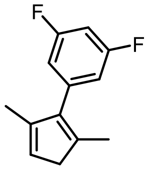

According to *General Procedure 6*, starting from enone **8h** (1.31 mmol) with MeLi·LiBr (1.5 M in Et_2O, 1.5 equiv.), cyclopentadiene **Cp13** (134 mg, 0.65 mmol, 50% yield) was obtained as a colourless liquid. Purification was performed by flash column chromatography on silica gel (wet loading with pentane/DCM = 20:1, 10 cm column height, isocratic: pentane).

^{1}H NMR (400 MHz, $CDCl_3$) δ = 6.77 – 6.69 (m, 3H), 5.96 – 5.93 (m, 1H), 2.97 – 2.94 (m, 2H), 1.99 (s, 3H), 1.88 – 1.85 (m, 3H) ppm; **^{13}C{^{1}H} NMR** (101 MHz, $CDCl_3$) δ = 162.9 (dd, *J* = 247.7, 13.2 Hz), 142.5, 141.7, 140.5 (t, *J* = 3.1 Hz), 140.4 (t, *J* = 9.8 Hz), 125.0, 112.2 (dd, *J* = 18.2, 6.5 Hz), 102.0 (t, *J* = 25.3 Hz), 44.6, 15.1, 14.7 ppm; **^{19}F{^{1}H} NMR** (376 MHz, $CDCl_3$) δ = -110.84 ppm; **IR** (ATR) ṽ = 2919 (w), 1621 (m), 1589 (s), 1428 (m), 1381 (w), 1358 (w), 1303 (w), 1292 (w), 1117 (s), 985 (m), 860 (m), 839 (w), 817 (w), 735 (w), 671 (w) cm^{-1}; **HRMS** (Sicrit plasma/LTQ-Orbitrap) *m/z* = calcd. for $[C_{13}H_{12}F_2]^+$, $[M]^+$: 206.0902, found: 206.0901; **R_f** (pentane) = 0.52.

7-(2,5-Dimethylcyclopenta-1,4-dien-1-yl)benzo[*b*]thiophene (**Cp14**)

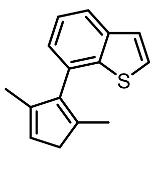

According to *General Procedure 6*, starting from enone **8n** (1.10 mmol) with MeLi·LiBr (1.5 M in Et_2O, 1.5 equiv.), cyclopentadiene **Cp14** (212 mg, 0.94 mmol, 85% yield) was obtained as a yellow solid. Purification was performed by flash column chromatography on silica gel (wet loading with pentane, 10 cm column height, isocratic: pentane/Et_2O = 50:1).

^{1}H NMR (400 MHz, CD_2Cl_2) δ = 7.80 (dd, *J* = 7.9, 1.2 Hz, 1H), 7.47 – 7.43 (m, 1H), 7.42 – 7.38 (m, 2H), 7.14 – 7.10 (m, 1H), 6.02 – 5.99 (m, 1H), 3.15 – 2.96 (m, 2H), 1.87 (s, 3H), 1.77 – 1.74 (m, 3H) ppm; **^{13}C{^{1}H} NMR** (101 MHz, CD_2Cl_2) δ = 143.4, 142.1, 141.5, 140.8, 140.0, 132.3, 127.0, 125.3, 124.8, 124.7, 124.6, 122.5, 44.8, 15.1, 14.7 ppm; **IR** (ATR) ṽ = 2910 (w), 2871 (w), 1457 (w), 1445 (w), 1377 (m), 1327 (w), 1107 (w), 1093 (w), 1039 (w), 1002 (w), 951 (w), 881 (w), 857 (w), 811 (w), 797 (s), 734 (w), 710 (m), 689 (m), 678 (w) cm^{-1}; **HRMS** (Sicrit plasma/LTQ-Orbitrap) *m/z* = calcd. for $[C_{15}H_{15}S]^+$, $[M+H]^+$: 227.0889, found: 227.0887; **R_f** (pentane) = 0.34; **M.p.** = 59-60 °C.

5-(2,5-Dimethylcyclopenta-1,4-dien-1-yl)-1,2,3-trimethoxybenzene (**Cp15**)

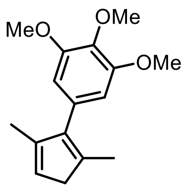

According to *General Procedure 6*, starting from enone **8m** (2.76 mmol) with MeLi·LiBr (1.5 M in Et_2O, 1.5 equiv.), cyclopentadiene **Cp15** (541 mg, 2.08 mmol, 75% yield) was obtained as a yellow oil. Purification was performed by flash column chromatography on silica gel (wet loading with pentane, 10 cm column height, isocratic: pentane/Et_2O = 3:1).

^{1}H NMR (400 MHz, $CDCl_3$) δ = 6.41 (s, 2H), 5.97 – 5.94 (m, 1H), 3.89 (s, 3H), 3.86 (s, 6H), 2.97 – 2.93 (m, 2H), 2.01 (s, 3H), 1.92 – 1.88 (m, 3H) ppm; **^{13}C{^{1}H} NMR** (101 MHz, $CDCl_3$) δ = 153.0, 143.3, 142.4, 140.1, 136.7, 132.6, 124.6, 106.4, 61.0, 56.2, 44.4, 15.4, 14.9 ppm; **IR** (ATR) ṽ = 2937 (w), 1579 (m), 1505 (m), 1452 (w), 1410 (w), 1379 (w), 1359 (w), 1307 (w), 1236 (m), 1182 (w), 1153 (w), 1127 (s), 1009 (w), 837 (w), 733 (w) cm^{-1}; **HRMS** (nanochip-ESI/LTQ-Orbitrap) *m/z* = calcd. for $[C_{16}H_{21}O_3]^+$, $[M+H]^+$: 261.1485, found: 261.1481; **R_f** (pentane/Et_2O, 3:1) = 0.42.

9-(2,5-Dimethylcyclopenta-1,4-dien-1-yl)anthracene (**Cp16**)

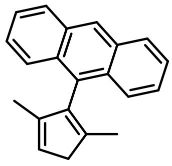

According to *General Procedure 6*, starting from enone **8l** (0.81 mmol) with MeLi·LiBr (1.5 M in Et_2O, 1.5 equiv.) and in THF instead of Et_2O, cyclopentadiene **Cp16** (167 mg, 0.62 mmol, 77% yield) was obtained as a light-yellow solid. Purification was performed

by flash column chromatography on silica gel (wet loading with pentane/Et_2O = 20:1, 10 cm column height, gradient: pentane → pentane/Et_2O = 9:1).

^{1}H NMR (400 MHz, CD_2Cl_2) δ = 8.46 (s, 1H), 8.07 – 8.03 (m, 2H), 7.84 – 7.80 (m, 2H), 7.50 – 7.45 (m, 2H), 7.43 – 7.38 (m, 2H), 6.17 – 6.14 (m, 1H), 3.29 – 3.26 (m, 2H), 1.69 (s, 3H), 1.51 – 1.49 (m, 3H) ppm; **^{13}C{^{1}H} NMR** (101 MHz, CD_2Cl_2) δ = 145.2, 143.1, 139.3, 132.5, 131.9, 130.8, 128.9, 127.0, 126.4, 125.7, 125.6, 124.6, 45.0, 14.7, 14.5 ppm; **IR** (ATR) ṽ = 3053 (w), 2911 (w), 1681 (w), 1441 (m), 1378 (w), 1012 (w), 922 (m), 887 (w), 845 (w), 790 (w), 737 (s), 698 (w), 553 (w) cm^{-1}; **HRMS** (Sicrit plasma/LTQ-Orbitrap) *m/z* = calcd. for $[C_{21}H_{19}]^+$, $[M+H]^+$: 271.1481, found: 271.1480; **R_f** (pentane) = 0.29; **M.p.** = 159-160 °C.

2-(2,5-Dimethylcyclopenta-1,4-dien-1-yl)-1,3-difluorobenzene (**Cp17**)

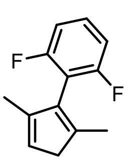

According to *General Procedure 6*, starting from enone **8i** (1.60 mmol) with MeLi·LiBr (1.5 M in Et_2O, 1.5 equiv.), cyclopentadiene **Cp17** (251 mg, 1.22 mmol, 76% yield) was obtained as a light-yellow liquid. Purification was performed by flash column chromatography on silica gel (wet loading with pentane, 10 cm column height, isocratic: pentane).

^{1}H NMR (400 MHz, $CDCl_3$) δ = 7.31 – 7.22 (m, 1H, *overlap with chloroform*), 6.98 – 6.90 (m, 2H), 5.99 – 5.95 (m, 1H), 3.04 – 3.00 (m, 2H), 1.91 (s, 3H), 1.84 – 1.80 (m, 3H) ppm; **^{13}C{^{1}H} NMR** (101 MHz, $CDCl_3$) δ = 160.8 (dd, *J* = 247.0, 8.0 Hz), 144.9, 143.2, 130.1, 128.8 (t, *J* = 10.2 Hz), 124.4, 113.6 (t, *J* = 21.6 Hz), 111.4 (dd, *J* = 19.4, 7.2 Hz), 44.8, 15.0, 14.3 (t, *J* = 1.8 Hz) ppm; **^{19}F{^{1}H} NMR** (376 MHz, $CDCl_3$) δ = -111.23 ppm; **IR** (ATR) ṽ = 2916 (w), 1620 (w), 1580 (w), 1565 (w), 1461 (s), 1377 (w), 1269 (m), 1231 (m), 1035 (w), 993 (s), 979 (m), 951 (w), 858 (w), 784 (s), 731 (m), 555 (w), 545 (w), 506 (w) cm^{-1}; **HRMS** (Sicrit plasma/LTQ-Orbitrap) *m/z* = calcd. for $[C_{13}H_{13}F_2]^+$, $[M+H]^+$: 207.0980, found: 207.0980; **R_f** (pentane) = 0.45.

2-(2,5-Dimethylcyclopenta-1,4-dien-1-yl)-1,3-dimethoxybenzene (**Cp18**)

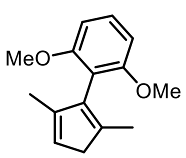

According to *General Procedure 6*, starting from enone **8j** (2.37 mmol) with MeLi·LiBr (1.5 M in Et_2O, 1.5 equiv.), cyclopentadiene **Cp18** (467 mg, 2.03 mmol, 86% yield) was obtained as an off-green solid. Purification was performed by flash column chromatography on silica gel (wet loading with pentane, 10 cm column height, isocratic: pentane/Et_2O = 3:1).

^{1}H NMR (400 MHz, CD_2Cl_2) δ = 7.26 (t, *J* = 8.3 Hz, 1H), 6.63 (d, *J* = 8.4 Hz, 2H), 5.91 – 5.89 (m, 1H), 3.75 (s, 6H), 2.96 – 2.93 (m, 2H), 1.78 (s, 3H), 1.71 – 1.68 (m, 3H) ppm; **^{13}C{^{1}H} NMR** (101 MHz, CD_2Cl_2) δ = 158.8, 145.2, 141.9, 135.1, 128.8, 123.2, 114.1, 104.2, 56.0, 44.5, 14.9, 14.4 ppm; **IR** (ATR) ṽ = 2932 (w), 2872 (w), 2830 (w), 1587 (w), 1468 (m), 1431 (w), 1371 (w), 1278 (w), 1246 (m), 1112 (s), 1040 (w), 973 (w), 950 (w), 782 (w), 733 (w) cm^{-1}; **HRMS** (Sicrit plasma/LTQ-Orbitrap) *m/z* = calcd. for $[C_{15}H_{19}O_2]^+$, $[M+H]^+$: 231.1380, found: 231.1378; **R_f** (pentane/Et_2O, 3:1) = 0.70; **M.p.** = 91-92 °C.

2-(2,5-Dimethylcyclopenta-1,4-dien-1-yl)-1,3-diisopropoxybenzene (**Cp19**)

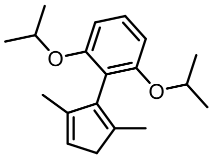

According to *General Procedure 6*, starting from enone **8k** (1.86 mmol) with MeLi·LiBr (1.5 M in Et_2O, 1.5 equiv.), cyclopentadiene **Cp19** (359 mg, 1.25 mmol, 67% yield) was obtained as an orange liquid. Purification was performed by flash column chromatography on silica gel (wet loading with pentane/DCM = 20:1, 10 cm column height, gradient: pentane → pentane/Et_2O = 9:1).

^{1}H NMR (500 MHz, $CDCl_3$) δ = 7.15 (t, *J* = 8.2 Hz, 1H), 6.61 (d, *J* = 8.2 Hz, 2H), 5.87 – 5.84 (m, 1H), 4.22 (hept, *J* = 6.1 Hz, 2H), 2.93 – 2.90 (m, 2H), 1.81 (s, 3H), 1.78 – 1.76 (m, 3H), 1.19 – 1.15 (m, 12H) ppm; **^{13}C{^{1}H} NMR** (126 MHz, $CDCl_3$) δ = 157.5, 145.1, 140.7, 135.5, 127.8, 122.2, 119.3, 109.8, 71.7, 44.2, 22.3, 22.2, 15.3, 14.6 ppm; **IR** (ATR) ṽ = 2975 (m), 2921 (w), 2872 (w), 1576 (w), 1457 (s), 1382 (w), 1335 (w), 1263 (w), 1244 (m), 1117 (s), 1056 (m), 1027 (w), 949 (w), 734 (w) cm^{-1}; **HRMS** (ESI/QTOF) *m/z* = calcd. for $[C_{19}H_{27}O_2]^+$, $[M+H]^+$: 287.2006, found: 287.2001; **R$_f$** (pentane/Et_2O, 9:1) = 0.70.

2-(3,3-Dimethylbut-1-yn-1-yl)-1,3-dimethylcyclopenta-1,3-diene (**Cp20**)

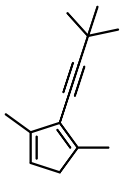

According to *General Procedure 6*, starting from enone **9a** (1.22 mmol) with MeLi·LiBr (1.5 M in Et_2O, 1.5 equiv.), alkynyl-substituted cyclopentadiene **Cp20** (132 mg, 0.76 mmol, 62% yield) was obtained as a colourless liquid. Purification was performed by flash column chromatography on silica gel (wet loading with pentane, 10 cm column height, isocratic: pentane).

^{1}H NMR (400 MHz, $CDCl_3$) δ = 5.80 – 5.77 (m, 1H), 2.87 – 2.84 (m, 2H), 2.09 (s, 3H), 1.96 – 1.94 (m, 3H), 1.31 (s, 9H) ppm; **^{13}C{^{1}H} NMR** (101 MHz, $CDCl_3$) δ = 148.3, 142.5, 125.3, 123.3, 103.7, 73.6, 44.1, 31.5, 28.4, 15.6, 14.4 ppm; **IR** (ATR) ṽ = 2968 (s), 2928 (m), 2909 (m), 2871 (w), 1475 (w), 1455 (w), 1443 (w), 1378 (w), 1362 (m), 1345 (w), 1271 (m), 1247 (w), 1203 (w), 1099 (w), 1003 (w), 950 (w), 885 (w), 859 (w), 733 (m) cm^{-1}; **HRMS** (Sicrit plasma/LTQ-Orbitrap) *m/z* = calcd. for $[C_{13}H_{19}]^+$, $[M+H]^+$: 175.1481, found: 175.1480; **R$_f$** (pentane) = 0.51.

1-((2,5-Dimethylcyclopenta-1,4-dien-1-yl)ethynyl)-3,5-difluorobenzene (**Cp21**)

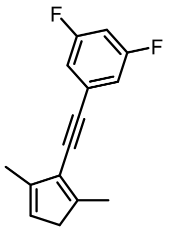

According to *General Procedure 6*, starting from enone **9b** (0.85 mmol) with MeMgBr (3.0 M in Et_2O, 1.5 equiv.) at 0 °C and 10 min/3 h addition/elimination time, alkynyl-substituted cyclopentadiene **Cp21** (157 mg, 0.68 mmol, 80% yield) was obtained as a light-yellow liquid. Purification was performed by flash column chromatography on silica gel (wet loading with pentane, 10 cm column height, isocratic: pentane).

^{1}H NMR (400 MHz, $CDCl_3$) δ = 7.03 – 6.95 (m, 2H), 6.76 (tt, *J* = 9.0, 2.4 Hz, 1H), 5.87 – 5.84 (m, 1H), 2.98 – 2.95 (m, 2H), 2.20 (s, 3H), 2.04 – 2.02 (m, 3H) ppm; **^{13}C{^{1}H} NMR** (101 MHz, $CDCl_3$) δ = 162.9 (dd, *J* = 248.3, 13.4 Hz), 152.8, 141.8, 126.7 (t, *J* = 11.8 Hz), 124.3, 124.0, 114.4 (dd, *J* = 19.2, 7.4 Hz), 104.0 (t, *J* = 25.4 Hz), 92.1 (t, *J* = 4.0 Hz), 86.8, 44.7, 16.0, 14.4 ppm; **^{19}F{^{1}H} NMR** (377 MHz, $CDCl_3$) δ = -110.10 ppm; **IR** (ATR) ṽ = 2911 (w), 2207 (w), 1613 (s), 1584 (s), 1472 (w), 1428 (m), 1377 (w), 1320 (w), 1235 (w),

1169 (w), 1121 (s), 988 (m), 855 (m), 670 (w) cm^{-1}; **HRMS** (Sicrit plasma/LTQ-Orbitrap) *m/z* = calcd. for $[C_{15}H_{12}F_2]^+$, $[M]^+$: 230.0902, found: 230.0901; **R$_f$** (pentane) = 0.48.

2-Chloro-1,3-dimethylcyclopenta-1,3-diene (**Cp22**)

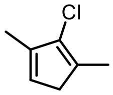

According to *General Procedure 6*, starting from enone **11a** (2.98 mmol) with MeMgBr (3.0 M in Et_2O, 1.5 equiv.) at 0 °C and 10 min/2 h addition/elimination time, chloro-substituted cyclopentadiene **Cp22** (261 mg, 2.03 mmol, 68% yield) was obtained as a light-yellow liquid. Purification was performed by flash column chromatography on silica gel (wet loading with pentane, 10 cm column height, isocratic: pentane). After work-up, extra care should be taken during evaporation (200 mbar at 20 °C) due to the volatility of this cyclopentadiene.

^{1}H NMR (400 MHz, $CDCl_3$) δ = 5.92 – 5.89 (m, 1H), 2.86 – 2.83 (m, 2H), 2.00 (s, 3H), 1.95 – 1.93 (m, 3H) ppm; **^{13}C{^{1}H} NMR** (101 MHz, $CDCl_3$) δ = 141.1, 136.7, 129.8, 123.9, 42.6, 13.80, 13.77 ppm; **IR** (ATR) ṽ = 2983 (w), 2935 (w), 1719 (w), 1688 (w), 1439 (w), 1372 (w), 1330 (m), 1289 (w), 1178 (w), 1106 (s), 1066 (w), 1019 (w), 999 (m), 940 (w), 911 (w), 812 (w), 734 (w) cm^{-1}; **HRMS** (Sicrit plasma/LTQ-Orbitrap) *m/z* = calcd. for $[C_7H_{10}Cl]^+$, $[M+H]^+$: 129.0466, found: 129.0465; **R$_f$** (pentane) = 0.74.

2-Bromo-1,3-dimethylcyclopenta-1,3-diene (**Cp23**)

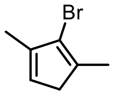

According to *General Procedure 6*, starting from enone **7a** (2.06 mmol) with MeMgBr (3.0 M in Et_2O, 1.5 equiv.) at 0 °C and 10 min/2 h addition/elimination time, bromo-substituted cyclopentadiene **Cp23** (274 mg, 1.59 mmol, 77% yield) was obtained as a colourless liquid. Purification was performed by flash column chromatography on silica gel (wet loading with pentane, 10 cm column height, isocratic: pentane). This bromo-bearing Cp is less stable than the trialkyl-substituted cyclopentadienes (i.e. partial decomposition was observed within 24 hours at 25 °C) and than its chloro-analogue **Cp22**. Therefore, it should either be used immediately or stored in a freezer at -40 °C.

^{1}H NMR (400 MHz, $CDCl_3$) δ = 5.94 – 5.91 (m, 1H), 2.88 – 2.84 (m, 2H), 2.01 (s, 3H), 1.96 – 1.94 (m, 3H) ppm; **^{13}C{^{1}H} NMR** (101 MHz, $CDCl_3$) δ = 142.0, 140.7, 123.9, 120.4, 43.8, 15.5, 15.1 ppm; **IR** (ATR) ṽ = 2977 (w), 2915 (w), 2877 (w), 1624 (w), 1438 (m), 1377 (m), 1327 (w), 1317 (w), 1282 (w), 1202 (w), 1186 (w), 1105 (m), 1058 (w), 1030 (w), 1016 (w), 989 (s), 971 (w), 948 (w), 891 (w), 858 (w), 731 (m), 571 (m) cm^{-1}; **HRMS** (Sicrit plasma/LTQ-Orbitrap) *m/z* = calcd. for $[C_7H_{10}Br]^+$, $[M+H]^+$: 172.9960, found: 172.9960; **R$_f$** (pentane) = 0.70.

((2,5-Dimethylcyclopenta-1,4-dien-1-yl)oxy)benzene (**Cp24**)

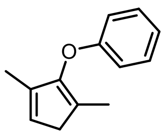

According to *General Procedure 6*, starting from enone **11b** (6.79 mmol) with MeLi·LiBr (1.5 M in Et_2O, 3.0 equiv.), phenoxy-substituted cyclopentadiene **Cp24** (684 mg, 3.67 mmol, 54% yield) was obtained as a light-orange solid. Purification was performed by flash column chromatography on silica gel (wet loading with pentane/DCM = 20:1, 10 cm column height, isocratic: pentane/Et_2O = 20:1).

^{1}H NMR (600 MHz, CD_2Cl_2) δ = 7.29 – 7.24 (m, 2H), 6.99 – 6.95 (m, 1H), 6.92 – 6.89 (m, 2H), 5.95 – 5.93 (m, 1H), 2.89 – 2.87 (m, 2H), 1.82 (s, 3H), 1.71 – 1.69 (m, 3H) ppm; **^{13}C{^{1}H} NMR** (151 MHz, CD_2Cl_2) δ =

158.5, 150.7, 139.6, 129.8, 125.4, 124.7, 121.8, 115.6, 40.5, 12.8, 11.8 ppm; **IR** (ATR) ṽ = 2913 (w), 1655 (w), 1596 (w), 1489 (m), 1446 (w), 1383 (w), 1346 (w), 1235 (s), 1215 (m), 1162 (w), 1128 (w), 1002 (w), 837 (w), 749 (w), 690 (w) cm^{-1}; **HRMS** (Sicrit plasma/LTQ-Orbitrap) *m/z* = calcd. for $[C_{13}H_{15}O]^+$, $[M+H]^+$: 187.1117, found: 187.1116; **R$_f$** (pentane) = 0.41; **M.p.** = 43-44 °C.

(2,5-Dimethylcyclopenta-1,4-dien-1-yl)(phenyl)sulfane (**Cp25**)

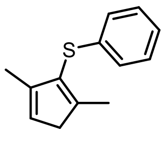

According to *General Procedure 6*, starting from enone **11c** (1.70 mmol) with MeLi·LiBr (1.5 M in Et_2O, 1.5 equiv.), sulfide-bearing cyclopentadiene **Cp25** (298 mg, 1.47 mmol, 87% yield) was obtained as a smelly orange liquid. Purification was performed by flash column chromatography on silica gel (wet loading with pentane/Et_2O = 20:1, 10 cm column height, isocratic: pentane/Et_2O = 9:1).

^{1}H NMR (400 MHz, $CDCl_3$) δ = 7.24 – 7.18 (m, 2H), 7.12 – 7.06 (m, 3H), 6.00 – 5.97 (m, 1H), 3.06 – 3.04 (m, 2H), 2.17 (s, 3H), 1.83 – 1.80 (m, 3H) ppm; **^{13}C{^{1}H} NMR** (101 MHz, $CDCl_3$) δ = 153.3, 144.3, 138.2, 129.9, 128.9, 126.5, 124.9, 124.2, 44.6, 15.5, 14.4 ppm; **IR** (ATR) ṽ = 2913 (w), 1580 (w), 1476 (m), 1438 (m), 1374 (w), 1083 (w), 1024 (w), 999 (w), 949 (w), 856 (w), 735 (s), 689 (s), 492 (w), 466 (w) cm^{-1}; **HRMS** (Sicrit plasma/LTQ-Orbitrap) *m/z* = calcd. for $[C_{13}H_{15}S]^+$, $[M+H]^+$: 203.0889, found: 203.0896; **R$_f$** (pentane/Et_2O, 9:1) = 0.81.

((2,5-Dimethylcyclopenta-1,4-dien-1-yl)sulfonyl)benzene (**Cp26**) *and* ((2,5-Dimethylcyclopenta-2,4-dien-1-yl)sulfonyl)benzene (**Cp26'**)

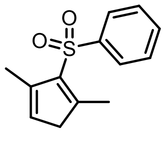

Cp26
major

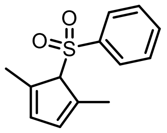

Cp26'
minor

According to *General Procedure 6*, starting from enone **11d** (0.88 mmol) with MeMgBr (3.0 M in Et_2O, 1.5 equiv.) at 0 °C in THF instead of Et_2O and 10 min/30 min addition/elimination time, sulfone-bearing cyclopentadienes **Cp26** and **Cp26'** (111 mg, 0.47 mmol, 54% yield, 1.5:1 regiomeric ratio) were obtained as a white solid. Purification was performed by flash column chromatography on silica gel (wet loading with DCM, 15 cm column height, isocratic: DCM). Remaining cyclopentenone starting material **11d**, that had undergone competitive enolization due to deprotonation rather than addition by the Grignard reagent, could be recovered as well (45%).

^{1}H NMR (400 MHz, $CDCl_3$) *Major regiomer:* δ = 7.92 – 7.88 (m, 2H), 7.61 – 7.49 (m, 3H), 5.87 – 5.83 (m, 1H), 3.06 – 3.03 (m, 2H), 2.50 (s, 3H), 1.99 – 1.96 (m, 3H) ppm; *Minor regiomer:* δ = 7.72 – 7.67 (m, 2H), 7.61 – 7.49 (m, 1H), 7.43 – 7.37 (m, 2H), 5.87 – 5.83 (m, 2H), 4.19 (s, 1H), 2.22 (s, 6H) ppm; **^{13}C{^{1}H} NMR** (101 MHz, $CDCl_3$) δ = 158.2, 142.6, 139.6, 138.4, 137.0, 135.3, 133.7, 133.4, 133.1, 129.2, 128.7, 128.0, 127.1, 126.2, 78.4, 46.6, 16.0, 15.9, 15.4 ppm; **IR** (ATR) ṽ = 2922 (w), 1446 (w), 1304 (s), 1168 (m), 1149 (s), 1093 (w), 1084 (w), 757 (w), 727 (m), 689 (w), 623 (w), 598 (m), 546 (m), 533 (w) cm^{-1}; **HRMS** (APCI/QTOF) *m/z* = calcd. for $[C_{13}H_{15}O_2S]^+$, $[M+H]^+$: 235.0787, found: 235.0789; **R$_f$** (DCM) = 0.46; **M.p.** = 67-71 °C.

<u>1-(2-Bromo-3-methylcyclopenta-1,3-dien-1-yl)-4-chlorobenzene</u> (**Cp27**) *and*
<u>1-(5-Bromo-4-methylcyclopenta-1,4-dien-1-yl)-4-chlorobenzene</u> (**Cp27'**)

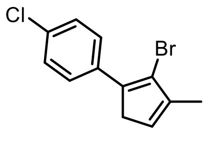

Cp27
major

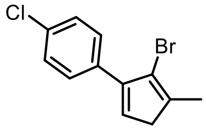

Cp27'
minor

According to *General Procedure 6*, starting from enone **7a** (1.55 mmol) with (4-chlorophenyl)magnesium bromide (1.0 M in 2-Me-THF, 1.5 equiv.) at 0 °C in THF instead of Et_2O and 10 min/24 h addition/elimination time, cyclopentadienes **Cp27** and **Cp27'** (401 mg, 1.49 mmol, 96% yield, 4:1 regiomeric ratio) were obtained as a white crystalline solid. Purification was performed by flash column chromatography on silica gel (wet loading with pentane, 10 cm column height, isocratic: pentane).

^{1}H NMR (400 MHz, $CDCl_3$) *Major regiomer:* δ = 7.68 – 7.64 (m, 2H), 7.36 – 7.32 (m, 2H), 6.17 – 6.14 (m, 1H), 3.33 – 3.30 (m, 2H), 2.05 – 2.02 (m, 3H) ppm; *Minor regiomer:* δ = 7.44 – 7.41 (m, 2H), 7.36 – 7.32 (m, 2H), 6.30 (t, *J* = 1.8 Hz, 1H), 3.05 (d, *J* = 1.8 Hz, 2H), 2.10 (s, 3H) ppm; **^{13}C{^{1}H} NMR** (151 MHz, $CDCl_3$) *Major regiomer:* δ = 143.7, 140.4, 134.1, 133.0, 128.6, 128.5, 126.2, 121.2, 42.8, 15.3 ppm; **IR** (ATR) ṽ = 2915 (w), 1487 (s), 1443 (w), 1375 (w), 1315 (w), 1094 (s), 1013 (w), 983 (w), 948 (m), 889 (w), 873 (w), 821 (s), 739 (w), 661 (w), 528 (w), 499 (w) cm^{-1}; **HRMS** (APPI/LTQ-Orbitrap) *m/z* = calcd. for $[C_{12}H_9BrCl]^-$, $[M-H]^-$: 266.9582, found: 266.9591; **R_f** (pentane) = 0.52; **M.p.** = 42-43 °C.

<u>(3-Isopropyl-2-methylcyclopenta-1,3-dien-1-yl)benzene</u> (**Cp28**) *and*
<u>(4-Isopropyl-5-methylcyclopenta-1,4-dien-1-yl)benzene</u> (**Cp28'**)

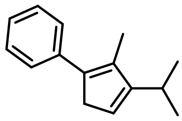

Cp28
major

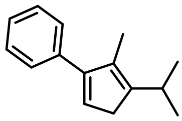

Cp28'
minor

According to *General Procedure 6*, starting from enone **5a** (0.55 mmol) with PhLi (1.9 M in Bu_2O, 2.0 equiv.), cyclopentadienes **Cp28** and **Cp28'** (94 mg, 0.47 mmol, 86% yield, 9:1 regiomeric ratio) were obtained as a light-yellow liquid. Purification was performed by flash column chromatography on silica gel (wet loading with pentane, 15 cm column height, isocratic: pentane). The major regiomer's structure was confirmed *via* 2D NOESY to be **Cp28** due to interaction between the isopropyl group and the Cp ring's olefinic hydrogen.

^{1}H NMR (400 MHz, $CDCl_3$) *Major regiomer:* δ = 7.38 – 7.35 (m, 4H), 7.24 – 7.18 (m, 1H), 6.08 – 6.06 (m, 1H), 3.26 – 3.23 (m, 2H), 2.73 – 2.60 (m, 1H), 2.13 (t, *J* = 1.9 Hz, 3H), 1.20 (d, *J* = 6.8 Hz, 6H) ppm; **^{13}C{^{1}H} NMR** (101 MHz, $CDCl_3$) *Major regiomer:* δ = 156.2, 140.7, 138.3, 137.9, 128.4, 128.0, 125.9, 122.6, 42.2, 27.3, 22.7, 12.8 ppm; **IR** (ATR) ṽ = 2960 (m), 2930 (w), 2870 (w), 1599 (w), 1492 (w), 1379 (w), 1269 (w), 1061 (w), 1045 (w), 980 (w), 910 (w), 756 (m), 738 (w), 697 (s) cm^{-1}; **HRMS** (HESI/LTQ-Orbitrap) *m/z* = calcd. for $[C_{15}H_{19}]^+$, $[M+H]^+$: 199.1481, found: 199.1483; **R_f** (pentane) = 0.43.

5,5'-(2-Methylcyclopenta-1,3-diene-1,3-diyl)bis(1,3-bis(trifluoromethyl)benzene) (**Cp29**) *and* 5,5'-(2-Methylcyclopenta-3,5-diene-1,3-diyl)bis(1,3-bis(trifluoromethyl)benzene) (**Cp29'**)

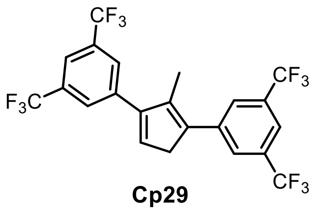

Cp29
major

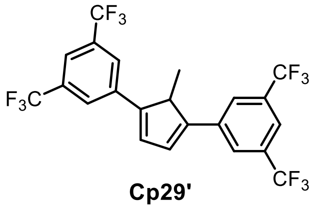

Cp29'
minor

Step 1. According to *General Procedure 6*, starting from enone **5b** (0.92 mmol) with (3,5-bis(trifluoromethyl)phenyl)magnesium bromide (0.5 M in THF, 1.2 equiv.) and 24 h/18 h addition/elimination time, not the cyclopentadiene, but the cyclopentenol intermediate was obtained.

Step 2. To affect dehydration, the crude cyclopentenol was redissolved in toluene (10 mL/mmol), 4-toluenesulfonic acid monohydrate (PTSA, 174 mg, 1 equiv.) was added, and the reaction mixture was stirred in a heating block at 80 °C under air for 13 hours. After cooling to room temperature (25 °C), the mixture was diluted with pentane, sequentially washed with water, sat. aq. $NaHCO_3$ solution, and brine, then dried over $MgSO_4$, filtered, and concentrated under reduced pressure. Purification was performed by flash column chromatography on silica gel (wet loading with pentane, 10 cm column height, isocratic: pentane), affording cyclopentadienes **Cp29** and **Cp29'** (277 mg, 0.55 mmol, 60% yield over 2 steps, 3:1 regiomeric ratio) as a light-yellow solid. Notably, in the ^{13}C NMR spectrum, not all carbon signals are fully resolved due to abundant multiplets because of C-F coupling as well as overlap within and between both regiomers.

^{1}H NMR (400 MHz, $CDCl_3$) *Major regiomer:* δ = 7.92 – 7.75 (m, 6H), 6.67 – 6.65 (t, *J* = 1.6 Hz, 1H), 3.56 – 3.53 (m, 2H), 2.17 (t, *J* = 2.0 Hz, 3H) ppm; *Minor regiomer:* δ = 7.92 – 7.75 (m, 6H), 7.06 (d, *J* = 0.8 Hz, 2H), 4.04 (q, *J* = 7.7 Hz, 1H), 1.23 (d, *J* = 7.7 Hz, 3H) ppm; **^{13}C{^{1}H} NMR** (101 MHz, $CDCl_3$) δ = 151.5, 148.1, 140.6, 139.5, 139.2, 139.1, 138.6, 136.7, 132.9, 132.4 (q, *J* = 33.2 Hz), 132.2, 131.8, 131.5, 129.7, 128.3 (q, *J* = 3.7 Hz), 127.8 (q, *J* = 3.6 Hz), 126.0 (q, *J* = 4.0 Hz), 124.9, 124.8, 122.2, 122.1, 121.4 (hept, *J* = 3.9 Hz), 120.7 (hept, *J* = 3.8 Hz), 120.2 (hept, *J* = 4.0 Hz), 45.6, 43.1, 16.9, 14.1 ppm; **^{19}F{^{1}H} NMR** (376 MHz, $CDCl_3$) δ = -62.86, -62.89, -62.93, -62.97 ppm; **IR** (ATR) ṽ = 1383 (w), 1350 (w), 1325 (w), 1277 (s), 1169 (m), 1130 (s), 1077 (w), 897 (w), 845 (w), 706 (w), 683 (w) cm^{-1}; **HRMS** (APPI/LTQ-Orbitrap) *m/z* = calcd. for $[C_{22}H_{12}F_{12}]^+$, $[M]^+$: 504.0742, found: 504.0755; **R_f** (pentane) = 0.73; **M.p.** = 27-28 °C.

2-(2-(4-Methoxyphenyl)-3-methylcyclopenta-1,3-dien-1-yl)thiophene (**Cp30**) *and*
2-(5-(4-Methoxyphenyl)-4-methylcyclopenta-1,4-dien-1-yl)thiophene (**Cp30'**)

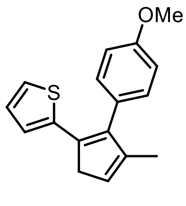

Cp30
major

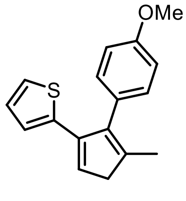

Cp30'
minor

According to *General Procedure 6*, starting from enone **8b** (0.43 mmol) with 2-thienyllithium (1.0 M in THF, 1.5 equiv.), cyclopentadienes **Cp30** and **Cp30'** (106 mg, 0.40 mmol, 92% yield, 18:1 regiomeric ratio) were obtained as a yellow solid. Purification was performed by flash column chromatography on silica gel (wet loading with pentane, 10 cm column height, isocratic: pentane/Et_2O = 9:1). The major regiomer's structure was confirmed *via* 2D NOESY to be **Cp30** due to interaction between the methyl group and the Cp ring's olefinic hydrogen. A suitable crystal for X-ray analysis of **Cp30** (**Figure S3**) was obtained by slow evaporation of a concentrated solution in pentane.

^{1}H NMR (400 MHz, $CDCl_3$) *Major regiomer:* δ = 7.21 – 7.17 (m, 2H), 7.01 – 6.96 (m, 3H), 6.87 – 6.83 (m, 2H), 6.05 – 6.02 (m, 1H), 3.87 (s, 3H), 3.47 – 3.44 (m, 2H), 1.80 – 1.78 (m, 3H) ppm; **^{13}C{^{1}H} NMR** (101 MHz, $CDCl_3$) *Major regiomer:* δ = 159.3, 144.9, 143.2, 140.6, 135.5, 130.7, 129.0, 126.7, 125.0, 123.7, 123.6, 114.3, 55.3, 42.4, 14.8 ppm; **IR** (ATR) ṽ = 2910 (w), 1607 (w), 1515 (m), 1497 (w), 1463 (w), 1441 (w), 1285 (w), 1244 (s), 1174 (w), 1035 (m), 947 (w), 829 (m), 695 (m), 595 (w) cm^{-1}; **HRMS** (Sicrit plasma/LTQ-Orbitrap) *m/z* = calcd. for $[C_{17}H_{17}OS]^+$, $[M+H]^+$: 269.0995, found: 269.0994; **R_f** (pentane/Et_2O, 9:1) = 0.59; **M.p.** = 97-98 °C; **XRD** (Cu*Kα*, R_1 = 4.97%) CCDC: 2479661.

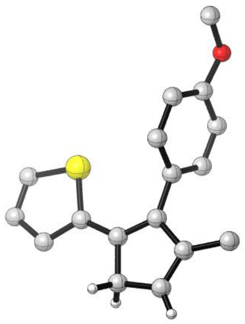

Figure S3. Solid-state X-ray structure of 1,2,3-Cp **Cp30** (CCDC: 2479661) showing 50% probability thermal ellipsoids. Hydrogen atoms (except for on the Cp ring), solvent molecules, and disorder are omitted for clarity.

<u>1-Methoxy-4-(5-methyl-2-(phenylethynyl)cyclopenta-1,4-dien-1-yl)benzene</u> (**Cp31**) *and* <u>1-Methoxy-4-(2-methyl-5-(phenylethynyl)cyclopenta-1,4-dien-1-yl)benzene</u> (**Cp31'**)

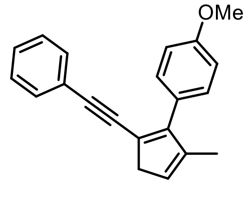

Cp31
major

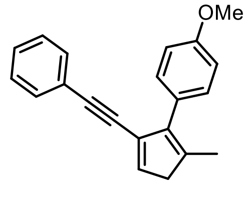

Cp31'
minor

According to *General Procedure 6*, starting from enone **8b** (0.39 mmol) with (phenylethynyl)magnesium bromide (1.0 M in THF, 2.0 equiv.) at 0 °C and 1 h/1 h addition/elimination time, cyclopentadienes **Cp31** and **Cp31'** (53 mg, 0.19 mmol, 48% yield, 3:1 regiomeric ratio) were obtained as a light-brown oil. Purification was performed by flash column chromatography on silica gel (wet loading with pentane/DCM = 20:1, 10 cm column height, isocratic: pentane/Et_2O = 9:1). Remaining cyclopentenone starting material **8b**, that had undergone competitive enolization due to deprotonation rather than addition by the Grignard reagent, could be recovered as well. The major regiomer's structure was confirmed *via* 2D NOESY to be **Cp31** due to interaction between the methyl group and the Cp ring's olefinic hydrogen.

^{1}H NMR (400 MHz, CD_2Cl_2) *Major regiomer:* δ = 7.56 – 7.48 (m, 2H), 7.41 – 7.24 (m, 5H), 7.01 – 6.95 (m, 2H), 6.20 – 6.16 (m, 1H), 3.85 (s, 3H), 3.28 – 3.25 (m, 2H), 2.04 – 2.01 (m, 3H) ppm; *Minor regiomer:* δ = 7.56 – 7.48 (m, 2H), 7.41 – 7.24 (m, 5H), 7.01 – 6.95 (m, 2H), 5.00 – 4.97 (m, 1H), 3.84 (s, 3H), 2.79 – 2.75 (m, 2H), 1.42 (s, 3H) ppm; **^{13}C{^{1}H} NMR** (101 MHz, CD_2Cl_2) δ = 159.8, 159.6, 154.4, 152.7, 149.1, 143.3, 132.9, 131.8, 131.5, 130.8, 130.5, 130.0, 129.8, 128.9, 128.75, 128.71, 128.65, 128.2, 128.1, 125.8, 124.4, 123.8, 122.2, 122.0, 113.8, 105.1, 93.7, 88.3, 81.9, 74.0, 55.6, 44.3, 35.0, 30.5, 15.5 ppm; **IR** (ATR) ṽ = 2952 (w), 1607 (w), 1508 (m), 1486 (w), 1463 (w), 1442 (w), 1289 (w), 1240 (s), 1176 (m), 1035 (w), 832 (m), 755 (m), 690 (m), 530 (w) cm^{-1}; **HRMS** (Sicrit plasma/LTQ-Orbitrap) *m/z* = calcd. for $[C_{21}H_{19}O]^+$, $[M+H]^+$: 287.1430, found: 287.1430; **R_f** (pentane/Et_2O, 9:1) = 0.53.

<u>1,3-Diisopropyl-2-methylcyclopenta-1,3-diene</u> (**Cp32**)

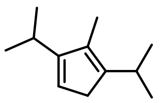

According to *General Procedure 6*, starting from enone **5a** (1.23 mmol) with isopropyllithium (0.7 M in pentane, 3.0 equiv.), cyclopentadiene **Cp32** (65 mg, 0.40 mmol, 32% yield) was obtained as a light-yellow liquid. Purification was performed by flash column chromatography on silica gel (wet loading with pentane, 10 cm column height, isocratic: pentane). Remaining cyclopentenone starting material **5a**, that had undergone competitive enolization due to deprotonation rather than addition by the organolithium reagent, could be recovered as well.

^{1}H NMR (600 MHz, $CDCl_3$) δ = 5.85 – 5.82 (m, 1H), 2.89 (hept, *J* = 6.9 Hz, 1H), 2.79 – 2.76 (m, 2H), 2.61 – 2.53 (m, 1H), 1.87 (t, *J* = 1.7 Hz, 3H), 1.13 (d, *J* = 6.8 Hz, 6H), 1.08 (d, *J* = 7.0 Hz, 6H) ppm; **^{13}C{^{1}H} NMR** (151 MHz, $CDCl_3$) δ = 155.3, 148.2, 133.6, 119.8, 37.4, 27.4, 27.3, 23.3, 22.7, 10.8 ppm; **IR** (ATR) ṽ = 2959 (s), 2924 (m), 2870 (m), 1465 (w), 1380 (w), 1361 (w), 1030 (w), 971 (w), 884 (w), 747 (w), 693 (w) cm^{-1}; **HRMS** (Sicrit plasma/LTQ-Orbitrap) *m/z* = calcd. for $[C_{12}H_{21}]^+$, $[M+H]^+$: 165.1638, found: 165.1637; **R_f** (pentane) = 0.69.

1-(3-Cyclohexyl-2-methylcyclopenta-1,3-dien-1-yl)-3,5-bis(trifluoromethyl)benzene (**Cp33**) *and* 1-(4-Cyclohexyl-5-methylcyclopenta-1,4-dien-1-yl)-3,5-bis(trifluoromethyl)benzene (**Cp33'**)

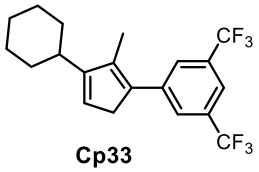

Cp33
major

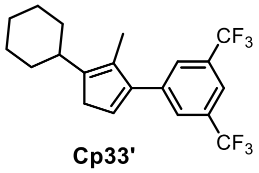

Cp33'
minor

According to *General Procedure 6*, starting from enone **5b** (0.35 mmol) with cyclohexylmagnesium chloride (1.0 M in 2-Me-THF, 2.0 equiv.) at 0 °C and 20 min/7 h addition/elimination time, cyclopentadienes **Cp33** and **Cp33'** (45 mg, 0.12 mmol, 34% yield, 6.5:1 regiomeric ratio) were obtained as a light-yellow liquid. Purification was performed by flash column chromatography on silica gel (wet loading with pentane, 10 cm column height, isocratic: pentane). Remaining cyclopentenone starting material **5b**, that had undergone competitive enolization due to deprotonation rather than addition by the Grignard reagent, could be recovered as well. The major regiomer's structure was confirmed *via* 2D NOESY to be **Cp33** due to interaction between the cyclohexyl group and the Cp ring's olefinic hydrogen.

^{1}H NMR (400 MHz, $CDCl_3$) *Major regiomer:* δ = 7.77 – 7.74 (m, 2H), 7.68 (s, 1H), 6.15 – 6.11 (m, 1H), 3.29 – 3.26 (m, 2H), 2.31 – 2.22 (m, 1H), 2.13 (t, *J* = 2.0 Hz, 3H), 1.95 – 1.63 (m, 6H), 1.45 – 1.19 (m, 6H) ppm; *Minor regiomer:* δ = 7.82 – 7.77 (m, 3H), 6.35 (t, *J* = 1.6 Hz, 1H), 3.03 – 3.00 (m, 2H), 2.62 – 2.52 (m, 1H), 1.95 – 1.63 (m, 6H), 1.45 – 1.19 (m, 6H) ppm; **^{13}C{^{1}H} NMR** (101 MHz, $CDCl_3$) *Major regiomer:* δ = 155.3, 141.6, 140.1, 137.4, 131.6 (q, *J* = 32.9 Hz), 127.6 (q, *J* = 3.8 Hz), 125.0, 123.7 (q, *J* = 272.6 Hz), 119.2 (hept, *J* = 3.9 Hz), 42.2, 37.4, 33.4, 26.9, 12.9 ppm; **^{19}F{^{1}H} NMR** (376 MHz, $CDCl_3$) *Major regiomer:* δ = -62.93 ppm; *Minor regiomer:* δ = -62.88 ppm; **IR** (ATR) ṽ = 2927 (m), 2854 (w), 1450 (w), 1389 (w), 1311 (w), 1277 (s), 1174 (m), 1133 (s), 895 (w), 844 (w), 704 (w), 683 (w) cm^{-1}; **HRMS** (APCI/QTOF) *m/z* = calcd. for $[C_{20}H_{21}F_6]^+$, $[M+H]^+$: 375.1542, found: 375.1529; **R_f** (pentane) = 0.67.

1-(5-(*tert*-Butyl)-2-methylcyclopenta-1,4-dien-1-yl)-4-methoxybenzene (**Cp34**) *and*
1-(2-(*tert*-Butyl)-5-methylcyclopenta-1,4-dien-1-yl)-4-methoxybenzene (**Cp34'**)

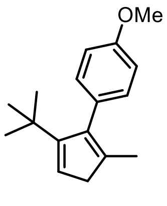

Cp34
major

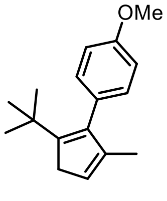

Cp34'
minor

According to *General Procedure 6*, starting from enone **8b** (0.51 mmol) with *tert*-butyllithium (1.9 M in pentane, 2.0 equiv.), cyclopentadienes **Cp34** and **Cp34'** (74 mg, 0.30 mmol, 60% yield, 1.2:1 regiomeric ratio) were obtained as a light-yellow liquid. Purification was performed by flash column chromatography on silica gel (wet loading with pentane, 10 cm column height, isocratic: pentane/Et_2O = 20:1). Remaining cyclopentenone starting material **8b**, that had undergone competitive enolization due to deprotonation rather than addition by the organolithium reagent, could be recovered as well. Both regiomers' structure was confirmed *via* 2D NOESY through the interaction between the methyl group and either the Cp ring's CH_2 moiety (for **Cp34**) or its olefinic hydrogen (for **Cp34'**).

^{1}H NMR (400 MHz, $CDCl_3$) *Major regiomer:* δ = 7.10 – 7.03 (m, 2H), 6.91 – 6.86 (m, 2H), 5.92 (t, *J* = 1.8 Hz, 1H), 3.84 (s, 3H), 2.87 (d, *J* = 1.8 Hz, 2H), 1.70 (s, 3H), 1.03 (s, 9H) ppm; *Minor regiomer:* δ = 7.10 – 7.03 (m, 2H), 6.91 – 6.86 (m, 2H), 5.91 – 5.88 (m, 1H), 3.83 (s, 3H), 3.05 – 3.02 (m, 2H), 1.62 – 1.59 (m, 3H), 1.03 (s, 9H) ppm; **^{13}C{^{1}H} NMR** (151 MHz, $CDCl_3$) δ = 158.3, 157.7, 152.3, 145.1, 142.5, 140.8, 132.3, 131.9, 131.6, 130.7, 122.7, 121.0, 113.25, 113.21, 55.3, 43.0, 41.1, 34.3, 33.6, 31.8, 30.4, 15.0, 14.4 ppm; **IR** (ATR) ṽ = 2953 (m), 2906 (w), 2868 (w), 1607 (w), 1507 (m), 1464 (w), 1442 (w), 1360 (w), 1283 (m), 1243 (s), 1173 (w), 1039 (w), 831 (m) cm^{-1}; **HRMS** (Sicrit plasma/LTQ-Orbitrap) *m/z* = calcd. for $[C_{17}H_{23}O]^+$, $[M+H]^+$: 243.1743, found: 243.1742; **R$_f$** (pentane/Et_2O, 20:1) = 0.63.

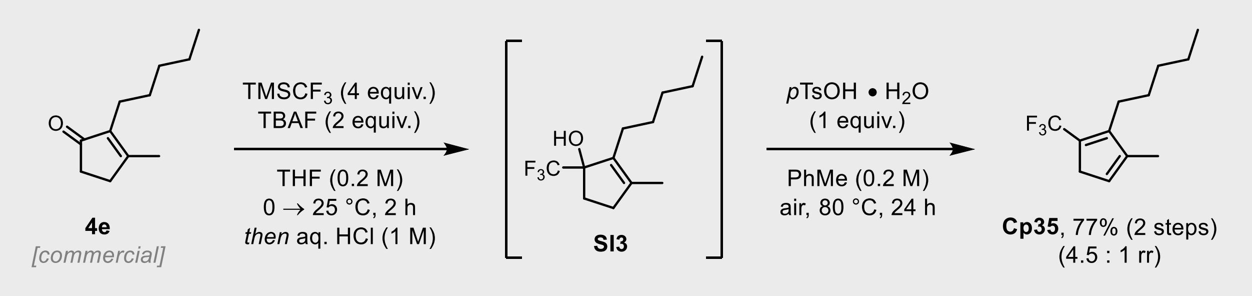

Scheme S14. Telescoped trifluoromethylation-elimination of enones towards CF_3-substituted 1,2,3-Cps.

<u>3-Methyl-2-pentyl-1-(trifluoromethyl)cyclopenta-1,3-diene</u> (**Cp35**) *and*
<u>1-Methyl-2-pentyl-3-(trifluoromethyl)cyclopenta-1,3-diene</u> (**Cp35'**)

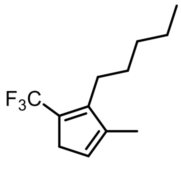

Cp35
major

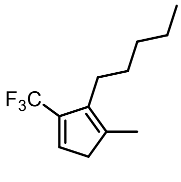

Cp35'
minor

According to the following adaptatation of a reported procedure (**Scheme S14**),[28] trifluoromethyl-bearing cyclopentadienes **Cp35** and **Cp35'** were obtained.

Step 1. At 0 °C (ice bath) and under an atmosphere of nitrogen, trifluoro-methyltrimethylsilane (Ruppert-Prakash reagent, 2.73 mL, 4.0 equiv.) was added to a solution of commercial dihydrojasmone **4e** (768 mg, 4.62 mmol, 1.0 equiv.) in anhydrous THF (5 mL/mmol). Next, tetrabutylammonium fluoride solution (TBAF, 1 M in THF, 9.24 mL, 2.0 equiv.) was added to the colourless solution, which turned dark pink. The ice bath was removed, and the reaction mixture was stirred room temperature (25 °C) for 2 hours. After quenching with sat. aq. NH_4Cl solution, aq. HCl solution (1 M, 3 mL/mmol) was added. The mixture was stirred vigorously for 10 min, after which it was extracted with Et_2O (2x). The combined organic layers were sequentially washed with sat. aq. $NaHCO_3$ solution and brine, dried over $MgSO_4$, filtered, and concentrated under reduced pressure, affording not the cyclopentadiene, but cyclopentenol **SI3**.

Step 2. To affect dehydration, the crude cyclopentenol **SI3** was redissolved in toluene (5 mL/mmol), 4-toluenesulfonic acid monohydrate (PTSA, 879 mg, 1 equiv.) was added, and the reaction mixture was stirred in a heating block at 80 °C under air for 24 hours. After cooling to room temperature (25 °C), the mixture was diluted with pentane, sequentially washed with water, sat. aq. $NaHCO_3$ solution, and brine, then dried over $MgSO_4$, filtered, and concentrated under reduced pressure. Purification was performed by flash column chromatography on silica gel (wet loading with pentane, 10 cm column height, isocratic: pentane), affording trifluoromethyl-substituted cyclopentadienes **Cp35** and **Cp35'** (781 mg, 3.58 mmol, 77% yield over 2 steps, 4.5:1 regiomeric ratio) as a yellow liquid. Notably, aside from the described regiomers, two other double bond isomers are also present, although only in a small amount. The major regiomer's structure was confirmed *via* 2D NOESY to be **Cp35** due to interaction between the Cp ring's methyl group and its olefinic hydrogen.

^{1}H NMR (600 MHz, $CDCl_3$) *Major regiomer:* δ = 6.16 – 6.13 (m, 1H), 3.09 – 3.06 (m, 2H), 2.47 – 2.42 (m, 2H), 1.97 – 1.94 (m, 3H), 1.48 – 1.40 (m, 2H), 1.37 – 1.28 (m, 4H), 0.92 – 0.88 (m, 3H) ppm; *Minor regiomer:* δ = 6.66 – 6.63 (m, 1H), 2.96 – 2.93 (m, 2H), 2.34 – 2.30 (m, 2H), 1.97 – 1.94 (m, 3H), 1.48 – 1.40 (m, 2H), 1.37 – 1.28 (m, 4H), 0.92 – 0.88 (m, 3H) ppm; **^{13}C{^{1}H} NMR** (151 MHz, $CDCl_3$) *Major*

regiomer: δ = 152.0 (q, *J* = 4.4 Hz), 143.2, 129.9, 127.2 (q, *J* = 32.6 Hz), 124.4 (q, *J* = 268.7 Hz), 39.0, 32.1, 29.4, 26.5, 22.5, 14.1, 13.7 ppm; **^{19}F{^{1}H} NMR** (376 MHz, $CDCl_3$) *Major regiomer:* δ = -57.22 ppm; *Minor regiomer:* δ = -62.60 ppm; **IR** (ATR) ṽ = 2960 (w), 2931 (w), 2873 (w), 1373 (w), 1325 (w), 1262 (w), 1247 (w), 1159 (m), 1114 (s), 1102 (s), 1061 (w), 1006 (w), 462 (w) cm^{-1}; **HRMS** (APPI/LTQ-Orbitrap) *m/z* = calcd. for $[C_{12}H_{17}F_3]^+$, $[M]^+$: 218.1277, found: 218.1286; **R$_f$** (pentane) = 0.72.

Unsuccessful Attempts of the One-pot Addition-Elimination towards 1,2,3-Cps.

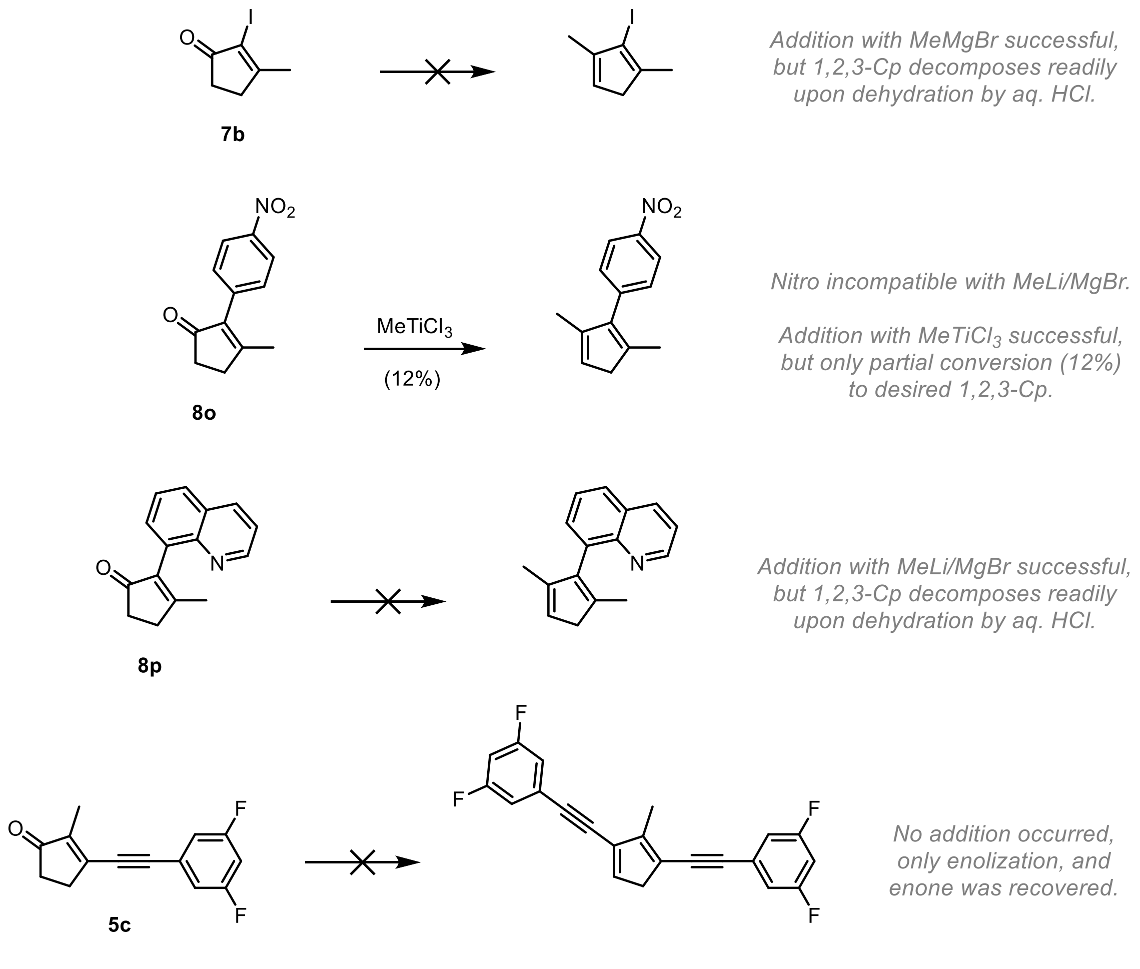

Scheme S15. Brief outline of the few unsuccessful attempts of one-pot addition-eliminations towards 1,2,3-Cps.

4. Diversification of 1,2,3-Cps

4.1 Diels-Alder Cycloaddition towards Norbornadienes

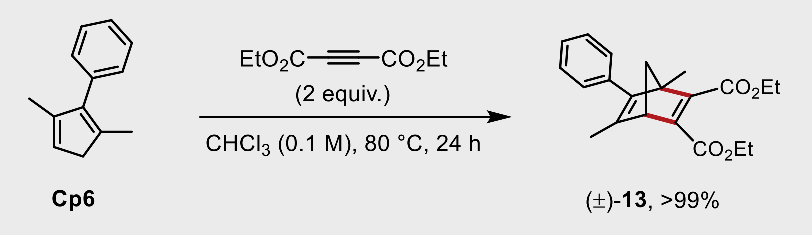

Scheme S16. Conversion of 1,2,3-Cps into substituted norbornadienes *via* Diels-Alder cycloaddition.

Diethyl 1,5-dimethyl-6-phenylbicyclo[2.2.1]hepta-2,5-diene-2,3-dicarboxylate (±)-**13**

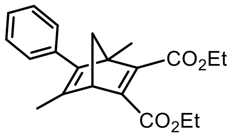

According to the following adaptatation of a reported procedure (**Scheme S16**),[29] racemic diene **13** was obtained.

An oven-dried microwave vial was placed under an atmosphere of nitrogen by Schlenk technique, and charged with a solution of 1,2,3-trisubstituted cyclopentadiene **Cp6** (80 mg, 0.47 mmol, 1.0 equiv.) and diethyl but-2-ynedioate (160 mg, 2.0 equiv) in chloroform (10 mL/mmol). The colourless solution was stirred in a heating block at 80 °C for 24 hours. After cooling to room temperature (25 °C), the reaction mixture was concentrated under reduced pressure and directly purified by flash column chromatography on silica gel (wet loading with pentane, 18 cm column height, gradient: pentane/EtOAc = 20:1 → 10:1), affording racemic norbornadiene **13** (160 mg, 0.47 mmol, quantitative yield) as a colourless oil.

^{1}H NMR (400 MHz, $CDCl_3$) δ = 7.37 – 7.31 (m, 2H), 7.25 – 7.19 (m, 3H), 4.42 – 4.28 (m, 2H), 4.27 – 4.13 (m, 2H), 3.72 (t, *J* = 1.6 Hz, 1H), 2.28 (dd, *J* = 6.7, 1.6 Hz, 1H), 2.20 (dd, *J* = 6.7, 1.6 Hz, 1H), 1.89 (s, 3H), 1.38 (t, *J* = 7.2 Hz, 3H), 1.35 (s, 3H), 1.28 (t, *J* = 7.1 Hz, 3H) ppm; **^{13}C{^{1}H} NMR** (101 MHz, $CDCl_3$) δ = 167.2, 164.3, 158.7, 148.9, 148.7, 147.8, 135.4, 128.2, 128.1, 126.6, 74.6, 64.5, 61.3, 60.9, 54.7, 15.6, 15.2, 14.4, 14.3 ppm; **IR** (ATR) ṽ = 2978 (w), 2931 (w), 1706 (s), 1619 (w), 1446 (w), 1367 (w), 1300 (w), 1289 (w), 1258 (s), 1229 (m), 1210 (m), 1167 (w), 1125 (w), 1094 (m), 1040 (m), 762 (w), 703 (w) cm^{-1}; **HRMS** (Sicrit plasma/LTQ-Orbitrap) *m/z* = calcd. for $[C_{21}H_{25}O_4]^+$, $[M+H]^+$: 341.1747, found: 341.1738; **R_f** (pentane/EtOAc, 15:1) = 0.25.

4.2 Synthesis of Tetrasubstituted Cp Derivatives

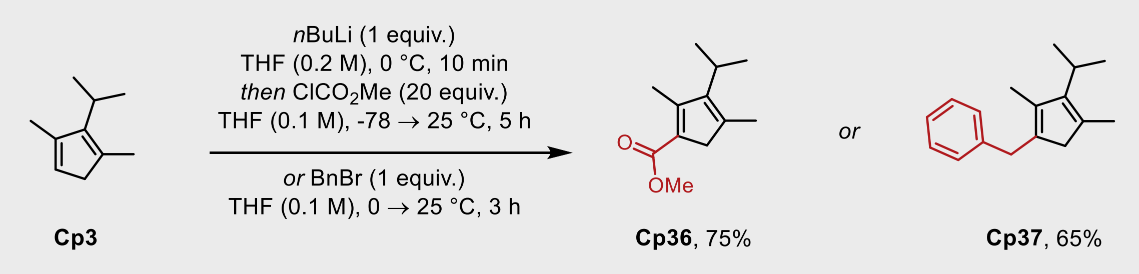

Scheme S17. Acylation or alkylation of 1,2,3-Cps towards tetrasubstituted cyclopentadienes.

Methyl 3-isopropyl-2,4-dimethylcyclopenta-1,3-diene-1-carboxylate (**Cp36**)

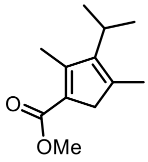

According to the following procedure (**Scheme S17**), tetrasubstituted cyclopentadiene **Cp36** was obtained by acylation of **Cp3**.

At 0 °C (ice bath) and under an atmosphere of nitrogen, *n*-butyllithium solution (1.6 M in hexanes, 0.29 mL, 1.0 equiv.) was added dropwise to a solution of 1,2,3-trisubstituted cyclopentadiene **Cp3** (63 mg, 0.46 mmol, 1.0 equiv.) in anhydrous THF (5 mL/mmol). The reaction mixture was stirred at 0 °C for 10 min. In another microwave vial, at -78 °C (acetone/dry ice bath) and under an atmosphere of nitrogen, the resulting lithium cyclopentadienide solution was added dropwise to a solution of methyl chloroformate (0.72 mL, 20.0 equiv.) in anhydrous THF (5 mL/mmol). The reaction mixture was allowed to slowly warm up in the cold bath to room temperature (25 °C) under stirring for 5 hours. Then, it was quenched with sat. aq. NH_4Cl solution, diluted with water, and extracted with Et_2O (2x). The combined organic layers were washed with brine, dried over $MgSO_4$, filtered, and concentrated under reduced pressure. The residue was purified by flash column chromatography on silica gel (wet loading with pentane, 15 cm column height, gradient: pentane → pentane/Et_2O = 9:1), affording acylated cyclopentadiene **Cp36** (67 mg, 0.34 mmol, 75% yield) as a light-orange liquid. Remaining trisubstituted cyclopentadiene starting material **Cp3** could be recovered as well (24%). Notably, this tetrasubstituted cyclopentadiene was isolated as a single regiomer.

^{1}H NMR (400 MHz, $CDCl_3$) δ = 3.72 (s, 3H), 3.13 (q, *J* = 2.5 Hz, 2H), 2.86 (hept, *J* = 7.2 Hz, 1H), 2.36 (t, *J* = 2.5 Hz, 3H), 2.04 (s, 3H), 1.20 (d, *J* = 7.2 Hz, 6H) ppm; **^{13}C{^{1}H} NMR** (101 MHz, $CDCl_3$) δ = 165.8, 158.2, 146.7, 142.5, 126.1, 50.8, 46.1, 26.4, 21.5, 14.9, 14.5 ppm; **IR** (ATR) ṽ = 2959 (w), 2874 (w), 1700 (s), 1561 (w), 1435 (w), 1378 (w), 1356 (w), 1336 (w), 1244 (m), 1193 (m), 1171 (w), 1143 (w), 1078 (w), 1045 (w), 751 (w) cm^{-1}; **HRMS** (Sicrit plasma/LTQ-Orbitrap) *m/z* = calcd. for $[C_{12}H_{19}O_2]^+$, $[M+H]^+$: 195.1380, found: 195.1378; **R_f** (pentane/Et_2O, 9:1) = 0.47.

((3-Isopropyl-2,4-dimethylcyclopenta-1,3-dien-1-yl)methyl)benzene (**Cp37**)

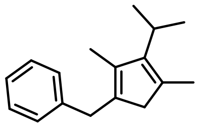

According to the following procedure (**Scheme S17**), tetrasubstituted cyclopentadiene **Cp37** was obtained by benzylation of **Cp3**.

At 0 °C (ice bath) and under an atmosphere of nitrogen, *n*-butyllithium solution (1.6 M in hexanes, 0.29 mL, 1.0 equiv.) was added dropwise to a solution of 1,2,3-trisubstituted cyclopentadiene **Cp3** (63 mg, 0.46 mmol, 1.0 equiv.) in anhydrous THF (5 mL/mmol). The reaction mixture was stirred at 0 °C for 10 min. In another microwave vial, at 0 °C (ice bath) and under an atmosphere of nitrogen, the resulting lithium cyclopentadienide solution was added dropwise to a solution of benzyl bromide (79 mg, 1.0 equiv.) in anhydrous THF (5 mL/mmol). The ice bath was removed, and the reaction mixture was stirred at room temperature (25 °C) for 3 hours. Then, it was quenched with sat. aq. NH_4Cl solution, diluted with water, and extracted with Et_2O (2x). The combined organic layers were washed with brine, dried over $MgSO_4$, filtered, and concentrated under reduced pressure. The residue was purified by flash column chromatography on silica gel (wet loading with pentane, 18 cm column height, isocratic: pentane), affording benzylated cyclopentadiene **Cp37** (68 mg, 0.30 mmol, 65% yield) as a light-orange liquid. Remaining trisubstituted cyclopentadiene starting material **Cp3** could be recovered as well. Notably, this tetrasubstituted cyclopentadiene was isolated as a mixture of three regiomers (in 5:2:1 ratio with **Cp37** as major), but within a few days it completely isomerised to the thermodynamic double bond pattern of **Cp37**.

^{1}H NMR (600 MHz, $CDCl_3$) δ = 7.28 – 7.24 (m, 2H), 7.18 – 7.15 (m, 1H), 7.15 – 7.12 (m, 2H), 3.62 (s, 2H), 2.84 (hept, *J* = 7.2 Hz, 1H), 2.65 (q, *J* = 1.9 Hz, 2H), 1.97 (t, *J* = 1.9 Hz, 3H), 1.90 (s, 3H), 1.20 (d, *J* = 7.2 Hz, 6H) ppm; **^{13}C{^{1}H} NMR** (151 MHz, $CDCl_3$) δ = 145.0, 142.1, 137.1, 136.0, 132.6, 128.7, 128.4, 125.7, 47.3, 34.5, 27.0, 21.8, 14.2, 12.8 ppm; **IR** (ATR) ṽ = 3027 (w), 2959 (s), 2926 (s), 2869 (m), 1603 (w), 1494 (w), 1453 (m), 1382 (w), 1362 (w), 745 (w), 731 (w), 698 (s) cm^{-1}; **HRMS** (Sicrit plasma/LTQ-Orbitrap) *m/z* = calcd. for $[C_{17}H_{23}]^+$, $[M+H]^+$: 227.1794, found: 227.1794; **R_f** (pentane) = 0.39.

5. Synthesis of Cp Metal Complexes

5.1 Cp Cobalt, Iridium, Ruthenium, and Titanium Complexes

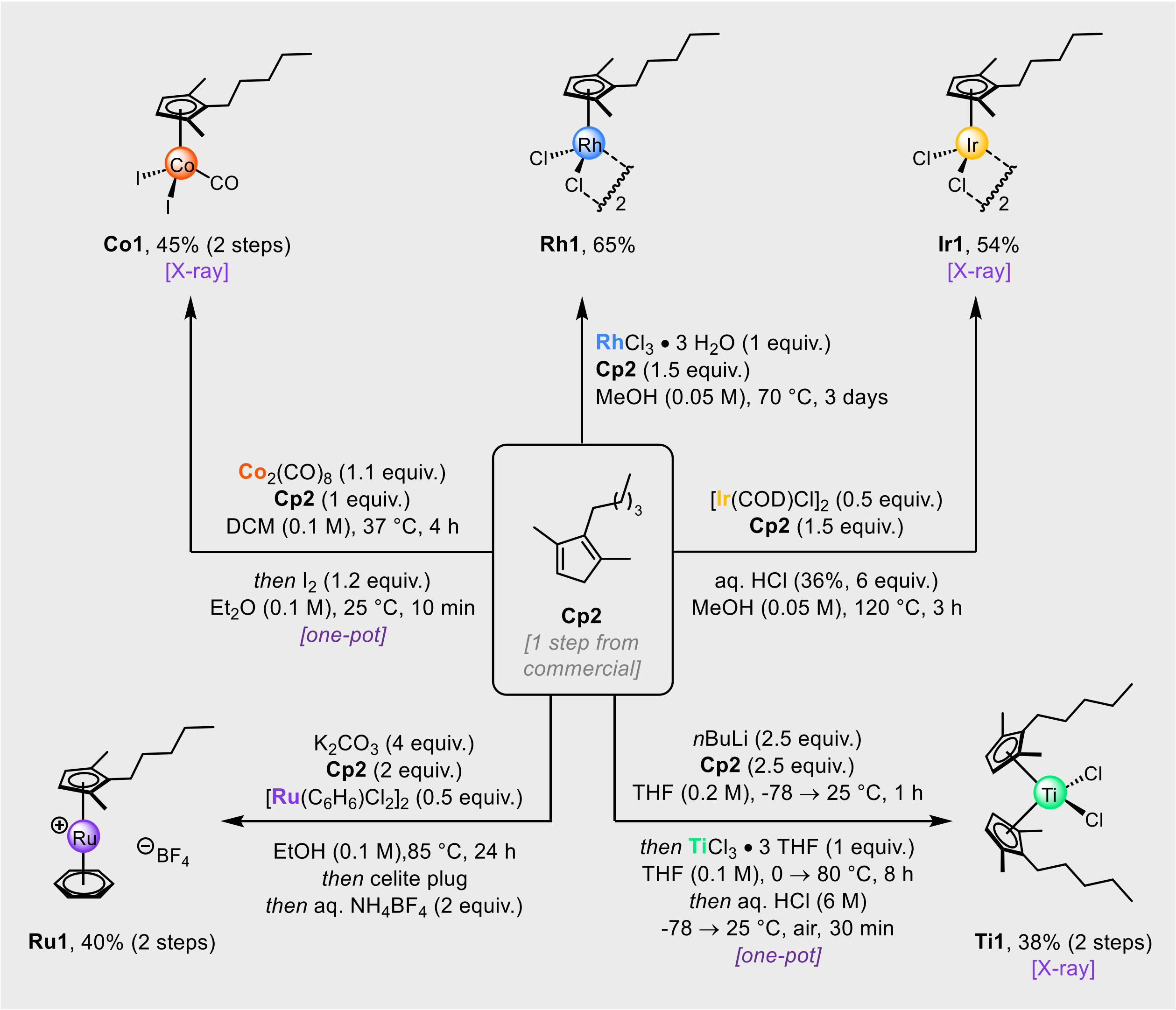

Scheme S18. Complexation ability of 1,2,3-Cps with a selection of early and late transition metals.

<u>Cobalt complex **Co1**</u>

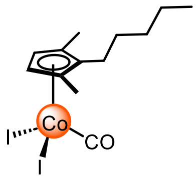

According to the following procedure (**Scheme S18**), Co(III) complex **Co1** was obtained.

Step 1. In a nitrogen-filled glovebox, an oven-dried microwave vial was charged with dicobalt octacarbonyl (248 mg, 1.1 equiv.) and capped. Outside of the glovebox, it was connected to a Schlenk line and a solution of 1,2,3-trisubstituted cyclopentadiene **Cp2** (103 mg, 0.63 mmol, 1.0 equiv.) in anhydrous degassed DCM (10 mL/mmol) was added. The black reaction mixture was stirred in a heating block at 37 °C for 4 hours.

Step 2. After cooling to room temperature (25 °C), a solution of iodine (193 mg, 1.2 equiv.) in anhydrous degassed Et_2O (10 mL/mmol) was added to the reaction mixture, and it was stirred for >10 min. The reaction vessel was kept connected to a Schlenk line considering the instantaneous release of carbon

monoxide gas. After careful purging of the head space with nitrogen, the vial was opened, and all volatiles were removed *in vacuo*. The black residue was purified by flash column chromatography on silica gel (dry loading, 5 cm column height, gradient: pentane/DCM = 100/0 → 50/50), which was performed rapidly to minimize the complex' decomposition on silica. The DCM gradient was only applied as soon as a dark brown band had completely eluted with pentane. The dark violet fractions were collected, and the solvents were removed *in vacuo* to obtain cobalt(III) complex **Co1** (142 mg, 0.28 mmol, 45% yield over 2 steps) as a black solid (dark purple solution in chloroform). A suitable crystal for X-ray analysis (**Figure S4**) was obtained by slow evaporation of a concentrated solution in DCM.

Notably, the CO ligand turned out to be rather labile as – even in the solid state – complex **Co1** slowly dimerized with release of CO to the $[CpCoI_2]_2$-type species **Co2**. However, it could be transformed back to the monomeric $CpCo(CO)I_2$ architecture of **Co1** by exposure to CO gas (10 bar) for 10 min (in DCM).

^{1}H NMR (400 MHz, CDCl3) δ = 5.30 (s, 2H), 2.56 – 2.49 (m, 2H), 2.32 (s, 6H), 1.46 – 1.30 (m, 6H), 0.93 – 0.88 (m, 3H) ppm; **^{13}C{^{1}H} NMR** (101 MHz, CDCl3) δ = 197.9, 110.9, 102.8, 86.7, 32.0, 29.9, 26.6, 22.5, 14.0, 13.2 ppm; **IR** (ATR) ṽ = 2954 (m), 2924 (m), 2856 (w), 2053 (s), 1455 (m), 1376 (w), 1350 (w), 1302 (w), 1105 (w), 1023 (w), 965 (w), 918 (w), 852 (w), 498 (w), 470 (w) cm^{-1}; **HRMS** (ESI/APCI) *m/z* = calcd. for $[C_{12}H_{19}CoI]^+$, $[M\text{-}CO\text{-}I^-]^+$: 348.9858, found: 348.9857; **XRD** (Cu*Kα*, R_1 = 1.83%) CCDC: 2310525.

<u>Cobalt complex **Co2**</u>

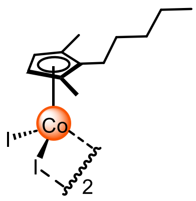

Over time (i.e. within a week in chloroform solution, or within a few months in the solid state) at room temperature (25 °C), monomeric cobalt(III) complex **Co1** slowly released CO and dimerized into $[CpCoI_2]_2$-type complex **Co2** as a black solid (dark green solution in chloroform). The process was easily followed *via* infrared spectroscopy, where the characteristic CO stretching signal (strong band at 2053 cm^{-1}) disappeared.

^{1}H NMR (400 MHz, $CDCl_3$) δ = 4.79 (s, 2H), 2.37 (t, *J* = 7.7 Hz, 2H), 1.94 (s, 6H), 1.29 – 1.10 (m, 6H), 0.83 (t, *J* = 6.7 Hz, 3H) ppm; **^{13}C{^{1}H} NMR** (101 MHz, $CDCl_3$) δ = 92.2, 77.7, 77.4, 32.0, 29.1, 27.0, 22.5, 14.0, 12.8 ppm; **IR** (ATR) ṽ = 3063 (w), 2954 (s), 2924 (s), 2856 (m), 1465 (m), 1455 (m), 1376 (w), 1261 (w), 1099 (w), 1045 (w), 1024 (m), 869 (w), 803 (w) cm^{-1}; **HRMS** (APCI/QTOF) *m/z* = calcd. for $[C_{12}H_{19}CoI]^+$, $[(M/2)\text{-}I^-]^+$: 348.9858, found: 348.9847.

<u>Iridium complex **Ir1**</u>

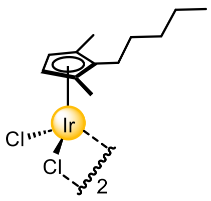

According to the following procedure (**Scheme S18**), Ir(III) complex **Ir1** was obtained.

In a nitrogen-filled glovebox, an oven-dried-dried microwave vial was charged with cyclooctadiene iridium(I) chloride dimer (169 mg, 0.25 mmol, 0.5 equiv.) and capped. Outside of the glovebox, it was connected to a Schlenk line and a solution of 1,2,3-trisubstituted cyclopentadiene **Cp2** (124 mg, 1.5 equiv.) in anhydrous degassed MeOH (20 mL/mmol) was added. Next, aqueous HCl (36%, 1 mL/mmol, *ca.* 6 equiv.) was added dropwise, and the resulting reaction mixture was stirred in a heating block at 120 °C for 3 hours. After cooling to room temperature (25 °C), all volatiles were removed *in vacuo*. The residue was redissolved

in DCM, and filtered through a pad of celite (2 cm) and anhydrous sodium sulfate (2 cm) with DCM as eluent. After evaporation of all volatiles *in vacuo*, the residue was again redissolved in DCM, and filtered through a pad of silica (5 cm) with sequentially DCM (to remove impurities) and EtOAc (to collect the complex) as eluent. After removal of all volatiles *in vacuo*, the residue was redissolved once more in a minimal amount of DCM (0.5 mL). Under stirring, hexane (40 mL) was slowly added, which induced precipitation of a bright orange solid. The suspension was concentrated under reduced pressure until half of the solvent (*ca.* 20 mL) remained and was then filtered on a glass sinter funnel. The orange filter cake was washed with pentane, affording iridium(III) complex **Ir1** (58 mg, 0.14 mmol, 54% yield) as a bright orange powder. A suitable crystal for X-ray analysis (**Figure S4**) was obtained by slow vapour diffusion of pentane into a concentrated solution in chloroform.

^{1}H NMR (400 MHz, $CDCl_3$) δ = 5.46 (s, 2H), 2.21 – 2.14 (m, 2H), 1.74 (s, 6H), 1.47 – 1.38 (m, 2H), 1.36 – 1.26 (m, 4H), 0.88 (t, *J* = 6.8 Hz, 3H) ppm; **^{13}C{^{1}H} NMR** (101 MHz, $CDCl_3$) δ = 93.3, 87.8, 71.4, 31.9, 27.8, 23.8, 22.5, 14.0, 11.5 ppm; **IR** (ATR) ṽ = 3090 (w), 2952 (s), 2920 (s), 2856 (s), 1466 (s), 1452 (s), 1378 (m), 1109 (m), 1038 (w), 892 (w), 734 (m), 432 (w), 419 (w) cm^{-1}; **HRMS** (ESI/QTOF) *m/z* = calcd. for $[C_{12}H_{19}ClIr]^+$, $[(M/2)-Cl^-]^+$: 391.0799, found: 391.0790; **XRD** (Mo*Kα*, R_1 = 3.44%) CCDC: 2418643.

<u>Ruthenium complex **Ru1**</u>

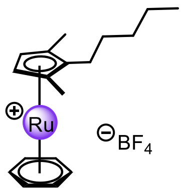

According to the following procedure (**Scheme S18**), Ru(II) complex **Ru1** was obtained.

Step 1. A flame-dried microwave vial was charged with potassium carbonate (325 mg, 4 equiv.) and (benzene)ruthenium(II) dichloride dimer (74 mg, 0.29 mmol, 0.5 equiv.), capped, and placed under an atmosphere of nitrogen by Schlenk technique. A solution of 1,2,3-trisubstituted cyclopentadiene **Cp2** (193 mg, 2.0 equiv.) in degassed ethanol (10 mL/mmol) was added, and the mixture was stirred in a heating block at 85 °C for 24 hours. After cooling to room temperature (25 °C), the reaction mixture was filtered through a pad of celite (3 cm) with ethanol as eluent.

Step 2. All volatiles were removed *in vacuo*, after which the residue was redissolved in ethanol (10 mL/mmol), and a solution of ammonium tetrafluoroborate (123 mg, 2.0 equiv.) in water (5 mL/mmol) was added, resulting in a brown precipitate. The ethanol was removed under reduced pressure, and the resulting aqueous suspension was cooled at 0 °C for 10 min, after which it was filtered on a glass sinter funnel. The filter cake was washed with water, then collected with acetone, and all volatiles were removed *in vacuo*. The residue was redissolved in a minimal amount of acetone (1 mL). Under stirring, Et_2O (50 mL) was slowly added, which induced precipitation of gray solids. After filtration on a glass sinter funnel, the filter cake was washed with Et_2O, affording the first crop of **Ru1** (78 mg). When concentrating the filtrate and repeating above procedure, a second pure crop (22 mg) could be obtained and was combined, ultimately affording 100 mg of ruthenium(II) complex **Ru1** (0.23 mmol, 40% yield over 2 steps) as a gray solid.

^{1}H NMR (400 MHz, $CDCl_3$) δ = 5.99 (s, 6H), 5.28 (s, 2H), 2.43 – 2.37 (m, 2H), 2.03 (s, 6H), 1.43 – 1.34 (m, 2H), 1.34 – 1.25 (m, 4H), 0.87 (t, *J* = 6.7 Hz, 3H) ppm; **^{13}C{^{1}H} NMR** (101 MHz, $CDCl_3$) δ = 102.7, 98.7, 86.8,

80.2, 31.6, 30.1, 25.5, 22.5, 14.0, 12.6 ppm; **^{19}F{^{1}H} NMR** (376 MHz, $CDCl_3$) δ = -152.34 ppm; **IR** (ATR) ṽ = 3091 (w), 2956 (w), 2927 (w), 2860 (w), 1469 (w), 1442 (w), 1384 (w), 1055 (s), 977 (w), 915 (w), 828 (w), 729 (w), 520 (w), 422 (w) cm^{-1}; **HRMS** (ESI/QTOF) *m/z* = calcd. for $[C_{18}H_{25}Ru]^+$, $[M-BF_4^-]^+$: 343.0994, found: 343.1001.

Titanium complex **Ti1**

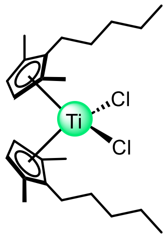

According to the following procedure (**Scheme S18**), Ti(IV) complex **Ti1** was obtained.

Step 1. At -78 °C (acetone/dry ice bath) and under an atmosphere of nitrogen, *n*-butyllithium solution (1.6 M in hexanes, 0.61 mL, 2.5 equiv.) was added dropwise to a solution of 1,2,3-trisubstituted cyclopentadiene **Cp2** (161 mg, 2.5 equiv.) in anhydrous THF (5 mL/mmol). The cold bath was removed, and the reaction mixture was stirred at room temperature (25 °C) for 1 hour. In a nitrogen-filled glovebox, a second oven-dried microwave vial was charged with titanium(III) chloride tetrahydrofuran complex (146 mg, 0.39 mmol, 1.0 equiv.) and capped. Outside of the glovebox, it was connected to a Schlenk line, and the bright blue solid was suspended in anhydrous THF (5 mL/mmol). At 0 °C (ice bath), the lithium cyclopentadienide solution was added dropwise to the titanium precursor suspension, and the resulting brown reaction mixture was stirred in a heating block at 80 °C for 8 hours.

Step 2. At -78 °C (acetone/dry ice bath), aqueous HCl (6 M, 10 mL/mmol) was added dropwise to the mixture under vigorous stirring, resulting in a colour change to dark red. The cold bath was removed, the vial was opened, and the reaction mixture was allowed to stir at room temperature (25 °C) under air for 30 min. The resulting suspension of bright orange-red solids was diluted with water and extracted with DCM (3x). The combined organic layers were washed with brine, dried over $MgSO_4$, filtered over a pad of celite (3 cm), and concentrated under reduced pressure. The residue was redissolved in a minimal amount of DCM (2 mL), after which hexane (60 mL) was added. The red solution was concentrated under reduced pressure until half of the solvent (*ca.* 30 mL) remained and was then filtered on a glass sinter funnel. The collected red needles were washed with pentane, affording the first crop of **Ti1** (47 mg). When concentrating the filtrate and repeating above procedure, a second pure crop (19 mg) could be obtained and was combined, ultimately affording 66 mg of titanium(IV) complex **Ti1** (0.15 mmol, 38% yield over 2 steps) as red needle-shaped crystals. A suitable crystal for X-ray analysis (**Figure S4**) was immediately obtained from the first crop.

^{1}H NMR (400 MHz, $CDCl_3$) δ = 5.90 (s, 4H), 2.58 – 2.49 (m, 4H), 2.02 (s, 12H), 1.46 – 1.38 (m, 4H), 1.38 – 1.28 (m, 8H), 0.91 – 0.85 (m, 6H) ppm; **^{13}C{^{1}H} NMR** (101 MHz, $CDCl_3$) δ = 136.3, 134.2, 111.8, 32.2, 28.8, 27.2, 22.7, 15.7, 14.1 ppm; **IR** (ATR) ṽ = 2960 (w), 2949 (w), 2916 (m), 2856 (w), 1496 (w), 1466 (w), 1378 (w), 1041 (w), 1029 (w), 906 (s), 855 (w), 835 (w), 730 (s), 650 (w) cm^{-1}; **HRMS** (APCI/QTOF) *m/z* = calcd. for $[C_{24}H_{38}ClTi]^+$, $[M-Cl^-]^+$: 409.2136, found: 409.2130; **XRD** (Mo*Kα*, R_1 = 3.37%) CCDC: 2418644.

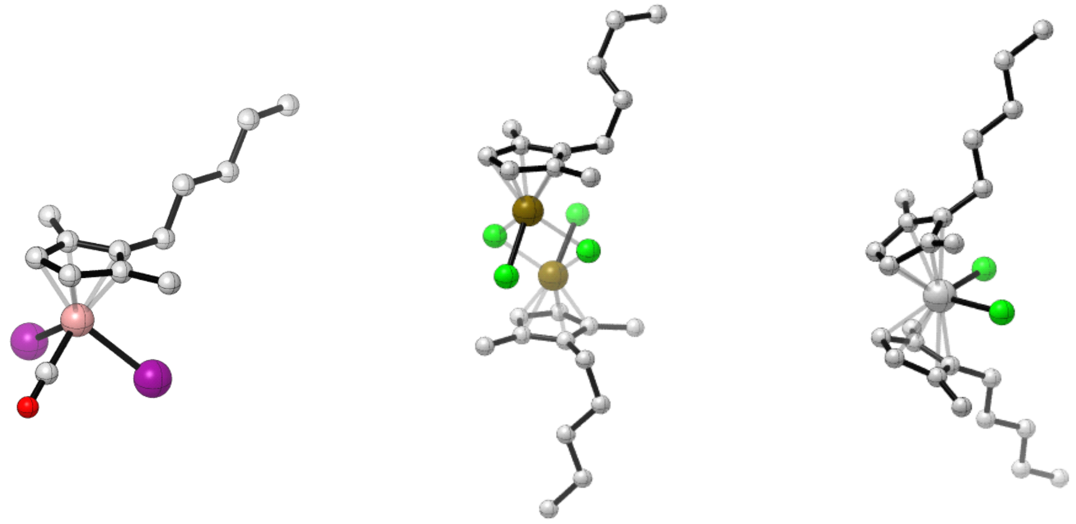

Figure S4. Solid-state X-ray structure of complexes **Co1** (*left*, CCDC: 2310525), **Ir1** (*middle*, CCDC: 2418643), and **Ti1** (*right*, CCDC: 2418644) showing 50% probability thermal ellipsoids. Hydrogen atoms, solvent molecules, and disorder are omitted for clarity.

5.2 Cp Rhodium Complexes

***General Procedure 7* – Rhodium(III) Complexation of Cyclopentadienes.**

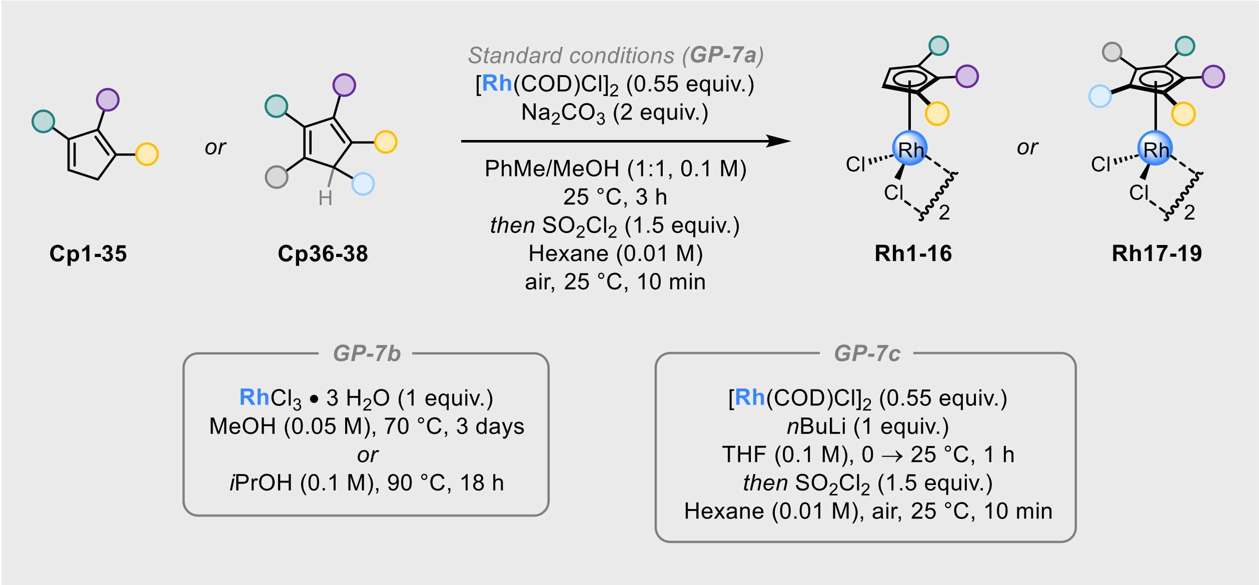

Scheme S19. Rhodium(III) complexation of cyclopentadienes towards $[CpRhCl_2]_2$-type dimers.

***General Procedure 7a*: With $[Rh(COD)Cl]_2$ and Na_2CO_3 –** An oven-dried microwave vial was charged with sodium carbonate (2.0 equiv.) and cyclooctadiene rhodium(I) chloride dimer (0.55 equiv.), capped, and placed under an atmosphere of nitrogen by Schlenk technique. The solids were suspended in anhydrous methanol (5 mL/mmol), after which a solution of the desired 1,2,3-trisubstituted cyclopentadiene (1.0 equiv.) in toluene (5 mL/mmol) was added. The reaction mixture was stirred at room temperature (25 °C) for 3 hours, during which consumption of the suspended yellow rhodium precursor could be observed. Next, all volatiles were removed *in vacuo*. The residue was redissolved in toluene and passed

through a pad of neutral aluminum oxide (*or* silica, 3 cm) with toluene as eluent to give a homogeneous light-yellow solution. After removal of all volatiles *in vacuo*, the residue was redissolved in hexane (*or* Et_2O, 50 mL/mmol). Under vigorous stirring, a solution of sulfuryl chloride (1.5 equiv.) in hexane (50 mL/mmol) was added at room temperature (25 °C) under air, resulting in an immediate colour change and the precipitation of a fine orange-red powder. After filtration on a glass sinter funnel, the filter cake was washed with pentane, affording the corresponding dimeric $[CpRhCl_2]_2$-type rhodium(III) complex.

***General Procedure 7b*: With $RhCl_3$·3 H_2O –** An oven-dried microwave vial was charged with rhodium trichloride trihydrate (1.0 equiv.), capped, and placed under an atmosphere of nitrogen by Schlenk technique. A solution of the desired 1,2,3-trisubstituted cyclopentadiene (1.0 equiv.) in methanol (20 mL/mmol) was added, and the reaction mixture was stirred in a heating block at 70 °C for 3 days. Alternatively, isopropanol (10 mL/mmol) was used, and the reaction mixture was stirred in a heating block at 90 °C for 18 hours. After cooling to room temperature (25 °C), all volatiles were removed *in vacuo*. The residue was redissolved in DCM, and filtered through a pad of celite (3 cm) with DCM as eluent. After evaporation of all volatiles *in vacuo*, the residue was again redissolved in DCM, and filtered through a pad of silica (5 cm) with sequentially DCM (to remove impurities) and EtOAc (to collect the complex) as eluent. Removal of all volatiles *in vacuo* afforded the corresponding rhodium(III) complex.

***General Procedure 7c*: With $[Rh(COD)Cl]_2$ and nBuLi –** At 0 °C (ice bath) and under an atmosphere of nitrogen, *n*-butyllithium solution (1.6 M in hexanes, 1.0 equiv.) was added dropwise to a solution of the desired 1,2,3-trisubstituted cyclopentadiene (1.0 equiv.) in anhydrous THF (5 mL/mmol). The reaction mixture was stirred at 0 °C for 10 min. In another microwave vial, at 0 °C (ice bath) and under an atmosphere of nitrogen, the resulting lithium cyclopentadienide solution was added dropwise to a suspension of cyclooctadiene rhodium(I) chloride dimer (0.55 equiv.) in anhydrous THF (5 mL/mmol). The ice bath was removed, and the reaction mixture was stirred at room temperature (25 °C) for 1 hour. After removal of all volatiles *in vacuo*, the rest of the protocol is identical to *General Procedure 7a* (silica filtration with toluene, evaporation, oxidation with sulfuryl chloride in hexane, filtration).

Note: the preferred solvent for the NMR characterization of most **Rh** complexes was d_6-DMSO, since it breaks up the dimeric $[CpRhCl_2]_2$ structures forming monomeric adducts. This usually resulted in more resolved spectra with sharper signals compared to $CDCl_3$. Additionally, it simplified the spectra in the case of planar chirality with the racemic complexes existing as both homo- and heterochiral dimers.

Rhodium complex **Rh1**

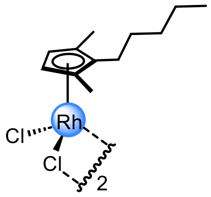

According to *General Procedure 7b*, starting from cyclopentadiene **Cp2** (0.71 mmol, 1.5 equiv.) and $RhCl_3$·3 H_2O (0.47 mmol) in methanol at 70 °C, Rh(III) complex **Rh1** (103 mg, 0.31 mmol of monomer, 65% yield) was obtained as a red solid. When *General Procedure 7c* was followed, a similar yield of **Rh1** (96 mg, 0.28 mmol of monomer, 60% yield over 2 steps) was obtained.

^{1}H NMR (400 MHz, $CDCl_3$) δ = 5.23 (s, 2H), 2.28 (t, *J* = 7.8 Hz, 2H), 1.76 (s, 6H), 1.43 – 1.35 (m, 2H), 1.34 – 1.26 (m, 4H), 0.87 (t, *J* = 6.7 Hz, 3H) ppm; **^{13}C{^{1}H} NMR** (101 MHz, $CDCl_3$) δ = 100.6 (d, *J* = 8.5 Hz), 97.7

(d, J = 8.7 Hz), 79.3 (d, J = 9.4 Hz), 31.8, 27.5, 23.8, 22.5, 14.0, 11.6 ppm; **IR** (ATR) ṽ = 3086 (w), 3078 (m), 2942 (m), 2915 (s), 2854 (m), 1479 (w), 1460 (s), 1450 (s), 1377 (m), 1265 (w), 1109 (w), 1040 (m), 1027 (w), 964 (w), 888 (w), 733 (s), 700 (w), 590 (w), 429 (w) cm^{-1}; **HRMS** (ESI/QTOF) *m/z* = calcd. for $[C_{12}H_{19}ClRh]^+$, $[(M/2)-Cl^-]^+$: 301.0225, found: 301.0233.

Rhodium complex **Rh2**

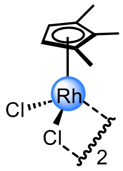

According to *General Procedure 7a*, starting from cyclopentadiene **Cp1** (0.63 mmol), Rh(III) complex **Rh2** (144 mg, 0.51 mmol of monomer, 81% yield over 2 steps) was obtained as an orange-red solid.

^{1}H NMR (400 MHz, d_6-DMSO) δ = 5.59 (s, 2H), 1.71 (s, 6H), 1.61 (s, 3H) ppm; **^{13}C{^{1}H} NMR** (101 MHz, d_6-DMSO) δ = 105.4 (d, J = 6.9 Hz), 101.1 (d, J = 6.0 Hz), 82.5 (d, J = 8.4 Hz), 10.7, 8.4 ppm; **IR** (ATR) ṽ = 3087 (m), 3058 (m), 2964 (w), 2915 (w), 1478 (s), 1454 (s), 1377 (w), 1302 (w), 1201 (w), 1044 (m), 1027 (m), 896 (w), 737 (w), 696 (w), 686 (w), 443 (w), 427 (w) cm^{-1}; **HRMS** (ESI/QTOF) *m/z* = calcd. for $[C_8H_{11}ClRh]^+$, $[(M/2)-Cl^-]^+$: 244.9599, found: 244.9600.

Rhodium complex **Rh3**

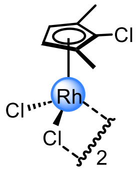

According to *General Procedure 7a*, starting from cyclopentadiene **Cp22** (0.26 mmol), chloro-bearing Rh(III) complex **Rh3** (54 mg, 0.18 mmol of monomer, 69% yield over 2 steps) was obtained as an orange-red solid. A suitable crystal for X-ray analysis (**Figure S5**) was obtained by slow vapour diffusion of hexane into a concentrated solution in chloroform.

^{1}H NMR (400 MHz, d_6-DMSO) δ = 5.79 (s, 2H), 1.82 (s, 6H) ppm; **^{13}C{^{1}H} NMR** (101 MHz, d_6-DMSO) δ = 105.8 (d, J = 6.4 Hz), 103.8 (d, J = 6.1 Hz), 81.4 (d, J = 8.1 Hz), 10.5 ppm; **IR** (ATR) ṽ = 3074 (w), 2980 (w), 2923 (w), 1714 (m), 1667 (w), 1468 (m), 1439 (s), 1373 (w), 1350 (w), 1331 (w), 1282 (w), 1105 (w), 1088 (w), 1073 (w), 1029 (m), 999 (w), 911 (w), 730 (s), 596 (w) cm^{-1}; **HRMS** (ESI/QTOF) *m/z* = calcd. for $[C_7H_8Cl_2Rh]^+$, $[(M/2)-Cl^-]^+$: 264.9053, found: 264.9060; **XRD** (Cu*Kα*, R_1 = 3.54%) CCDC: 2479662.

Rhodium complex (±)-**Rh4**

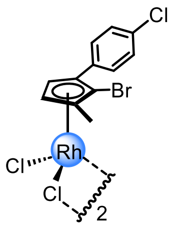

According to *General Procedure 7a*, starting from cyclopentadiene **Cp27** (0.25 mmol), racemic bromo-substituted Rh(III) complex **Rh4** (35 mg, 0.08 mmol of monomer, 31% yield over 2 steps) was obtained as an orange-red solid. A suitable crystal for X-ray analysis (**Figure S5**) was obtained by slow vapour diffusion of hexane into a concentrated solution in chloroform, hereby confirming the molecular structure of **Rh4**; however, the collected X-ray diffraction data were of rather low quality.

^{1}H NMR (400 MHz, d_6-DMSO) δ = 7.92 – 7.87 (m, 2H), 7.59 – 7.54 (m, 2H), 6.44 (d, J = 2.8 Hz, 1H), 6.08 (d, J = 2.8 Hz, 1H), 1.88 (s, 3H) ppm; **^{13}C{^{1}H} NMR** (101 MHz, d_6-DMSO) δ = 135.0, 131.8, 128.5, 127.0, 108.3 (d, J = 6.2 Hz), 100.3 (d, J = 6.6 Hz), 94.8 (d, J = 5.5 Hz), 86.7 (d, J = 7.9 Hz), 81.5 (d, J = 8.1 Hz), 12.1 ppm; **IR** (ATR) ṽ = 3067 (w), 1593 (w), 1434 (s), 1407 (w), 1377 (w), 1294 (w), 1099 (w), 1088 (w), 1013 (w), 892 (w), 834 (m), 760 (m), 745 (m), 717 (w), 532 (w), 453 (w) cm^{-1}; **HRMS** (ESI/QTOF) *m/z* = calcd. for $[C_{12}H_9BrCl_2Rh]^+$, $[(M/2)-Cl^-]^+$: 404.8314, found: 404.8322; **XRD** (Cu*Kα*, R_1 = 22.66%) CCDC: 2479660.

Rhodium complex **Rh5**

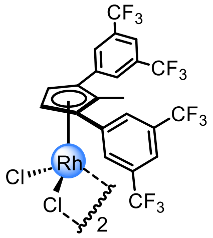

According to *General Procedure 7a*, starting from cyclopentadiene **Cp29** (0.12 mmol), Rh(III) complex **Rh5** (61 mg, 0.09 mmol of monomer, 76% yield over 2 steps) was obtained as an orange-red solid. Notably, the compound did not precipitate during the oxidation step in hexane. Purification was performed by flash column chromatography on silica gel (wet loading with DCM, 5 cm column height, isocratic: DCM *then* EtOAc). A suitable crystal for X-ray analysis (**Figure S5**) was obtained by slow vapour diffusion of hexane into a concentrated solution in chloroform.

^{1}H NMR (400 MHz, $CDCl_3$) δ = 8.20 (s, 4H), 8.01 (s, 2H), 6.15 (s, 2H), 1.99 (s, 3H) ppm; **^{13}C{^{1}H} NMR** (101 MHz, $CDCl_3$) δ = 133.0 (q, *J* = 34.1 Hz), 130.6, 129.9 (q, *J* = 4.0 Hz), 124.4 – 124.1 (m), 122.8 (q, *J* = 273.2 Hz), 103.0 (d, *J* = 7.1 Hz), 93.0 (d, *J* = 8.9 Hz), 85.9 (d, *J* = 8.1 Hz), 11.1 ppm; **^{19}F{^{1}H} NMR** (376 MHz, $CDCl_3$) δ = -62.96 ppm; **IR** (ATR) ṽ = 1360 (m), 1325 (w), 1278 (s), 1177 (m), 1129 (s), 1031 (w), 903 (m), 847 (w), 734 (w), 704 (w), 679 (w) cm^{-1}; **HRMS** (ESI/QTOF) *m/z* = calcd. for $[C_{24}H_{14}ClF_{12}NRh]^+$, $[(M/2)\text{-}Cl^-\text{+}MeCN]^+$: 681.9673, found: 681.9661; **XRD** (Mo*Kα*, R_1 = 3.15%) CCDC: 2479664.

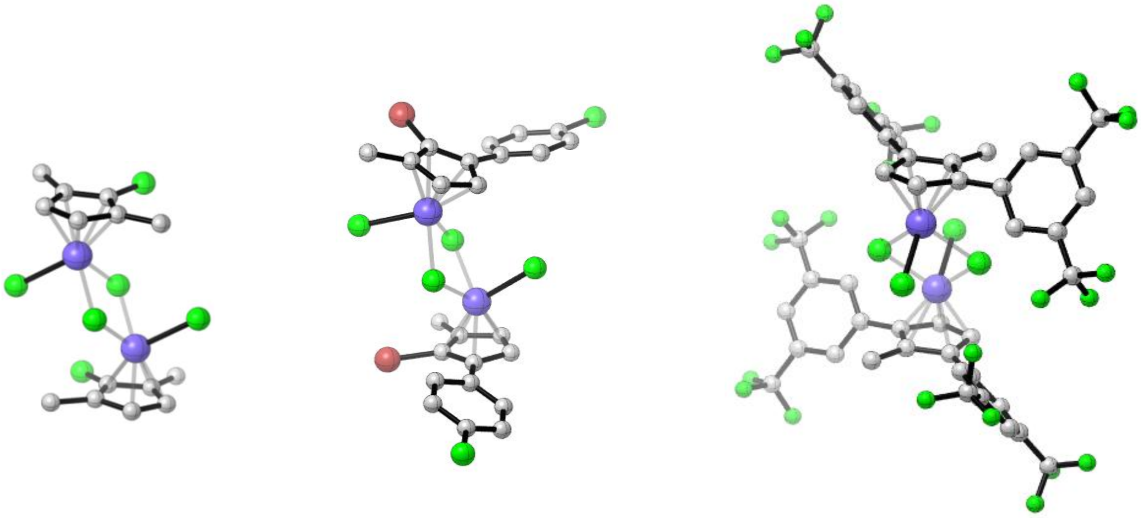

Figure S5. Solid-state X-ray structure of complexes **Rh3** (*left*, CCDC: 2479662), **Rh4** (*middle*, CCDC: 2479660), and **Rh5** (*right*, CCDC: 2479664) showing 50% probability thermal ellipsoids. Hydrogen atoms, solvent molecules, and disorder are omitted for clarity.

Rhodium complex **Rh6**

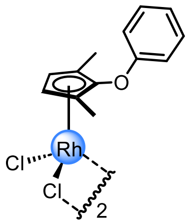

According to *General Procedure 7a*, starting from cyclopentadiene **Cp24** (0.16 mmol), phenoxy-substituted Rh(III) complex **Rh6** (45 mg, 0.13 mmol of monomer, 79% yield over 2 steps) was obtained as an orange solid. A suitable crystal for X-ray analysis (**Figure S6**) was obtained by slow vapour diffusion of hexane into a concentrated solution in chloroform.

^{1}H NMR (500 MHz, d_6-DMSO) δ = 7.48 – 7.41 (m, 4H), 7.30 – 7.26 (m, 1H), 5.66 (s, 2H), 1.45 (s, 6H) ppm; **^{13}C{^{1}H} NMR** (126 MHz, d_6-DMSO) δ = 153.8, 133.2 (d, *J* = 4.6 Hz), 129.9, 125.8, 119.8, 88.6 (d, *J* = 6.8

Hz), 81.6 (d, J = 8.7 Hz), 9.8 ppm; **IR** (ATR) ṽ = 3086 (w), 1458 (s), 1432 (w), 1411 (m), 1384 (w), 1208 (s), 1195 (m), 1044 (w), 1023 (w), 845 (w), 785 (w), 695 (w), 631 (w), 490 (w) cm^{-1}; **HRMS** (ESI/QTOF) m/z = calcd. for $[C_{13}H_{13}ClORh]^+$, $[(M/2)\text{-}Cl^-]^+$: 322.9704, found: 322.9695; **XRD** (Cu*Kα*, R_1 = 3.39%) CCDC: 2479665.

Rhodium complex **Rh7**

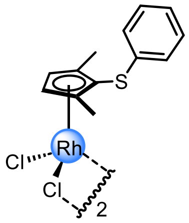

According to *General Procedure 7a*, starting from cyclopentadiene **Cp25** (0.55 mmol), sulfide-substituted Rh(III) complex **Rh7** (157 mg, 0.42 mmol of monomer, 76% yield over 2 steps) was obtained as an orange-red solid. A suitable crystal for X-ray analysis (**Figure S6**) was obtained by slow vapour diffusion of pentane into a concentrated solution in chloroform.

^{1}H NMR (400 MHz, d_6-DMSO) δ = 7.37 – 7.25 (m, 5H), 5.88 (s, 2H), 1.75 (s, 6H) ppm; **^{13}C{^{1}H} NMR** (101 MHz, d_6-DMSO) δ = 132.7, 129.6, 128.3, 127.1, 113.7 (d, J = 6.5 Hz), 91.8 (d, J = 6.4 Hz), 83.5 (d, J = 8.0 Hz), 11.7 ppm; **IR** (ATR) ṽ = 3065 (m), 1584 (w), 1478 (m), 1460 (w), 1439 (m), 1372 (w), 1361 (w), 1032 (w), 1022 (m), 1005 (w), 852 (w), 732 (s), 688 (m), 486 (w), 465 (w) cm^{-1}; **HRMS** (ESI/QTOF) m/z = calcd. for $[C_{13}H_{13}ClRhS]^+$, $[(M/2)\text{-}Cl^-]^+$: 338.9476, found: 338.9483; **XRD** (Cu*Kα*, R_1 = 2.25%) CCDC: 2479663.

Rhodium complex **Rh8**

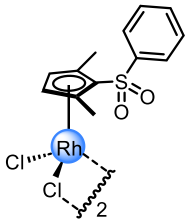

According to *General Procedure 7a*, starting from cyclopentadiene **Cp26** (0.21 mmol), sulfone-substituted Rh(III) complex **Rh8** (34 mg, 0.08 mmol of monomer, 40% yield over 2 steps) was obtained as an orange-red solid. After filtration, purification was performed by flash column chromatography on silica gel (wet loading with DCM, 5 cm column height, isocratic: DCM *then* EtOAc). Notably, in d_6-DMSO solution, the complex is rather unstable (i.e. full decomposition within 48 h).

^{1}H NMR (500 MHz, d_6-DMSO) δ = 8.03 – 7.99 (m, 2H), 7.81 – 7.77 (m, 1H), 7.69 – 7.64 (m, 2H), 5.83 (s, 2H), 2.00 (s, 6H) ppm; **^{13}C{^{1}H} NMR** (126 MHz, d_6-DMSO) δ = 140.3, 134.7, 129.8, 127.6, 115.2 (d, J = 5.8 Hz), 91.1 (d, J = 7.1 Hz), 85.5 (d, J = 7.5 Hz), 12.6 ppm; **IR** (ATR) ṽ = 2929 (w), 1691 (w), 1446 (m), 1304 (s), 1147 (s), 1090 (m), 1037 (w), 1023 (w), 999 (w), 911 (w), 758 (w), 726 (s), 689 (m), 639 (w), 623 (m), 593 (w), 553 (m) cm^{-1}; **HRMS** (ESI/QTOF) m/z = calcd. for $[C_{13}H_{13}ClO_2RhS]^+$, $[(M/2)\text{-}Cl^-]^+$: 370.9374, found: 370.9382.

Rhodium complex (±)-**Rh9**

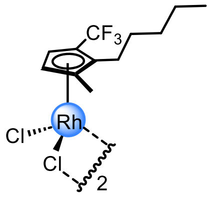

According to *General Procedure 7a*, starting from cyclopentadiene **Cp35** (0.34 mmol) and with KOAc (2 equiv.) at 70 °C for 1 h instead of Na_2CO_3 at 25 °C for 3 h, racemic trifluoromethyl-substituted Rh(III) complex **Rh9** (75 mg, 0.19 mmol of monomer, 56% yield over 2 steps) was obtained as an orange-red solid. In the ^{13}C NMR spectrum, the CF_3-bearing carbon appears as a resolved quartet of doublets due to coupling with both rhodium and fluorine. A suitable crystal for X-ray analysis (**Figure S6**) was obtained by slow vapour diffusion of hexane into a concentrated solution in chloroform. Notably, when *General Procedure 7b* is

followed in methanol at 70 °C, a mixture of three rhodium species is isolated, containing the desired complex **Rh9** as well as both the *rac*- and *meso*-rhodocenium chlorides (in 3:2:1 or 3:1:2 ratio).

^{1}H NMR (600 MHz, d_6-DMSO) δ = 6.54 (d, *J* = 2.8 Hz, 1H), 6.05 (d, *J* = 2.8 Hz, 1H), 2.40 – 2.33 (m, 1H), 2.31 – 2.24 (m, 1H), 1.79 (s, 3H), 1.57 – 1.42 (m, 2H), 1.39 – 1.25 (m, 4H), 0.87 (t, *J* = 7.1 Hz, 3H) ppm; **^{13}C{^{1}H} NMR** (151 MHz, d_6-DMSO) δ = 123.1 (q, *J* = 272.3 Hz), 111.1 (d, *J* = 4.9 Hz), 105.2 (d, *J* = 6.0 Hz), 90.0 – 89.9 (m), 85.3 (d, *J* = 7.3 Hz), 83.4 (qd, *J* = 38.7, 8.3 Hz), 31.1, 26.9, 23.8, 21.7, 13.6, 10.0 ppm; **^{19}F{^{1}H} NMR** (376 MHz, d_6-DMSO) δ = -55.25 ppm; **IR** (ATR) ṽ = 3056 (w), 2956 (w), 2929 (w), 2862 (w), 1459 (m), 1326 (m), 1229 (w), 1208 (w), 1182 (m), 1150 (s), 1138 (s), 1083 (m), 1043 (w), 892 (w) cm^{-1}; **HRMS** (nanochip-ESI/LTQ-Orbitrap) *m/z* = calcd. for $[C_{12}H_{16}ClF_3Rh]^+$, $[(M/2)\text{-}Cl^-]^+$: 354.9942, found: 354.9937; **XRD** (Cu*Kα*, R_1 = 5.25%) CCDC: 2479659.

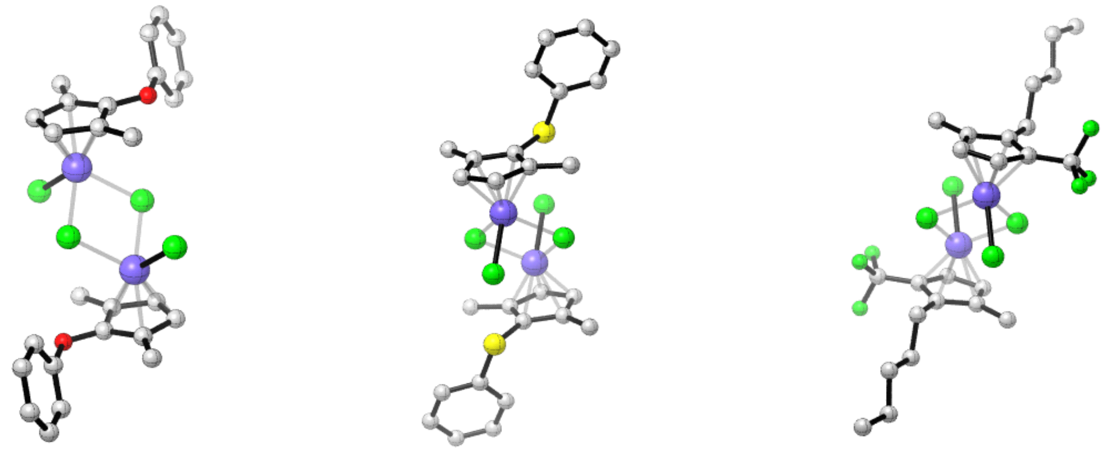

Figure S6. Solid-state X-ray structure of complexes **Rh6** (*left*, CCDC: 2479665), **Rh7** (*middle*, CCDC: 2479663), and **Rh9** (*right*, CCDC: 2479659) showing 50% probability thermal ellipsoids. Hydrogen atoms, solvent molecules, and disorder are omitted for clarity.

Rhodium complex **Rh10**

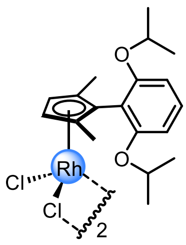

According to *General Procedure 7b*, starting from cyclopentadiene **Cp19** (0.19 mmol, 1.2 equiv.) and $RhCl_3$·3 H_2O (0.15 mmol) in isopropanol at 90 °C, Rh(III) complex **Rh10** (51 mg, 0.11 mmol of monomer, 72% yield) was obtained as an orange-red solid. Notably, when *General Procedure 7a* is followed instead, partial *ortho*-chlorination of the electron-rich arene ring occurs during the SO_2Cl_2-mediated oxidation step, affording a mixture of rhodium complexes.

^{1}H NMR (400 MHz, d_6-DMSO) δ = 7.3 (t, *J* = 8.4 Hz, 1H), 6.7 (d, *J* = 8.5 Hz, 1H), 6.6 (d, *J* = 8.1 Hz, 1H), 5.6 (s, 2H), 4.7 (hept, *J* = 6.0 Hz, 1H), 4.5 (hept, *J* = 6.0, 5.5 Hz, 1H), 1.6 (s, 6H), 1.4 (d, *J* = 6.0 Hz, 6H), 1.1 (d, *J* = 6.0 Hz, 6H) ppm; **^{13}C{^{1}H} NMR** (101 MHz, d_6-DMSO) δ = 156.8, 155.6, 130.9, 107.9 (d, *J* = 6.3 Hz), 106.1, 105.4, 104.9, 102.3 (d, *J* = 5.4 Hz), 81.0 (d, *J* = 8.9 Hz), 70.8, 69.2, 21.7, 21.6, 12.5 ppm; **IR** (ATR) ṽ = 2975 (w), 2927 (w), 1590 (m), 1468 (w), 1452 (m), 1385 (w), 1374 (w), 1286 (w), 1252 (m), 1112 (s), 1068 (m), 992 (w), 943 (w), 902 (w), 780 (w), 734 (w) cm^{-1}; **HRMS** (ESI/QTOF) *m/z* = calcd. for $[C_{19}H_{25}ClO_2Rh]^+$, $[(M/2)\text{-}Cl^-]^+$: 423.0593, found: 423.0592.

<u>Rhodium complex **Rh11**</u>

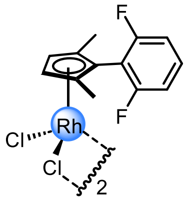

According to *General Procedure 7a*, starting from cyclopentadiene **Cp17** (0.21 mmol), Rh(III) complex **Rh11** (37 mg, 0.10 mmol of monomer, 45% yield over 2 steps) was obtained as an orange-red solid.

^{1}H NMR (600 MHz, d_6-DMSO) δ = 7.66 – 7.60 (m, 1H), 7.24 (t, *J* = 8.8 Hz, 2H), 5.79 (s, 2H), 1.68 (s, 6H) ppm; **^{13}C{^{1}H} NMR** (151 MHz, d_6-DMSO) δ = 160.1 (dd, *J* = 248.2, 5.8 Hz), 158.8 (dd, *J* = 258.3, 6.4 Hz), 132.4 (t, *J* = 10.3 Hz), 112.9 (dd, *J* = 21.5, 2.3 Hz), 111.6 (dd, *J* = 22.1, 2.9 Hz), 110.0 (d, *J* = 6.0 Hz), 105.5 (t, *J* = 18.6 Hz), 91.4 (d, *J* = 5.6 Hz), 82.7 (d, *J* = 8.2 Hz), 11.8 ppm; **^{19}F{^{1}H} NMR** (565 MHz, d_6-DMSO) δ = -95.38, -110.10 ppm; **IR** (ATR) ṽ = 3082 (w), 1625 (m), 1472 (w), 1455 (m), 1280 (w), 1239 (w), 1043 (w), 1019 (w), 997 (m), 917 (m), 896 (w), 792 (s), 738 (s), 724 (m), 670 (w), 643 (w), 547 (w) cm^{-1}; **HRMS** (ESI/QTOF) *m/z* = calcd. for $[C_{13}H_{11}ClF_2Rh]^+$, $[(M/2)\text{-}Cl^-]^+$: 342.9567, found: 342.9568.

<u>Rhodium complex **Rh12**</u>

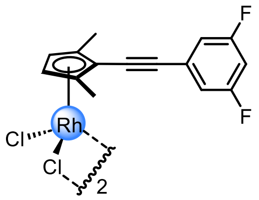

According to *General Procedure 7a*, starting from cyclopentadiene **Cp21** (0.21 mmol), alkynyl-substituted Rh(III) complex **Rh12** (74 mg, 0.18 mmol of monomer, 86% yield over 2 steps) was obtained as a red solid. Notably, its poor solubility in $CHCl_3$, unlike for **Rh1-11**, suggests a polymeric nature similar as for $[CpRhI_2]_n$.[32]

^{1}H NMR (400 MHz, d_6-DMSO) δ = 7.48 (tt, *J* = 9.5, 2.4 Hz, 1H), 7.44 – 7.39 (m, 2H), 5.81 (s, 2H), 1.92 (s, 6H) ppm; **^{13}C{^{1}H} NMR** (101 MHz, d_6-DMSO) δ = 162.4 (dd, *J* = 247.6, 13.8 Hz), 124.0 (t, *J* = 12.1 Hz), 115.1 (dd, *J* = 19.5, 7.9 Hz), 112.3 (d, *J* = 6.5 Hz), 106.2 (t, *J* = 25.9 Hz), 94.4 (t, *J* = 4.0 Hz), 83.0 (d, *J* = 7.9 Hz), 81.9 (d, *J* = 6.0 Hz), 81.5, 11.8 ppm; **^{19}F{^{1}H} NMR** (376 MHz, d_6-DMSO) δ = -108.58 ppm; **IR** (ATR) ṽ = 3058 (w), 1614 (s), 1588 (s), 1458 (w), 1427 (m), 1329 (w), 1190 (w), 1122 (s), 1036 (w), 989 (m), 961 (w), 866 (m), 846 (m), 672 (w) cm^{-1}; **HRMS** (ESI/QTOF) *m/z* = calcd. for $[C_{15}H_{11}ClF_2Rh]^+$, $[(M/2)\text{-}Cl^-]^+$: 366.9567, found: 366.9574.

<u>Rhodium complex (±)-**Rh13**</u>

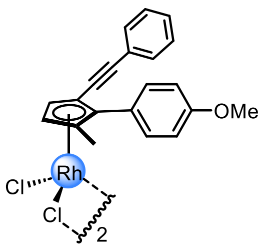

According to *General Procedure 7a*, starting from cyclopentadiene **Cp31** (0.08 mmol), racemic alkynyl-substituted Rh(III) complex **Rh13** (10 mg, 0.02 mmol of monomer, 26% yield over 2 steps) was obtained as a red solid.

^{1}H NMR (600 MHz, d_6-DMSO) δ = 7.82 – 7.79 (m, 2H), 7.53 – 7.47 (m, 3H), 7.46 – 7.43 (m, 2H), 7.10 – 7.04 (m, 2H), 6.17 (d, *J* = 2.5 Hz, 1H), 5.96 (d, *J* = 2.5 Hz, 1H), 3.82 (s, 3H), 1.90 (s, 3H) ppm; **^{13}C{^{1}H} NMR** (151 MHz, d_6-DMSO) δ = 160.2, 131.9, 131.6, 130.2, 129.0, 121.0, 119.5, 113.7, 107.9 (d, *J* = 6.0 Hz), 103.5 (d, *J* = 5.0 Hz), 97.9, 89.9 (d, *J* = 6.3 Hz), 84.4 (d, *J* = 7.6 Hz), 83.8 (d, *J* = 7.6 Hz), 81.1, 55.3, 12.5 ppm; **IR** (ATR) ṽ = 2214 (m), 1606 (m), 1573 (w), 1521 (m), 1496 (m), 1443 (m), 1405 (w), 1292 (w), 1254 (s), 1177 (m), 1028 (m), 911 (w), 834 (m), 758 (m), 729 (m), 690 (m), 567 (w), 559 (w) cm^{-1}; **HRMS** (ESI/QTOF) *m/z* = calcd. for $[C_{21}H_{17}ClORh]^+$, $[(M/2)\text{-}Cl^-]^+$: 423.0017, found: 423.0020.

Rhodium complex (±)-**Rh14**

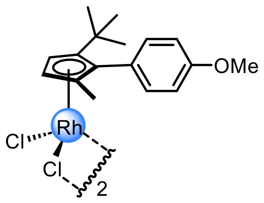

According to *General Procedure 7a*, starting from cyclopentadiene **Cp34** (0.14 mmol), racemic Rh(III) complex **Rh14** (23 mg, 0.05 mmol of monomer, 38% yield over 2 steps) was obtained as an orange solid. Notably, two aromatic hydrogens show very broad ^{1}H NMR signals, and their attached carbons are unresolved in the ^{13}C NMR spectrum.

^{1}H NMR (400 MHz, d_6-DMSO) δ = 7.87 (br s, 1H), 7.36 (br s, 1H), 6.99 – 6.93 (m, 2H), 5.90 (d, *J* = 2.5 Hz, 1H), 5.41 (d, *J* = 2.5 Hz, 1H), 3.79 (s, 3H), 1.46 (s, 3H), 1.14 (s, 9H) ppm; **^{13}C{^{1}H} NMR** (101 MHz, d_6-DMSO) δ = 159.3, 122.9 (d, *J* = 6.8 Hz), 122.1, 118.0 (d, *J* = 6.5 Hz), 113.5, 96.5 (d, *J* = 6.3 Hz), 80.0 (d, *J* = 8.5 Hz), 77.1 (d, *J* = 8.8 Hz), 55.1, 33.5, 29.5, 12.3 ppm; **IR** (ATR) ṽ = 2961 (w), 2912 (w), 1609 (w), 1519 (m), 1464 (w), 1455 (w), 1435 (w), 1417 (w), 1402 (w), 1289 (w), 1250 (s), 1180 (w), 1032 (w), 833 (w), 795 (w), 559 (w) cm^{-1}; **HRMS** (ESI/QTOF) *m/z* = calcd. for $[C_{17}H_{21}ClORh]^+$, $[(M/2)-Cl^-]^+$: 379.0330, found: 379.0317.

Rhodium complex (±)-**Rh15**

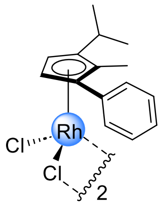

According to *General Procedure 7a*, starting from cyclopentadiene **Cp28** (0.20 mmol), racemic Rh(III) complex **Rh15** (38 mg, 0.10 mmol of monomer, 51% yield over 2 steps) was obtained as an orange-red solid.

^{1}H NMR (600 MHz, d_6-DMSO) δ = 7.71 – 7.68 (m, 2H), 7.53 – 7.49 (m, 1H), 7.47 – 7.43 (m, 2H), 6.02 (d, *J* = 2.6 Hz, 1H), 5.88 (d, *J* = 2.7 Hz, 1H), 2.65 (hept, *J* = 6.9 Hz, 1H), 1.92 (s, 3H), 1.29 (d, *J* = 6.7 Hz, 3H), 1.19 (d, *J* = 7.0 Hz, 3H) ppm; **^{13}C{^{1}H} NMR** (151 MHz, d_6-DMSO) δ = 130.1, 129.7, 128.7, 128.4, 113.4 (d, *J* = 6.8 Hz), 107.3 (d, *J* = 6.8 Hz), 99.0 (d, *J* = 6.0 Hz), 83.0 (d, *J* = 8.1 Hz), 80.1 (d, *J* = 8.2 Hz), 24.8, 21.6, 19.5, 10.5 ppm; **IR** (ATR) ṽ = 3063 (w), 2966 (m), 2929 (w), 2873 (w), 1474 (w), 1440 (m), 1416 (w), 1388 (w), 1367 (w), 1078 (w), 1063 (w), 1028 (w), 854 (w), 768 (m), 732 (w), 700 (s), 649 (w) cm^{-1}; **HRMS** (nanochip-ESI/LTQ-Orbitrap) *m/z* = calcd. for $[C_{15}H_{17}ClRh]^+$, $[(M/2)-Cl^-]^+$: 335.0068, found: 335.0074.

Rhodium complex **Rh16**

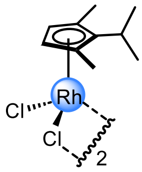

According to *General Procedure 7a*, starting from cyclopentadiene **Cp3** (0.34 mmol), Rh(III) complex **Rh16** (90 mg, 0.29 mmol of monomer, 86% yield over 2 steps) was obtained as an orange-red solid.

^{1}H NMR (400 MHz, d_6-DMSO) δ = 5.49 (s, 2H), 2.58 (hept, *J* = 7.1 Hz, 1H), 1.80 (s, 6H), 1.25 (d, *J* = 7.1 Hz, 6H) ppm; **^{13}C{^{1}H} NMR** (101 MHz, d_6-DMSO) δ = 109.6 (d, *J* = 6.7 Hz), 103.2 (d, *J* = 6.4 Hz), 81.9 (d, *J* = 8.6 Hz), 24.6, 20.2, 11.9 ppm; **IR** (ATR) ṽ = 2971 (w), 1469 (w), 1441 (w), 1366 (w), 1072 (w), 1036 (w), 923 (m), 907 (w), 878 (w), 728 (s), 690 (w), 643 (w), 506 (w) cm^{-1}; **HRMS** (ESI/QTOF) *m/z* = calcd. for $[C_{10}H_{15}ClRh]^+$, $[(M/2)-Cl^-]^+$: 272.9912, found: 272.9914.

Rhodium complex (±)-**Rh17**

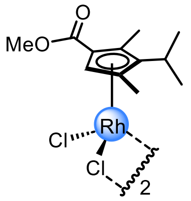

According to *General Procedure 7a*, starting from cyclopentadiene **Cp36** (0.19 mmol), racemic tetrasubstituted Rh(III) complex **Rh17** (27 mg, 0.07 mmol of monomer, 38% yield over 2 steps) was obtained as a red solid.

^{1}H NMR (800 MHz, d_6-DMSO) δ = 6.16 (s, 1H), 3.74 (s, 3H), 2.62 (hept, J = 7.1 Hz, 1H), 2.11 (s, 3H), 1.85 (s, 3H), 1.27 (d, J = 7.1 Hz, 3H), 1.25 (d, J = 7.1 Hz, 3H) ppm; **^{13}C{^{1}H} NMR** (101 MHz, d_6-DMSO) δ = 164.2, 113.7 (d, J = 5.8 Hz), 109.1 (d, J = 6.4 Hz), 105.7 (d, J = 6.2 Hz), 85.9 (d, J = 7.8 Hz), 77.9 (d, J = 9.0 Hz), 52.7, 24.7, 20.4, 19.8, 11.5, 10.8 ppm; **IR** (ATR) ṽ = 2969 (w), 1729 (s), 1437 (m), 1401 (w), 1369 (w), 1338 (w), 1234 (s), 1080 (w), 1053 (m), 1029 (w), 941 (w), 775 (w), 732 (m), 699 (w) cm^{-1}; **HRMS** (ESI/QTOF) m/z = calcd. for $[C_{12}H_{17}ClO_2Rh]^+$, $[(M/2)-Cl^-]^+$: 330.9967, found: 330.9972.

Rhodium complex (±)-**Rh18**

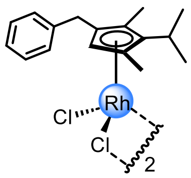

According to *General Procedure 7b*, starting from cyclopentadiene **Cp37** (0.27 mmol, 1.0 equiv.) and $RhCl_3$·3 H_2O (0.27 mmol) in isopropanol at 90 °C, racemic tetrasubstituted Rh(III) complex **Rh18** (69 mg, 0.17 mmol of monomer, 65% yield) was obtained as an orange-red solid.

^{1}H NMR (400 MHz, d_6-DMSO) δ = 7.36 – 7.30 (m, 2H), 7.30 – 7.22 (m, 3H), 5.79 (s, 1H), 3.48 (d, J = 15.3 Hz, 1H), 3.44 (d, J = 15.3 Hz, 1H), 2.59 (hept, J = 7.1 Hz, 1H), 1.80 (s, 3H), 1.79 (s, 3H), 1.23 (d, J = 7.1 Hz, 6H) ppm; **^{13}C{^{1}H} NMR** (101 MHz, d_6-DMSO) δ = 137.1, 128.8, 128.6, 126.8, 106.0 (d, J = 6.5 Hz), 105.3 (d, J = 7.3 Hz), 104.1 (d, J = 6.9 Hz), 98.6 (d, J = 8.0 Hz), 83.2 (d, J = 8.4 Hz), 30.0, 24.8, 20.4, 20.0, 11.4, 9.8 ppm; **IR** (ATR) ṽ = 2968 (w), 2929 (w), 1495 (w), 1472 (m), 1453 (m), 1439 (m), 1369 (w), 1057 (w), 1029 (w), 1002 (w), 750 (w), 733 (m), 705 (s), 463 (w) cm^{-1}; **HRMS** (ESI/QTOF) m/z = calcd. for $[C_{17}H_{21}ClRh]^+$, $[(M/2)-Cl^-]^+$: 363.0381, found: 363.0383.

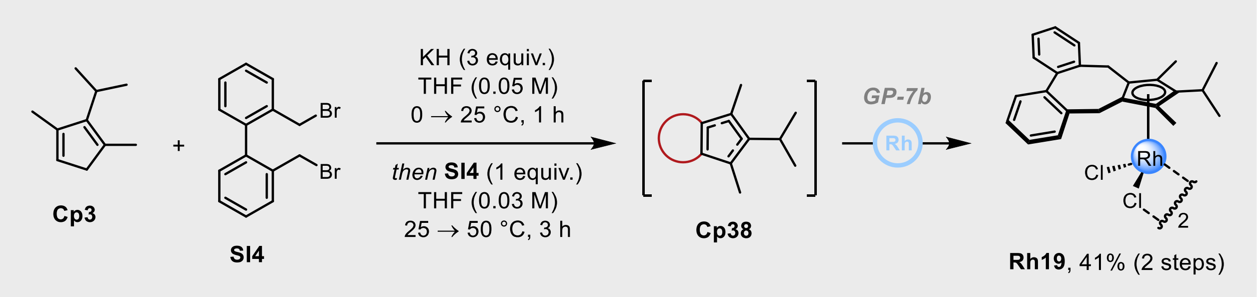

Scheme S20. Telescoped dialkylation-complexation of 1,2,3-Cps towards pentasubstituted CpRh complexes.

Rhodium complex **Rh19**

According to the following two-step procedure (**Scheme S20**), Rh(III) complex **Rh19** was obtained.

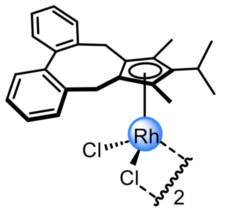

Part 1. In a nitrogen-filled glovebox, an oven-dried microwave vial was charged with potassium hydride (deoiled, 60 mg, 3.0 equiv.) and capped. Outside of the glovebox, it was connected to a Schlenk line and anhydrous THF (10 mL/mmol) was added. At 0 °C (ice bath), a solution of 1,2,3-trisubstituted cyclopentadiene **Cp3** (68 mg, 0.50 mmol, 1 equiv.) in anhydrous THF (10 mL/mmol) was added. The ice bath was removed, and the light-yellow suspension was allowed to warm up to room temperature (25 °C) under vigorous stirring for 1 hour, slowly turning light-brown. Then, at 25 °C, a solution of bis(bromomethyl)biphenyl **SI4** (170 mg, 0.50 mmol, 1.0 equiv.) in anhydrous THF (10 mL/mmol) was added to the potassium cyclopentadienide solution. The obtained reaction mixture was stirred for 3 hours in a heating block at 50 °C. After cooling to room temperature (25 °C), the brown reaction mixture was carefully quenched with sat. aq. NH_4Cl solution, then diluted with water, after which it was extracted with Et_2O (3 x). The combined organic layers were washed with brine, dried over $MgSO_4$, filtered, and concentrated under reduced pressure, affording the pentasubstituted cyclopentadiene **Cp38** as a complex mixture of double bond isomers.

Part 2. The crude cyclopentadiene **Cp38** was complexed to rhodium according to *General Procedure 7b* with $RhCl_3{\cdot}3\ H_2O$ (132 mg, 1.0 equiv.) in isopropanol at 90 °C, affording pentasubstituted Rh(III) complex **Rh19** (99 mg, 0.20 mmol of monomer, 41% yield over 2 steps) as a dark red solid.

^{1}H NMR (800 MHz, *d*$_6$-DMSO) δ = 7.52 – 7.49 (m, 1H), 7.41 – 7.37 (m, 2H), 7.36 – 7.32 (m, 3H), 7.31 – 7.29 (m, 1H), 7.15 – 7.11 (m, 1H), 3.44 (d, *J* = 16.4 Hz, 1H), 3.41 (d, *J* = 14.2 Hz, 1H), 3.21 (d, *J* = 14.2 Hz, 1H), 2.68 (hept, *J* = 7.2 Hz, 1H), 2.55 (d, *J* = 16.6 Hz, 1H), 1.87 (s, 3H), 1.85 (s, 3H), 1.30 (d, *J* = 7.3 Hz, 3H), 1.19 (d, *J* = 7.2 Hz, 3H) ppm; **^{13}C{^{1}H} NMR** (151 MHz, *d*$_6$-DMSO) δ = 141.4, 141.3, 134.6, 133.0, 132.7, 129.0, 128.63, 128.59, 128.4, 127.7, 127.6, 127.3, 105.4 (d, *J* = 6.4 Hz), 105.3 (d, *J* = 7.4 Hz), 102.9 (d, *J* = 6.8 Hz), 95.3 (d, *J* = 6.4 Hz), 93.0 (d, *J* = 7.8 Hz), 29.6, 26.8, 25.2, 20.3, 20.0, 9.4, 8.5 ppm; **IR** (ATR) ṽ = 3059 (w), 3019 (w), 2967 (m), 2930 (w), 2872 (w), 1479 (m), 1445 (m), 1370 (w), 1266 (w), 1026 (w), 1008 (w), 755 (s), 731 (m), 698 (w) cm^{-1}; **HRMS** (nanochip-ESI/LTQ-Orbitrap) *m/z* = calcd. for $[C_{24}H_{25}ClRh]^+$, $[(M/2)\text{-}Cl^-]^+$: 451.0694, found: 451.0690.

5.3 Cp Rhodium Phosphite Adducts

General Procedure 8 – *In-situ Conversion of [CpRhCl2]2 Complexes into Phosphite Adducts.*

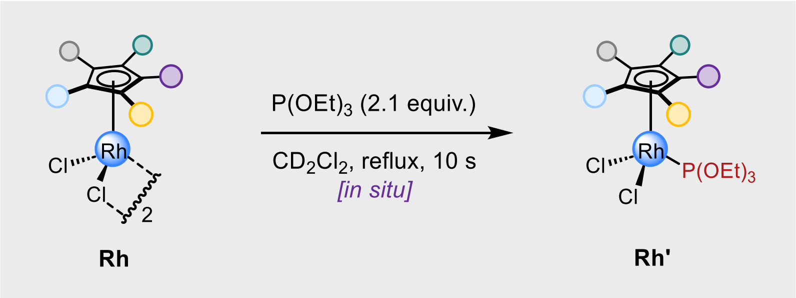

Scheme S21. In-situ conversion of [CpRhCl2]2 complexes into monomeric phosphite adducts.

In a screw-cap vial, to a solution of an analytical sample of the dimeric $[CpRhCl_2]_2$-type rhodium(III) complex **Rh** (*ca.* 2 mg, 1.0 equiv.) in CD_2Cl_2 (0.5 mL) was added a triethyl phosphite solution (0.13 M in CD_2Cl_2, 2.1 equiv.). The vial was closed tightly under air and shortly heated (*ca.* 10 seconds) at 80 °C to form the desired $CpRhCl_2P(OEt)_3$ adduct **Rh'** in situ. After cooling to room temperature (25 °C), the ^{31}P NMR spectrum was recorded immediately. If desired, purification could be performed by flash column chromatography on silica gel (wet loading with DCM, 5 cm column height, isocratic: DCM *then* DCM/Et_2O = 1:1), affording the corresponding phosphite adduct **Rh'** (usually *ca.* 95% yield).

Rhodium phosphite adduct **Cp*Rh'**

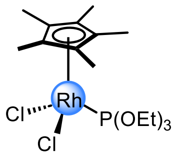

According to *General Procedure 8*, starting from an analytical sample of dimeric Rh(III) complex **Cp*Rh** (2.5 mg), phosphite adduct **Cp*Rh'** was obtained as a red solid with the characterization data matching those previously reported.[33]

$^{31}P\{^1H\}$ NMR (162 MHz, CD_2Cl_2) δ = 114.16 (d, J = 215.4 Hz) ppm.

Rhodium phosphite adduct **Rh1'**

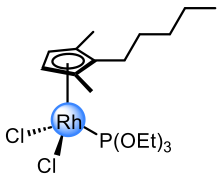

According to *General Procedure 8*, starting from an analytical sample of dimeric Rh(III) complex **Rh1** (2.1 mg), phosphite adduct **Rh1'** was obtained as a red solid.

$^{31}P\{^1H\}$ NMR (162 MHz, CD_2Cl_2) δ = 115.33 (d, J = 207.9 Hz) ppm; **HRMS** (ESI/QTOF) m/z = calcd. for $[C_{18}H_{34}ClO_3PRh]^+$, $[M-Cl^-]^+$: 467.0984, found: 467.0992.

Rhodium phosphite adduct **Rh2'**

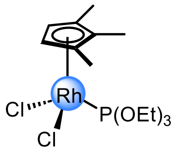

According to *General Procedure 8*, starting from an analytical sample of dimeric Rh(III) complex **Rh2** (2.2 mg), phosphite adduct **Rh2'** was obtained as a red solid.

$^{31}P\{^1H\}$ NMR (162 MHz, CD_2Cl_2) δ = 115.93 (d, J = 208.8 Hz) ppm; **HRMS** (ESI/QTOF) m/z = calcd. for $[C_{14}H_{26}ClO_3PRh]^+$, $[M-Cl^-]^+$: 411.0358, found: 411.0365.

Rhodium phosphite adduct **Rh3'**

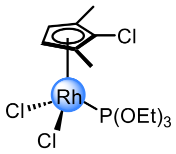

According to *General Procedure 8*, starting from an analytical sample of dimeric Rh(III) complex **Rh3** (2.3 mg), phosphite adduct **Rh3'** was obtained as a red solid.

^{31}P{^{1}H} NMR (162 MHz, CD_2Cl_2) δ = 110.23 (d, *J* = 206.8 Hz) ppm; **HRMS** (ESI/QTOF) *m/z* = calcd. for $[C_{13}H_{23}Cl_2O_3PRh]^+$, $[M-Cl^-]^+$: 430.9811, found: 430.9809.

Rhodium phosphite adduct **Rh5'**

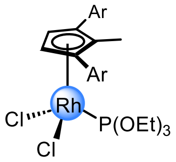

(Ar = 3,5-CF_3-C_6H_3)

According to *General Procedure 8*, starting from an analytical sample of dimeric Rh(III) complex **Rh5** (3.1 mg), phosphite adduct **Rh5'** was obtained as a red solid.

^{31}P{^{1}H} NMR (162 MHz, CD_2Cl_2) δ = 104.23 (d, *J* = 199.2 Hz) ppm; **HRMS** (ESI/QTOF) *m/z* = calcd. for $[C_{28}H_{26}ClF_{12}O_3PRh]^+$, $[M-Cl^-]^+$: 807.0166, found: 807.0167.

Rhodium phosphite adduct **Rh6'**

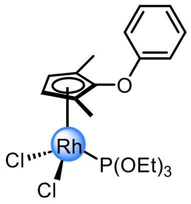

According to *General Procedure 8*, starting from an analytical sample of dimeric Rh(III) complex **Rh6** (2.1 mg), phosphite adduct **Rh6'** was obtained as a red solid.

^{31}P{^{1}H} NMR (162 MHz, CD_2Cl_2) δ = 114.67 (d, *J* = 212.1 Hz) ppm; **HRMS** (ESI/QTOF) *m/z* = calcd. for $[C_{19}H_{28}ClO_4PRh]^+$, $[M-Cl^-]^+$: 489.0463, found: 489.0481.

Rhodium phosphite adduct **Rh7'**

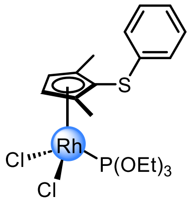

According to *General Procedure 8*, starting from an analytical sample of dimeric Rh(III) complex **Rh7** (3.2 mg), phosphite adduct **Rh7'** was obtained as a red solid.

^{31}P{^{1}H} NMR (162 MHz, CD_2Cl_2) δ = 109.60 (d, *J* = 203.9 Hz) ppm; **HRMS** (ESI/QTOF) *m/z* = calcd. for $[C_{19}H_{28}ClO_3PRhS]^+$, $[M-Cl^-]^+$: 505.0235, found: 505.0246.

Rhodium phosphite adduct **Rh8'**

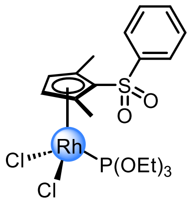

According to *General Procedure 8*, starting from an analytical sample of dimeric Rh(III) complex **Rh8** (0.8 mg), phosphite adduct **Rh8'** was obtained as a red solid.

^{31}P{^{1}H} NMR (162 MHz, CD_2Cl_2) δ = 102.31 (d, *J* = 192.8 Hz) ppm; **HRMS** (ESI/QTOF) *m/z* = calcd. for $[C_{19}H_{28}ClO_5PRhS]^+$, $[M-Cl^-]^+$: 537.0133, found: 537.0130.

Rhodium phosphite adduct (±)-**Rh9'**

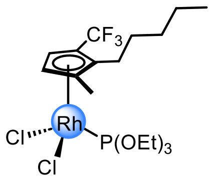

According to *General Procedure 8*, starting from an analytical sample of dimeric Rh(III) complex **Rh9** (2.2 mg), racemic phosphite adduct **Rh9'** was obtained as a red solid.

^{31}P{^{1}H} NMR (162 MHz, CD_2Cl_2) δ = 103.73 (dq, *J* = 198.8, 5.2 Hz) ppm; **HRMS** (ESI/QTOF) *m/z* = calcd. for $[C_{18}H_{31}ClF_3O_3PRh]^+$, $[M-Cl^-]^+$: 521.0701, found: 521.0714.

Rhodium phosphite adduct **Rh10’**

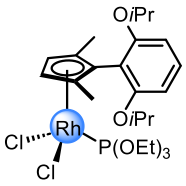

According to *General Procedure 8*, starting from an analytical sample of dimeric Rh(III) complex **Rh10** (1.8 mg), phosphite adduct **Rh10’** was obtained as a red solid.

^{31}P{^{1}H} NMR (162 MHz, CD_2Cl_2) δ = 112.70 (d, *J* = 208.8 Hz) ppm; **HRMS** (ESI/QTOF) *m/z* = calcd. for $[C_{25}H_{40}ClO_5PRh]^+$, $[M-Cl^-]^+$: 589.1351, found: 589.1360.

Rhodium phosphite adduct **Rh11’**

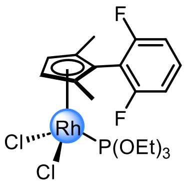

According to *General Procedure 8*, starting from an analytical sample of dimeric Rh(III) complex **Rh11** (2.5 mg), phosphite adduct **Rh11’** was obtained as a red solid.

^{31}P{^{1}H} NMR (162 MHz, CD_2Cl_2) δ = 109.96 (dd, *J* = 203.9, 4.0 Hz) ppm; **HRMS** (ESI/QTOF) *m/z* = calcd. for $[C_{19}H_{26}ClF_2O_3PRh]^+$, $[M-Cl^-]^+$: 509.0326, found: 509.0329.

Rhodium phosphite adduct **Rh12’**

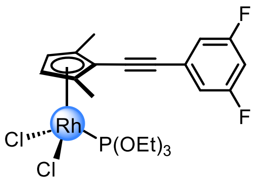

According to *General Procedure 8*, starting from an analytical sample of dimeric Rh(III) complex **Rh12** (1.9 mg), phosphite adduct **Rh12’** was obtained as a red solid. A suitable crystal for X-ray analysis (**Figure S7**) was obtained by slow vapour diffusion of hexane into a concentrated solution in chloroform.

^{31}P{^{1}H} NMR (162 MHz, CD_2Cl_2) δ = 110.01 (d, *J* = 204.7 Hz) ppm; **HRMS** (ESI/QTOF) *m/z* = calcd. for $[C_{21}H_{26}ClF_2O_3PRh]^+$, $[M-Cl^-]^+$: 533.0326, found: 533.0337; **XRD** (Mo*Kα*, R_1 = 6.59%) CCDC: 2503537.

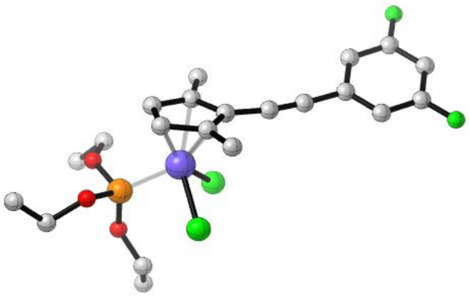

Figure S7. Solid-state X-ray structure of alkynyl-substituted Cp rhodium(III) phosphite adduct **Rh12’** (CCDC: 2503537) showing 50% probability thermal ellipsoids. Hydrogen atoms and disorder are omitted for clarity.

Rhodium phosphite adduct **Rh16’**

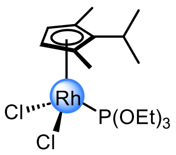

According to *General Procedure 8*, starting from an analytical sample of dimeric Rh(III) complex **Rh16** (2.4 mg), phosphite adduct **Rh16’** was obtained as a red solid.

^{31}P{^{1}H} NMR (162 MHz, CD_2Cl_2) δ = 115.75 (d, *J* = 206.8 Hz) ppm; **HRMS** (ESI/QTOF) *m/z* = calcd. for $[C_{16}H_{30}ClO_3PRh]^+$, $[M-Cl^-]^+$: 439.0671, found: 439.0672.

Rhodium phosphite adduct (±)-**Rh17’**

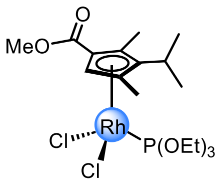

According to *General Procedure 8*, starting from an analytical sample of dimeric Rh(III) complex **Rh17** (1.7 mg), racemic phosphite adduct **Rh17’** was obtained as a red solid. The protocol was repeated successfully on a slightly larger scale (14.0 mg, 19 µmol) in a microwave vial with DCM at 80 °C for 10 min, affording 19.3 mg (18 µmol, 95% yield) of **Rh17’**. Notably, this racemate can be separated by chiral HPLC.

^{1}H NMR (500 MHz, CD_2Cl_2) δ = 4.26 – 4.14 (m, 6H), 3.76 (s, 3H), 2.76 (heptd, *J* = 7.1, 1.7 Hz, 1H), 2.10 (d, *J* = 4.6 Hz, 3H), 1.77 (d, *J* = 1.2 Hz, 3H), 1.37 (d, *J* = 7.1 Hz, 3H), 1.32 – 1.26 (m, 12H) ppm; **^{13}C{^{1}H} NMR** (126 MHz, CD_2Cl_2) δ = 165.6, 118.3 (app t, *J* = 4.4 Hz), 114.8 (dd, *J* = 17.6, 3.7 Hz), 108.7 (dd, *J* = 5.5, 1.4 Hz), 83.7 (dd, *J* = 7.1, 2.2 Hz), 77.2 (d, *J* = 8.8 Hz), 64.5 (d, *J* = 6.7 Hz), 52.6, 25.7 (d, *J* = 4.1 Hz), 21.1 (d, *J* = 4.5 Hz), 20.8 (d, *J* = 7.5 Hz), 16.3 (d, *J* = 6.1 Hz), 12.2, 11.6 (d, *J* = 2.4 Hz) ppm; **^{31}P{^{1}H} NMR** (162 MHz, CD_2Cl_2) δ = 107.20 (d, *J* = 200.9 Hz) ppm; **IR** (ATR) ṽ = 2969 (w), 2927 (w), 1725 (m), 1437 (w), 1390 (w), 1366 (w), 1223 (m), 1159 (w), 1052 (m), 1011 (s), 950 (s), 773 (m), 733 (m), 544 (w) cm^{-1}; **HRMS** (ESI/QTOF) *m/z* = calcd. for $[C_{18}H_{32}ClO_5PRh]^+$, $[M\text{-}Cl^-]^+$: 497.0725, found: 497.0733; **R_f** (DCM/Et_2O, 1:1) = 0.77; **Chiral HPLC** (Shimadzu Prominence UFLC XR system, Chiralpak IA, 4.6 x 150 mm, 3 µm, Hexane/*i*PrOH 90:10, 1.0 mL/min, 35 °C, 254 nm) t_r = 5.87 + 7.26 min.

Rhodium phosphite adduct (±)-**Rh18’**

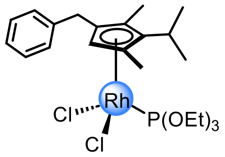

According to *General Procedure 8*, starting from an analytical sample of dimeric Rh(III) complex **Rh18** (2.7 mg), racemicphosphite adduct **Rh18’** was obtained as a red solid.

^{31}P{^{1}H} NMR (162 MHz, CD_2Cl_2) δ = 112.32 (d, *J* = 210.6 Hz) ppm; **HRMS** (ESI/QTOF) *m/z* = calcd. for $[C_{23}H_{36}ClO_3PRh]^+$, $[M\text{-}Cl^-]^+$: 529.1140, found: 529.1156.

Rhodium phosphite adduct **Rh19’**

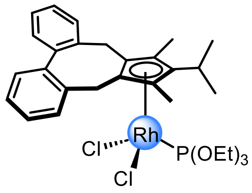

According to *General Procedure 8*, starting from an analytical sample of dimeric Rh(III) complex **Rh19** (3.1 mg), phosphite adduct **Rh19’** was obtained as a red solid.

^{31}P{^{1}H} NMR (162 MHz, CD_2Cl_2) δ = 110.23 (d, *J* = 215.9 Hz) ppm; **HRMS** (ESI/QTOF) *m/z* = calcd. for $[C_{30}H_{40}ClO_3PRh]^+$, $[M\text{-}Cl^-]^+$: 617.1453, found: 617.1460.

6. Benchmark Catalytic Transformations

6.1 Ir-catalyzed C(sp^3)-H Amidation of Oximes

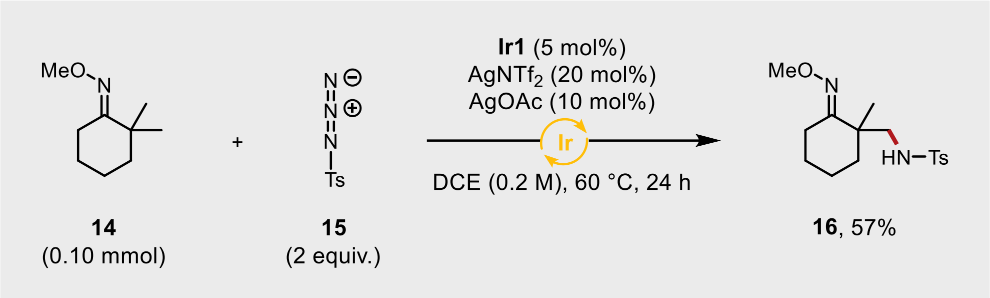

Scheme S22. Ir-catalyzed C(sp^3)-H amidation of oximes.

An oven-dried microwave vial was charged with **Ir1** catalyst (4.3 mg, 5 mol%), silver(I) bis(trifluoromethanesulfonyl)imide (8.0 mg, 20 mol%), and silver(I) acetate (1.7 mg, 10 mol%) under air. The vial was capped and placed under an atmosphere of nitrogen by Schlenk technique. A solution of oxime **14** (15.5 mg, 0.10 mmol, 1.0 equiv.) and tosyl azide **15** (39 mg, 2.0 equiv.) in DCE (5 mL/mmol) was added, and the reaction mixture was stirred in a heating block at 60 °C for 24 hours. After cooling to room temperature (25 °C), the reaction was diluted with ethyl acetate and then filtered through a pad of silica (3 cm) with ethyl acetate as eluent. After removal of all volatiles *in vacuo*, the resulting crude residue was analyzed by qNMR in $CDCl_3$ with ethylene carbonate as internal standard. Purification was performed by Prep. TLC (pentane/EtOAc = 20:1), affording amidated product **16** as a transparent oil (18.5 mg, 57 µmol, 57% yield) with the characterization data matching those previously reported.[34]

6.2 Ti-catalyzed Cross-selective Acyloin-type Condensation

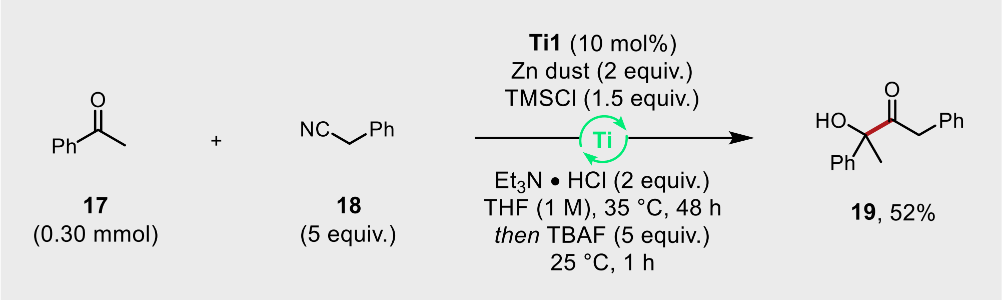

Scheme S23. Ti-catalyzed cross-selective acyloin-type condensation.

An oven-dried microwave vial was charged with **Ti1** catalyst (13.4 mg, 10 mol%), and triethylamine hydrochloride (83 mg, 2 equiv.). Inside a nitrogen-filled glovebox, Zn(0) dust (39 mg, 2 equiv.) and anhydrous degassed THF (1 mL/mmol) were added sequentially. The suspension was stirred for 5 min, capped, and taken outside of the glovebox. Using Hamilton syringes, nitrile **18** (174 µL, 5 equiv.), ketone **17** (35 µL, 0.30 mmol, 1.0 equiv.), and TMSCl (58 µL, 1.5 equiv.) were added sequentially to the reaction mixture, after which it was stirred in a heating block at 35 °C for 48 hours. After cooling to room temperature (25 °C), TBAF (1.0 M in THF, 1.5 mL, 5 equiv.) was added, and the mixture was stirred for 1

hour. Then, water was added (5 mL) and after 5 min of additional stirring, it was extracted with DCM (3x), dried over $MgSO_4$, filtered, and concentrated under reduced pressure. The resulting crude residue was analyzed by qNMR in $CDCl_3$ with 1,3,5-trimethoxybenzene as internal standard. Purification was performed by flash column chromatography on silica gel (wet loading with DCM, 15 cm column height, isocratic: DCM), affording α-hydroxyketone **19** (37.5 mg, 156 μmol, 52% yield) as transparent oil with the characterization data matching those previously reported.[35]

6.3 Rh-catalyzed C-H Annulation for 2-Substituted Indolines

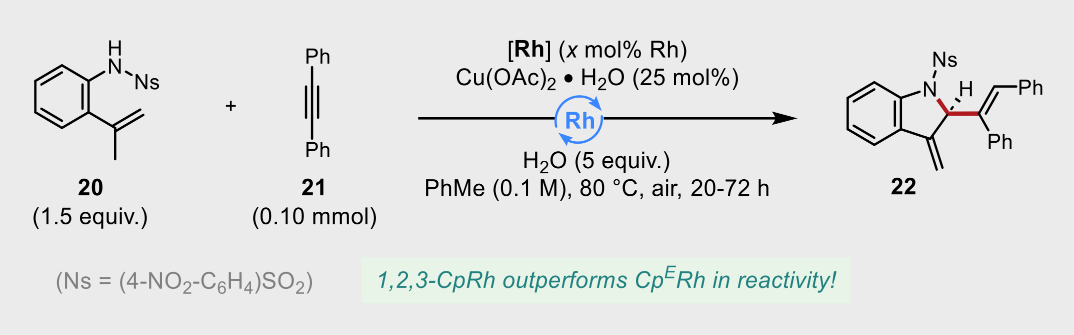

Scheme S24. Rh-catalyzed C-H annulation for 2-substituted indolines.

An oven-dried microwave vial was charged with **Rh** catalyst (*x* mol% CpRh monomer, with *x* = 2-6), copper(II) acetate monohydrate (5.0 mg, 25 mol%), aniline **20** (47.8 mg, 1.5 equiv.), and diphenylacetylene **21** (17.8 mg, 0.10 mmol, 1.0 equiv.). Water (9 μL, 5 equiv.) was carefully added at the bottom of the vial. Next, toluene (10 mL/mmol) was added, the vial was capped under air, and the reaction mixture was stirred in a heating block at 80 °C with an air-filled balloon attached for 20-72 hours. After cooling to room temperature (25 °C), the reaction was diluted with ethyl acetate and then filtered through a pad of silica (3 cm) with ethyl acetate as eluent. After removal of all volatiles *in vacuo*, the resulting crude residue was analyzed by qNMR in $CDCl_3$ with 1,3,5-trimethoxybenzene as internal standard. Purification was performed by Prep. TLC (pentane/DCM/EtOAc = 8:2:0.5), affording 2-substituted indoline **22** as a yellow solid with the characterization data matching those previously reported.[36]

The results of the catalyst screening are visualized in **Figure S8**.

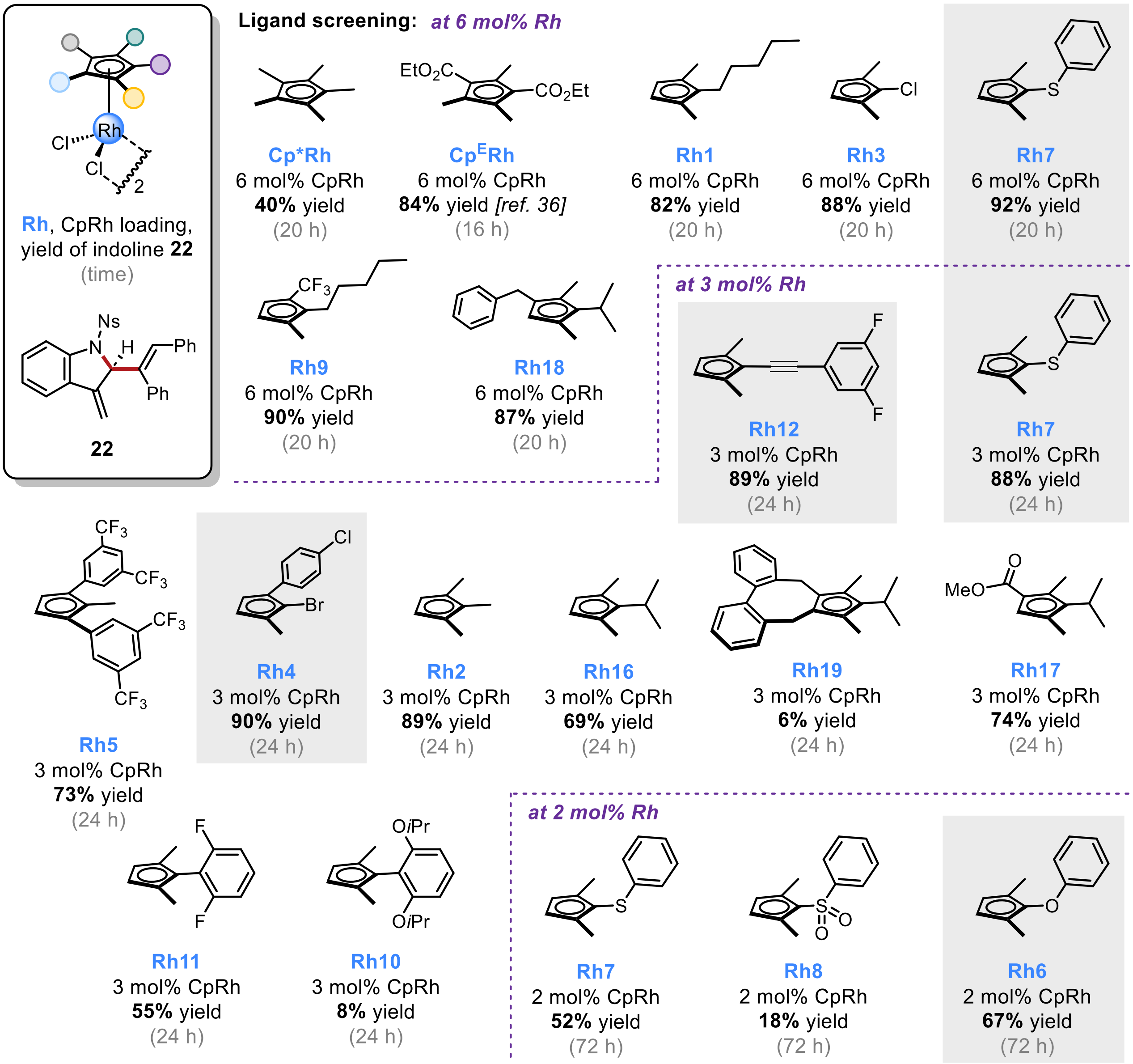

Figure S8. Catalytic efficiency of different Cp ligands in the Rh(III)-catalyzed C-H annulation for 2-substituted indolines.

6.4 Rh-catalyzed Regioselective Pyridine Synthesis

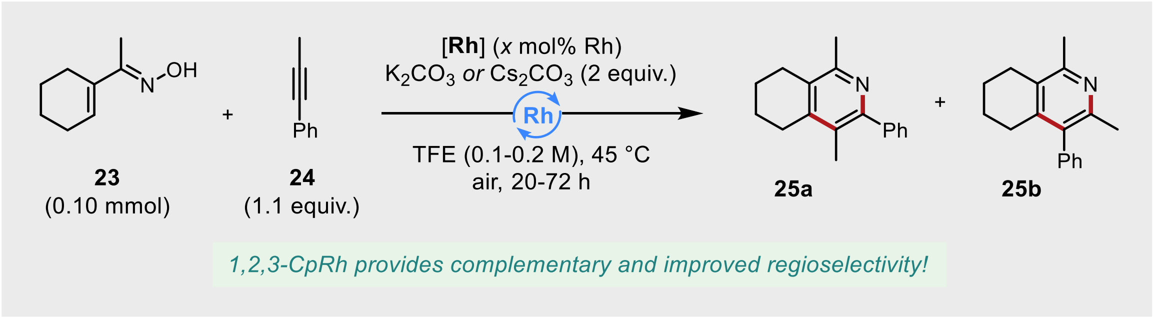

Scheme S25. Rh-catalyzed regioselective pyridine synthesis from oximes.

An oven-dried microwave vial was charged with **Rh** catalyst (*x* mol% CpRh monomer, with *x* = 1-5), potassium carbonate (28 mg, 2.0 equiv) or cesium carbonate (65 mg, 2.0 equiv.), and oxime **23** (13.9 mg, 0.10 mmol, 1.0 equiv.). A solution of unsymmetrical alkyne **24** (14 µL, 1.1 equiv.) in TFE (5-10 mL/mmol) was added, the vial was capped under air, and the reaction mixture was stirred in a heating block at 45 °C for 20-72 hours. After cooling to room temperature (25 °C), the reaction was diluted with ethyl acetate and then filtered through a pad of silica (3 cm) with ethyl acetate as eluent. After removal of all volatiles *in vacuo*, the resulting crude residue was analyzed by qNMR in $CDCl_3$ with 1,3,5-trimethoxybenzene as internal standard. Purification was performed by Prep. TLC (pentane/EtOAc = 4:1), affording pyridines **25a/25b** as a transparent oil with the characterization data matching those previously reported.[37]

The results of the catalyst screening are visualized in **Figure S9**.

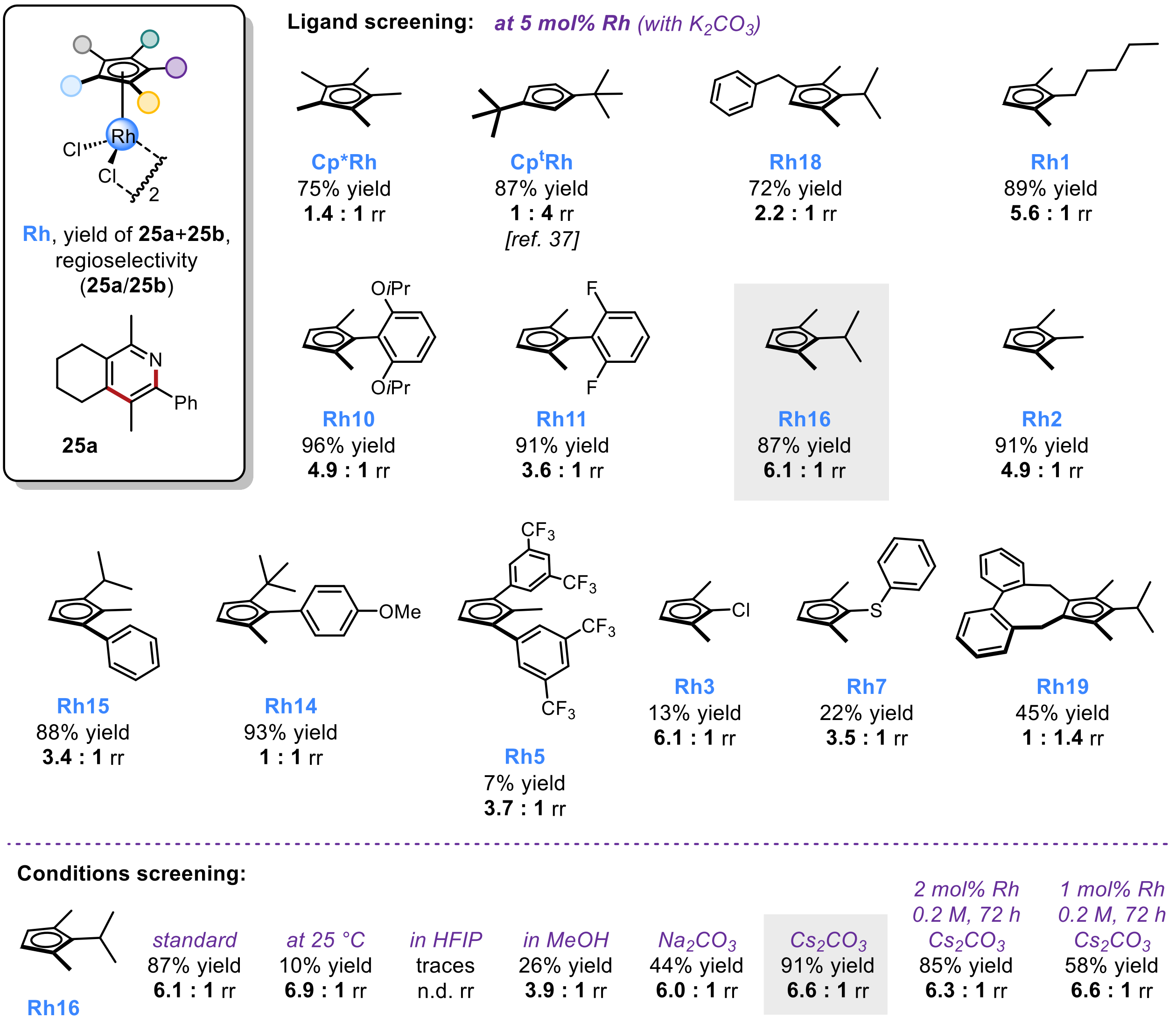

Figure S9. Ligand-controlled regioselectivity for different Cp ligands in Rh(III)-catalyzed pyridine synthesis.

6.5 Co-catalyzed C-H Annulation for Dihydroisoquinolones

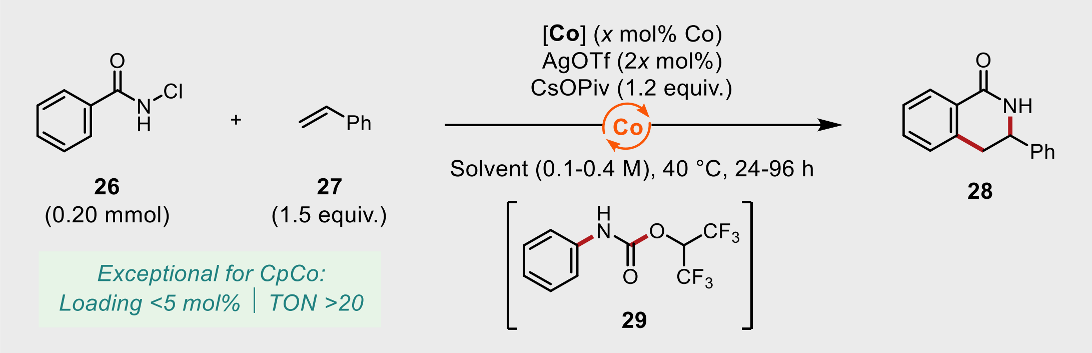

Scheme S26. Co-catalyzed C-H annulation for dihydroisoquinolones.

An oven-dried microwave vial was charged with **Co** catalyst (*x* mol% CpCo monomer, with *x* = 0.1-10), silver(I) triflate (2*x* mol%), cesium pivalate (57 mg, 1.2 equiv.), and *N*-chlorobenzamide **26** (31.1 mg, 0.20 mmol, 1.0 equiv) under air. The vial was capped and placed under an atmosphere of nitrogen by Schlenk technique. A solution of styrene **27** (35 µL, 1.5 equiv.) in the desired solvent (HFIP or NFTB, 2.5-10 mL/mmol) was added, and the reaction mixture was stirred in a heating block at 40 °C for 24-96 hours. After cooling to room temperature (25 °C), the reaction was diluted with ethyl acetate and then filtered through a pad of silica (3 cm) with ethyl acetate as eluent. After removal of all volatiles *in vacuo*, the resulting crude residue was analyzed by qNMR in $CDCl_3$ with ethylene carbonate as internal standard. Purification was performed by Prep. TLC (pentane/EtOAc = 3:2), affording dihydroisoquinolone **28** as a white solid with the characterization data matching those previously reported.[38]

The results of the catalyst loading investigation are given in **Table 3** of the main article.

7. X-ray Crystallographic Data

Crystallographic data for the structures reported in this paper has been deposited at the Cambridge Crystallographic Data Center (CCDC) as Supplementary Publication No. 2479661 (**Cp30**), 2310525 (**Co1**), 2418643 (**Ir1**), 2418644 (**Ti1**), 2479662 (**Rh3**), 2479660 (**Rh4**), 2479664 (**Rh5**), 2479665 (**Rh6**), 2479663 (**Rh7**), 2479659 (**Rh9**), and 2503537 (**Rh12’**). Copies of the data can be obtained free of charge on application to the CCDC via https://www.ccdc.cam.ac.uk/data_request/cif.

Cyclopentadiene **Cp30**

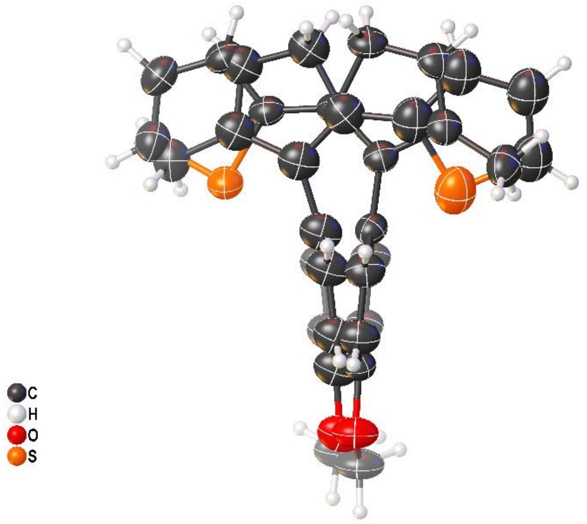

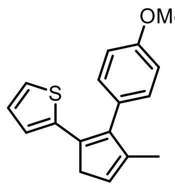

Cp30

Compound	Cp30
Formula	$C_{17}H_{16}OS$
$D_{calc.}$/ g cm^{-3}	1.246
μ/mm^{-1}	1.905
Formula Weight	268.36
Colour	colourless
Shape	prism-shaped
Size/mm^3	0.20×0.13×0.07
T/K	200.00(10)
Crystal System	monoclinic
Space Group	$P2_1/c$
a/Å	13.2493(12)
b/Å	15.2489(13)
c/Å	7.0987(5)
α/°	90
β/°	94.227(8)
γ/°	90
V/Å^3	1430.3(2)
Z	4
Z'	1
Wavelength/Å	1.54184
Radiation type	Cu K_α
Θ_{min}/°	3.345
Θ_{max}/°	76.826
Measured Refl's.	8656
Indep't Refl's	2963
Refl's I≥2σ(I)	2305
R_{int}	0.0319
Parameters	336
Restraints	294
Largest Peak/eÅ^3	0.175
Deepest Hole/eÅ^3	-0.350
GooF	1.033
wR_2 (all data)	0.1403
wR_2	0.1282
R_1 (all data)	0.0648
R_1	0.0497
CCDC number	2479661

Experimental. Single colourless prism-shaped crystals of **Cp30** were used as supplied. A suitable crystal with dimensions 0.20 × 0.13 × 0.07 mm^3 was selected and mounted on a SuperNova, Dual, Cu at home/near, Atlas diffractometer. The crystal was kept at a steady T = 200.00(10) K during data collection. The structure was solved with the ShelXT 2018/2 solution program[39] using iterative methods and by using Olex2 1.5 as the graphical interface.[40] The model was refined with ShelXL 2019/3 using full matrix least squares minimisation on $\boldsymbol{F}^2$.[41]

Crystal Data. $C_{17}H_{16}OS$, M_r = 268.36, monoclinic, $P2_1/c$ (No. 14), a = 13.2493(12) Å, b = 15.2489(13) Å, c = 7.0987(5) Å, β = 94.227(8)°, $\alpha = \gamma$ = 90°, V = 1430.3(2) Å^3, T = 200.00(10) K, Z = 4, Z' = 1, μ(Cu K_α) = 1.905, 8656 reflections measured, 2963 unique (R_{int} = 0.0319) which were used in all calculations. The final wR_2 was 0.1403 (all data) and R_1 was 0.0497 (I≥2σ(I)).

Cobalt(III) complex **Co1**

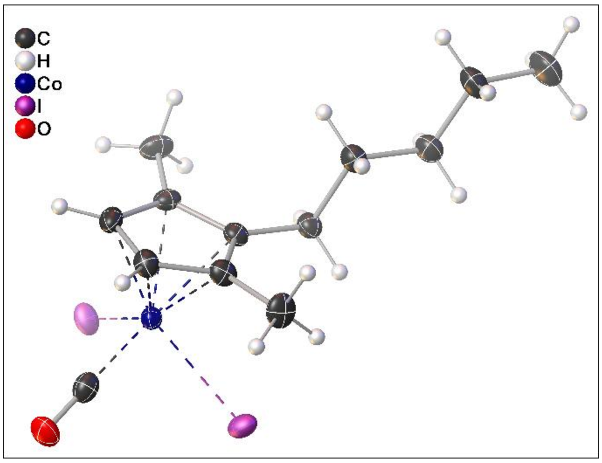

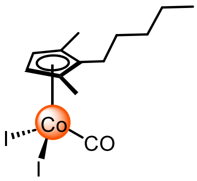

Co1

Experimental. Single clear dark black plate-shaped crystals of **Co1** were used as supplied. A suitable crystal with dimensions 0.13 × 0.02 × 0.01 mm³ was selected and mounted on an XtaLAB Synergy R, DW system, HyPix-Arc 150 diffractometer. The crystal was kept at a steady T = 140.00(10) K during data collection. The structure was solved with the ShelXT 2018/2 solution program[39] using dual methods and by using Olex2 1.5 as the graphical interface.[40] The model was refined with ShelXL 2019/3 using full matrix least squares minimisation on $\boldsymbol{F}^2$.[41]

Crystal Data. $C_{13}H_{19}CoI_2O$, M_r = 504.01, orthorhombic, $P2_12_12_1$ (No. 19), a = 6.16148(6) Å, b = 14.85920(13) Å, c = 53.1158(4) Å, $\alpha = \beta = \gamma$ = 90°, V = 4863.00(7) Å³, T = 140.00(10) K, Z = 12, Z' = 3, μ(Cu K$_\alpha$) = 38.072, 113284 reflections measured, 10047 unique (R_{int} = 0.0428) which were used in all calculations. The final wR_2 was 0.0410 (all data) and R_1 was 0.0183 (I≥2σ(I)).

**Compound**	**Co1**
Formula	$C_{13}H_{19}CoI_2O$
$D_{calc.}$/ g cm^{-3}	2.065
μ/mm^{-1}	38.072
Formula Weight	504.01
Colour	clear dark black
Shape	plate-shaped
Size/mm³	0.13×0.02×0.01
T/K	140.00(10)
Crystal System	orthorhombic
Flack Parameter	-0.035(2)
Space Group	$P2_12_12_1$
a/Å	6.16148(6)
b/Å	14.85920(13)
c/Å	53.1158(4)
α/°	90
β/°	90
γ/°	90
V/Å³	4863.00(7)
Z	12
Z'	3
Wavelength/Å	1.54184
Radiation type	Cu$K\alpha$
Θ_{min}/°	3.088
Θ_{max}/°	76.042
Measured Refl's.	113284
Indep't Refl's	10047
Refl's I≥2σ(I)	9615
R_{int}	0.0428
Parameters	469
Restraints	0
Largest Peak/e Å^{-3}	0.592
Deepest Hole/e Å^{-3}	-0.678
GooF	1.035
wR_2 (all data)	0.0410
wR_2	0.0406
R_1 (all data)	0.0202
R_1	0.0183
CCDC number	2310525

Iridium(III) complex **Ir1**

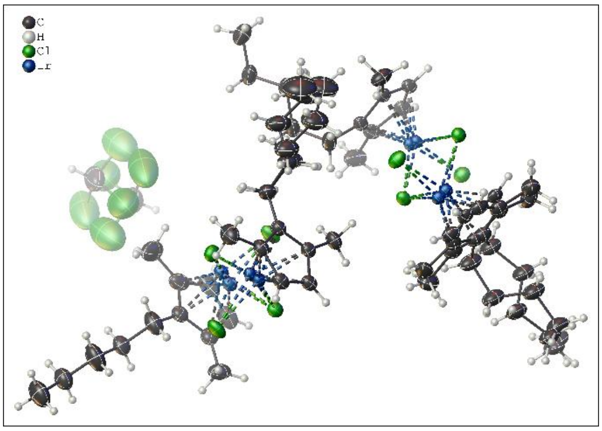

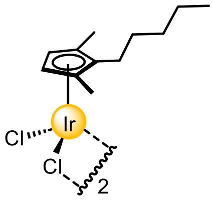

Ir1

Experimental. Single clear intense orange block-shaped crystals of **Ir1** were used as supplied. A suitable crystal with dimensions 0.19 × 0.10 × 0.09 mm^3 was selected and mounted on an XtaLAB Synergy R, DW system, HyPix-Arc 150 diffractometer. The crystal was kept at a steady T = 139.99(10) K during data collection. The structure was solved with the ShelXT 2018/2 solution program[39] using dual methods and by using Olex2 1.5 as the graphical interface.[40] The model was refined with ShelXL 2019/3 using full matrix least squares minimisation on $\boldsymbol{F}^2$.[41]

Crystal Data. $C_{24.25}H_{38.25}Cl_{4.75}Ir_2$, M_r = 882.58, triclinic, $P\bar{1}$ (No. 2), a = 9.70323(10) Å, b = 15.01368(16) Å, c = 20.1425(2) Å, α = 104.1250(9)°, β = 97.7080(8)°, γ = 93.3438(8)°, V = 2807.27(5) Å^3, T = 139.99(10) K, Z = 4, Z' = 2, μ(Mo K_α) = 9.935, 70160 reflections measured, 17128 unique (R_{int} = 0.0380) which were used in all calculations. The final wR_2 was 0.0846 (all data) and R_1 was 0.0344 (I≥2σ(I)).

**Compound**	**Ir1**
Formula	$C_{24.25}H_{38.25}Cl_{4.75}Ir_2$
$D_{calc.}$/ g cm^{-3}	2.088
μ/mm^{-1}	9.935
Formula Weight	882.58
Colour	clear intense orange
Shape	block-shaped
Size/mm^3	0.19×0.10×0.09
T/K	139.99(10)
Crystal System	triclinic
Space Group	$P\bar{1}$
a/Å	9.70323(10)
b/Å	15.01368(16)
c/Å	20.1425(2)
α/°	104.1250(9)
β/°	97.7080(8)
γ/°	93.3438(8)
V/Å^3	2807.27(5)
Z	4
Z'	2
Wavelength/Å	0.71073
Radiation type	Mo$K\alpha$
Θ_{min}/°	1.960
Θ_{max}/°	30.508
Measured Refl's.	70160
Indep't Refl's	17128
Refl's I≥2σ(I)	14258
R_{int}	0.0380
Parameters	743
Restraints	1305
Largest Peak/e Å^{-3}	3.360
Deepest Hole/e Å^{-3}	-0.860
GooF	1.023
wR_2 (all data)	0.0846
wR_2	0.0808
R_1 (all data)	0.0457
R_1	0.0344
CCDC number	2418643

Titanium(IV) complex **Ti1**

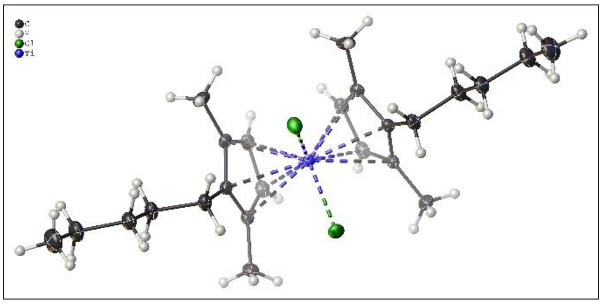

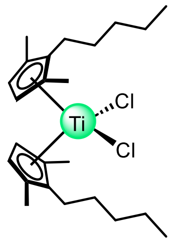

Ti1

Experimental. Single clear intense orange block-shaped crystals of **Ti1** were used as supplied. A suitable crystal with dimensions 0.25 × 0.12 × 0.10 mm^3 was selected and mounted on an XtaLAB Synergy R, DW system, HyPix-Arc 150 diffractometer. The crystal was kept at a steady T = 139.99(10) K during data collection. The structure was solved with the ShelXT 2018/2 solution program[39] using dual methods and by using Olex2 1.5 as the graphical interface.[40] The model was refined with ShelXL 2019/3 using full matrix least squares minimisation on F^2.[41]

Crystal Data. $C_{24}H_{38}Cl_2Ti$, M_r = 445.34, monoclinic, $P2_1/m$ (No. 11), a = 6.45178(18) Å, b = 25.4553(5) Å, c = 7.4727(2) Å, β = 106.175(3)°, α = γ = 90°, V = 1178.68(6) Å^3, T = 139.99(10) K, Z = 2, Z' = 0.5, μ(Mo K$_\alpha$) = 0.597, 20041 reflections measured, 6497 unique (R_{int} = 0.0302) which were used in all calculations. The final wR_2 was 0.0871 (all data) and R_1 was 0.0337 (I≥2σ(I)).

**Compound**	**Ti1**
Formula	$C_{24}H_{38}Cl_2Ti$
$D_{calc.}$/ g cm^{-3}	1.255
μ/mm^{-1}	0.597
Formula Weight	445.34
Colour	clear intense orange
Shape	block-shaped
Size/mm^3	0.25×0.12×0.10
T/K	139.99(10)
Crystal System	monoclinic
Space Group	$P2_1/m$
a/Å	6.45178(18)
b/Å	25.4553(5)
c/Å	7.4727(2)
α/°	90
β/°	106.175(3)
γ/°	90
V/Å^3	1178.68(6)
Z	2
Z'	0.5
Wavelength/Å	0.71073
Radiation type	Mo$K\alpha$
Θ_{min}/°	2.838
Θ_{max}/°	42.779
Measured Refl's.	20041
Indep't Refl's	6497
Refl's I≥2σ(I)	5152
R_{int}	0.0302
Parameters	203
Restraints	0
Largest Peak/e Å^{-3}	0.740
Deepest Hole/e Å^{-3}	-0.526
GooF	1.056
wR_2 (all data)	0.0871
wR_2	0.0830
R_1 (all data)	0.0470
R_1	0.0337
CCDC number	2418644

Rhodium(III) complex **Rh3**

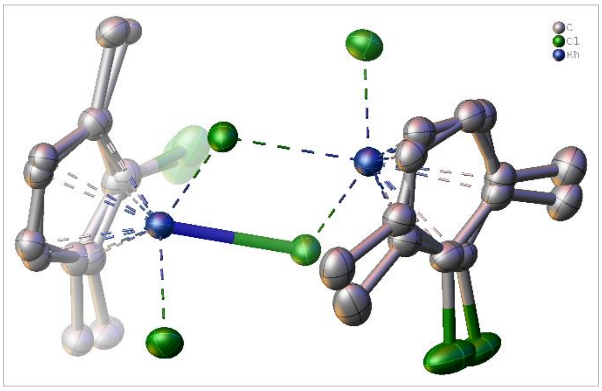

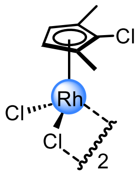

Rh3

Experimental. Single clear intense orange plate-shaped crystals of **Rh3** were used as supplied. A suitable crystal with dimensions 0.09 × 0.07 × 0.01 mm^3 was selected and mounted on a XtaLAB Synergy R, DW system, HyPix-Arc 150 diffractometer. The crystal was kept at a steady *T* = 139.99(10) K during data collection. The structure was solved with the ShelXT 2018/2 solution program[39] using dual methods and by using Olex2 1.5 as the graphical interface.[40] The model was refined with ShelXL 2019/3 using full matrix least squares minimisation on $\boldsymbol{F}^2$.[41]

Crystal Data. $C_{14}H_{16}Cl_6Rh_2$, M_r = 602.79, monoclinic, $P2_1/c$ (No. 14), a = 9.76278(18) Å, b = 13.1872(3) Å, c = 14.7147(3) Å, β = 104.459(2)°, $\alpha = \gamma$ = 90°, *V* = 1834.42(7) Å^3, *T* = 139.99(10) K, *Z* = 4, *Z'* = 1, μ(Cu K$_\alpha$) = 22.527, 18696 reflections measured, 3771 unique (R_{int} = 0.0415) which were used in all calculations. The final wR_2 was 0.0977 (all data) and R_1 was 0.0354 (I≥2 σ(I)).

**Compound**	**Rh3**
Formula	$C_{14}H_{16}Cl_6Rh_2$
$D_{calc.}$/ g cm^{-3}	2.183
μ/mm^{-1}	22.527
Formula Weight	602.79
Colour	clear intense orange
Shape	plate-shaped
Size/mm^3	0.09×0.07×0.01
T/K	139.99(10)
Crystal System	monoclinic
Space Group	$P2_1/c$
a/Å	9.76278(18)
b/Å	13.1872(3)
c/Å	14.7147(3)
α/°	90
β/°	104.459(2)
γ/°	90
V/Å^3	1834.42(7)
Z	4
Z'	1
Wavelength/Å	1.54184
Radiation type	Cu K$_\alpha$
Θ_{min}/°	4.569
Θ_{max}/°	75.609
Measured Refl's.	18696
Indep't Refl's	3771
Refl's I≥2 σ(I)	3280
R_{int}	0.0415
Parameters	316
Restraints	490
Largest Peak	1.784
Deepest Hole	-1.011
GooF	1.074
wR_2 (all data)	0.0977
wR_2	0.0947
R_1 (all data)	0.0415
R_1	0.0354
CCDC number	2479662

Rhodium(III) complex (±)-**Rh4**

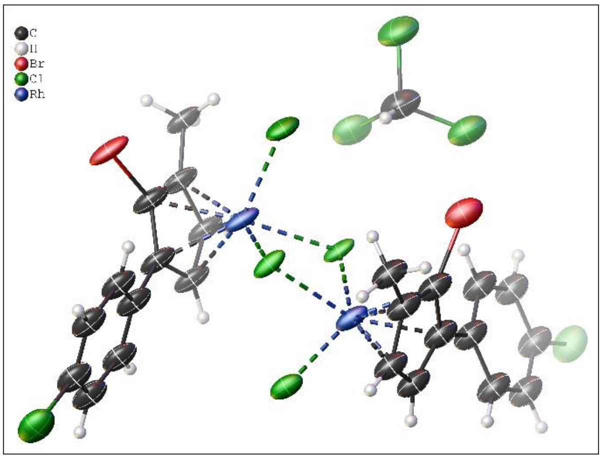

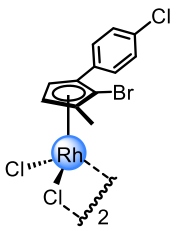

(±)-**Rh4**

Experimental. Single clear intense red plate-shaped crystals of **Rh4** were used as supplied. A suitable crystal with dimensions 0.21 × 0.14 × 0.02 mm³ was selected and mounted on a XtaLAB Synergy R, DW system, HyPix-Arc 150 diffractometer. The crystal was kept at a steady *T* = 140.00(10) K during data collection. The structure was solved with the ShelXT 2018/2 solution program[39] using dual methods and by using Olex2 1.5 as the graphical interface.[40] The model was refined with ShelXL 2019/3 using full matrix least squares minimisation on F^2.[41]

Crystal Data. $C_{27}H_{21}Br_2Cl_{15}Rh_2$, M_r = 1242.83, monoclinic, $P2_1/n$ (No. 14), a = 18.6813(18) Å, b = 11.9632(6) Å, c = 19.7185(17) Å, β = 114.773(11)°, α = γ = 90°, V = 4001.3(6) Å³, T = 140.00(10) K, Z = 4, Z' = 1, μ(Cu K$_\alpha$) = 18.418, 28253 reflections measured, 7696 unique (R_{int} = 0.0614) which were used in all calculations. The final wR_2 was 0.5710 (all data) and R_1 was 0.2266 (I≥2 σ(I)).

**Compound**	**Rh4**
Formula	$C_{27}H_{21}Br_2Cl_{15}Rh_2$
$D_{calc.}$/ g cm^{-3}	2.063
μ/mm^{-1}	18.418
Formula Weight	1242.83
Colour	clear intense red
Shape	plate-shaped
Size/mm³	0.21×0.14×0.02
T/K	140.00(10)
Crystal System	monoclinic
Space Group	$P2_1/n$
a/Å	18.6813(18)
b/Å	11.9632(6)
c/Å	19.7185(17)
α/°	90
β/°	114.773(11)
γ/°	90
V/Å³	4001.3(6)
Z	4
Z'	1
Wavelength/Å	1.54184
Radiation type	CuK$_\alpha$
Θ_{min}/°	2.737
Θ_{max}/°	75.422
Measured Refl's.	28253
Indep't Refl's	7696
Refl's I≥2 σ(I)	4828
R_{int}	0.0614
Parameters	303
Restraints	541
Largest Peak/e Å^{-3}	8.660
Deepest Hole/e Å^{-3}	-2.814
GooF	2.275
wR_2 (all data)	0.5710
wR_2	0.5457
R_1 (all data)	0.2589
R_1	0.2266
CCDC number	2479660

Rhodium(III) complex **Rh5**

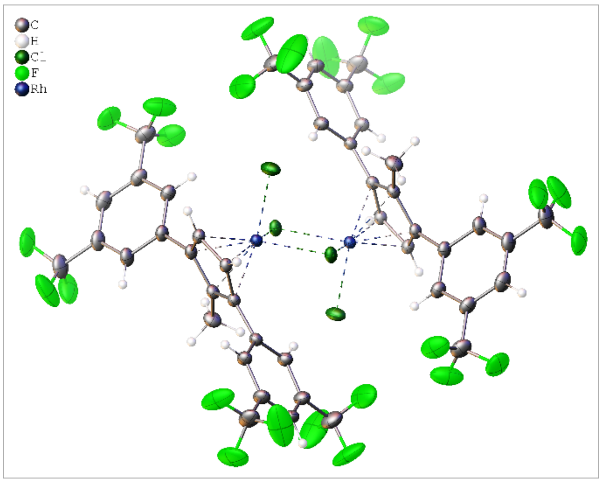

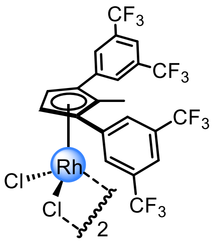

Rh5

Experimental. Single clear dark orange block-shaped crystals of **Rh5** were used as supplied. A suitable crystal with dimensions 0.17 × 0.12 × 0.10 mm³ was selected and mounted on a XtaLAB Synergy R, DW system, HyPix-Arc 150 diffractometer. The crystal was kept at a steady T = 140.00(10) K during data collection. The structure was solved with the ShelXT 2018/2 solution program[39] using dual methods and by using Olex2 1.5 as the graphical interface.[40] The model was refined with ShelXL 2019/3 using full matrix least squares minimisation on F^2.[41]

Crystal Data. $C_{44}H_{22}Cl_4F_{24}Rh_2$, M_r = 1354.23, triclinic, P-1 (No. 2), a = 8.58931(7) Å, b = 11.28419(8) Å, c = 12.57840(10) Å, α = 86.9950(6)°, β = 79.3779(7)°, γ = 86.7957(6)°, V = 1195.275(17) Å³, T = 140.00(10) K, Z = 1, Z' = 0.5, μ(Mo K$_\alpha$) = 1.041, 76577 reflections measured, 7294 unique (R_{int} = 0.0284) which were used in all calculations. The final wR_2 was 0.0880 (all data) and R_1 was 0.0315 (I≥2 σ(I)).

**Compound**	**Rh5**
Formula	$C_{44}H_{22}Cl_4F_{24}Rh_2$
$D_{calc.}$/ g cm^{-3}	1.881
μ/mm^{-1}	1.041
Formula Weight	1354.23
Colour	clear dark orange
Shape	block-shaped
Size/mm³	0.17×0.12×0.10
T/K	140.00(10)
Crystal System	triclinic
Space Group	P-1
a/Å	8.58931(7)
b/Å	11.28419(8)
c/Å	12.57840(10)
α/°	86.9950(6)
β/°	79.3779(7)
γ/°	86.7957(6)
V/Å³	1195.275(17)
Z	1
Z'	0.5
Wavelength/Å	0.71073
Radiation type	Mo K$_\alpha$
Θ_{min}/°	1.649
Θ_{max}/°	30.508
Measured Refl's.	76577
Indep't Refl's	7294
Refl's I≥2 σ(I)	6963
R_{int}	0.0284
Parameters	335
Restraints	72
Largest Peak	1.828
Deepest Hole	-0.751
GooF	1.060
wR_2 (all data)	0.0880
wR_2	0.0871
R_1 (all data)	0.0329
R_1	0.0315
CCDC number	2479664

Rhodium(III) complex **Rh6**

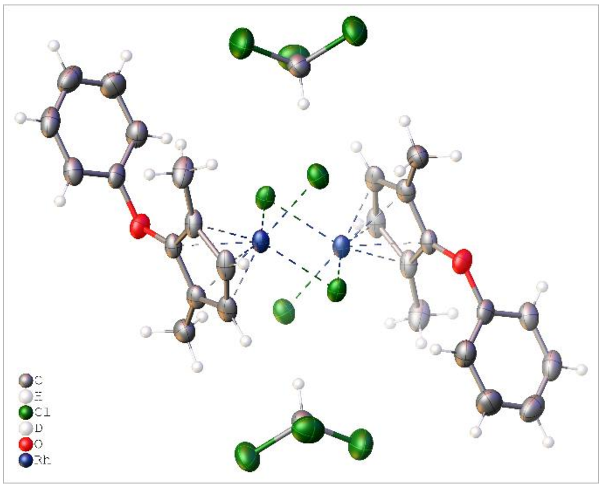

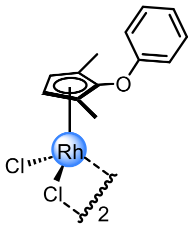

Rh6

Experimental. Single clear intense orange plate-shaped crystals of **Rh6** were used as supplied. A suitable crystal with dimensions 0.13 × 0.06 × 0.02 mm^3 was selected and mounted on a SuperNova, Dual, Cu at home/near, Atlas diffractometer. The crystal was kept at a steady T = 200.00(10) K during data collection. The structure was solved with the ShelXT 2018/2 solution program[39] using dual methods and by using Olex2 1.5 as the graphical interface.[40] The model was refined with ShelXL 2019/3 using full matrix least squares minimisation on $\boldsymbol{F}^2$.[41]

Crystal Data. $C_{28}H_{26}Cl_{10}D_2O_2Rh_2$, M_r = 958.83, monoclinic, $P2_1/n$ (No. 14), a = 12.3239(4) Å, b = 7.6057(2) Å, c = 18.9166(5) Å, β = 100.448(3)°, $\alpha = \gamma$ = 90°, V = 1743.70(9) Å^3, T = 200.00(10) K, Z = 2, Z' = 0.5, μ(CuK$_\alpha$) = 14.927, 9986 reflections measured, 3610 unique (R_{int} = 0.0329) which were used in all calculations. The final wR_2 was 0.0798 (all data) and R_1 was 0.0339 (I≥2 σ(I)).

**Compound**	**Rh6**
Formula	$C_{28}H_{26}Cl_{10}D_2O_2Rh_2$
$D_{calc.}$/ g cm^{-3}	1.826
μ/mm^{-1}	14.927
Formula Weight	958.83
Colour	clear intense orange
Shape	plate-shaped
Size/mm^3	0.13×0.06×0.02
T/K	200.00(10)
Crystal System	monoclinic
Space Group	$P2_1/n$
a/Å	12.3239(4)
b/Å	7.6057(2)
c/Å	18.9166(5)
α/°	90
β/°	100.448(3)
γ/°	90
V/Å^3	1743.70(9)
Z	2
Z'	0.5
Wavelength/Å	1.54184
Radiation type	CuK$_\alpha$
Θ_{min}/°	3.976
Θ_{max}/°	76.772
Measured Refl's.	9986
Indep't Refl's	3610
Refl's I≥2 σ(I)	3145
R_{int}	0.0329
Parameters	192
Restraints	0
Largest Peak	1.004
Deepest Hole	-0.455
GooF	1.169
wR_2 (all data)	0.0798
wR_2	0.0769
R_1 (all data)	0.0410
R_1	0.0339
CCDC number	2479665

Rhodium(III) complex **Rh7**

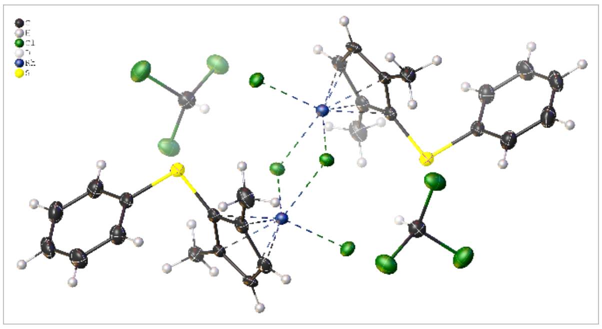

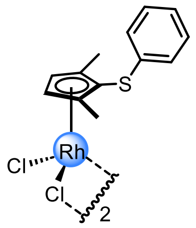

Rh7

Experimental. Single clear intense orange needle-shaped crystals of **Rh7** were used as supplied. A suitable crystal with dimensions 0.09 × 0.02 × 0.01 mm^3 was selected and mounted on a XtaLAB Synergy R, DW system, HyPix-Arc 150 diffractometer. The crystal was kept at a steady T = 139.99(10) K during data collection. The structure was solved with the ShelXT 2018/2 solution program[39] using dual methods and by using Olex2 1.5 as the graphical interface.[40] The model was refined with ShelXL 2019/3 using full matrix least squares minimisation on F^2.[41]

Crystal Data. $C_{28}H_{26}Cl_{10}D_2Rh_2S_2$, M_r = 990.95, orthorhombic, *Pbca* (No. 61), a = 9.27755(12) Å, b = 16.90152(16) Å, c = 23.1413(3) Å, $\alpha = \beta = \gamma$ = 90°, V = 3628.66(7) Å^3, T = 139.99(10) K, Z = 4, Z' = 0.5, μ(Cu K$_\alpha$) = 15.375, 31220 reflections measured, 3711 unique (R_{int} = 0.0274) which were used in all calculations. The final wR_2 was 0.0545 (all data) and R_1 was 0.0225 (I≥2 σ(I)).

**Compound**	**Rh7**
Formula	$C_{28}H_{26}Cl_{10}D_2Rh_2S_2$
$D_{calc.}$/ g cm^{-3}	1.814
μ/mm^{-1}	15.375
Formula Weight	990.95
Colour	clear intense orange
Shape	needle-shaped
Size/mm^3	0.09×0.02×0.01
T/K	139.99(10)
Crystal System	orthorhombic
Space Group	*Pbca*
a/Å	9.27755(12)
b/Å	16.90152(16)
c/Å	23.1413(3)
α/°	90
β/°	90
γ/°	90
V/Å^3	3628.66(7)
Z	4
Z'	0.5
Wavelength/Å	1.54184
Radiation type	Cu K$_\alpha$
Θ_{min}/°	3.820
Θ_{max}/°	75.654
Measured Refl's.	31220
Indep't Refl's	3711
Refl's I≥2 σ(I)	3297
R_{int}	0.0274
Parameters	197
Restraints	0
Largest Peak	0.407
Deepest Hole	-0.659
GooF	1.046
wR_2 (all data)	0.0545
wR_2	0.0528
R_1 (all data)	0.0278
R_1	0.0225
CCDC number	2479663

Rhodium(III) complex (±)-**Rh9**

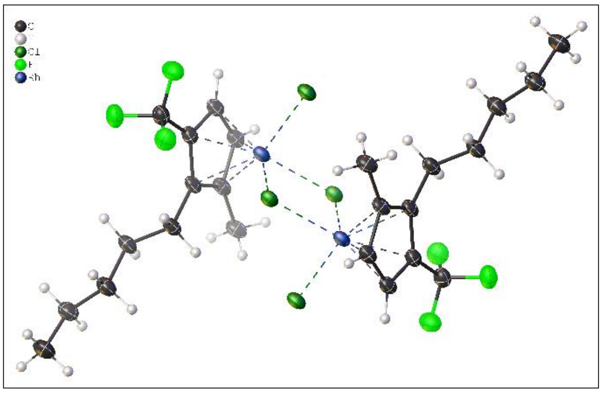

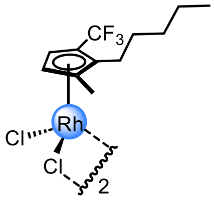

(±)-**Rh9**

Experimental. Single clear intense orange plate-shaped crystals of **Rh9** were used as supplied. A suitable crystal with dimensions 0.13 × 0.06 × 0.01 mm³ was selected and mounted on a XtaLAB Synergy R, DW system, HyPix-Arc 150 diffractometer. The crystal was kept at a steady *T* = 139.99(10) K during data collection. The structure was solved with the ShelXT 2018/2 solution program[39] using dual methods and by using Olex2 1.5 as the graphical interface.[40] The model was refined with ShelXL 2019/3 using full matrix least squares minimisation on ***F*²**.[41]

Crystal Data. $C_{24}H_{32}F_6Cl_4Rh_2$, M_r = 782.11, triclinic, *P*-1 (No. 2), a = 6.8897(4) Å, b = 9.0843(3) Å, c = 11.7349(7) Å, α = 77.837(4)°, β = 80.358(5)°, γ = 84.454(4)°, *V* = 706.40(6) Å³, *T* = 139.99(10) K, *Z* = 1, *Z'* = 0.5, μ(Cu K$_\alpha$) = 13.410, 9346 reflections measured, 2757 unique (R_{int} = 0.0329) which were used in all calculations. The final wR_2 was 0.1589 (all data) and R_1 was 0.0525 (I≥2 σ(I)).

**Compound**	**Rh9**
Formula	$C_{24}H_{32}F_6Cl_4Rh_2$
$D_{calc.}$/ g cm^{-3}	1.839
μ/mm^{-1}	13.410
Formula Weight	782.11
Colour	clear intense orange
Shape	plate-shaped
Size/mm³	0.13×0.06×0.01
T/K	139.99(10)
Crystal System	triclinic
Space Group	*P*-1
a/Å	6.8897(4)
b/Å	9.0843(3)
c/Å	11.7349(7)
α/°	77.837(4)
β/°	80.358(5)
γ/°	84.454(4)
V/Å³	706.40(6)
Z	1
Z'	0.5
Wavelength/Å	1.54184
Radiation type	Cu K$_\alpha$
Θ_{min}/°	3.898
Θ_{max}/°	74.344
Measured Refl's.	9346
Indep't Refl's	2757
Refl's I≥2 σ(I)	2463
R_{int}	0.0329
Parameters	165
Restraints	0
Largest Peak	1.164
Deepest Hole	-1.438
GooF	1.083
wR_2 (all data)	0.1589
wR_2	0.1559
R_1 (all data)	0.0573
R_1	0.0525
CCDC number	2479659

Rhodium(III) complex **Rh12’**

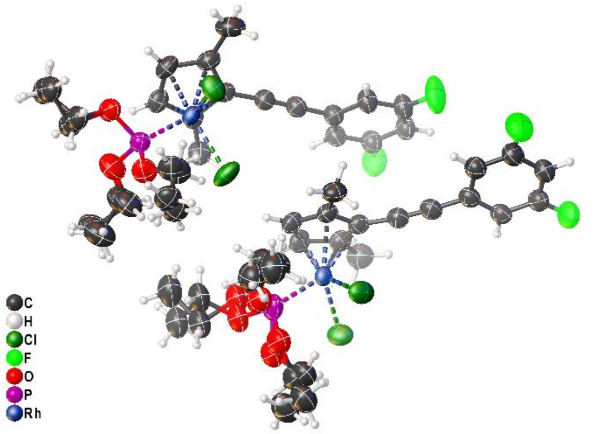

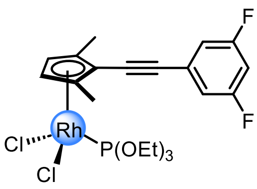

Rh12'

Experimental. Single clear intense orange plate-shaped crystals of **Rh12’** were used as supplied. A suitable crystal with dimensions 0.39 × 0.22 × 0.02 mm was selected and mounted on a XtaLAB Synergy R, DW system, HyPix-Arc 150 diffractometer. The crystal was kept at a steady *T* = 200.0(6) K during data collection. The structure was solved with the ShelXT 2018/2 solution program[39] using dual methods and by using Olex2 1.5 as the graphical interface.[40] The model was refined with ShelXL 2019/3 using full matrix least squares minimisation on F^2.[41]

Crystal Data. $C_{21}H_{26}Cl_2F_2O_3PRh$, M_r = 569.20, monoclinic, $P2_1/c$ (No. 14), a = 10.7618(3) Å, b = 27.0535(7) Å, c = 16.7963(4) Å, β = 91.724(2)°, $\alpha = \gamma$ = 90°, V = 4887.9(2) Å^3, T = 200.0(6) K, Z = 8, Z' = 2, μ(Mo K$_\alpha$) = 1.017, 69137 reflections measured, 14922 unique (R_{int} = 0.0904) which were used in all calculations. The final wR_2 was 0.1946 (all data) and R_1 was 0.0659 (I≥2σ(I)).

**Compound**	**Rh12’**
Formula	$C_{21}H_{26}Cl_2F_2O_3PRh$
$D_{calc.}$/ g cm^{-3}	1.547
μ/mm^{-1}	1.017
Formula Weight	569.20
Colour	clear intense orange
Shape	plate-shaped
Size/mm	0.39×0.22×0.02
T/K	200.0(6)
Crystal System	monoclinic
Space Group	$P2_1/c$
a/Å	10.7618(3)
b/Å	27.0535(7)
c/Å	16.7963(4)
α/°	90
β/°	91.724(2)
γ/°	90
V/Å^3	4887.9(2)
Z	8
Z'	2
Wavelength/Å	0.71073
Radiation type	Mo K$_\alpha$
Θ_{min}/°	1.933
Θ_{max}/°	30.508
Index range h	-15 ≤ h ≤ 12
Index range k	-38 ≤ k ≤ 38
Index range l	-23 ≤ l ≤ 23
Measured Refl's.	69137
Indep't Refl's	14922
Refl's I≥2σ(I)	10668
R_{int}	0.0904
Parameters	669
Restraints	1027
Largest Peak/eÅ^3	1.600
Deepest Hole/eÅ^3	-0.625
GooF	1.077
R_1 (I≥2σ(I) / all)	0.0659 / 0.0882
wR_2 (I≥2σ(I) / all)	0.1821 / 0.1946
CCDC number	2503537

8. References

[1] D. B. Ramachary, M. Kishor, *Org. Biomol. Chem.* **2008**, *6*, 4176–4187.

[2] L. D'Haenens, C. C. van de Sande, D. Tavernier, M. Vandewalle, *Bull. Soc. Chim. Belg.* **1986**, *95*, 273–281.

[3] G. Fraenkel, S. Boyd, A. Chow, J. Gallucci, *J. Am. Chem. Soc.* **1996**, *118*, 12804–12811.

[4] H. Liang, A. Schulé, J.-P. Vors, M. A. Ciufolini, *Org. Lett.* **2007**, *9*, 4119–4122.

[5] M. L. Quesada, R. H. Schlessinger, W. H. Parsons, *J. Org. Chem.* **1978**, *43*, 3968–3970.

[6] S. J. Alward, A. G. Fallis, *Can. J. Chem.* **1984**, *62*, 121–127.

[7] T. Fujita, S. Watanabe, K. Suga, T. Inaba, *J. Chem. Technol. Biotechnol.* **1979**, *29*, 100–106.

[8] B. Yeoul Lee, J. Wook Han, Y. Keun Chung, S. W. Lee, *J. Organomet. Chem.* **1999**, *587*, 181–190.

[9] J. Mathew, *J. Org. Chem.* **1990**, *55*, 5294–5297.

[10] K. Antczak, J. F. Kingston, S. J. Alward, A. G. Fallis, *Can. J. Chem.* **1984**, *62*, 829–837.

[11] H. Yu, C. Wan, J. Han, A. Li, *Acta Chim. Sin.* **2013**, *71*, 1488.

[12] P. Qu, S. A. Snyder, *J. Am. Chem. Soc.* **2021**, *143*, 11951–11956.

[13] C. R. Johnson, J. P. Adams, M. P. Braun, C. B. W. Senanayake, P. M. Wovkulich, M. R. Uskoković, *Tetrahedron Lett.* **1992**, *33*, 917–918.

[14] T. Takahashi, Z. Xi, Y. Nishihara, S. Huo, K. Kasai, K. Aoyagi, V. Denisov, E. Negishi, *Tetrahedron* **1997**, *53*, 9123–9134.

[15] H. Stetter, G. Lorenz, *Chem. Ber.* **1985**, *118*, 1115–1125.

[16] K. Itami, K. Mitsudo, K. Fujita, Y. Ohashi, J. I. Yoshida, *J. Am. Chem. Soc.* **2004**, *126*, 11058–11066.

[17] P. A. Wender, D. Staveness, *Org. Lett.* **2014**, *16*, 5140–5143.

[18] J. Krieger, G. Ricci, D. Lesuisse, C. Meyer, J. Cossy, *Angew. Chem. Int. Ed.* **2014**, *53*, 8705–8708.

[19] N. Münster, N. A. Parker, L. van Dijk, R. S. Paton, M. D. Smith, *Angew. Chem. Int. Ed.* **2017**, *56*, 9468–9472.

[20] A. G. Montalban, L.-O. Wittenberg, A. McKillop, *Tetrahedron Lett.* **1999**, *40*, 5893–5896.

[21] A. A. Ponaras, O. Zaim, *J. Org. Chem.* **1987**, *52*, 5630–5633.

[22] K. Matoba, M. Tokizawa, T. Morita, T. Yamazaki, *Chem. Pharm. Bull.* **1985**, *33*, 368–372.

[23] M. Kõllo, K. Rõuk, I. Järving, T. Pehk, M. Lopp, *Tetrahedron* **2023**, *136*, 133363.

[24] R. Broussier, S. Ninoreille, C. Legrand, B. Gautheron, *J. Organomet. Chem.* **1997**, *532*, 55–60.

[25] J. R. Sowa, R. J. Angelici, *J. Am. Chem. Soc.* **1991**, *113*, 2537–2544.

[26] G. Carr, C. Dean, D. Whittaker, *J. Chem. Soc. Perkin Trans. 2* **1989**, *71*, 71–76.

[27] B. Y. Lee, H. Moon, Y. K. Chung, N. Jeong, *J. Am. Chem. Soc.* **1994**, *116*, 2163–2164.

[28] A. Cleve, U. Klar, W. Schwede, *J. Fluor. Chem.* **2005**, *126*, 217–220.

[29] T. Zeng, Y. Li, R. Wang, J. Zhu, *Org. Lett.* **2024**, *26*, 3413–3418.

[30] L. E. Manzer, *J. Am. Chem. Soc.* **1978**, *100*, 8068–8073.

[31] X. Li, M. Nishiura, K. Mori, T. Mashiko, Z. Hou, *Chem. Commun.* **2007**, 4137.

[32] W. Lin, W. Li, D. Lu, F. Su, T.-B. Wen, H.-J. Zhang, *ACS Catal.* **2018**, *8*, 8070–8076.

[33] T. Piou, F. Romanov-Michailidis, M. Romanova-Michaelides, K. E. Jackson, N. Semakul, T. D. Taggart, B. S. Newell, C. D. Rithner, R. S. Paton, T. Rovis, *J. Am. Chem. Soc.* **2017**, *139*, 1296–1310.

[34] T. Kang, Y. Kim, D. Lee, Z. Wang, S. Chang, *J. Am. Chem. Soc.* **2014**, *136*, 4141–4144.

[35] M. Feurer, G. Frey, H.-T. Luu, D. Kratzert, J. Streuff, *Chem. Commun.* **2014**, *50*, 5370–5372.

[36] M. Font, B. Cendón, A. Seoane, J. L. Mascareñas, M. Gulías, *Angew. Chem. Int. Ed.* **2018**, *57*, 8255–8259.

[37] T. K. Hyster, T. Rovis, *Chem. Commun.* **2011**, *47*, 11846–11848.

[38] X. Yu, K. Chen, Q. Wang, W. Zhang, J. Zhu, *Org. Chem. Front.* **2018**, *5*, 994–997.

[39] G. M. Sheldrick, *Acta Crystallogr. Sect. A Found. Adv.* **2015**, *71*, 3–8.

[40] O. V. Dolomanov, L. J. Bourhis, R. J. Gildea, J. A. K. Howard, H. Puschmann, *J. Appl. Crystallogr.* **2009**, *42*, 339–341.

[41] G. M. Sheldrick, *Acta Crystallogr. Sect. C Struct. Chem.* **2015**, *71*, 3–8.

9. NMR spectra

Copies of the ^{1}H NMR, $^{13}C\{^{1}H\}$ NMR, $^{19}F\{^{1}H\}$ NMR, and $^{31}P\{^{1}H\}$ NMR spectra of new unpublished compounds (or compounds of which the published NMR data was incomplete) are provided with indication of the resonance frequency and the employed deuterated solvent. All spectra were recorded at 298 K, unless otherwise indicated.

Copies of the 2D NOESY spectra of cyclopentadienes **Cp28**, **Cp30-31**, and **Cp33-35** are also provided.

Copies of the IR spectra of complexes **Co1** and **Co2** are provided as well.

A copy of the chiral HPLC spectrum of racemic complex (±)-**Rh17’** is also provided.

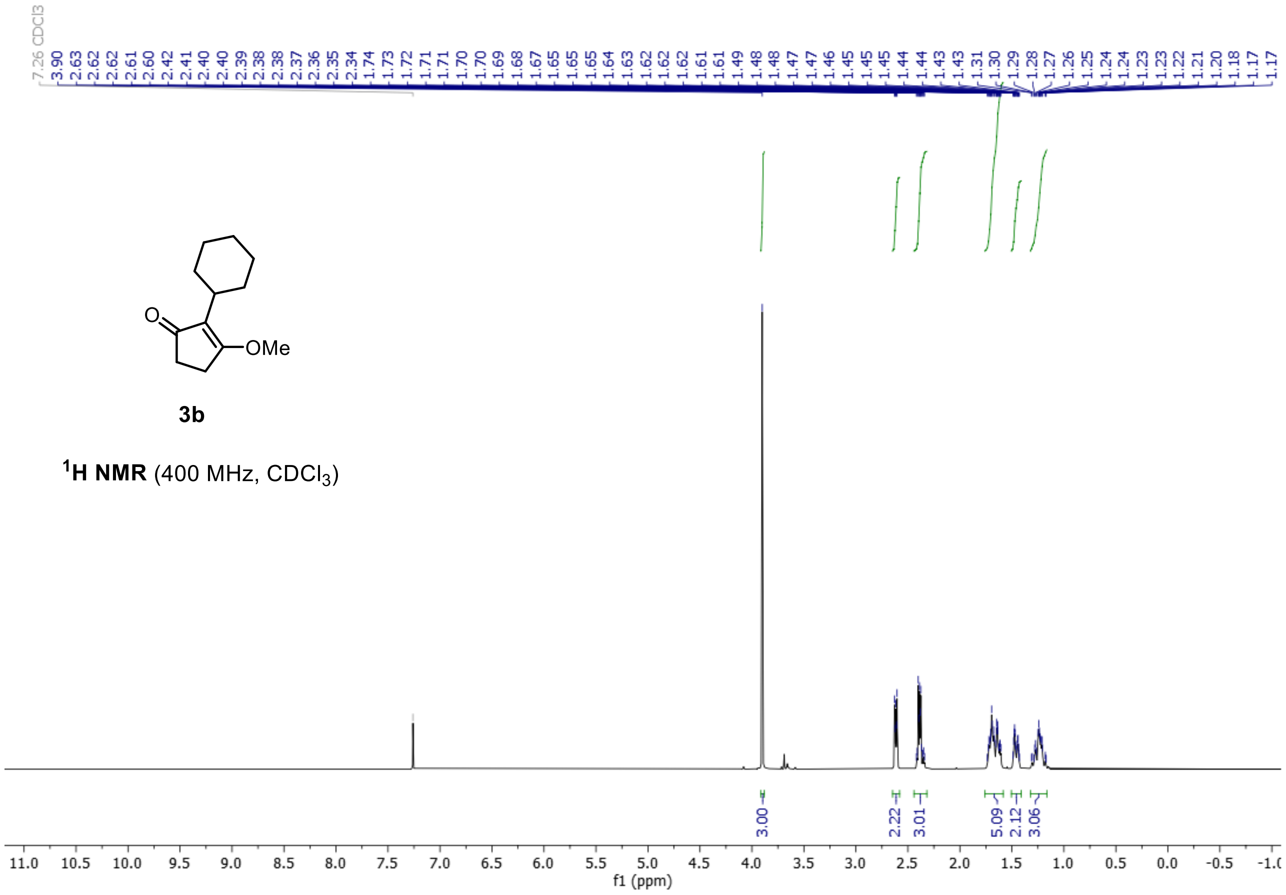
7.26 CDCl3
3.90
2.63
2.62
2.62
2.61
2.60
2.42
2.41
2.40
2.40
2.39
2.38
2.38
2.37
2.36
2.35
2.34
1.74
1.73
1.72
1.71
1.71
1.70
1.69
1.68
1.67
1.65
1.65
1.65
1.64
1.63
1.62
1.62
1.62
1.61
1.61
1.49
1.48
1.48
1.47
1.47
1.46
1.45
1.45
1.45
1.44
1.44
1.43
1.43
1.31
1.30
1.29
1.28
1.27
1.26
1.25
1.24
1.24
1.23
1.23
1.22
1.21
1.20
1.18
1.17
1.17
O
OMe
3b
1H NMR (400 MHz, CDCl3)
3.00
2.22
3.01
5.09
2.12
3.06
f1 (ppm)

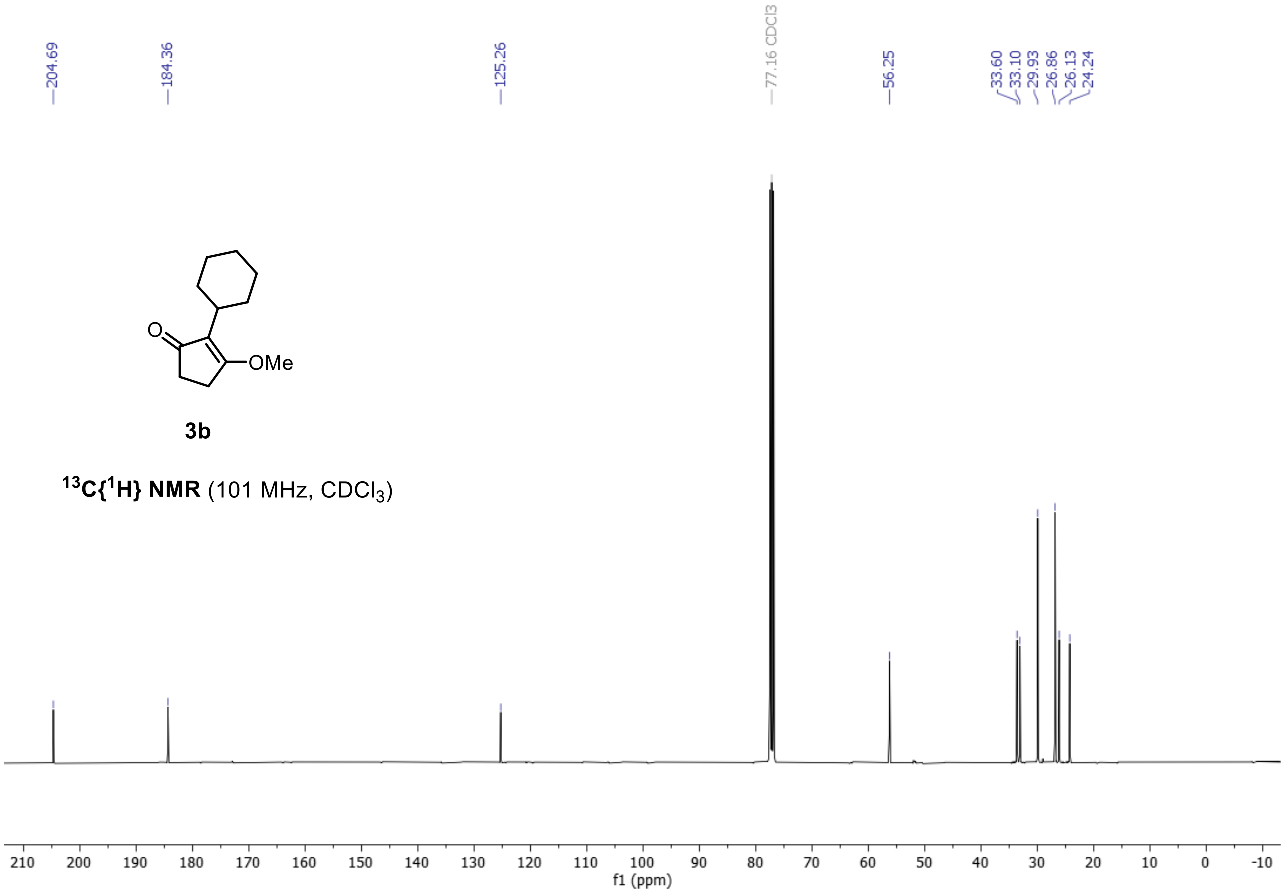
204.69
184.36
125.26
77.16 CDCl3
56.25
33.60
33.10
29.93
26.86
26.13
24.24
O
OMe
3b
13C{1H} NMR (101 MHz, CDCl3)
f1 (ppm)

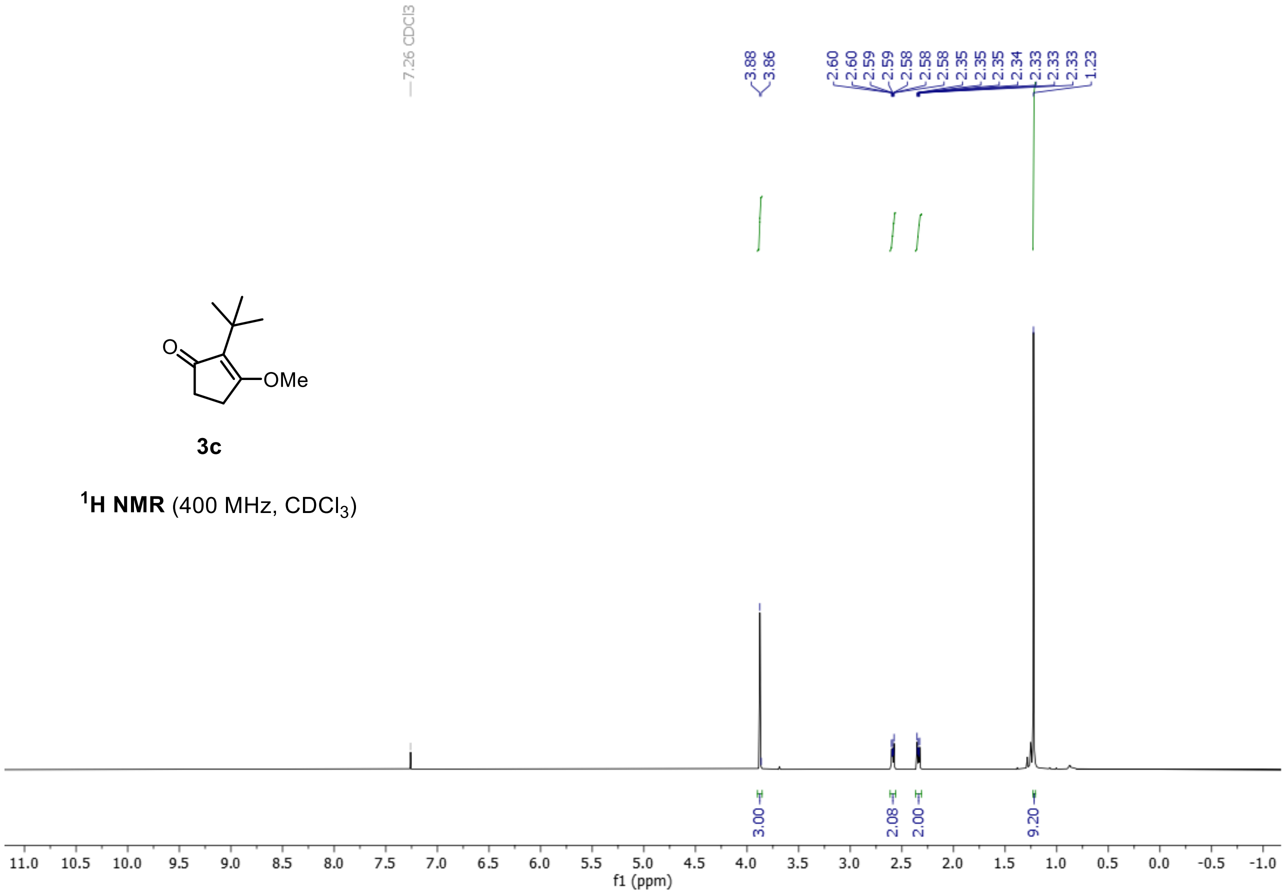
3c
1H NMR (400 MHz, CDCl3)
OMe
O
f1 (ppm)

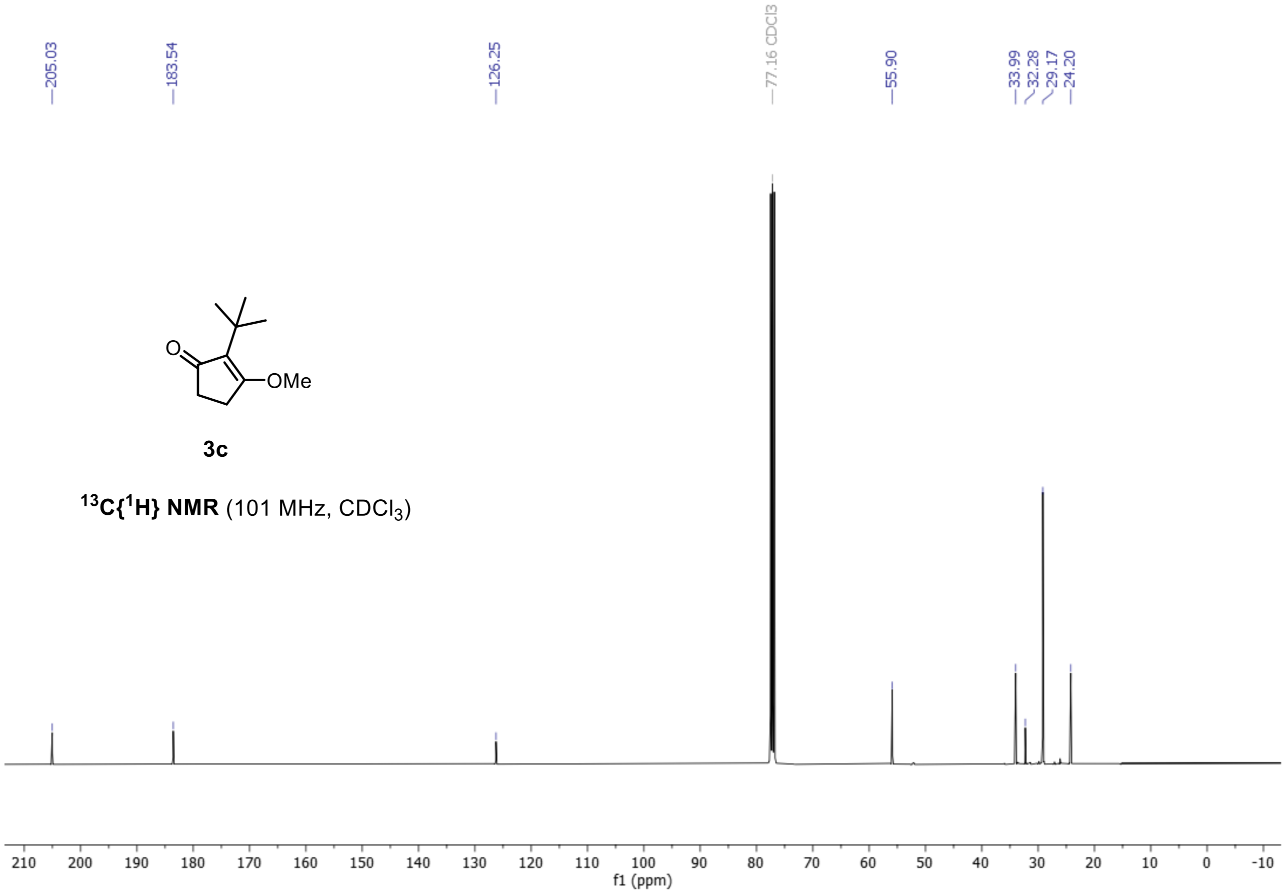
3c
13C{1H} NMR (101 MHz, CDCl3)
OMe
O
f1 (ppm)

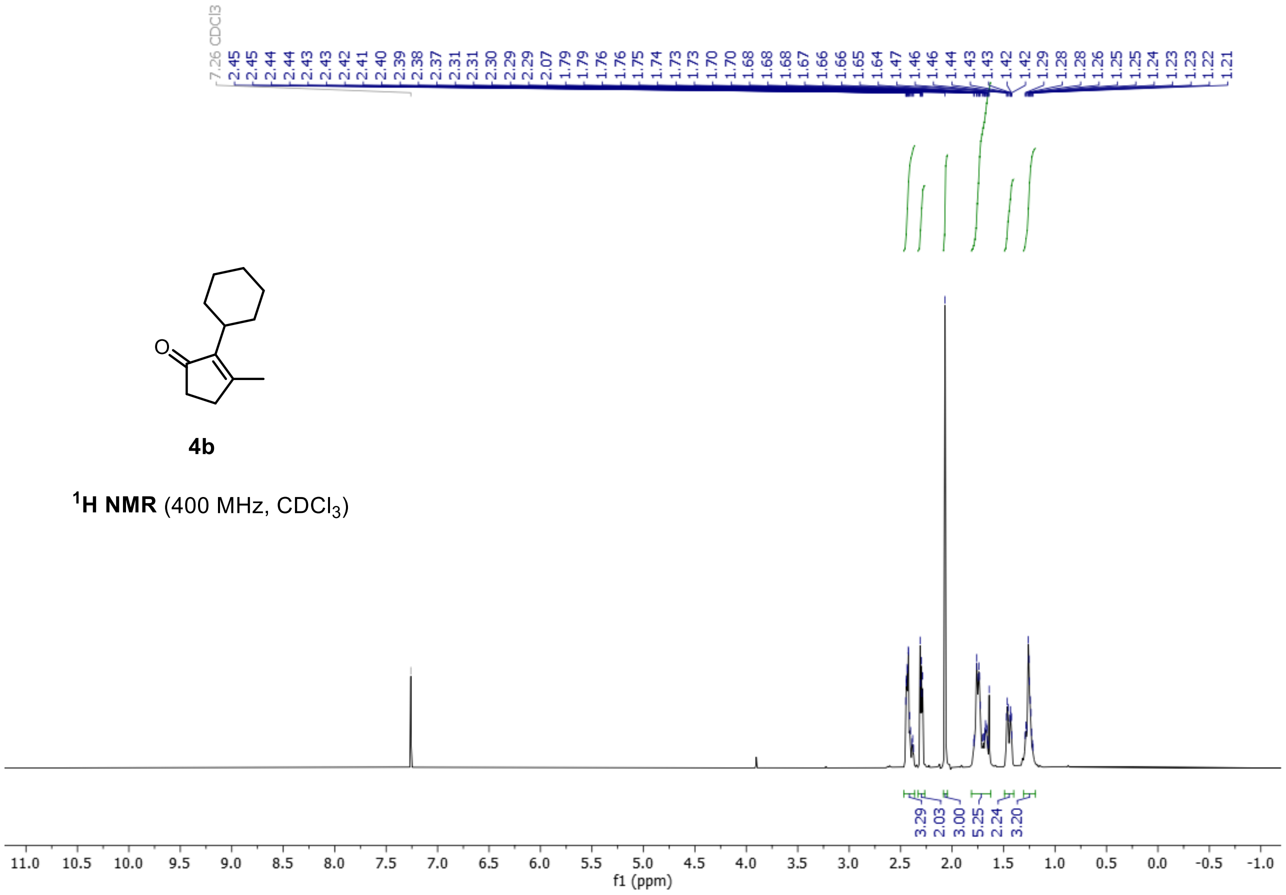

4b

^{1}H NMR (400 MHz, $CDCl_3$)

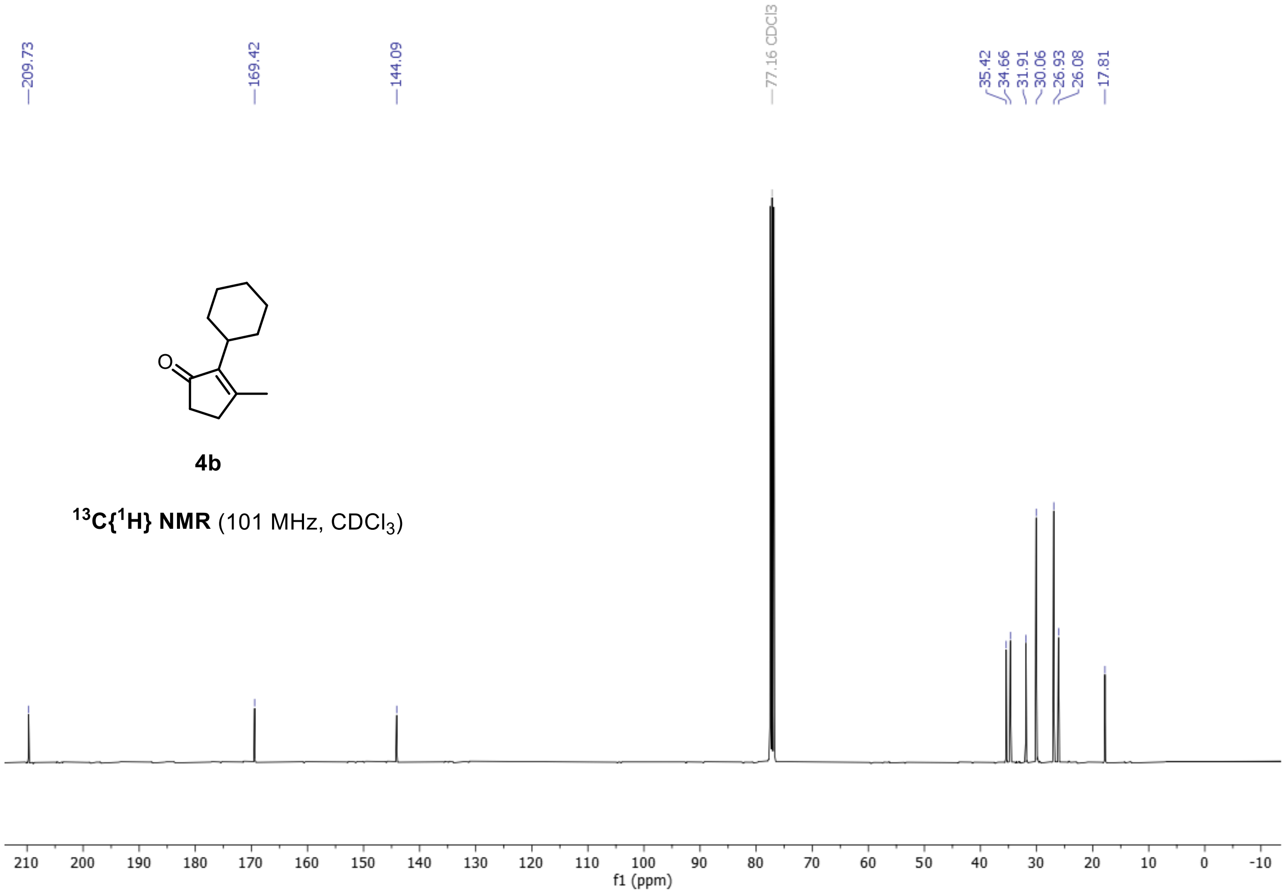

4b

^{13}C{^{1}H} NMR (101 MHz, $CDCl_3$)

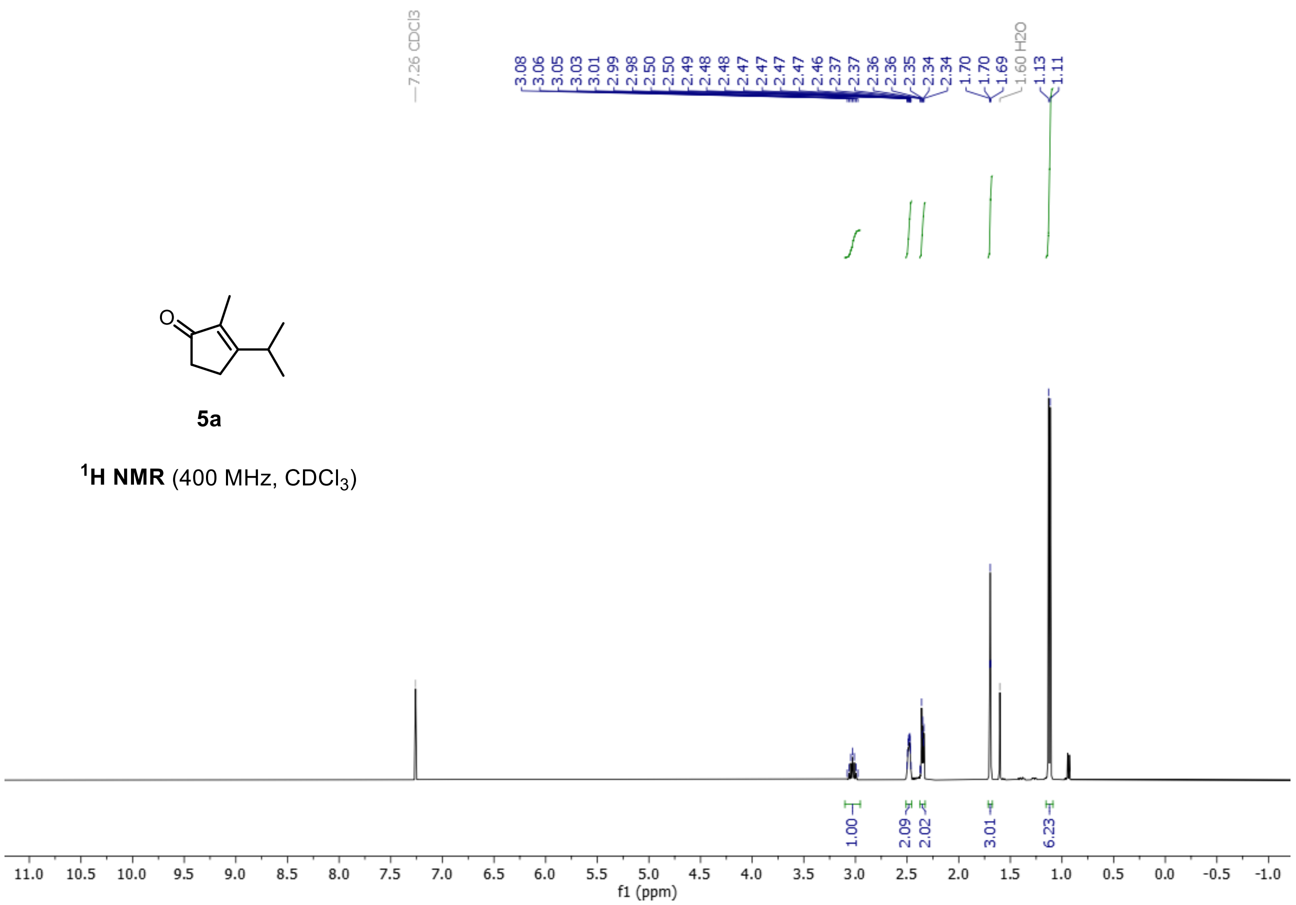

7.26 CDCl3
3.08
3.06
3.05
3.03
3.01
2.99
2.98
2.50
2.50
2.49
2.48
2.47
2.47
2.47
2.47
2.46
2.37
2.37
2.36
2.36
2.35
2.34
2.34
1.70
1.70
1.69
1.60 H2O
1.13
1.11
O
5a
1H NMR (400 MHz, CDCl3)
1.00
2.09
2.02
3.01
6.23
11.0 10.5 10.0 9.5 9.0 8.5 8.0 7.5 7.0 6.5 6.0 5.5 5.0 4.5 4.0 3.5 3.0 2.5 2.0 1.5 1.0 0.5 0.0 -0.5 -1.0
f1 (ppm)

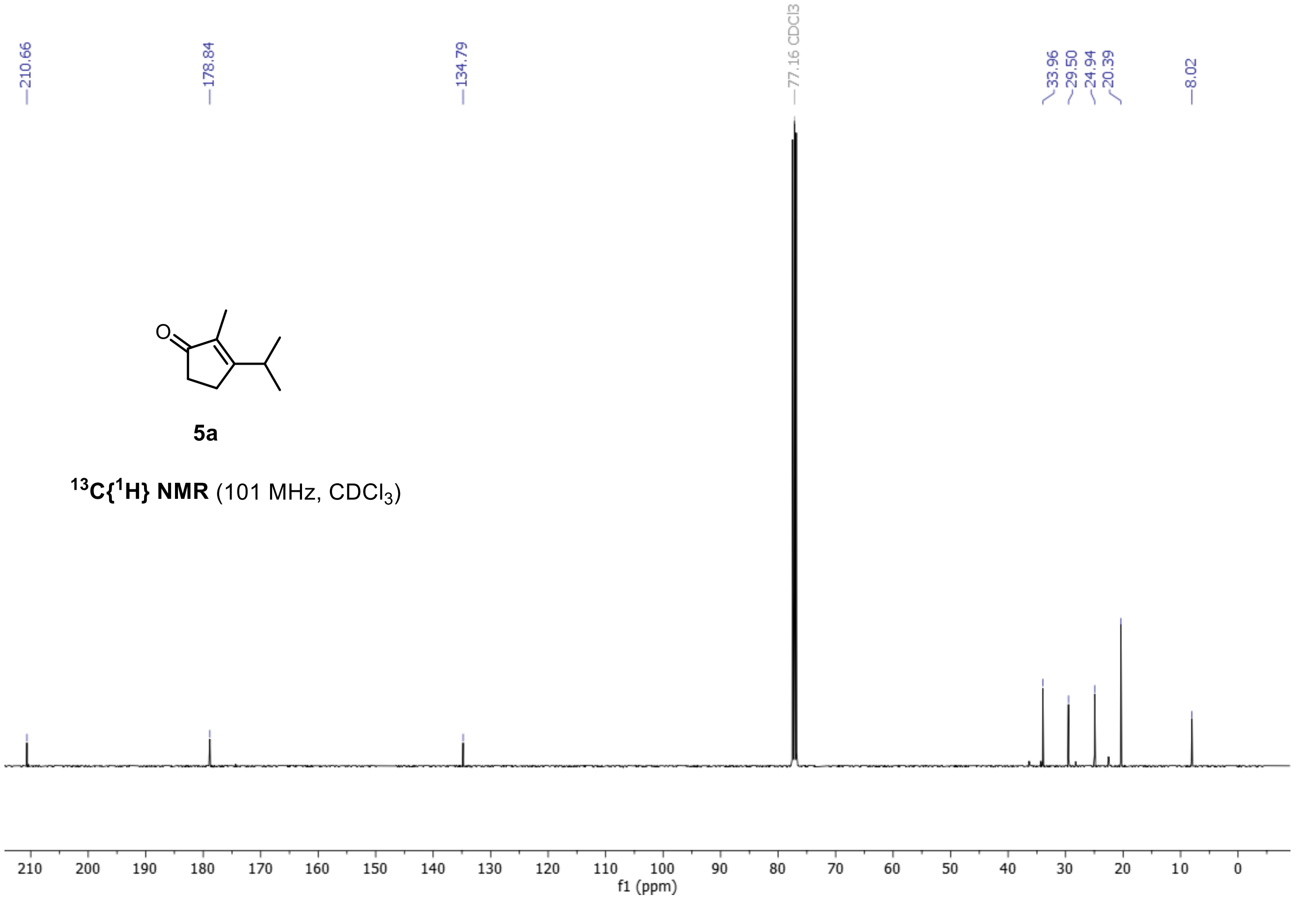

210.66
178.84
134.79
77.16 CDCl3
33.96
29.50
24.94
20.39
8.02
O
5a
13C{1H} NMR (101 MHz, CDCl3)
210 200 190 180 170 160 150 140 130 120 110 100 90 80 70 60 50 40 30 20 10 0
f1 (ppm)

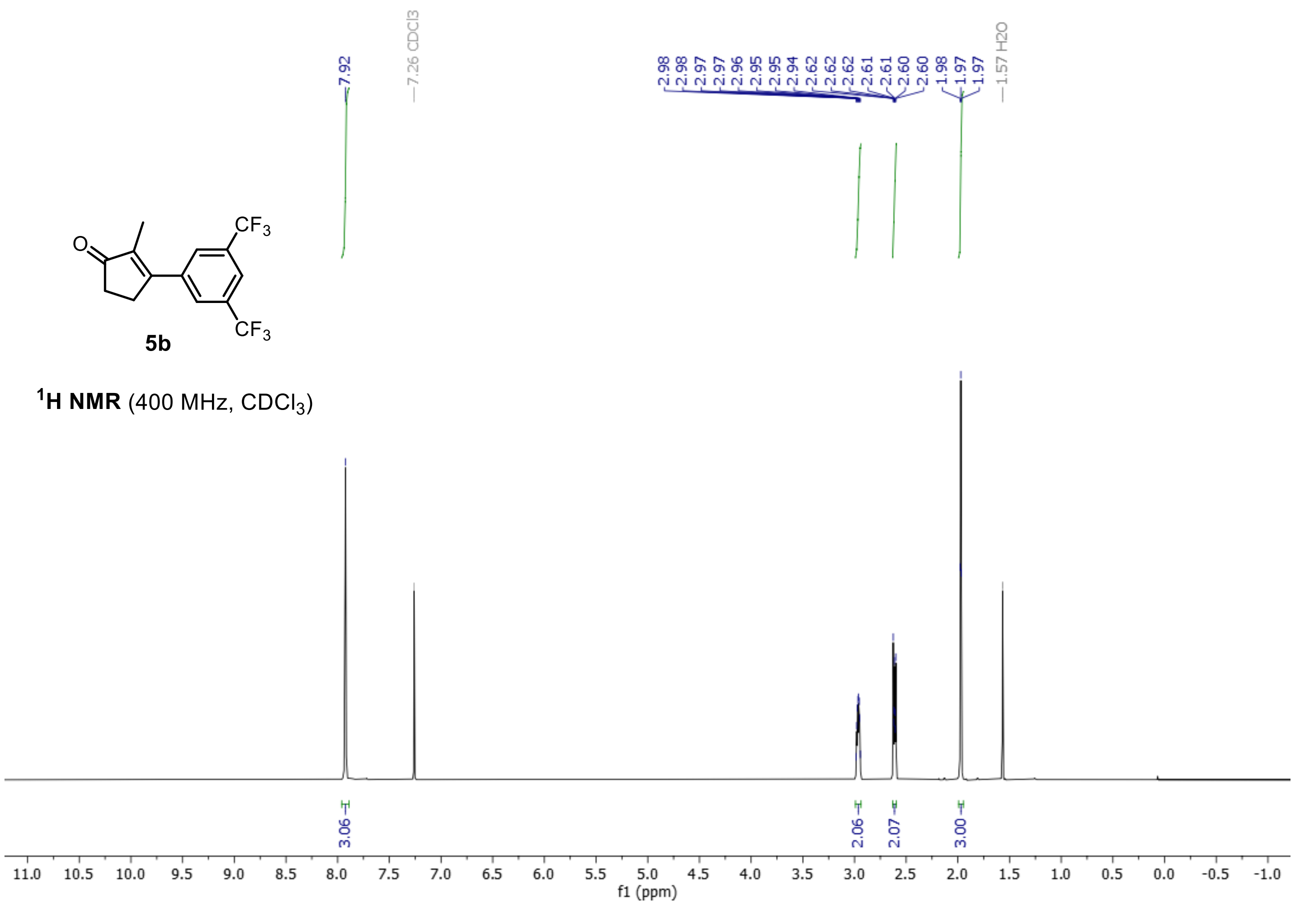

7.92
7.26 CDCl3
2.98 2.98 2.97 2.97 2.96 2.95 2.95 2.94 2.62 2.62 2.61 2.61 2.60 2.60 1.98 1.97 1.97
1.57 H2O
CF3
O
CF3
5b
1H NMR (400 MHz, CDCl3)
3.06
2.06
2.07
3.00
11.0 10.5 10.0 9.5 9.0 8.5 8.0 7.5 7.0 6.5 6.0 5.5 5.0 4.5 4.0 3.5 3.0 2.5 2.0 1.5 1.0 0.5 0.0 -0.5 -1.0
f1 (ppm)

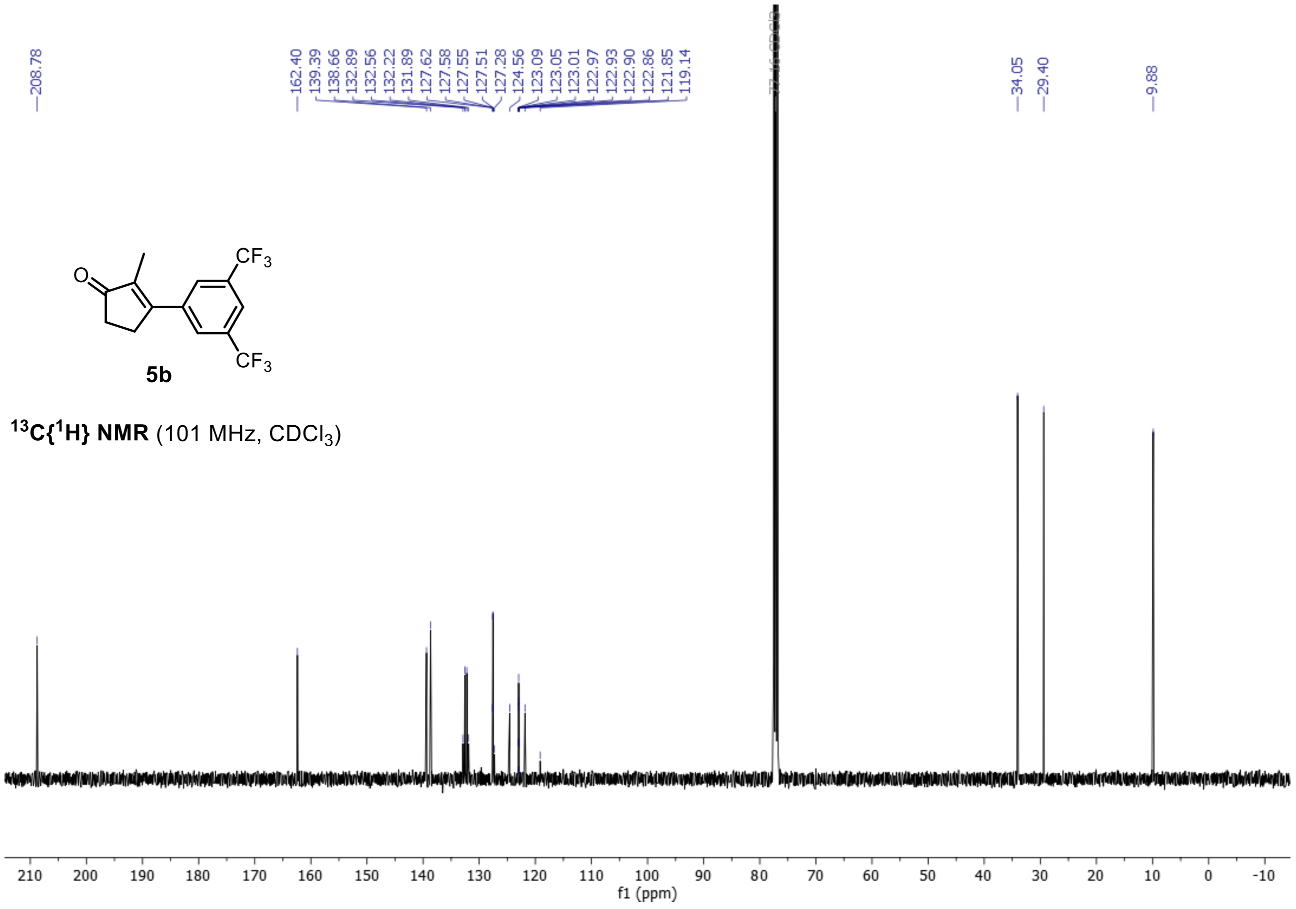

208.78
162.40 139.39 138.66 132.89 132.56 132.22 131.89 127.62 127.58 127.55 127.51 127.28 124.56 123.09 123.05 123.01 122.97 122.93 122.90 122.86 121.85 119.14
34.05
29.40
9.88
CF3
O
CF3
5b
13C{1H} NMR (101 MHz, CDCl3)
210 200 190 180 170 160 150 140 130 120 110 100 90 80 70 60 50 40 30 20 10 0 -10
f1 (ppm)

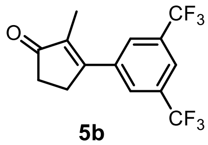

5b

$^{19}F\{^1H\}$ NMR (376 MHz, $CDCl_3$)

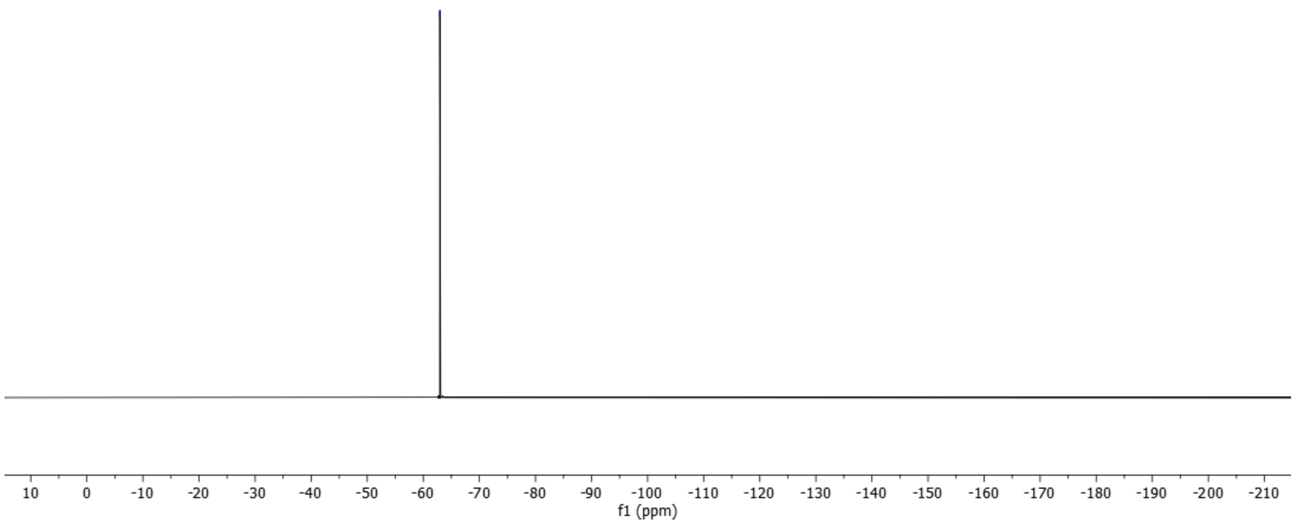

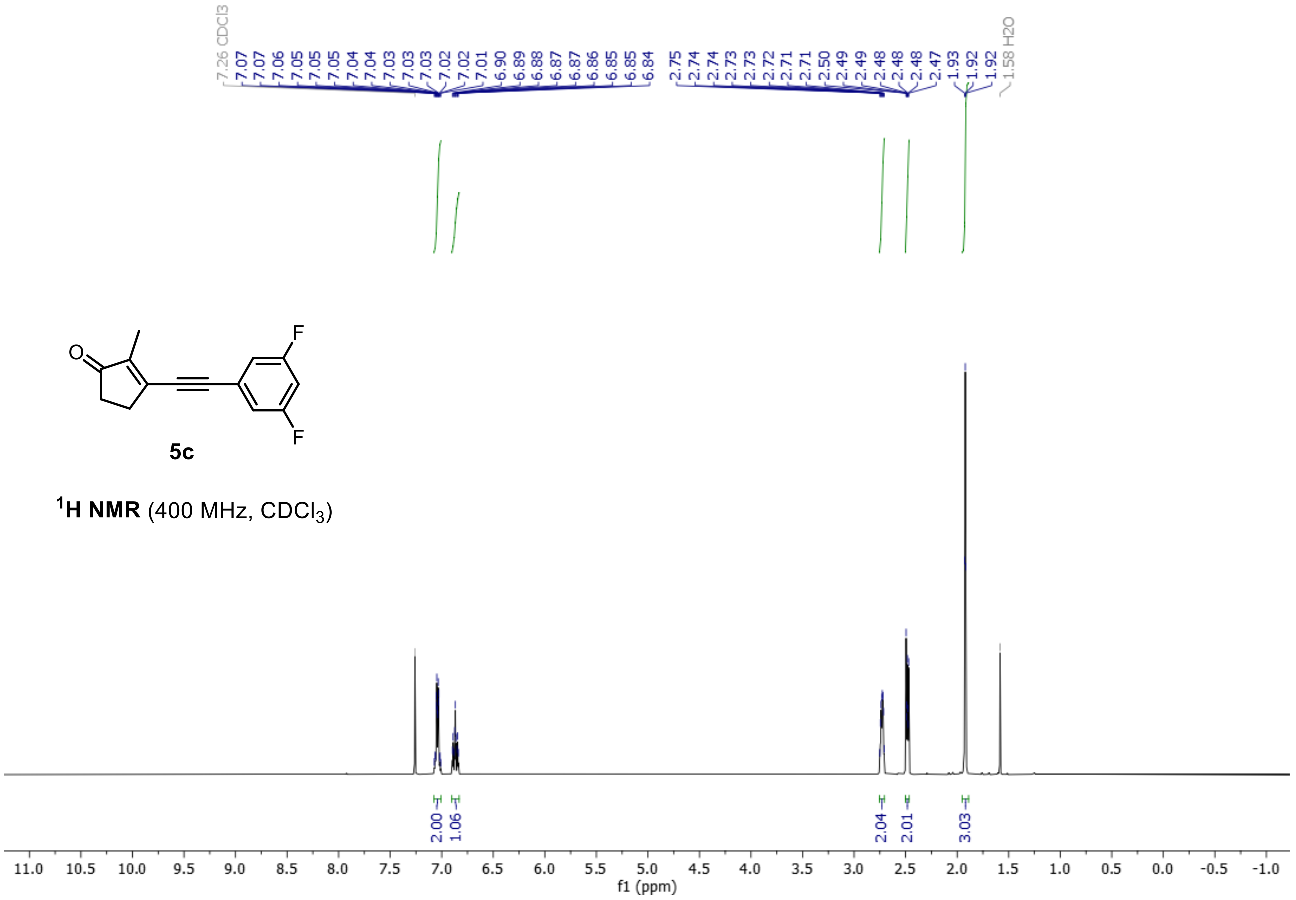
5c
^{1}H NMR (400 MHz, CDCl$_3$)
f1 (ppm)

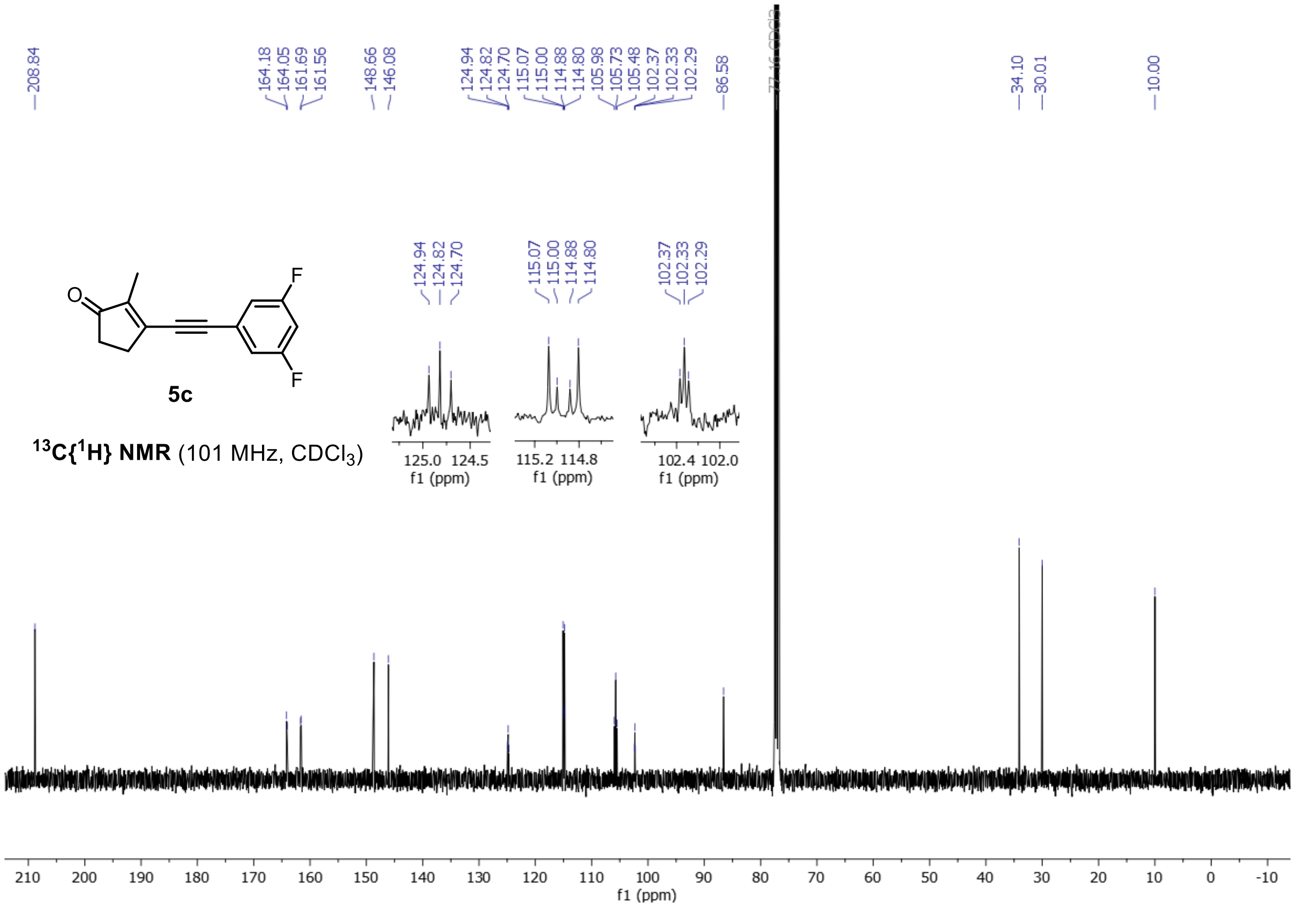
5c
^{13}C{^{1}H} NMR (101 MHz, CDCl$_3$)
f1 (ppm)

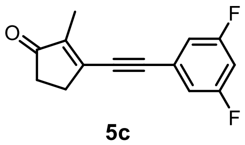

5c

$^{19}F\{^{1}H\}$ NMR (376 MHz, $CDCl_3$)

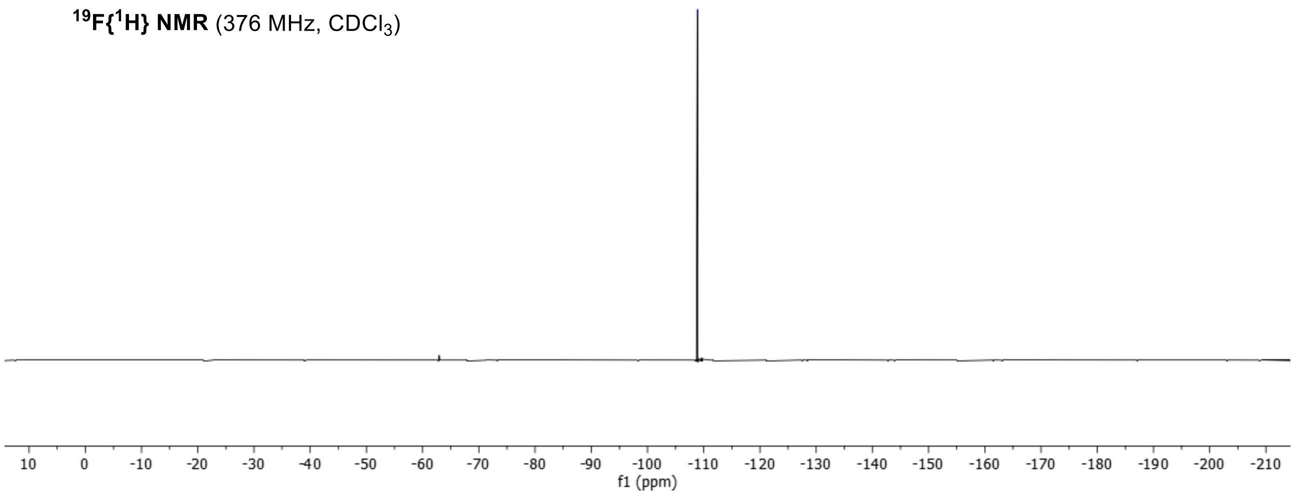

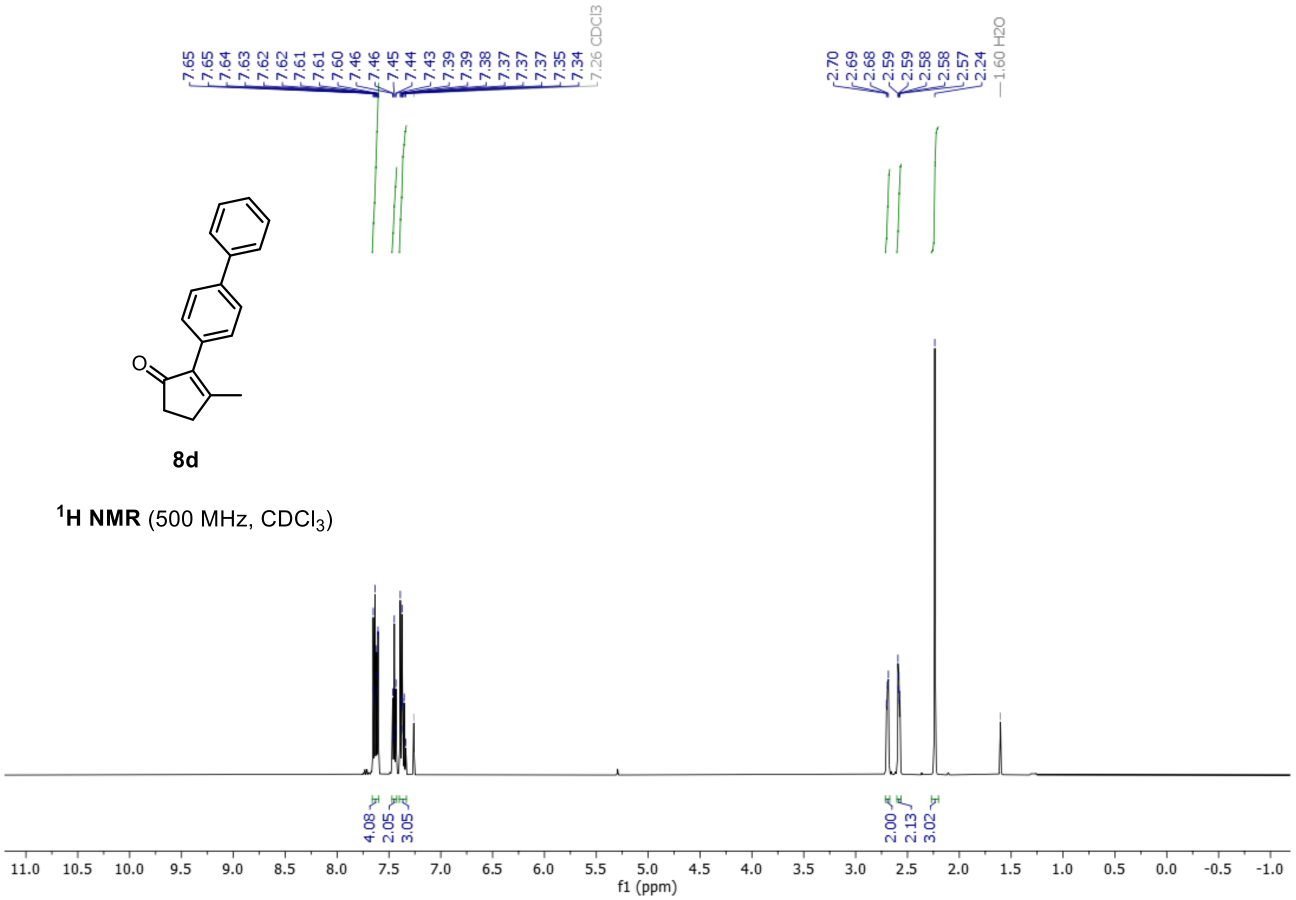
8d
^{1}H NMR (500 MHz, $CDCl_3$)
7.65 7.65 7.64 7.63 7.62 7.62 7.61 7.61 7.60 7.46 7.46 7.45 7.44 7.43 7.39 7.39 7.38 7.37 7.37 7.37 7.35 7.34 7.26 CDCl3
2.70 2.69 2.68 2.59 2.59 2.58 2.58 2.57 2.24 1.60 H2O
4.08 2.05 3.05 2.00 2.13 3.02
f1 (ppm)

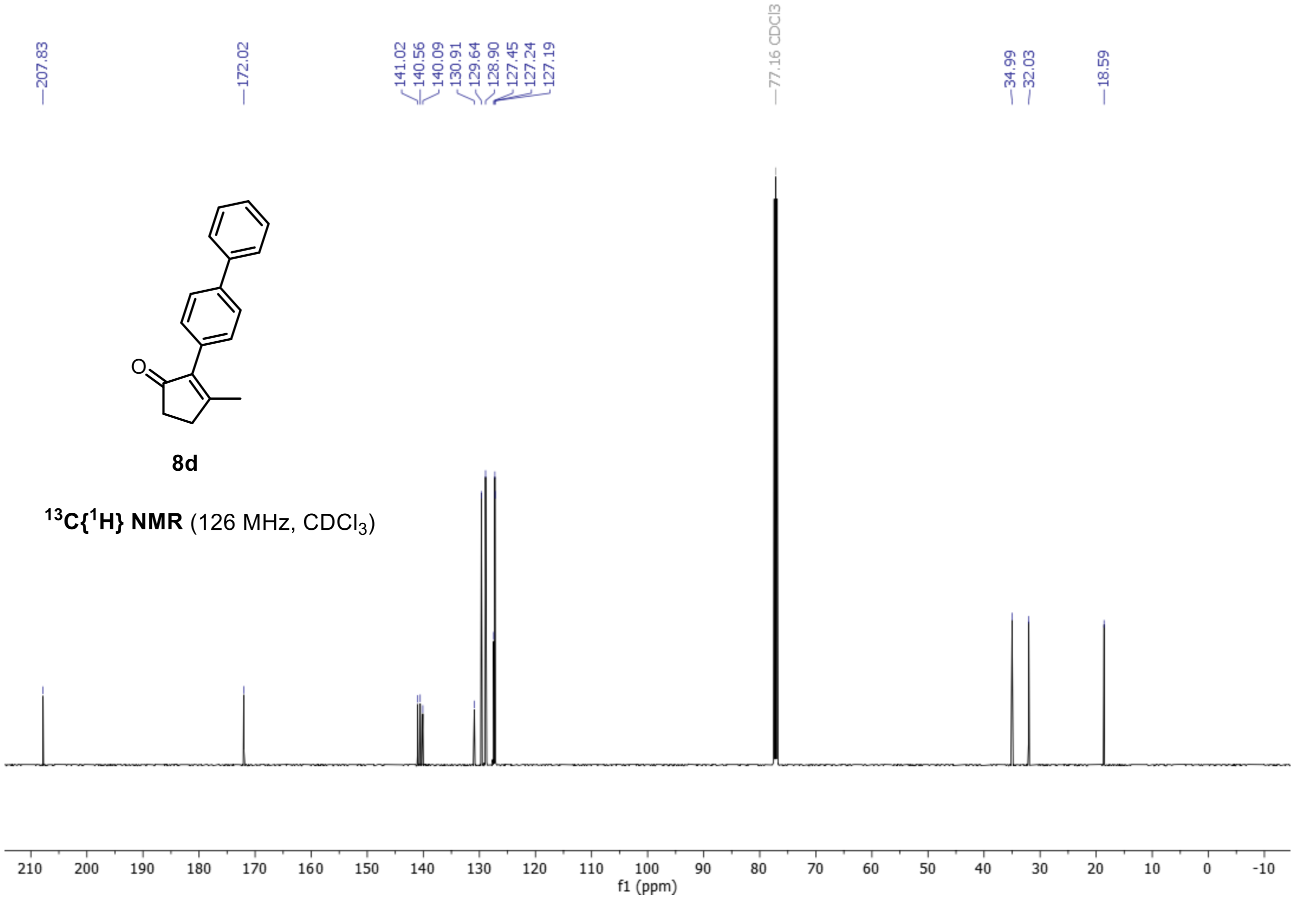
8d
^{13}C{^{1}H} NMR (126 MHz, $CDCl_3$)
207.83 172.02 141.02 140.56 140.09 130.91 129.64 128.90 127.45 127.24 127.19 77.16 CDCl3 34.99 32.03 18.59
f1 (ppm)

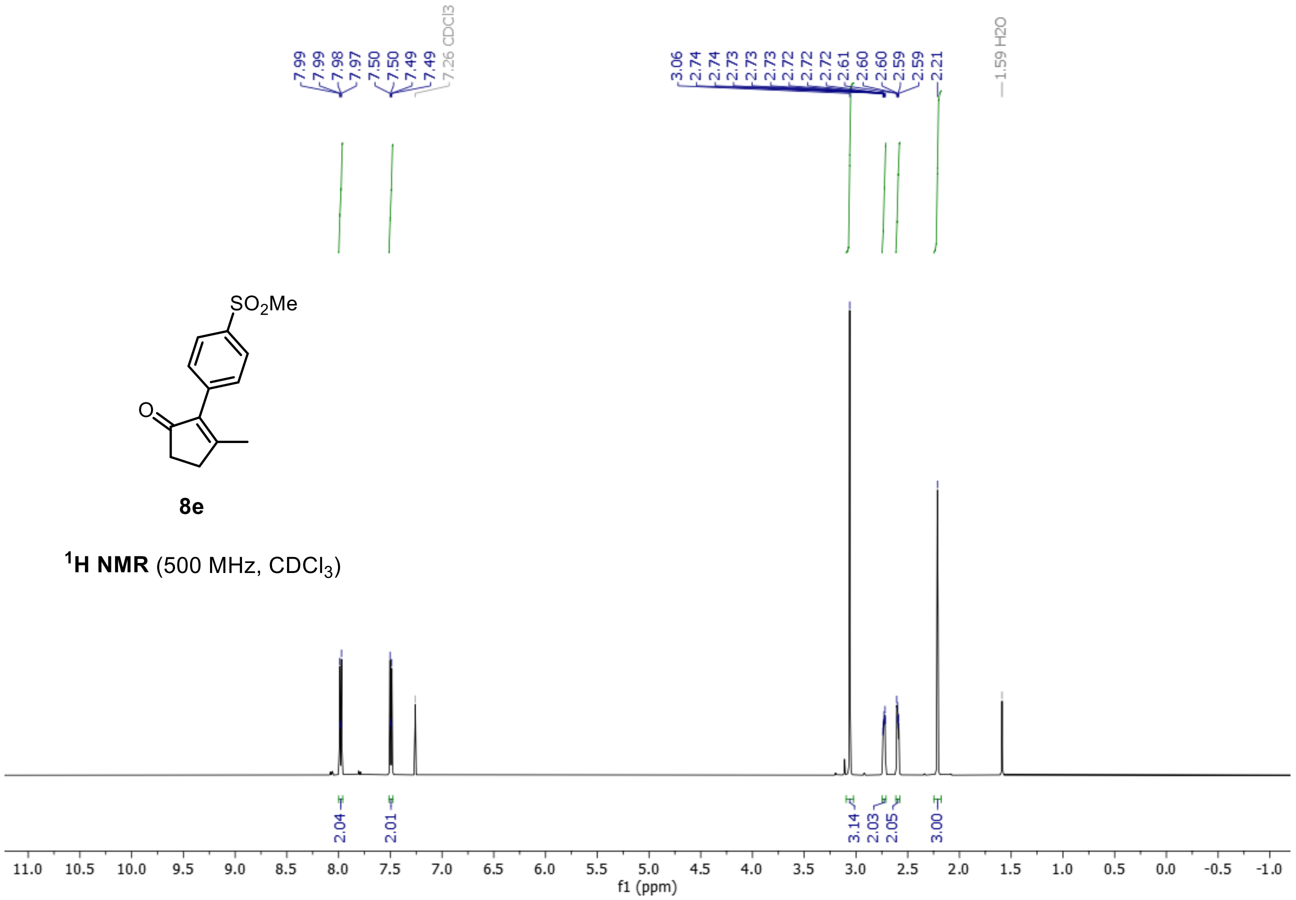
7.99
7.99
7.98
7.97
7.50
7.50
7.49
7.49
7.26 CDCl3
3.06
2.74
2.74
2.73
2.73
2.73
2.72
2.72
2.72
2.61
2.60
2.60
2.59
2.59
2.21
1.59 H2O
SO2Me
O
8e
1H NMR (500 MHz, CDCl3)
2.04
2.01
3.14
2.03
2.05
3.00
f1 (ppm)

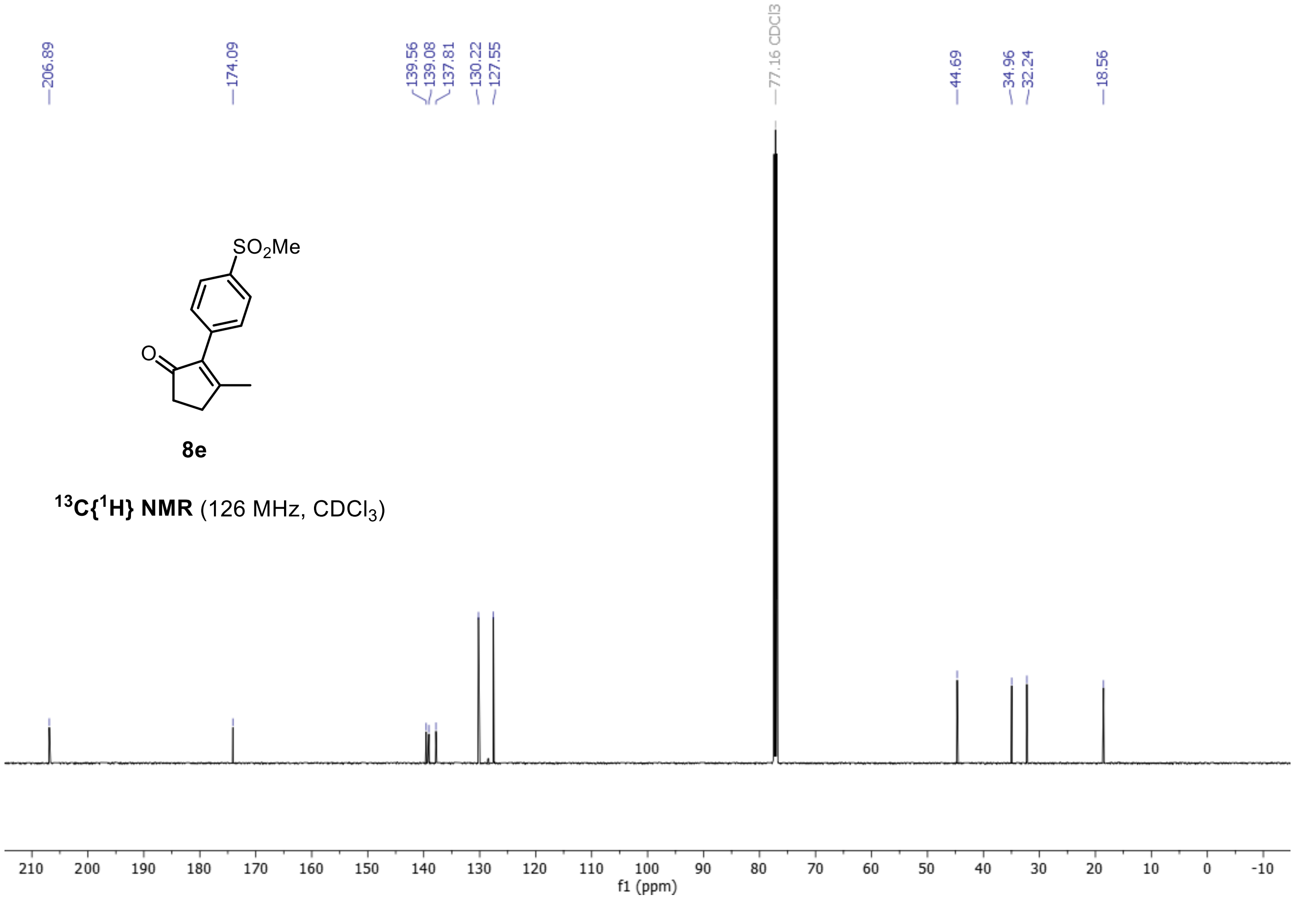
206.89
174.09
139.56
139.08
137.81
130.22
127.55
77.16 CDCl3
44.69
34.96
32.24
18.56
SO2Me
O
8e
13C{1H} NMR (126 MHz, CDCl3)
f1 (ppm)

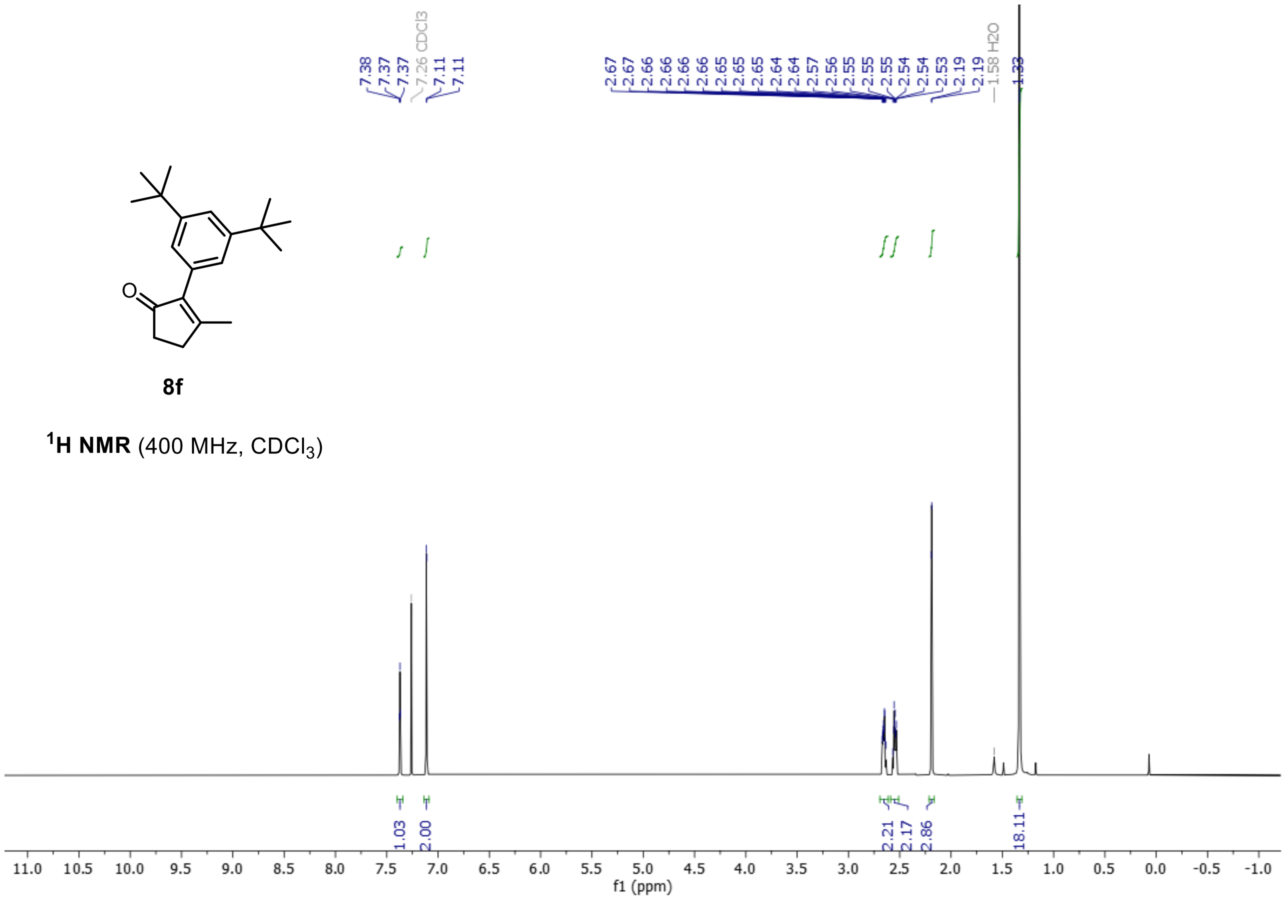

8f

^{1}H NMR (400 MHz, $CDCl_3$)

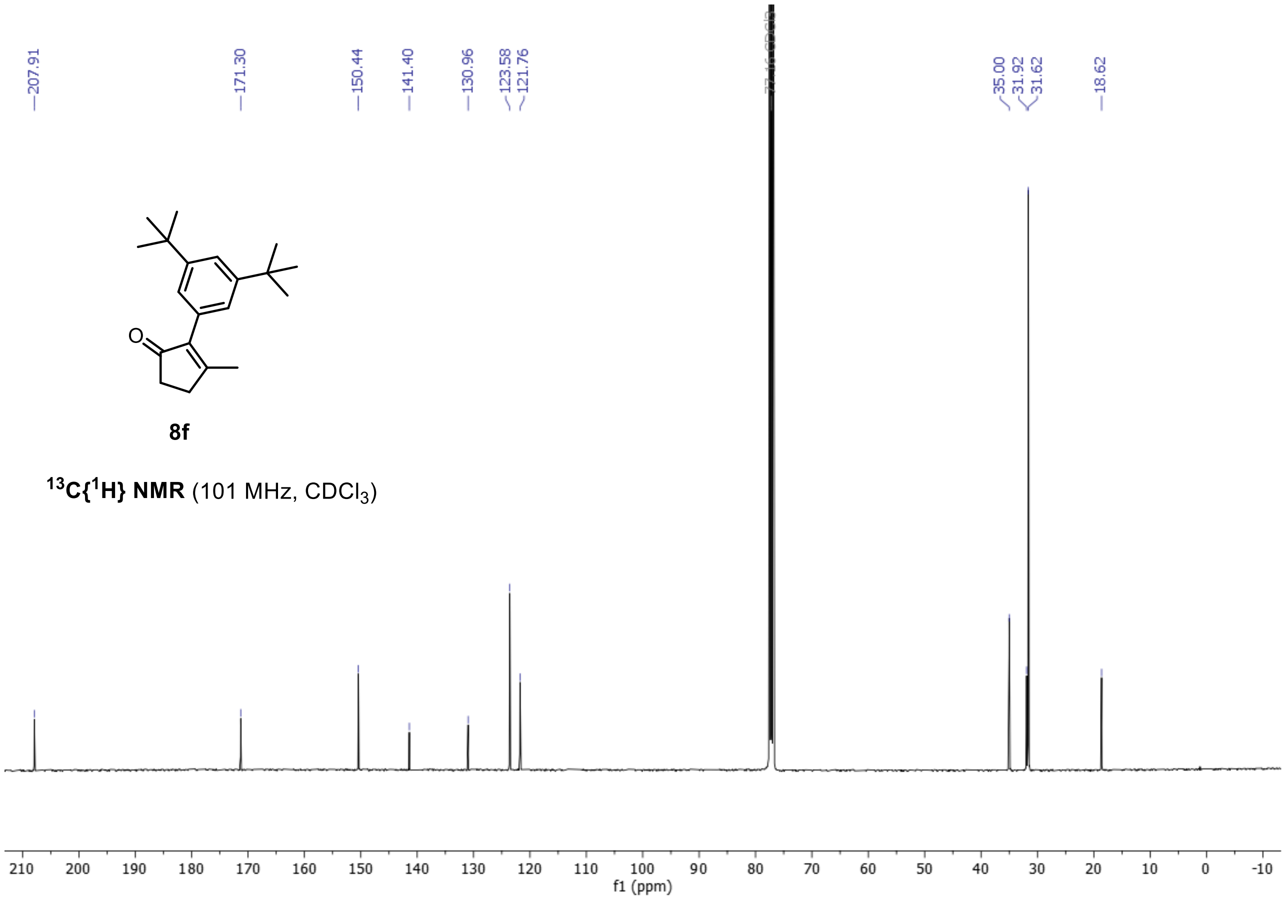

8f

$^{13}C\{^{1}H\}$ NMR (101 MHz, $CDCl_3$)

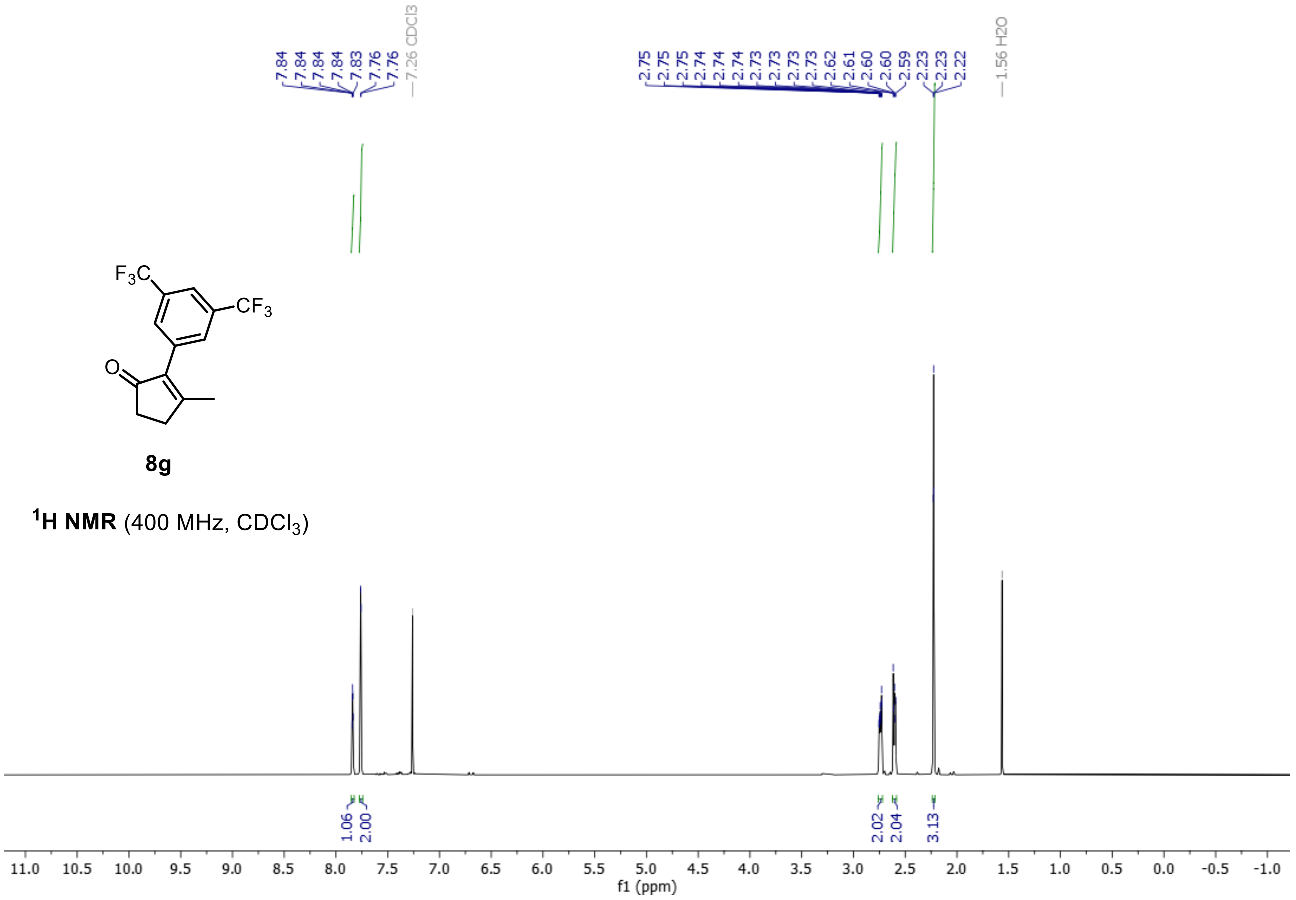

8g
¹H NMR (400 MHz, CDCl3)
f1 (ppm)

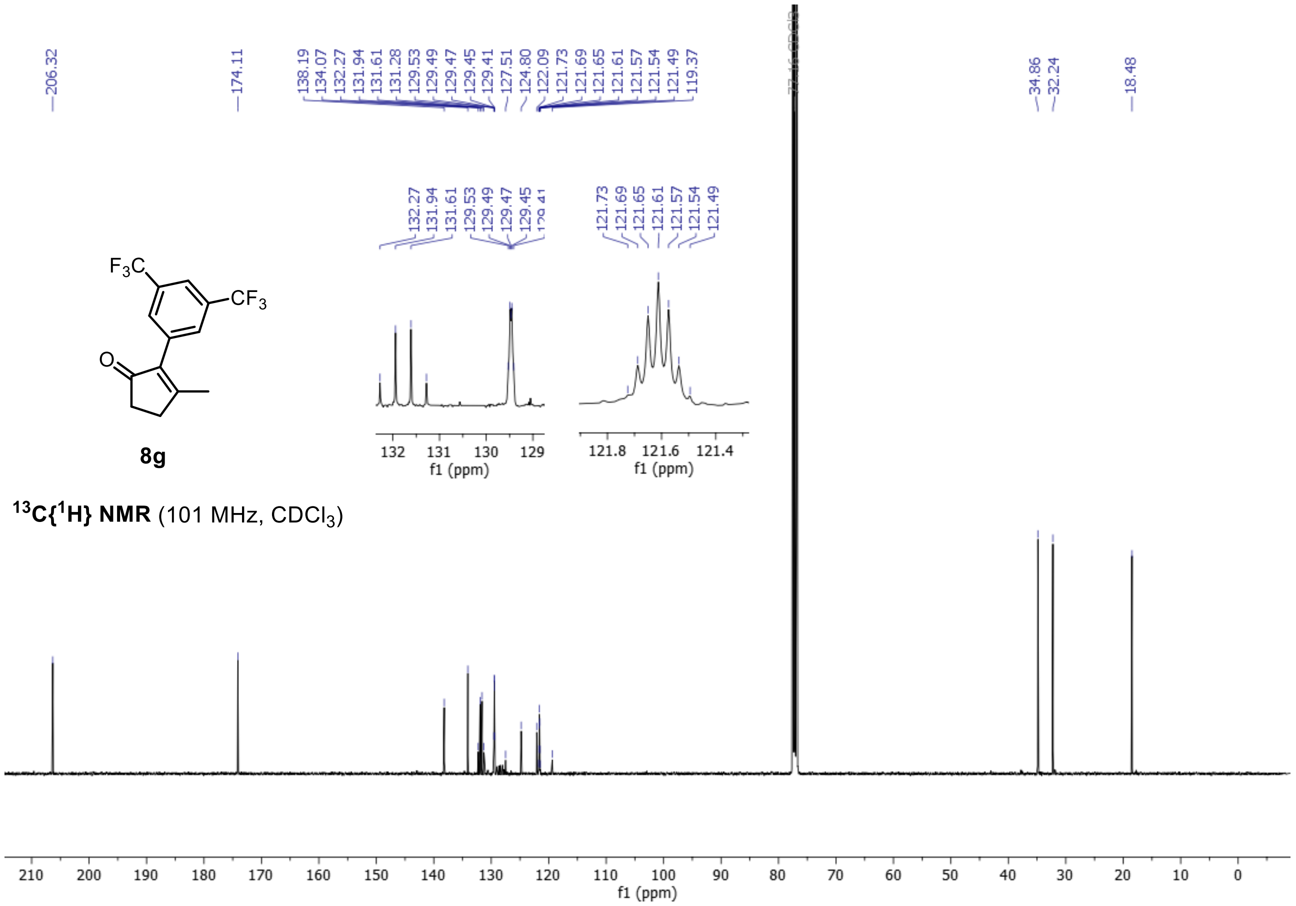

8g
¹³C{¹H} NMR (101 MHz, CDCl3)
f1 (ppm)

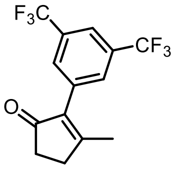

8g

^{19}F{^{1}H} NMR (376 MHz, $CDCl_3$)

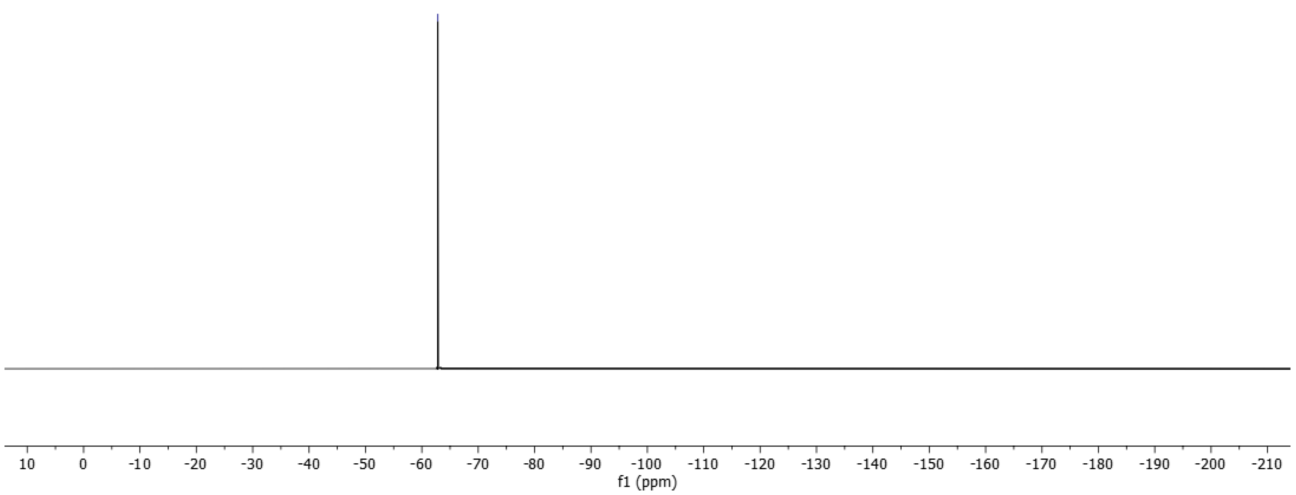

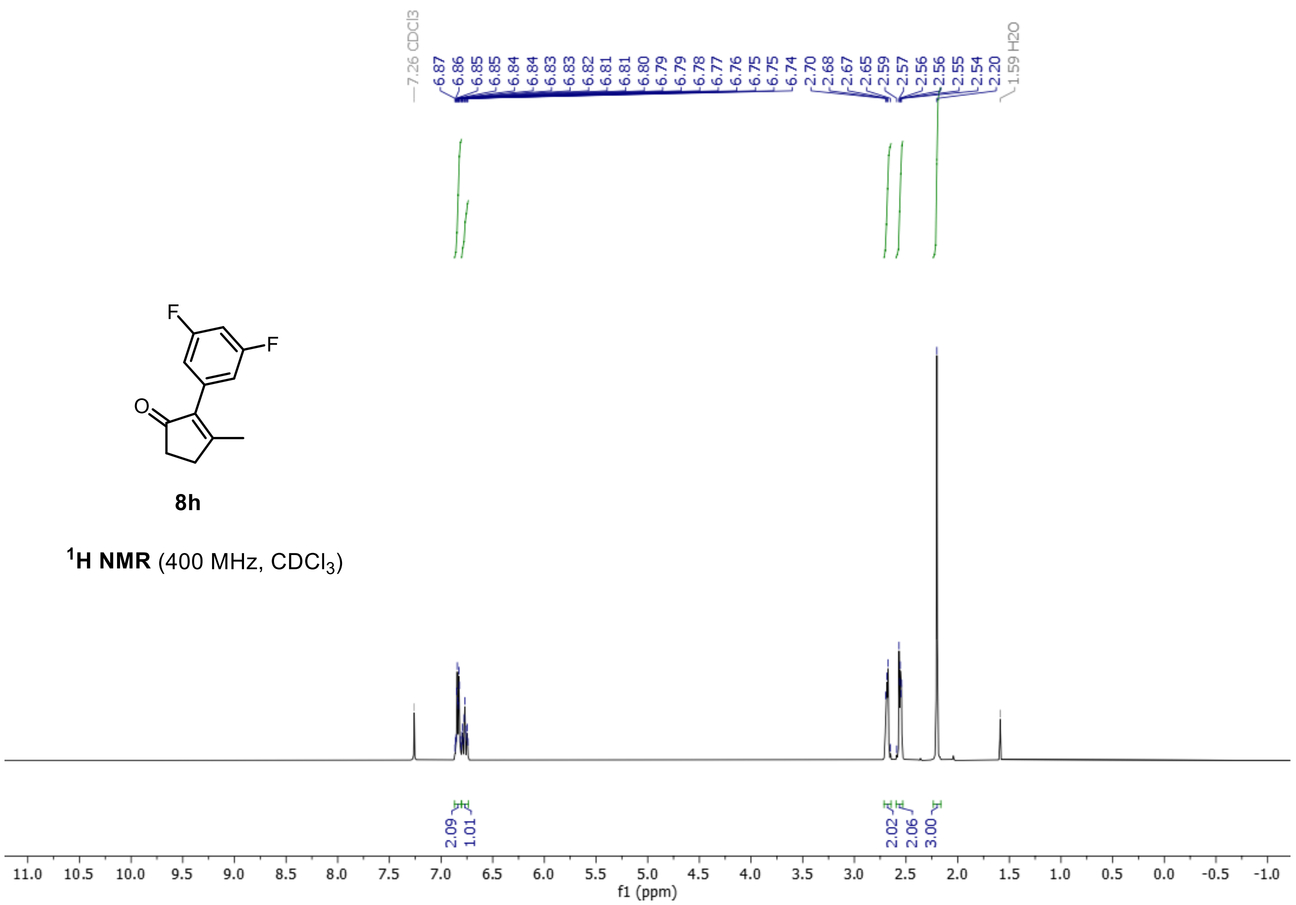

8h

^{1}H NMR (400 MHz, $CDCl_3$)

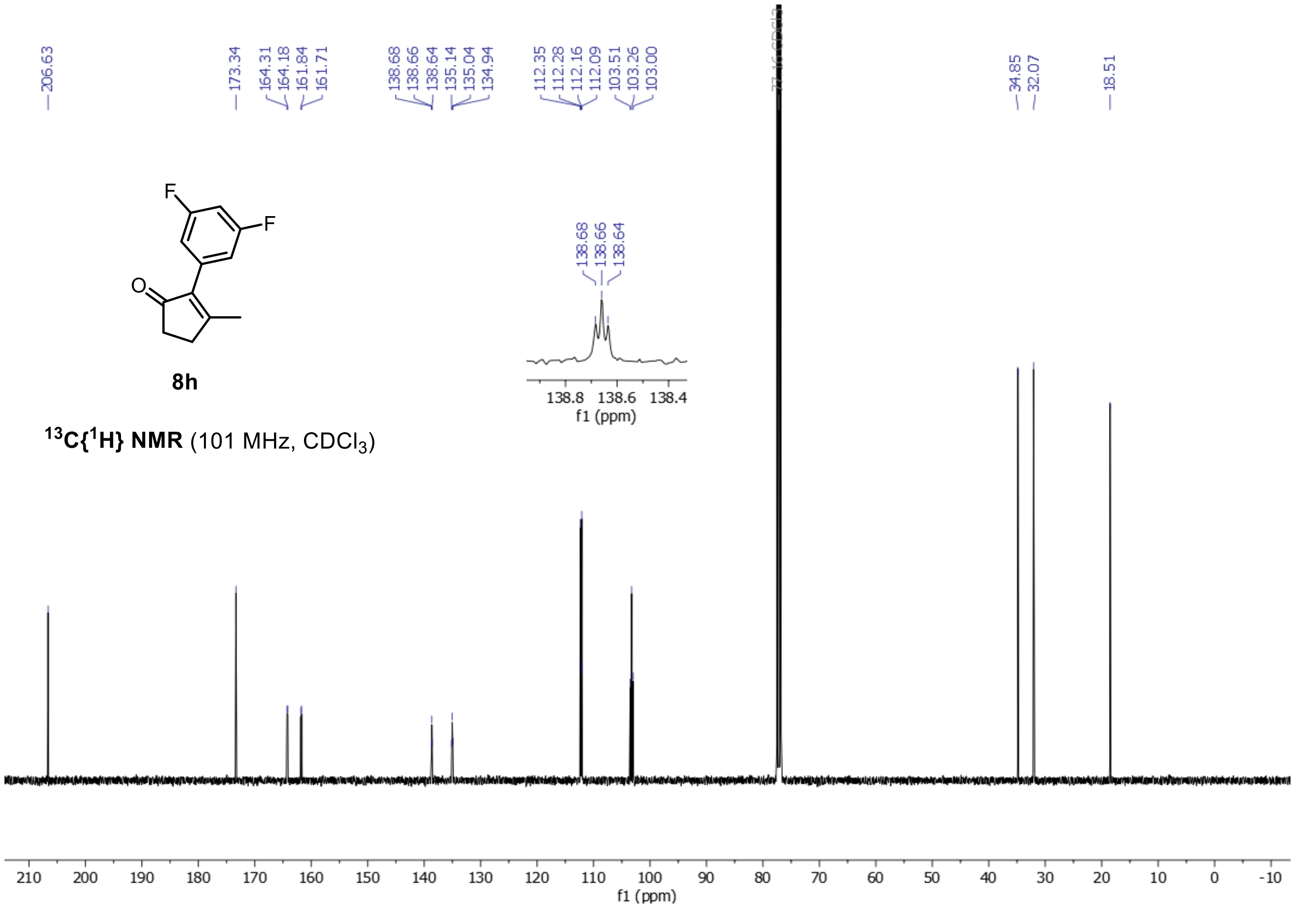

8h

^{13}C{^{1}H} NMR (101 MHz, $CDCl_3$)

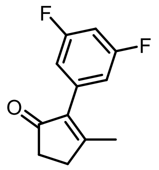

8h

$^{19}F\{^1H\}$ NMR (376 MHz, $CDCl_3$)

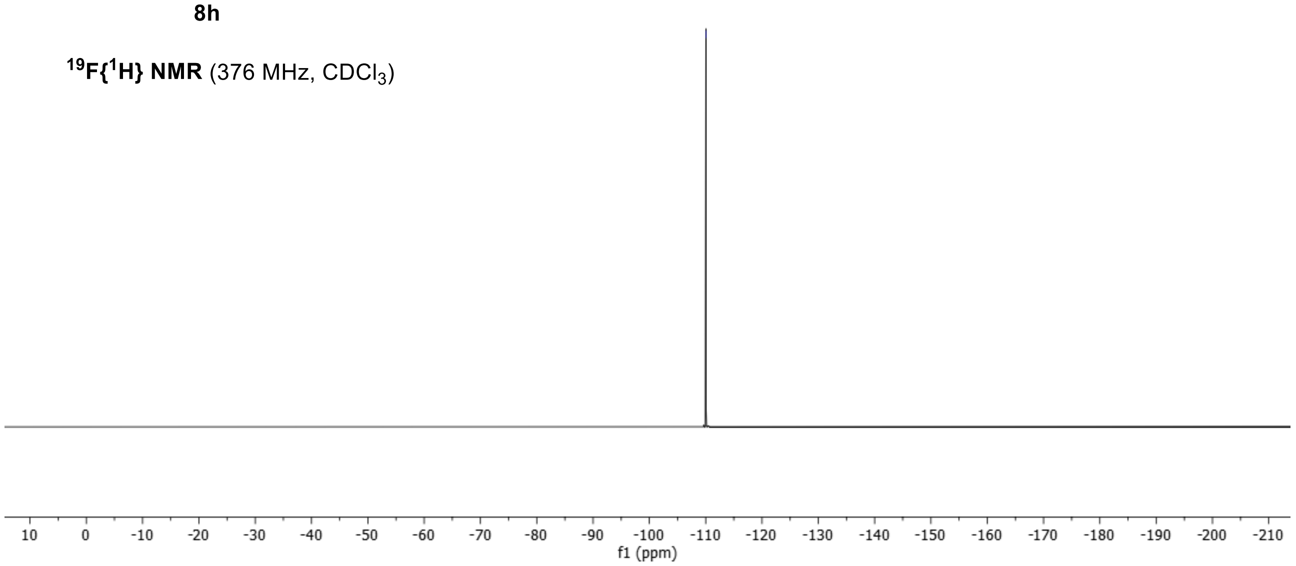

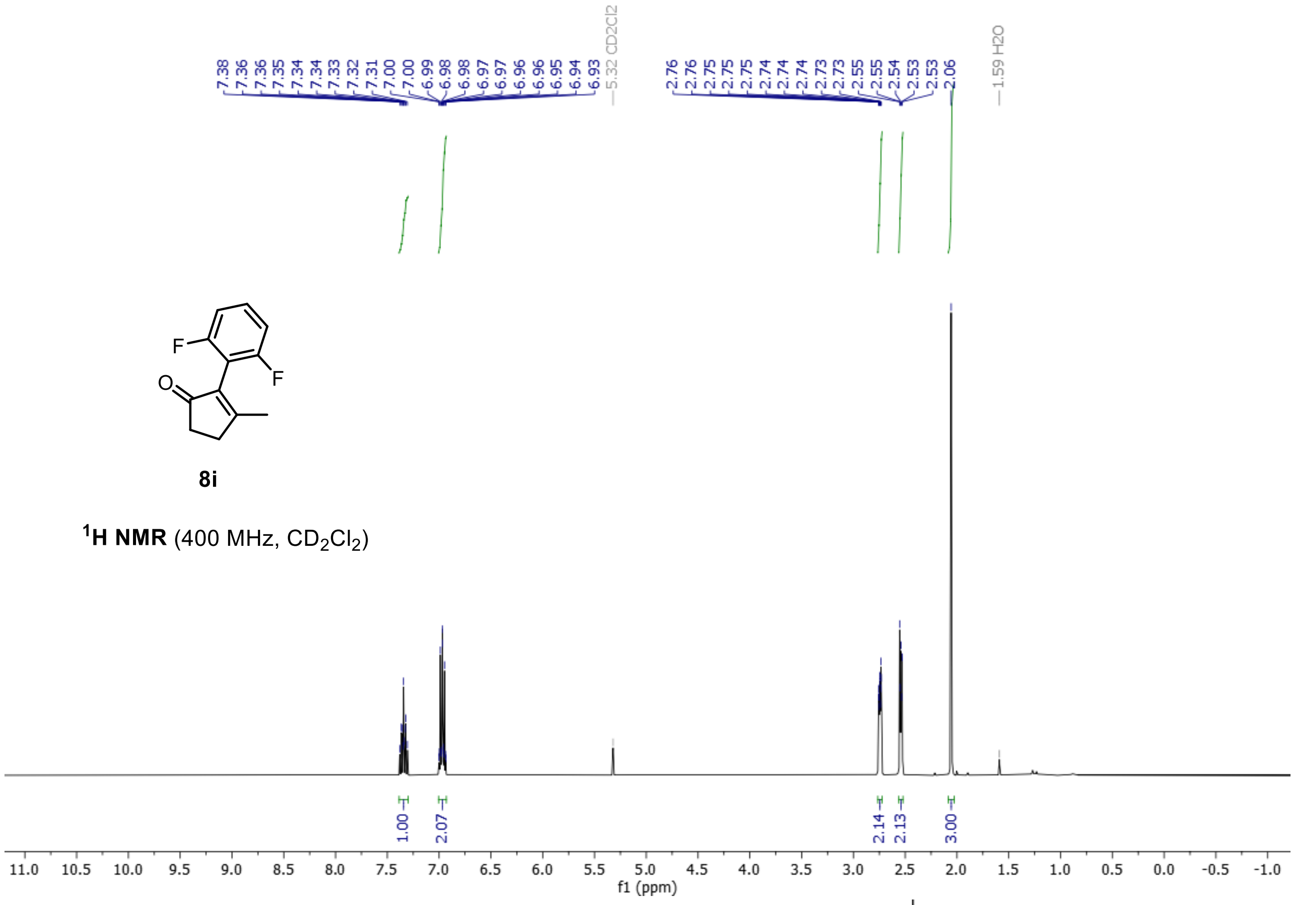
7.38
7.36
7.36
7.35
7.34
7.34
7.33
7.32
7.31
7.00
7.00
6.99
6.98
6.98
6.97
6.97
6.96
6.96
6.95
6.94
6.93
5.32 CD2Cl2
2.76
2.76
2.75
2.75
2.75
2.74
2.74
2.73
2.73
2.55
2.55
2.54
2.53
2.53
2.06
1.59 H2O
F
F
O
8i
^{1}H NMR (400 MHz, CD_2Cl_2)
1.00
2.07
2.14
2.13
3.00
11.0 10.5 10.0 9.5 9.0 8.5 8.0 7.5 7.0 6.5 6.0 5.5 5.0 4.5 4.0 3.5 3.0 2.5 2.0 1.5 1.0 0.5 0.0 -0.5 -1.0
f1 (ppm)

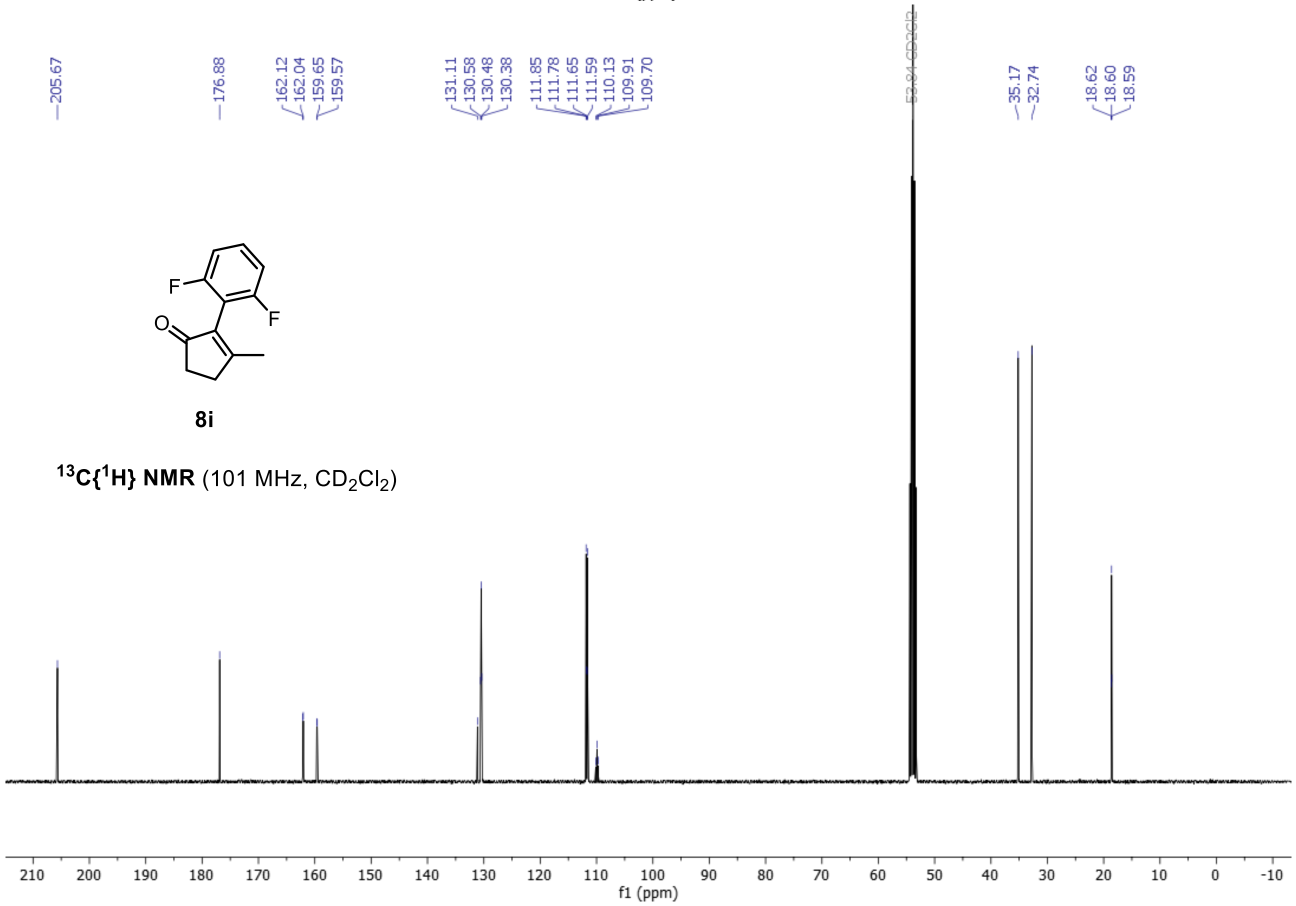
205.67
176.88
162.12
162.04
159.65
159.57
131.11
130.58
130.48
130.38
111.85
111.78
111.65
111.59
110.13
109.91
109.70
53.84 CD2Cl2
35.17
32.74
18.62
18.60
18.59
F
F
O
8i
^{13}C{^{1}H} NMR (101 MHz, CD_2Cl_2)
210 200 190 180 170 160 150 140 130 120 110 100 90 80 70 60 50 40 30 20 10 0 -10
f1 (ppm)

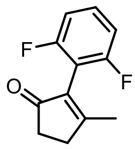

8i

^{19}**F{^{1}H} NMR** (376 MHz, CD_2Cl_2)

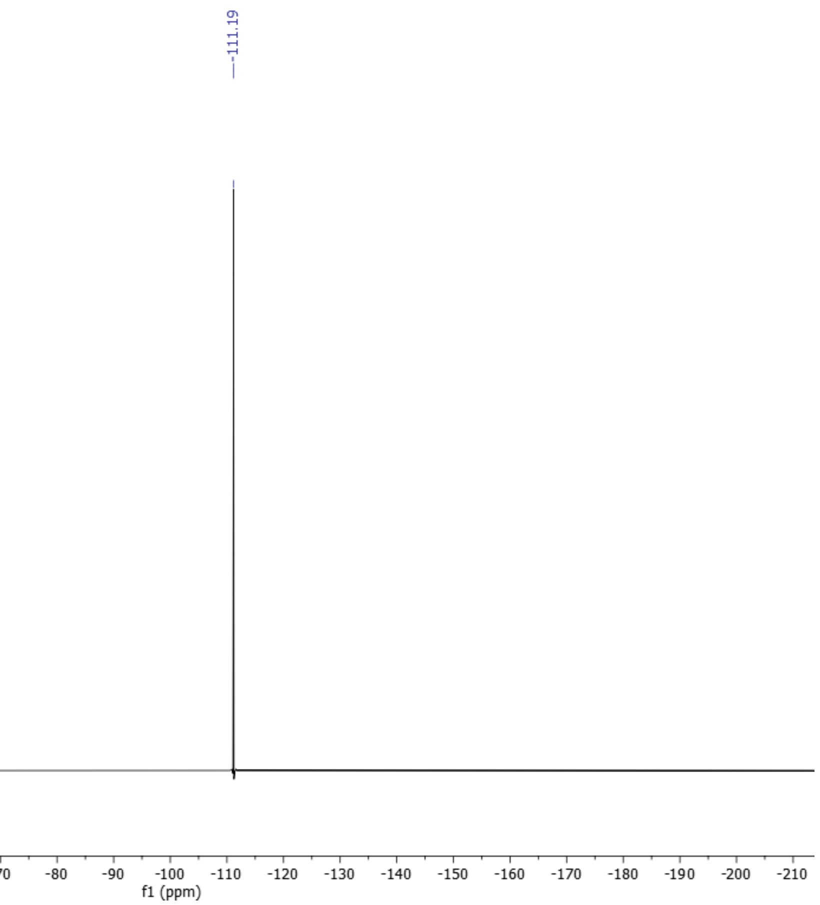

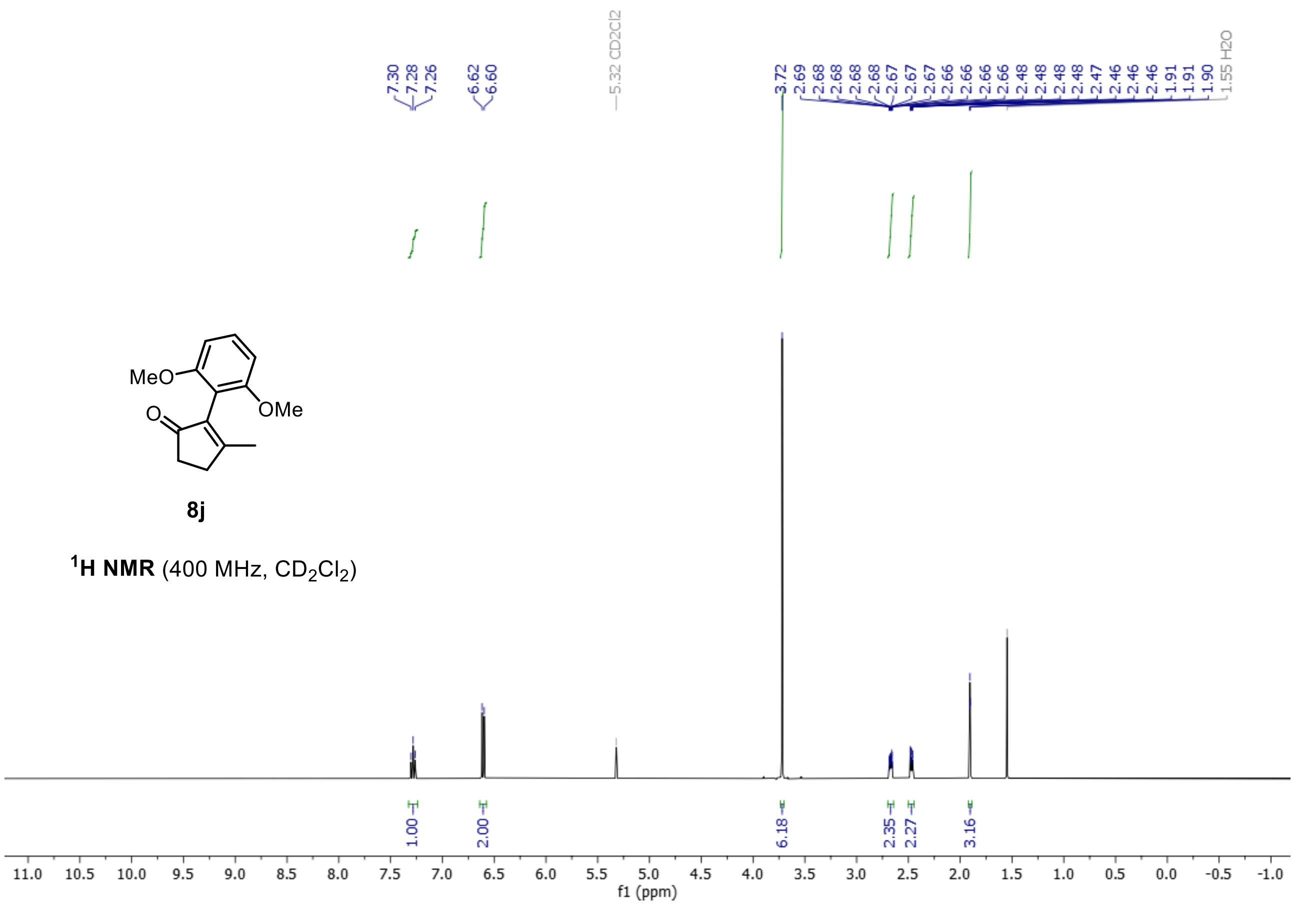

8j

^{1}H NMR (400 MHz, CD_2Cl_2)

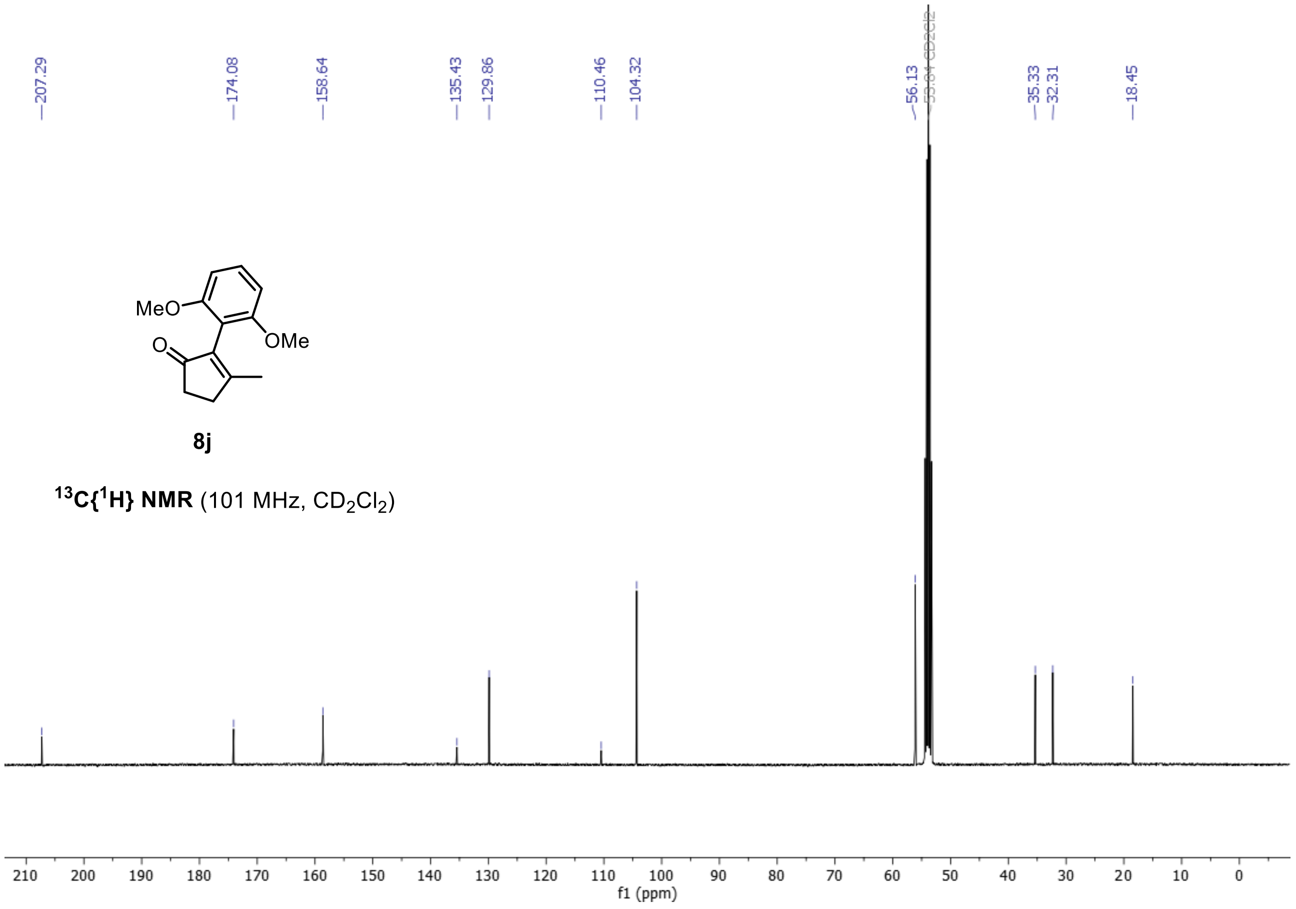

8j

^{13}C{^{1}H} NMR (101 MHz, CD_2Cl_2)

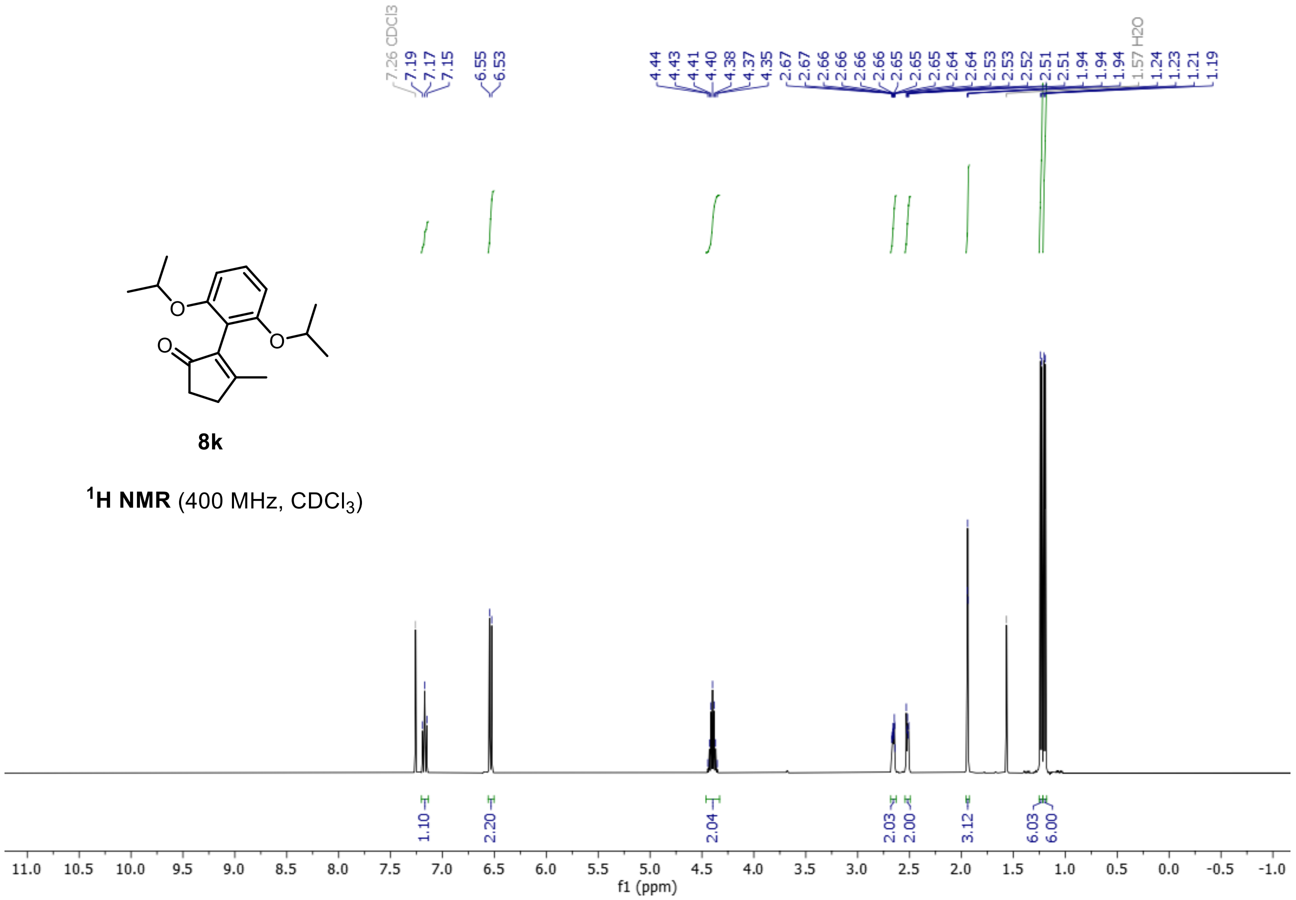

8k

^{1}H NMR (400 MHz, $CDCl_3$)

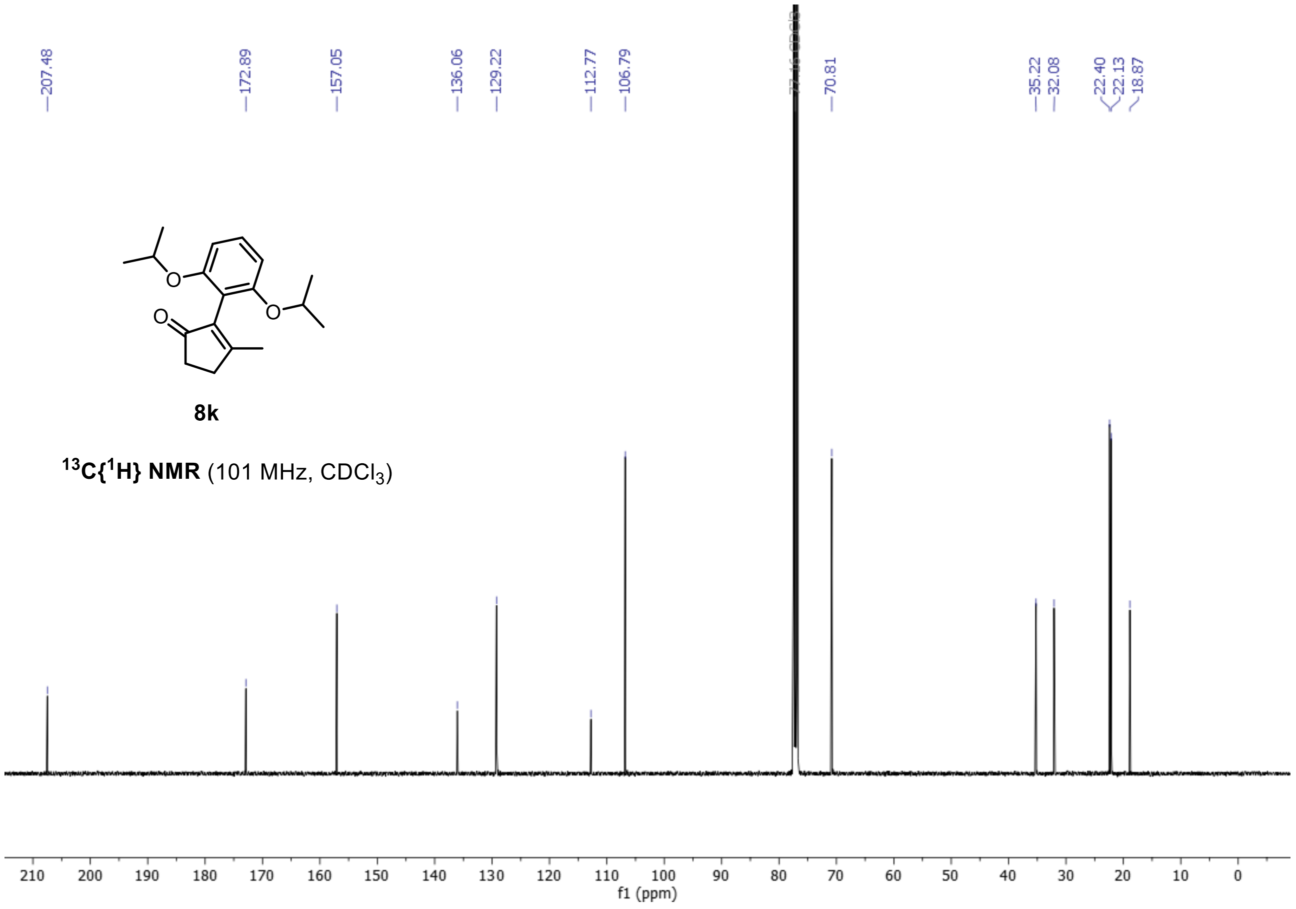

8k

$^{13}C\{^1H\}$ NMR (101 MHz, $CDCl_3$)

8l

^{1}H NMR (400 MHz, CD_2Cl_2)

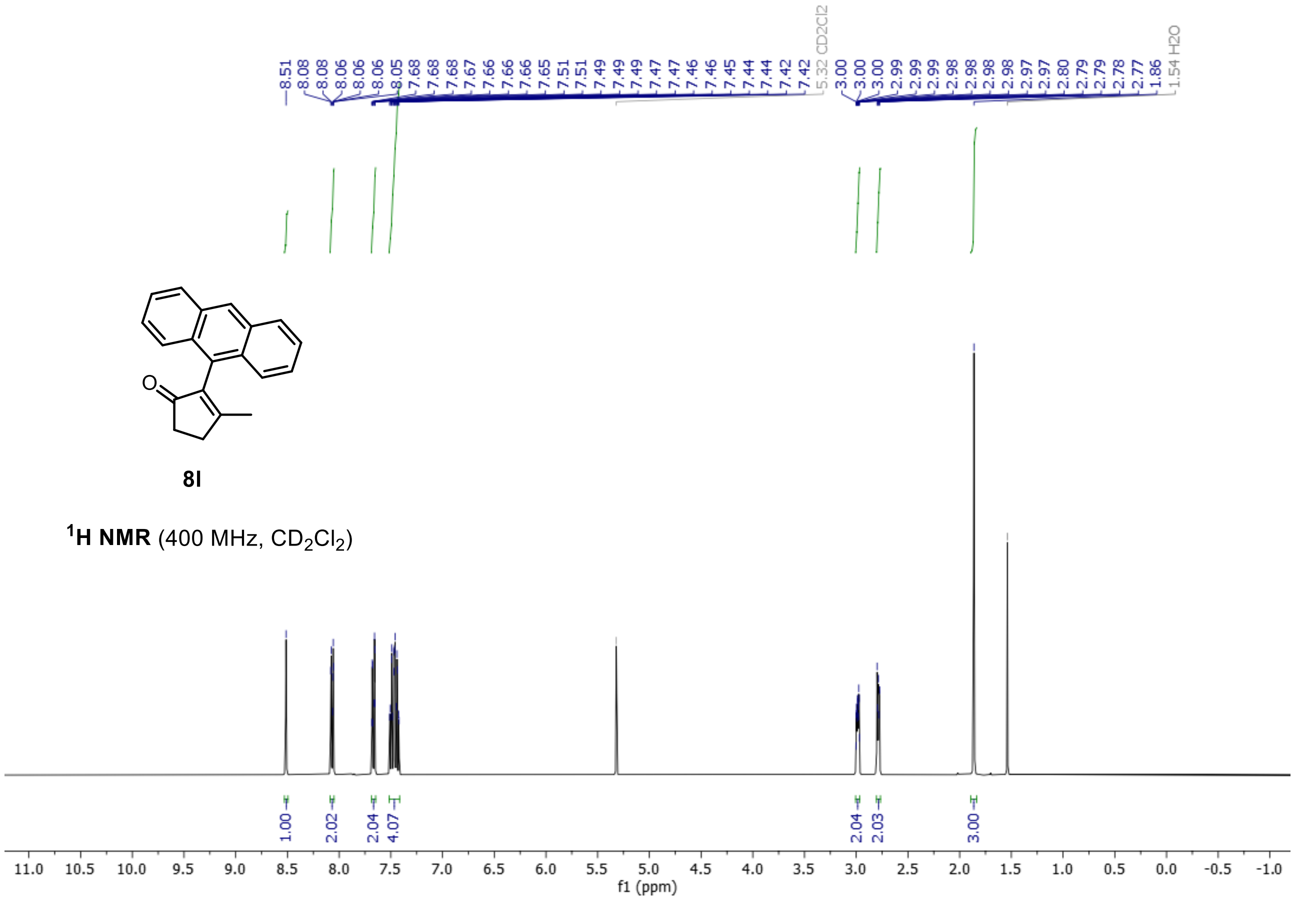

8l

$^{13}C\{^1H\}$ NMR (101 MHz, CD_2Cl_2)

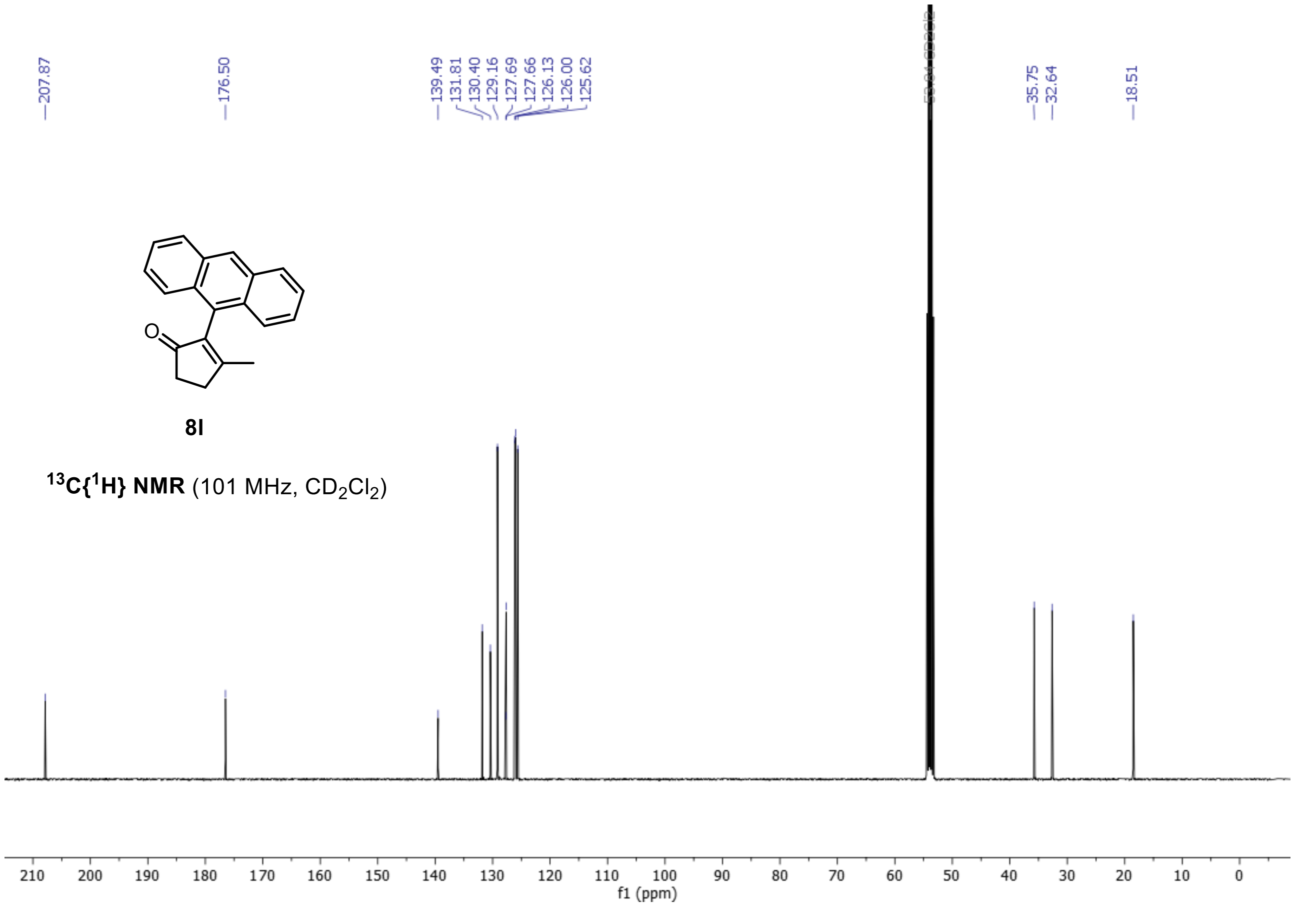

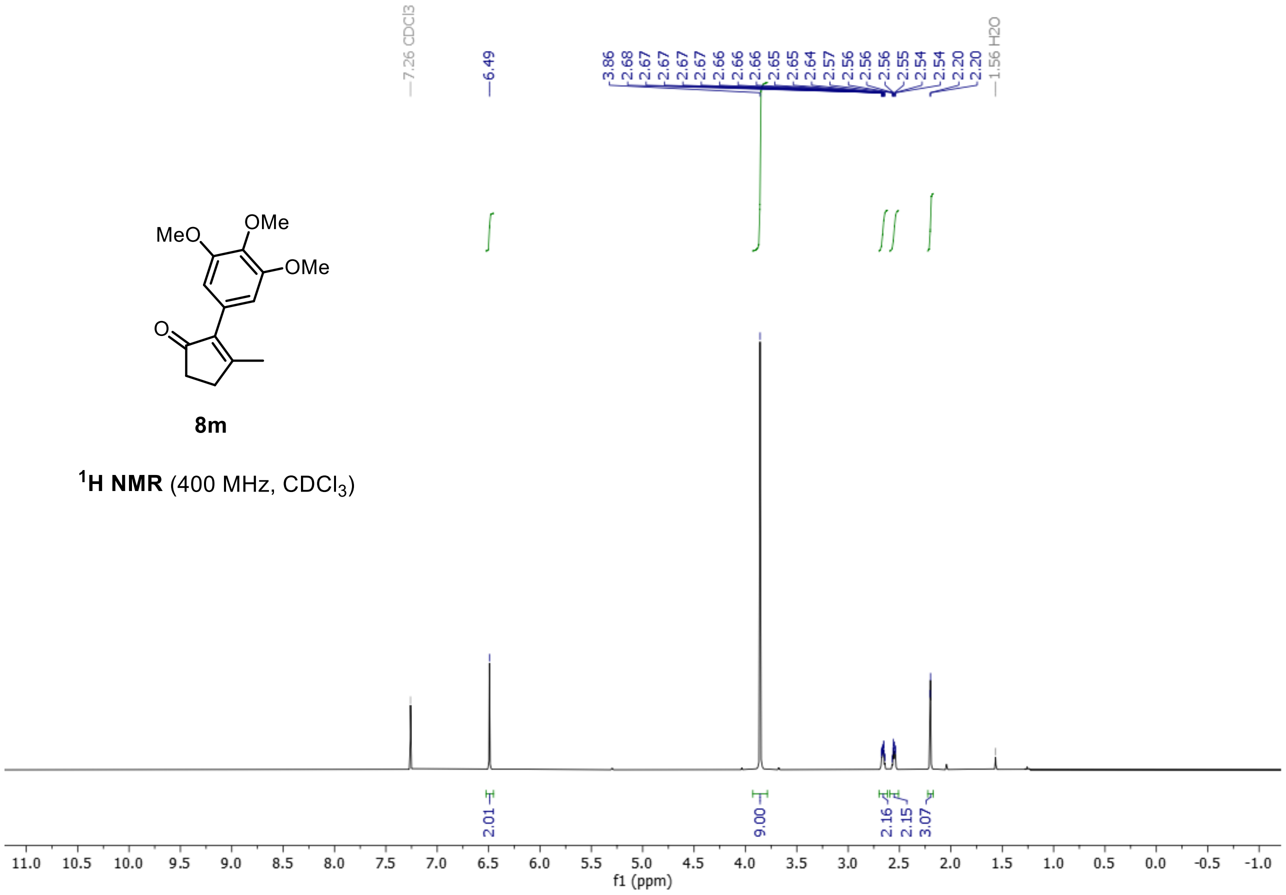
8m
1H NMR (400 MHz, CDCl3)
7.26 CDCl3
6.49
3.86
2.68
2.67
2.67
2.67
2.67
2.66
2.66
2.66
2.65
2.65
2.64
2.57
2.56
2.56
2.56
2.55
2.54
2.54
2.20
2.20
1.56 H2O
2.01
9.00
2.16
2.15
3.07
11.0 10.5 10.0 9.5 9.0 8.5 8.0 7.5 7.0 6.5 6.0 5.5 5.0 4.5 4.0 3.5 3.0 2.5 2.0 1.5 1.0 0.5 0.0 -0.5 -1.0
f1 (ppm)

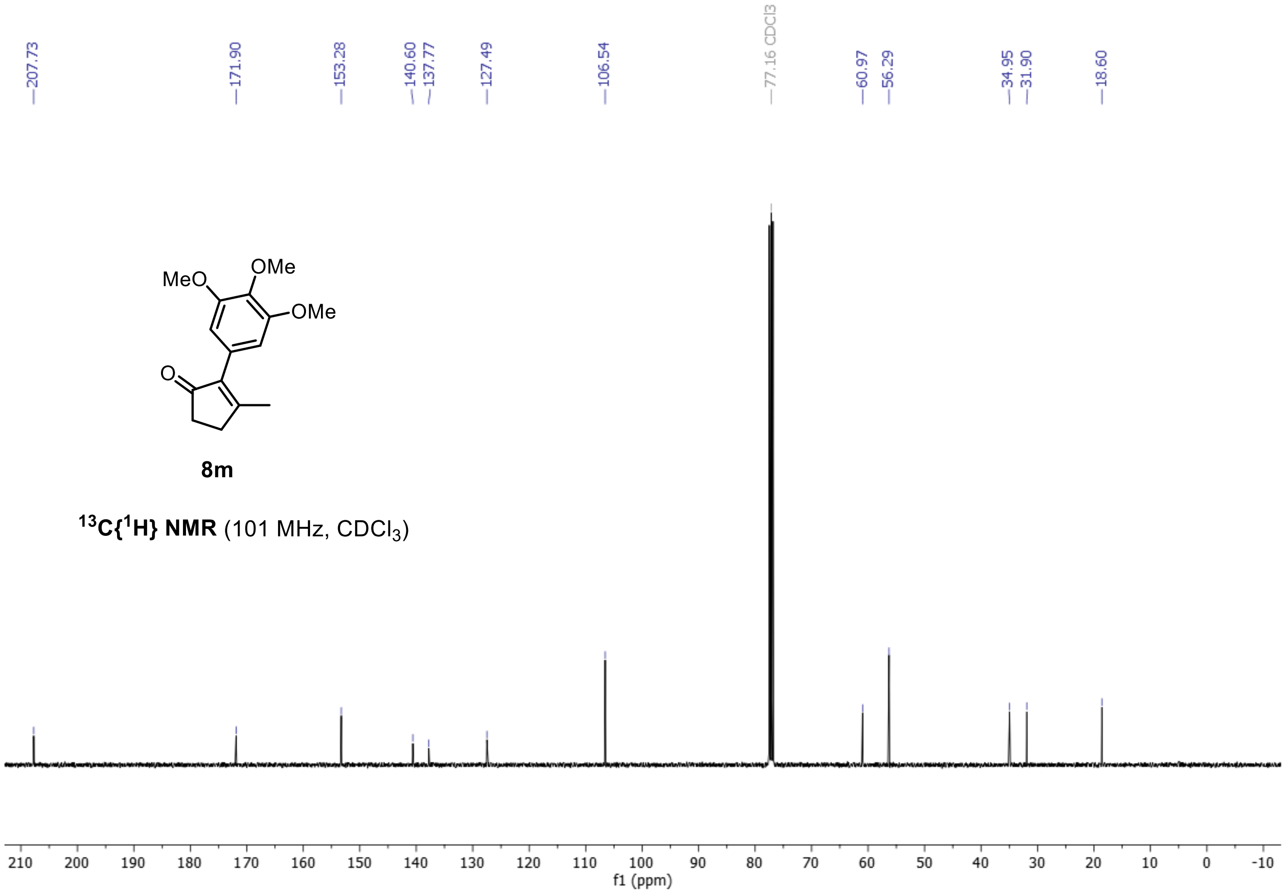
8m
13C{1H} NMR (101 MHz, CDCl3)
207.73
171.90
153.28
140.60
137.77
127.49
106.54
77.16 CDCl3
60.97
56.29
34.95
31.90
18.60
210 200 190 180 170 160 150 140 130 120 110 100 90 80 70 60 50 40 30 20 10 0 -10
f1 (ppm)

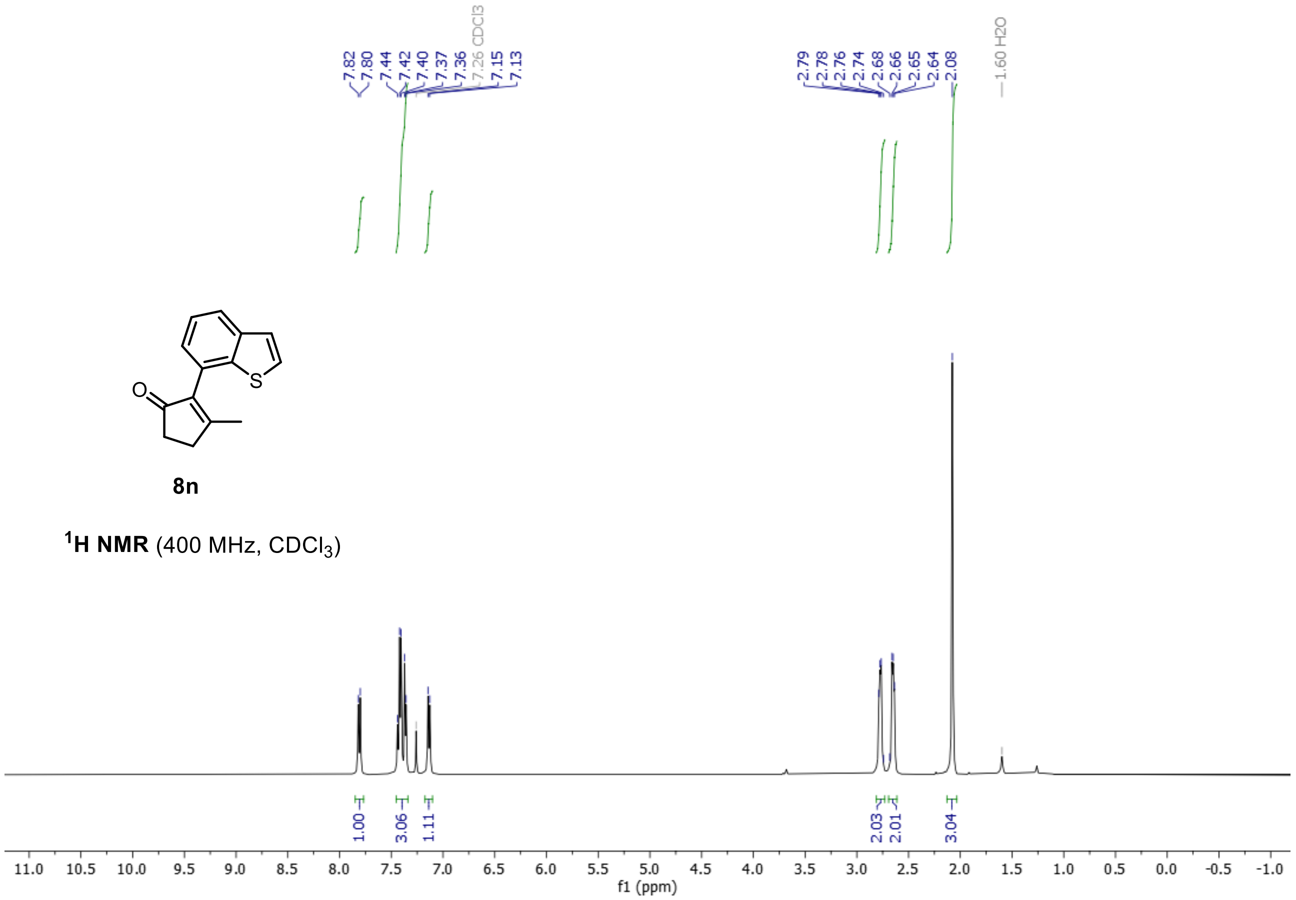

8n

^{1}H NMR (400 MHz, $CDCl_3$)

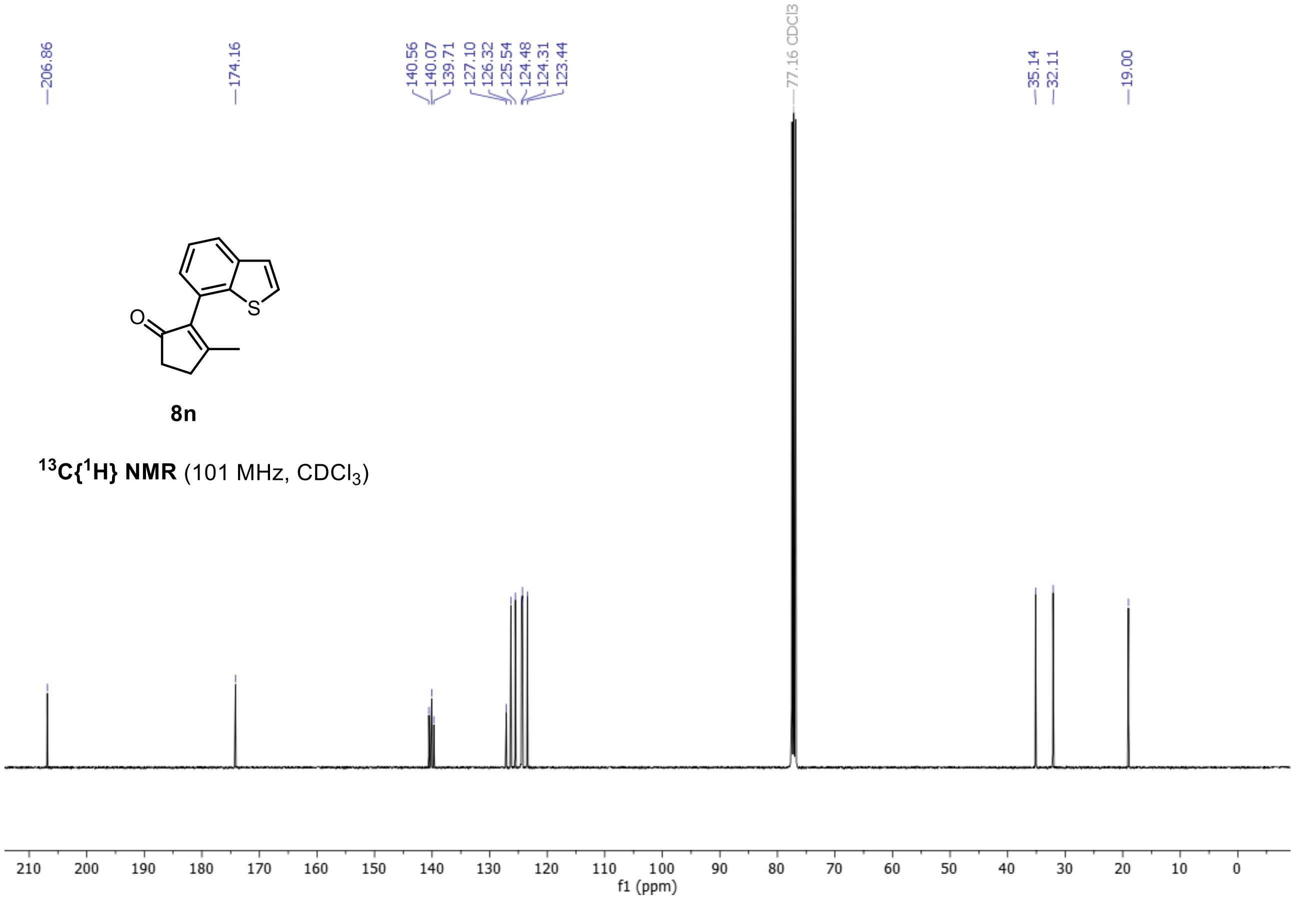

8n

^{13}C{^{1}H} NMR (101 MHz, $CDCl_3$)

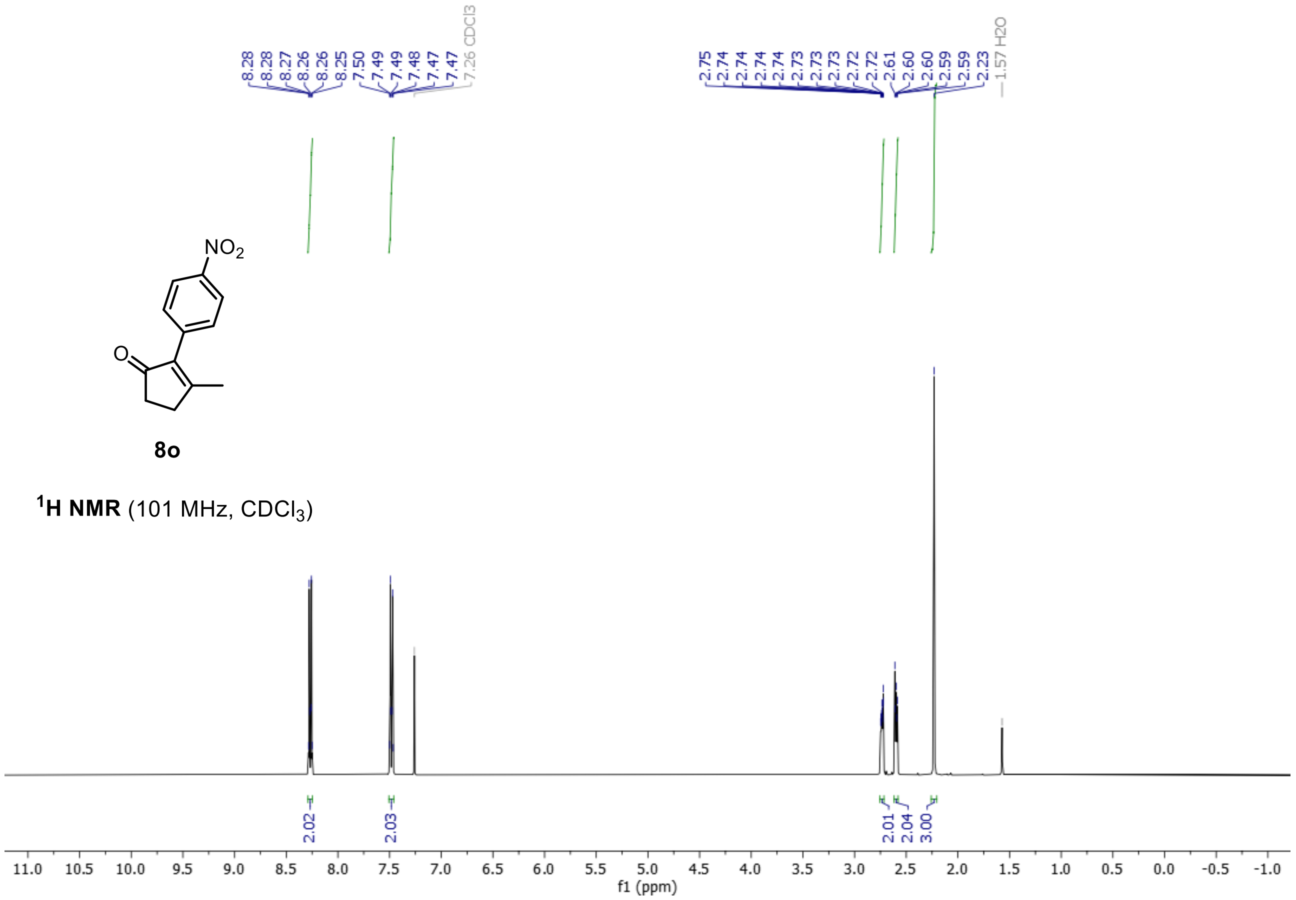

8o

^{1}H NMR (101 MHz, $CDCl_3$)

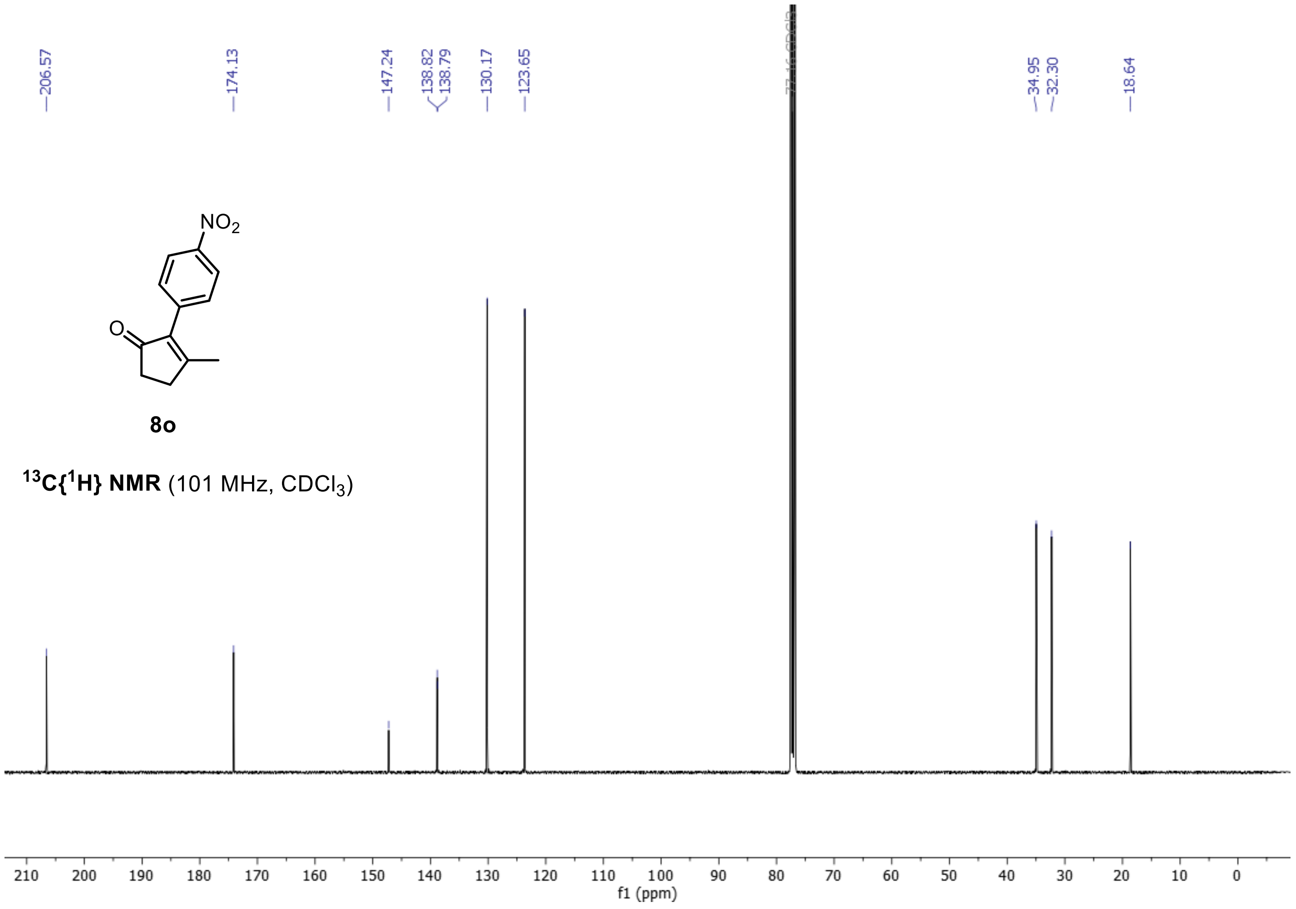

8o

^{13}C{^{1}H} NMR (101 MHz, $CDCl_3$)

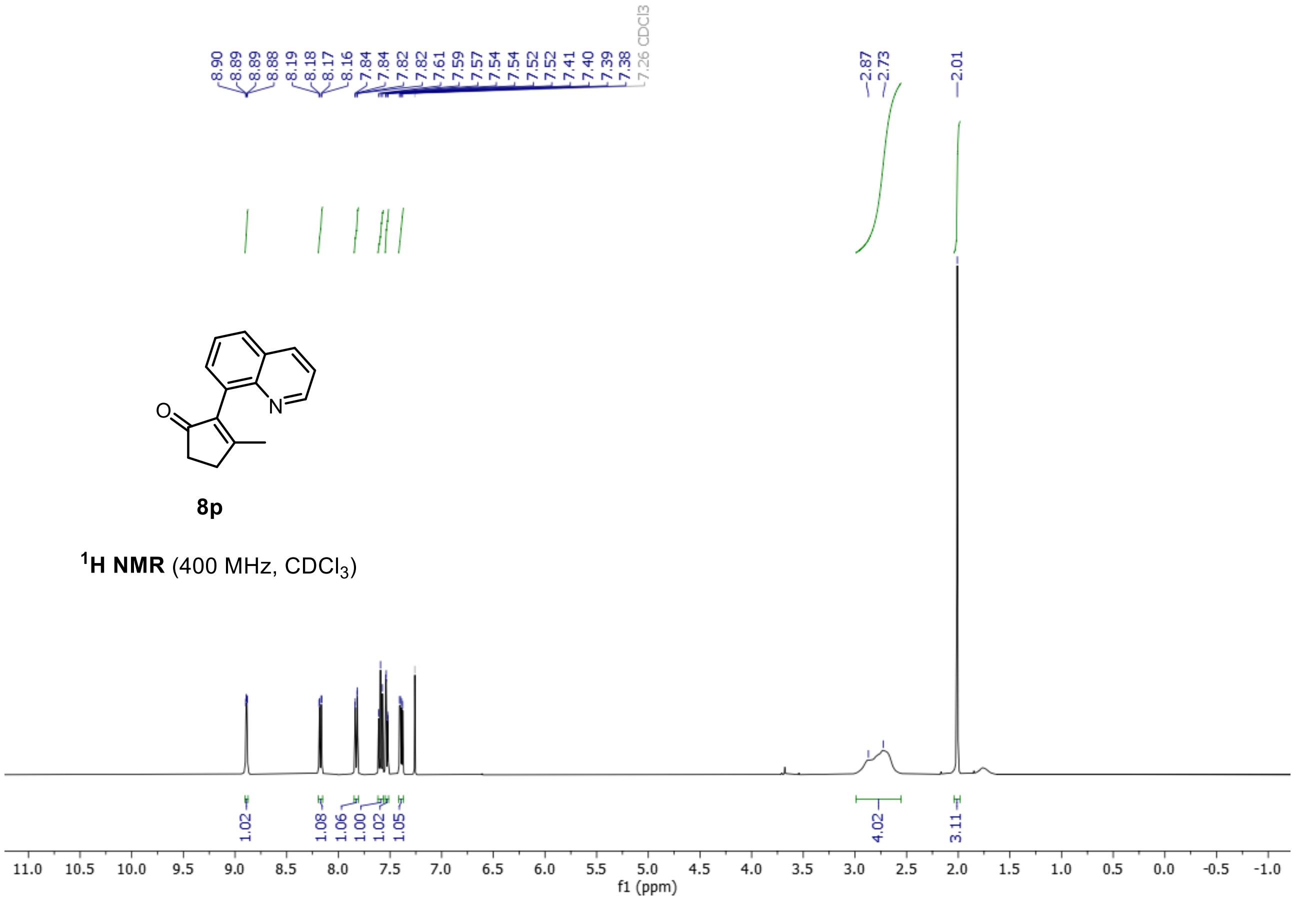

8p

^{1}H NMR (400 MHz, $CDCl_3$)

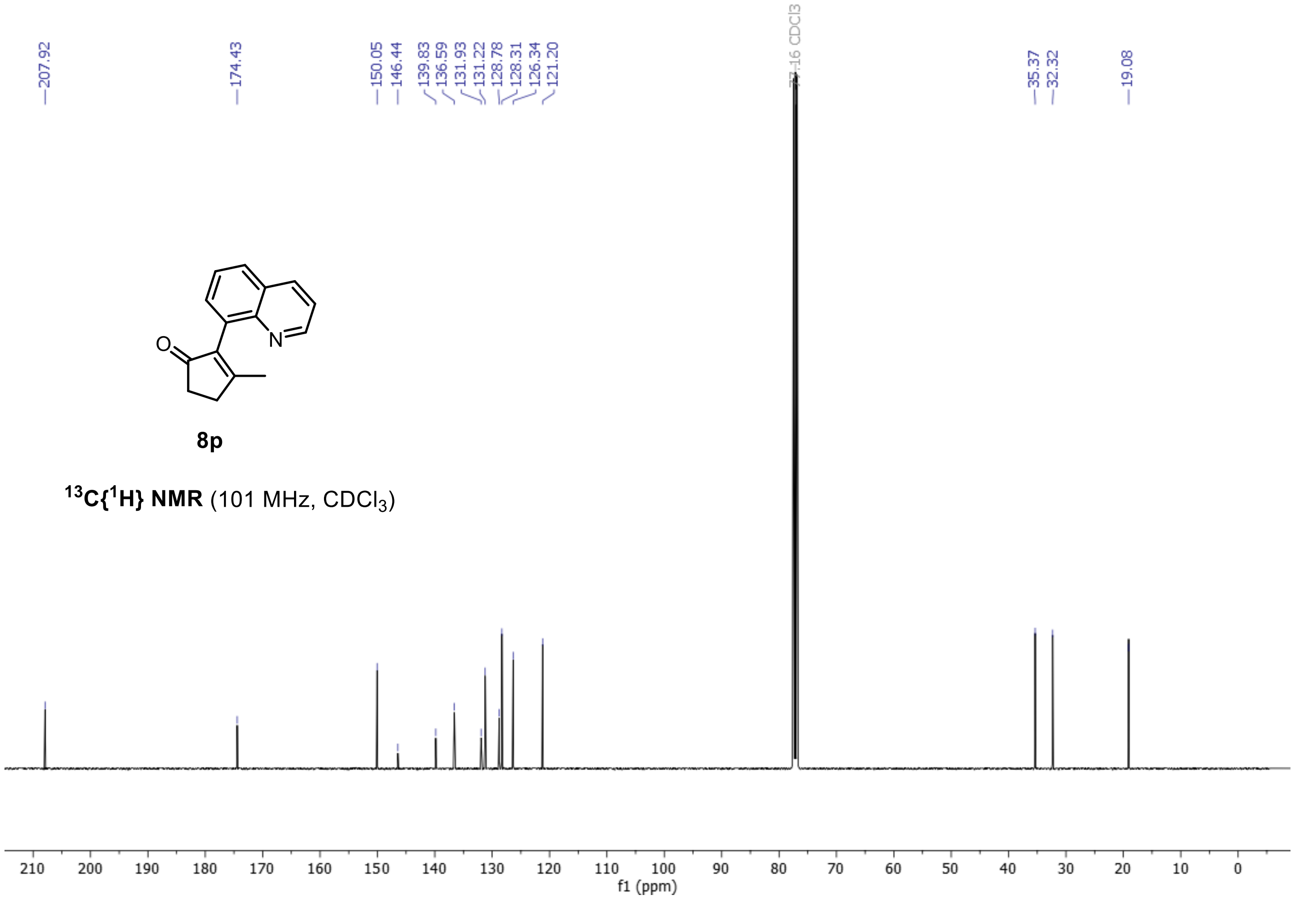

8p

^{13}C{^{1}H} NMR (101 MHz, $CDCl_3$)

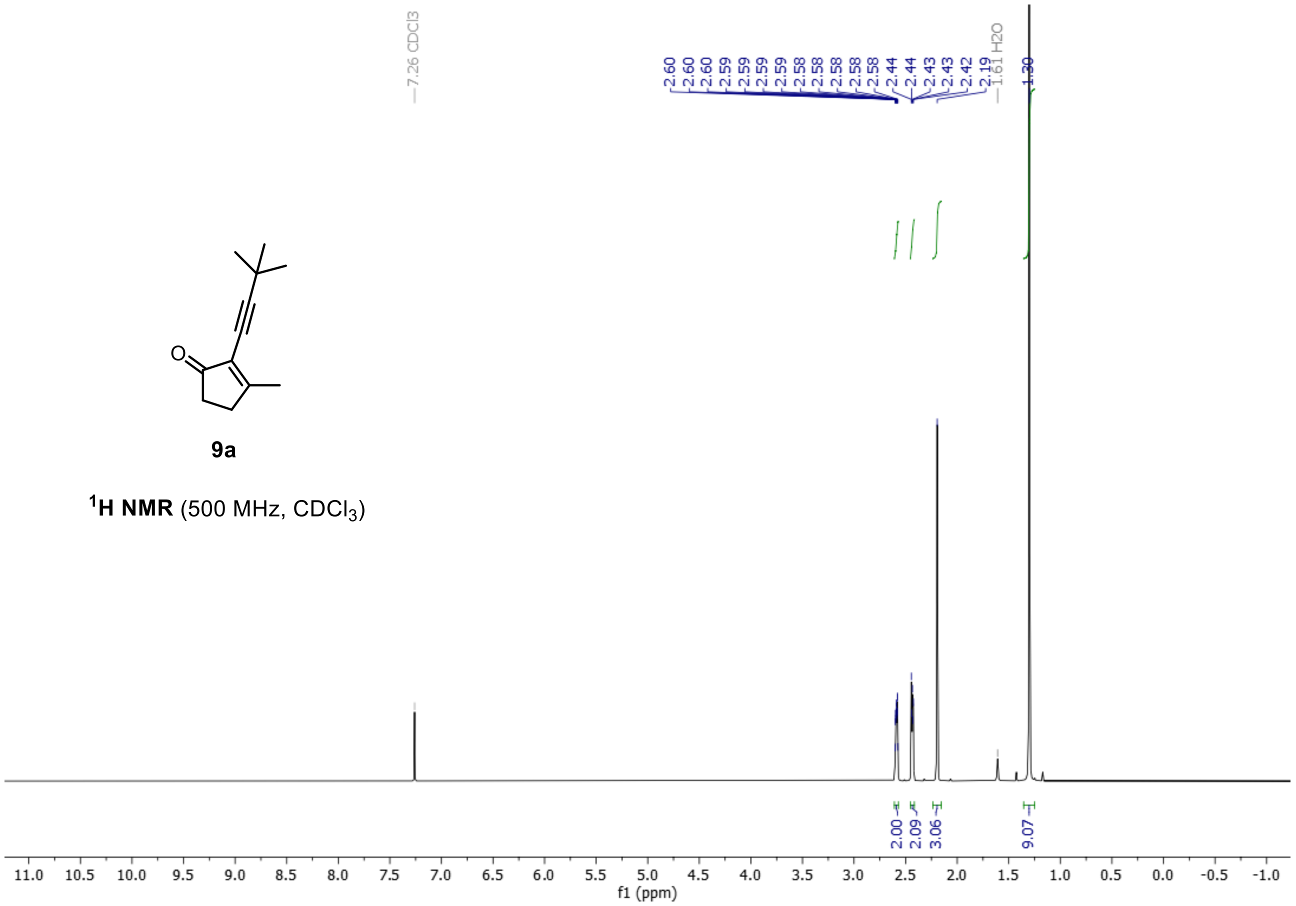
9a
1H NMR (500 MHz, CDCl3)
f1 (ppm)

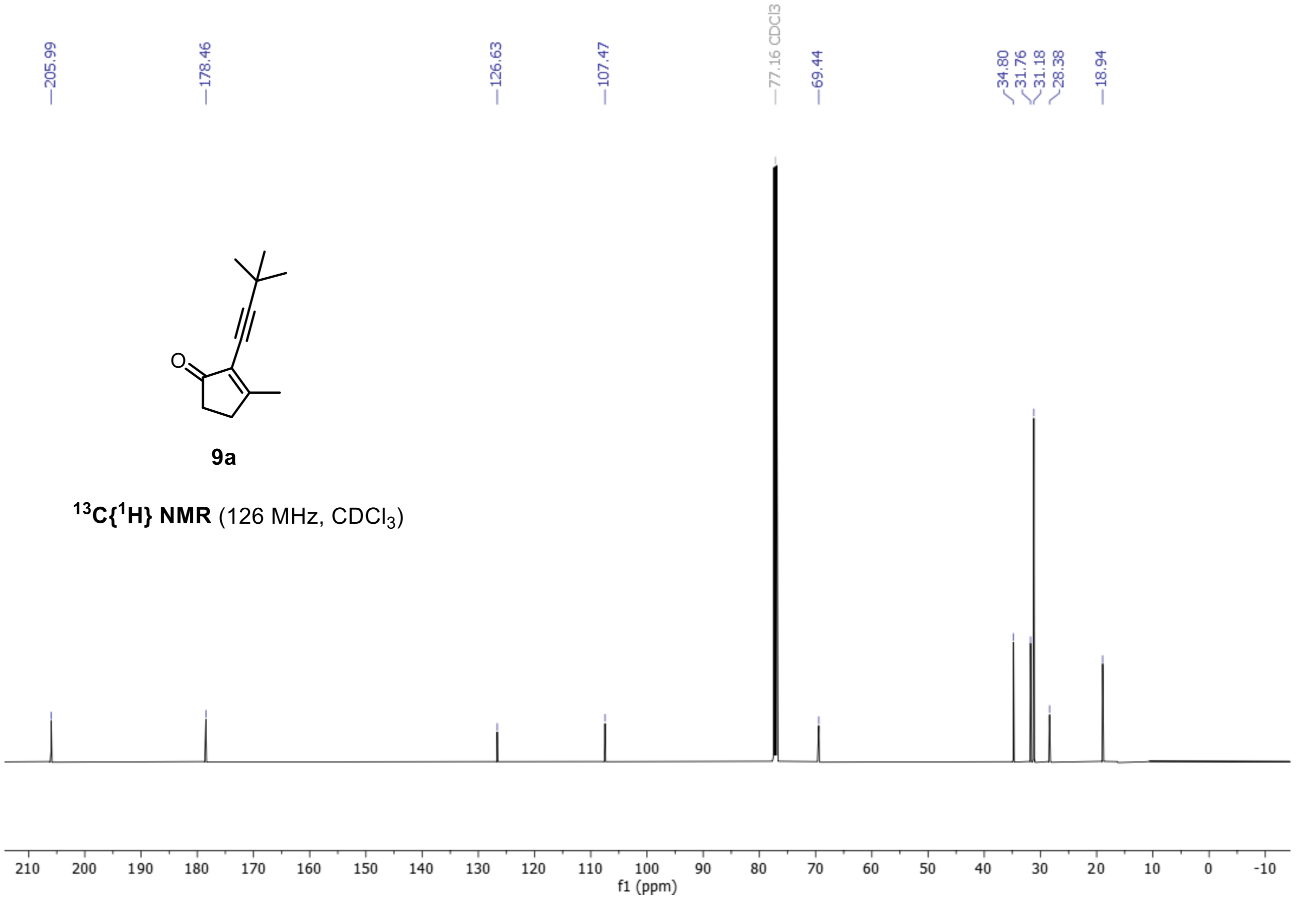
9a
13C{1H} NMR (126 MHz, CDCl3)
f1 (ppm)

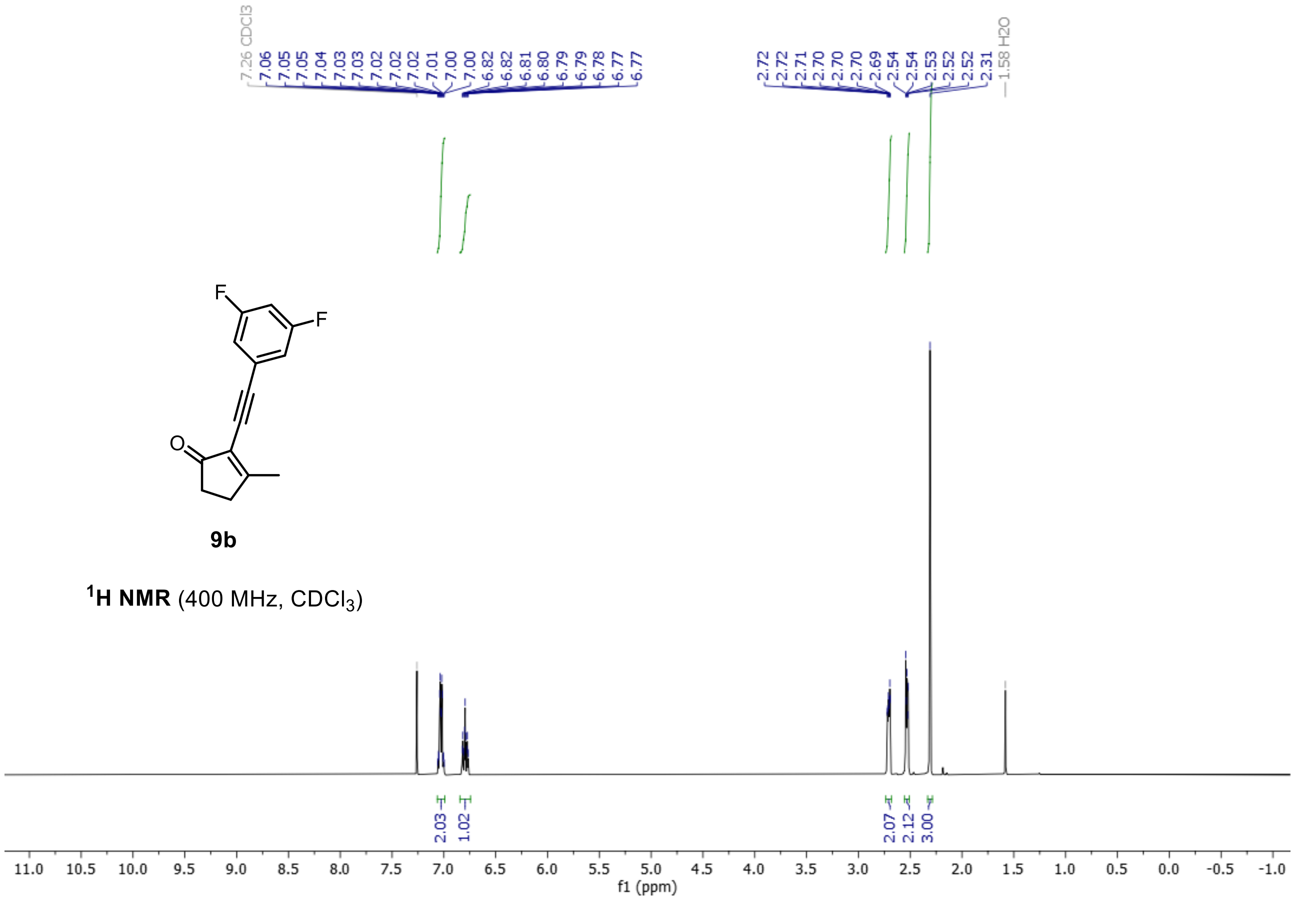

9b

^{1}H NMR (400 MHz, $CDCl_3$)

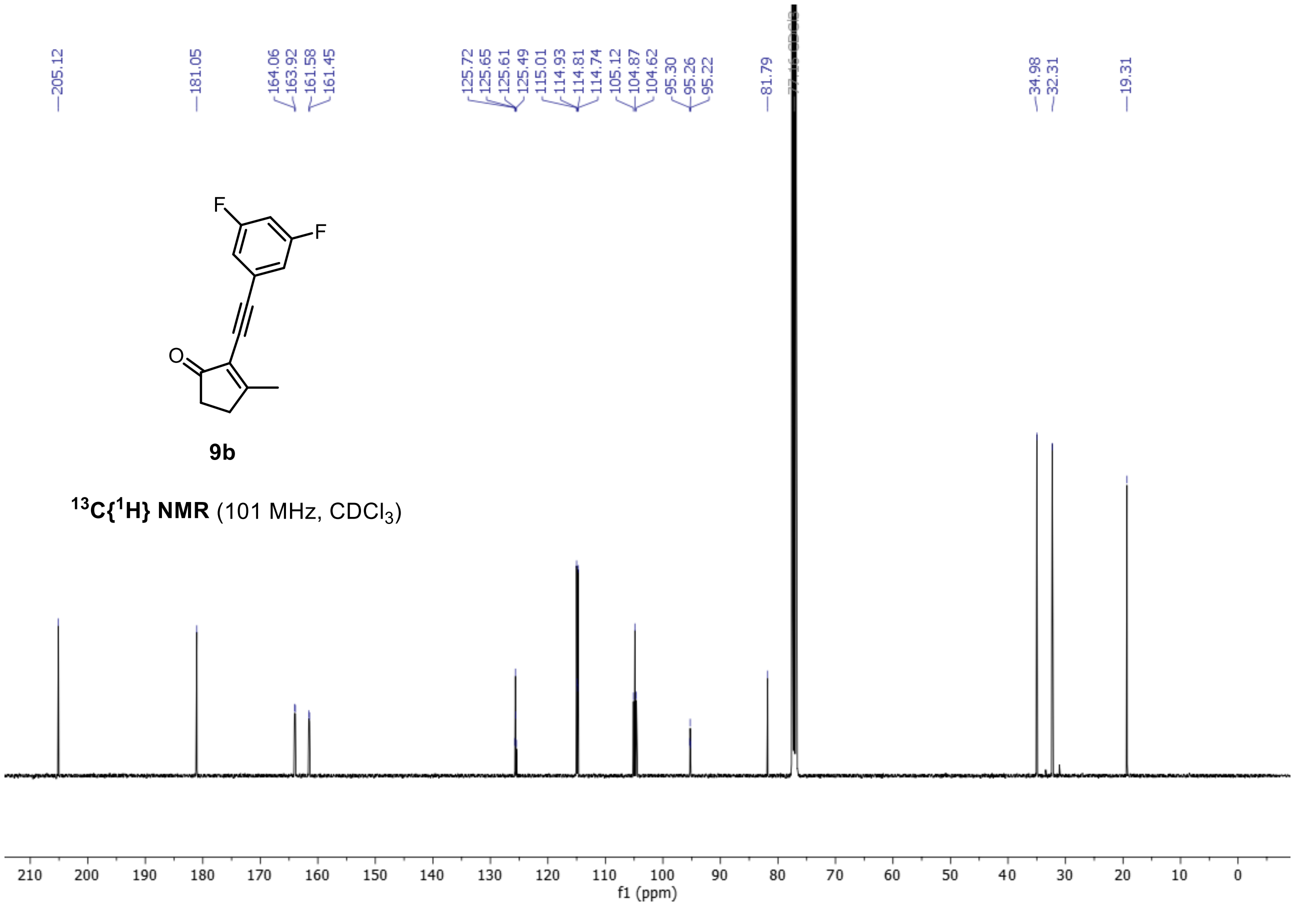

9b

^{13}C{^{1}H} NMR (101 MHz, $CDCl_3$)

9b

$^{19}F\{^1H\}$ NMR (376 MHz, $CDCl_3$)

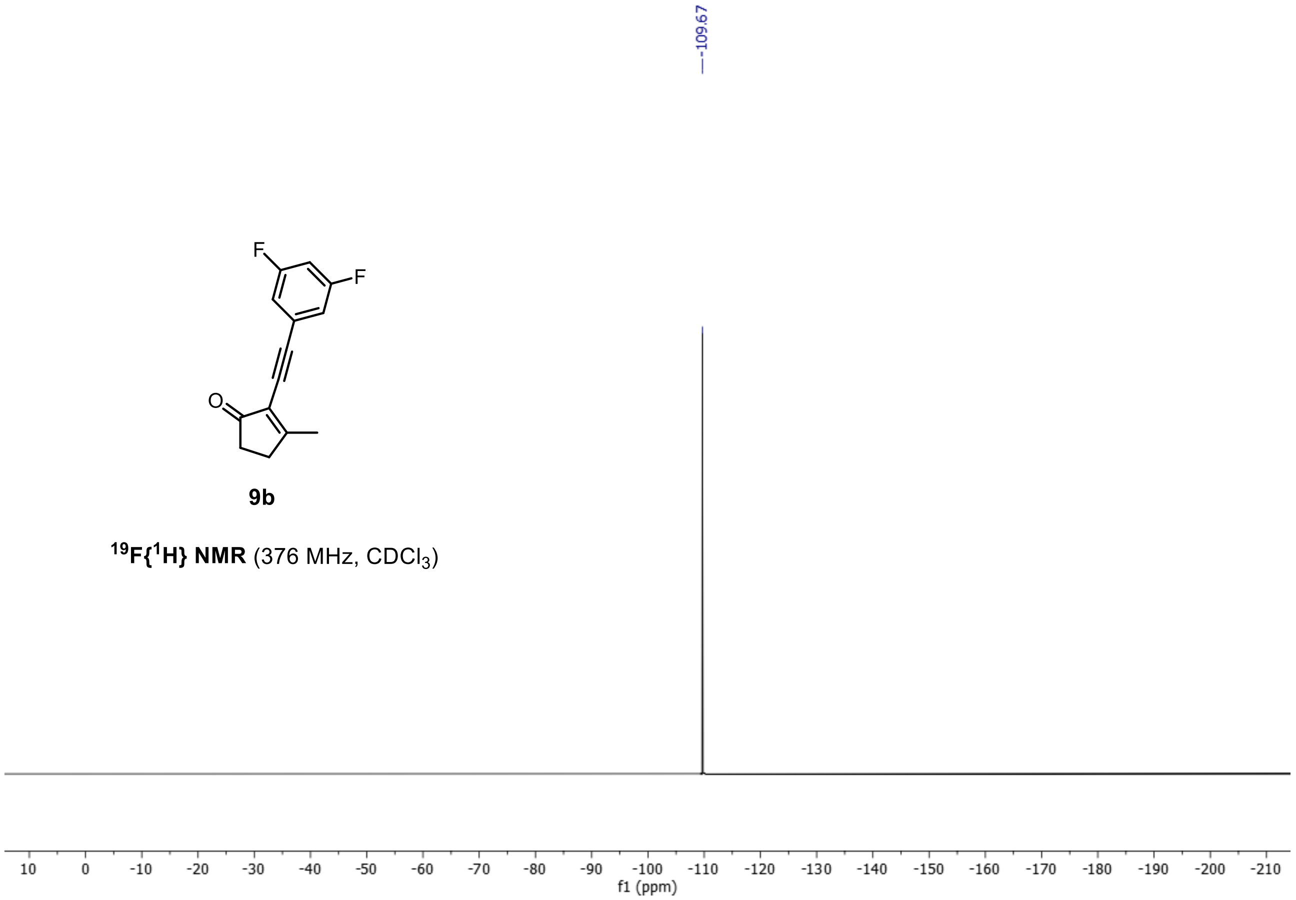

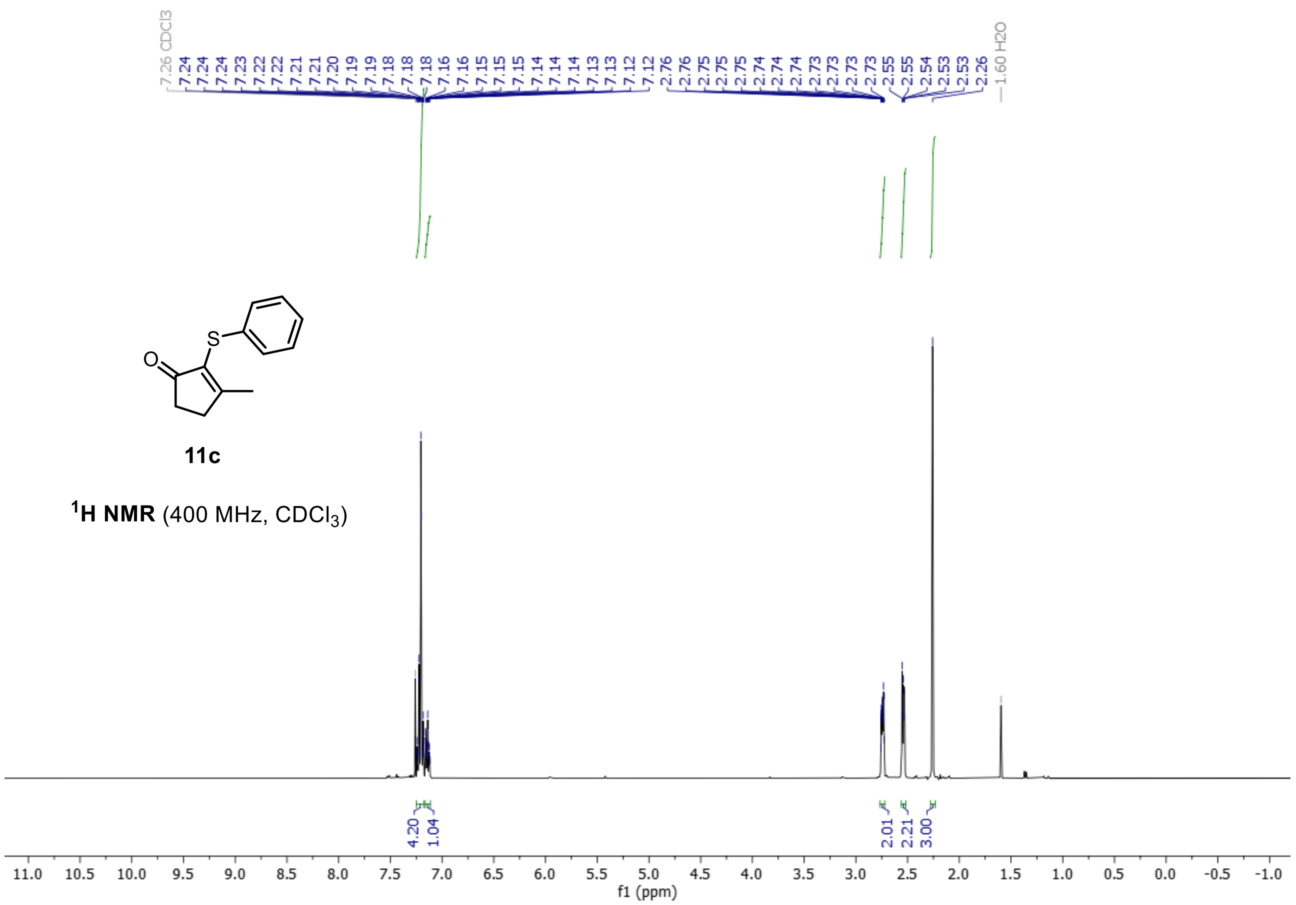

11c
^{1}H NMR (400 MHz, $CDCl_3$)
7.26 CDCl3
1.60 H2O
4.20
1.04
2.01
2.21
3.00
f1 (ppm)

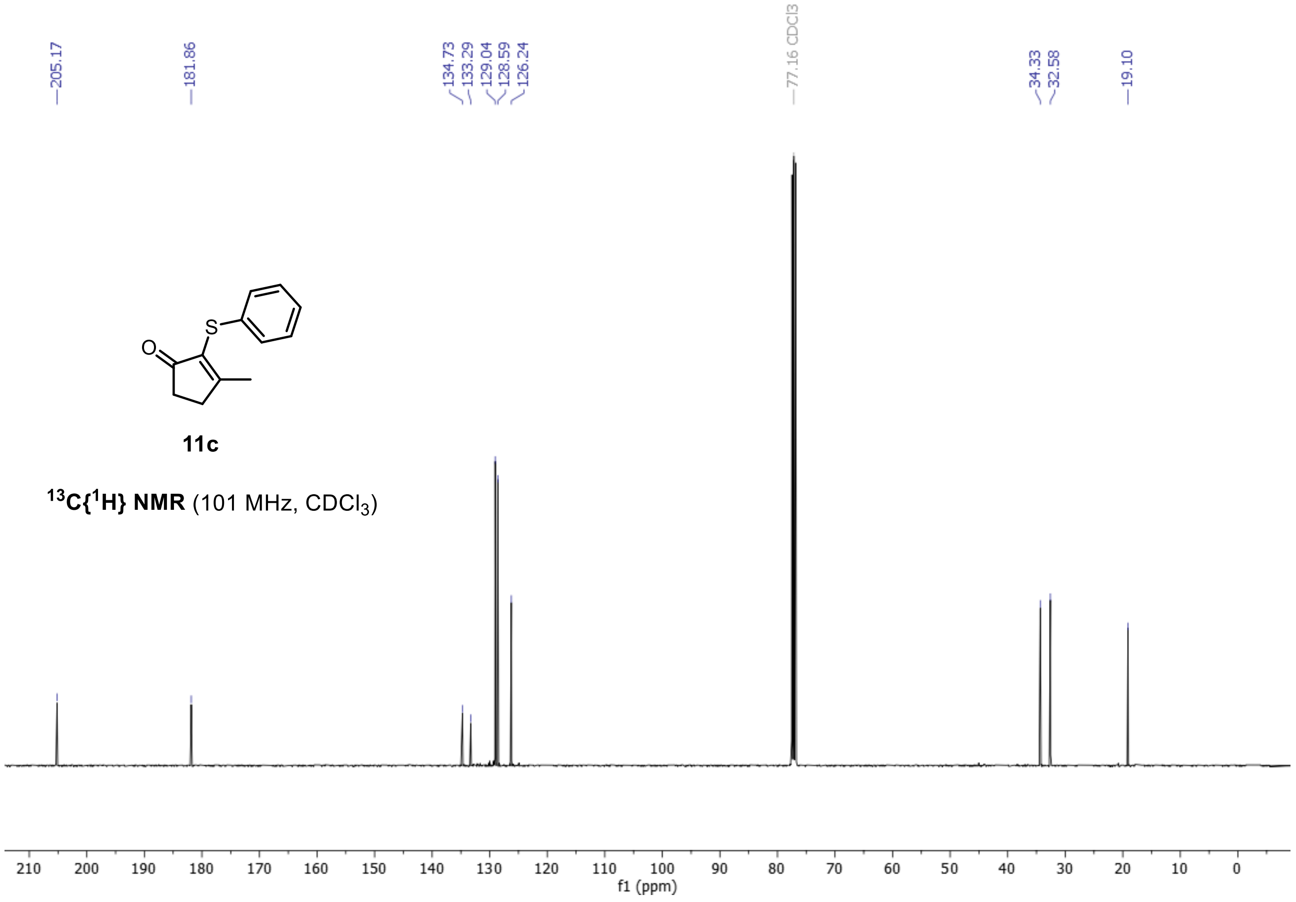

11c
^{13}C{^{1}H} NMR (101 MHz, $CDCl_3$)
205.17
181.86
134.73
133.29
129.04
128.59
126.24
77.16 CDCl3
34.33
32.58
19.10
f1 (ppm)

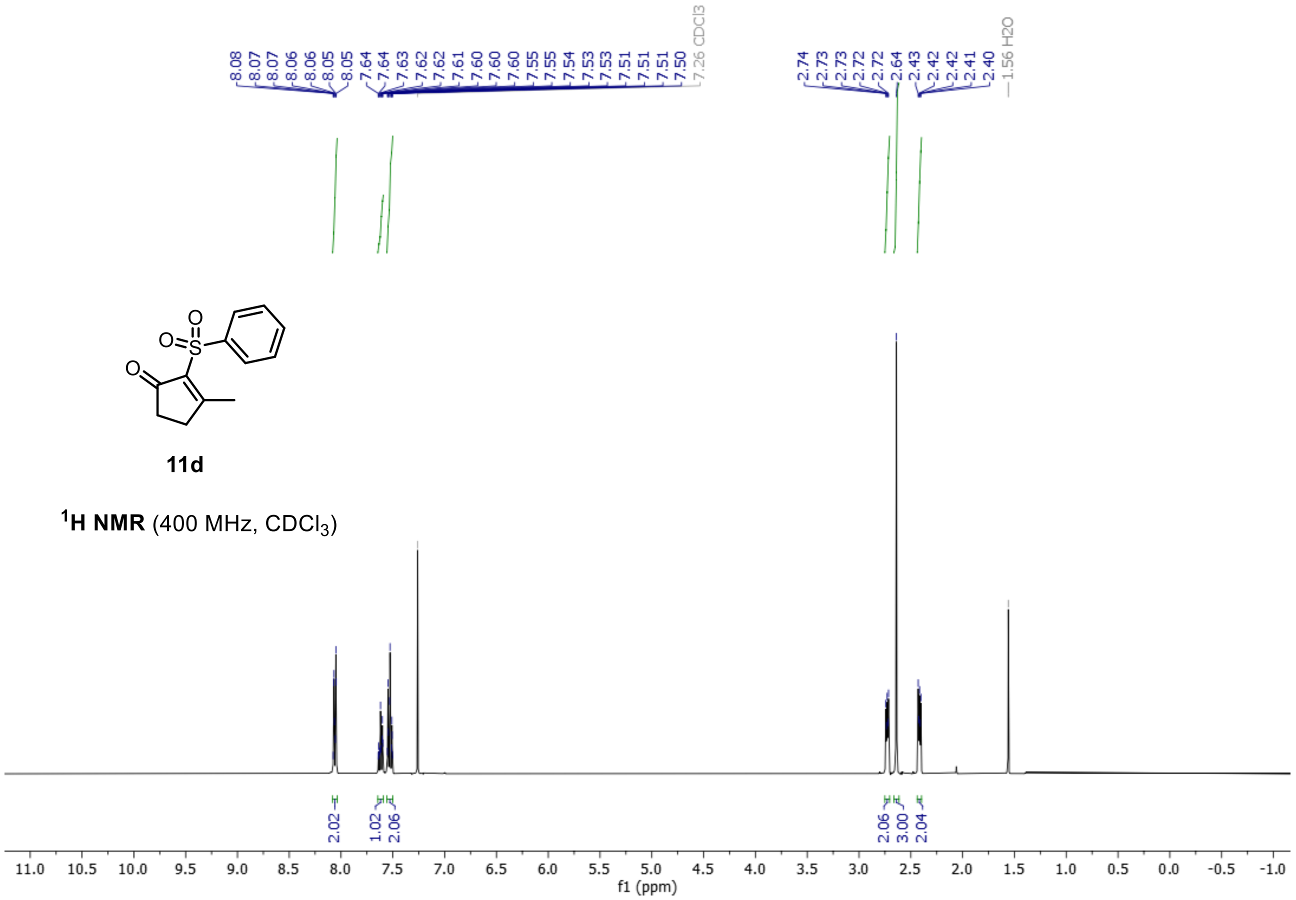
8.08
8.07
8.07
8.06
8.06
8.05
8.05
7.64
7.64
7.63
7.62
7.62
7.61
7.60
7.60
7.60
7.55
7.55
7.54
7.53
7.53
7.51
7.51
7.51
7.50
7.26 CDCl3
2.74
2.73
2.73
2.72
2.72
2.64
2.43
2.42
2.42
2.41
2.40
1.56 H2O
11d
1H NMR (400 MHz, CDCl3)
2.02
1.02
2.06
2.06
3.00
2.04
f1 (ppm)

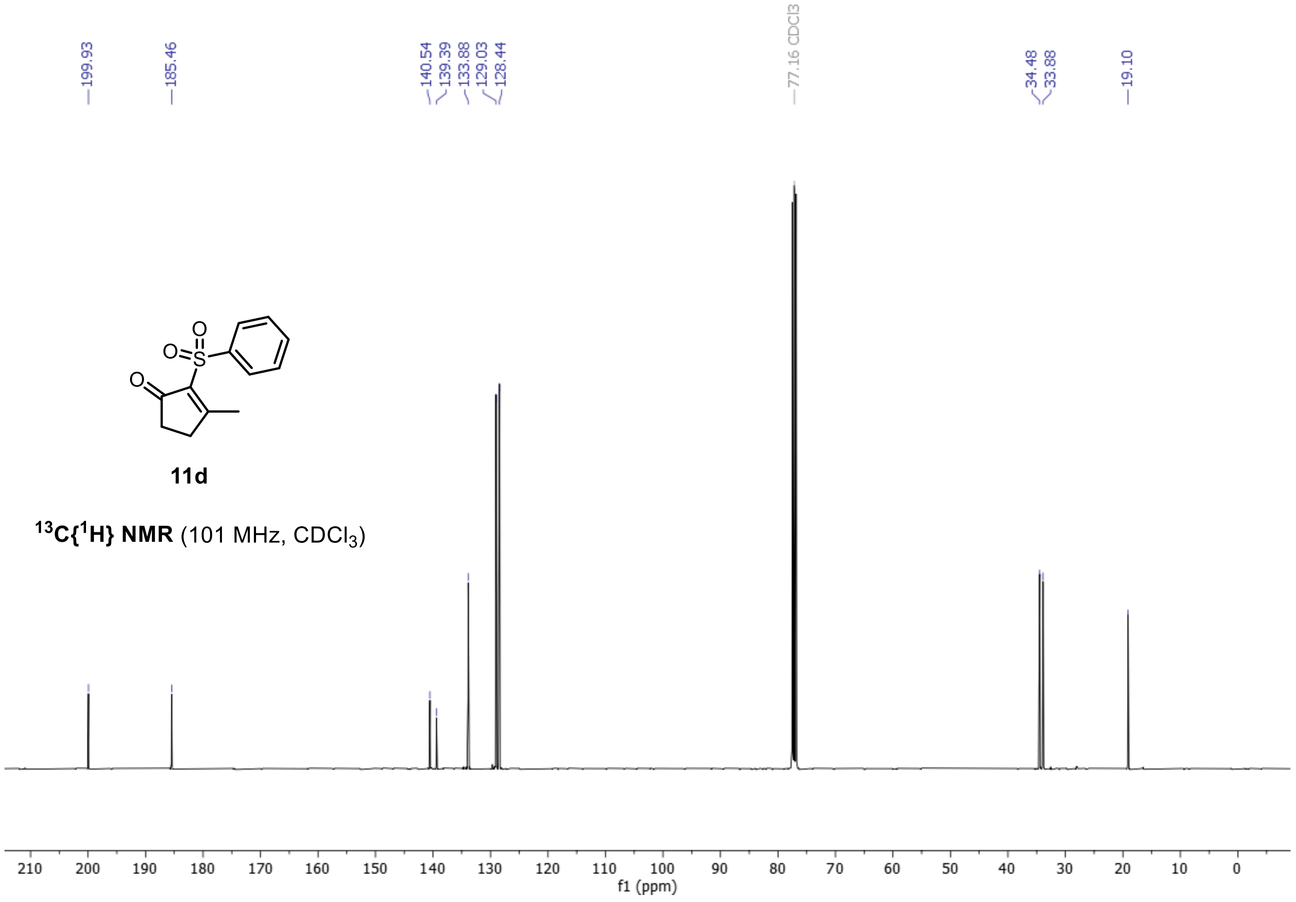
199.93
185.46
140.54
139.39
133.88
129.03
128.44
77.16 CDCl3
34.48
33.88
19.10
11d
13C{1H} NMR (101 MHz, CDCl3)
f1 (ppm)

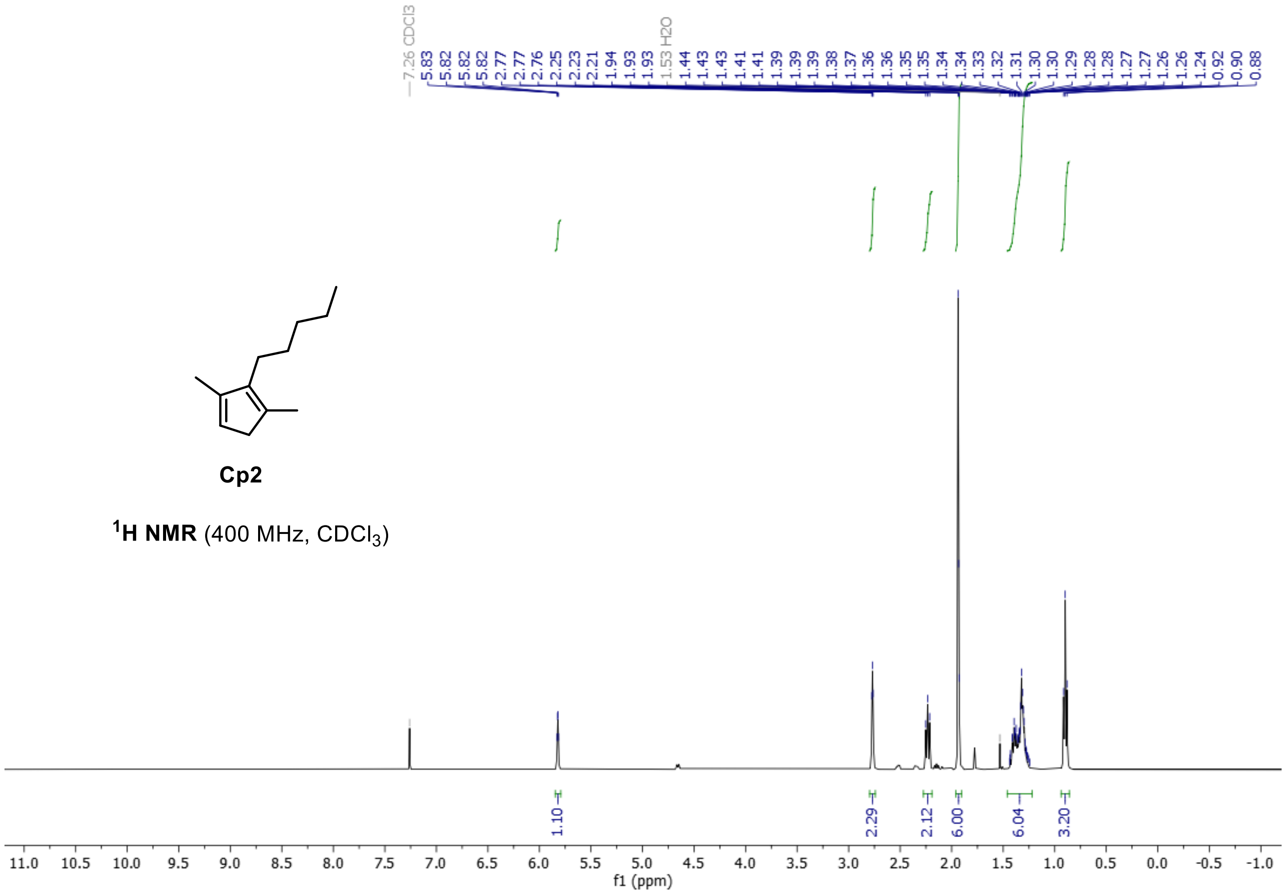

Cp2

^{1}H NMR (400 MHz, $CDCl_3$)

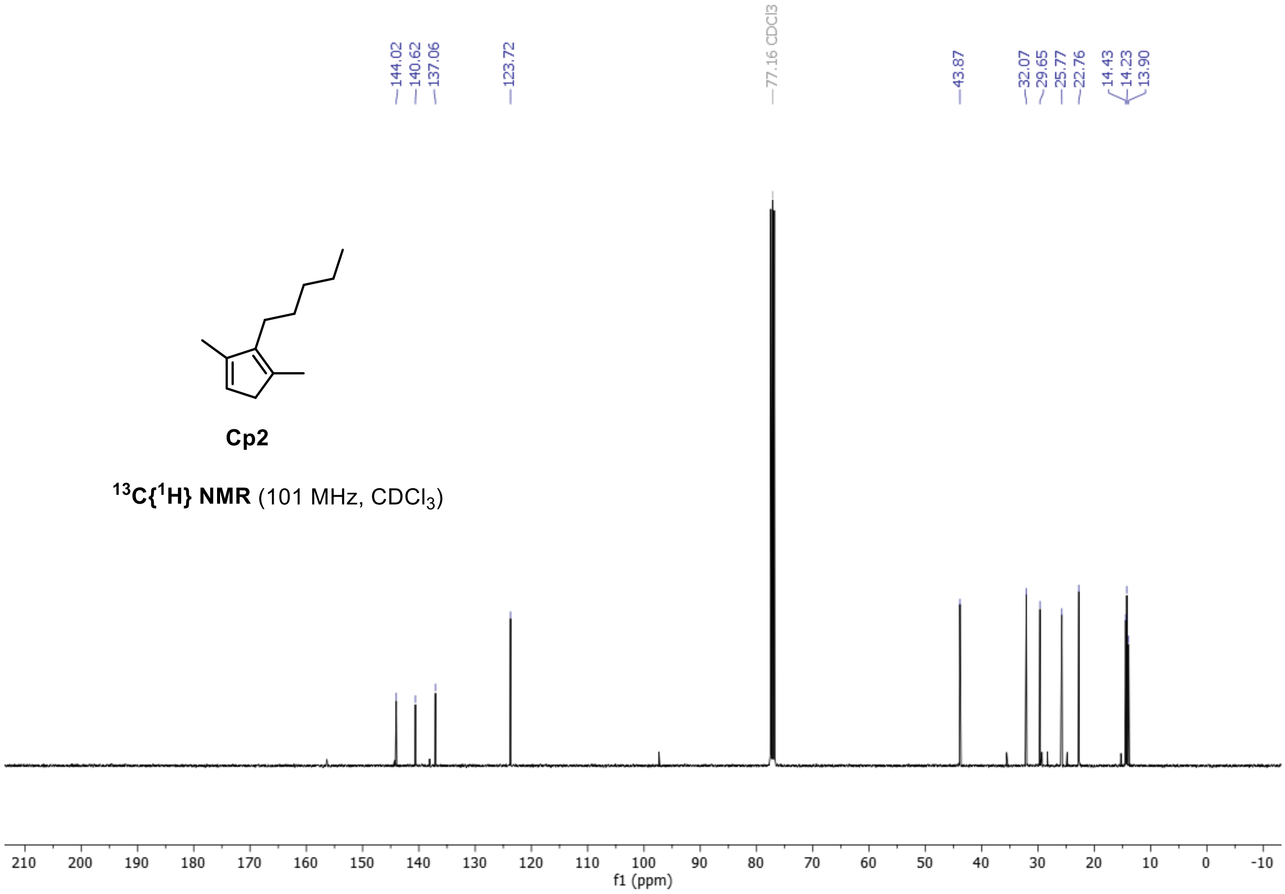

Cp2

^{13}C{^{1}H} NMR (101 MHz, $CDCl_3$)

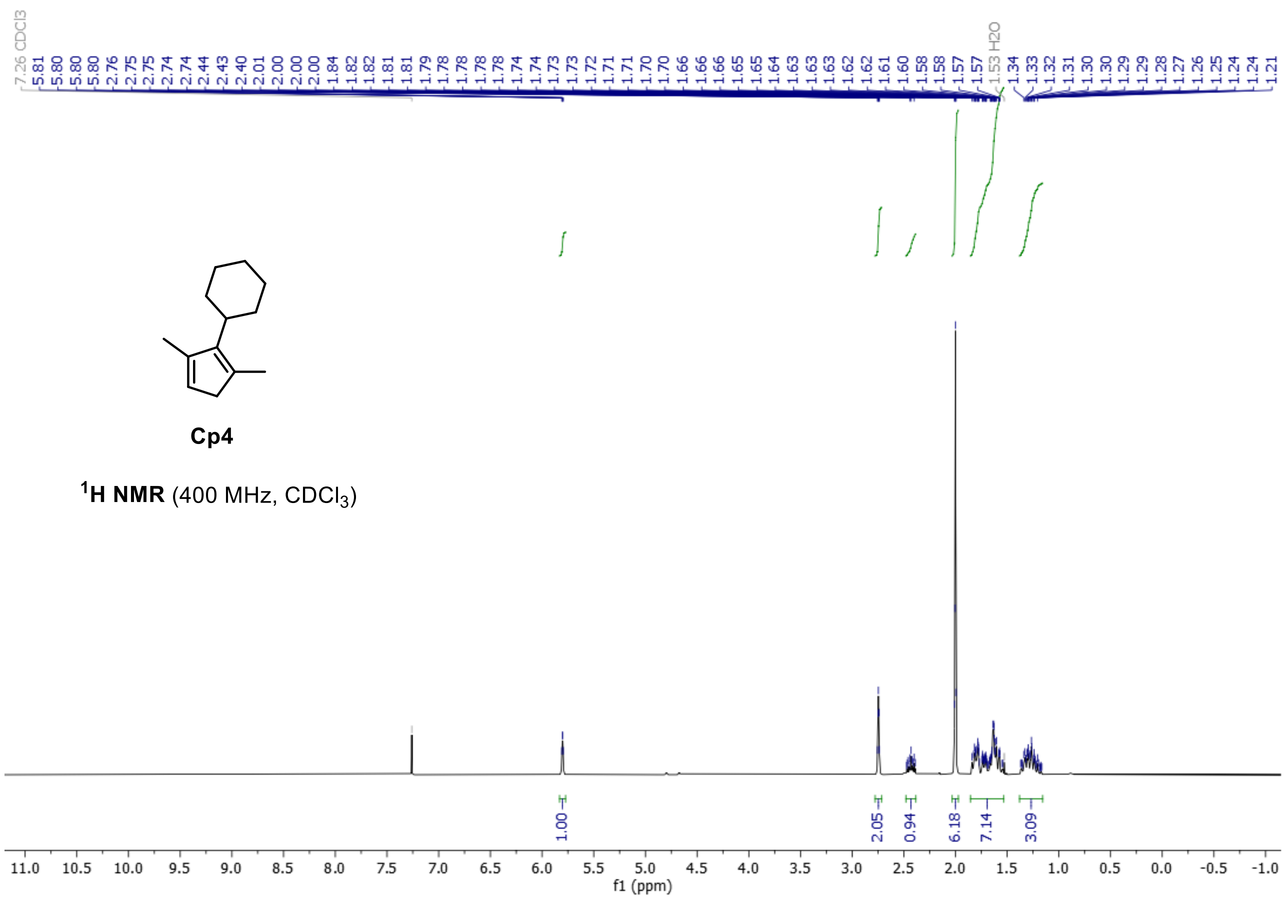

Cp4

^{1}H NMR (400 MHz, $CDCl_3$)

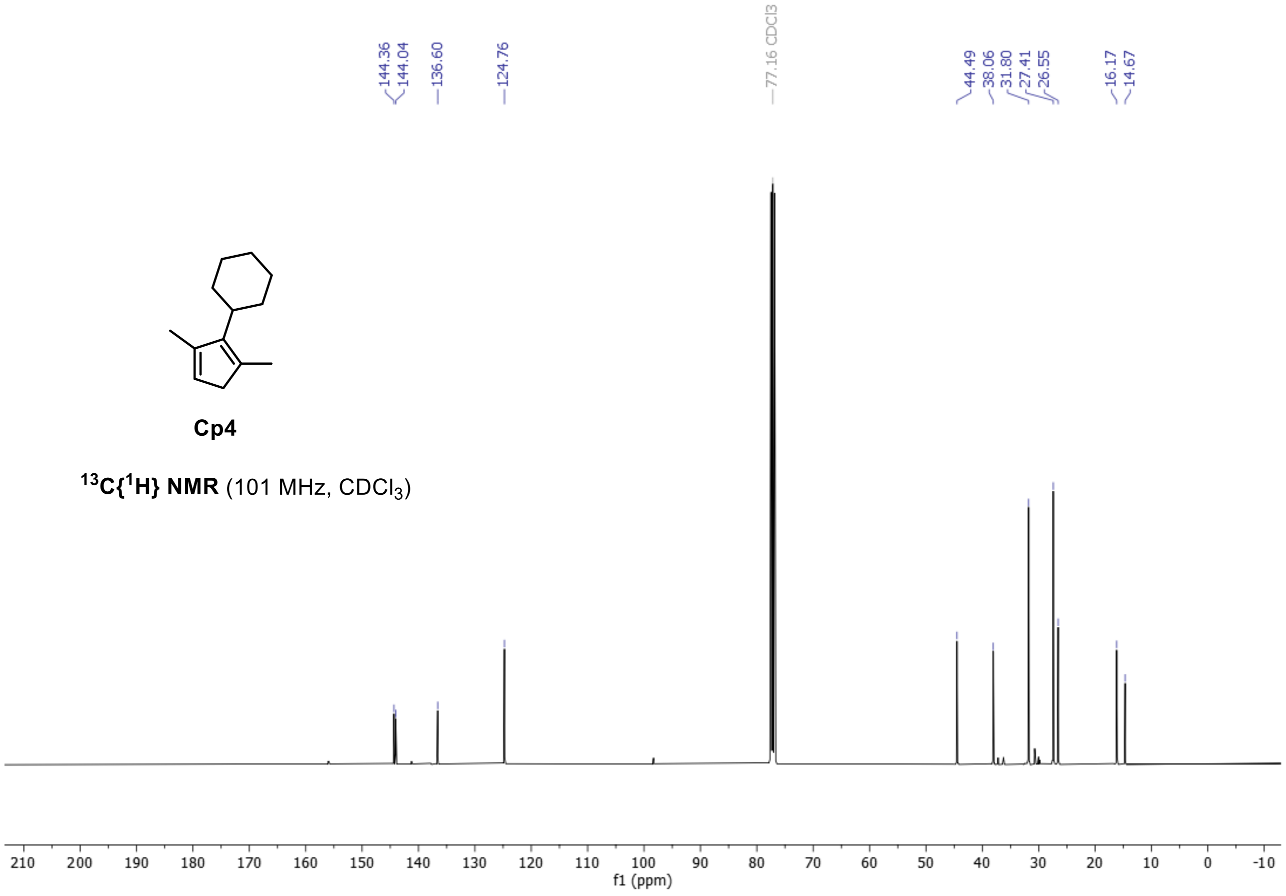

Cp4

^{13}C{^{1}H} NMR (101 MHz, $CDCl_3$)

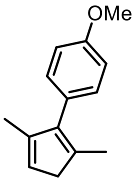

Cp7

^{1}H NMR (400 MHz, $CDCl_3$)

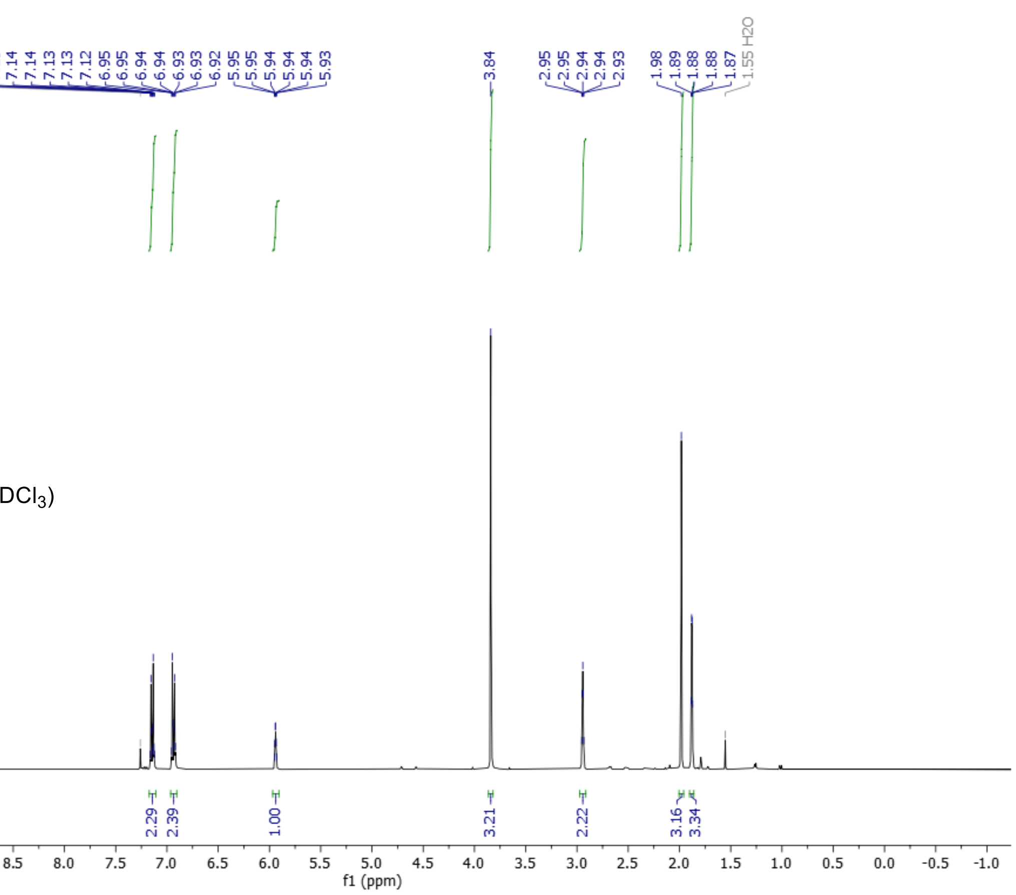

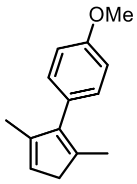

Cp7

$^{13}C\{^{1}H\}$ NMR (101 MHz, $CDCl_3$)

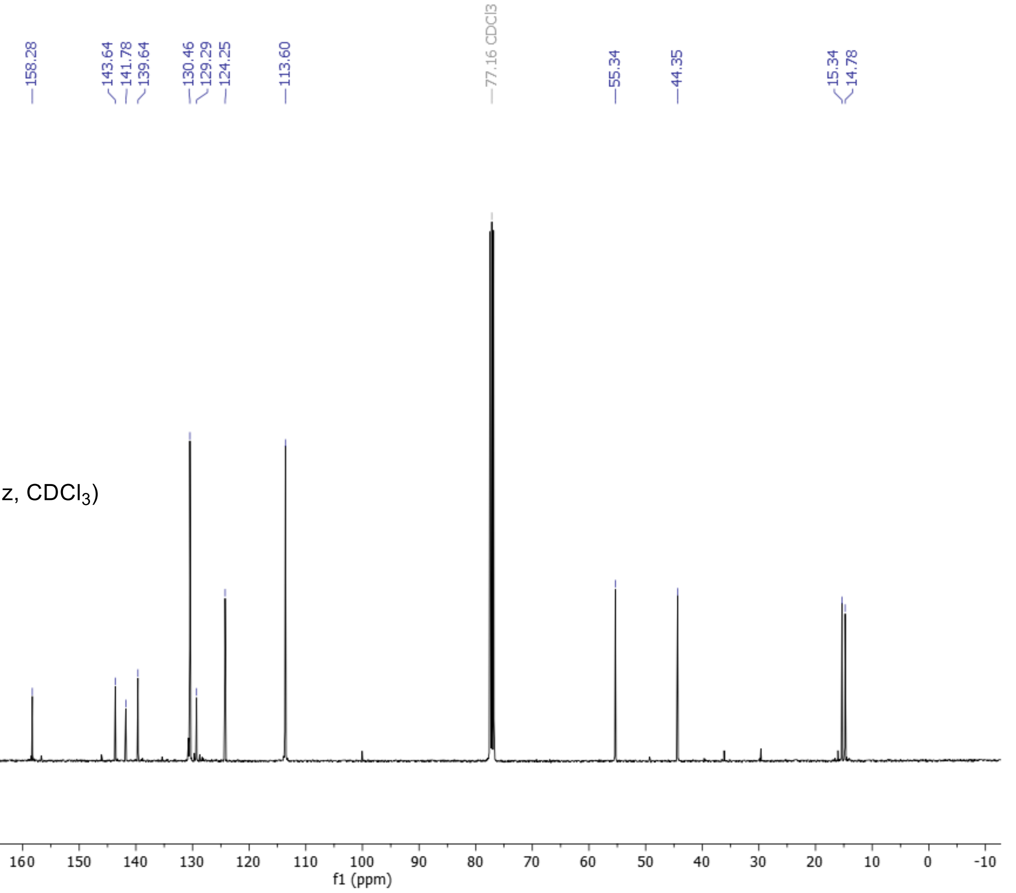

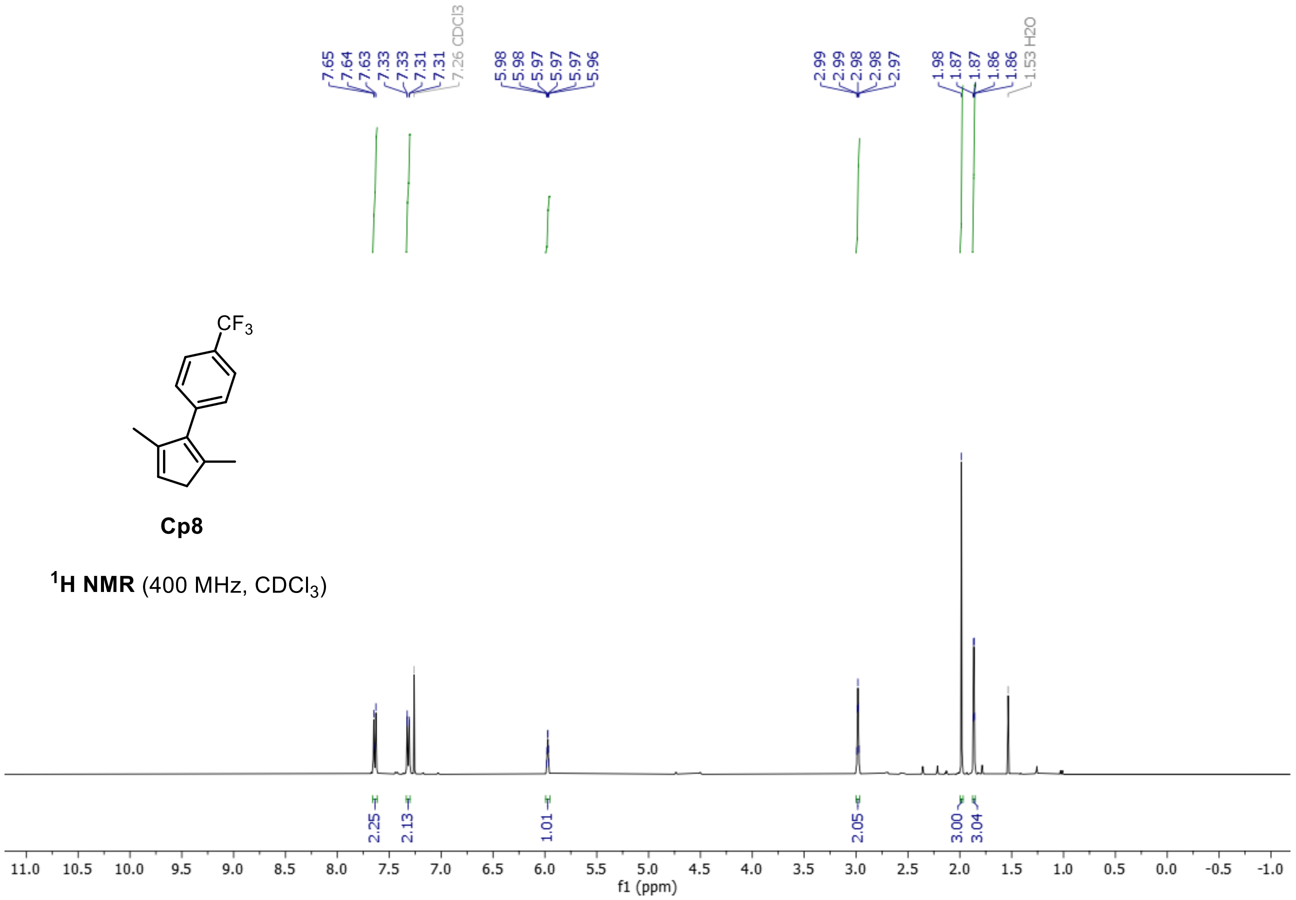

Cp8

^{1}H NMR (400 MHz, $CDCl_3$)

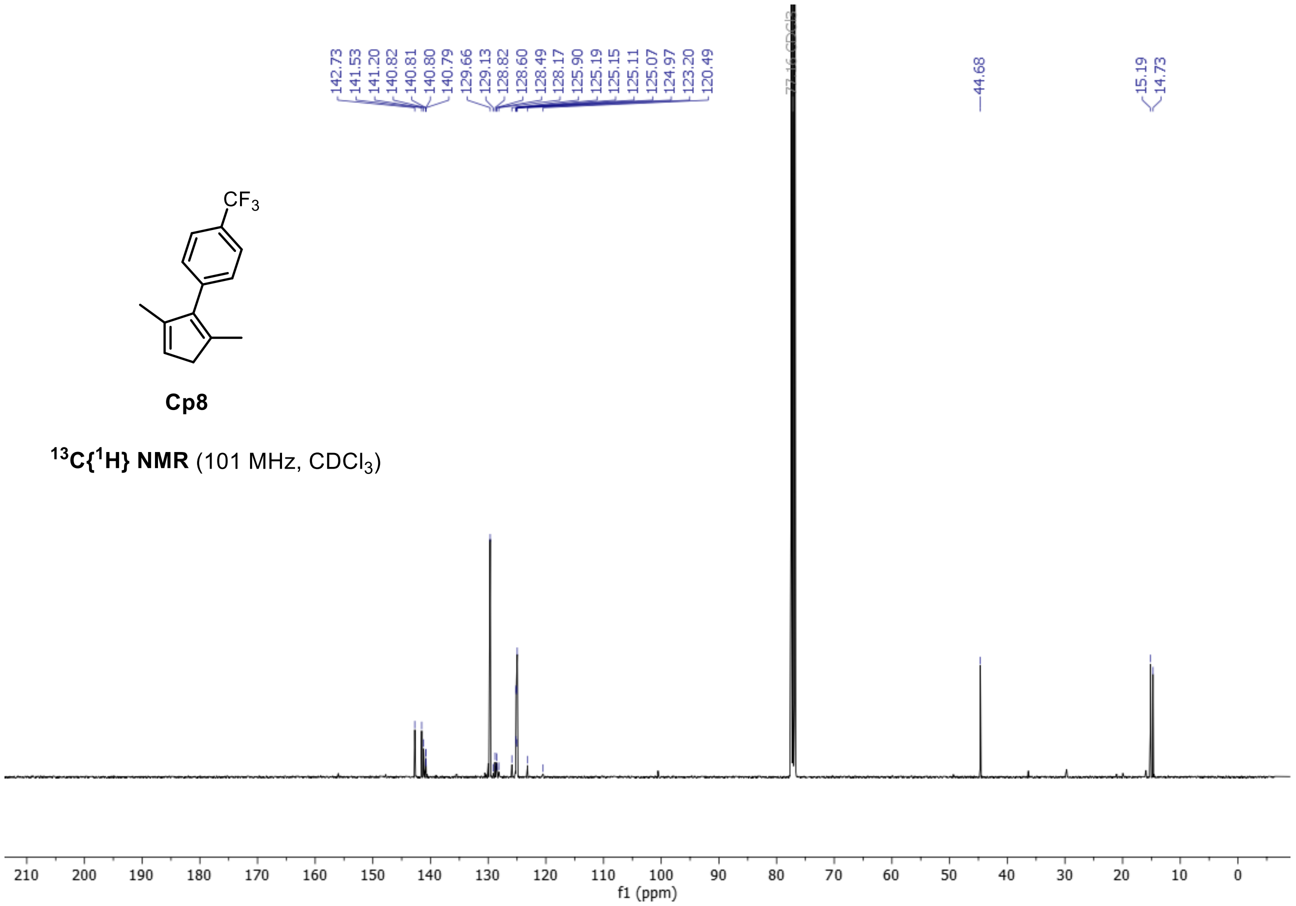

Cp8

^{13}C{^{1}H} NMR (101 MHz, $CDCl_3$)

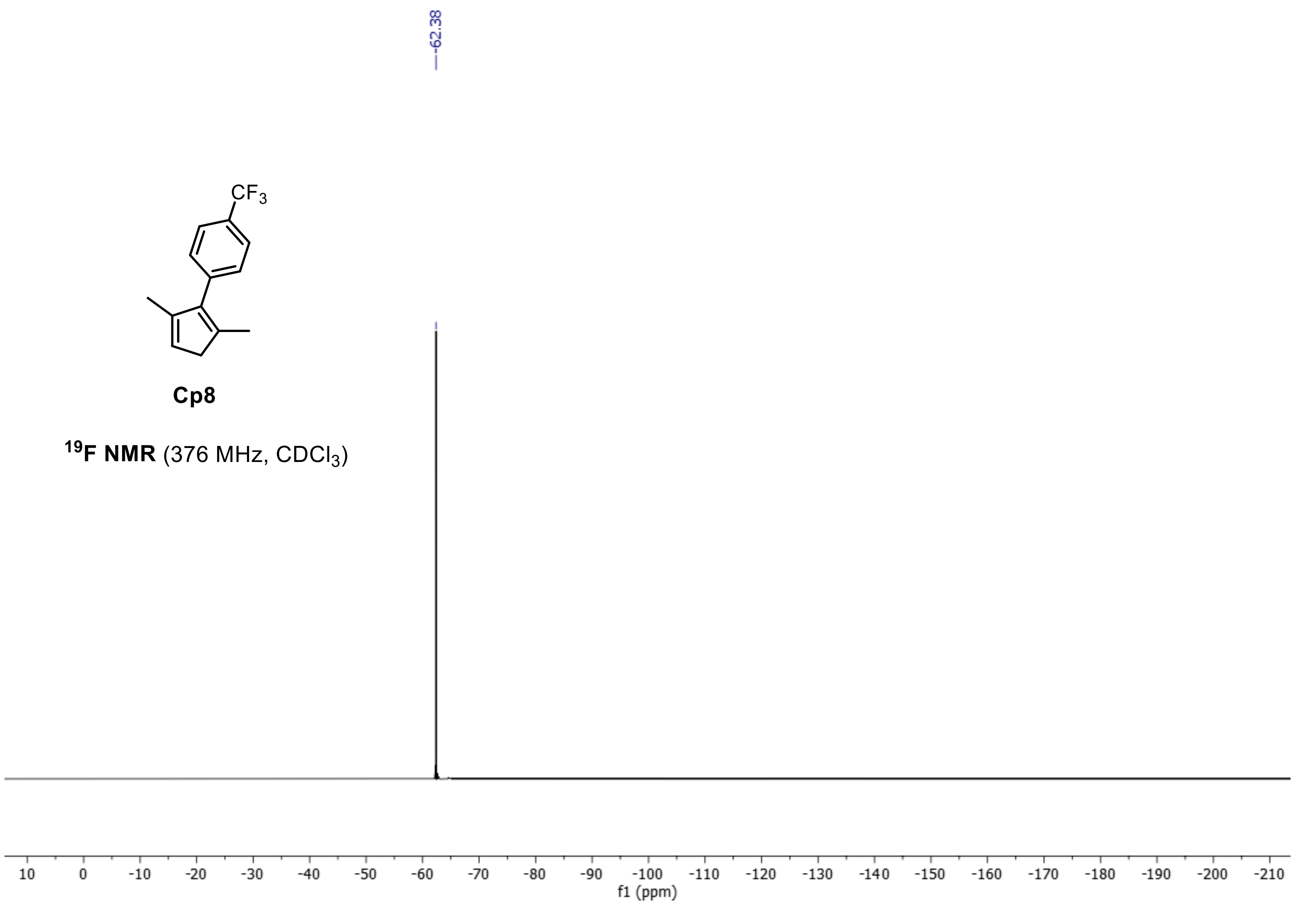
-62.38
CF3
Cp8
19F NMR (376 MHz, CDCl3)
10
0
-10
-20
-30
-40
-50
-60
-70
-80
-90
-100
-110
-120
-130
-140
-150
-160
-170
-180
-190
-200
-210
f1 (ppm)

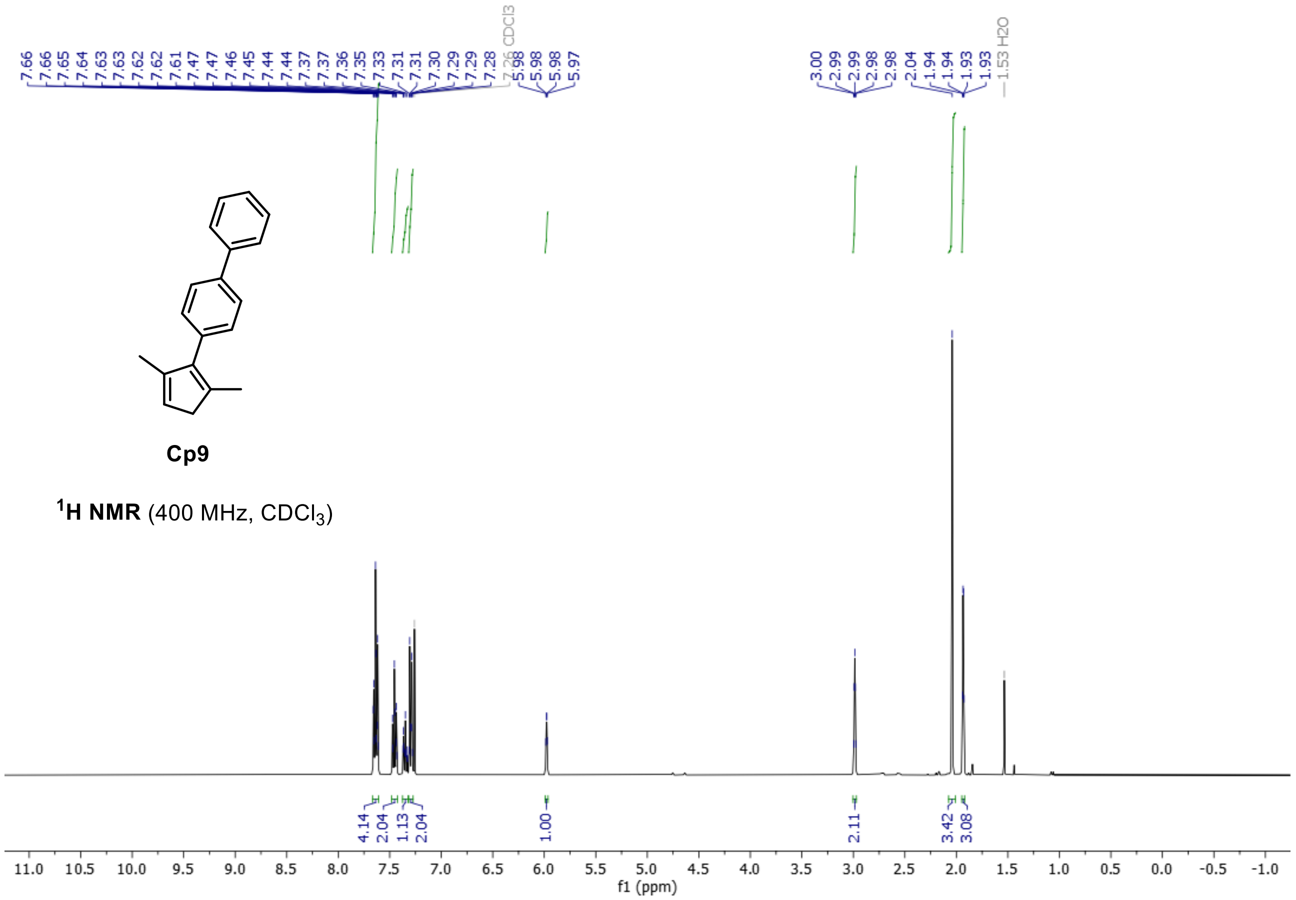
Cp9
^{1}H NMR (400 MHz, CDCl$_3$)
7.66 7.66 7.65 7.64 7.63 7.63 7.62 7.62 7.61 7.47 7.47 7.46 7.45 7.44 7.44 7.37 7.37 7.36 7.35 7.33 7.31 7.31 7.30 7.29 7.29 7.28 7.26 CDCl3 5.98 5.98 5.98 5.97
3.00 2.99 2.99 2.98 2.98 2.04 1.94 1.94 1.93 1.93 1.53 H2O
4.14 2.04 1.13 2.04 1.00 2.11 3.42 3.08
f1 (ppm)

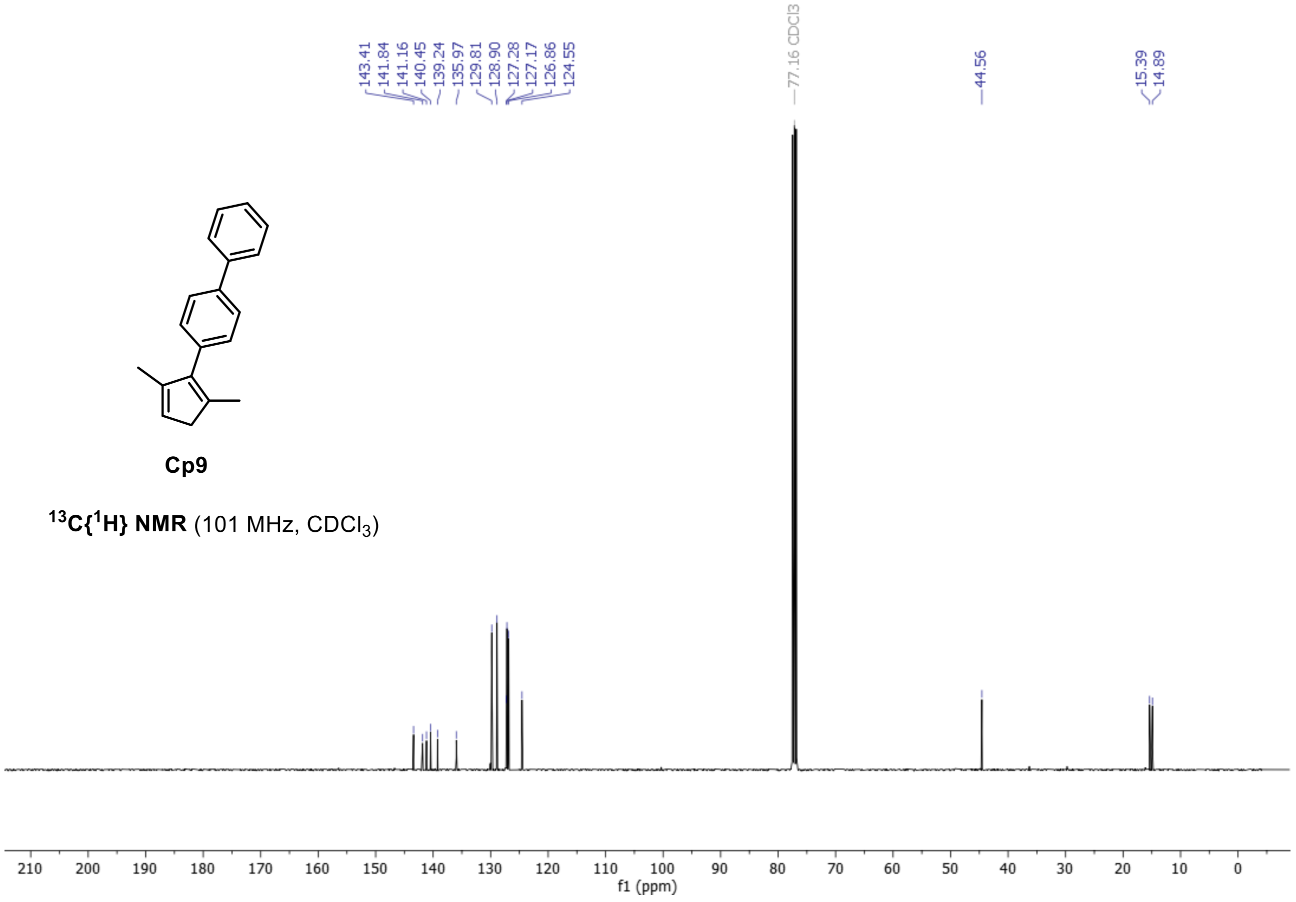
Cp9
^{13}C{^{1}H} NMR (101 MHz, CDCl$_3$)
143.41 141.84 141.16 140.45 139.24 135.97 129.81 128.90 127.28 127.17 126.86 124.55
77.16 CDCl3
44.56
15.39 14.89
f1 (ppm)

Cp10

^{1}H NMR (400 MHz, $CDCl_3$)

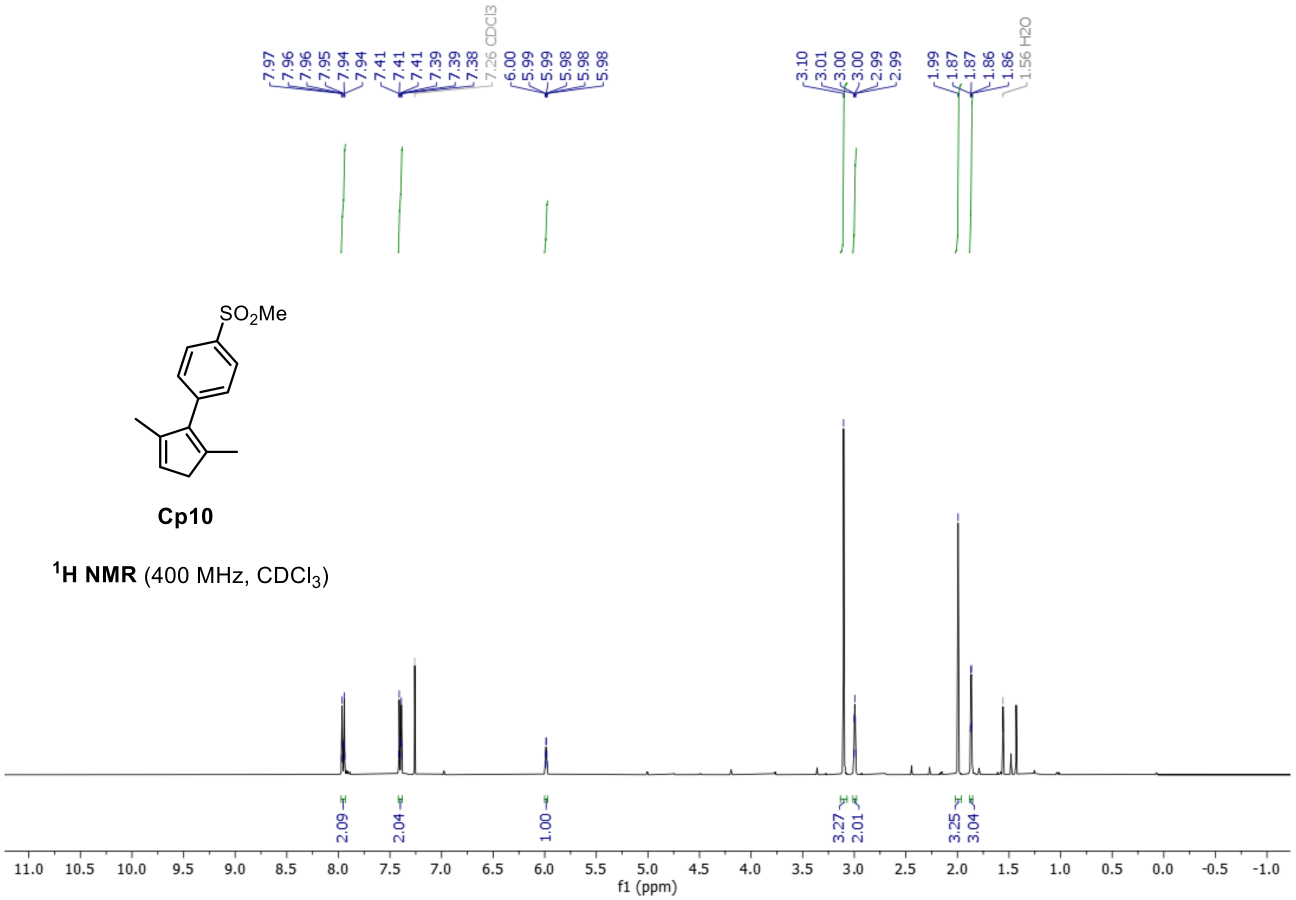

Cp10

^{13}C{^{1}H} NMR (101 MHz, $CDCl_3$)

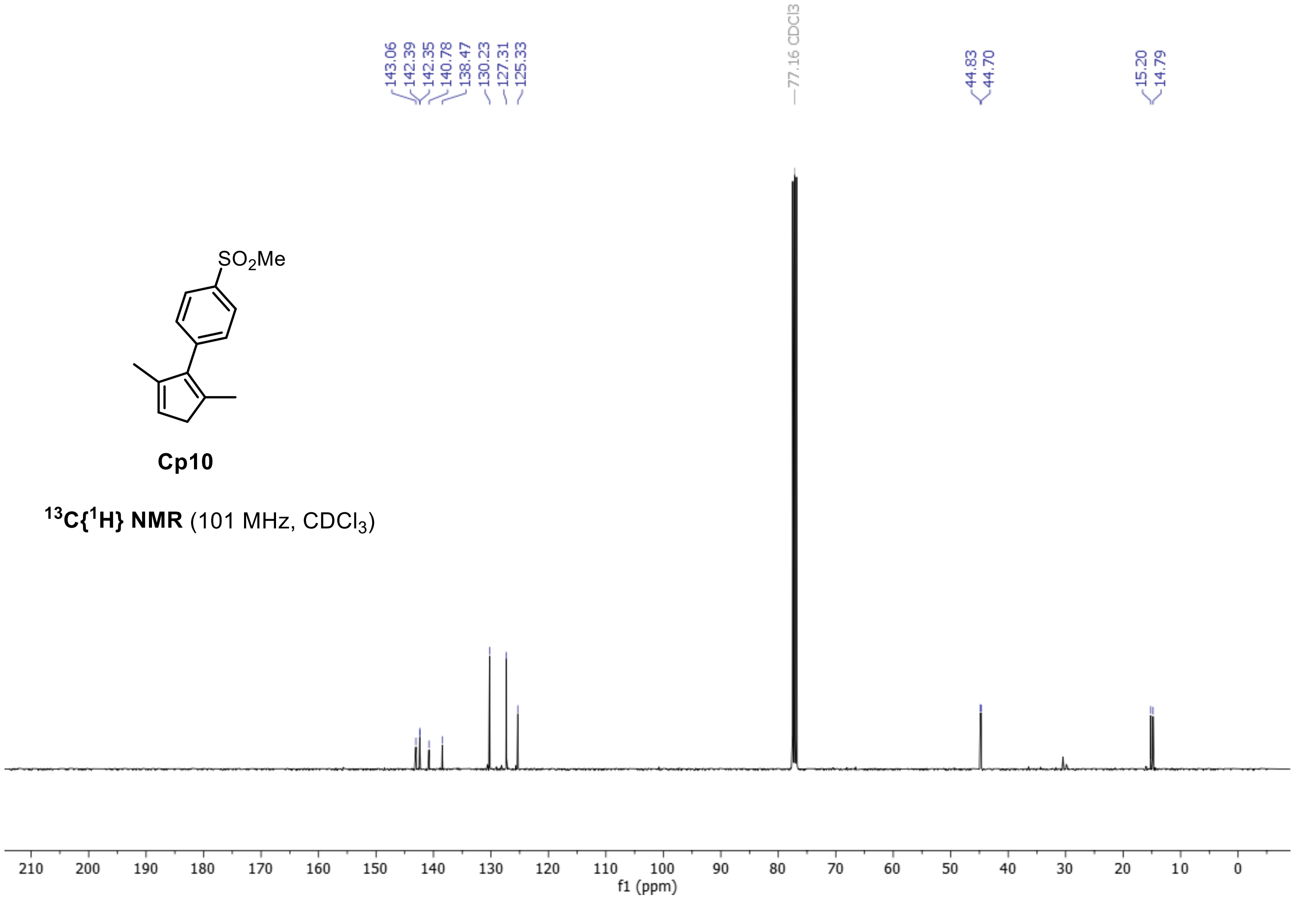

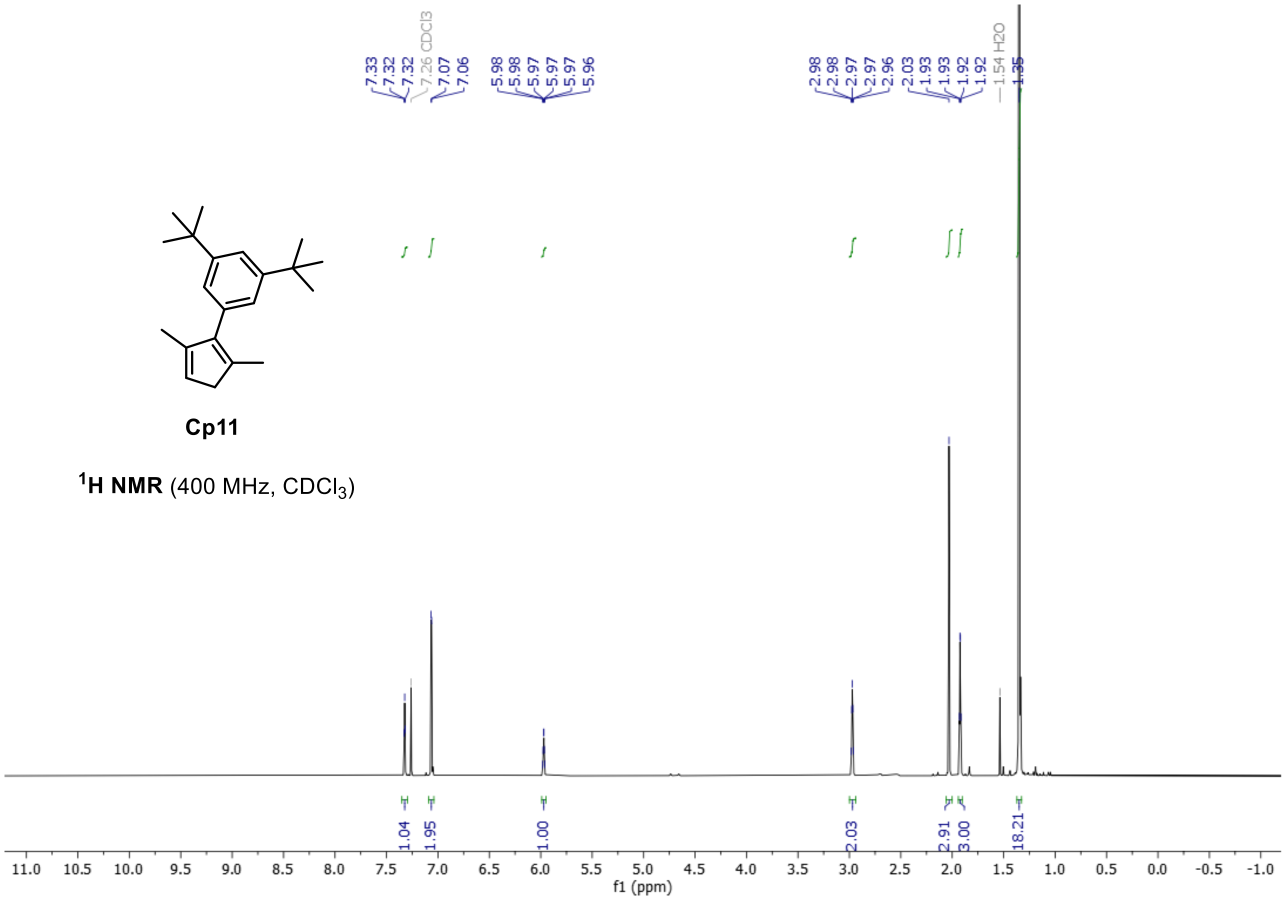
7.33
7.32
7.32
7.26 CDCl3
7.07
7.06
5.98
5.98
5.97
5.97
5.97
5.96
2.98
2.98
2.97
2.97
2.96
2.03
1.93
1.93
1.92
1.92
1.54 H2O
1.35
Cp11
^1^H NMR (400 MHz, CDCl3)
1.04
1.95
1.00
2.03
2.91
3.00
18.21
f1 (ppm)

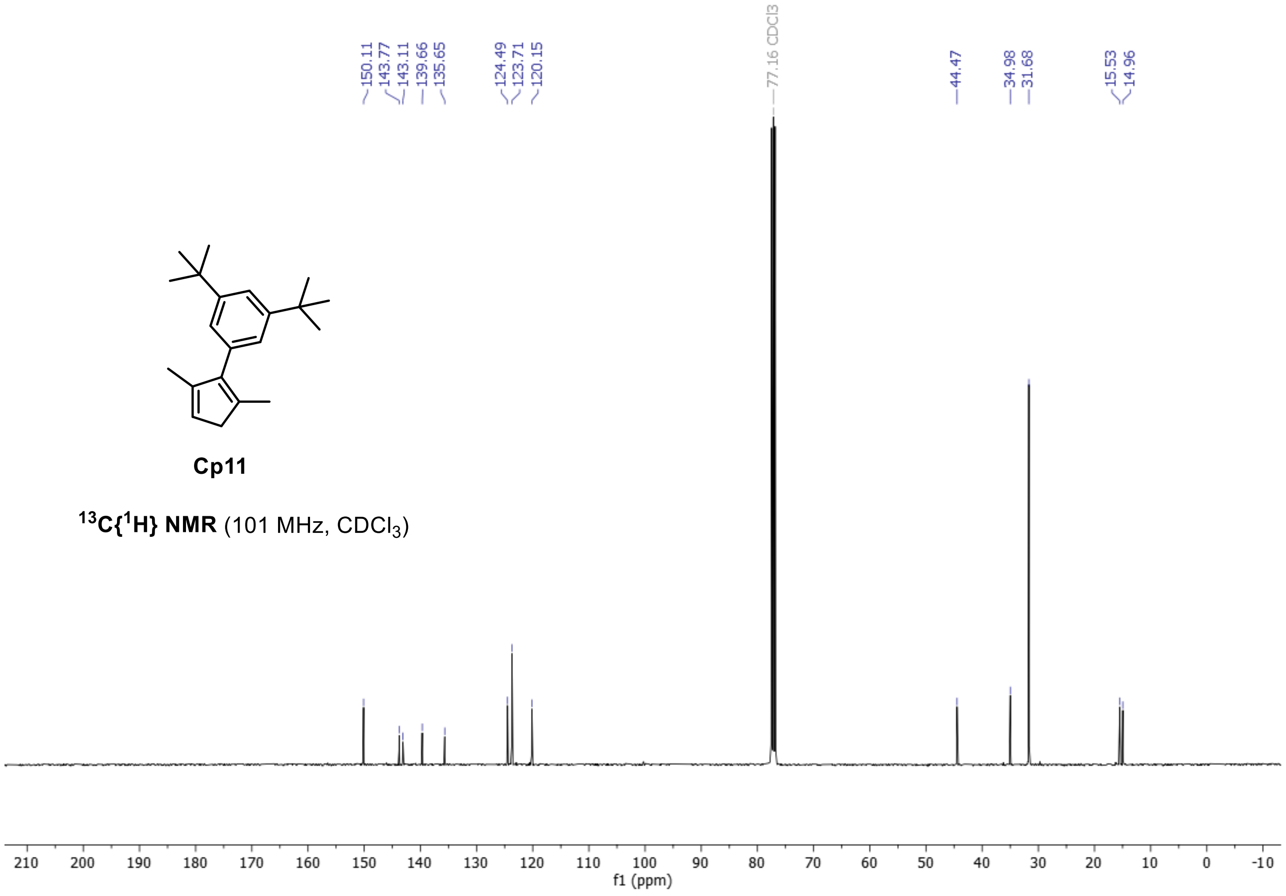
150.11
143.77
143.11
139.66
135.65
124.49
123.71
120.15
77.16 CDCl3
44.47
34.98
31.68
15.53
14.96
Cp11
^13^C{^1^H} NMR (101 MHz, CDCl3)
f1 (ppm)

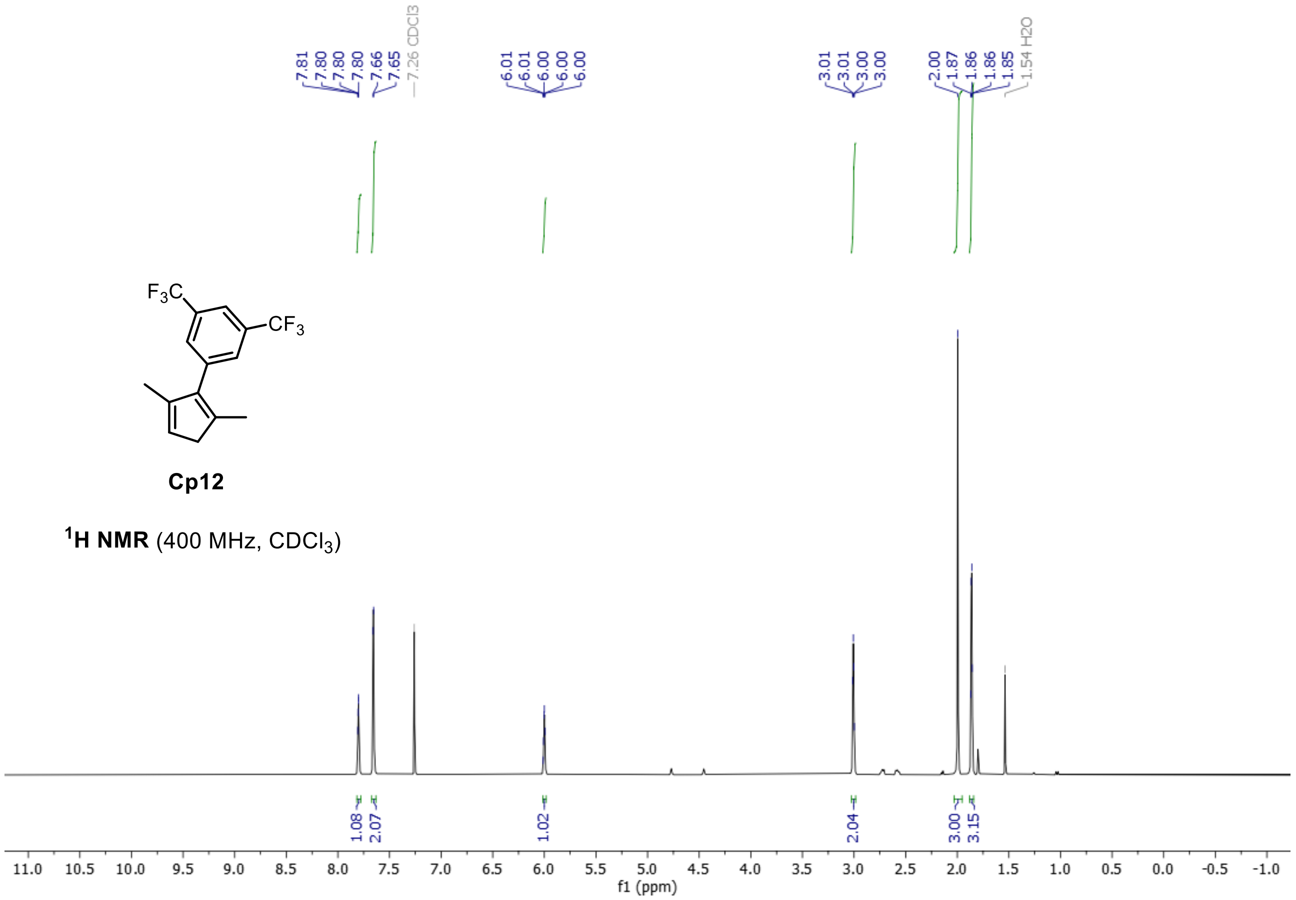
Cp12
1H NMR (400 MHz, CDCl3)
f1 (ppm)

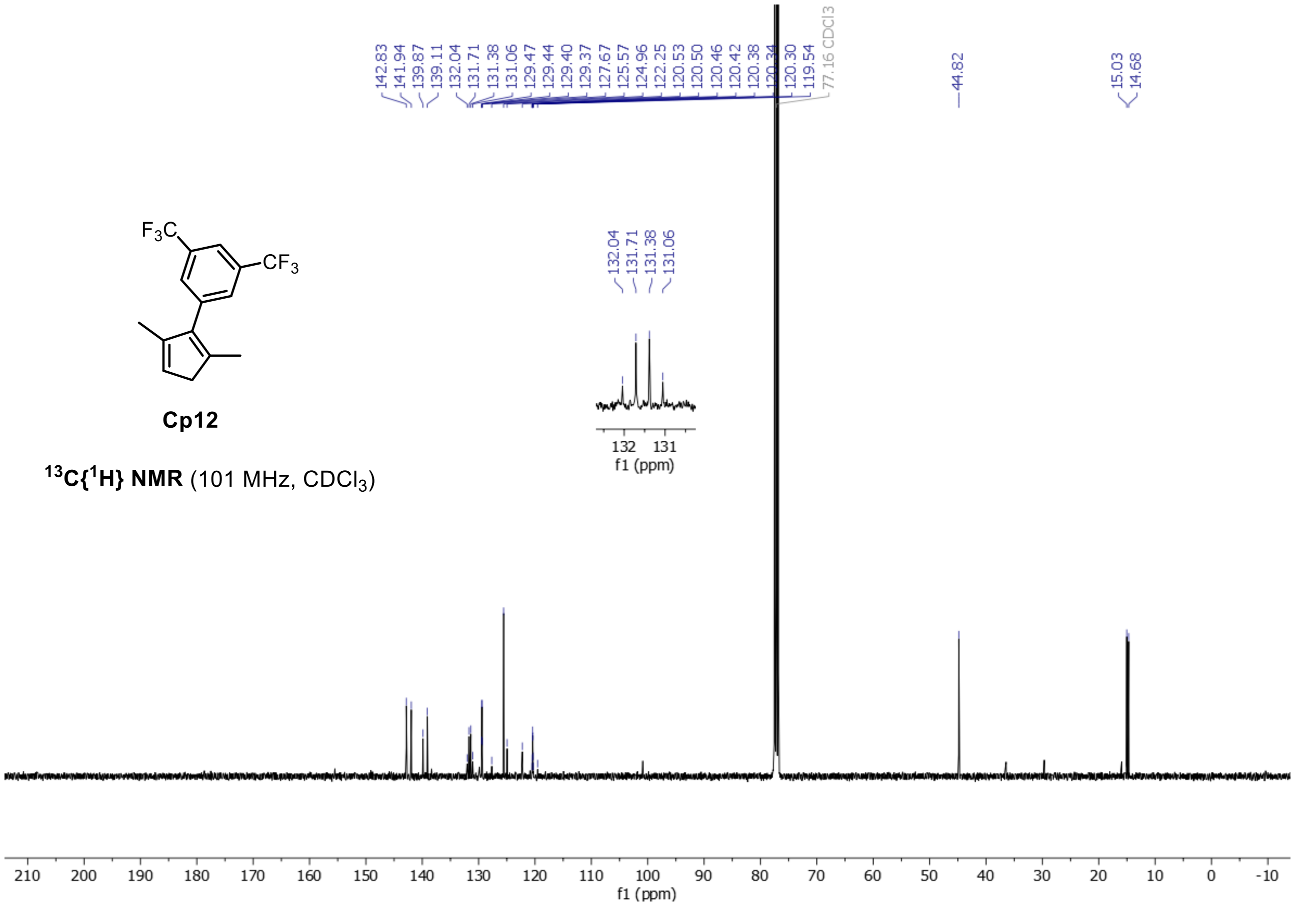
Cp12
13C{1H} NMR (101 MHz, CDCl3)
f1 (ppm)

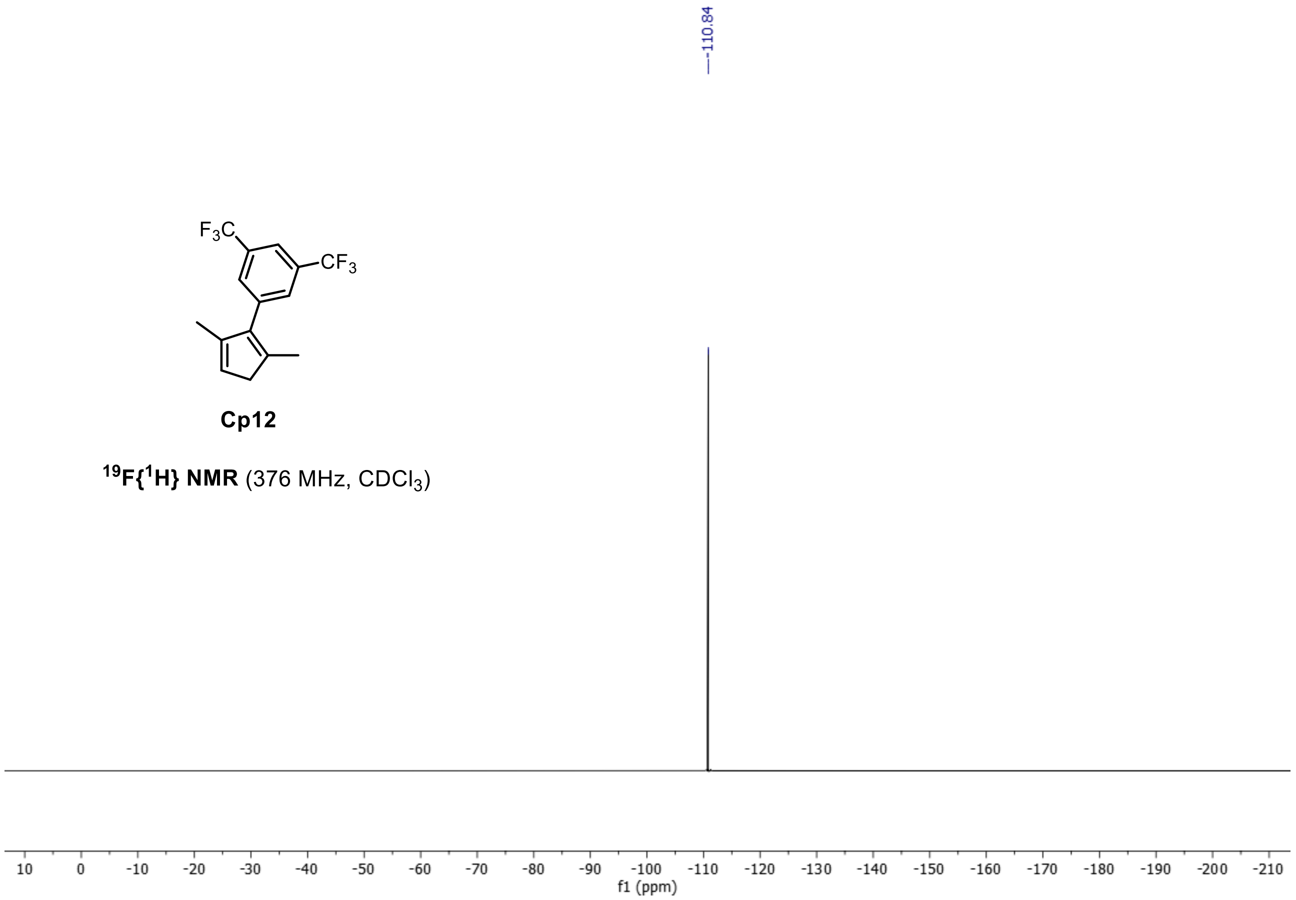

Cp12

^{19}F{^{1}H} NMR (376 MHz, $CDCl_3$)

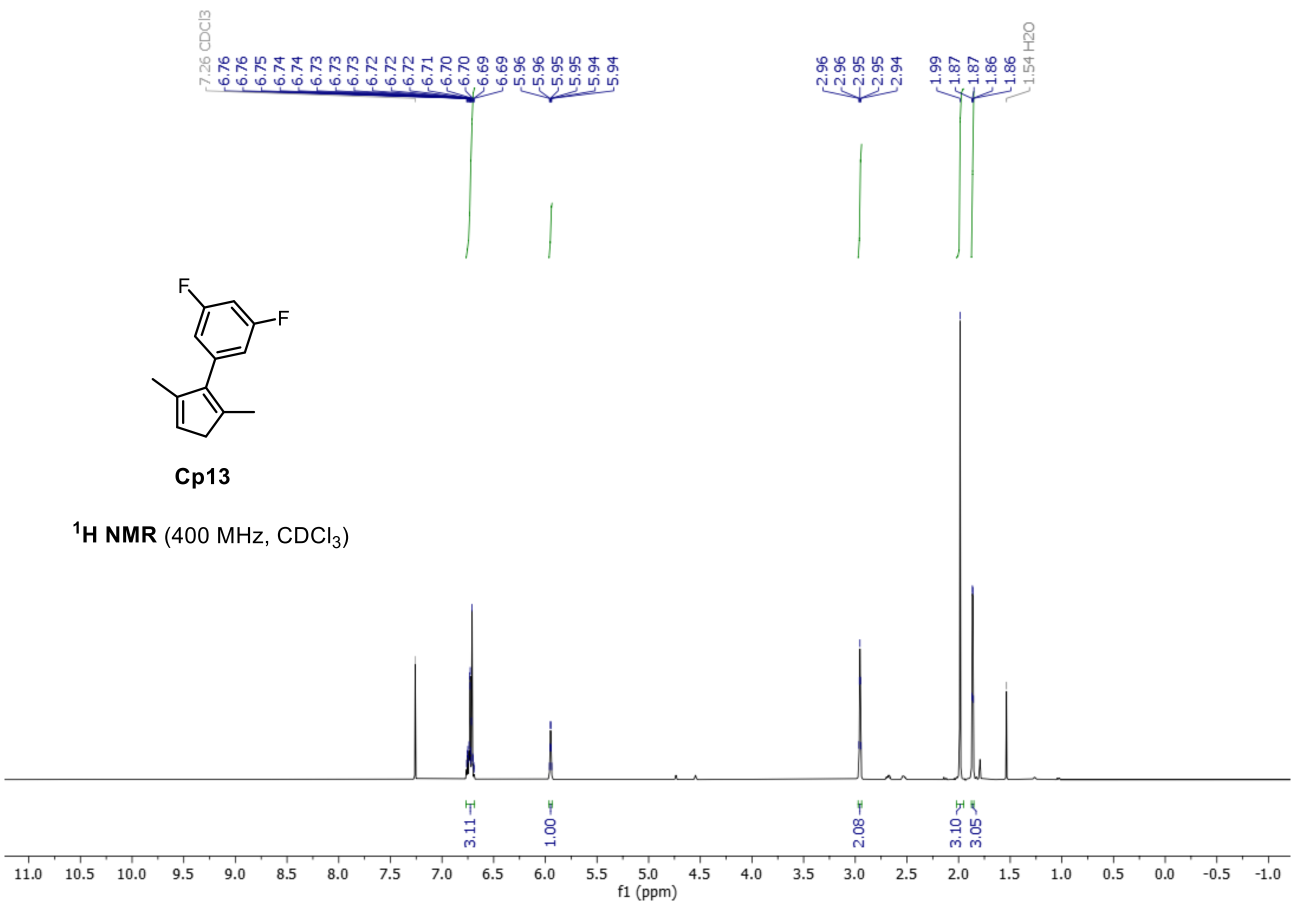

Cp13

^{1}H NMR (400 MHz, $CDCl_3$)

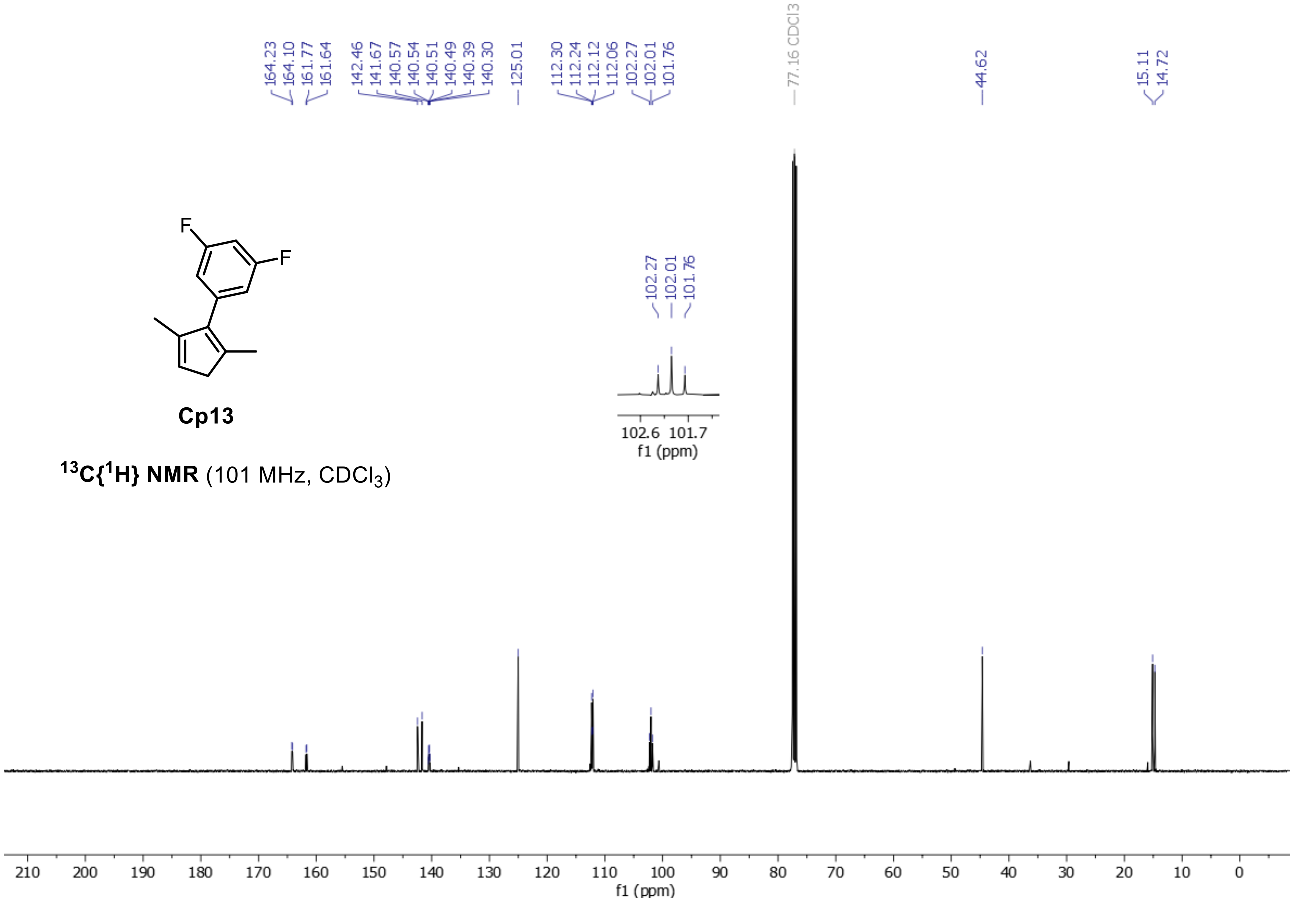

Cp13

^{13}C{^{1}H} NMR (101 MHz, $CDCl_3$)

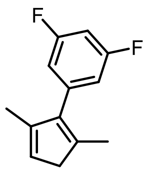

Cp13

^{19}F{^{1}H} NMR (376 MHz, $CDCl_3$)

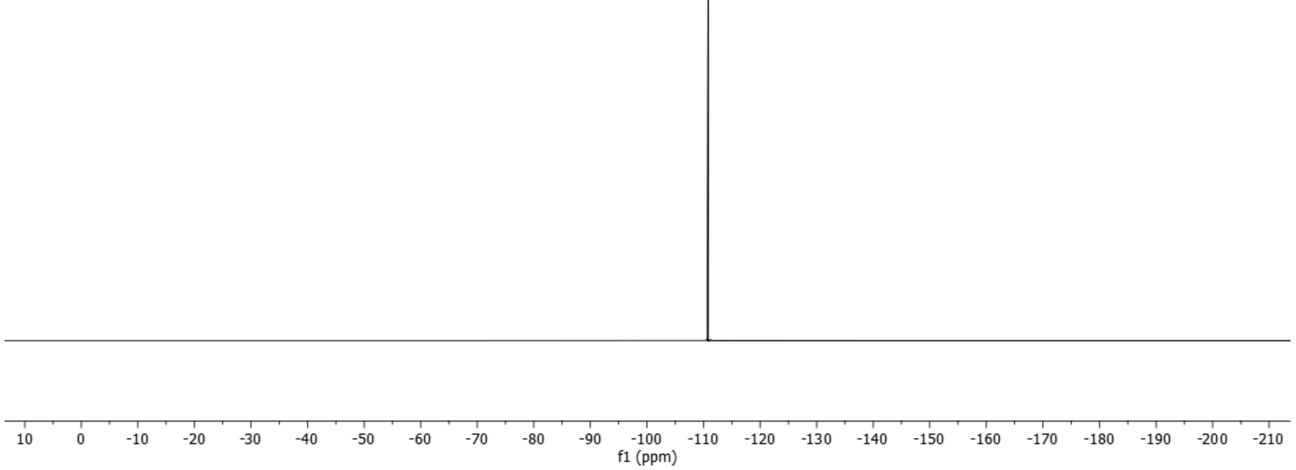

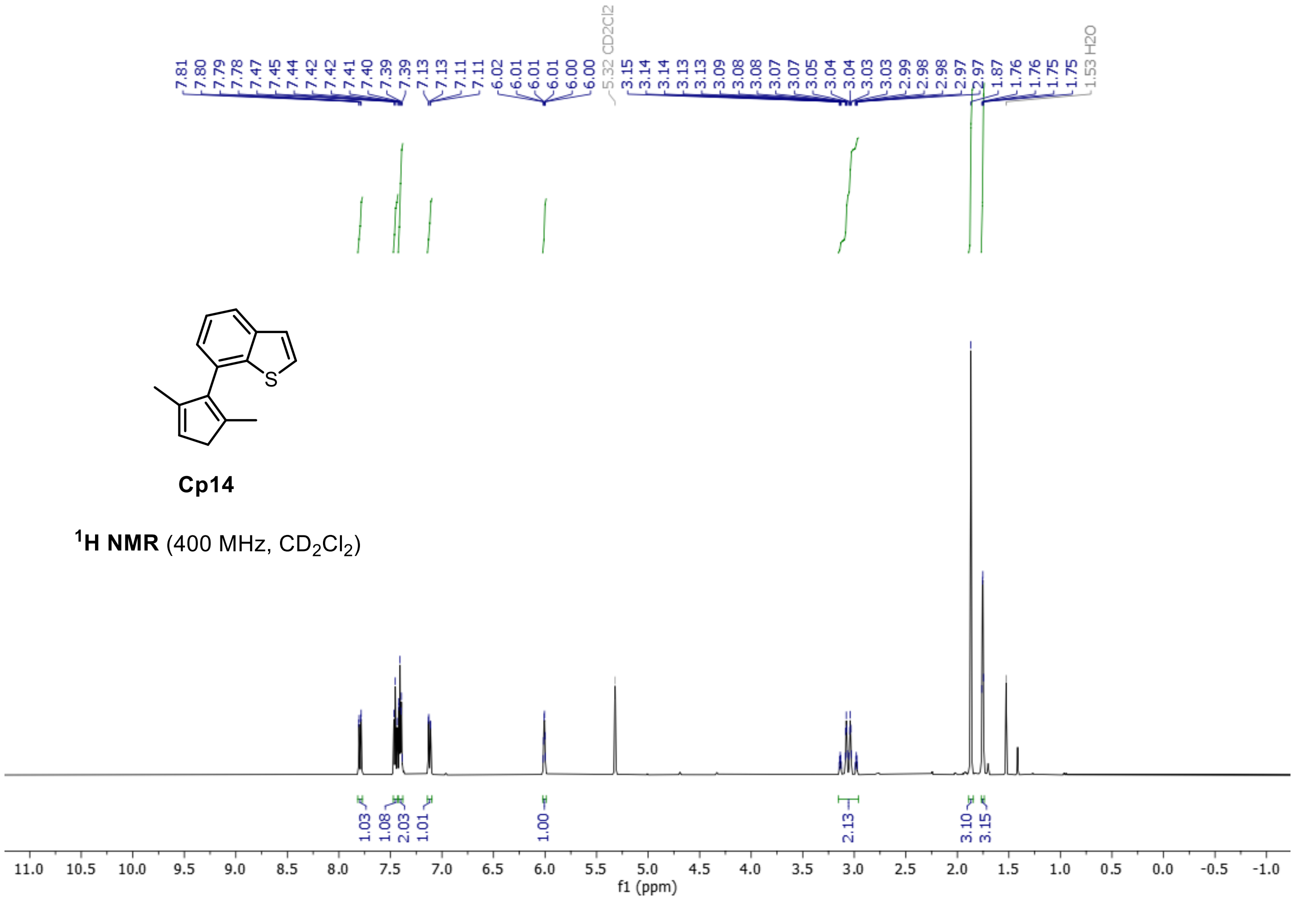
7.81 7.80 7.79 7.78 7.47 7.45 7.44 7.42 7.42 7.41 7.40 7.39 7.39 7.13 7.13 7.11 7.11 6.02 6.01 6.01 6.01 6.00 6.00 5.32 CD2Cl2 3.15 3.14 3.14 3.13 3.13 3.09 3.08 3.08 3.07 3.07 3.05 3.04 3.04 3.03 3.03 2.99 2.98 2.98 2.97 2.97 1.87 1.76 1.76 1.75 1.75 1.53 H2O
S
Cp14
1H NMR (400 MHz, CD2Cl2)
1.03 1.08 2.03 1.01 1.00 2.13 3.10 3.15
11.0 10.5 10.0 9.5 9.0 8.5 8.0 7.5 7.0 6.5 6.0 5.5 5.0 4.5 4.0 3.5 3.0 2.5 2.0 1.5 1.0 0.5 0.0 -0.5 -1.0
f1 (ppm)

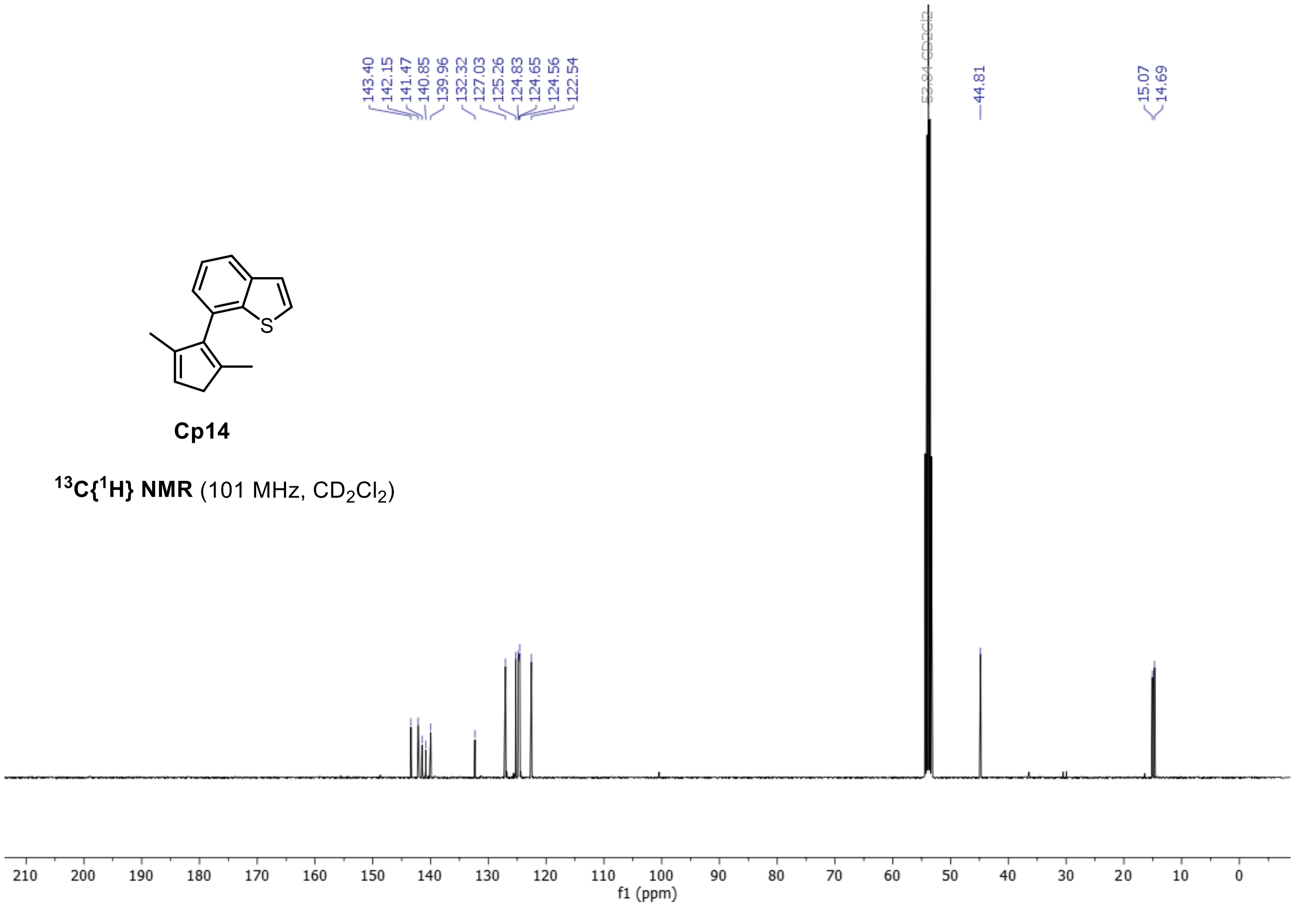
143.40 142.15 141.47 140.85 139.96 132.32 127.03 125.26 124.83 124.65 124.56 122.54
53.84 CD2Cl2
44.81
15.07 14.69
S
Cp14
13C{1H} NMR (101 MHz, CD2Cl2)
210 200 190 180 170 160 150 140 130 120 110 100 90 80 70 60 50 40 30 20 10 0
f1 (ppm)

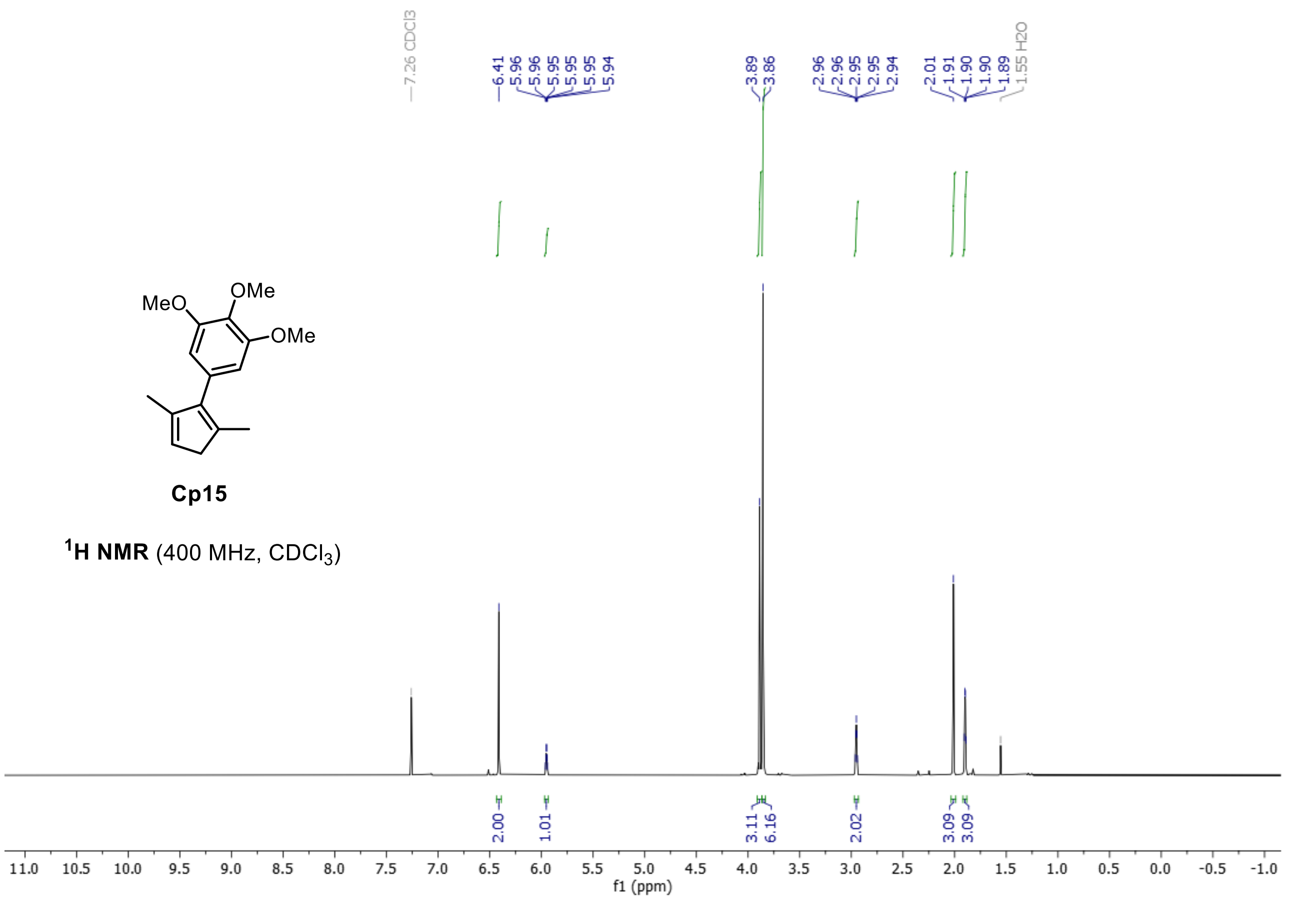

7.26 CDCl3
6.41
5.96
5.96
5.95
5.95
5.95
5.94
3.89
3.86
2.96
2.96
2.95
2.95
2.94
2.01
1.91
1.90
1.90
1.89
1.55 H2O
MeO
OMe
OMe
Cp15
^{1}H NMR (400 MHz, $CDCl_3$)
2.00
1.01
3.11
6.16
2.02
3.09
3.09
f1 (ppm)

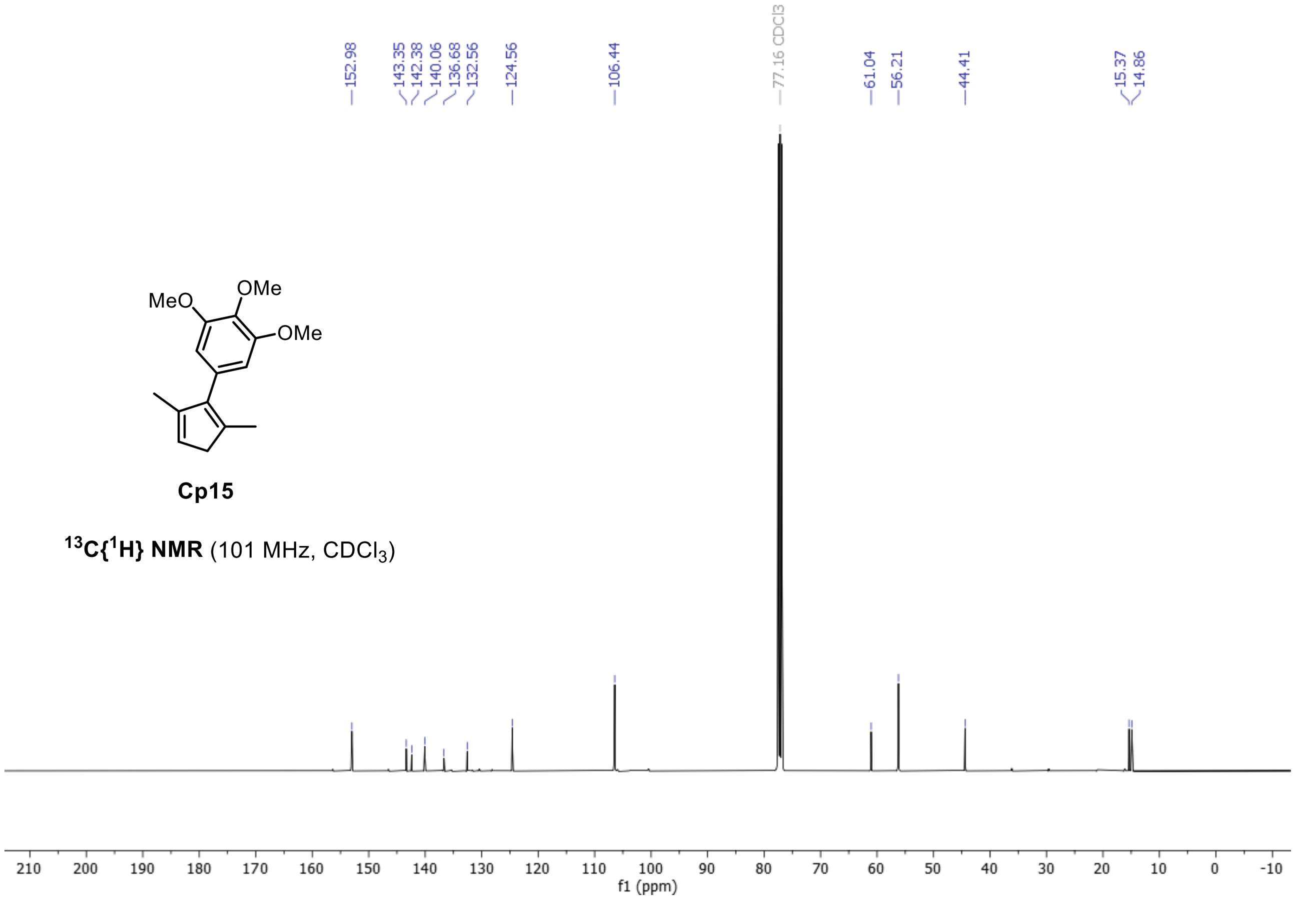

152.98
143.35
142.38
140.06
136.68
132.56
124.56
106.44
77.16 CDCl3
61.04
56.21
44.41
15.37
14.86
MeO
OMe
OMe
Cp15
^{13}C{^{1}H} NMR (101 MHz, $CDCl_3$)
f1 (ppm)

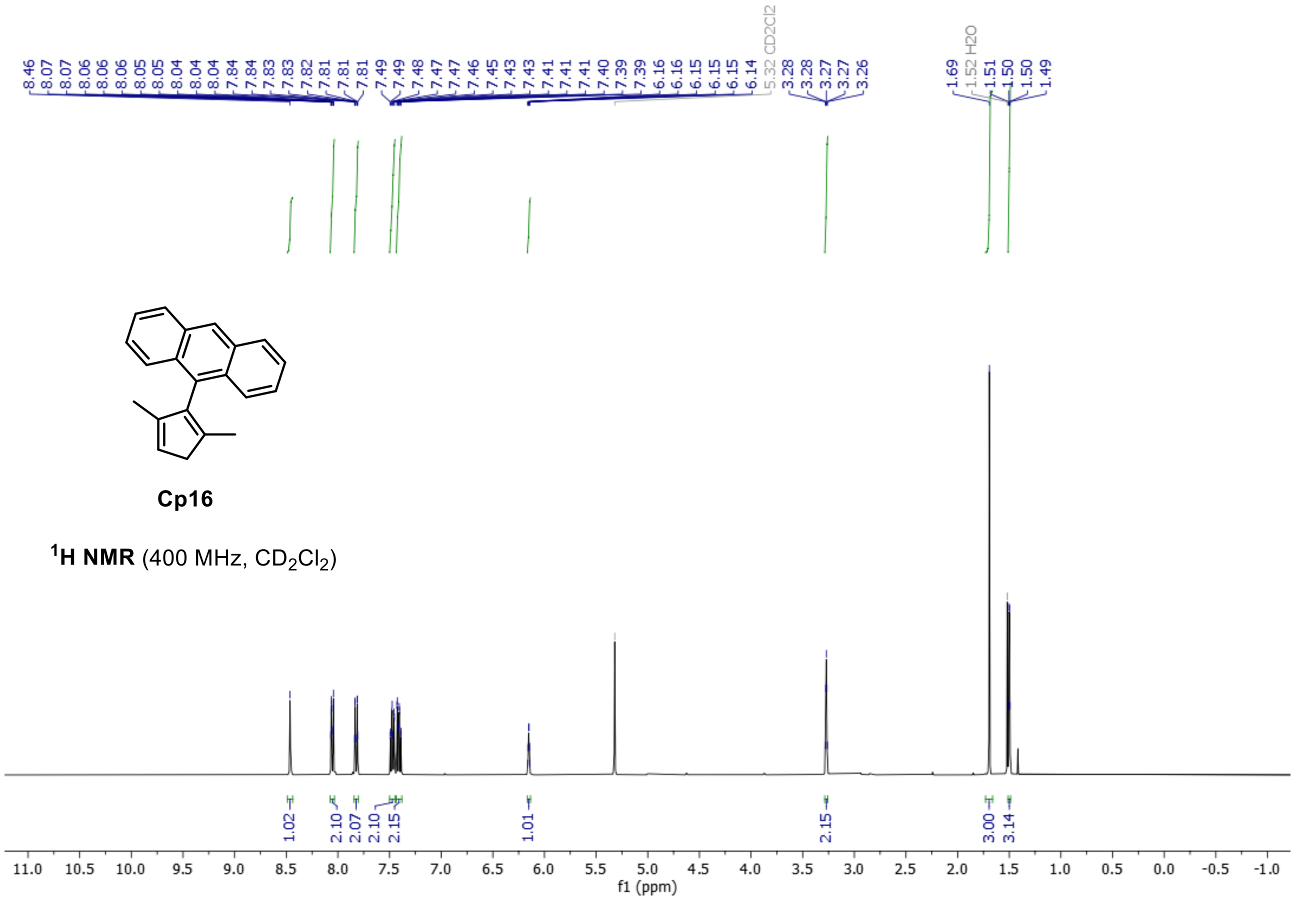

Cp16

^{1}H NMR (400 MHz, CD_2Cl_2)

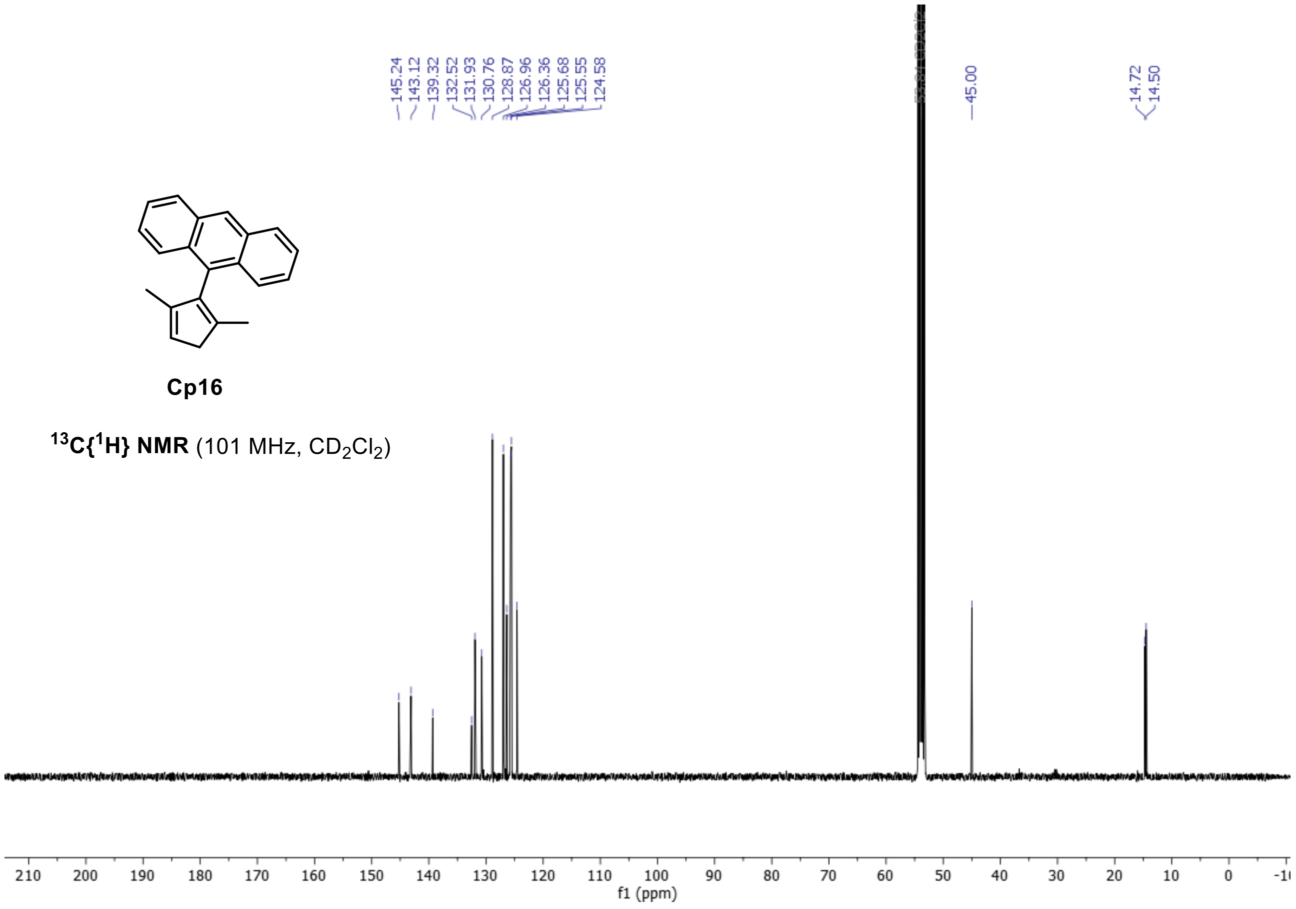

Cp16

^{13}C{^{1}H} NMR (101 MHz, CD_2Cl_2)

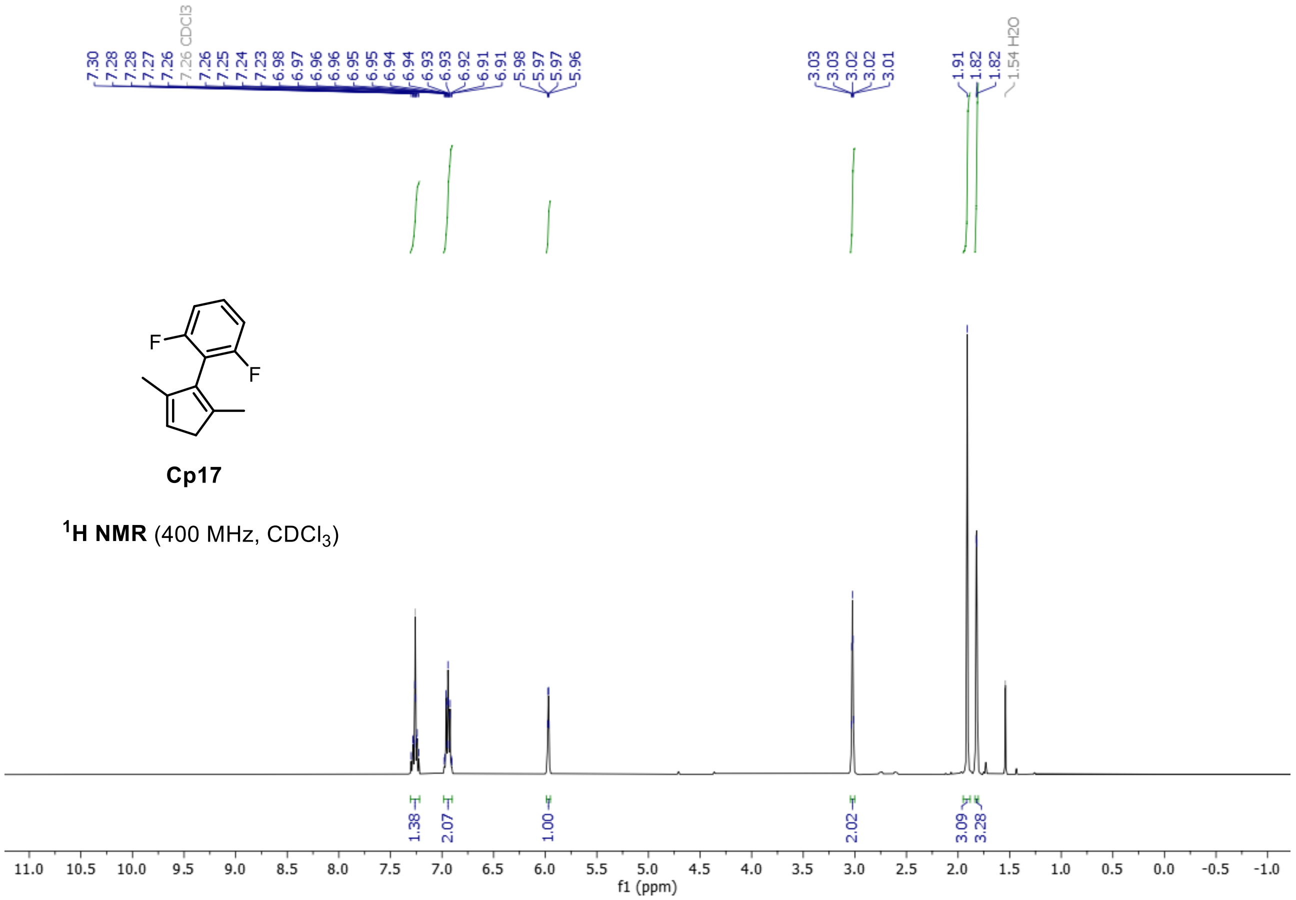

Cp17

¹H NMR (400 MHz, $CDCl_3$)

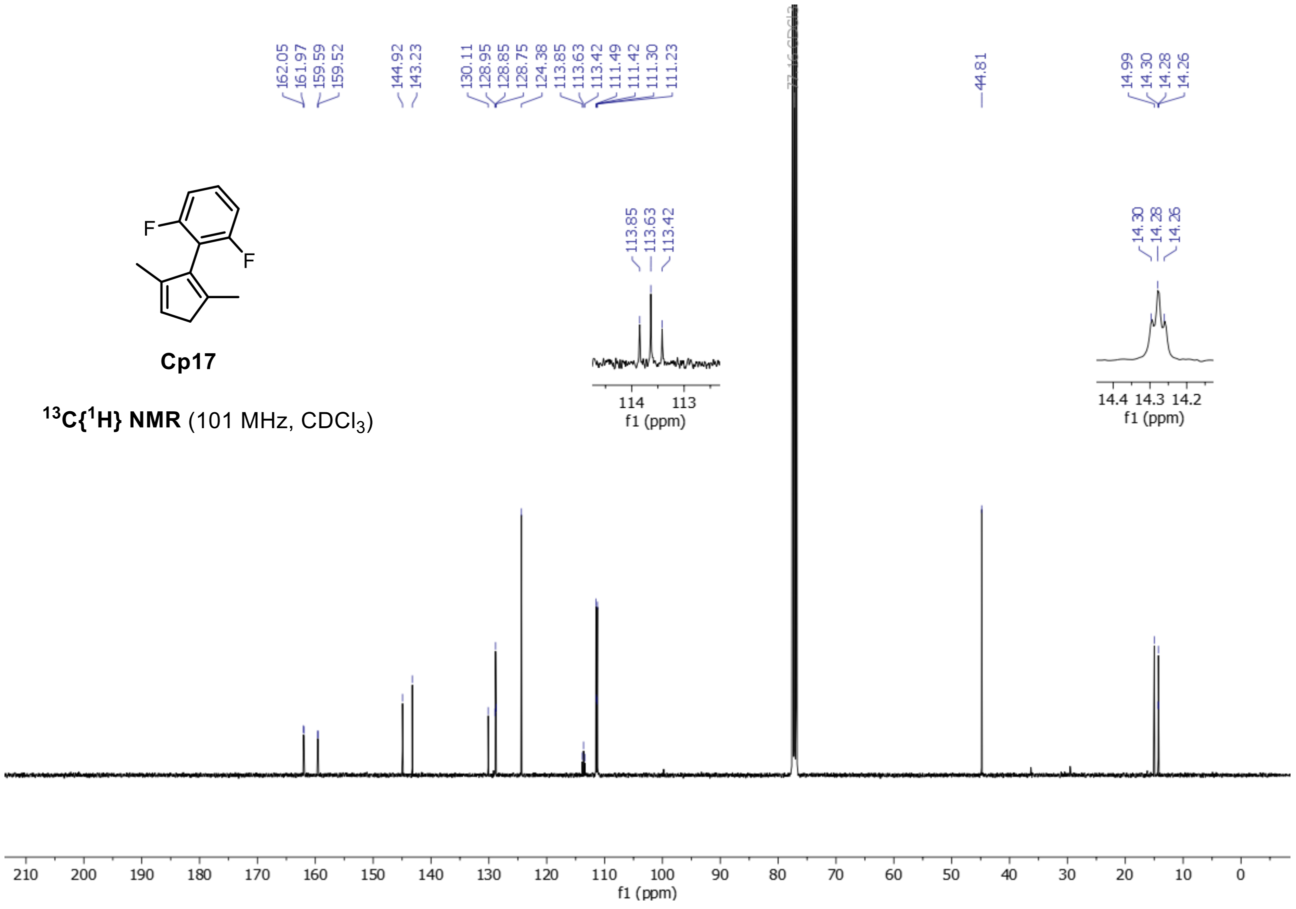

Cp17

$^{13}C\{^1H\}$ NMR (101 MHz, $CDCl_3$)

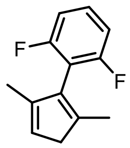

Cp17

$^{19}F\{^{1}H\}$ NMR (376 MHz, $CDCl_3$)

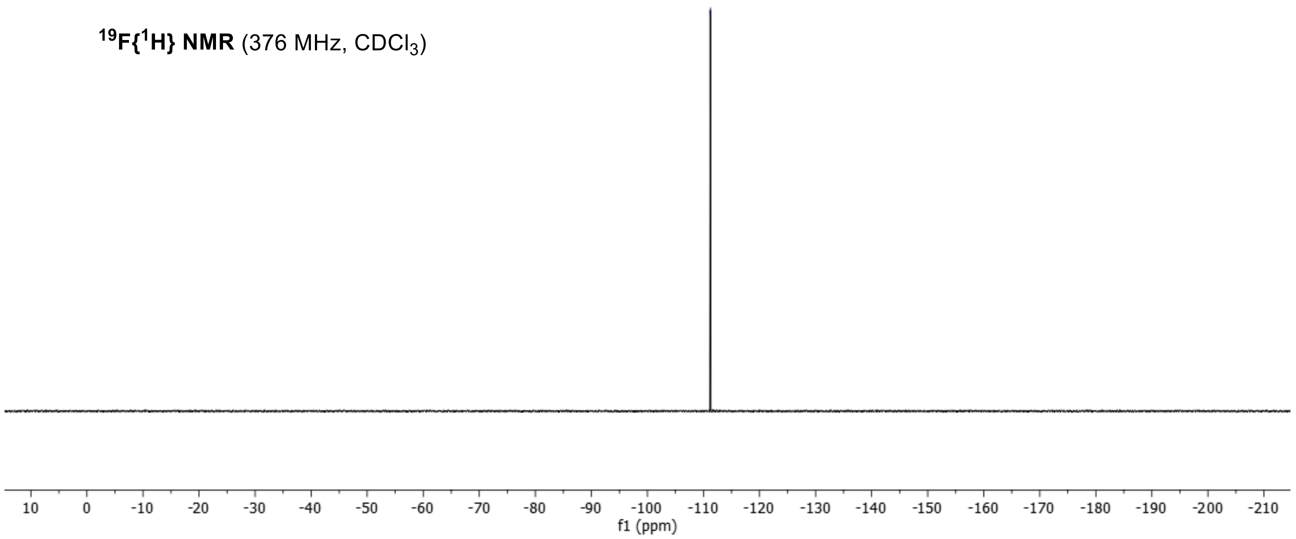

Cp18

^{1}H NMR (400 MHz, CD_2Cl_2)

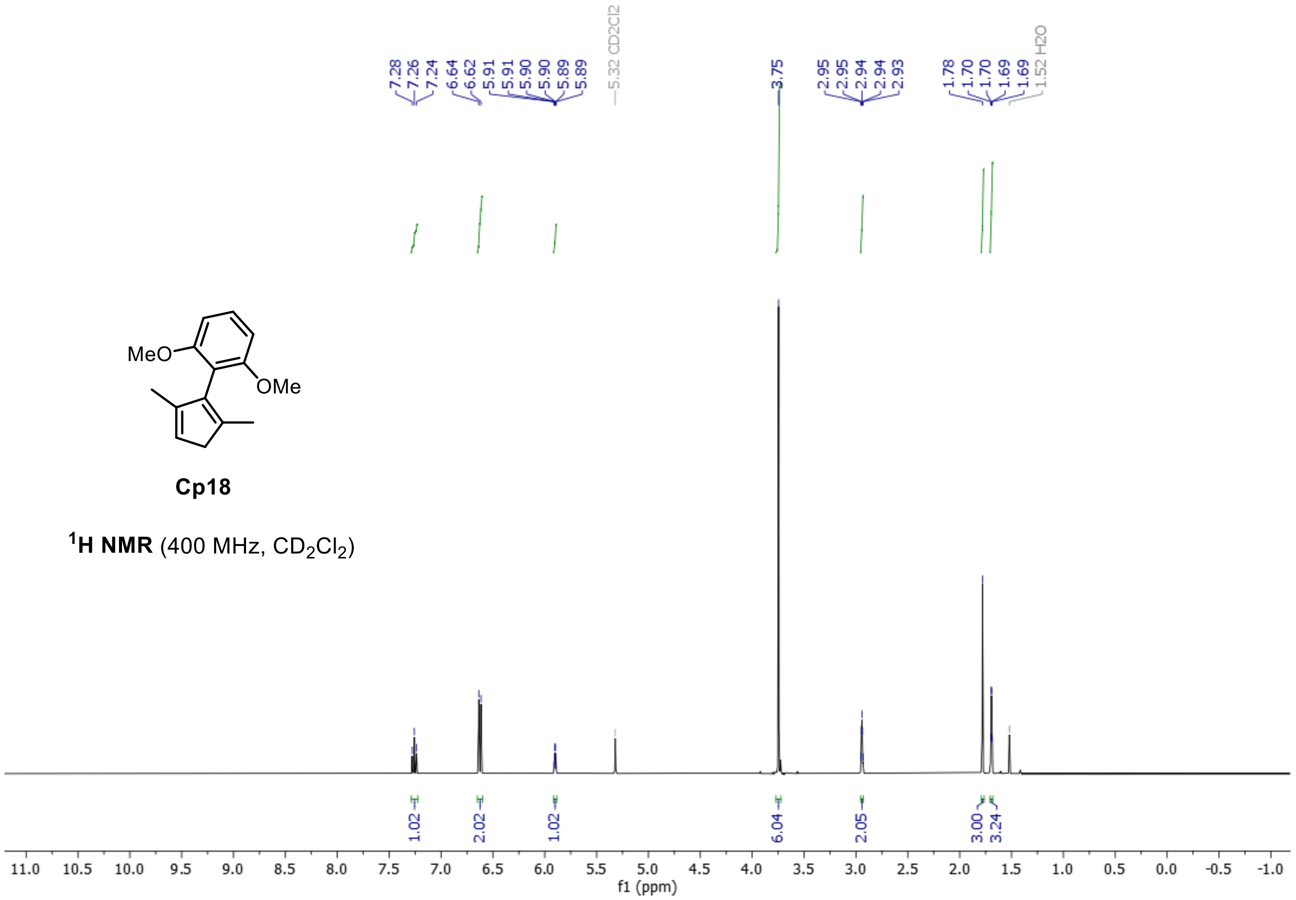

Cp18

^{13}C{^{1}H} NMR (101 MHz, CD_2Cl_2)

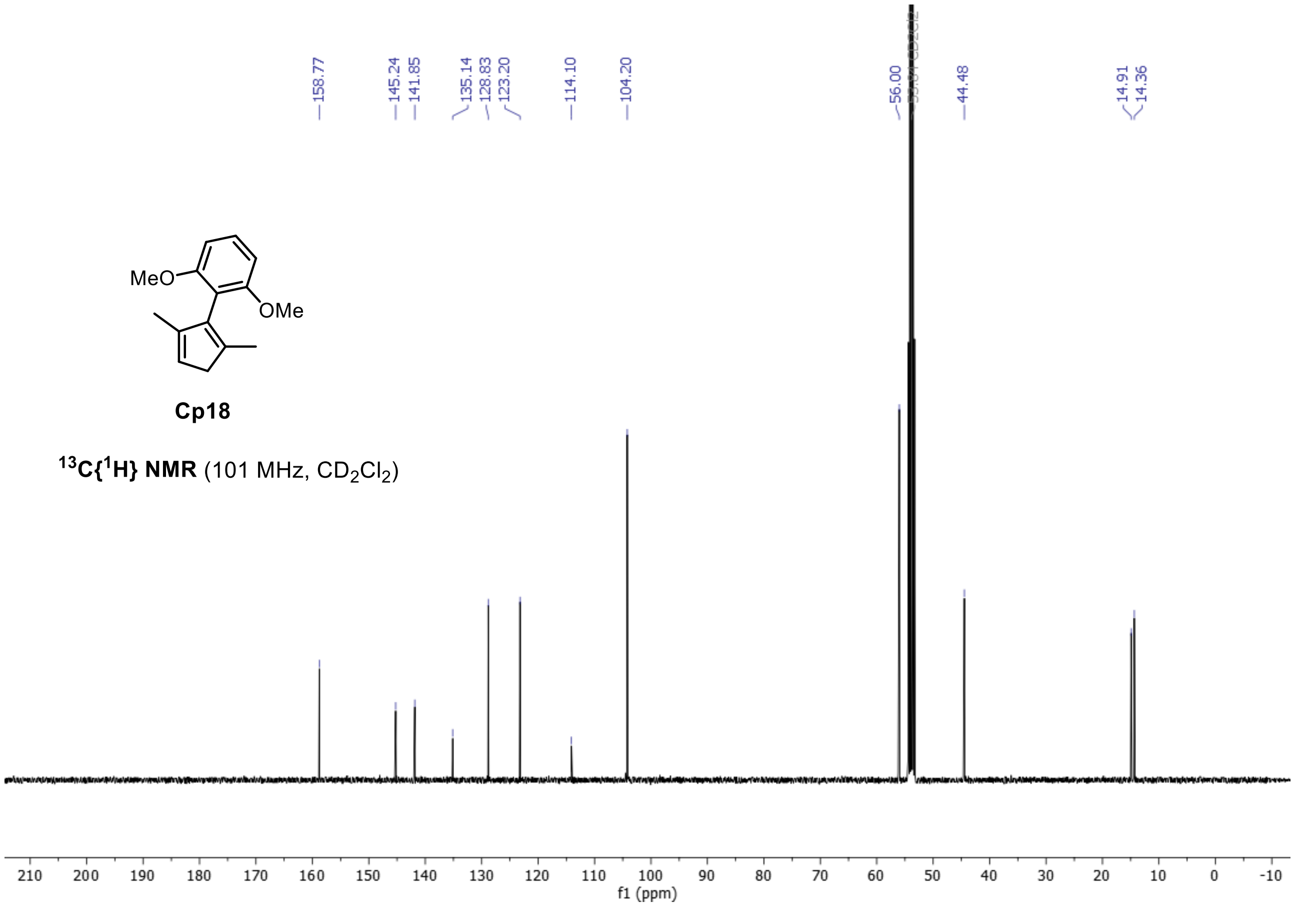

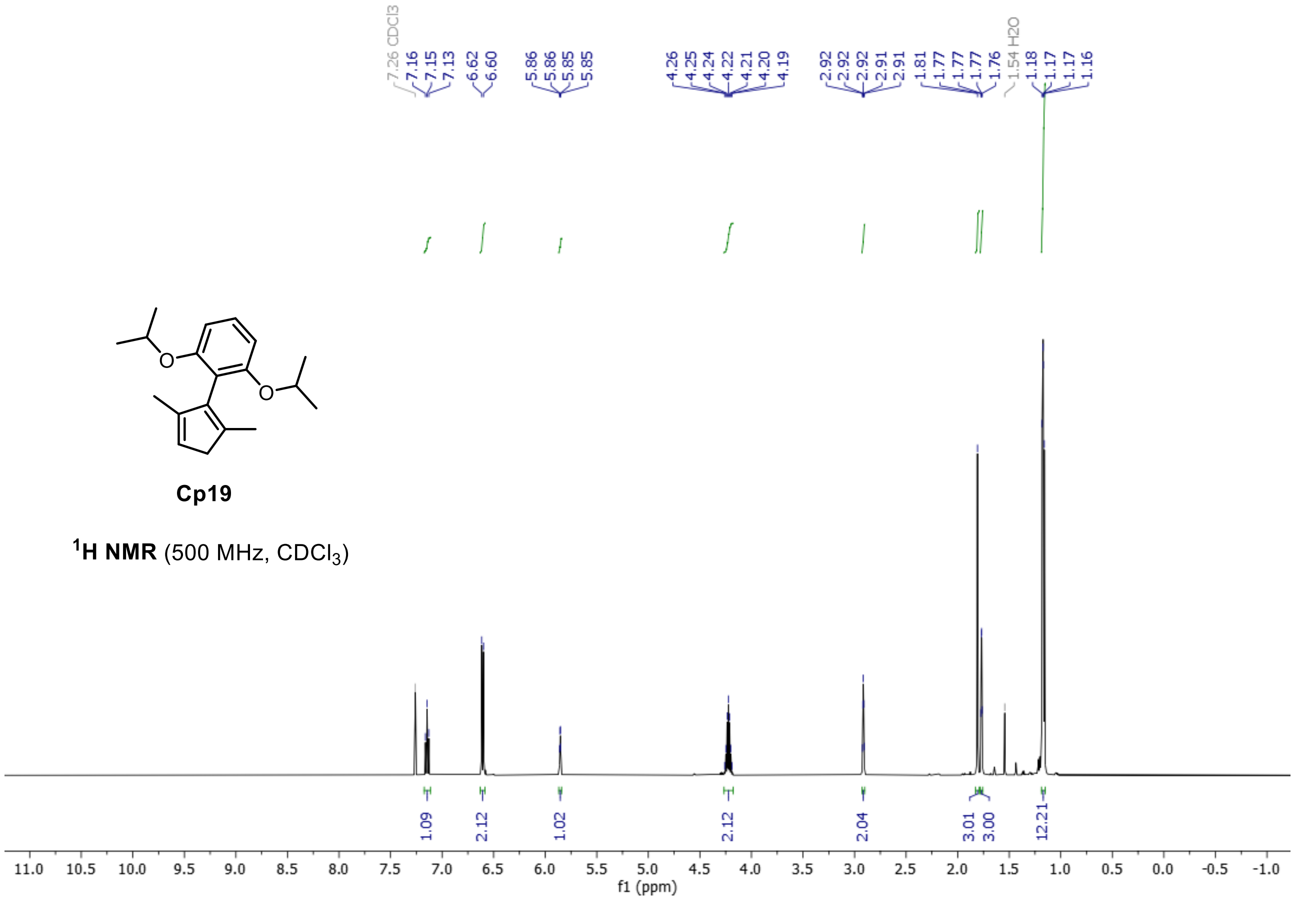

Cp19

^{1}H NMR (500 MHz, $CDCl_3$)

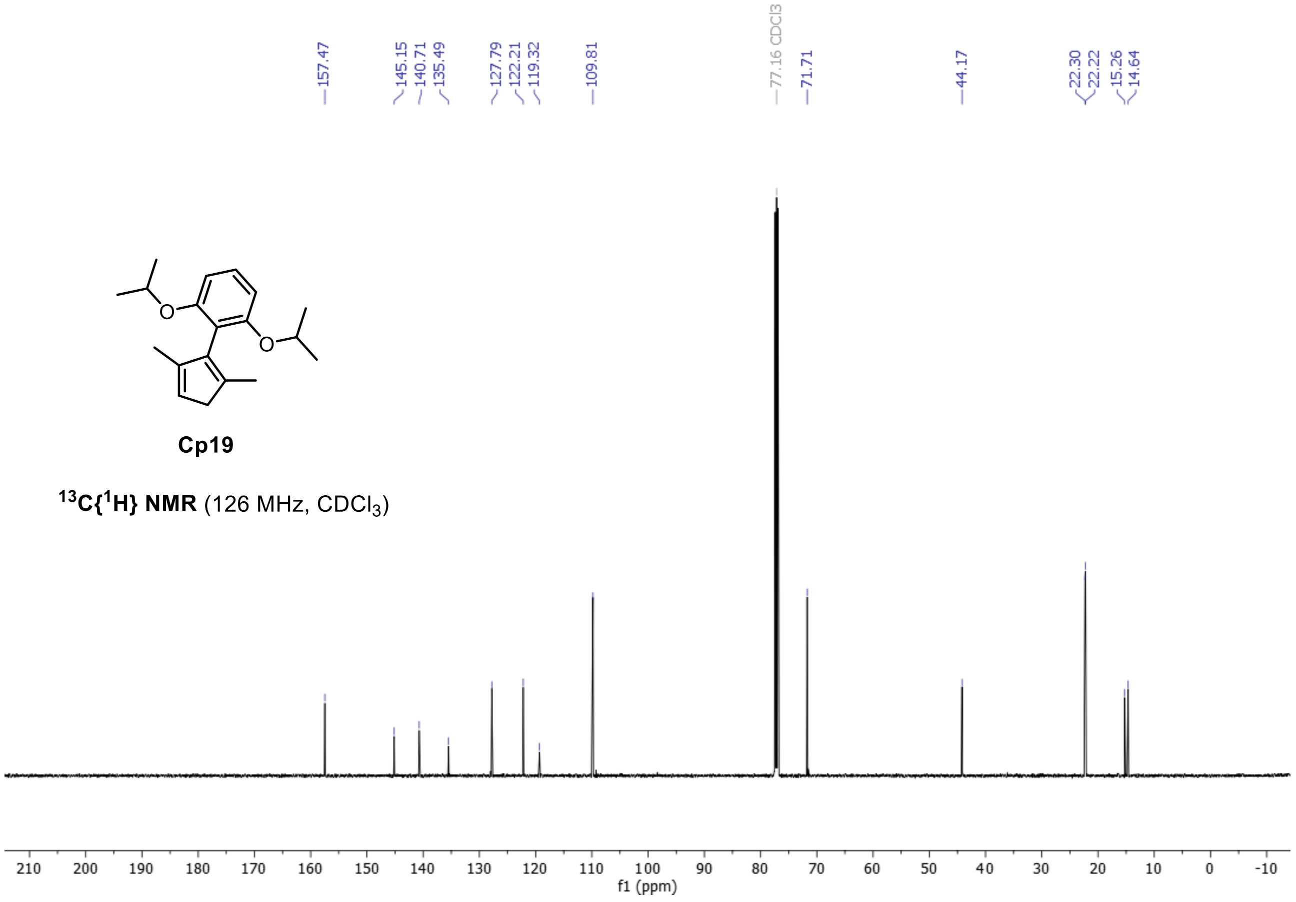

Cp19

^{13}C{^{1}H} NMR (126 MHz, $CDCl_3$)

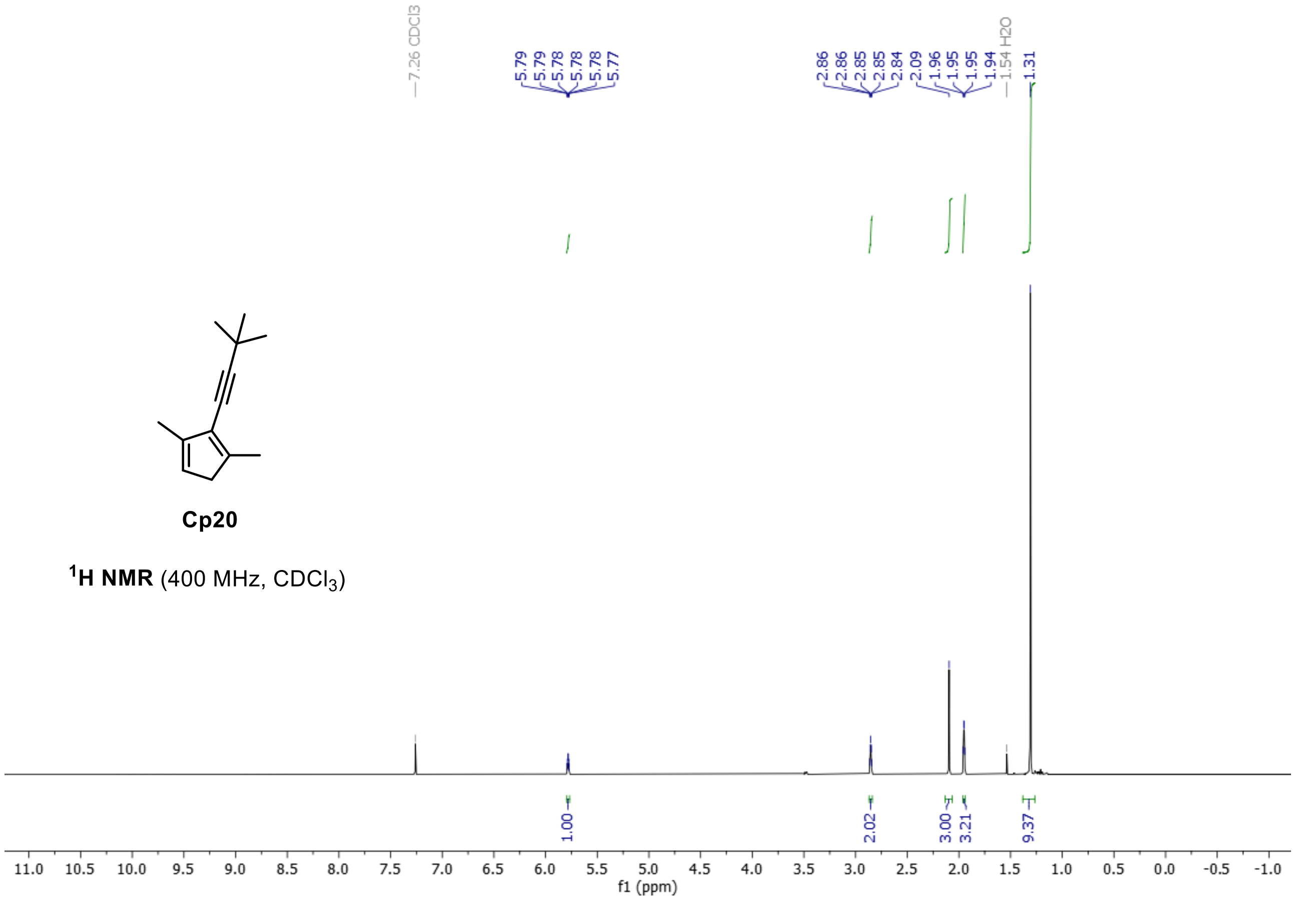

Cp20

^{1}H NMR (400 MHz, $CDCl_3$)

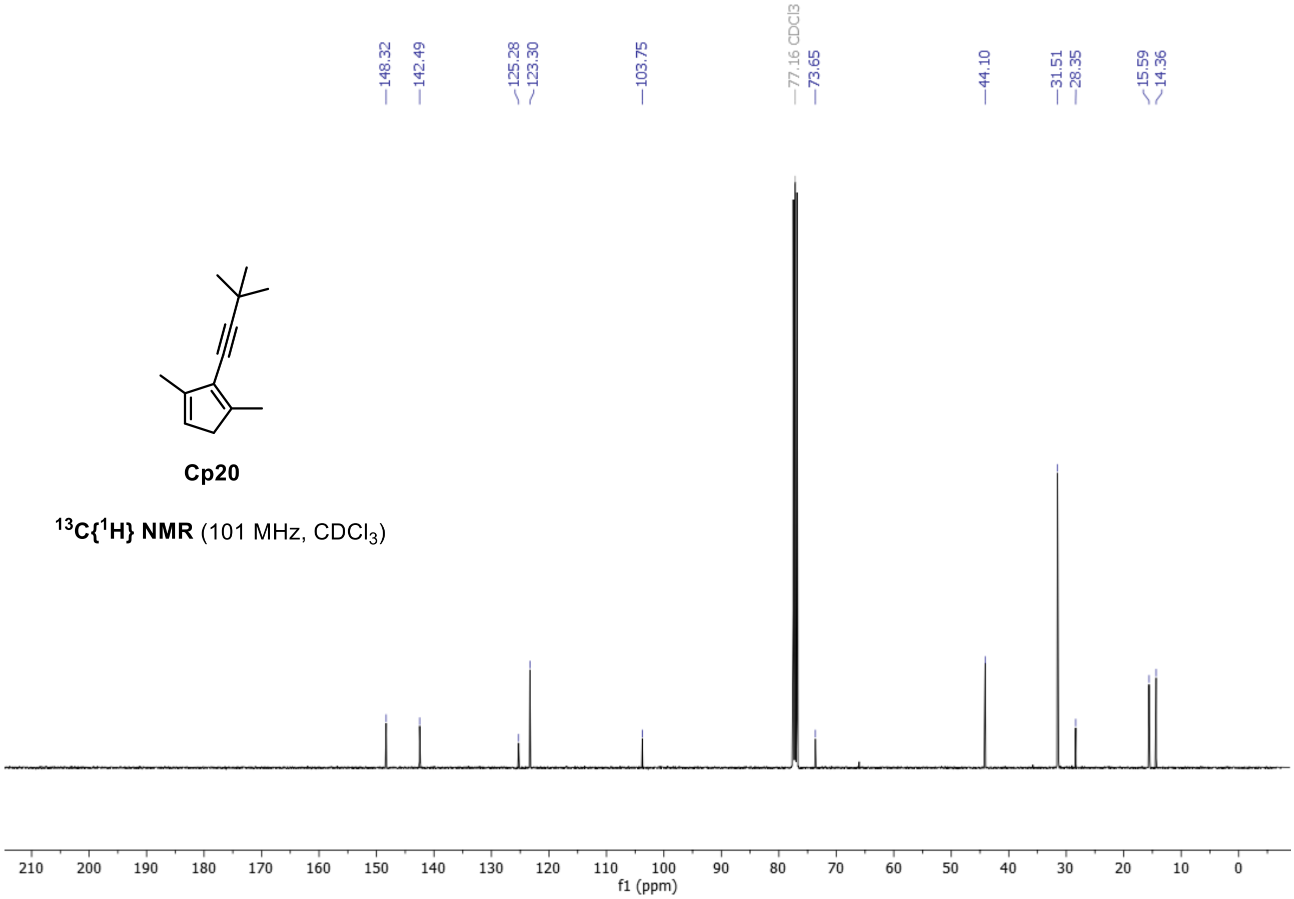

Cp20

^{13}C{^{1}H} NMR (101 MHz, $CDCl_3$)

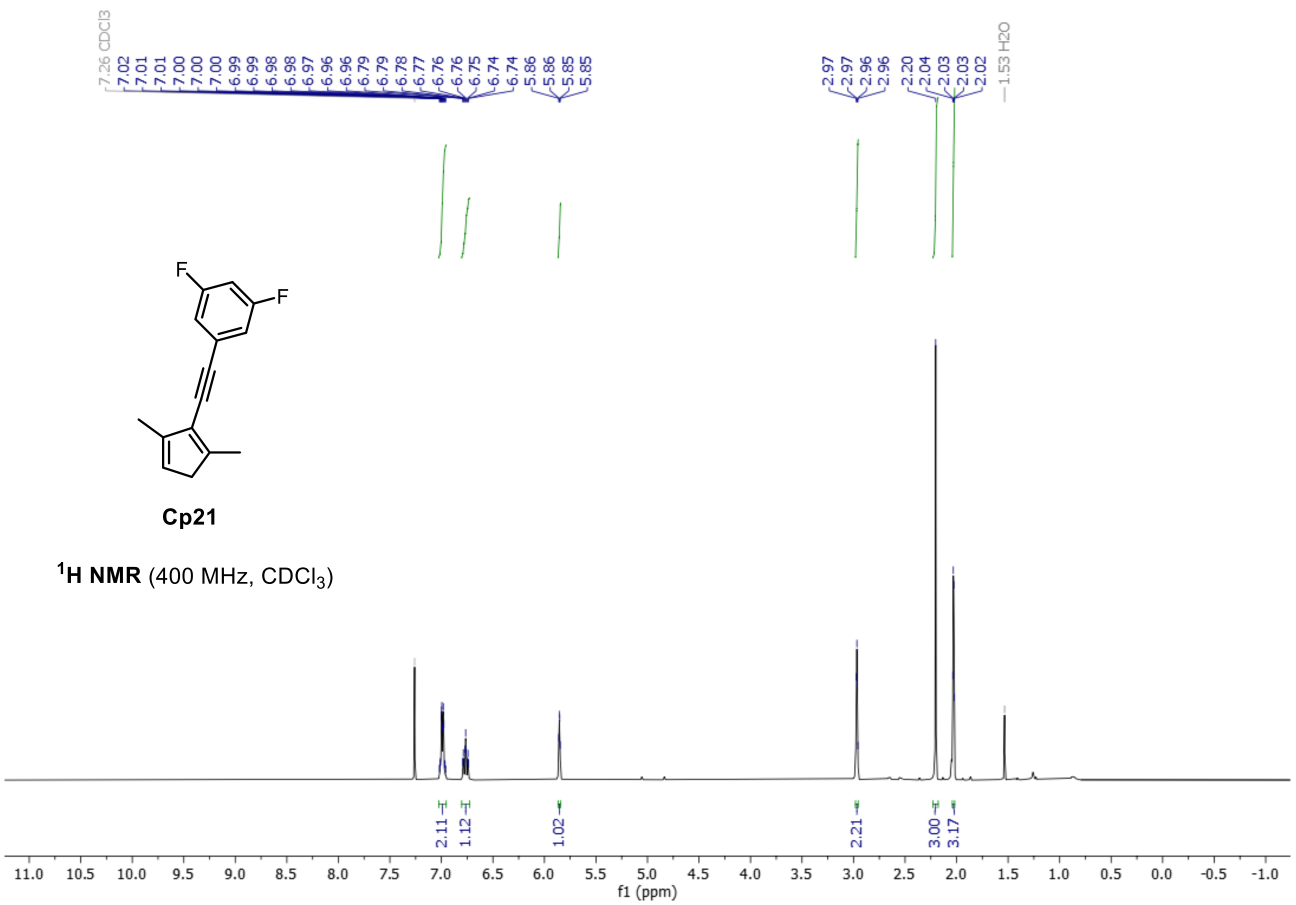

Cp21

^{1}H NMR (400 MHz, $CDCl_3$)

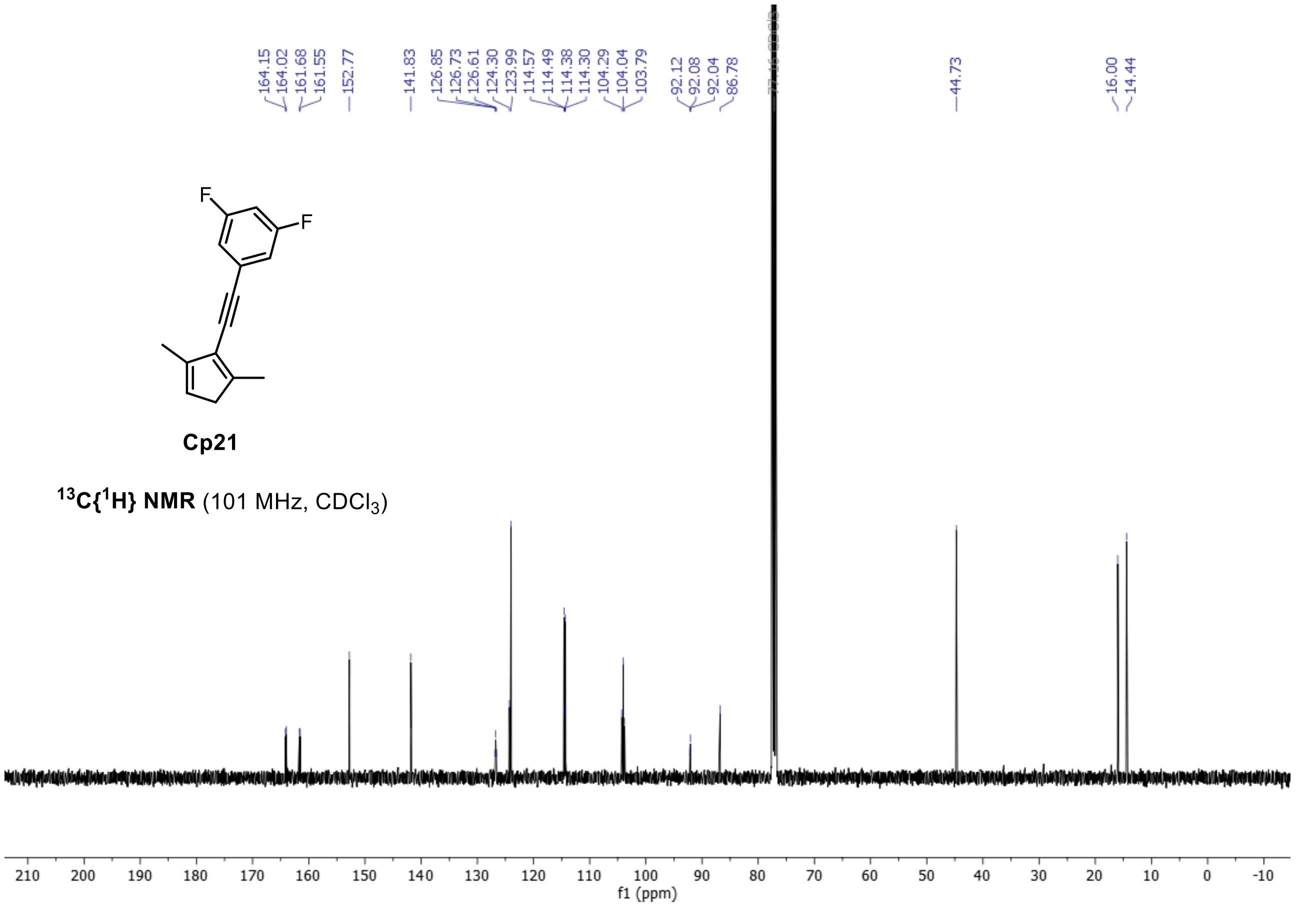

Cp21

^{13}C{^{1}H} NMR (101 MHz, $CDCl_3$)

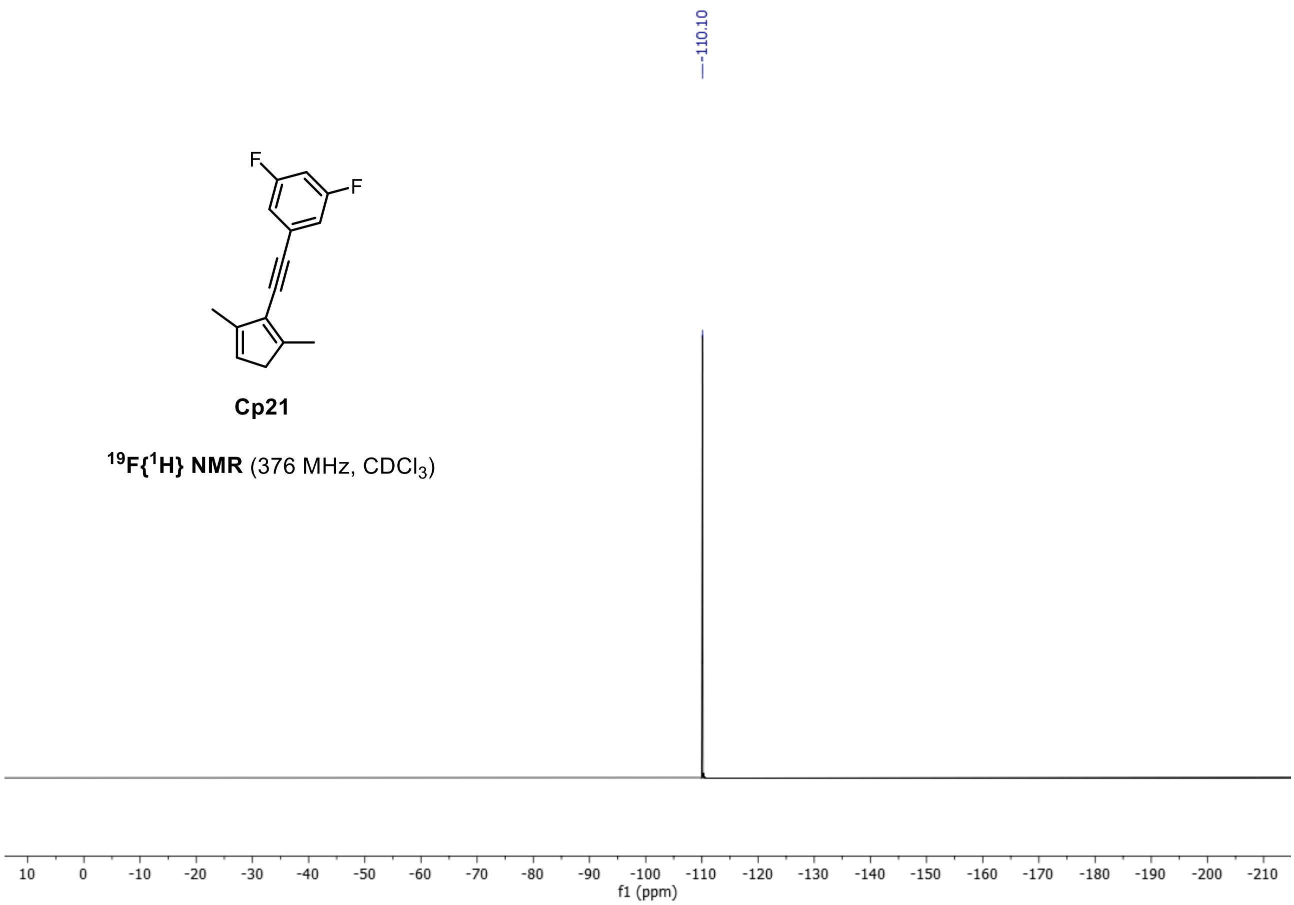

Cp21

$^{19}F\{^{1}H\}$ NMR (376 MHz, $CDCl_3$)

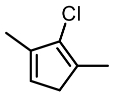

Cp22

^{1}H NMR (400 MHz, $CDCl_3$)

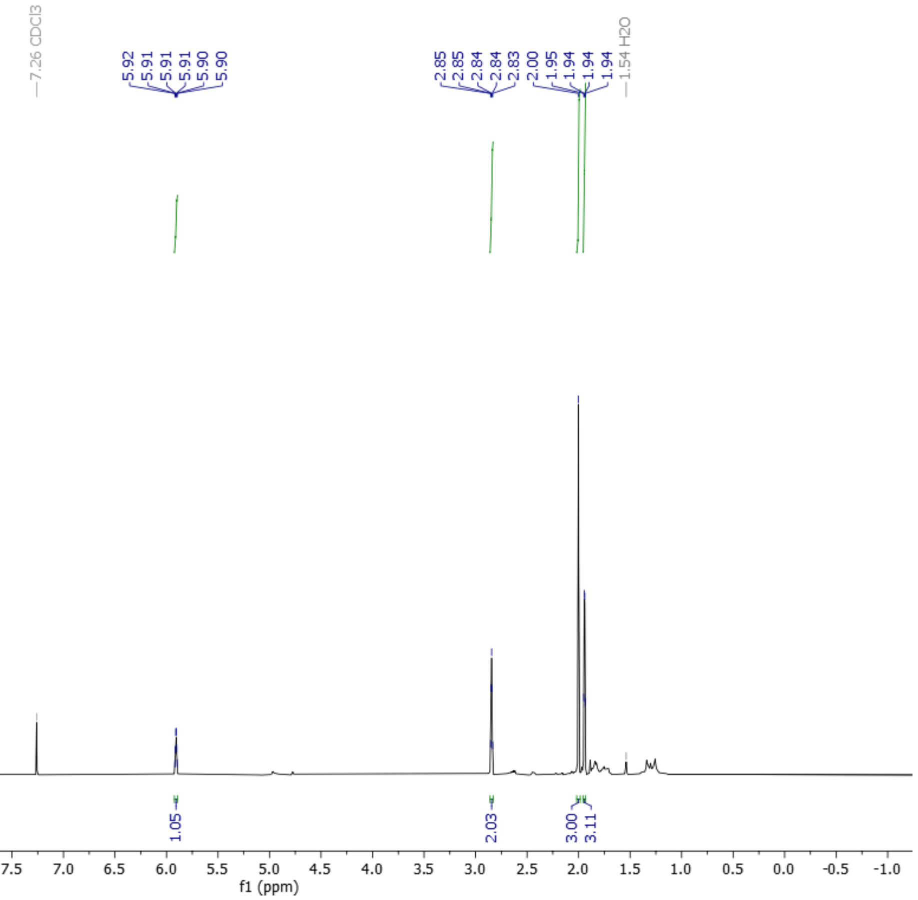

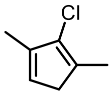

Cp22

^{13}C{^{1}H} NMR (101 MHz, $CDCl_3$)

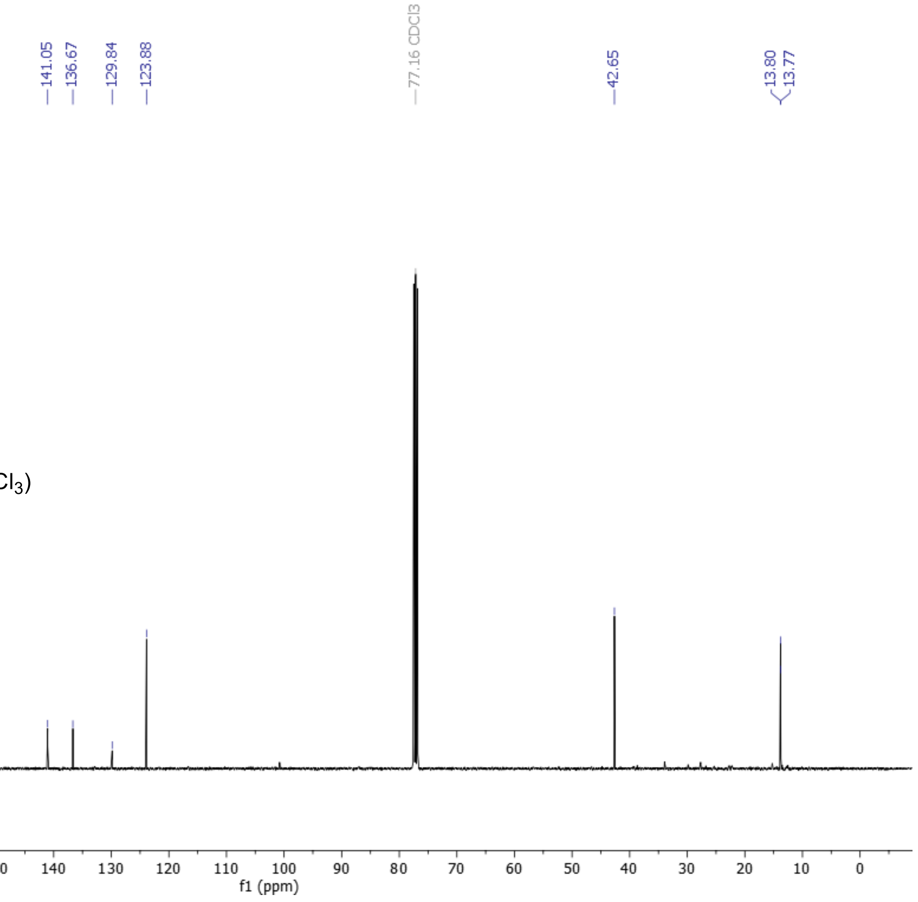

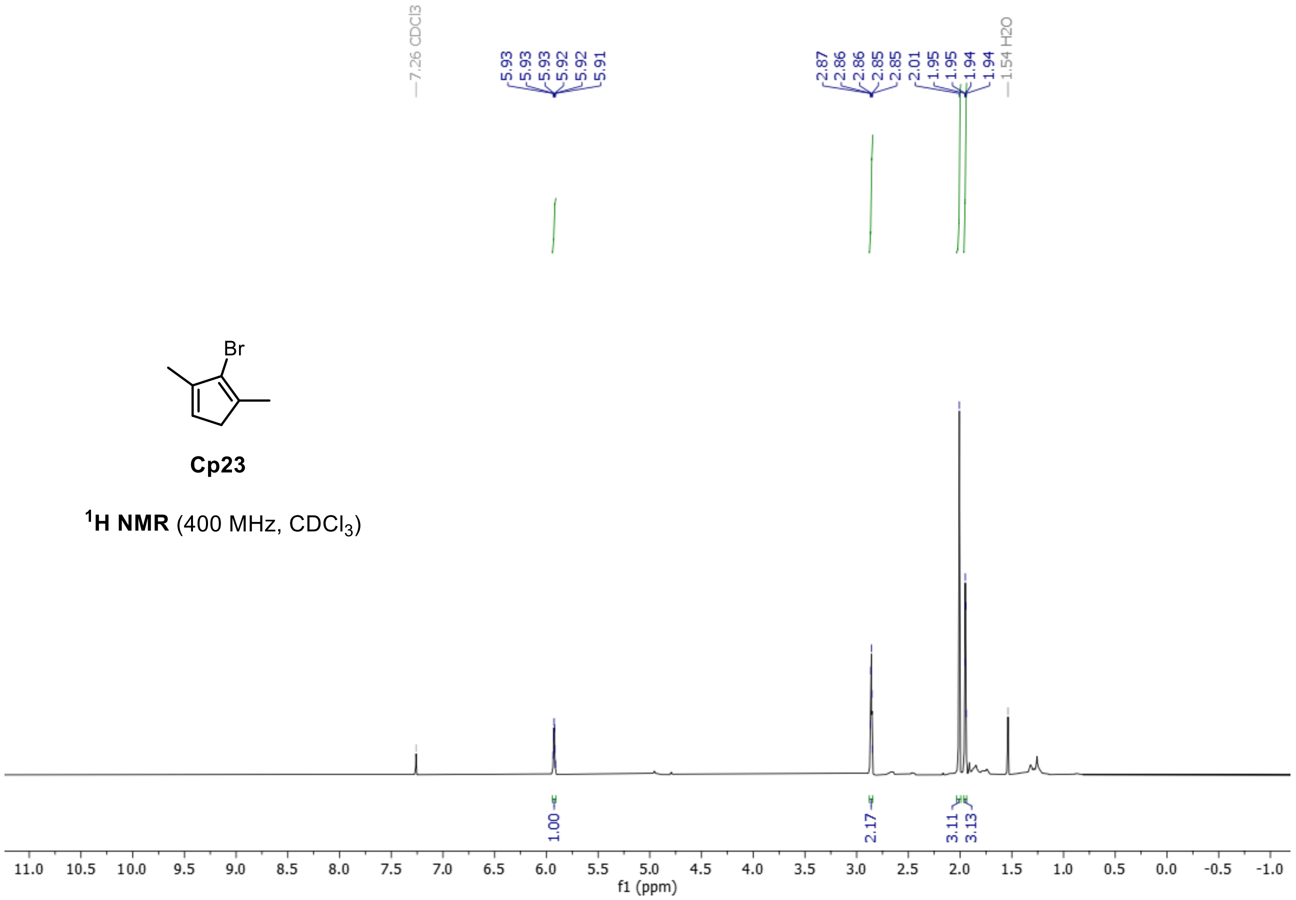

Cp23

^{1}H NMR (400 MHz, $CDCl_3$)

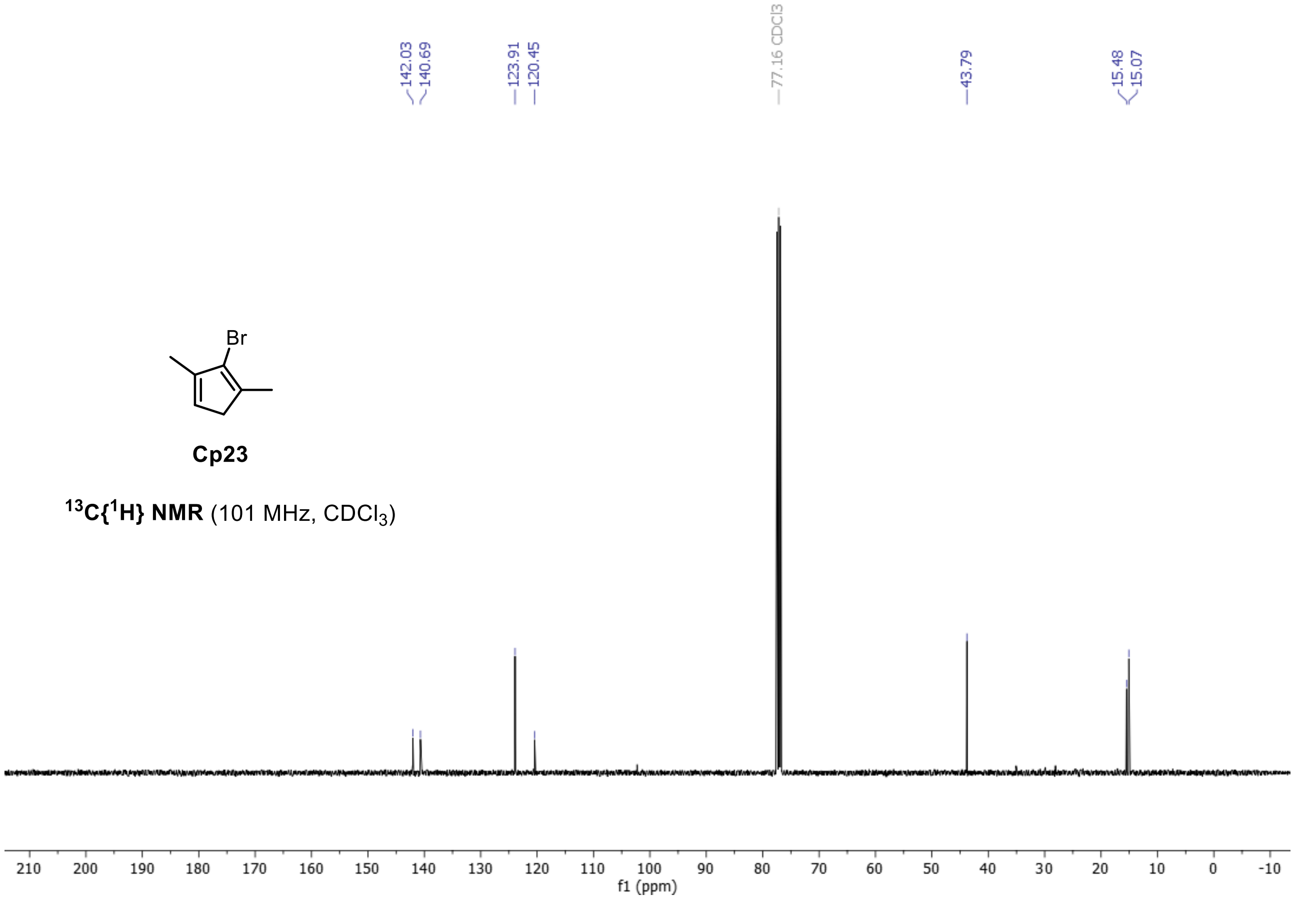

Cp23

$^{13}C\{^{1}H\}$ NMR (101 MHz, $CDCl_3$)

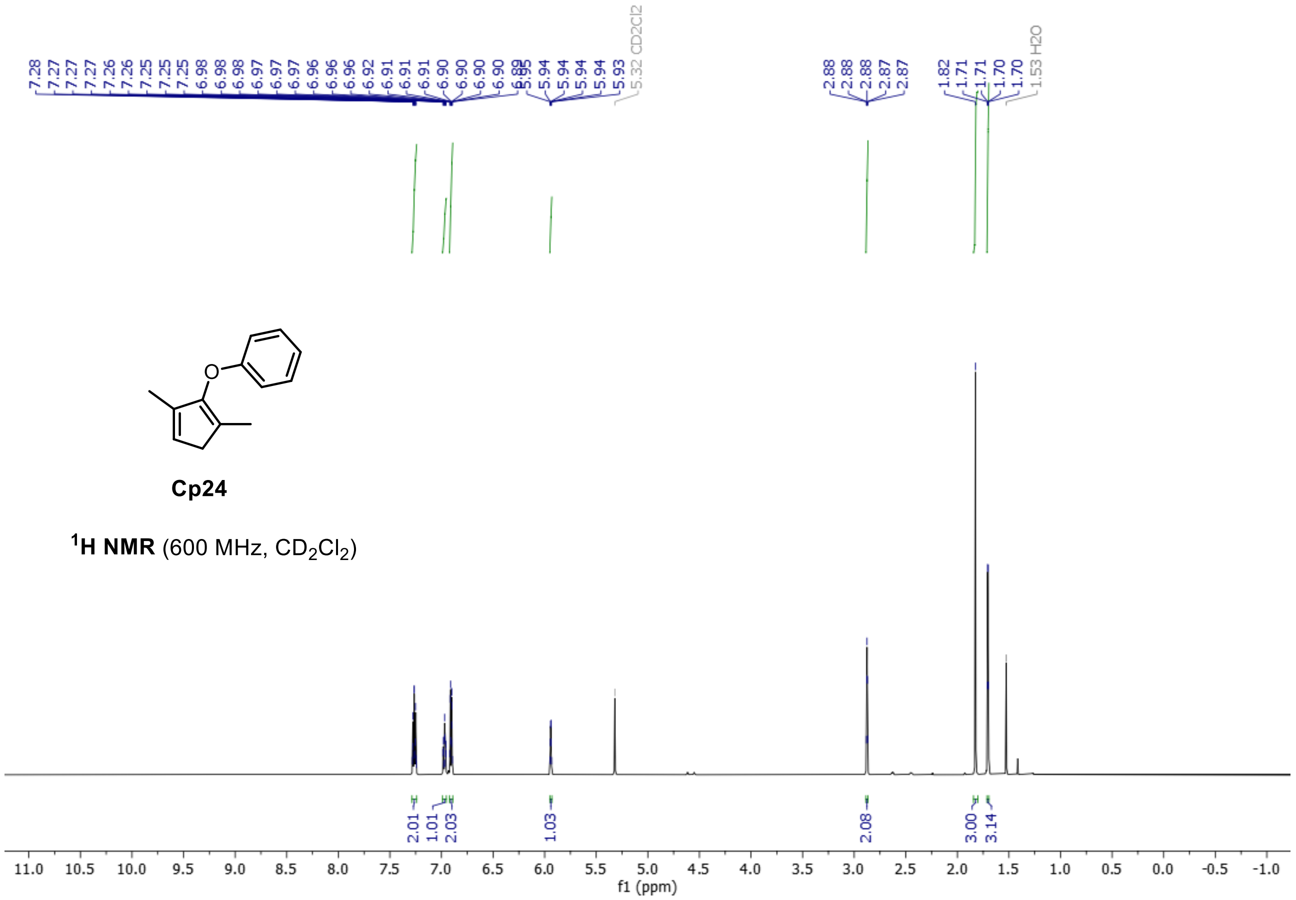
7.28 7.27 7.27 7.27 7.26 7.26 7.25 7.25 7.25 6.98 6.98 6.98 6.97 6.97 6.97 6.96 6.96 6.96 6.92 6.91 6.91 6.91 6.90 6.90 6.90 6.90 6.89 5.95 5.94 5.94 5.94 5.94 5.93
5.32 CD2Cl2
2.88 2.88 2.88 2.87 2.87
1.82 1.71 1.71 1.70 1.70
1.53 H2O
O
Cp24
^{1}H NMR (600 MHz, CD_2Cl_2)
2.01 1.01 2.03 1.03 2.08 3.00 3.14
11.0 10.5 10.0 9.5 9.0 8.5 8.0 7.5 7.0 6.5 6.0 5.5 5.0 4.5 4.0 3.5 3.0 2.5 2.0 1.5 1.0 0.5 0.0 -0.5 -1.0
f1 (ppm)

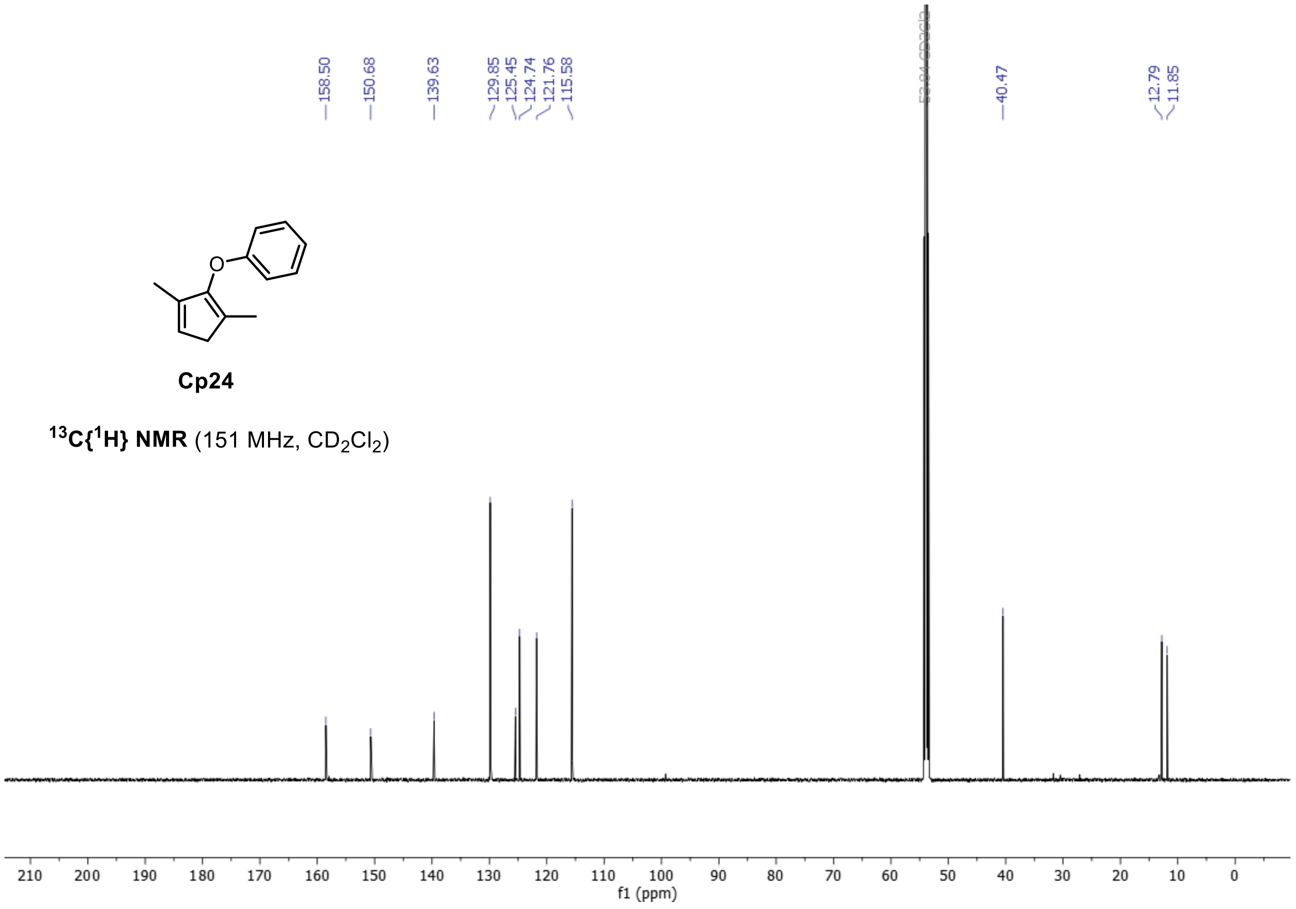
158.50
150.68
139.63
129.85 125.45 124.74 121.76 115.58
53.84 CD2Cl2
40.47
12.79 11.85
O
Cp24
^{13}C{^{1}H} NMR (151 MHz, CD_2Cl_2)
210 200 190 180 170 160 150 140 130 120 110 100 90 80 70 60 50 40 30 20 10 0
f1 (ppm)

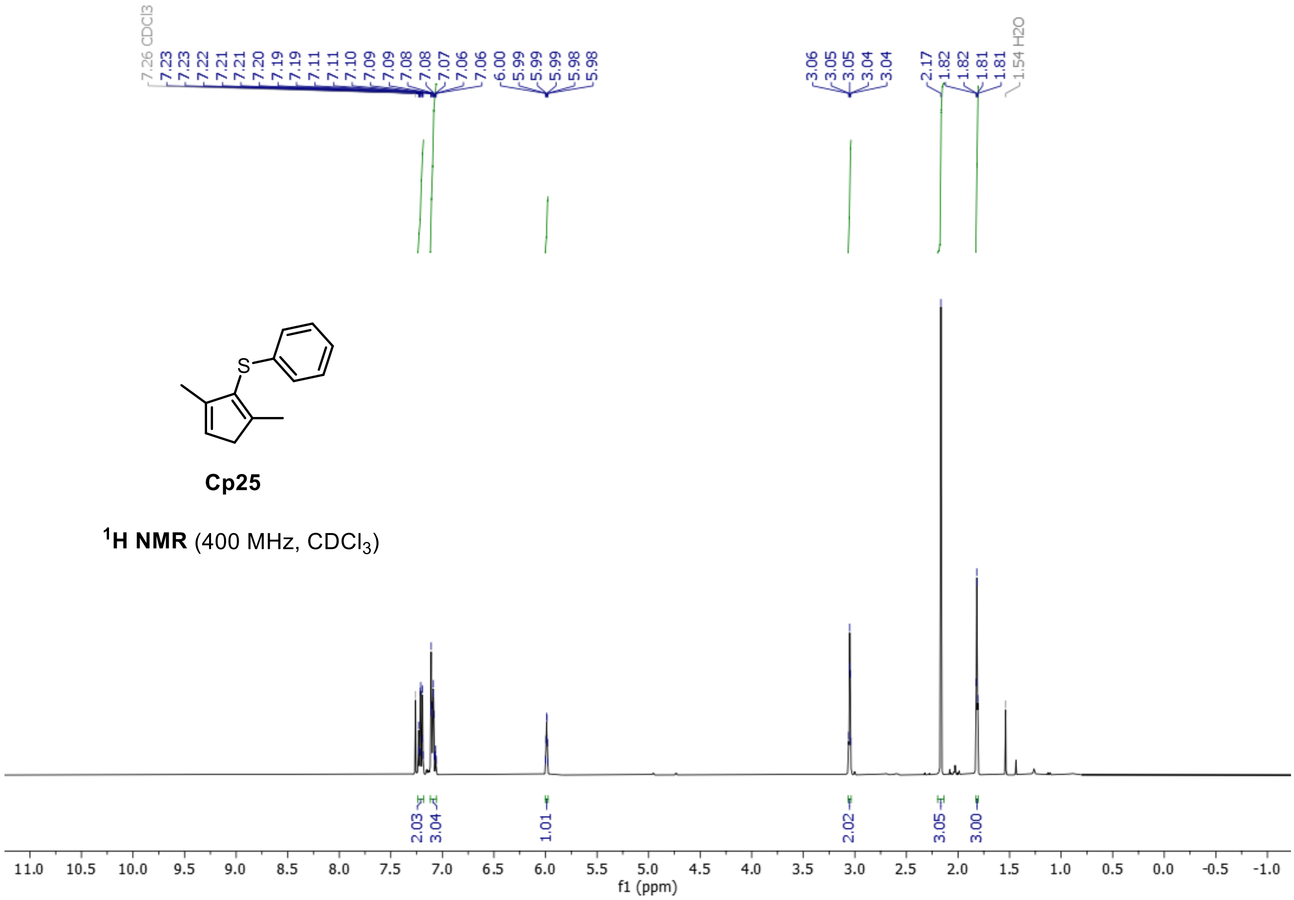
7.26 CDCl3
7.23
7.23
7.22
7.21
7.21
7.20
7.19
7.19
7.11
7.11
7.10
7.09
7.09
7.08
7.08
7.07
7.06
7.06
6.00
5.99
5.99
5.99
5.98
5.98
3.06
3.05
3.05
3.04
3.04
2.17
1.82
1.82
1.81
1.81
1.54 H2O
S
Cp25
^{1}H NMR (400 MHz, $CDCl_3$)
2.03
3.04
1.01
2.02
3.05
3.00
f1 (ppm)

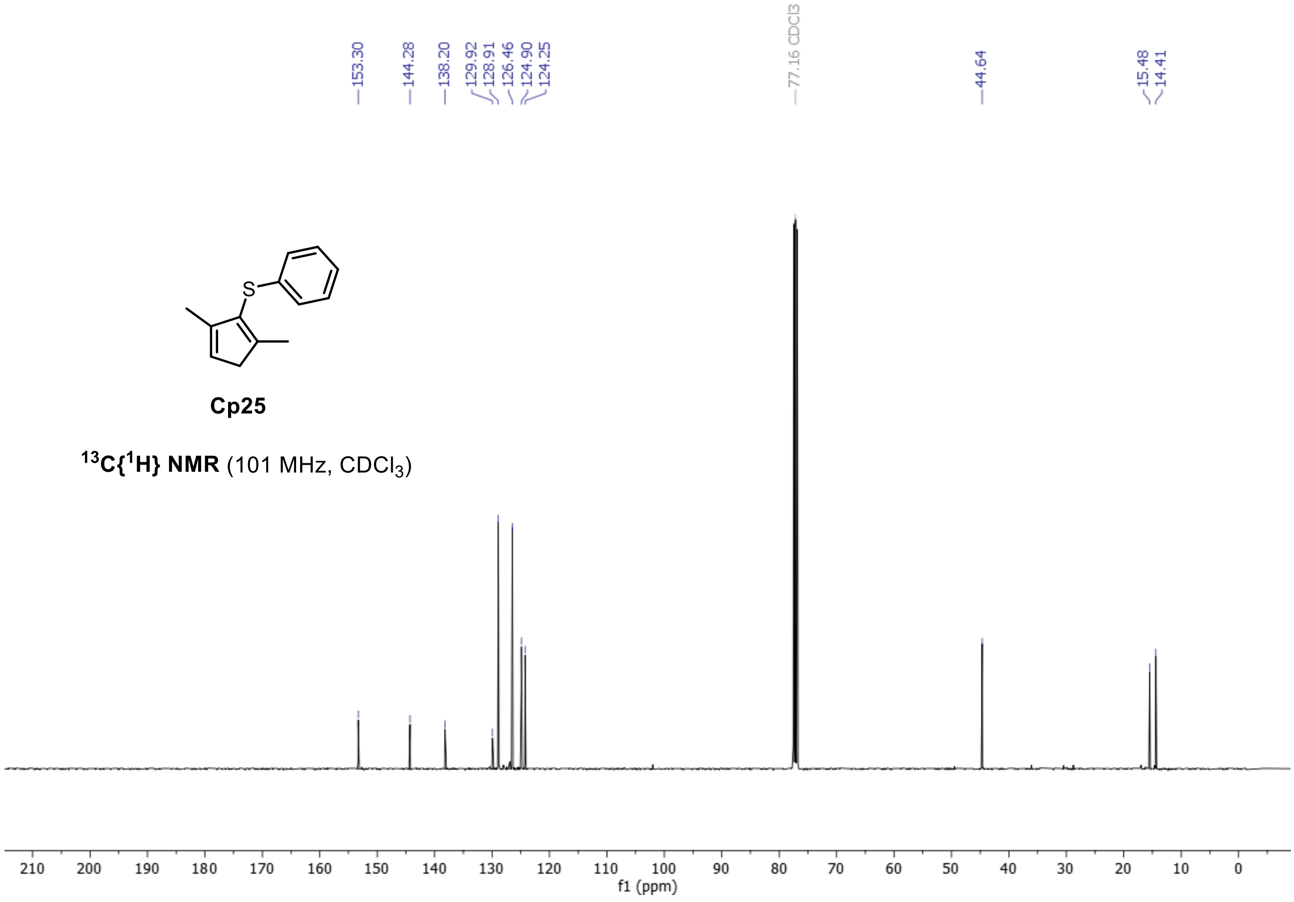
153.30
144.28
138.20
129.92
128.91
126.46
124.90
124.25
77.16 CDCl3
44.64
15.48
14.41
S
Cp25
$^{13}C\{^1H\}$ NMR (101 MHz, $CDCl_3$)
f1 (ppm)

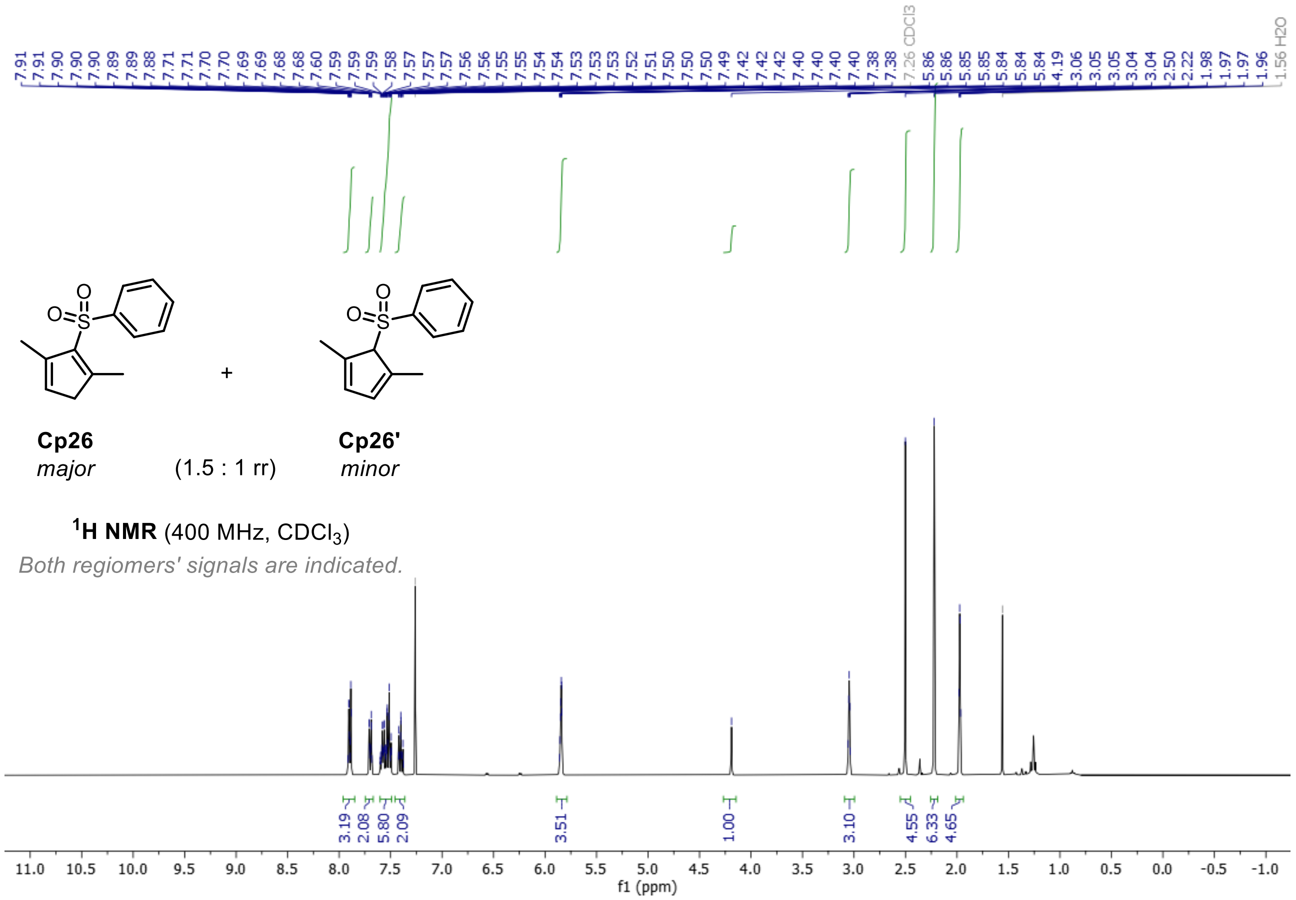
7.91 7.91 7.90 7.90 7.90 7.89 7.89 7.88 7.71 7.71 7.70 7.70 7.69 7.69 7.68 7.68 7.60 7.59 7.59 7.59 7.58 7.57 7.57 7.57 7.56 7.56 7.55 7.55 7.54 7.54 7.53 7.53 7.53 7.52 7.51 7.50 7.50 7.50 7.49 7.42 7.42 7.42 7.40 7.40 7.40 7.38 7.38 7.26 CDCl3 5.86 5.86 5.85 5.85 5.84 5.84 5.84 4.19 3.06 3.05 3.05 3.04 3.04 2.50 2.22 1.98 1.97 1.97 1.96 1.56 H2O
Cp26
major
(1.5 : 1 rr)
Cp26'
minor
1H NMR (400 MHz, CDCl3)
Both regiomers' signals are indicated.
3.19 2.08 5.80 2.09 3.51 1.00 3.10 4.55 6.33 4.65
11.0 10.5 10.0 9.5 9.0 8.5 8.0 7.5 7.0 6.5 6.0 5.5 5.0 4.5 4.0 3.5 3.0 2.5 2.0 1.5 1.0 0.5 0.0 -0.5 -1.0
f1 (ppm)

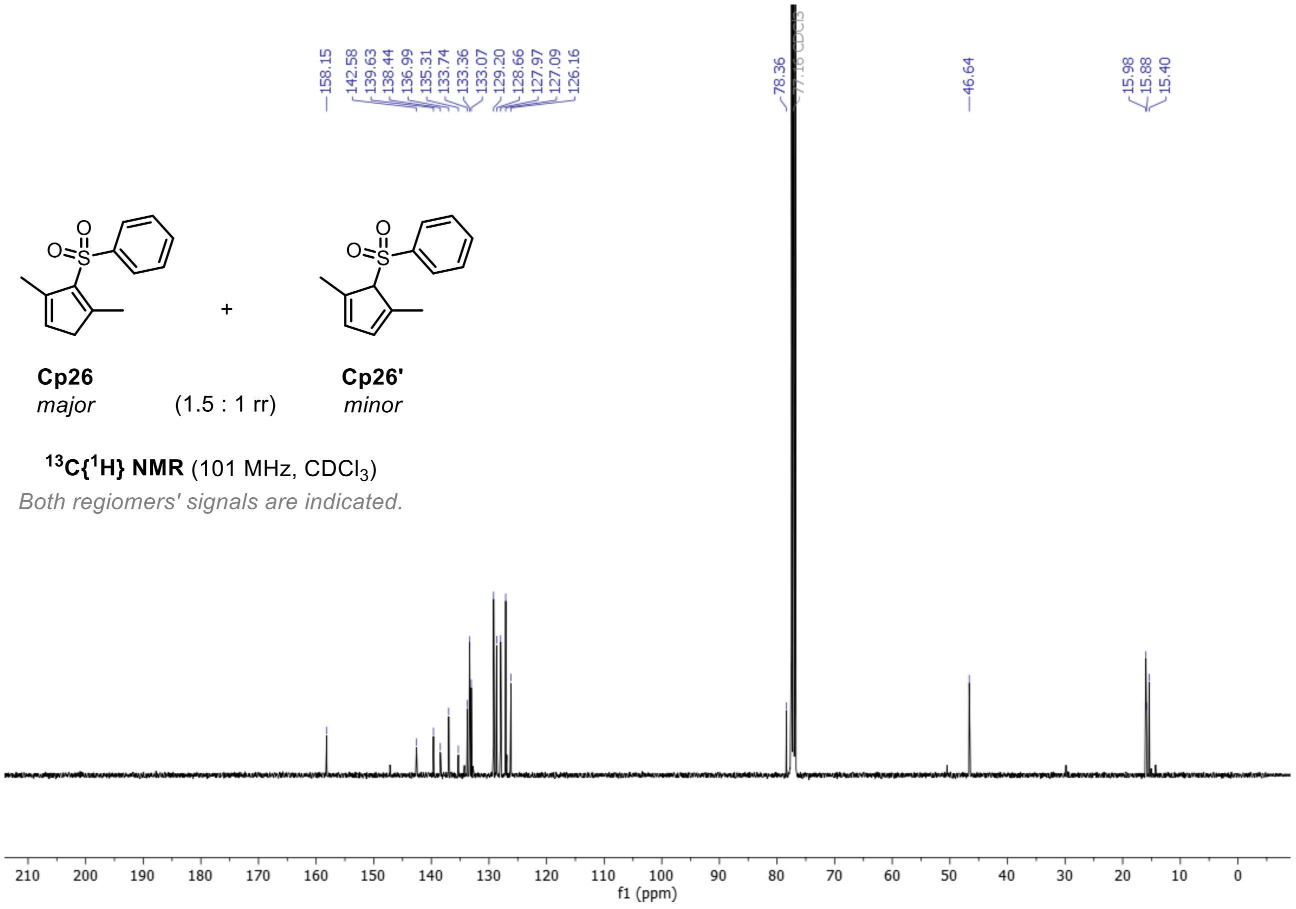
158.15 142.58 139.63 138.44 136.99 135.31 133.74 133.36 133.07 129.20 128.66 127.97 127.09 126.16 78.36 77.16 CDCl3 46.64 15.98 15.88 15.40
Cp26
major
(1.5 : 1 rr)
Cp26'
minor
13C{1H} NMR (101 MHz, CDCl3)
Both regiomers' signals are indicated.
210 200 190 180 170 160 150 140 130 120 110 100 90 80 70 60 50 40 30 20 10 0
f1 (ppm)

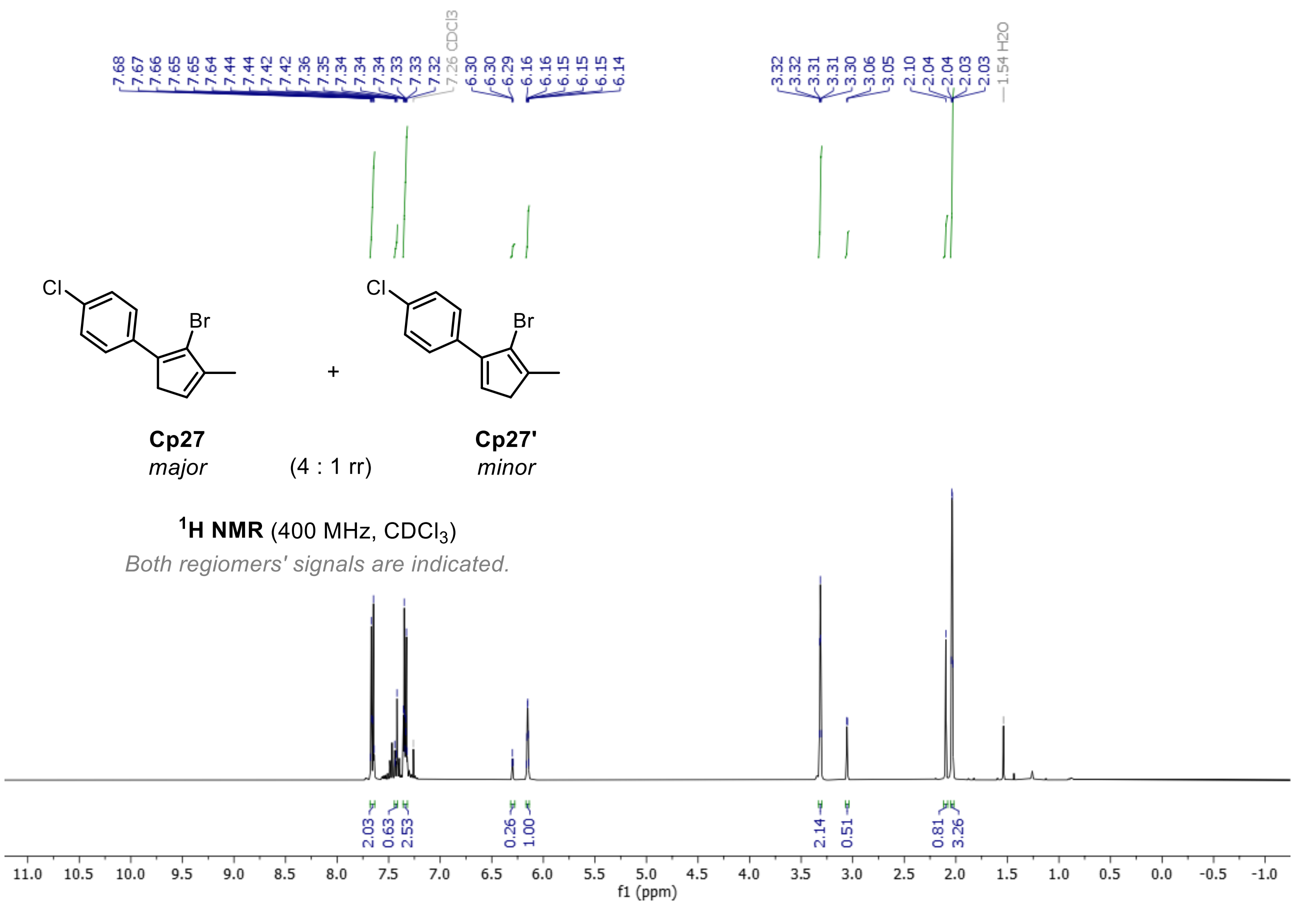
7.68 7.67 7.66 7.65 7.65 7.64 7.44 7.44 7.42 7.42 7.36 7.35 7.34 7.34 7.34 7.33 7.33 7.32 7.26 CDCl3 6.30 6.30 6.29 6.16 6.16 6.15 6.15 6.15 6.14
3.32 3.32 3.31 3.31 3.30 3.06 3.05 2.10 2.04 2.04 2.03 2.03 1.54 H2O
Cl
Br
+
Cp27
major
(4 : 1 rr)
Cp27'
minor
1H NMR (400 MHz, CDCl3)
Both regiomers' signals are indicated.
2.03 0.63 2.53 0.26 1.00 2.14 0.51 0.81 3.26
11.0 10.5 10.0 9.5 9.0 8.5 8.0 7.5 7.0 6.5 6.0 5.5 5.0 4.5 4.0 3.5 3.0 2.5 2.0 1.5 1.0 0.5 0.0 -0.5 -1.0
f1 (ppm)

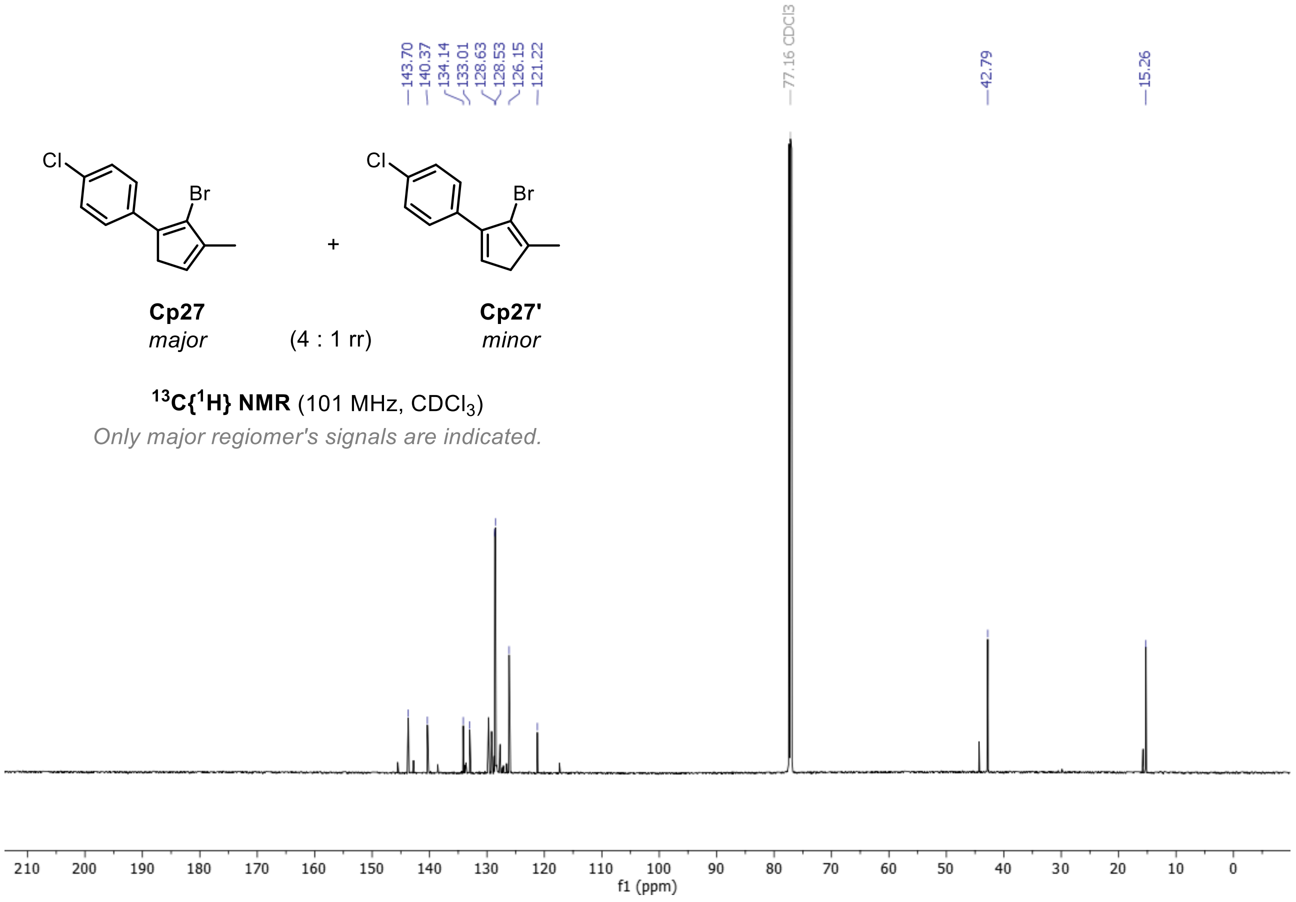
143.70 140.37 134.14 133.01 128.63 128.53 126.15 121.22
77.16 CDCl3
42.79
15.26
Cl
Br
+
Cp27
major
(4 : 1 rr)
Cp27'
minor
13C{1H} NMR (101 MHz, CDCl3)
Only major regiomer's signals are indicated.
210 200 190 180 170 160 150 140 130 120 110 100 90 80 70 60 50 40 30 20 10 0
f1 (ppm)

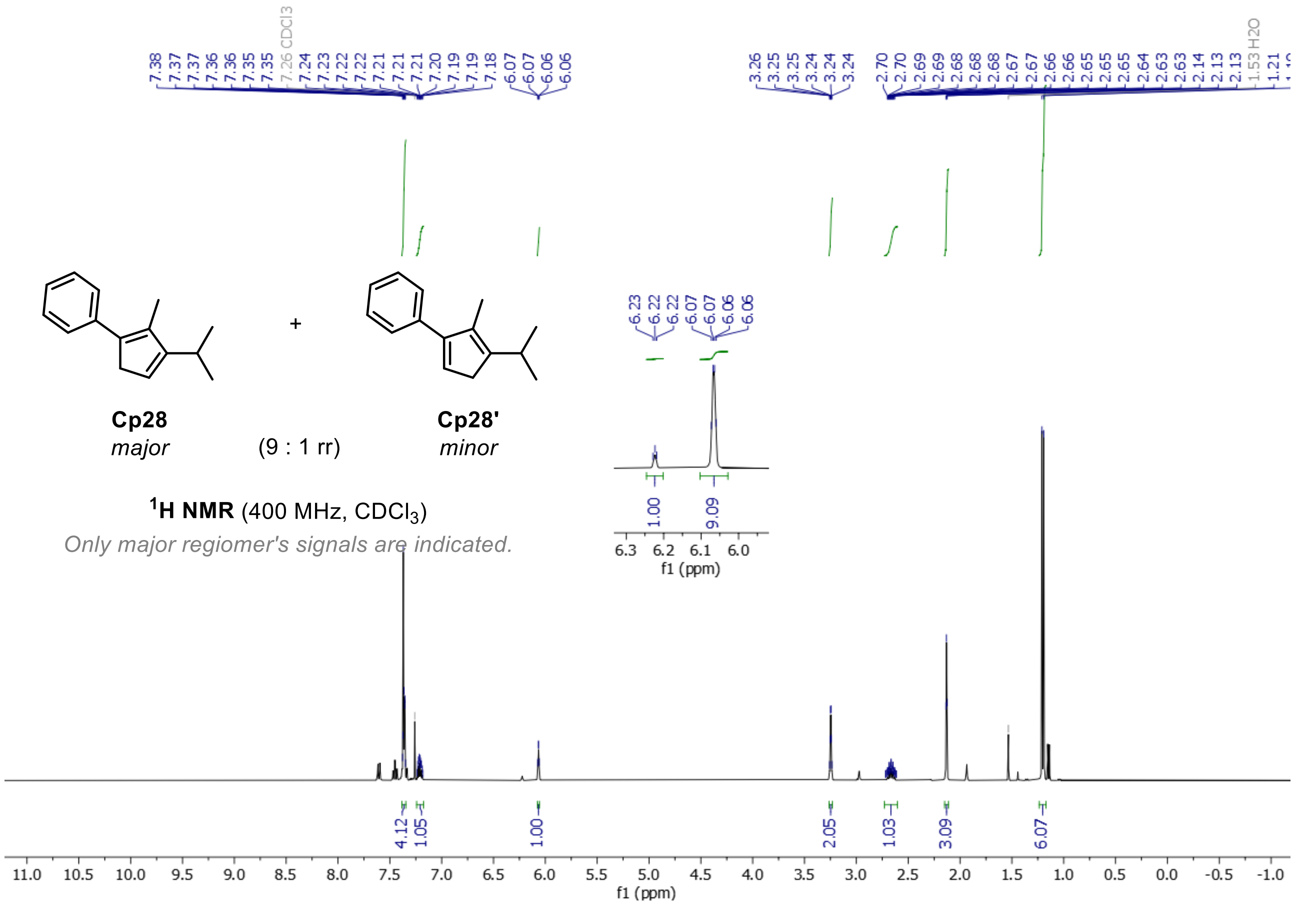
7.38
7.37
7.37
7.36
7.36
7.35
7.35
7.26 CDCl3
7.24
7.23
7.22
7.22
7.21
7.21
7.21
7.20
7.19
7.19
7.18
6.07
6.07
6.06
6.06
3.26
3.25
3.25
3.24
3.24
3.24
2.70
2.70
2.69
2.69
2.68
2.68
2.68
2.67
2.67
2.66
2.66
2.65
2.65
2.65
2.64
2.63
2.63
2.14
2.13
2.13
1.53 H2O
1.21
Cp28
major
+
(9 : 1 rr)
Cp28'
minor
1H NMR (400 MHz, CDCl3)
Only major regiomer's signals are indicated.
6.23
6.22
6.22
6.07
6.07
6.06
6.06
1.00
9.09
f1 (ppm)
4.12
1.05
1.00
2.05
1.03
3.09
6.07
f1 (ppm)

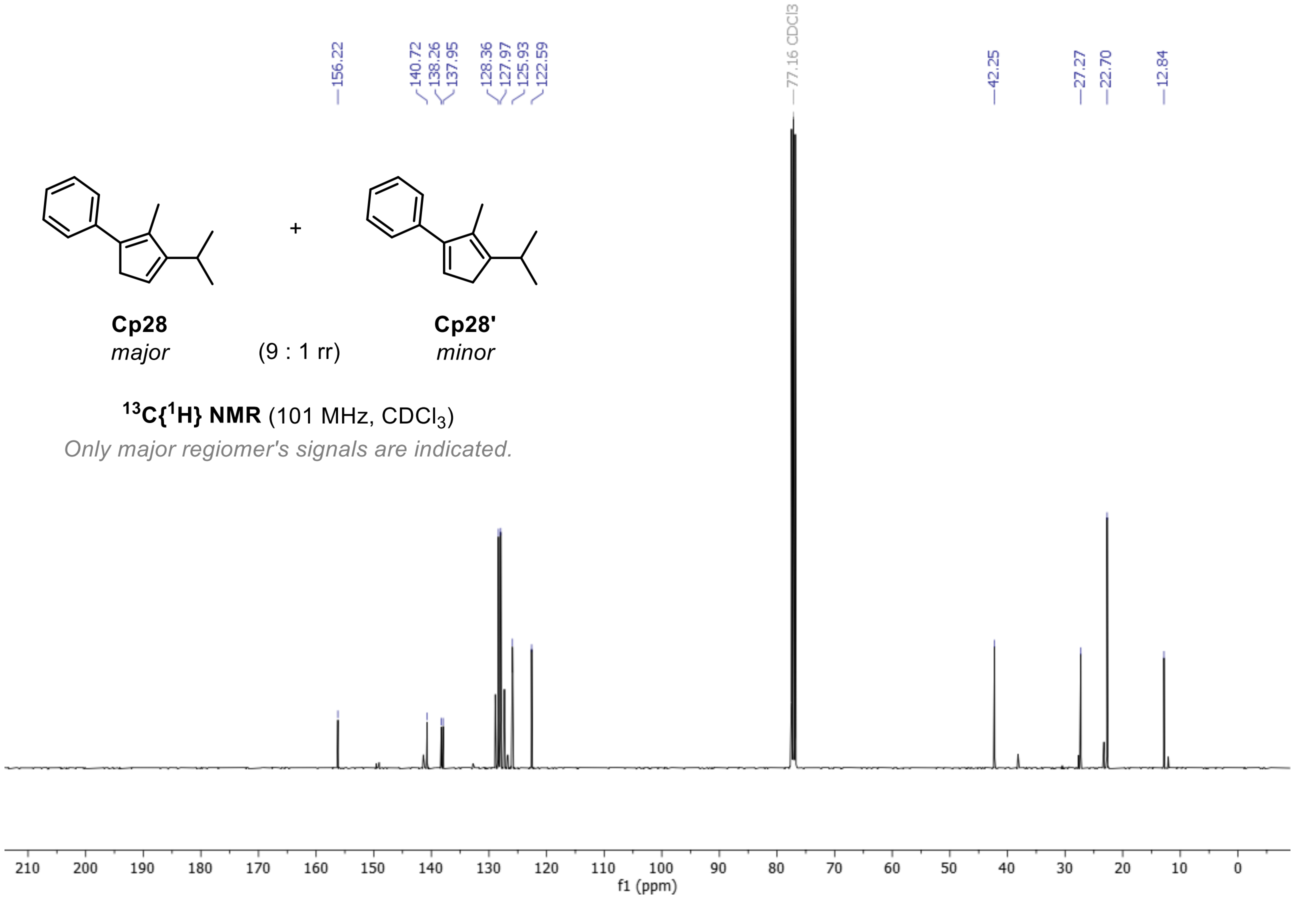
156.22
140.72
138.26
137.95
128.36
127.97
125.93
122.59
77.16 CDCl3
42.25
27.27
22.70
12.84
Cp28
major
+
(9 : 1 rr)
Cp28'
minor
13C{1H} NMR (101 MHz, CDCl3)
Only major regiomer's signals are indicated.
f1 (ppm)

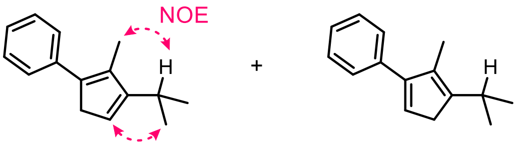

Cp28 *major* (9 : 1 rr) **Cp28'** *minor*

2D NOESY (400 MHz, $CDCl_3$)

Key interactions of the major regiomer are indicated.

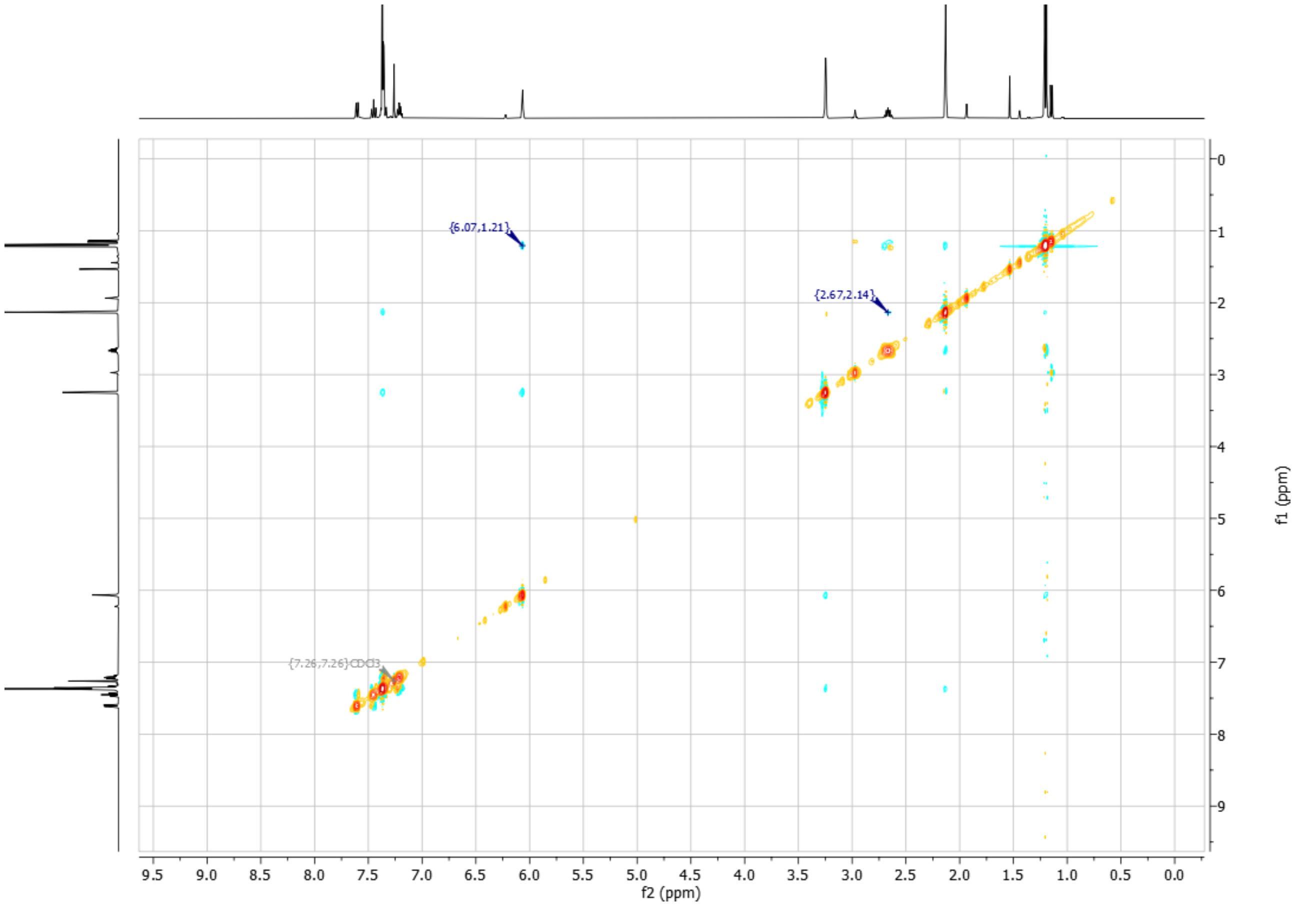

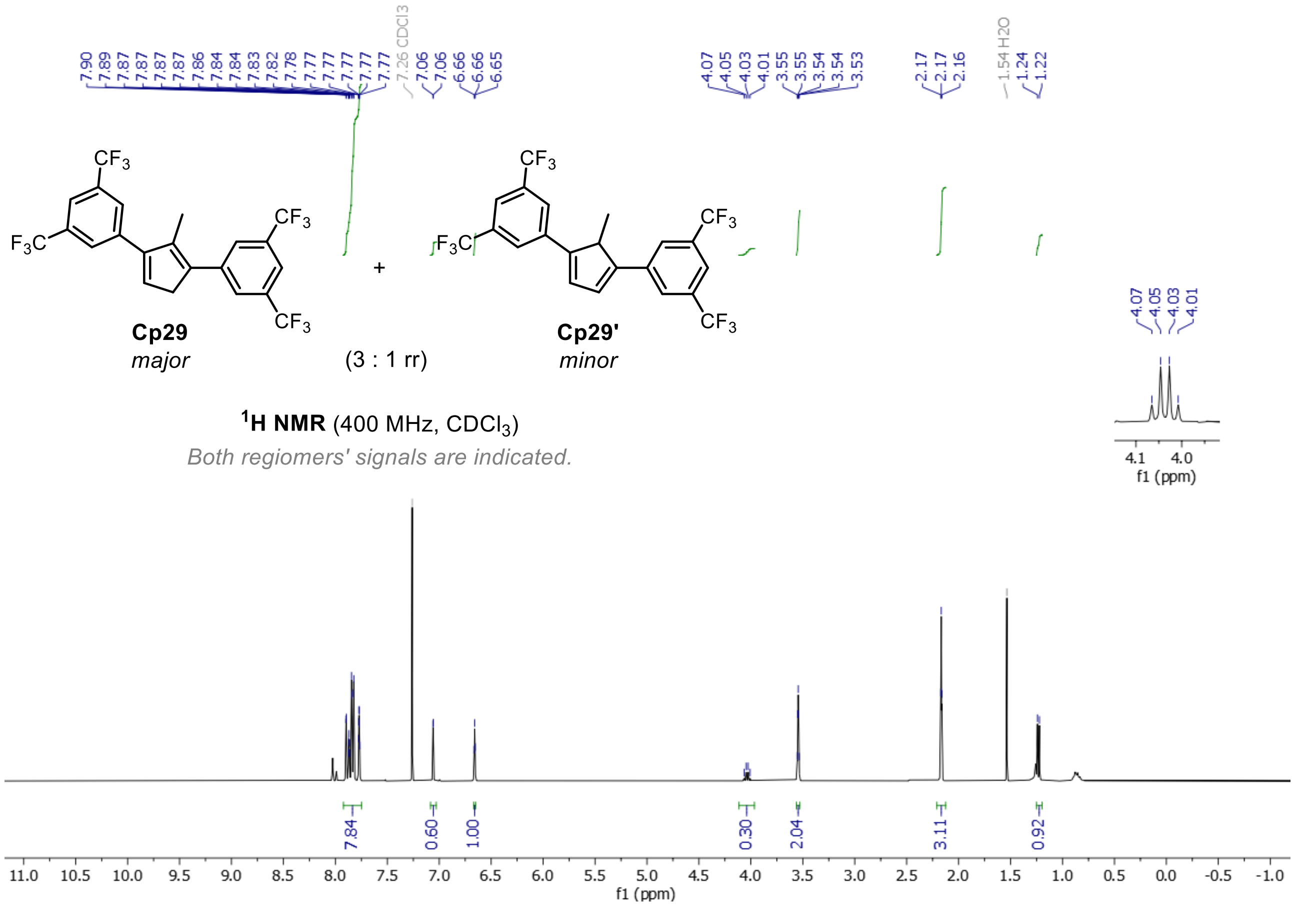
7.90 7.89 7.87 7.87 7.87 7.86 7.84 7.84 7.83 7.82 7.78 7.77 7.77 7.77 7.77 7.77
7.26 CDCl3
7.06 7.06 6.66 6.66 6.65
4.07 4.05 4.03 4.01 3.55 3.55 3.54 3.54 3.53
2.17 2.17 2.16
1.54 H2O
1.24 1.22
CF3
F3C
Cp29
major
(3 : 1 rr)
Cp29'
minor
¹H NMR (400 MHz, CDCl3)
Both regiomers' signals are indicated.
7.84 0.60 1.00 0.30 2.04 3.11 0.92
f1 (ppm)

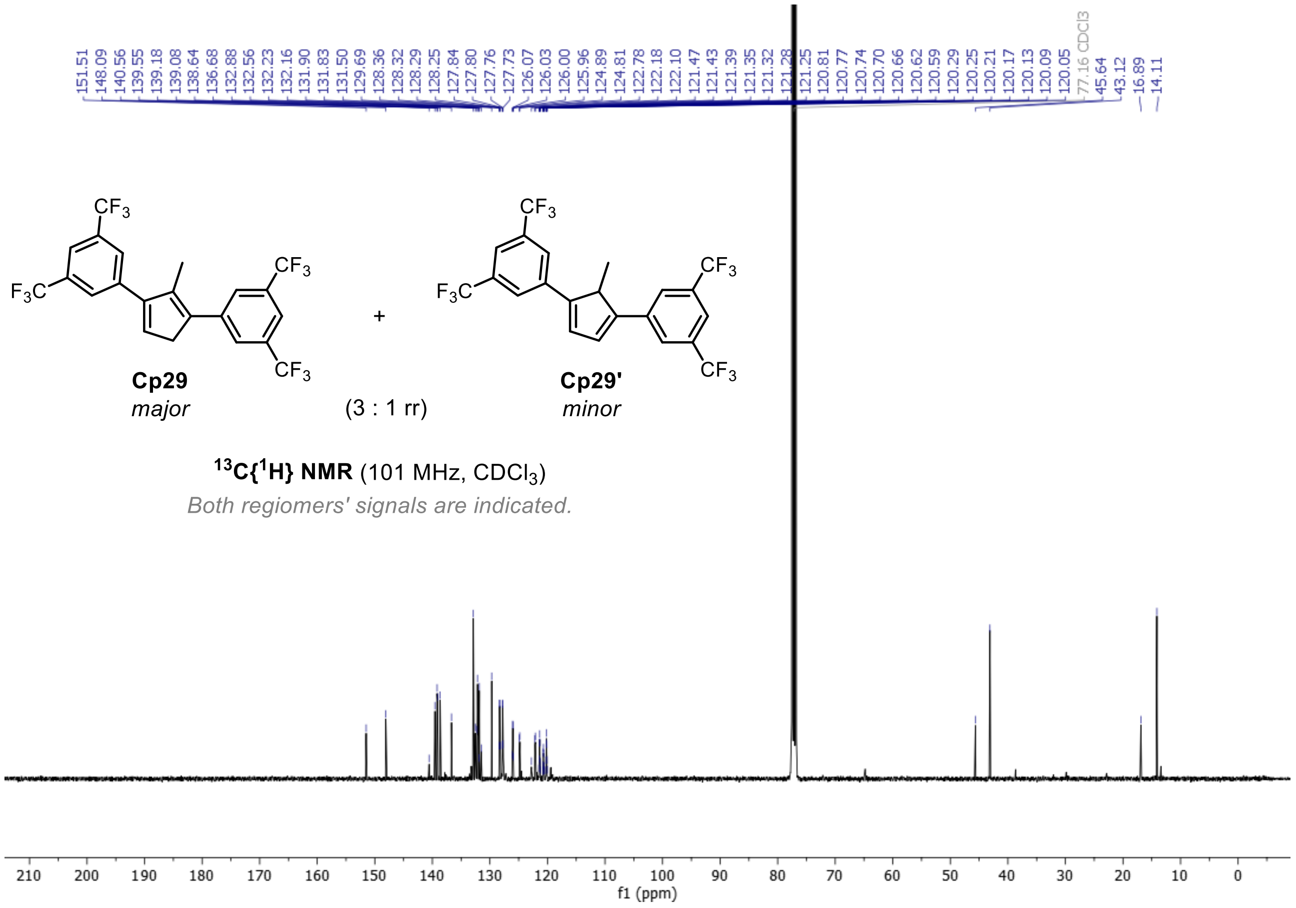
151.51 148.09 140.56 139.55 139.18 139.08 138.64 136.68 132.88 132.56 132.23 132.16 131.90 131.83 131.50 129.69 128.36 128.32 128.29 128.25 127.84 127.80 127.76 127.73 126.07 126.03 126.00 125.96 124.89 124.81 122.78 122.18 122.10 121.47 121.43 121.39 121.35 121.32 121.28 121.25 120.81 120.77 120.74 120.70 120.66 120.62 120.59 120.29 120.25 120.21 120.17 120.13 120.09 120.05
77.16 CDCl3
45.64 43.12 16.89 14.11
CF3
F3C
Cp29
major
(3 : 1 rr)
Cp29'
minor
¹³C{¹H} NMR (101 MHz, CDCl3)
Both regiomers' signals are indicated.
f1 (ppm)

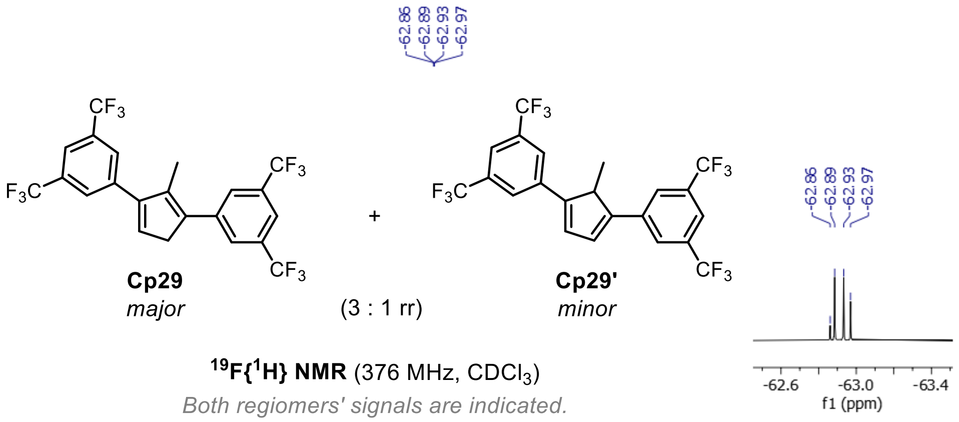

$^{19}F\{^{1}H\}$ NMR (376 MHz, $CDCl_3$)

Both regiomers' signals are indicated.

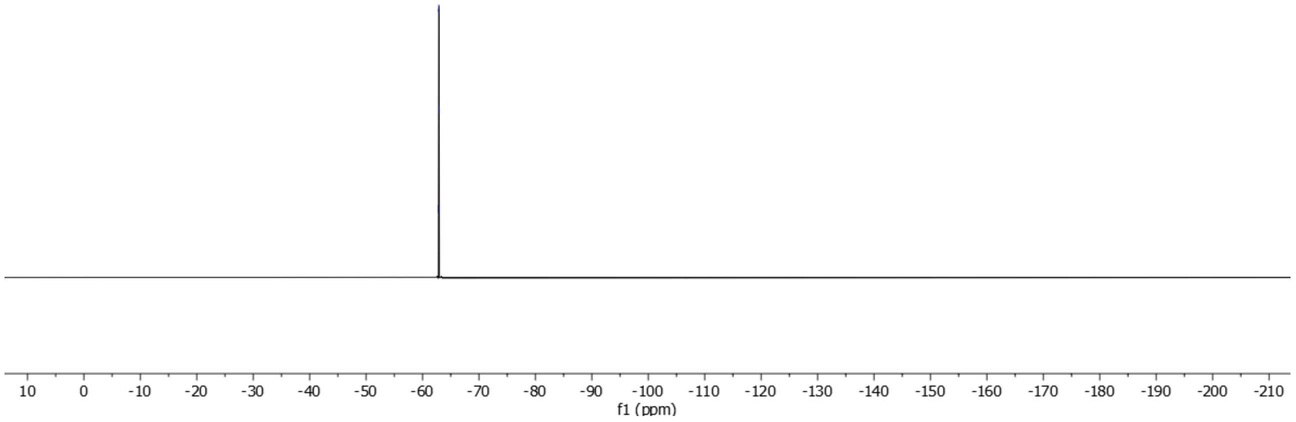

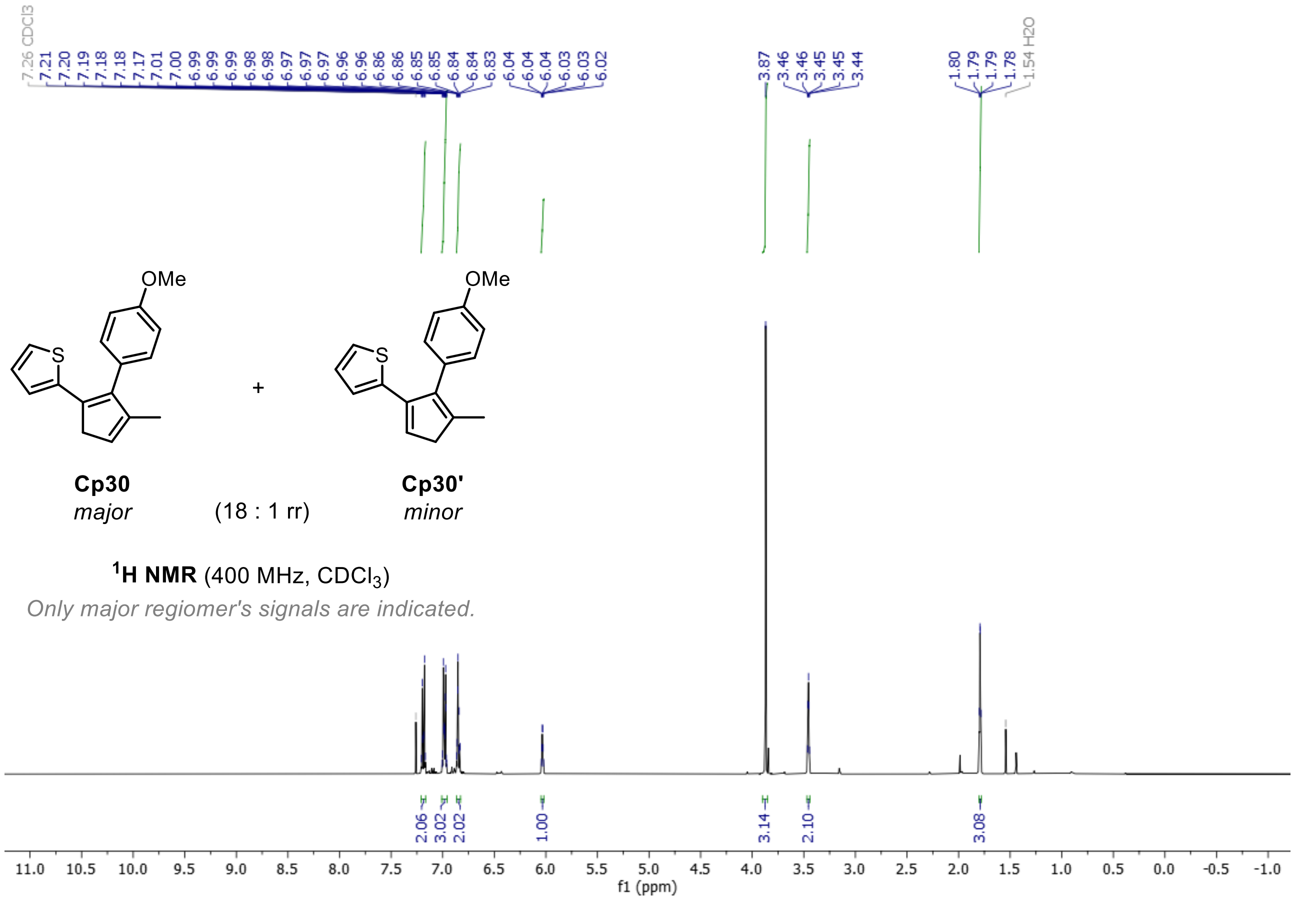
7.26 CDCl3
7.21
7.20
7.19
7.18
7.18
7.17
7.01
7.00
6.99
6.99
6.99
6.98
6.98
6.97
6.97
6.97
6.96
6.96
6.86
6.86
6.85
6.85
6.84
6.84
6.83
6.04
6.04
6.04
6.03
6.03
6.02
3.87
3.46
3.46
3.45
3.45
3.44
1.80
1.79
1.79
1.78
1.54 H2O
OMe
S
+
Cp30
major
(18 : 1 rr)
Cp30'
minor
1H NMR (400 MHz, CDCl3)
Only major regiomer's signals are indicated.
2.06
3.02
2.02
1.00
3.14
2.10
3.08
f1 (ppm)

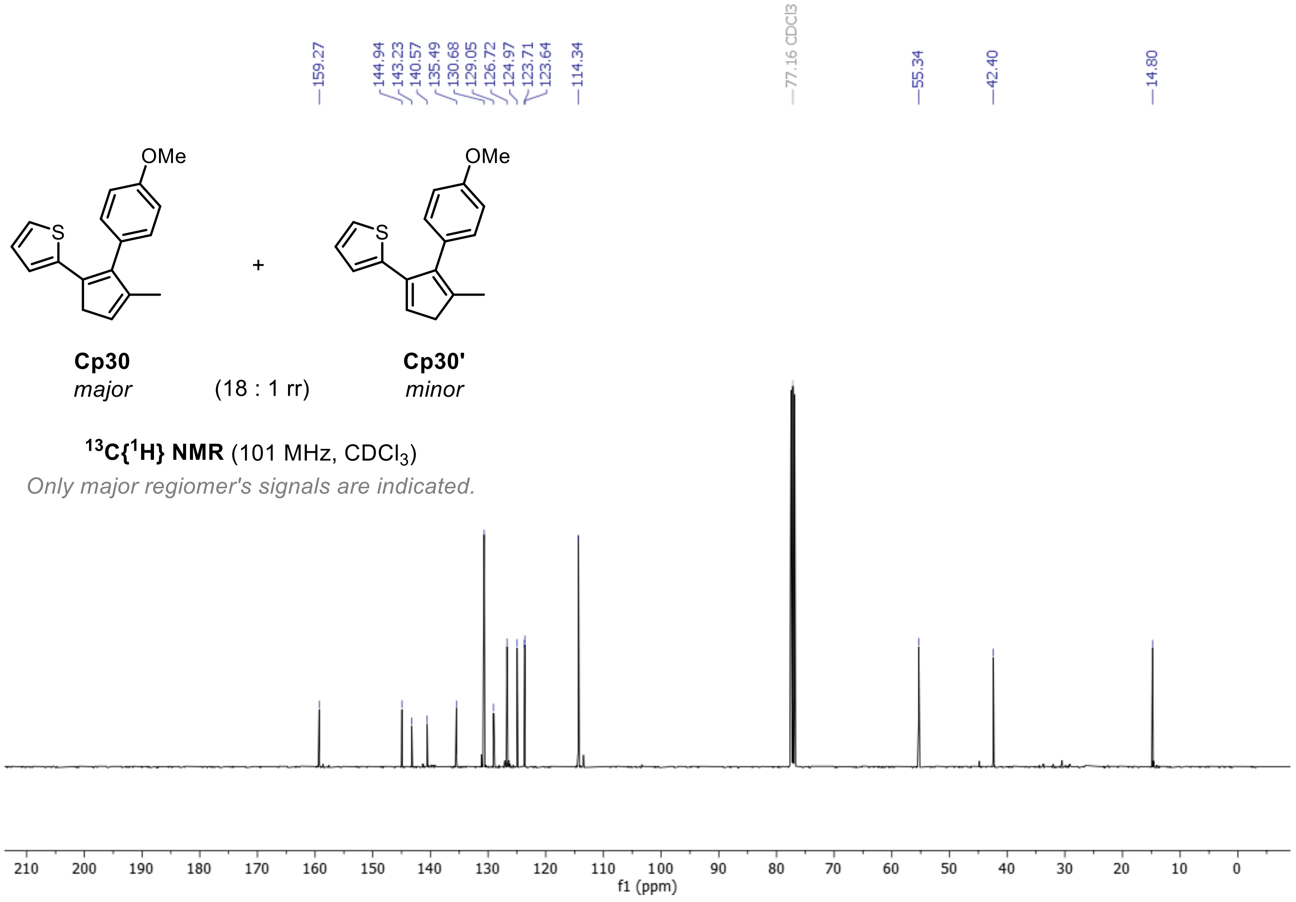
159.27
144.94
143.23
140.57
135.49
130.68
129.05
126.72
124.97
123.71
123.64
114.34
77.16 CDCl3
55.34
42.40
14.80
OMe
S
+
Cp30
major
(18 : 1 rr)
Cp30'
minor
13C{1H} NMR (101 MHz, CDCl3)
Only major regiomer's signals are indicated.
f1 (ppm)

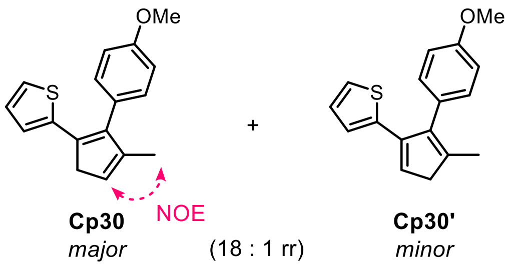

2D NOESY (400 MHz, $CDCl_3$)

Key interaction of the major regiomer is indicated.

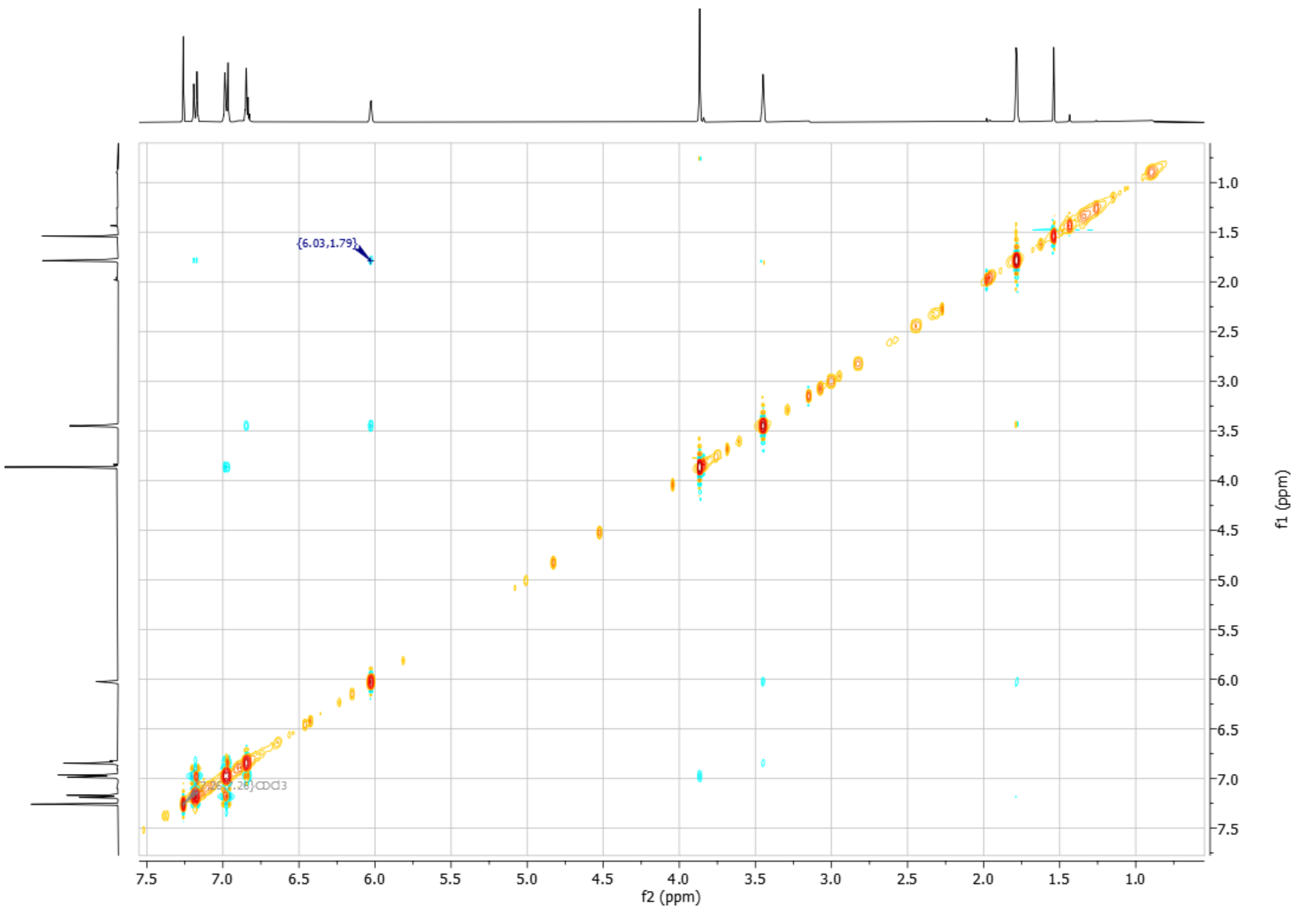

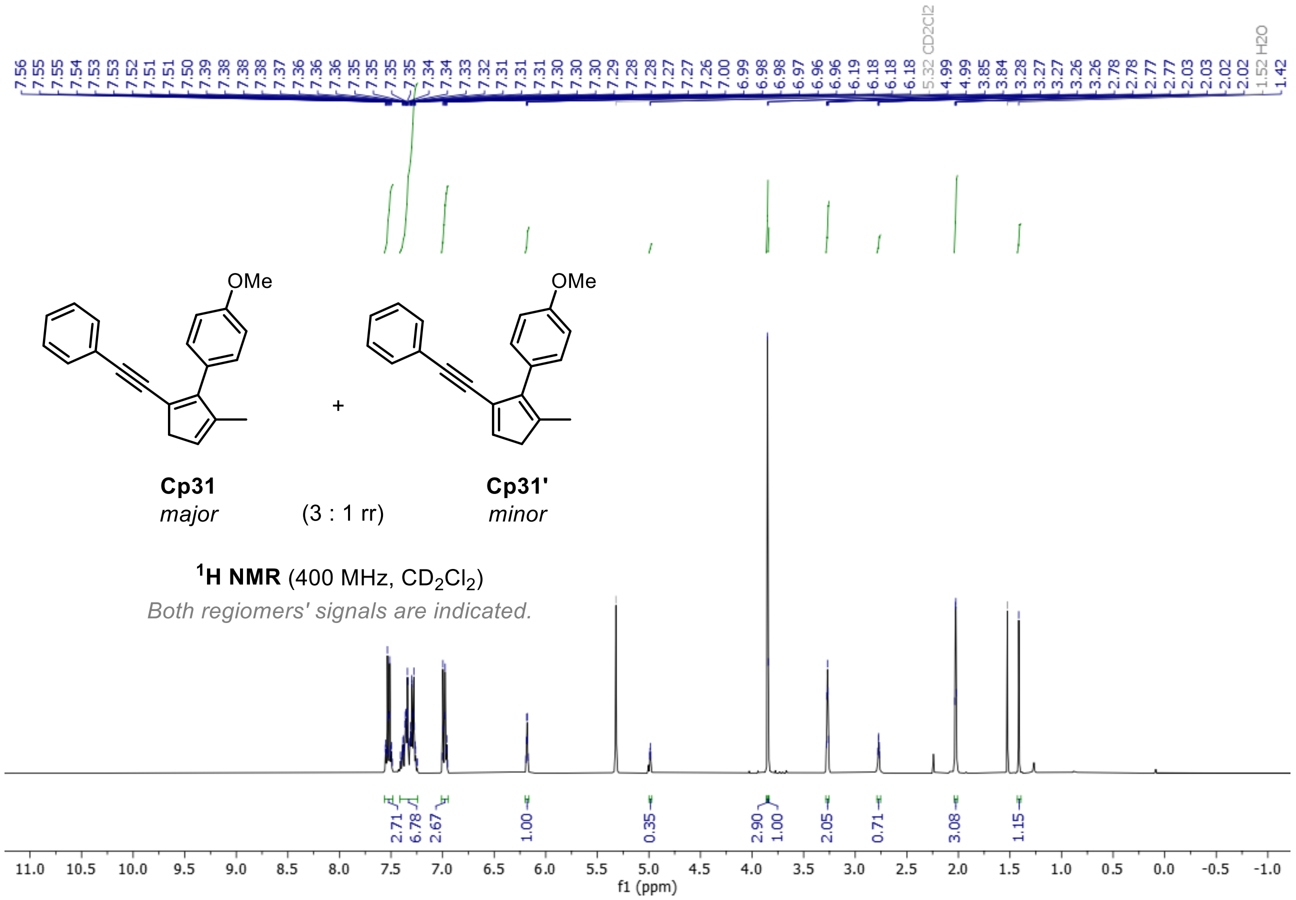
7.56 7.55 7.55 7.54 7.53 7.53 7.52 7.51 7.51 7.50 7.39 7.38 7.38 7.38 7.37 7.36 7.36 7.36 7.35 7.35 7.35 7.35 7.34 7.34 7.33 7.32 7.31 7.31 7.31 7.30 7.30 7.30 7.29 7.28 7.28 7.27 7.27 7.26 7.00 6.99 6.98 6.98 6.97 6.96 6.96 6.19 6.18 6.18 5.32 CD2Cl2 4.99 4.99 3.85 3.84 3.28 3.27 3.27 3.26 3.26 2.78 2.78 2.77 2.77 2.03 2.03 2.02 2.02 1.52 H2O 1.42
OMe
OMe
+
Cp31
major
(3 : 1 rr)
Cp31'
minor
^{1}H NMR (400 MHz, CD_2Cl_2)
Both regiomers' signals are indicated.
2.71
6.78
2.67
1.00
0.35
2.90
1.00
2.05
0.71
3.08
1.15
11.0 10.5 10.0 9.5 9.0 8.5 8.0 7.5 7.0 6.5 6.0 5.5 5.0 4.5 4.0 3.5 3.0 2.5 2.0 1.5 1.0 0.5 0.0 -0.5 -1.0
f1 (ppm)

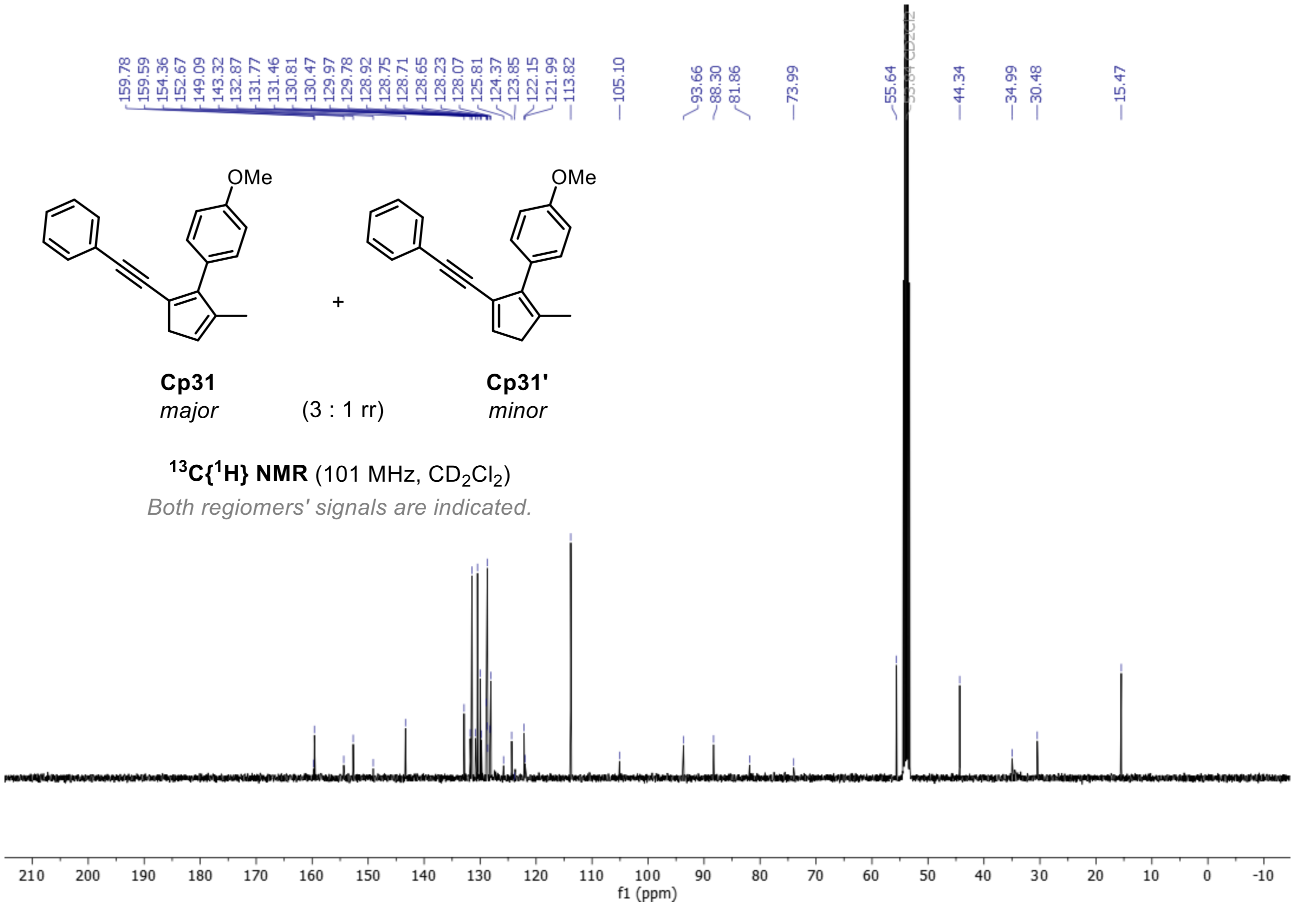
159.78 159.59 154.36 152.67 149.09 143.32 132.87 131.77 131.46 130.81 130.47 129.97 129.78 128.92 128.75 128.71 128.65 128.23 128.07 125.81 124.37 123.85 122.15 121.99 113.82
105.10
93.66 88.30 81.86
73.99
55.64
53.84 CD2Cl2
44.34
34.99 30.48
15.47
OMe
OMe
+
Cp31
major
(3 : 1 rr)
Cp31'
minor
^{13}C{^{1}H} NMR (101 MHz, CD_2Cl_2)
Both regiomers' signals are indicated.
210 200 190 180 170 160 150 140 130 120 110 100 90 80 70 60 50 40 30 20 10 0 -10
f1 (ppm)

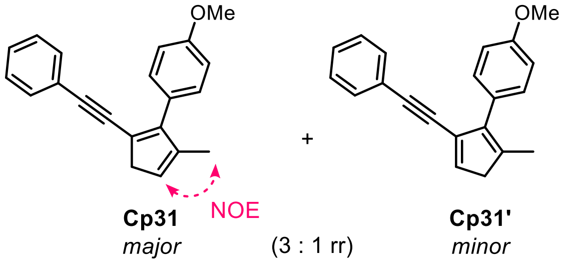

2D NOESY (400 MHz, CD_2Cl_2)

Key interaction of the major regiomer is indicated.

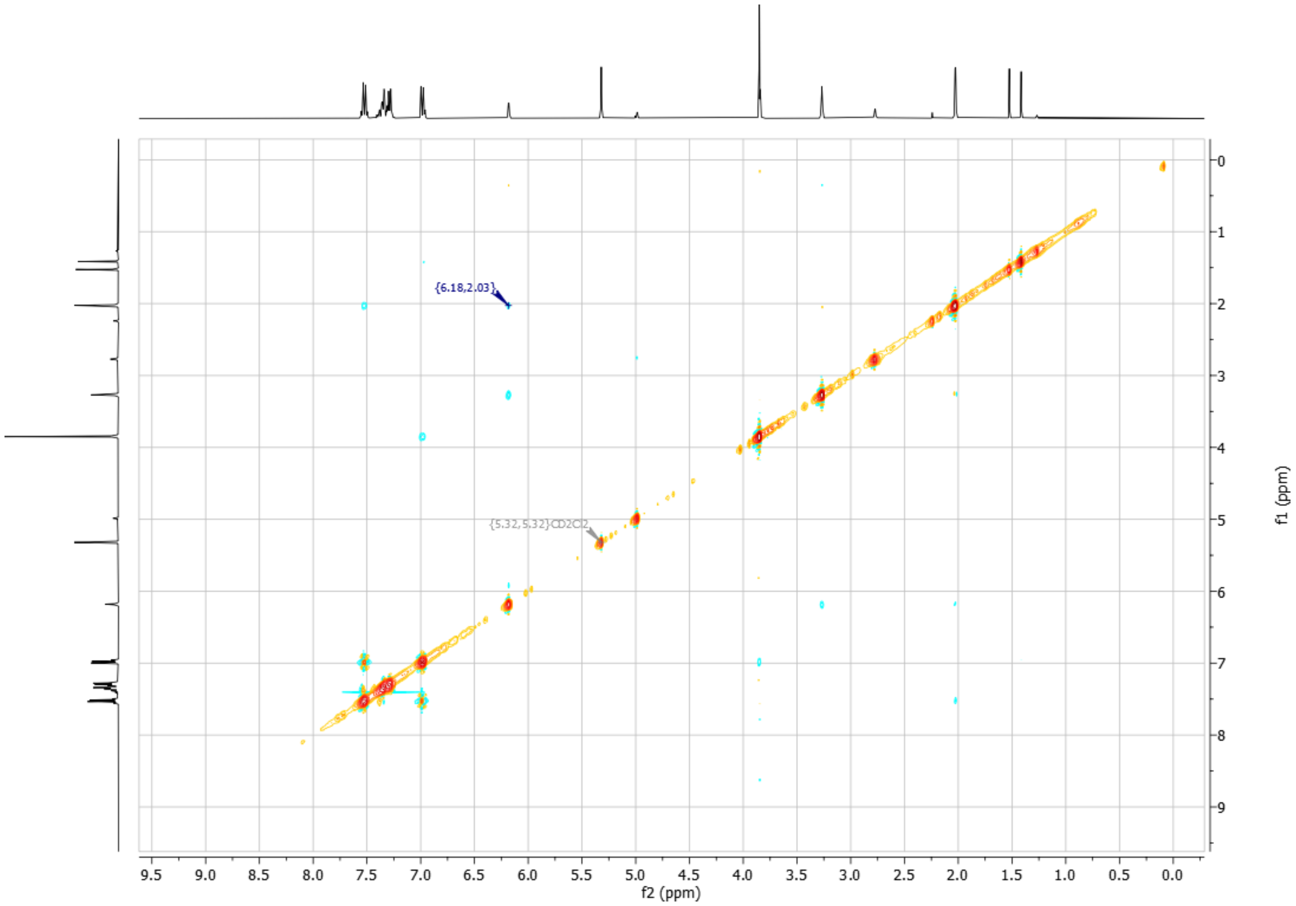

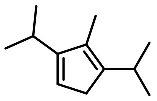

Cp32

^{1}H NMR (600 MHz, $CDCl_3$)

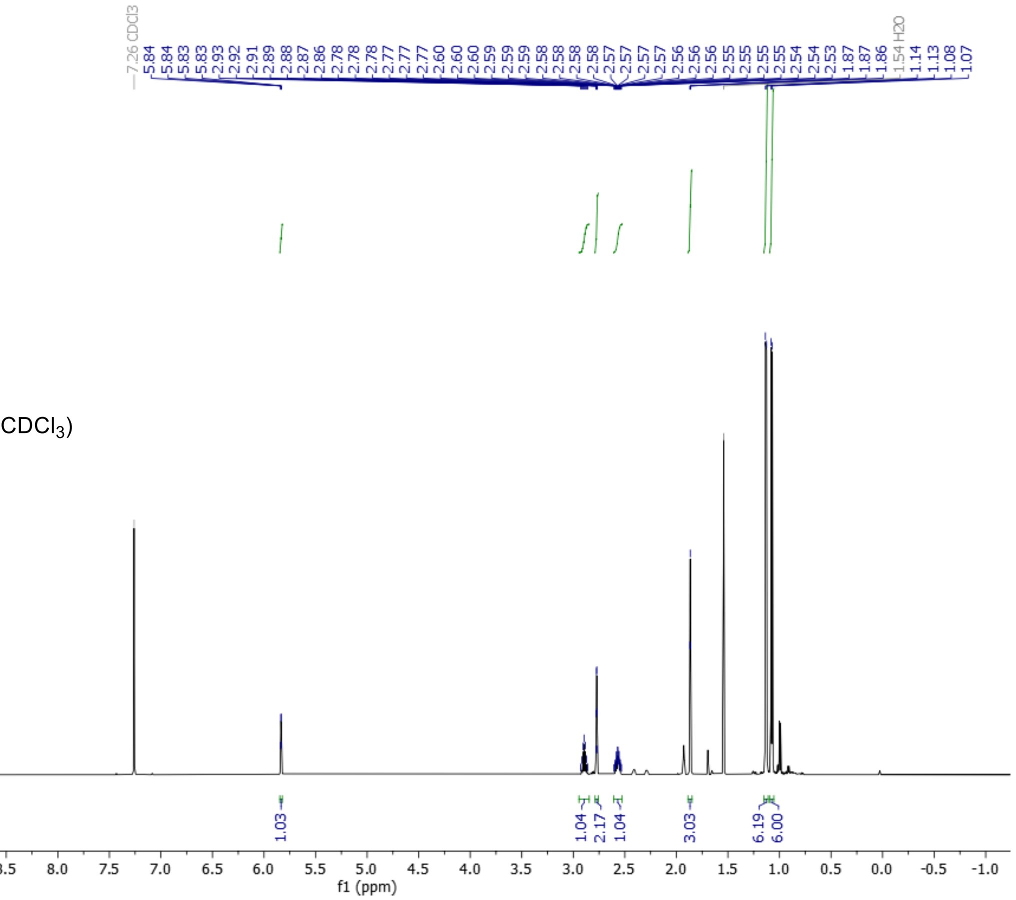

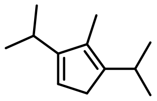

Cp32

^{13}C{^{1}H} NMR (151 MHz, $CDCl_3$)

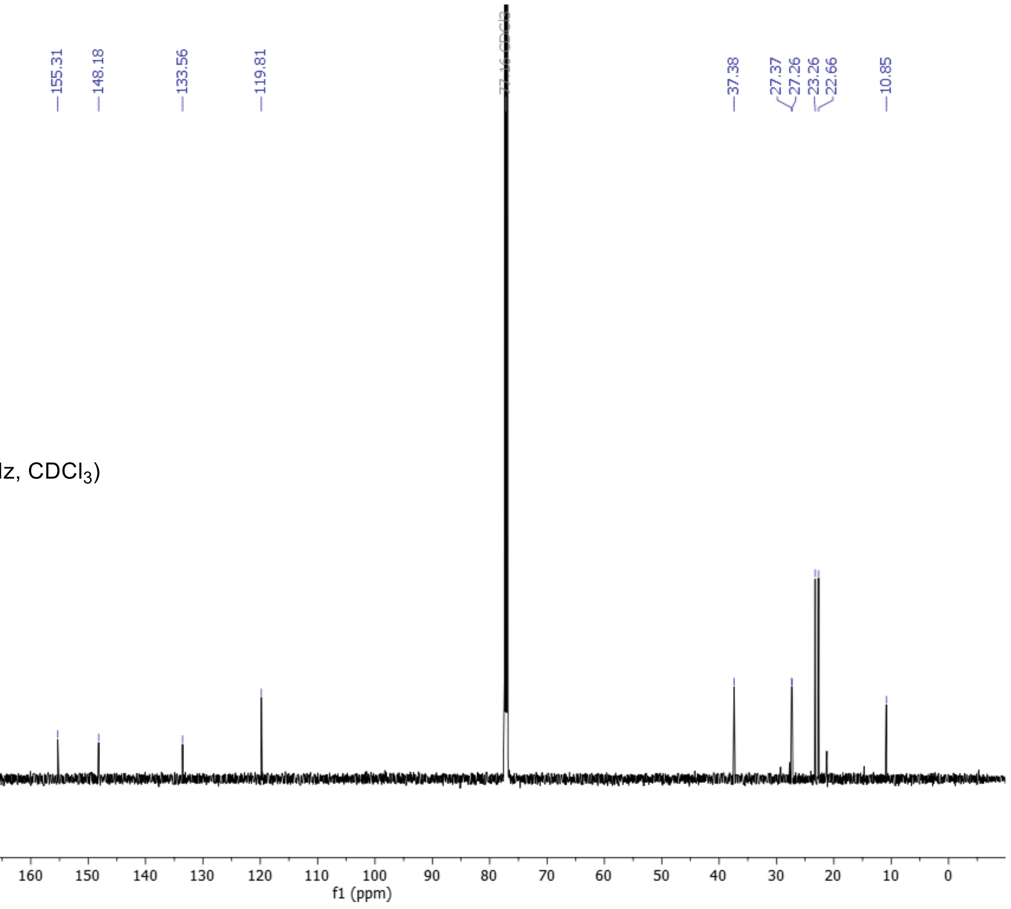

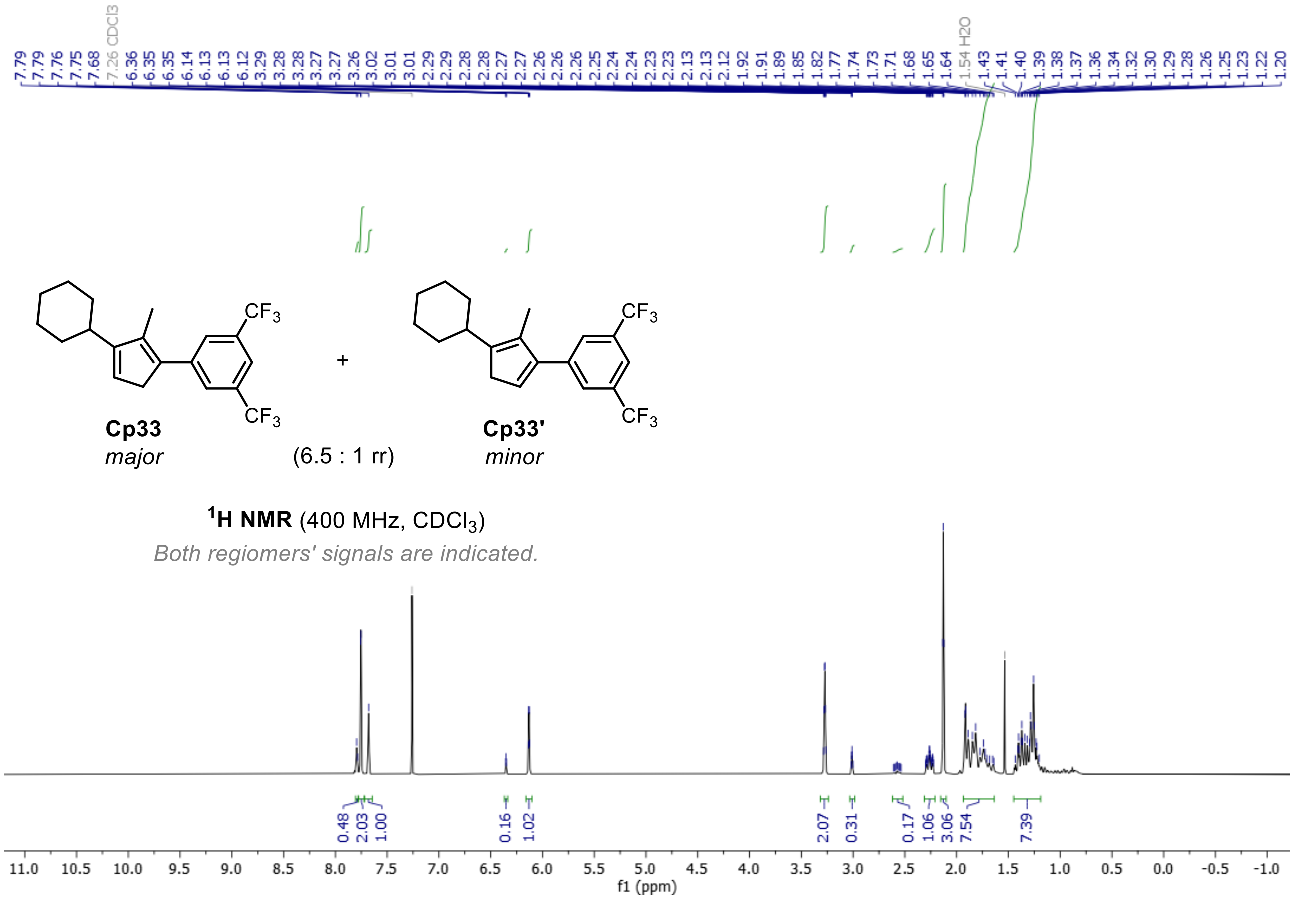

^{1}H NMR (400 MHz, $CDCl_3$)

Both regiomers' signals are indicated.

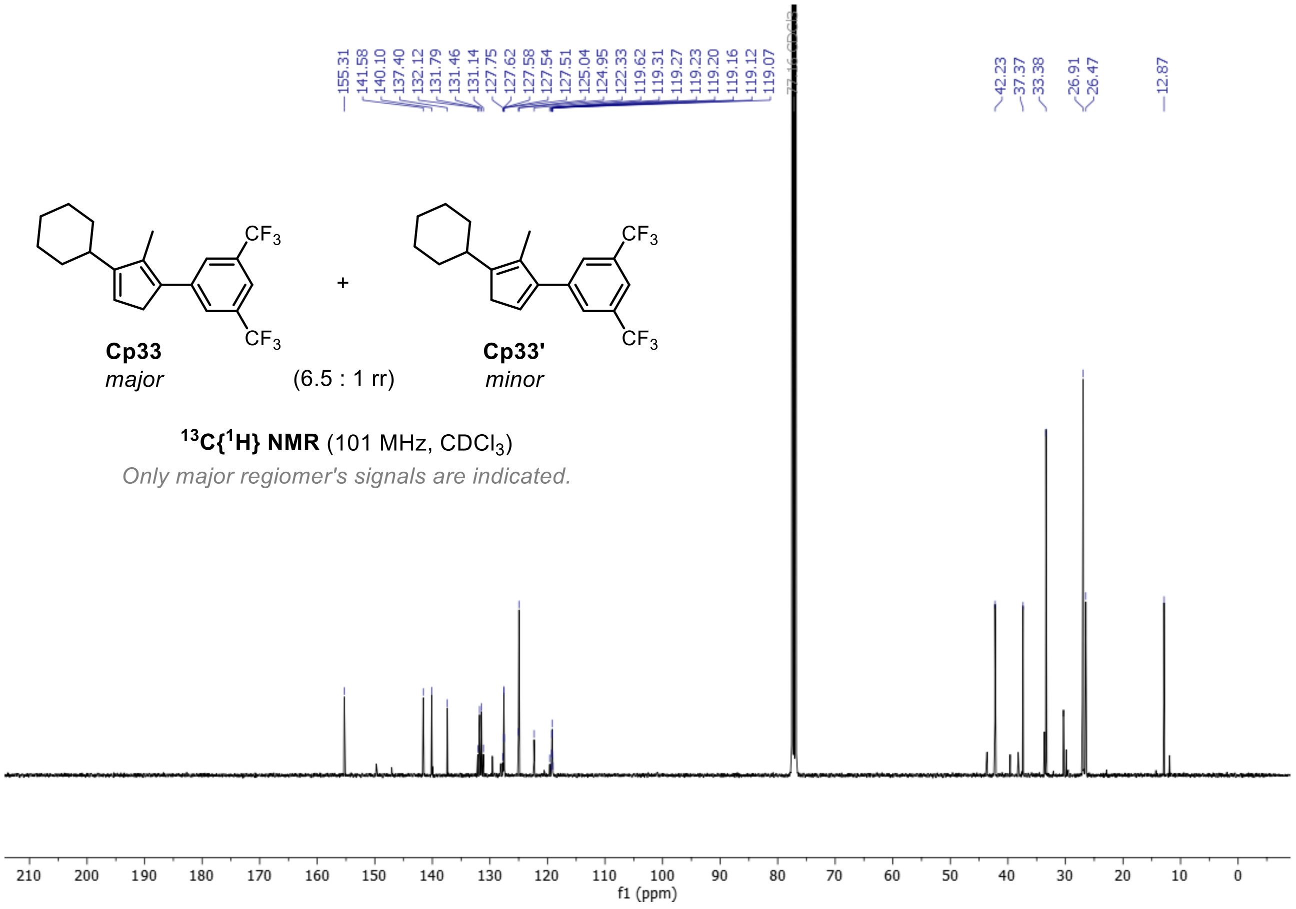

$^{13}C\{^1H\}$ NMR (101 MHz, $CDCl_3$)

Only major regiomer's signals are indicated.

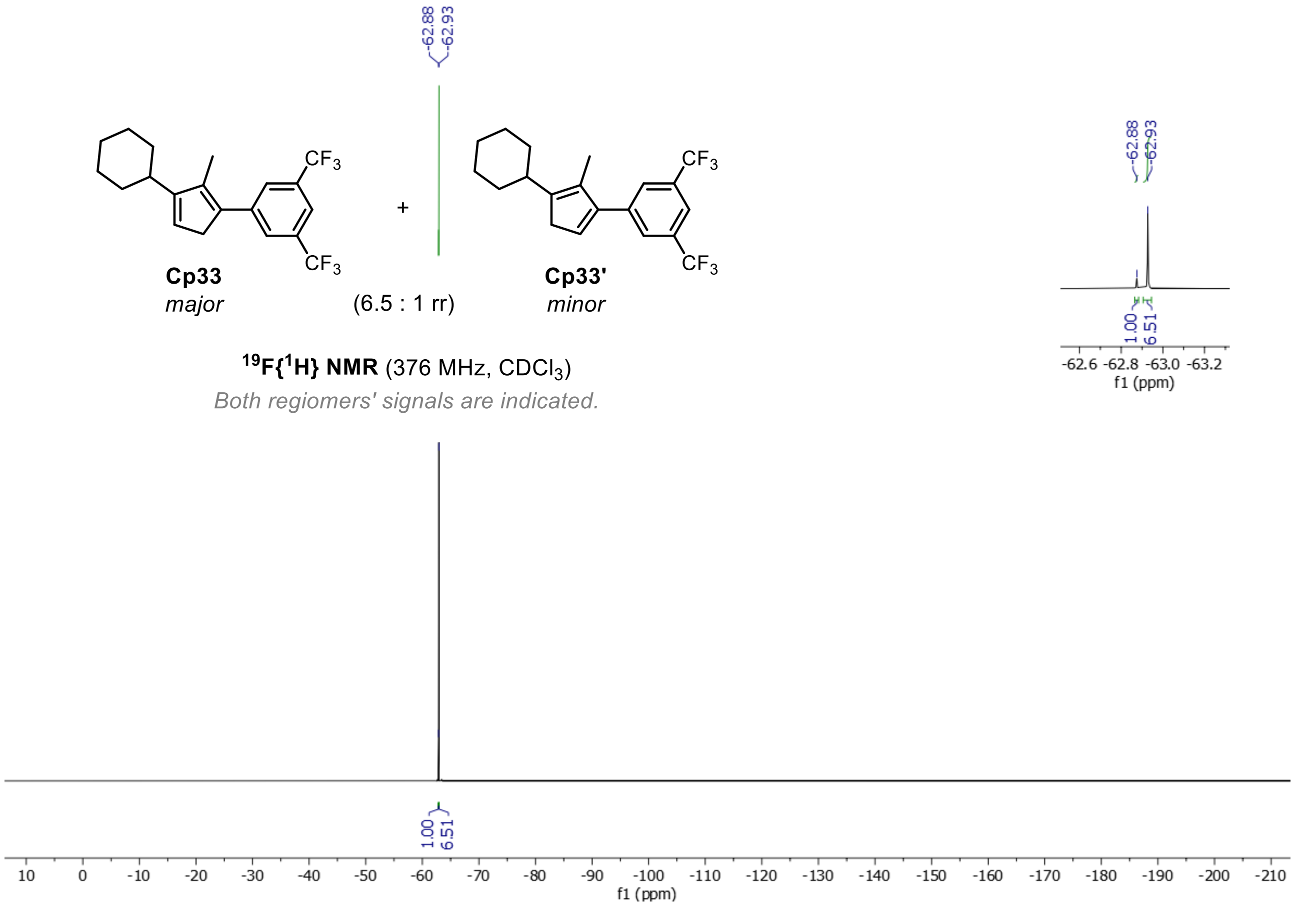
-62.88
-62.93
CF3
CF3
+
Cp33
major
(6.5 : 1 rr)
Cp33'
minor
19F{1H} NMR (376 MHz, CDCl3)
Both regiomers' signals are indicated.
1.00
6.51
-62.6 -62.8 -63.0 -63.2
f1 (ppm)
10 0 -10 -20 -30 -40 -50 -60 -70 -80 -90 -100 -110 -120 -130 -140 -150 -160 -170 -180 -190 -200 -210
f1 (ppm)

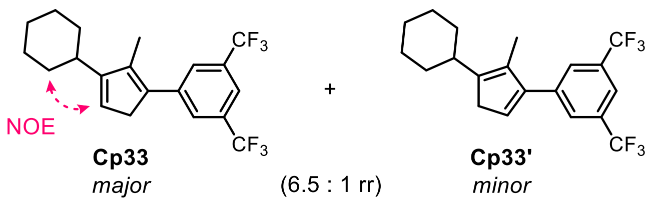

2D NOESY (400 MHz, $CDCl_3$)

Key interaction of the major regiomer is indicated.

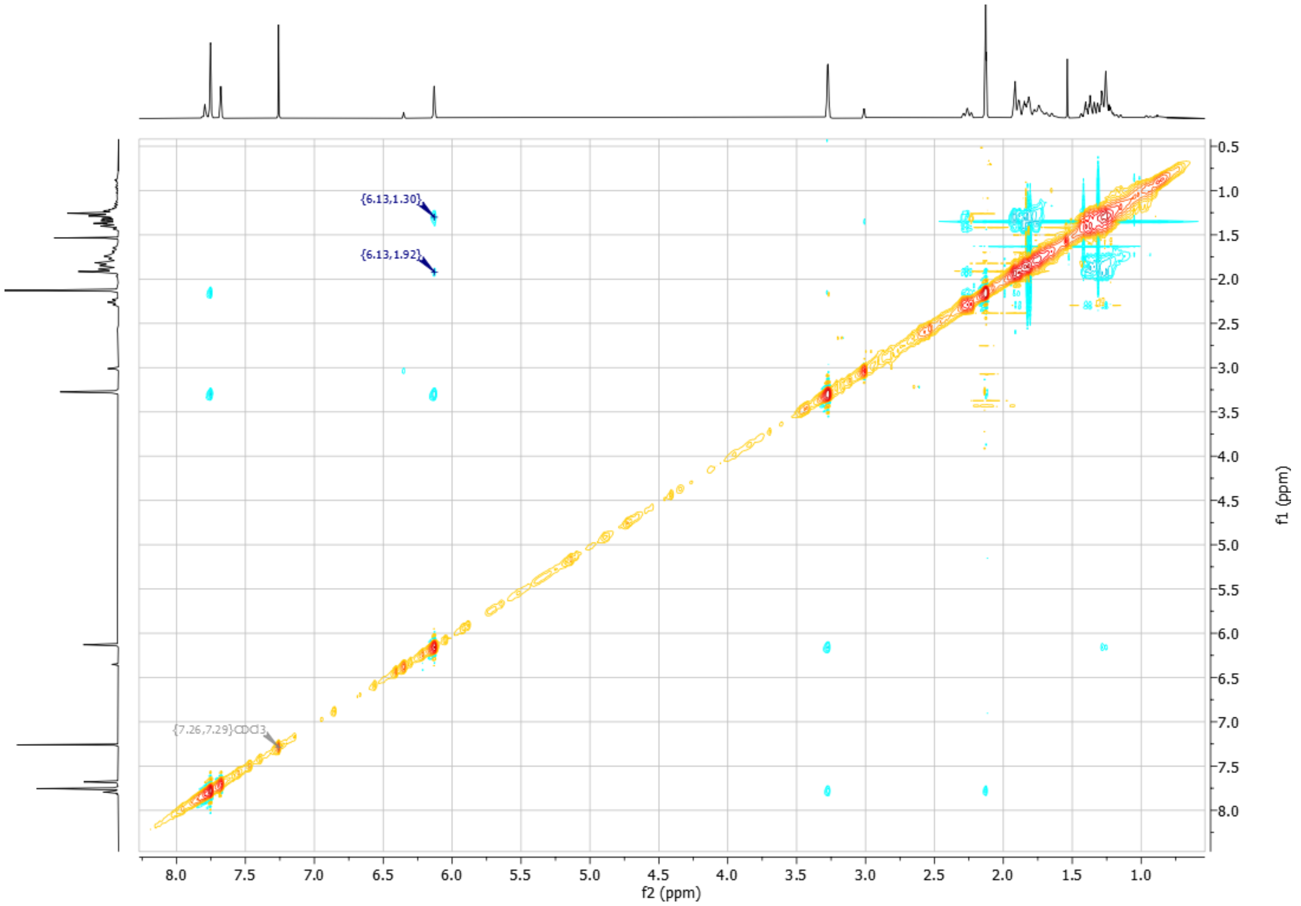

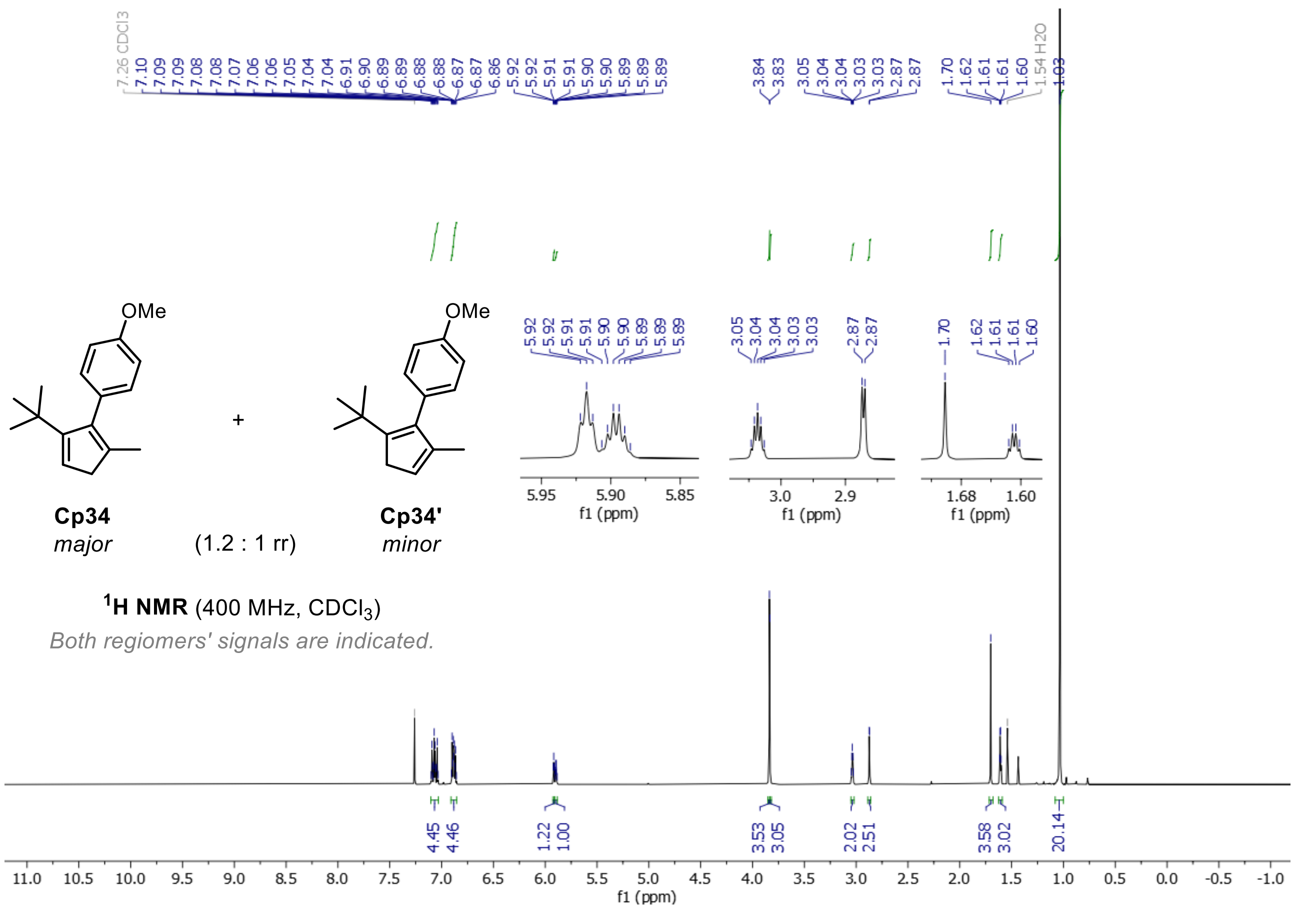

Cp34 *major* (1.2 : 1 rr) **Cp34'** *minor*

^{1}H NMR (400 MHz, $CDCl_3$)

Both regiomers' signals are indicated.

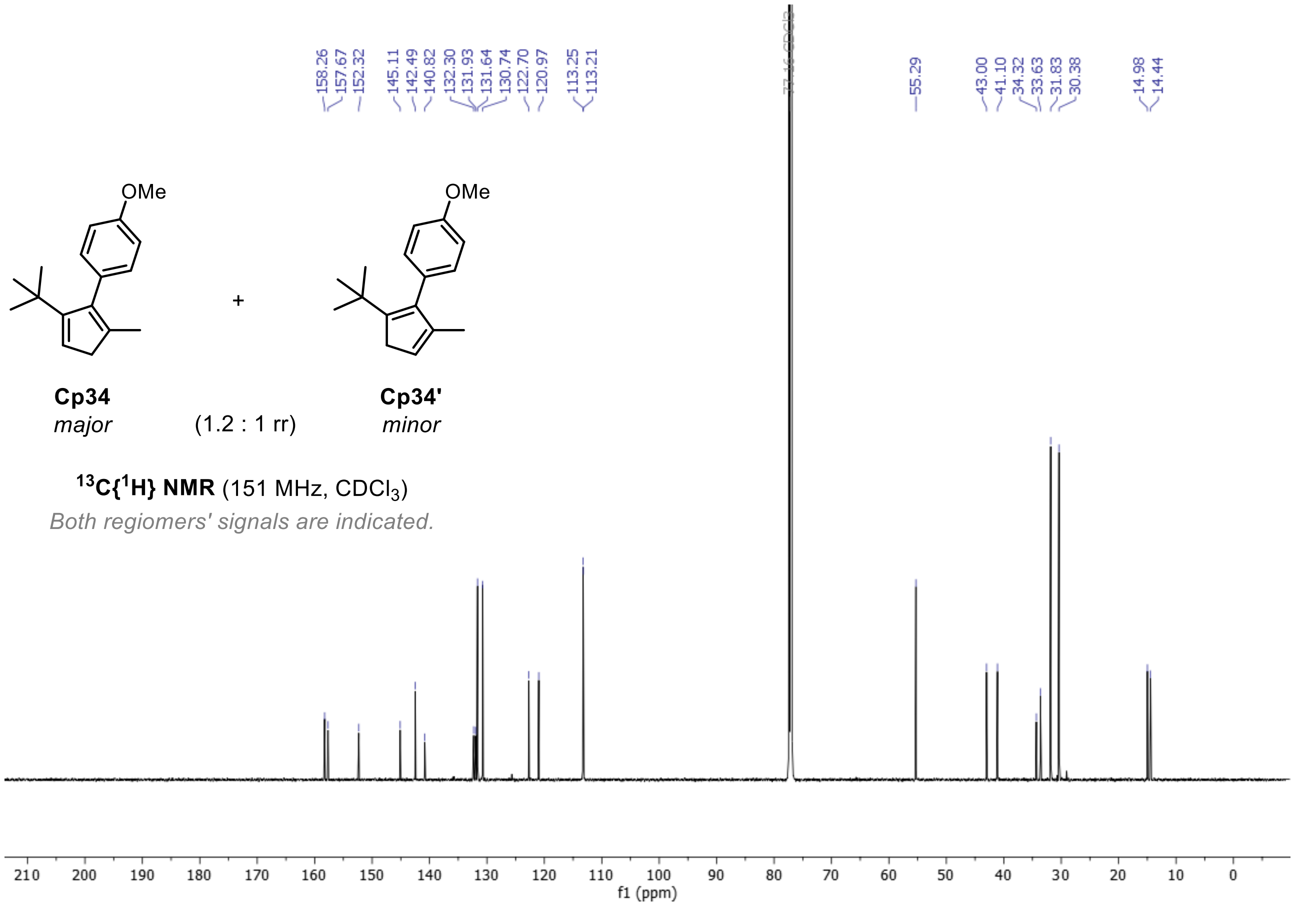

Cp34 *major* (1.2 : 1 rr) **Cp34'** *minor*

^{13}C{^{1}H} NMR (151 MHz, $CDCl_3$)

Both regiomers' signals are indicated.

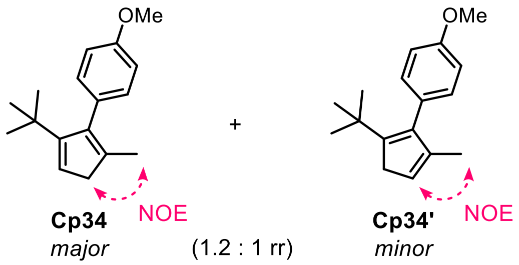

2D NOESY (400 MHz, $CDCl_3$)

Key interactions of both regiomers are indicated.

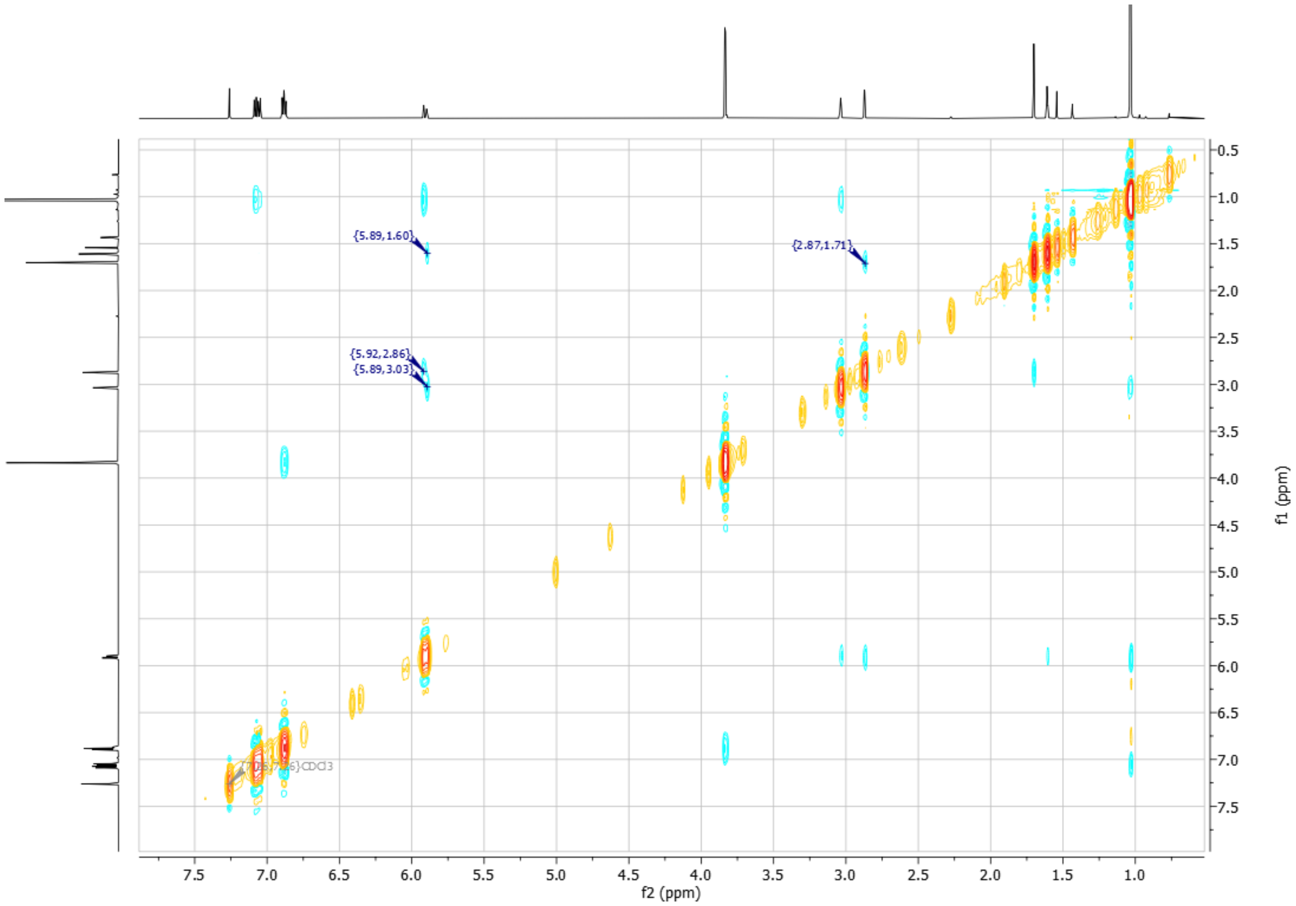

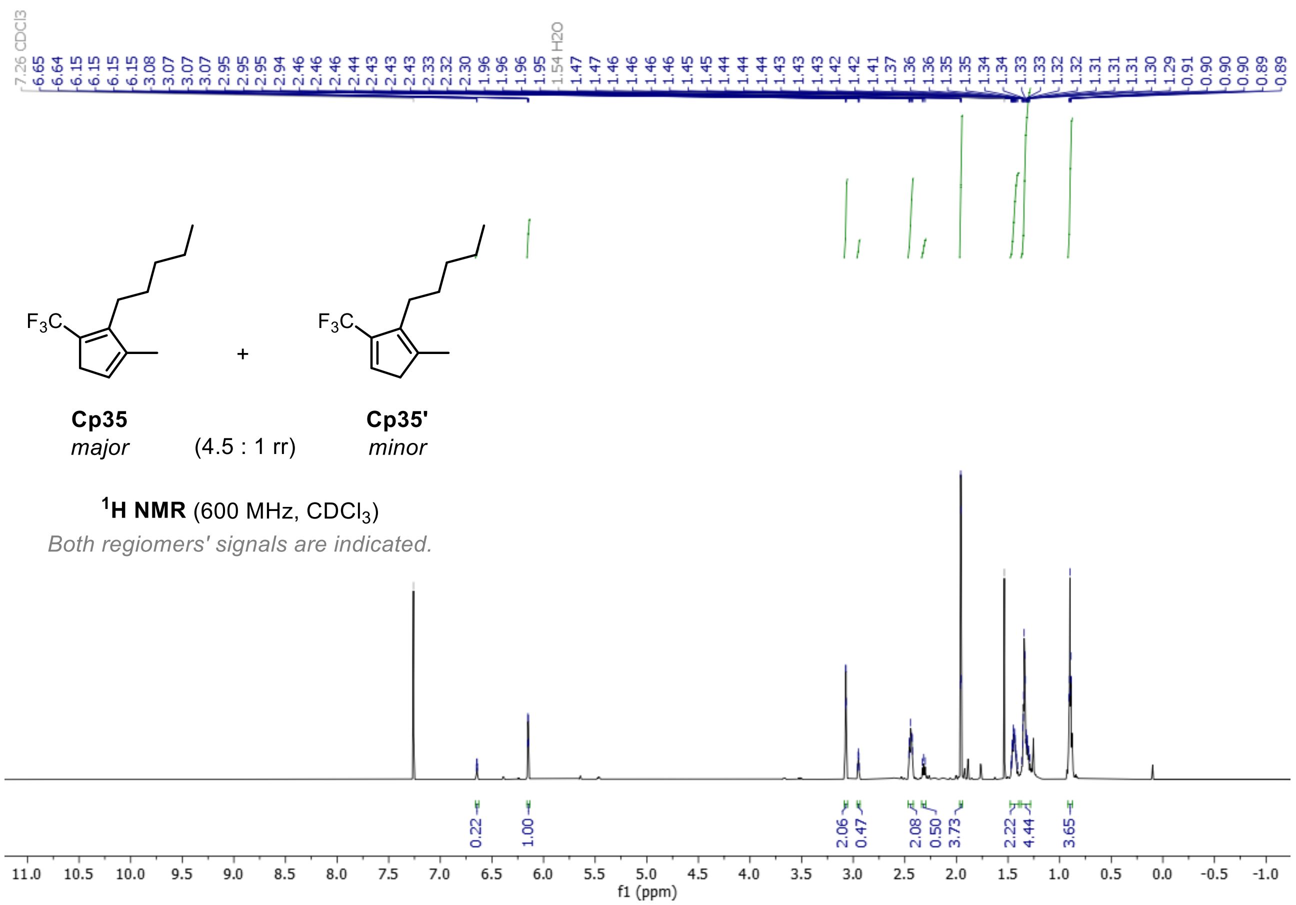
7.26 CDCl3
6.65 6.64 6.15 6.15 6.15 6.15 3.08 3.07 3.07 3.07 2.95 2.95 2.95 2.94 2.46 2.46 2.46 2.44 2.43 2.43 2.43 2.33 2.32 2.30 1.96 1.96 1.96 1.95
1.54 H2O
1.47 1.47 1.46 1.46 1.46 1.45 1.45 1.44 1.44 1.44 1.43 1.43 1.43 1.42 1.42 1.41 1.37 1.36 1.36 1.35 1.35 1.34 1.34 1.33 1.33 1.32 1.32 1.31 1.31 1.31 1.30 1.29 0.91 0.90 0.90 0.90 0.89 0.89
F_3C
+
Cp35
major
(4.5 : 1 rr)
Cp35'
minor
^{1}H NMR (600 MHz, $CDCl_3$)
Both regiomers' signals are indicated.
0.22 1.00 2.06 0.47 2.08 0.50 3.73 2.22 4.44 3.65
11.0 10.5 10.0 9.5 9.0 8.5 8.0 7.5 7.0 6.5 6.0 5.5 5.0 4.5 4.0 3.5 3.0 2.5 2.0 1.5 1.0 0.5 0.0 -0.5 -1.0
f1 (ppm)

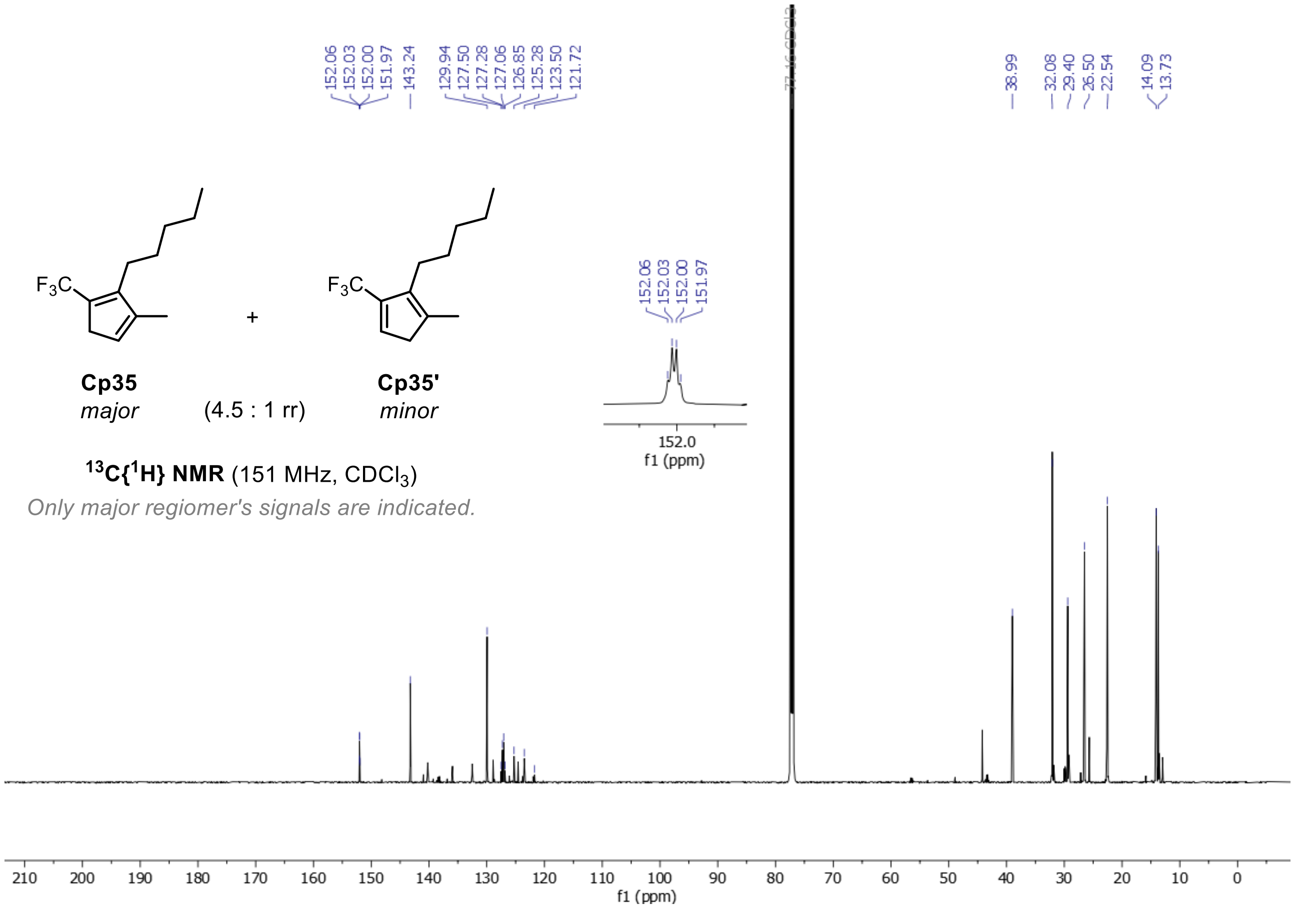
152.06 152.03 152.00 151.97 143.24 129.94 127.50 127.28 127.06 126.85 125.28 123.50 121.72
77.16 CDCl3
38.99 32.08 29.40 26.50 22.54 14.09 13.73
F_3C
+
Cp35
major
(4.5 : 1 rr)
Cp35'
minor
^{13}C{^{1}H} NMR (151 MHz, $CDCl_3$)
Only major regiomer's signals are indicated.
152.06 152.03 152.00 151.97
152.0
f1 (ppm)
210 200 190 180 170 160 150 140 130 120 110 100 90 80 70 60 50 40 30 20 10 0
f1 (ppm)

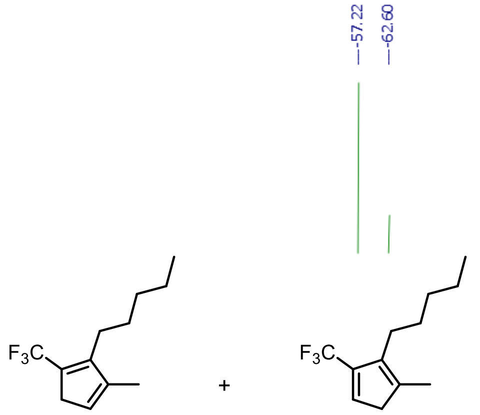

Cp35 *major* (4.5 : 1 rr) **Cp35'** *minor*

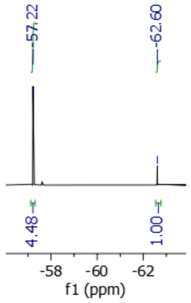

^{19}F{^{1}H} NMR (376 MHz, $CDCl_3$)

Both regiomers' signals are indicated.

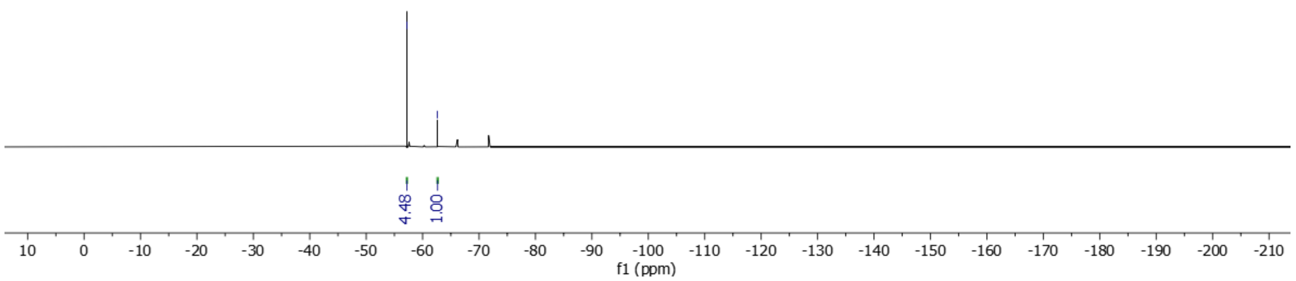

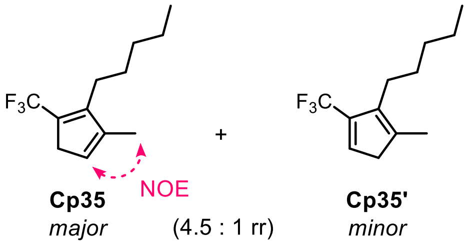

2D NOESY (400 MHz, $CDCl_3$)

Key interaction of the major regiomer is indicated.

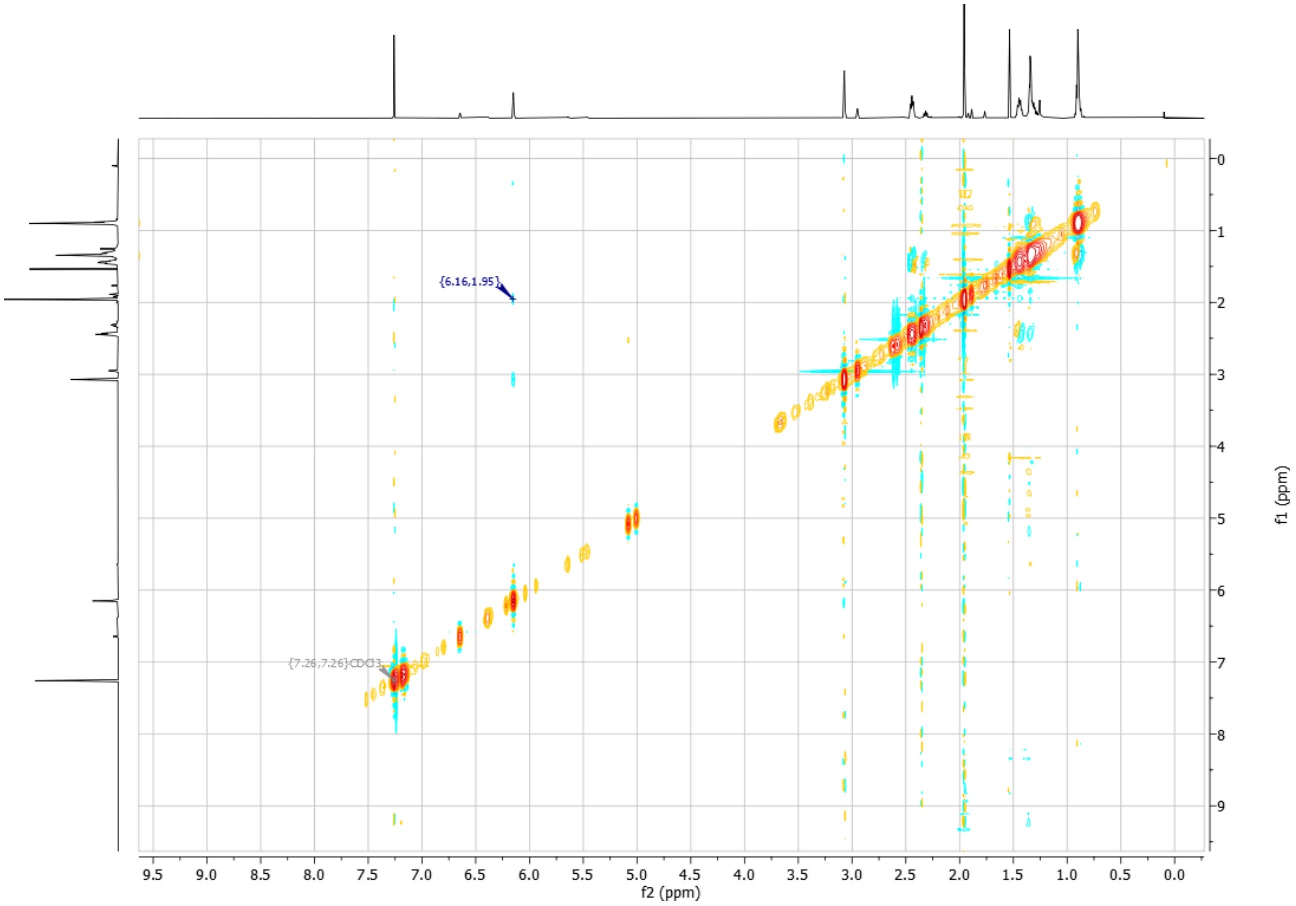

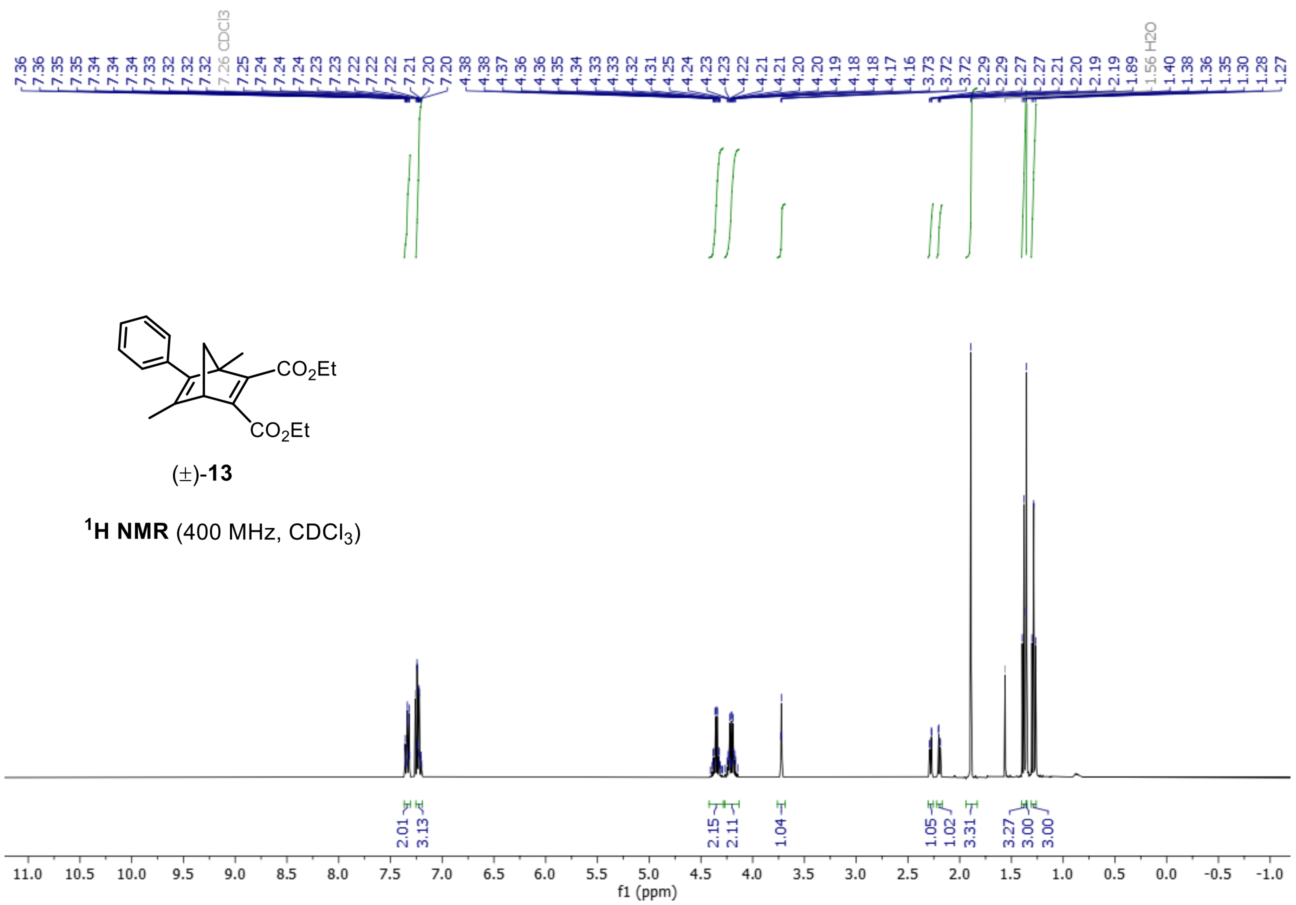
7.36 7.36 7.35 7.35 7.34 7.34 7.34 7.33 7.32 7.32 7.32 7.26 CDCl3 7.25 7.24 7.24 7.24 7.23 7.23 7.22 7.22 7.22 7.21 7.20 7.20 4.38 4.38 4.37 4.36 4.36 4.35 4.34 4.33 4.33 4.32 4.31 4.25 4.24 4.23 4.23 4.22 4.21 4.21 4.20 4.20 4.19 4.18 4.18 4.17 4.16 3.73 3.72 3.72 2.29 2.29 2.27 2.27 2.21 2.20 2.19 2.19 1.89 1.56 H2O 1.40 1.38 1.36 1.35 1.30 1.28 1.27
CO2Et
CO2Et
(±)-13
1H NMR (400 MHz, CDCl3)
2.01
3.13
2.15
2.11
1.04
1.05
1.02
3.31
3.27
3.00
3.00
11.0 10.5 10.0 9.5 9.0 8.5 8.0 7.5 7.0 6.5 6.0 5.5 5.0 4.5 4.0 3.5 3.0 2.5 2.0 1.5 1.0 0.5 0.0 -0.5 -1.0
f1 (ppm)

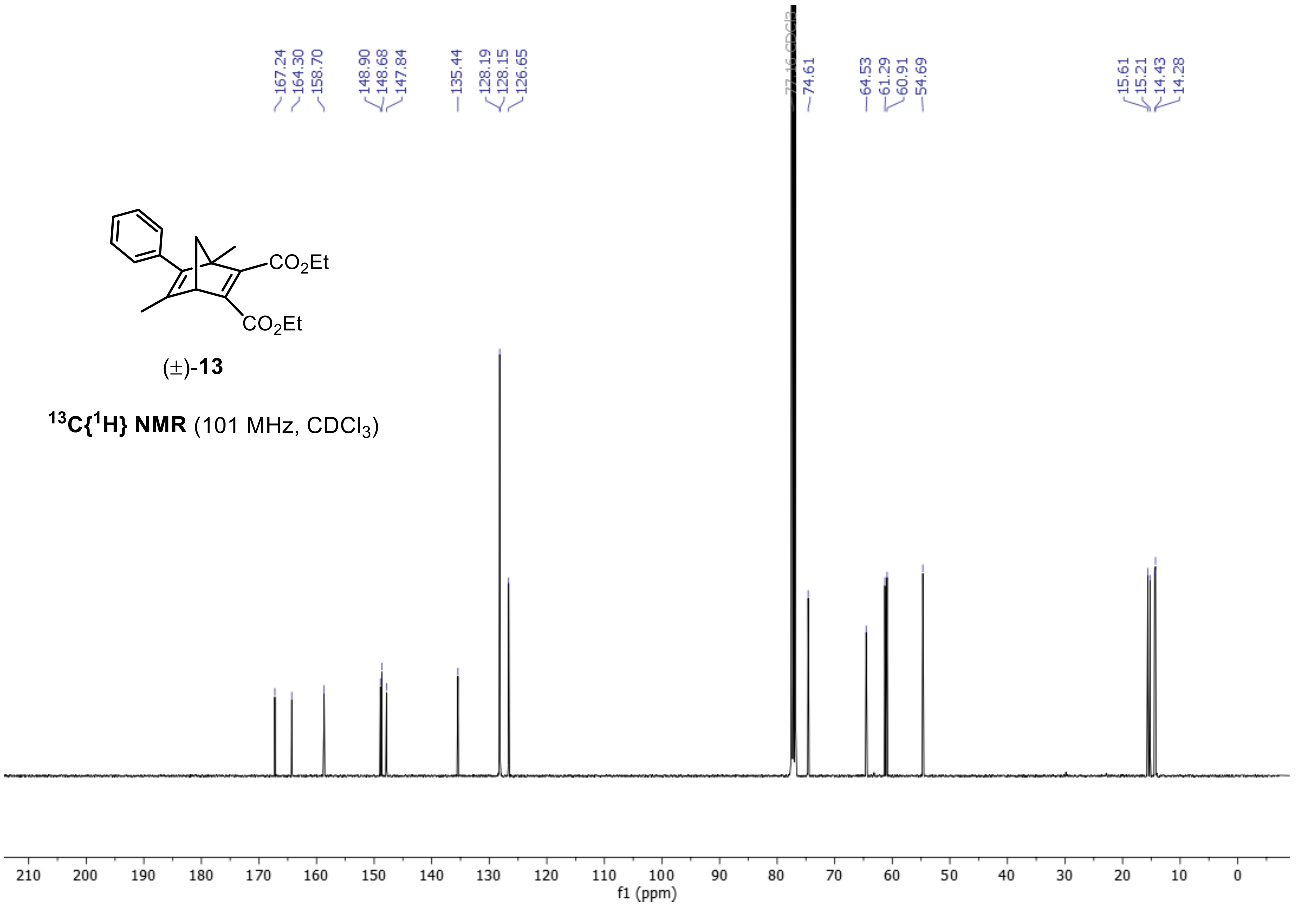
167.24 164.30 158.70 148.90 148.68 147.84 135.44 128.19 128.15 126.65 77.16 CDCl3 74.61 64.53 61.29 60.91 54.69 15.61 15.21 14.43 14.28
CO2Et
CO2Et
(±)-13
13C{1H} NMR (101 MHz, CDCl3)
210 200 190 180 170 160 150 140 130 120 110 100 90 80 70 60 50 40 30 20 10 0
f1 (ppm)

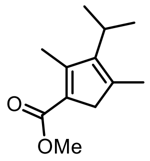

Cp36

^{1}H NMR (400 MHz, $CDCl_3$)

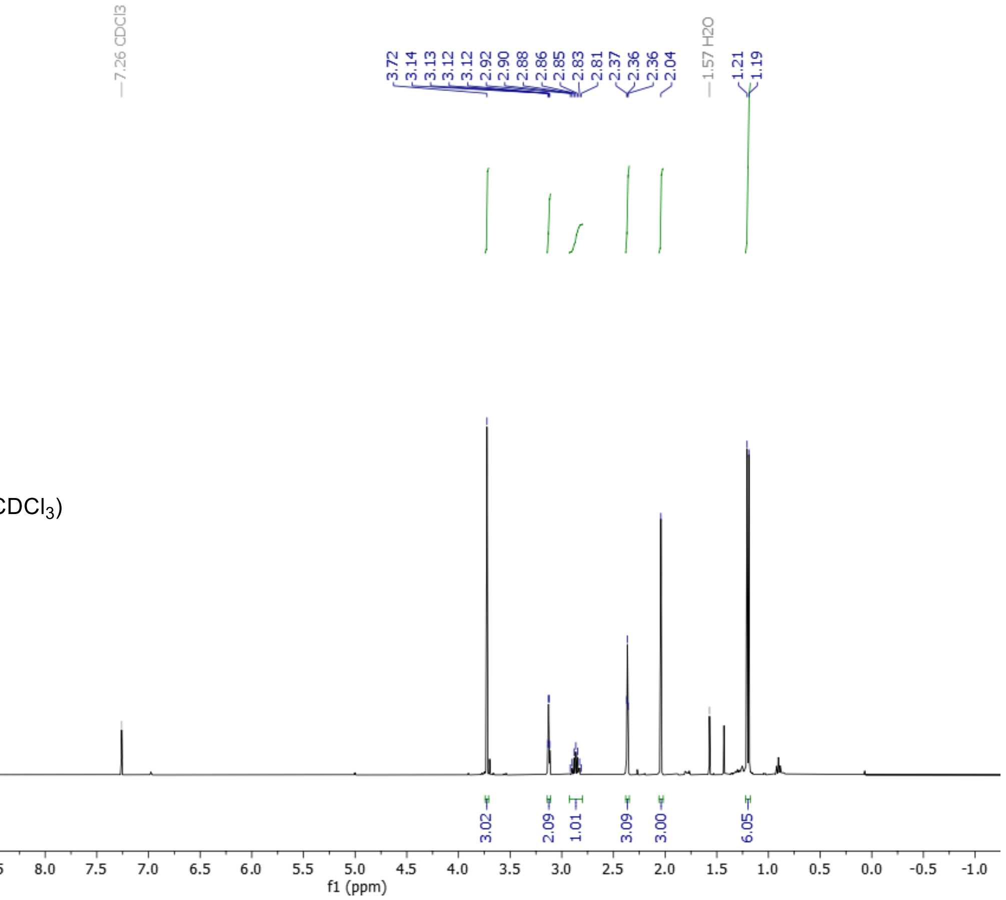

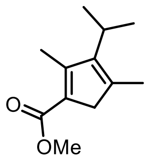

Cp36

^{13}C{^{1}H} NMR (101 MHz, $CDCl_3$)

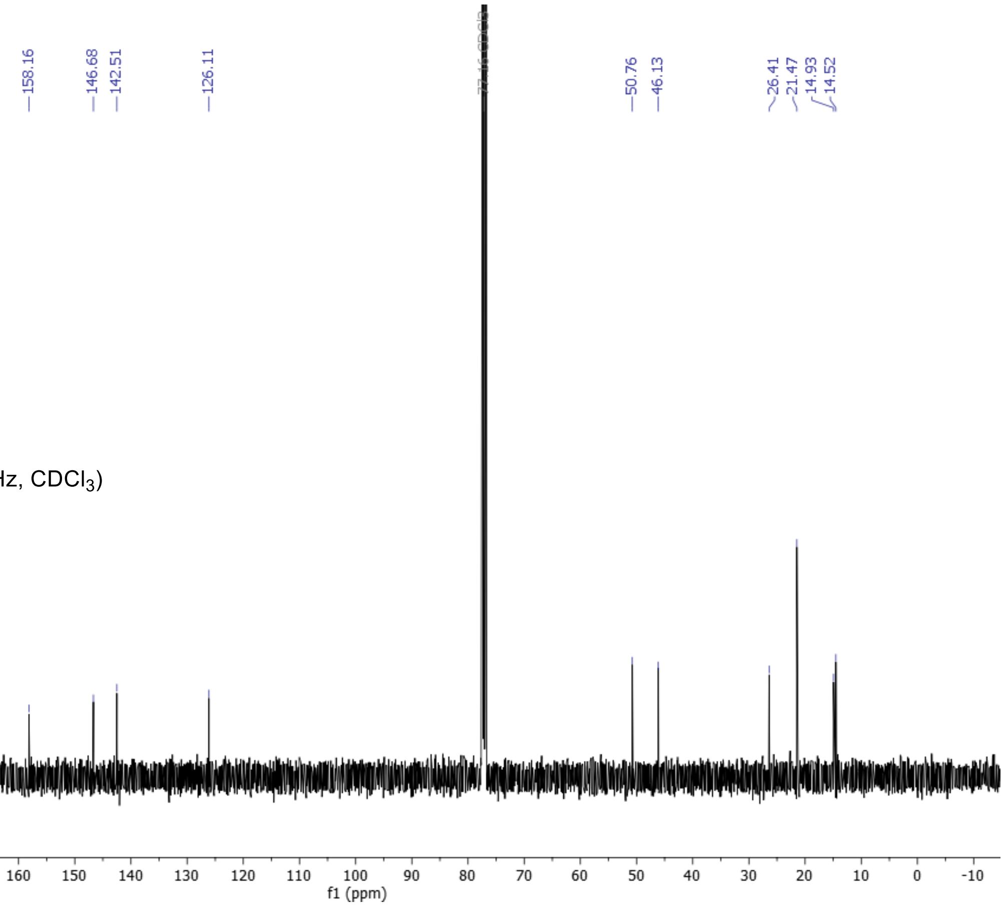

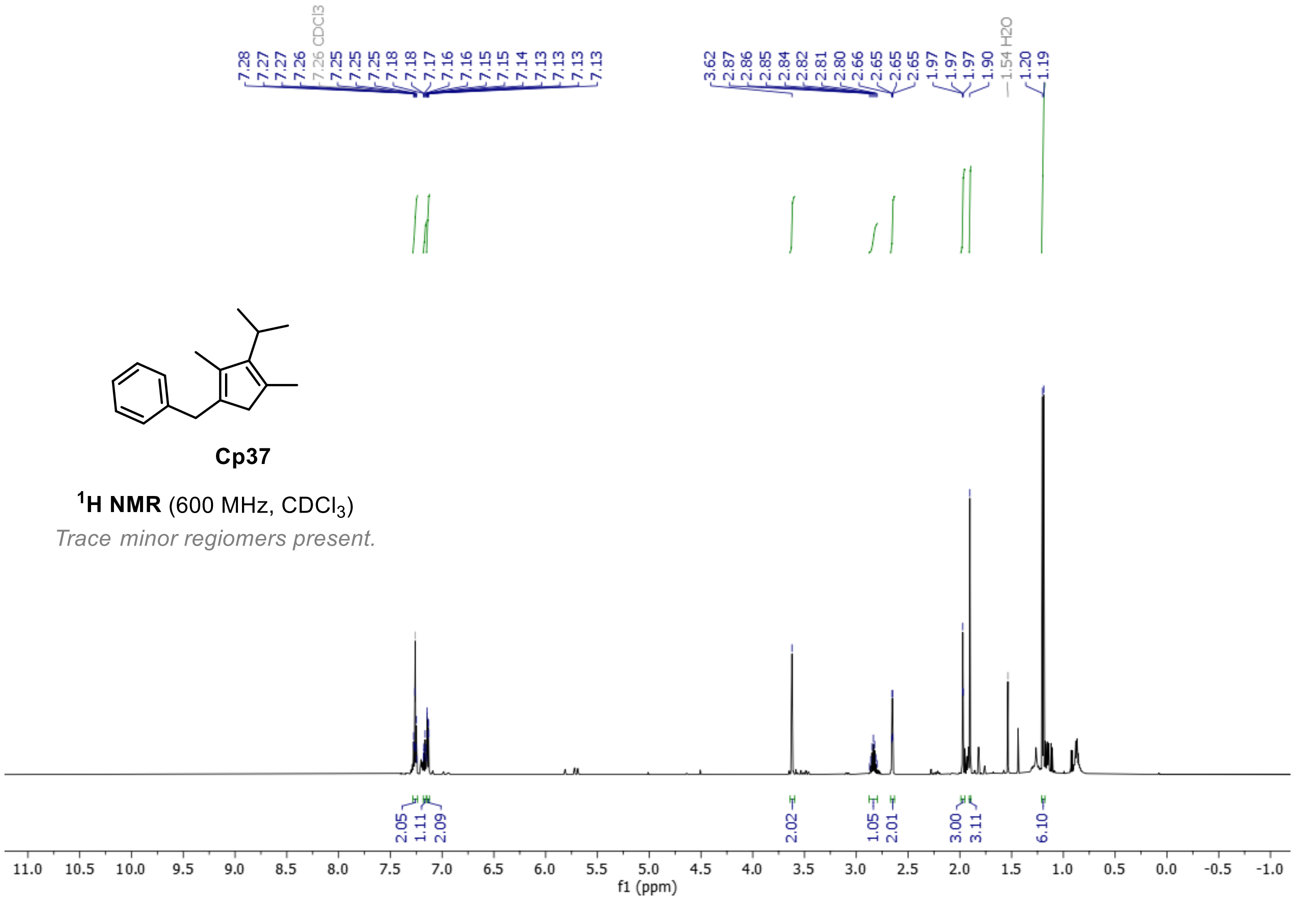

Cp37

^{1}H NMR (600 MHz, $CDCl_3$)

Trace minor regiomers present.

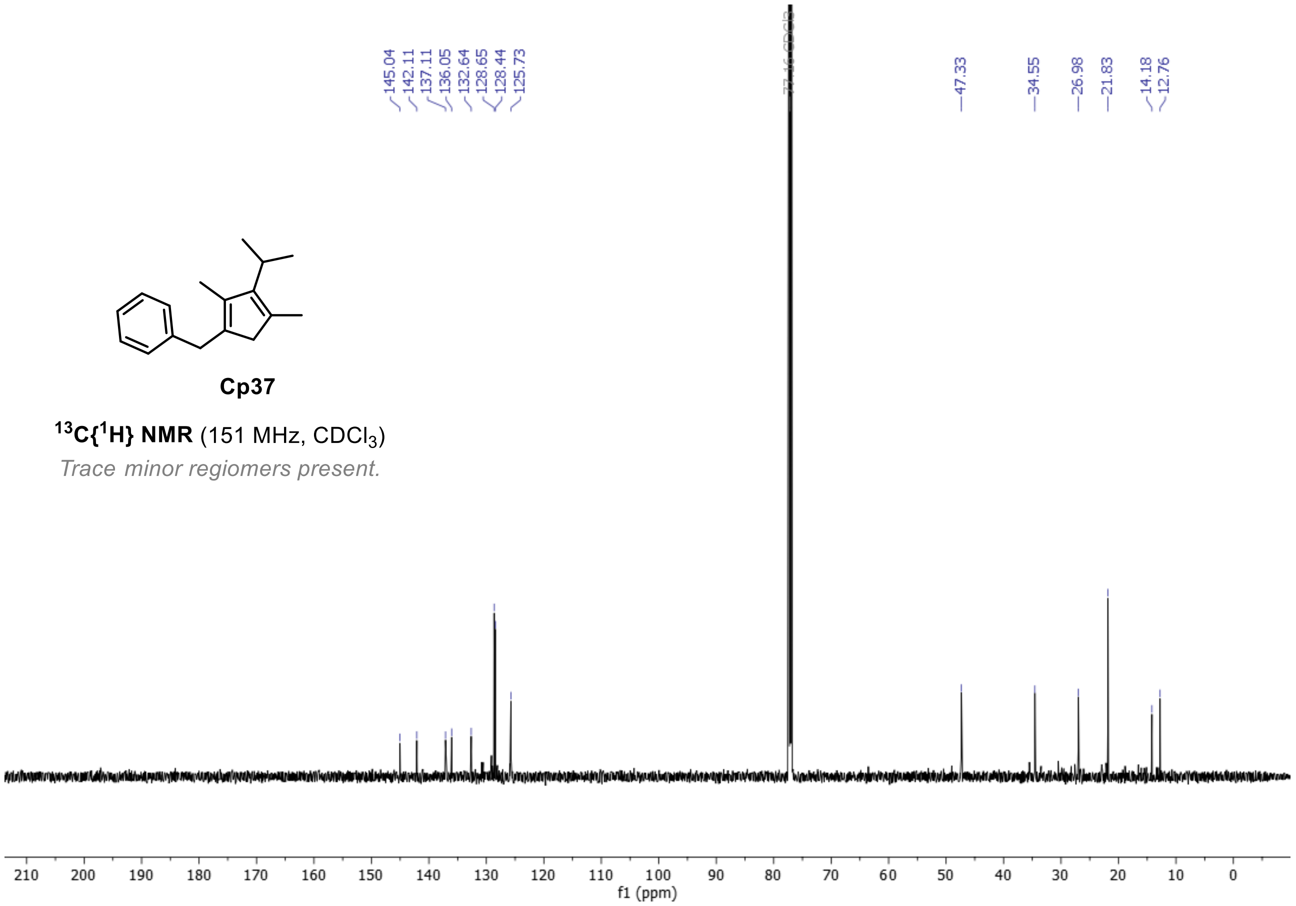

Cp37

^{13}C{^{1}H} NMR (151 MHz, $CDCl_3$)

Trace minor regiomers present.

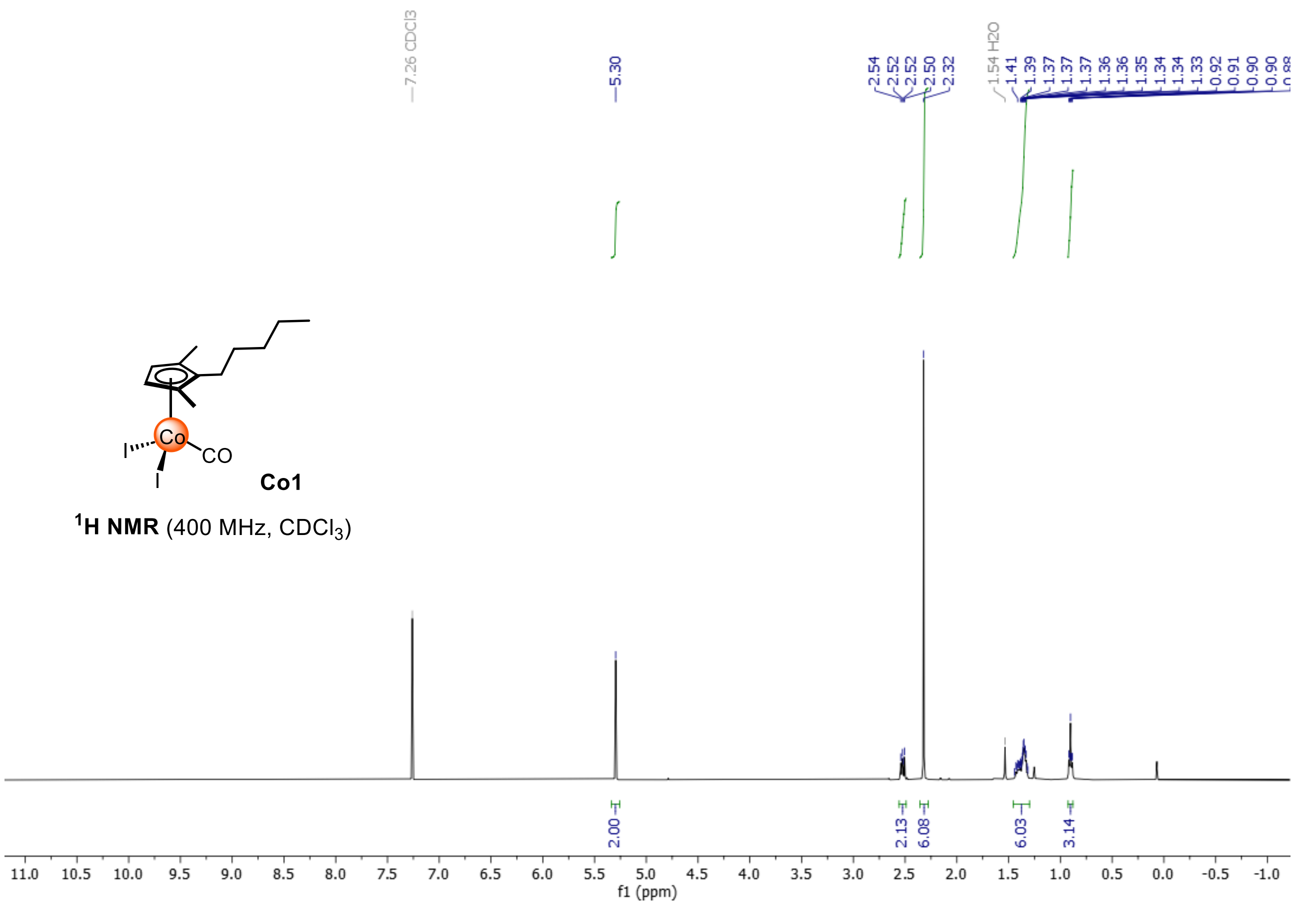
7.26 CDCl3
5.30
2.54
2.52
2.52
2.50
2.32
1.54 H2O
1.41
1.39
1.37
1.37
1.37
1.36
1.36
1.35
1.34
1.34
1.33
0.92
0.91
0.90
0.90
Co
CO
I
I
Co1
1H NMR (400 MHz, CDCl3)
2.00
2.13
6.08
6.03
3.14
f1 (ppm)

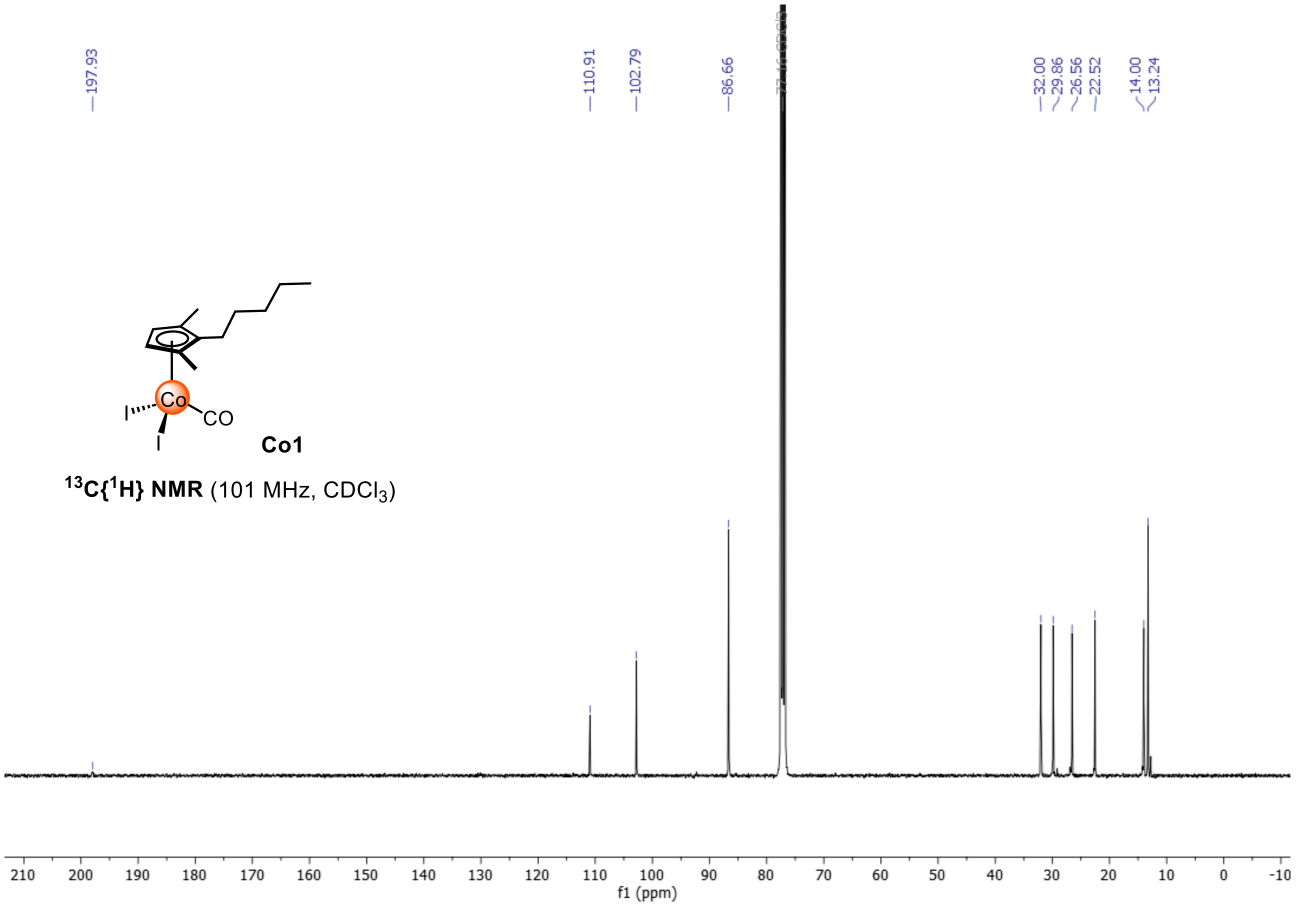
197.93
110.91
102.79
86.66
32.00
29.86
26.56
22.52
14.00
13.24
Co
CO
I
I
Co1
13C{1H} NMR (101 MHz, CDCl3)
f1 (ppm)

Co2

^{1}**H NMR** (400 MHz, $CDCl_3$)

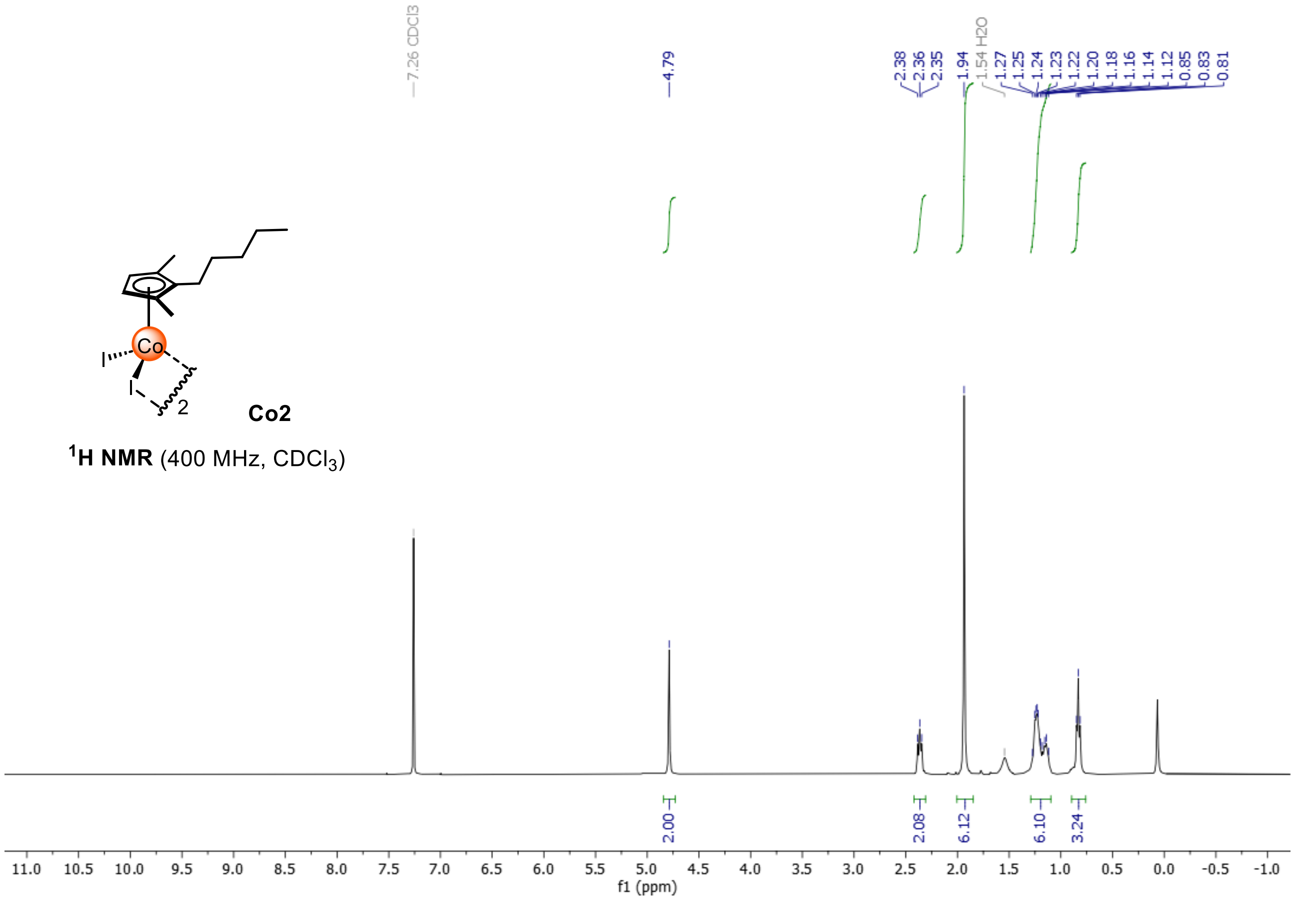

Co2

^{13}**C{^{1}H} NMR** (101 MHz, $CDCl_3$)

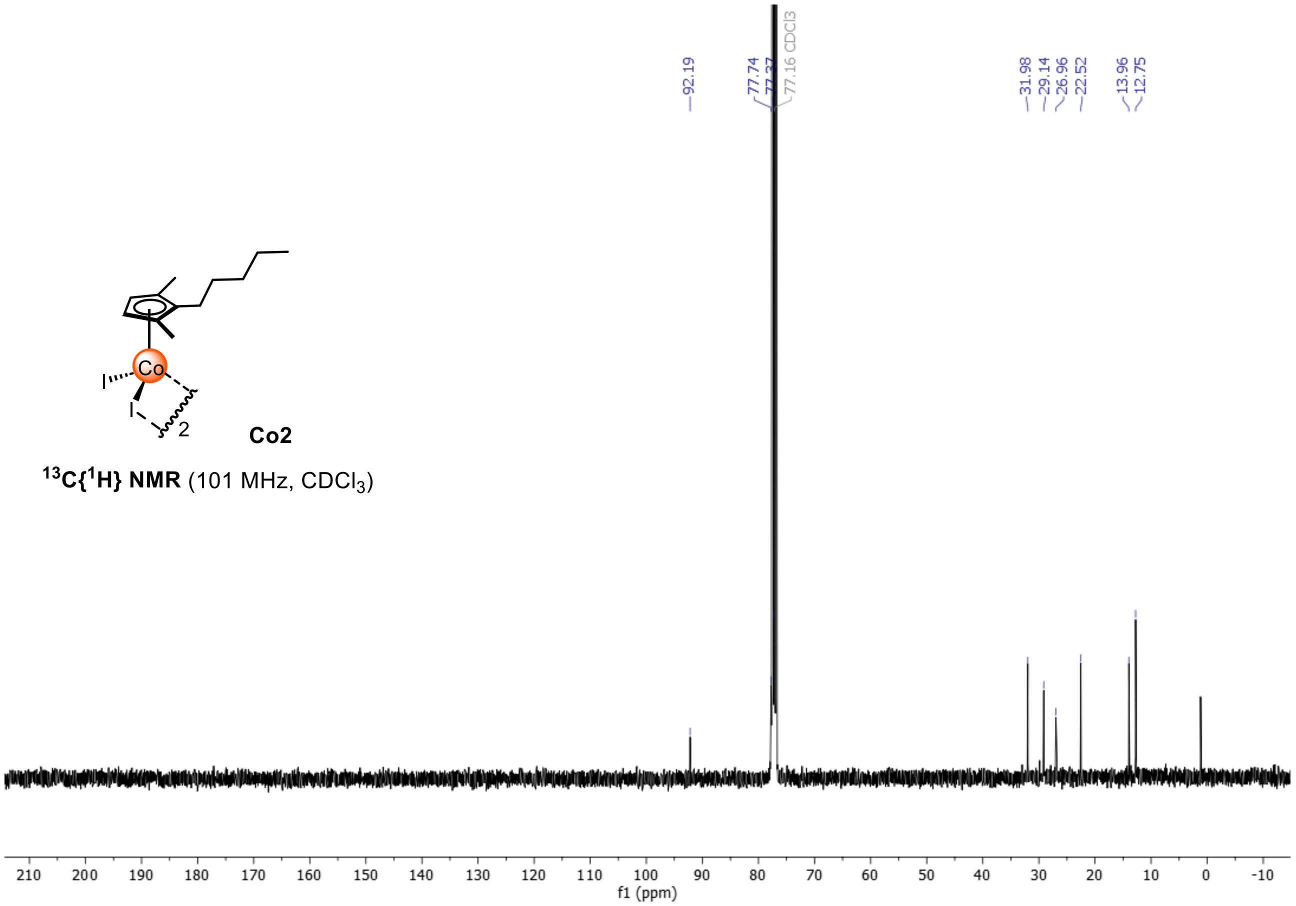

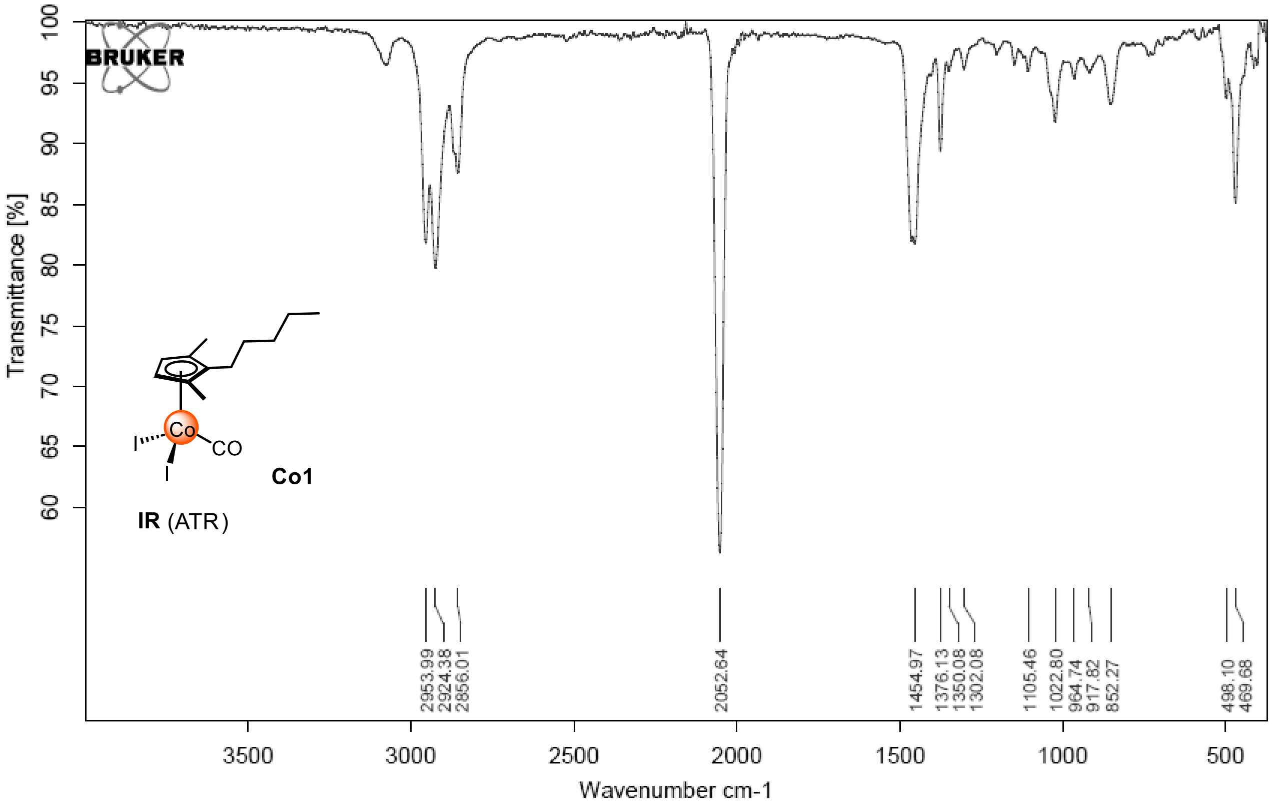
Transmittance [%]
Wavenumber cm-1
2953.99
2924.38
2856.01
2052.64
1454.97
1376.13
1350.08
1302.08
1105.46
1022.80
964.74
917.82
852.27
498.10
469.68
Co
CO
Co1
IR (ATR)

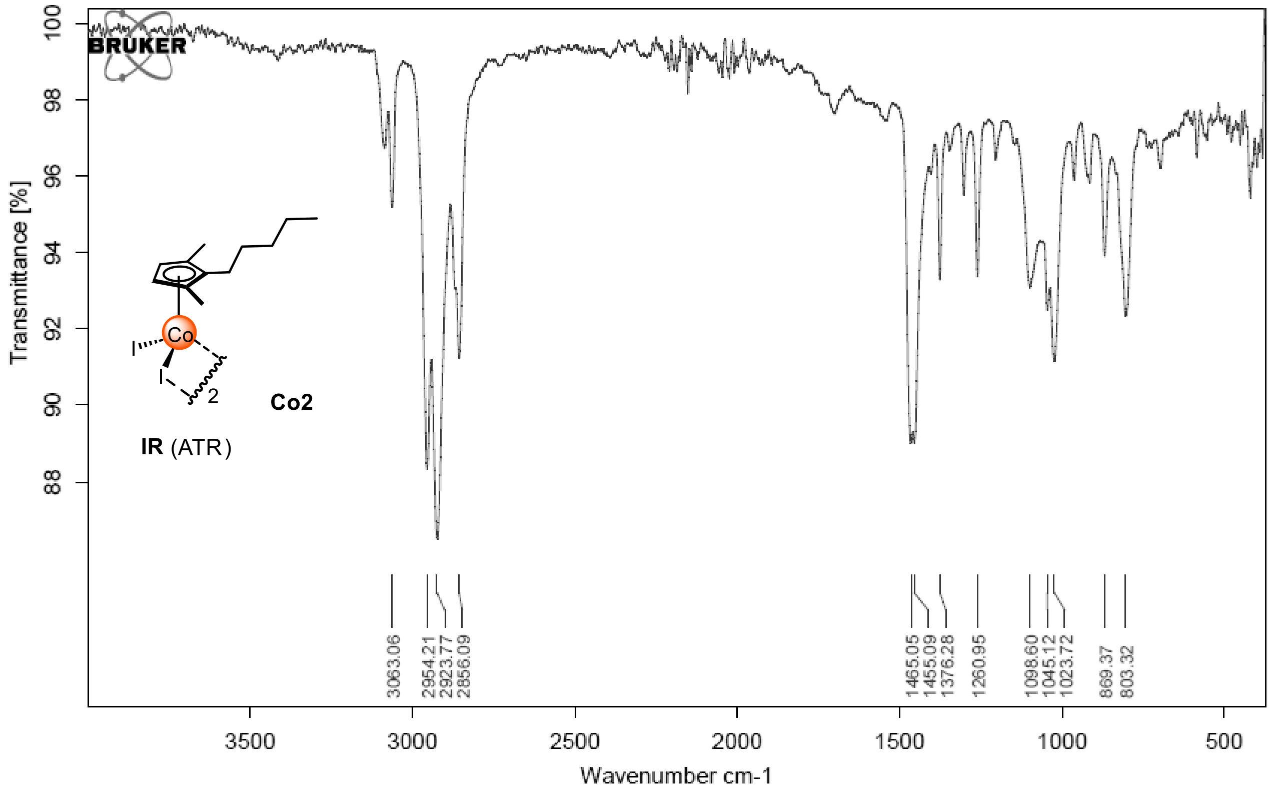
Transmittance [%]
Wavenumber cm-1
3063.06
2954.21
2923.77
2856.09
1465.05
1455.09
1376.28
1260.95
1098.60
1045.12
1023.72
869.37
803.32
Co
2
Co2
IR (ATR)

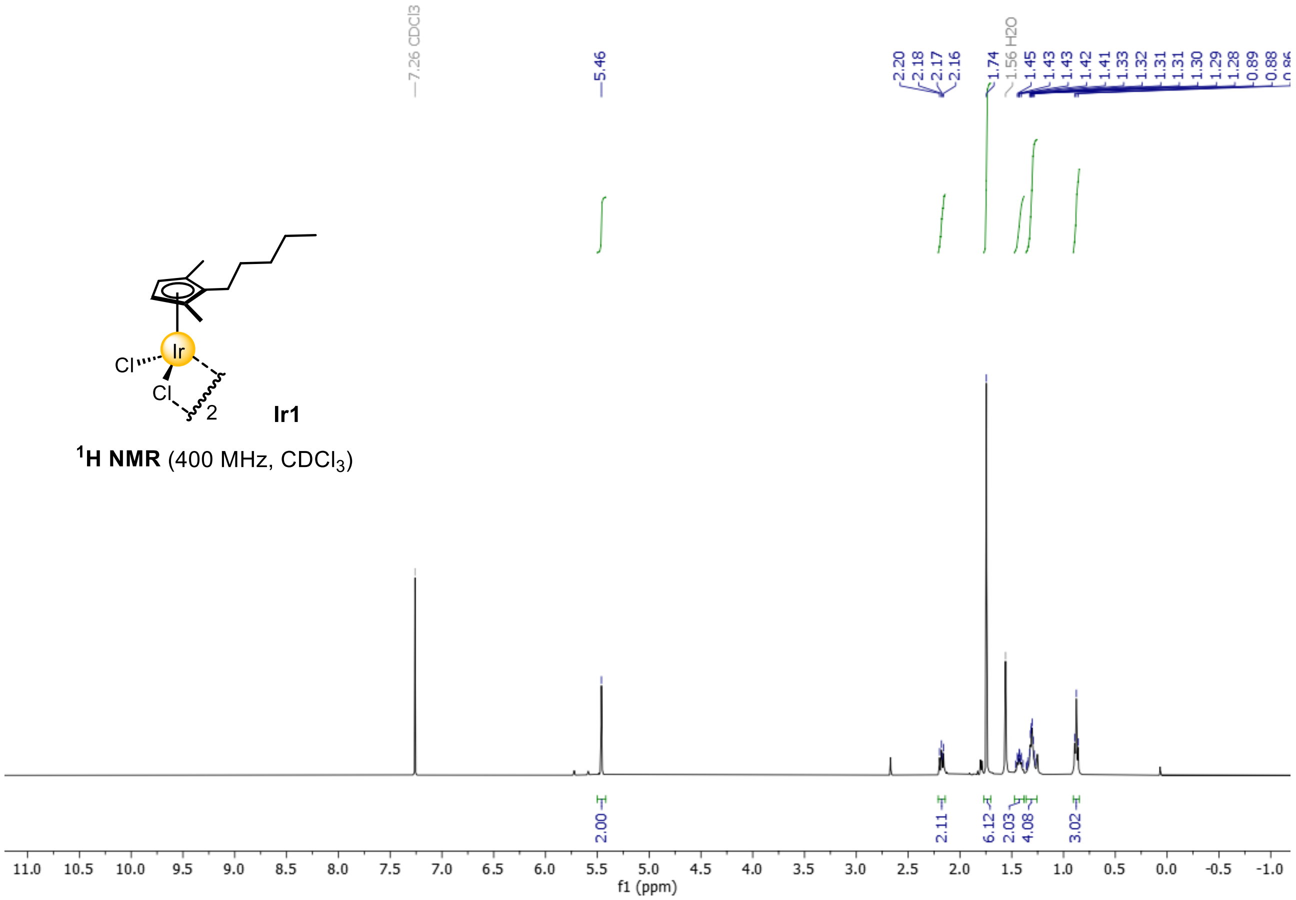

^{1}**H NMR** (400 MHz, $CDCl_3$)

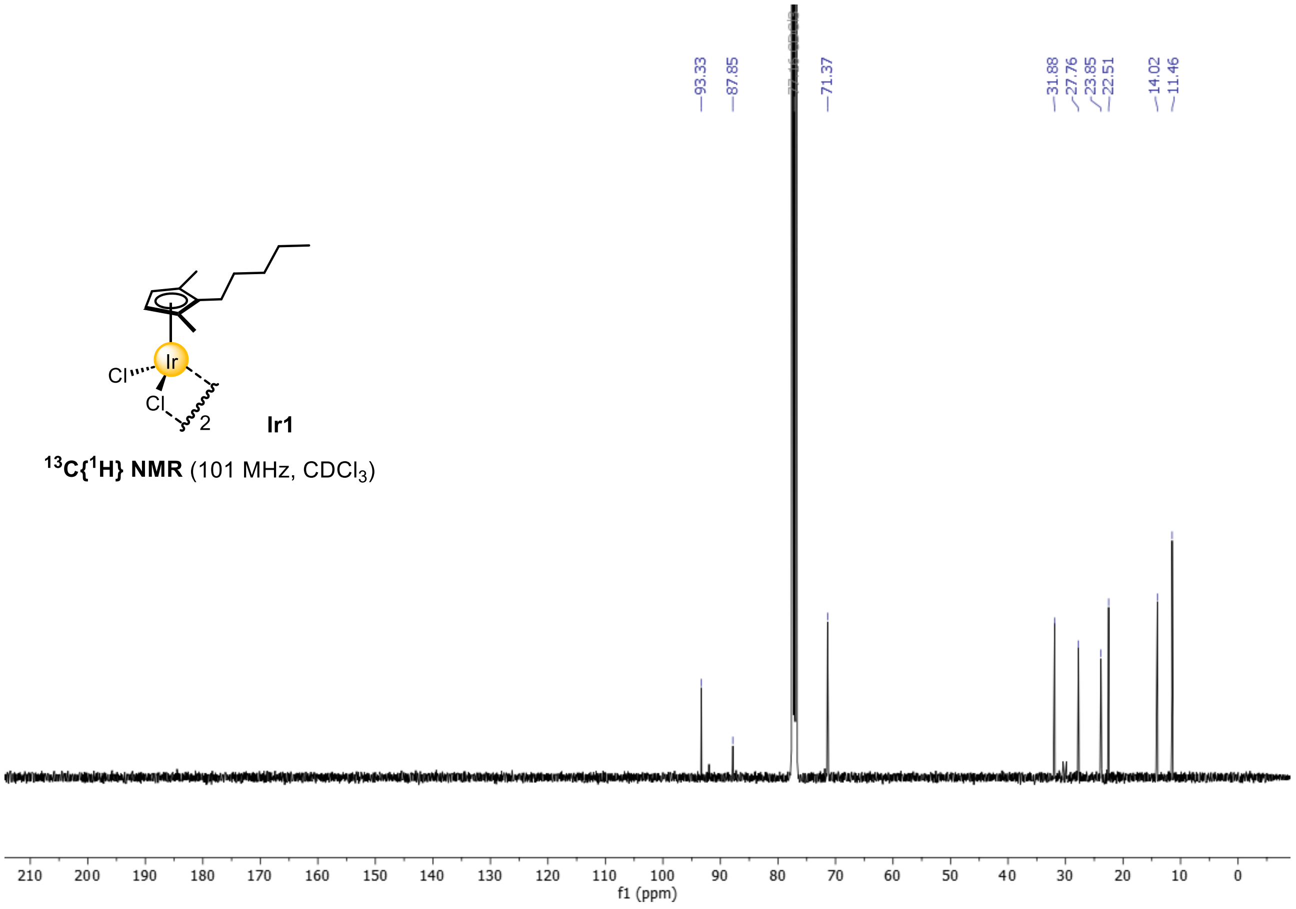

^{13}**C{^{1}H} NMR** (101 MHz, $CDCl_3$)

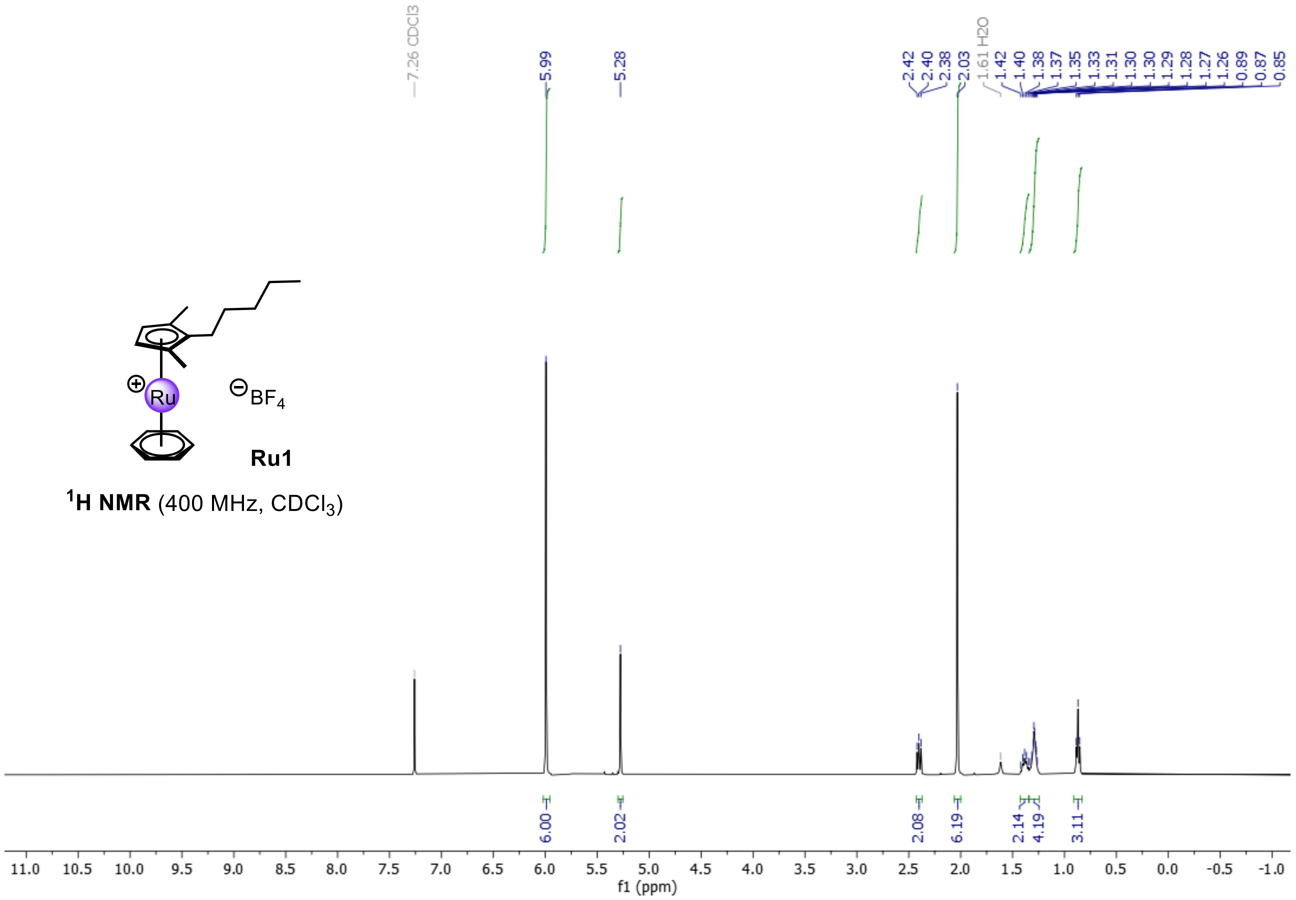
7.26 CDCl3
5.99
5.28
2.42
2.40
2.38
2.03
1.61 H2O
1.42
1.40
1.38
1.37
1.35
1.33
1.31
1.30
1.30
1.29
1.28
1.27
1.26
0.89
0.87
0.85
Ru
BF4
Ru1
¹H NMR (400 MHz, CDCl3)
6.00
2.02
2.08
6.19
2.14
4.19
3.11
f1 (ppm)

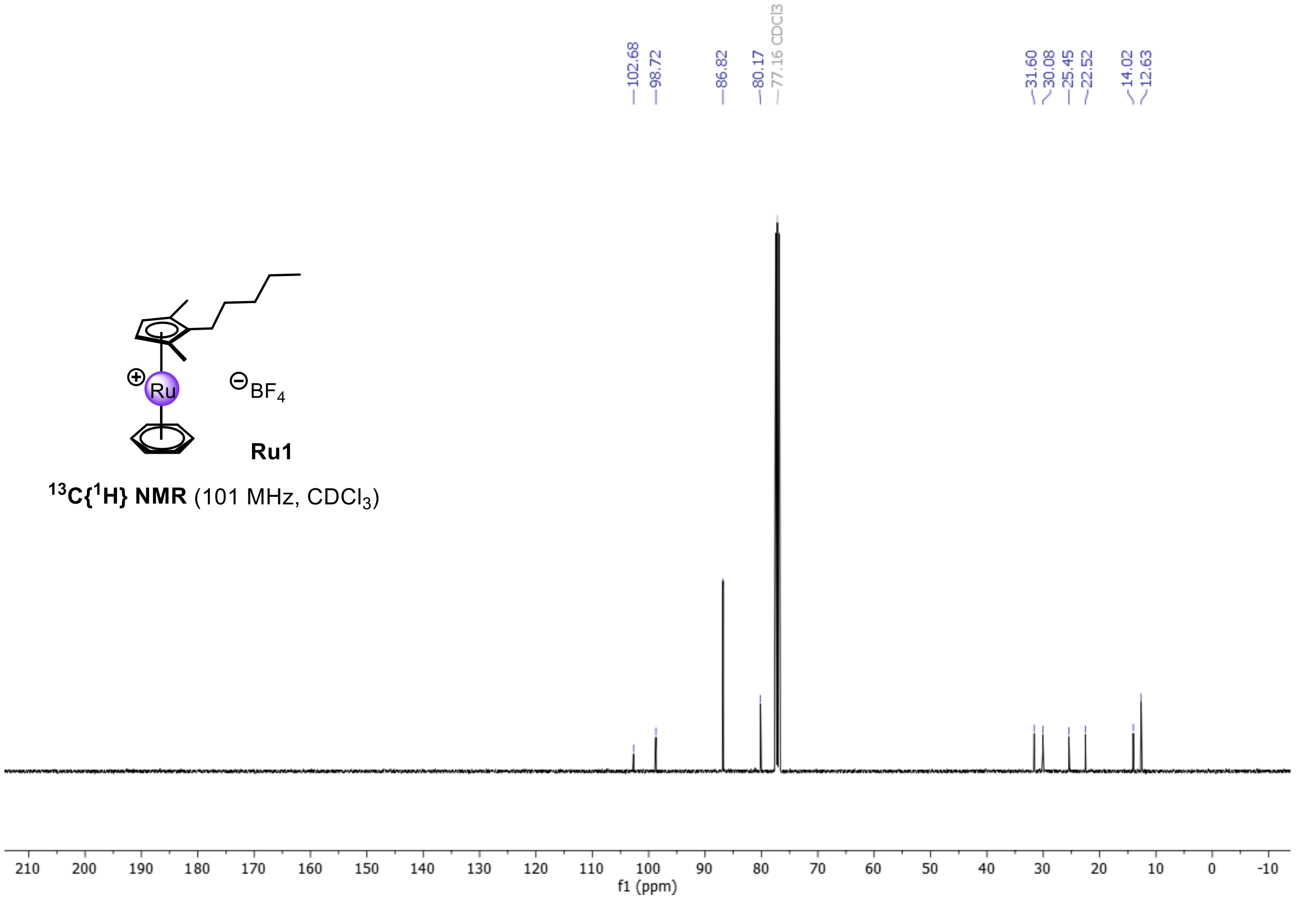
102.68
98.72
86.82
80.17
77.16 CDCl3
31.60
30.08
25.45
22.52
14.02
12.63
Ru
BF4
Ru1
¹³C{¹H} NMR (101 MHz, CDCl3)
f1 (ppm)

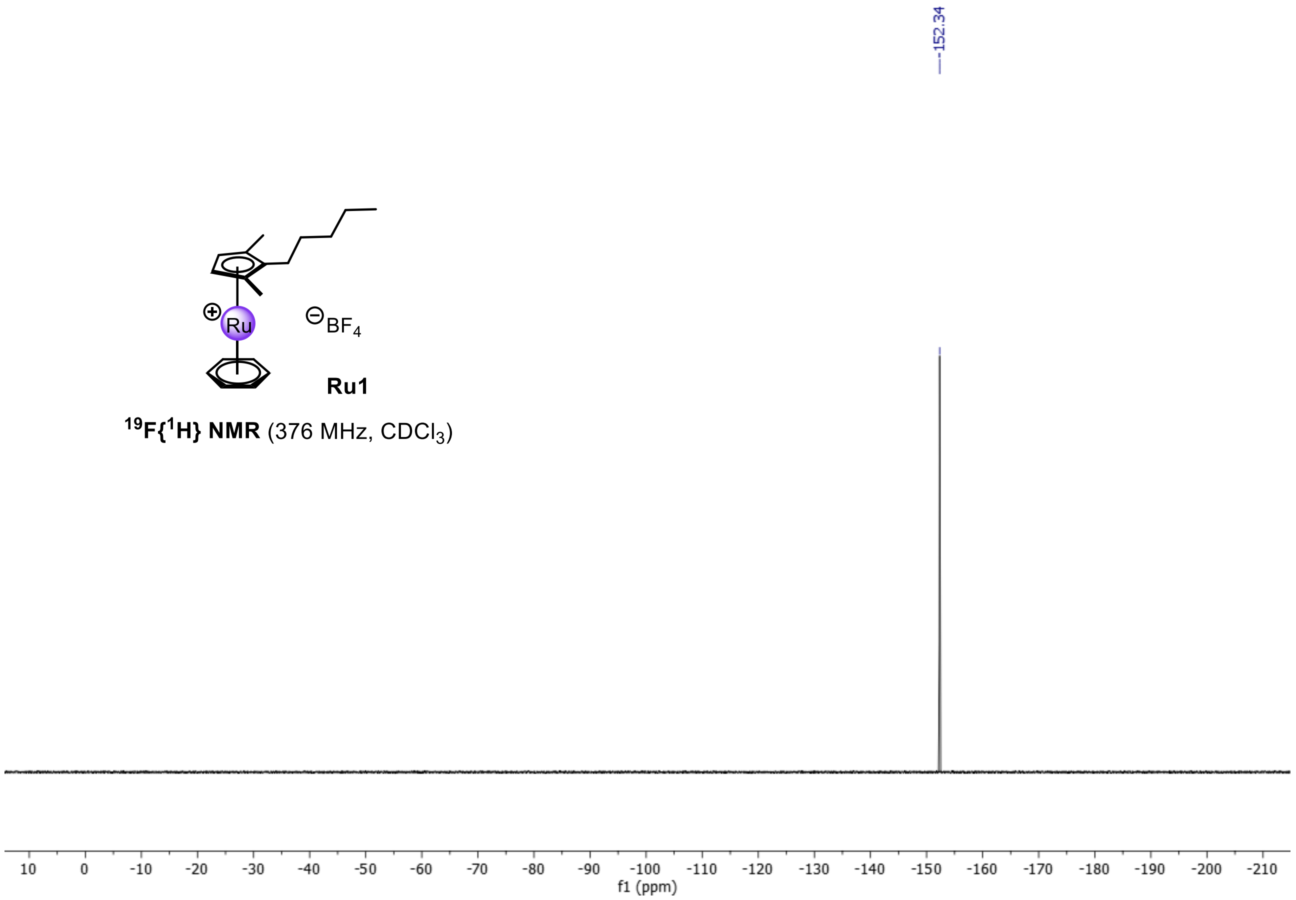
-152.34
Ru
BF4
Ru1
19F{1H} NMR (376 MHz, CDCl3)
10
0
-10
-20
-30
-40
-50
-60
-70
-80
-90
-100
-110
-120
-130
-140
-150
-160
-170
-180
-190
-200
-210
f1 (ppm)

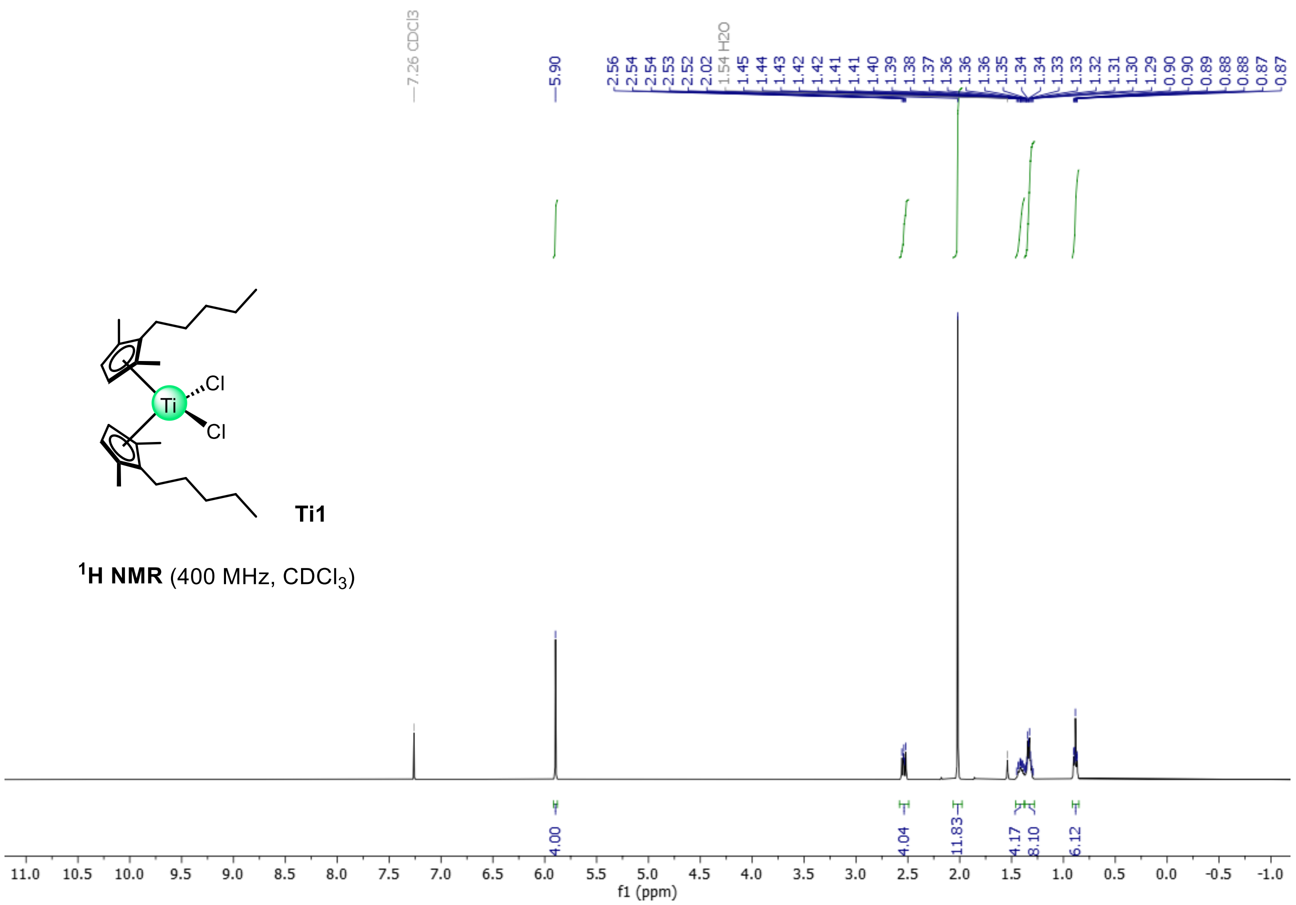
7.26 CDCl3
5.90
2.56
2.54
2.54
2.53
2.52
2.02
1.54 H2O
1.45
1.44
1.43
1.42
1.42
1.41
1.41
1.40
1.39
1.38
1.37
1.36
1.36
1.36
1.35
1.34
1.34
1.33
1.33
1.32
1.31
1.30
1.29
0.90
0.90
0.89
0.88
0.88
0.87
0.87
Cl
Ti
Cl
Ti1
1H NMR (400 MHz, CDCl3)
4.00
4.04
11.83
4.17
8.10
6.12
f1 (ppm)

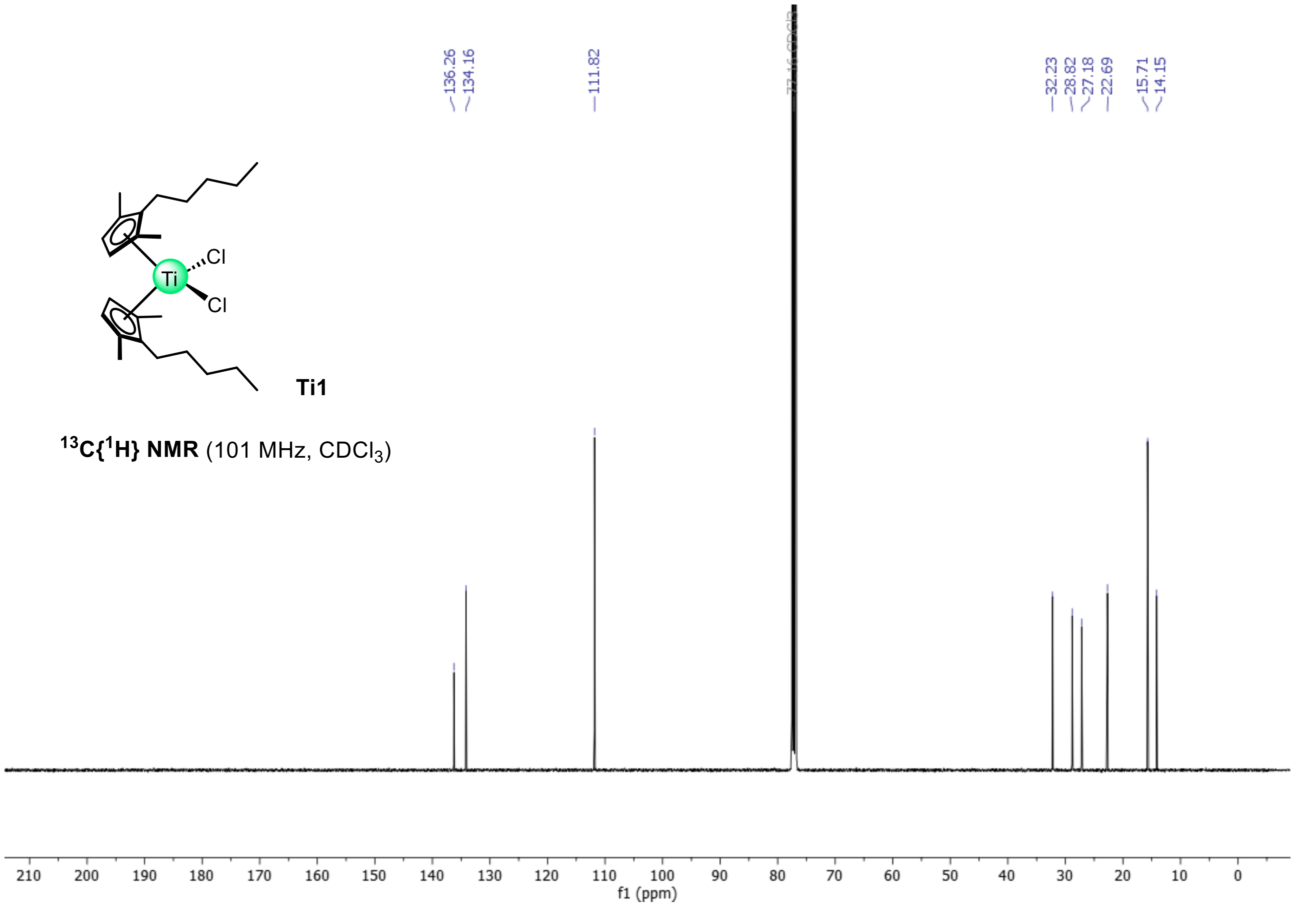
136.26
134.16
111.82
32.23
28.82
27.18
22.69
15.71
14.15
Cl
Ti
Cl
Ti1
13C{1H} NMR (101 MHz, CDCl3)
f1 (ppm)

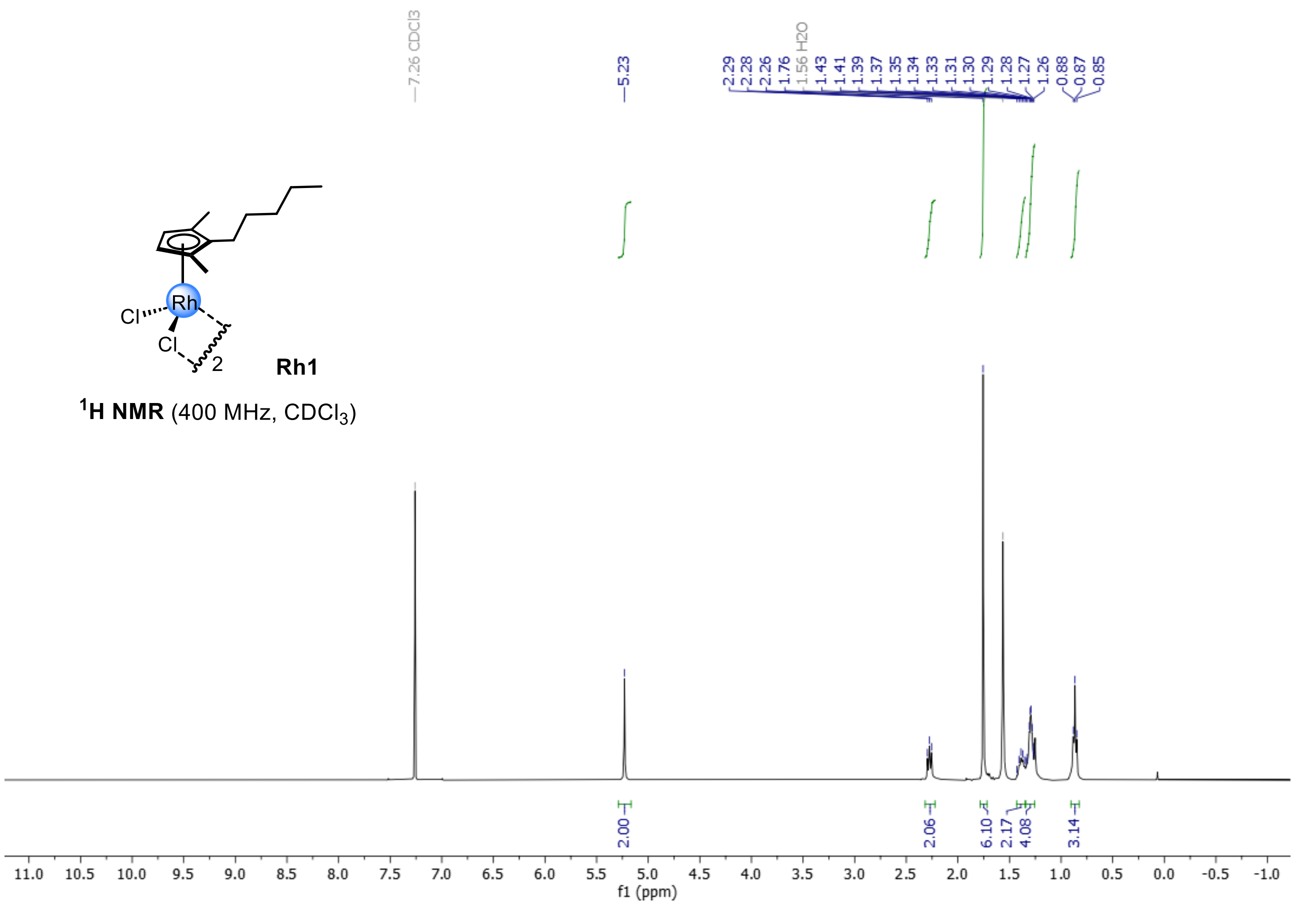
Rh
Cl
Cl
2
Rh1
^{1}H NMR (400 MHz, CDCl$_3$)
7.26 CDCl3
5.23
2.29
2.28
2.26
1.76
1.56 H2O
1.43
1.41
1.39
1.37
1.35
1.34
1.33
1.31
1.30
1.29
1.28
1.27
1.26
0.88
0.87
0.85
2.00
2.06
6.10
2.17
4.08
3.14
f1 (ppm)

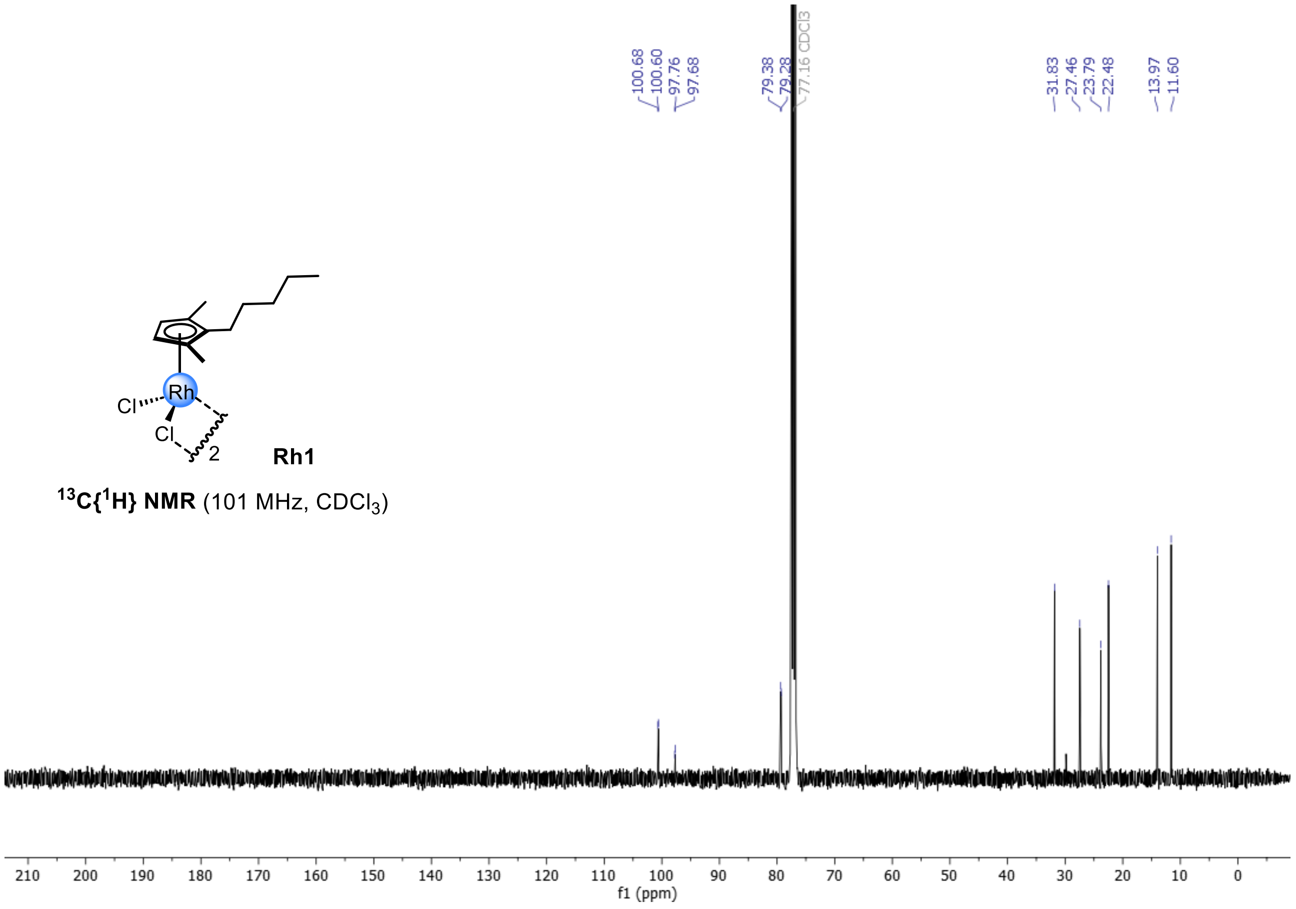
Rh
Cl
Cl
2
Rh1
^{13}C{^{1}H} NMR (101 MHz, CDCl$_3$)
100.68
100.60
97.76
97.68
79.38
79.28
77.16 CDCl3
31.83
27.46
23.79
22.48
13.97
11.60
f1 (ppm)

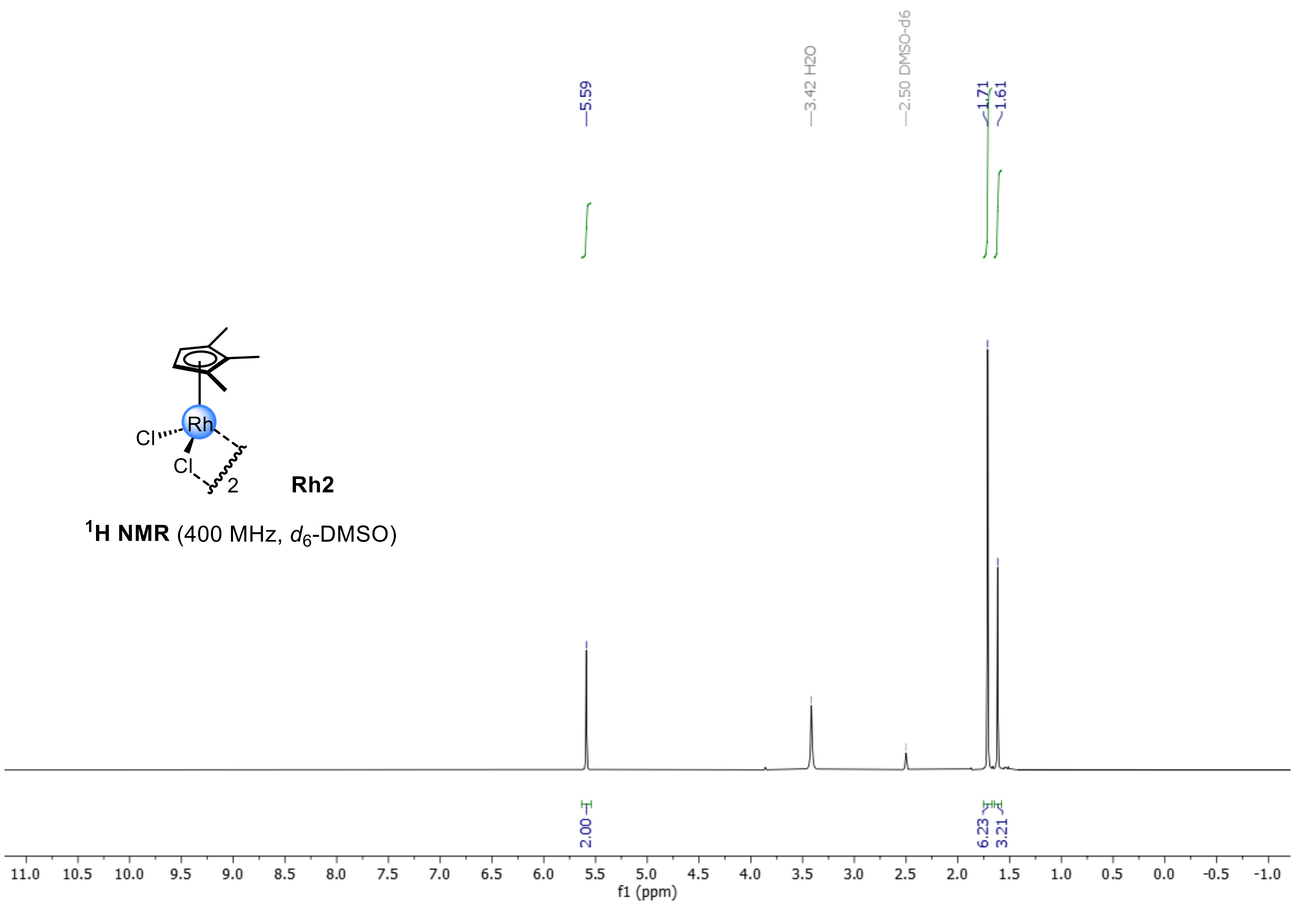

Rh2

^{1}H NMR (400 MHz, d_6-DMSO)

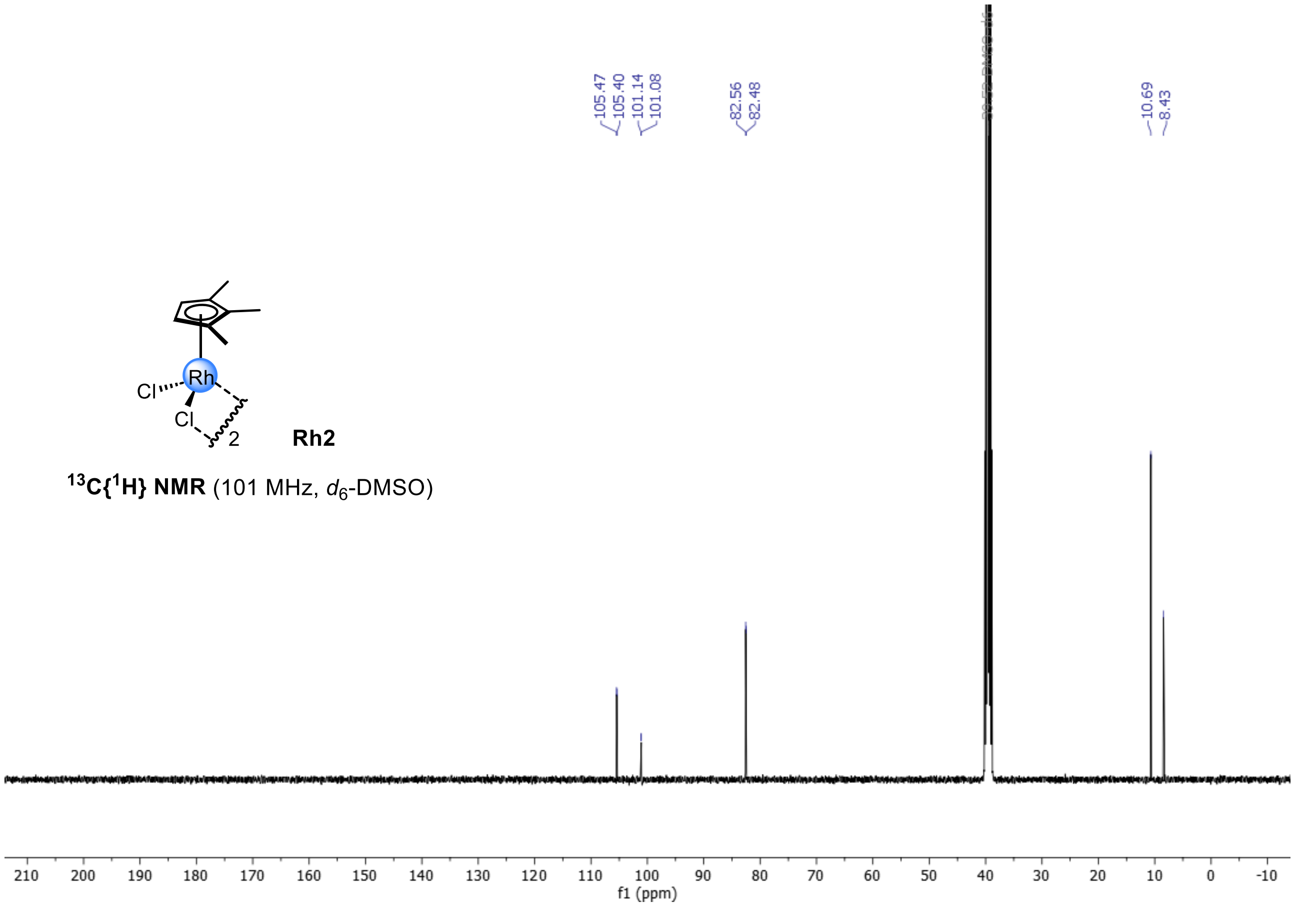

Rh2

$^{13}C\{^{1}H\}$ NMR (101 MHz, d_6-DMSO)

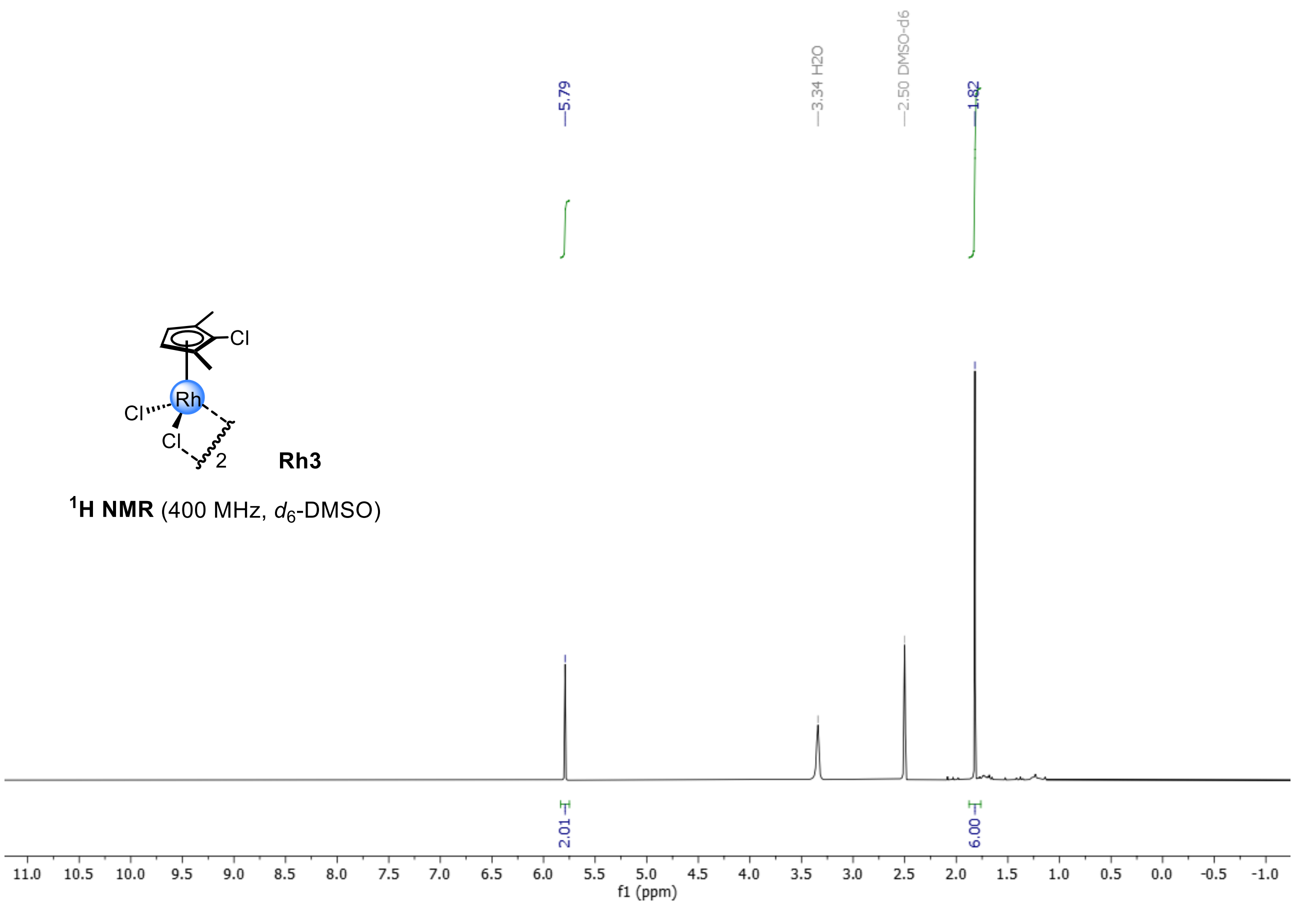
5.79
3.34 H2O
2.50 DMSO-d6
1.82
Cl
Rh
Cl
Cl
2
Rh3
^{1}H NMR (400 MHz, d_6-DMSO)
2.01
6.00
11.0 10.5 10.0 9.5 9.0 8.5 8.0 7.5 7.0 6.5 6.0 5.5 5.0 4.5 4.0 3.5 3.0 2.5 2.0 1.5 1.0 0.5 0.0 -0.5 -1.0
f1 (ppm)

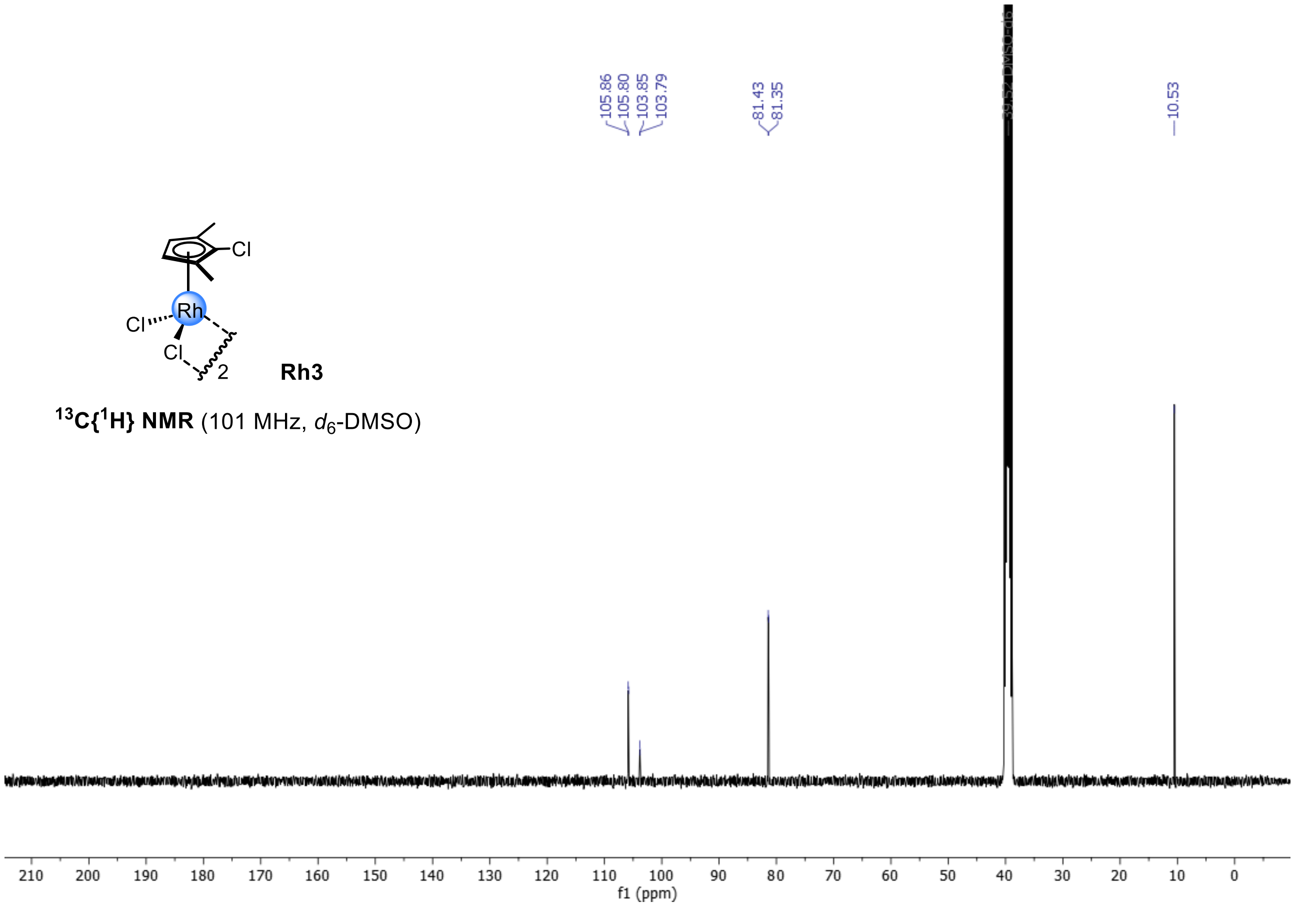
105.86
105.80
103.85
103.79
81.43
81.35
39.52 DMSO-d6
10.53
Cl
Rh
Cl
Cl
2
Rh3
^{13}C{^{1}H} NMR (101 MHz, d_6-DMSO)
210 200 190 180 170 160 150 140 130 120 110 100 90 80 70 60 50 40 30 20 10 0
f1 (ppm)

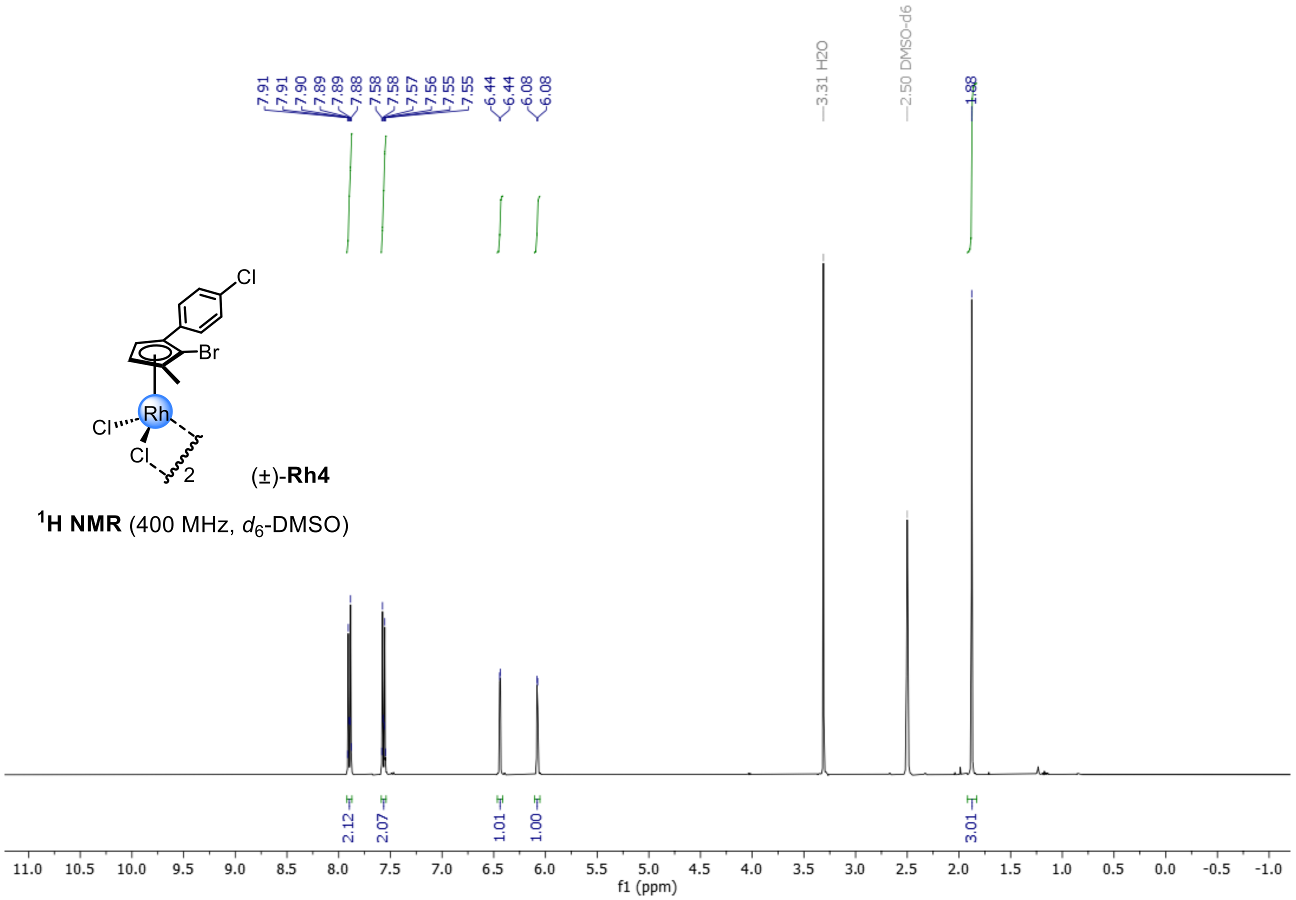
7.91
7.91
7.90
7.89
7.89
7.88
7.58
7.58
7.57
7.56
7.55
7.55
6.44
6.44
6.08
6.08
3.31 H2O
2.50 DMSO-d6
1.88
Cl
Br
Rh
Cl
Cl
2
(±)-**Rh4**
^{1}**H NMR** (400 MHz, d_6-DMSO)
2.12
2.07
1.01
1.00
3.01
f1 (ppm)

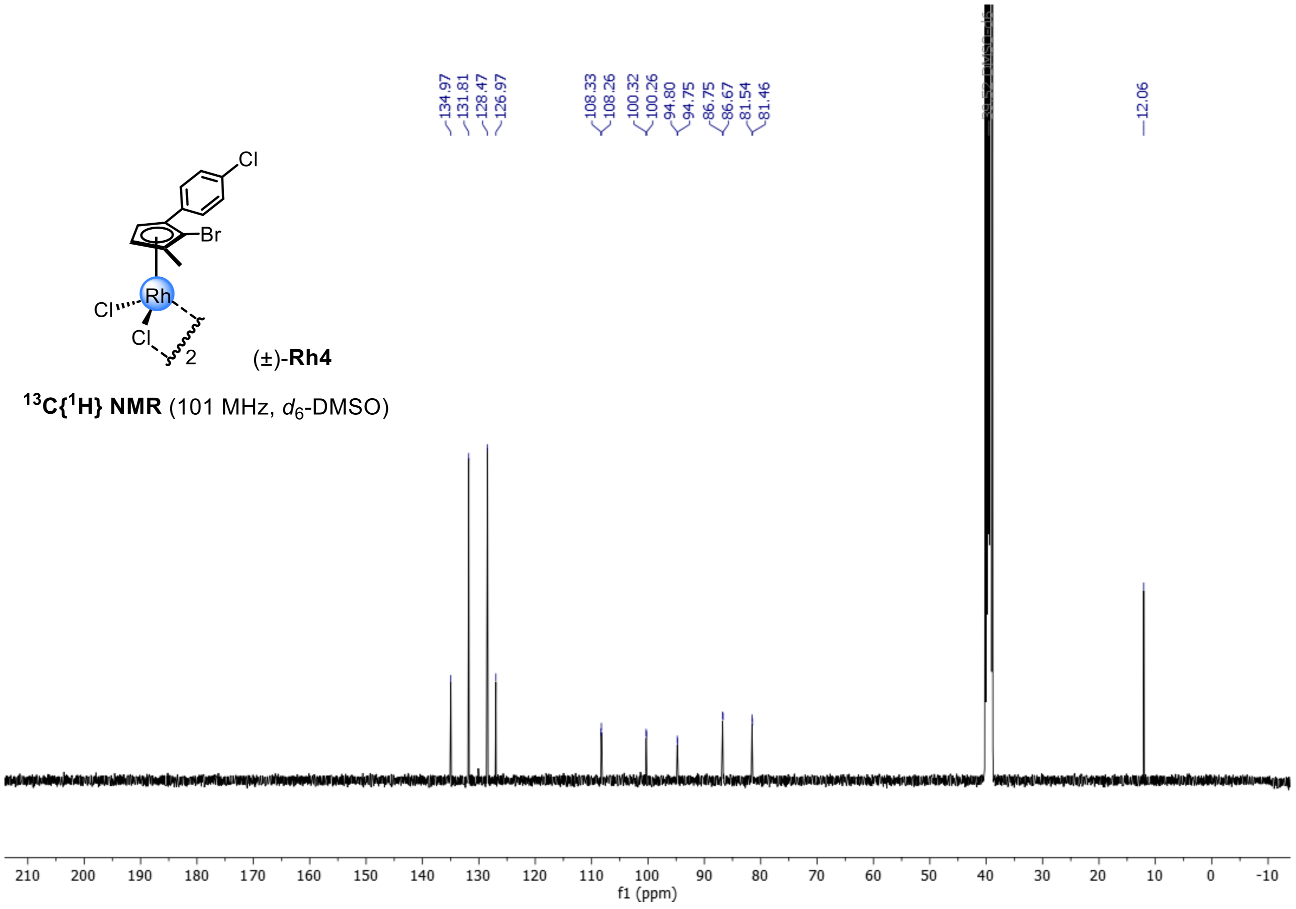
134.97
131.81
128.47
126.97
108.33
108.26
100.32
100.26
94.80
94.75
86.75
86.67
81.54
81.46
12.06
Cl
Br
Rh
Cl
Cl
2
(±)-**Rh4**
^{13}**C{^{1}H} NMR** (101 MHz, d_6-DMSO)
f1 (ppm)

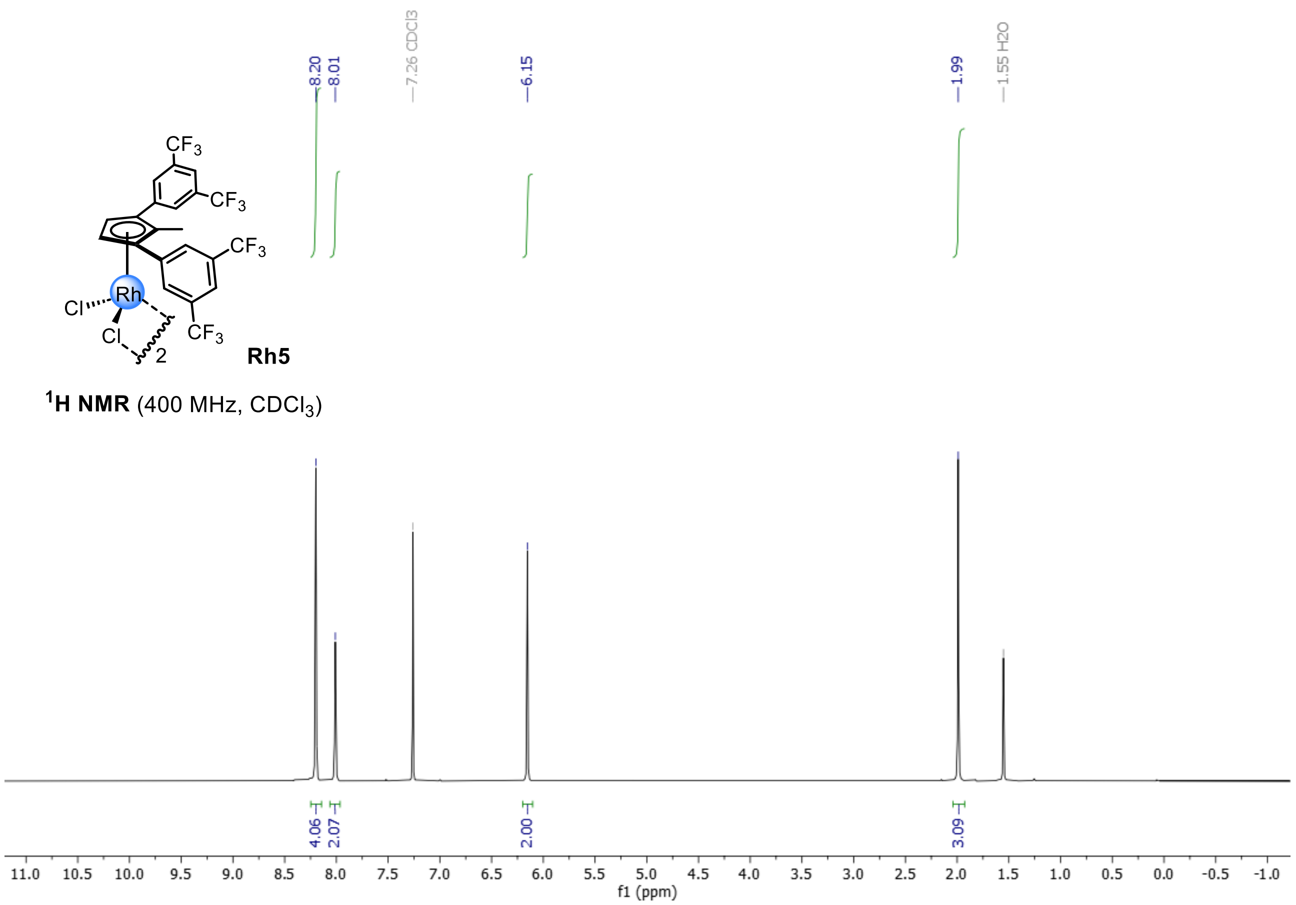
8.20
8.01
7.26 CDCl3
6.15
1.99
1.55 H2O
CF3
Rh
Cl
2
Rh5
1H NMR (400 MHz, CDCl3)
4.06
2.07
2.00
3.09
11.0 10.5 10.0 9.5 9.0 8.5 8.0 7.5 7.0 6.5 6.0 5.5 5.0 4.5 4.0 3.5 3.0 2.5 2.0 1.5 1.0 0.5 0.0 -0.5 -1.0
f1 (ppm)

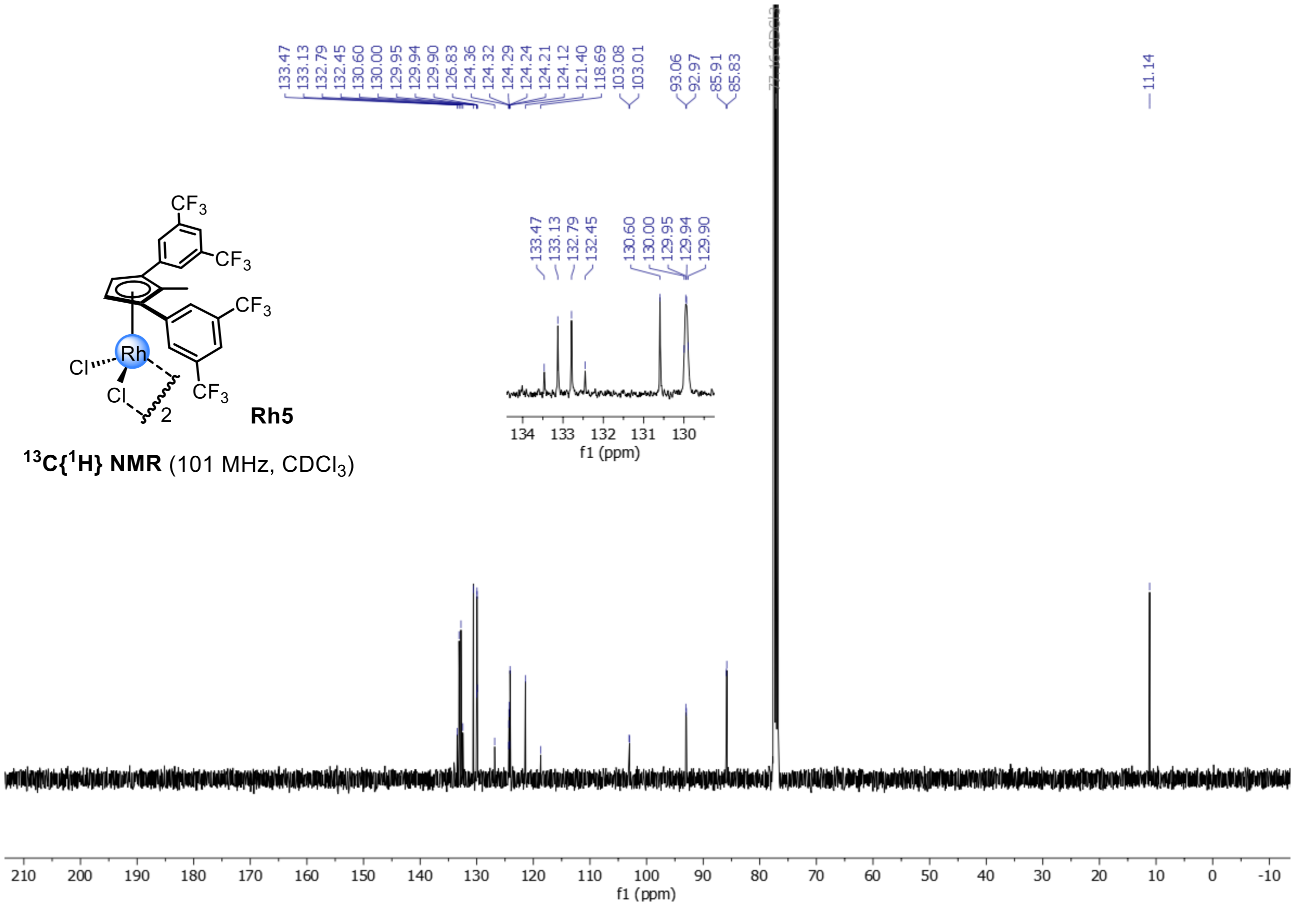
133.47
133.13
132.79
132.45
130.60
130.00
129.95
129.94
129.90
126.83
124.36
124.32
124.29
124.24
124.21
124.12
121.40
118.69
103.08
103.01
93.06
92.97
85.91
85.83
11.14
CF3
Rh
Cl
2
Rh5
13C{1H} NMR (101 MHz, CDCl3)
134 133 132 131 130
f1 (ppm)
210 200 190 180 170 160 150 140 130 120 110 100 90 80 70 60 50 40 30 20 10 0 -10
f1 (ppm)

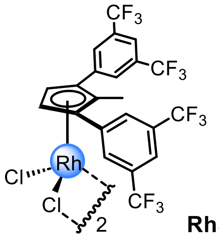

Rh5

^{19}F{^{1}H} NMR (376 MHz, $CDCl_3$)

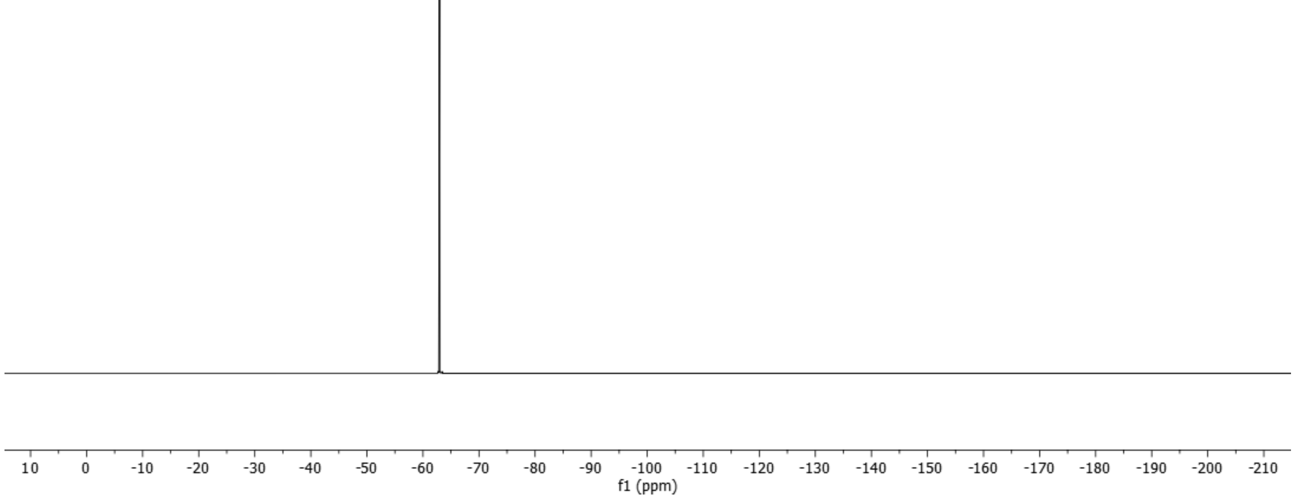

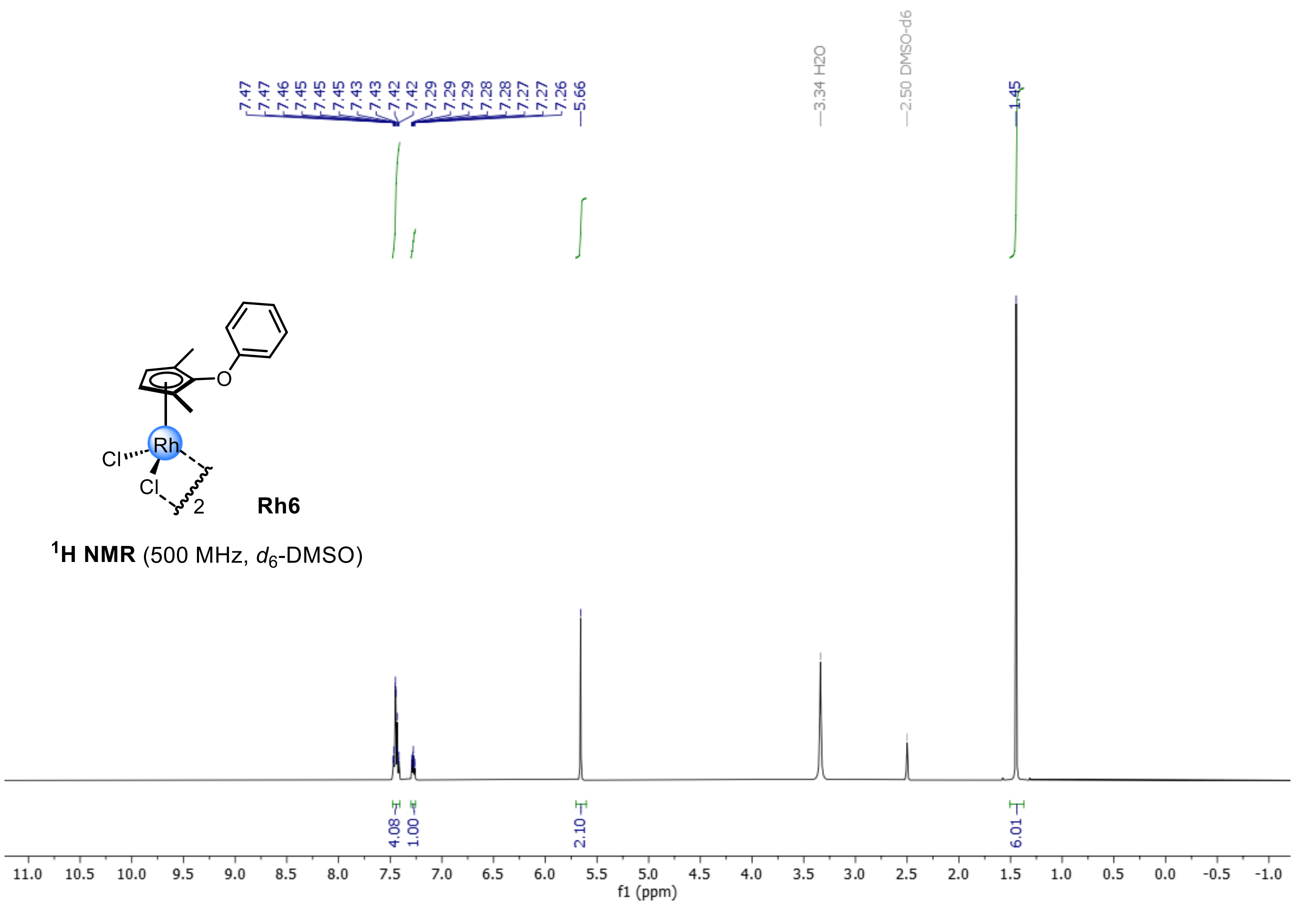
7.47
7.47
7.46
7.45
7.45
7.45
7.43
7.43
7.42
7.42
7.29
7.29
7.29
7.28
7.28
7.27
7.27
7.26
5.66
3.34 H2O
2.50 DMSO-d6
1.45
Cl
Rh
Cl
O
2
Rh6
¹H NMR (500 MHz, d6-DMSO)
4.08
1.00
2.10
6.01
f1 (ppm)

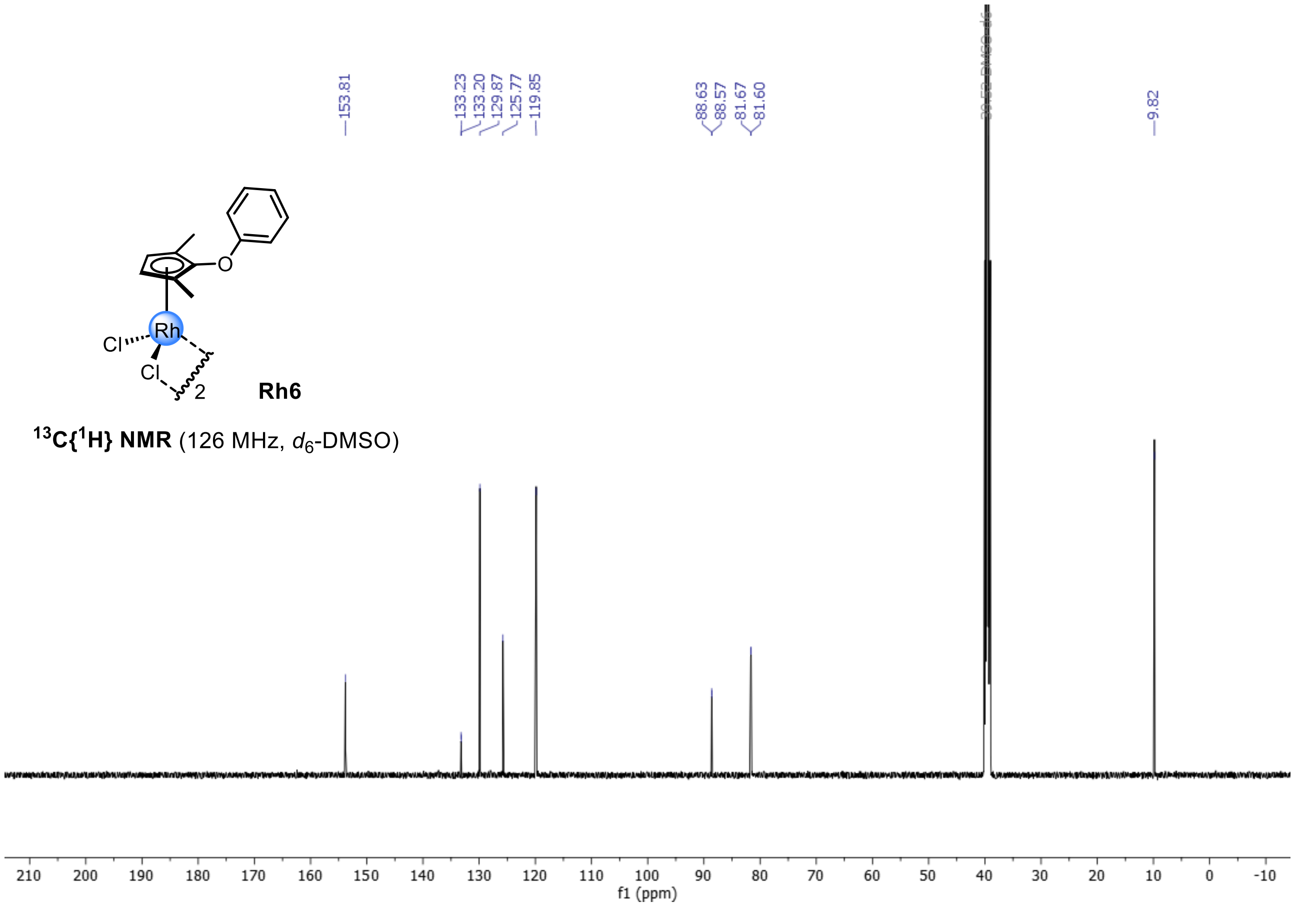
153.81
133.23
133.20
129.87
125.77
119.85
88.63
88.57
81.67
81.60
9.82
Cl
Rh
Cl
O
2
Rh6
¹³C{¹H} NMR (126 MHz, d6-DMSO)
f1 (ppm)

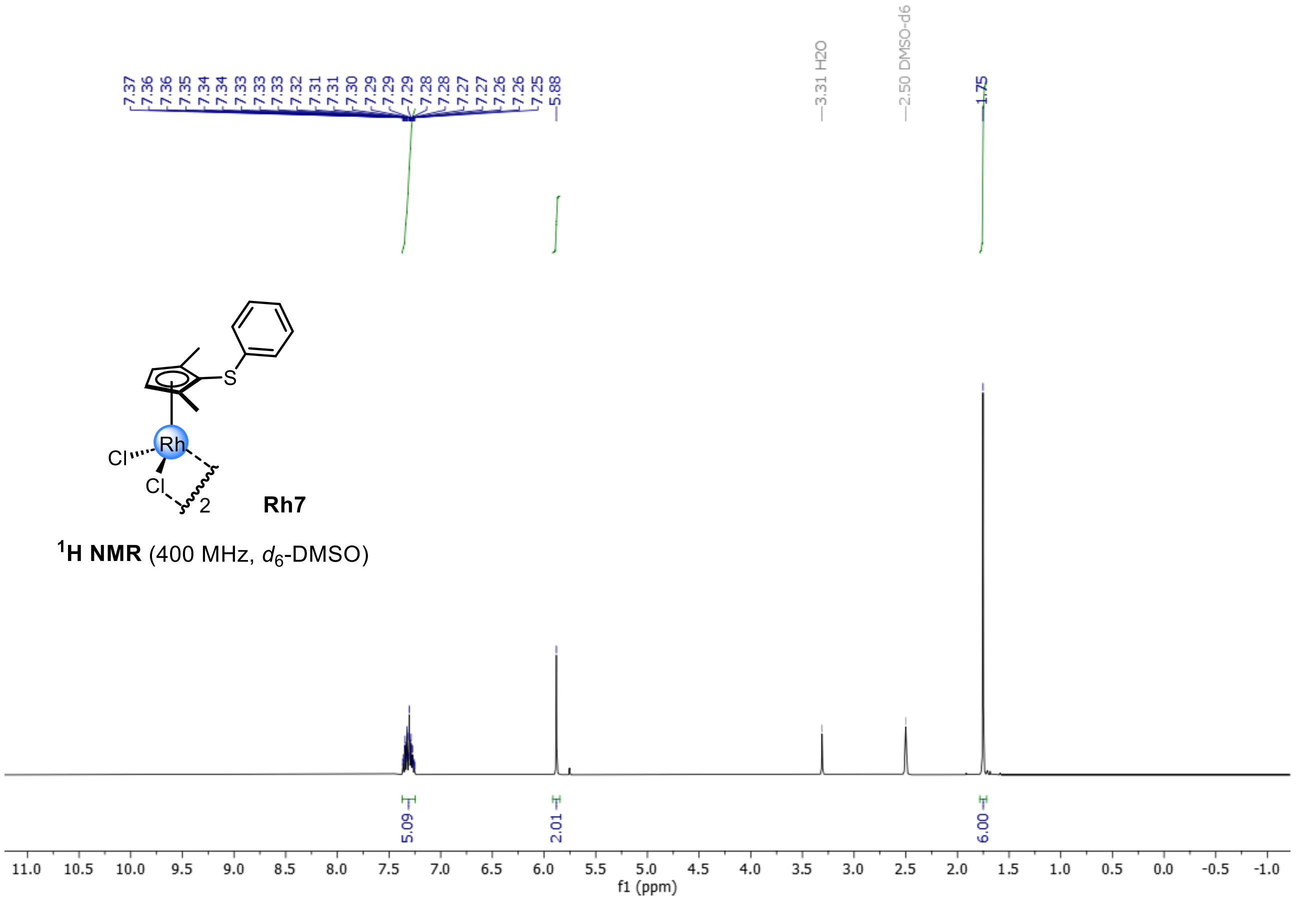

Rh7

^{1}H NMR (400 MHz, d_6-DMSO)

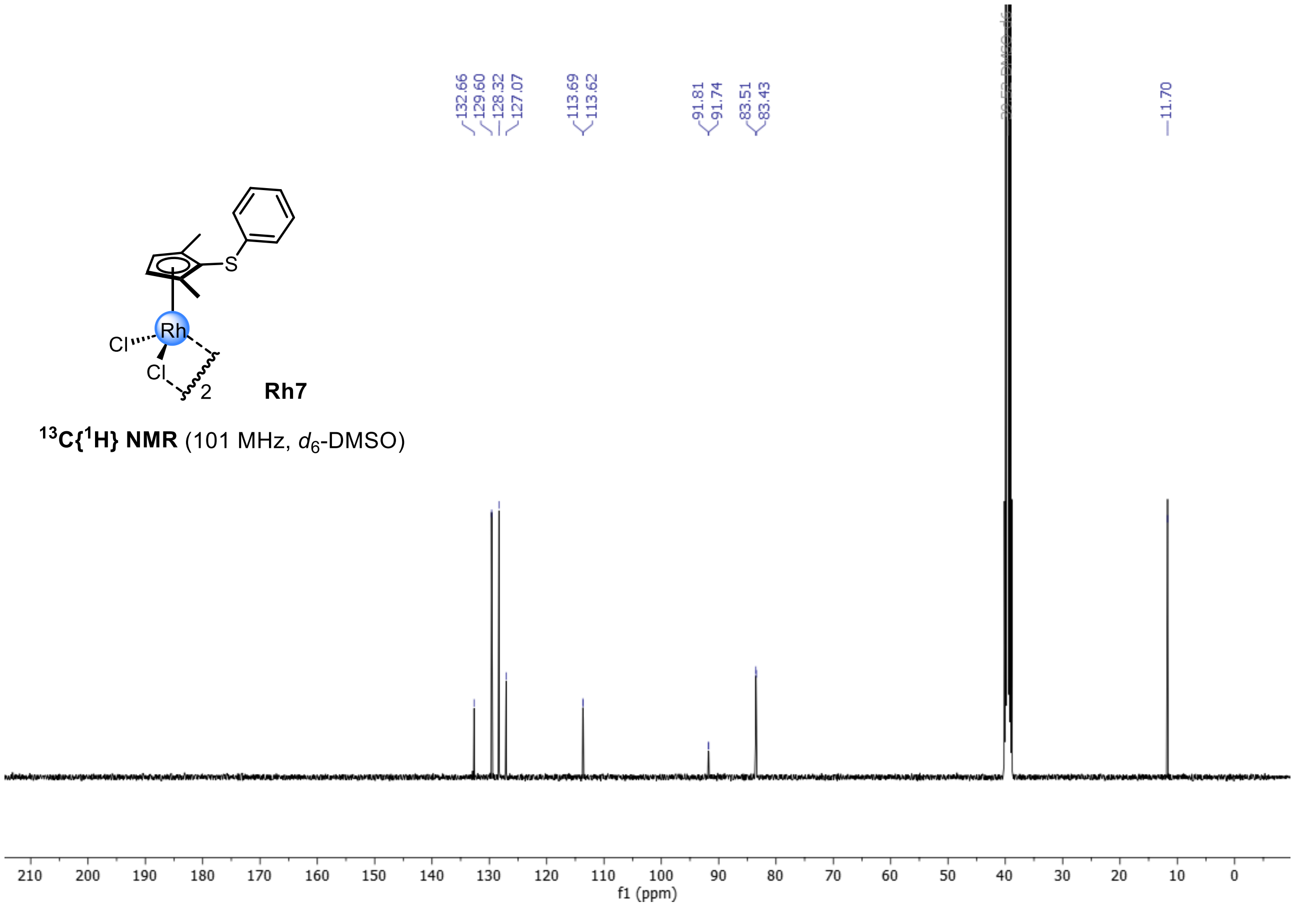

Rh7

^{13}C{^{1}H} NMR (101 MHz, d_6-DMSO)

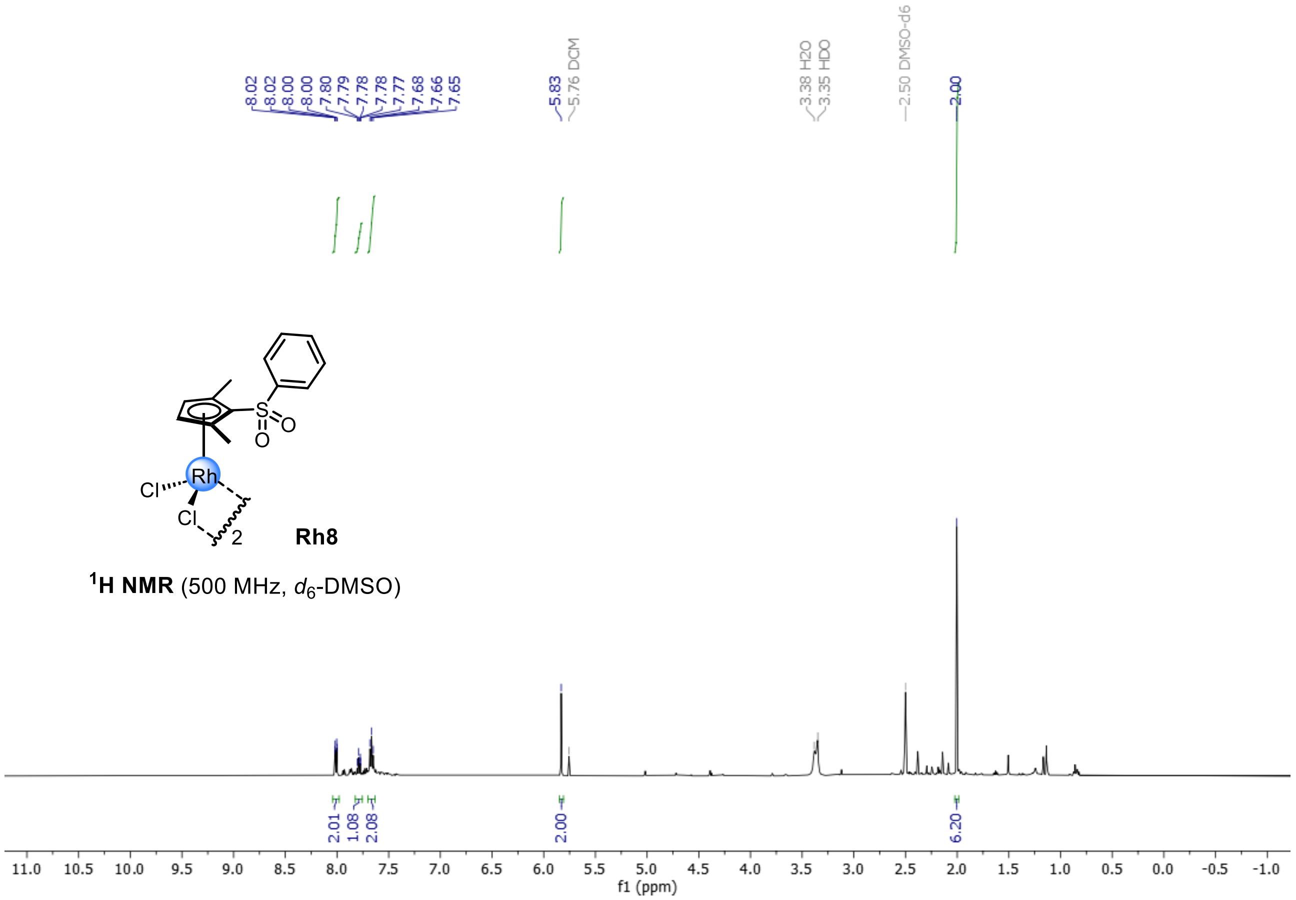

Rh8

^{1}H NMR (500 MHz, d_6-DMSO)

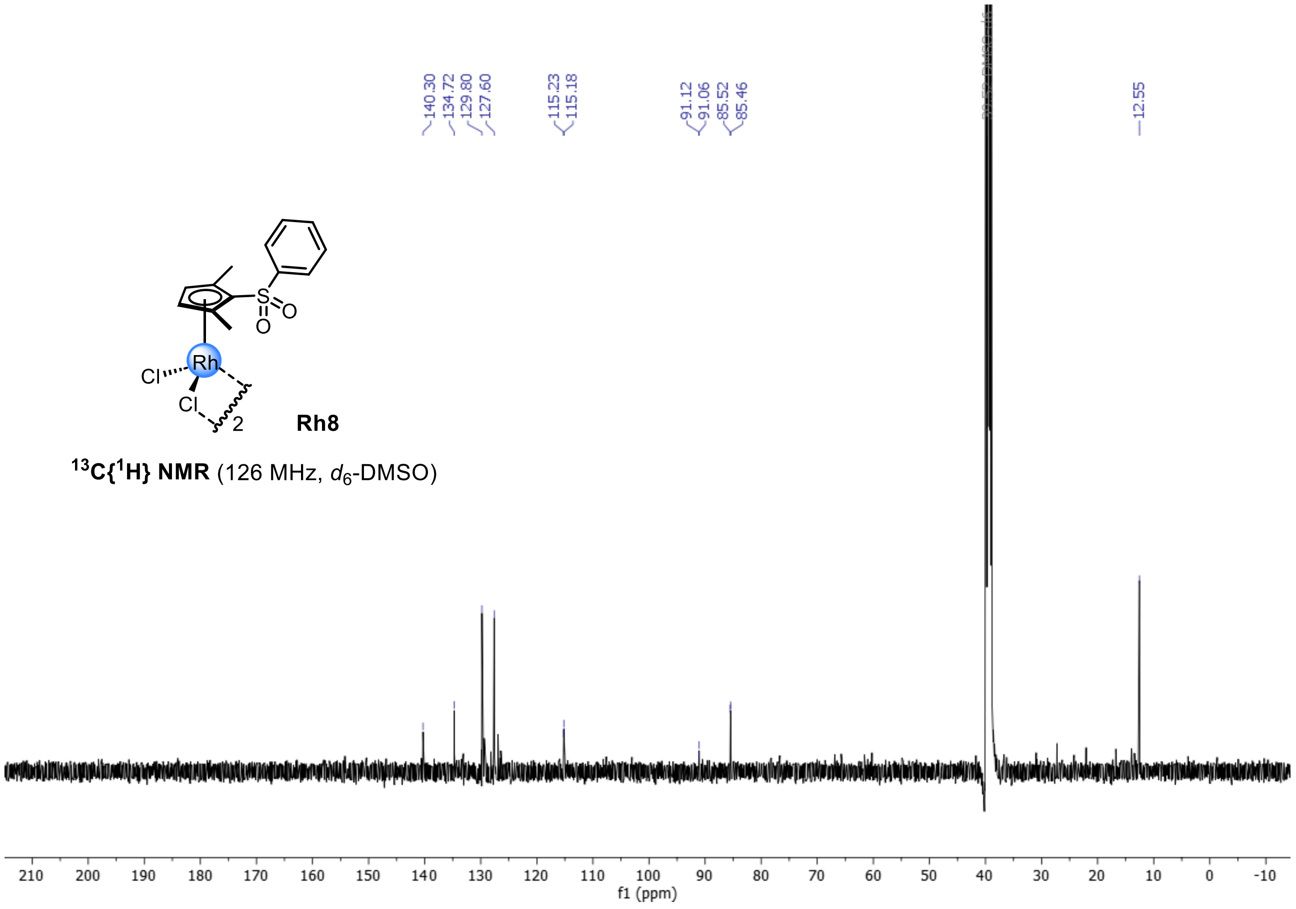

Rh8

^{13}C{^{1}H} NMR (126 MHz, d_6-DMSO)

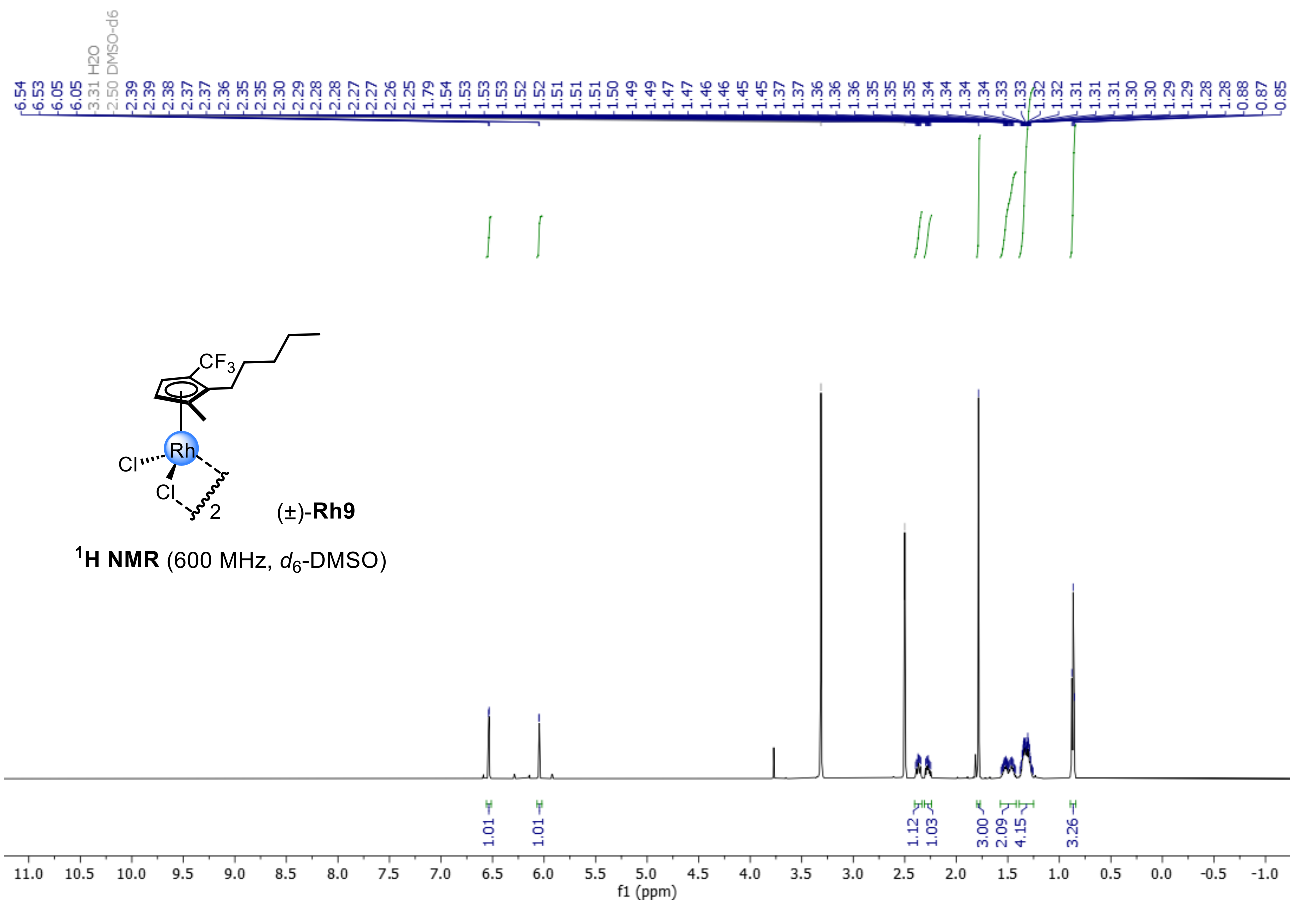
(±)-Rh9
1H NMR (600 MHz, d6-DMSO)

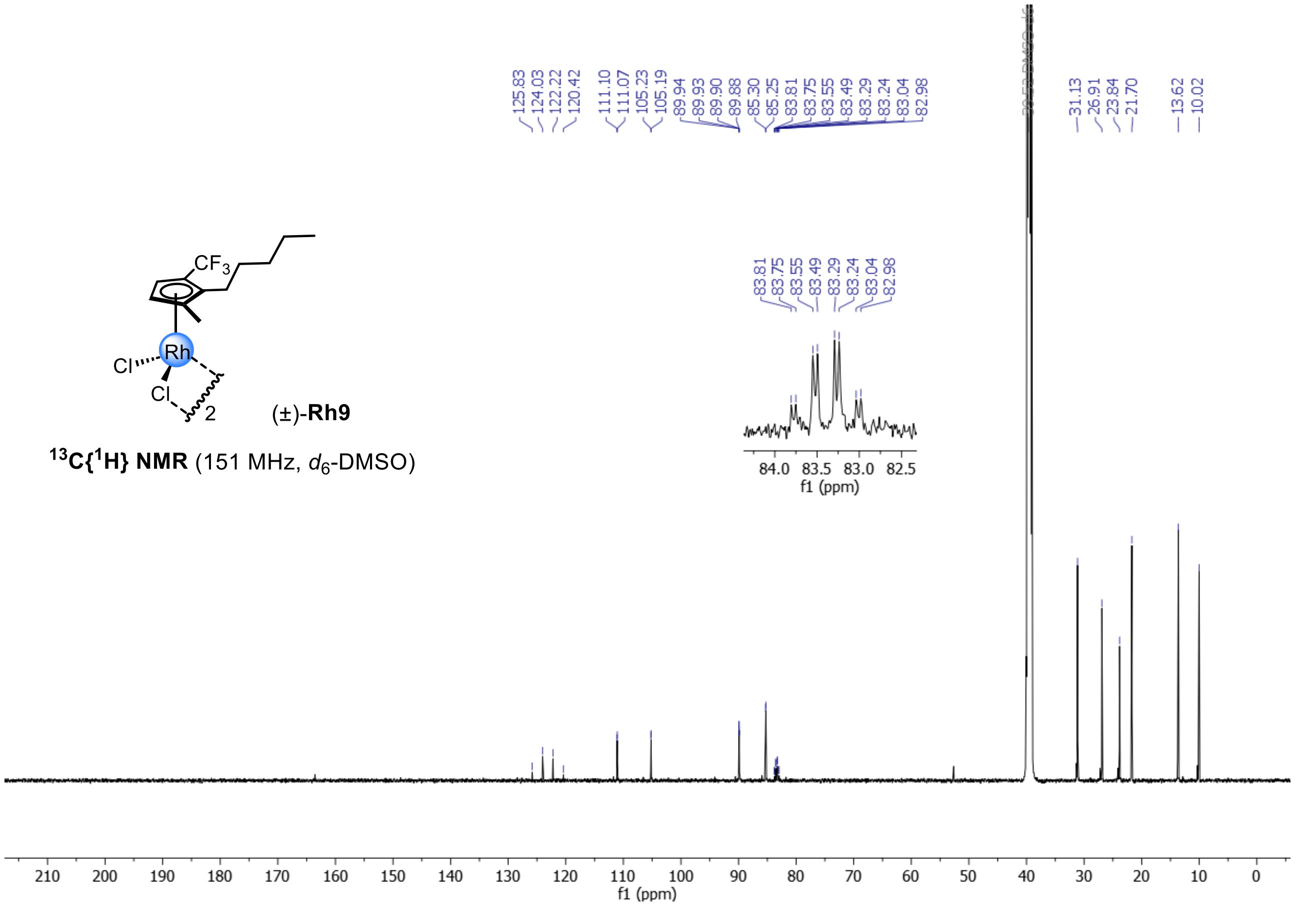
(±)-Rh9
13C{1H} NMR (151 MHz, d6-DMSO)

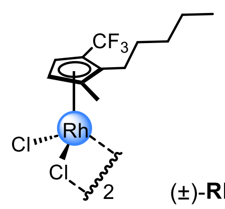

(±)-**Rh9**

^{19}F{^{1}H} NMR (376 MHz, d_6-DMSO)

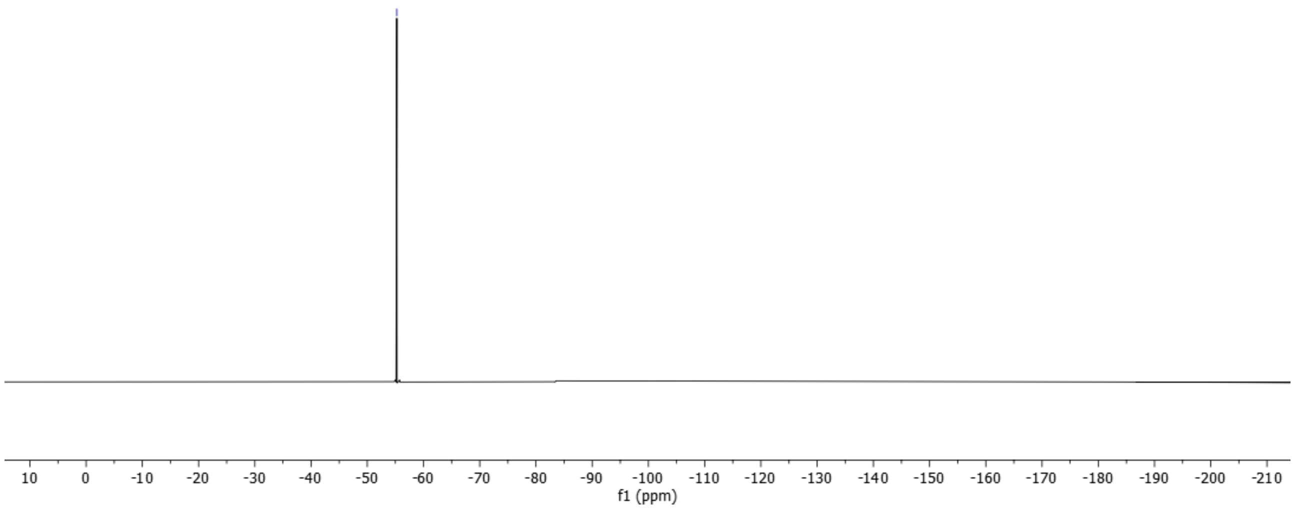

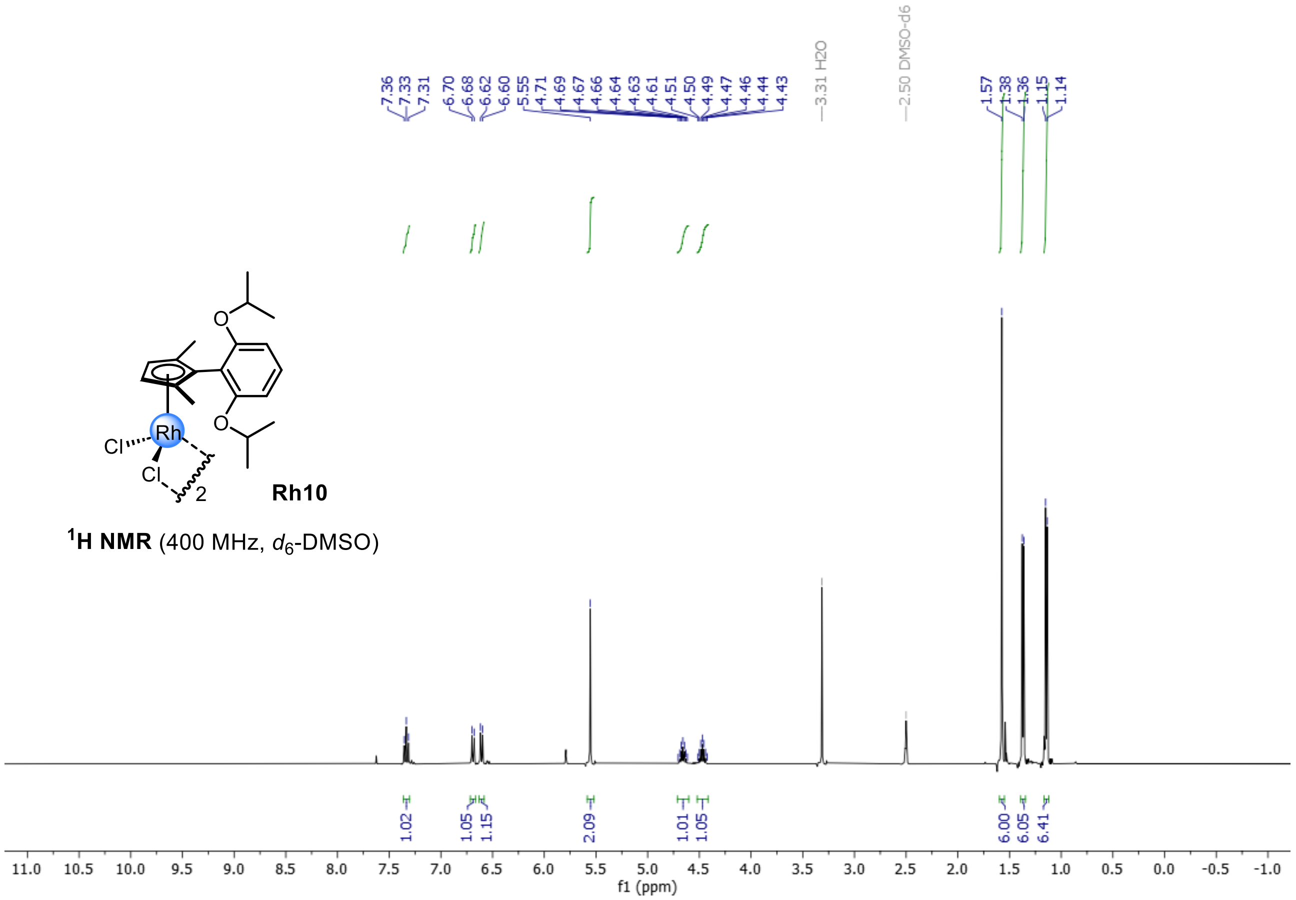
7.36
7.33
7.31
6.70
6.68
6.62
6.60
5.55
4.71
4.69
4.67
4.66
4.64
4.63
4.61
4.51
4.50
4.49
4.47
4.46
4.44
4.43
3.31 H2O
2.50 DMSO-d6
1.57
1.38
1.36
1.15
1.14
Rh
Cl
Cl
O
O
2
Rh10
1H NMR (400 MHz, d6-DMSO)
1.02
1.05
1.15
2.09
1.01
1.05
6.00
6.05
6.41
f1 (ppm)

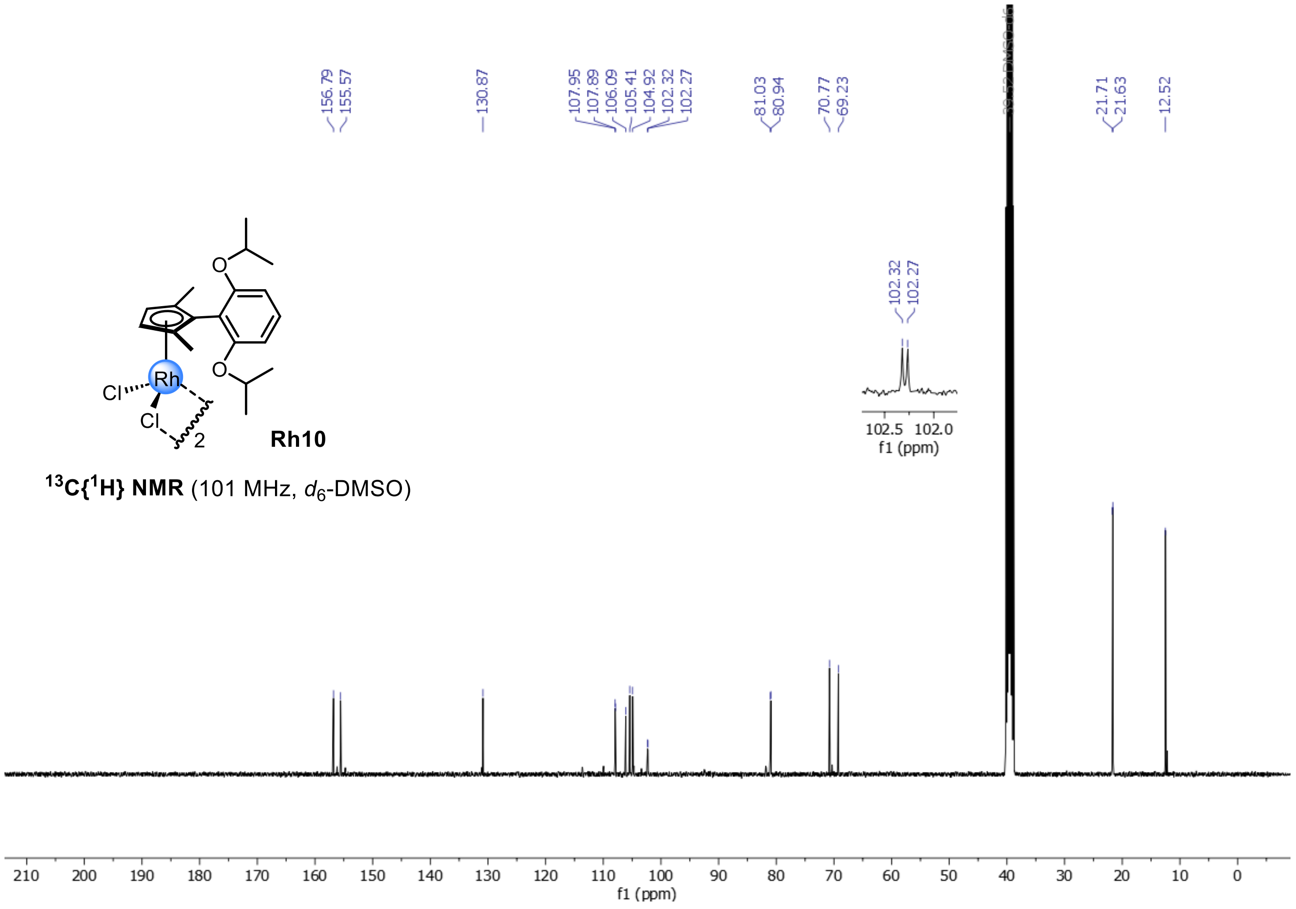
156.79
155.57
130.87
107.95
107.89
106.09
105.41
104.92
102.32
102.27
81.03
80.94
70.77
69.23
21.71
21.63
12.52
Rh
Cl
Cl
O
O
2
Rh10
13C{1H} NMR (101 MHz, d6-DMSO)
102.32
102.27
102.5
102.0
f1 (ppm)
f1 (ppm)

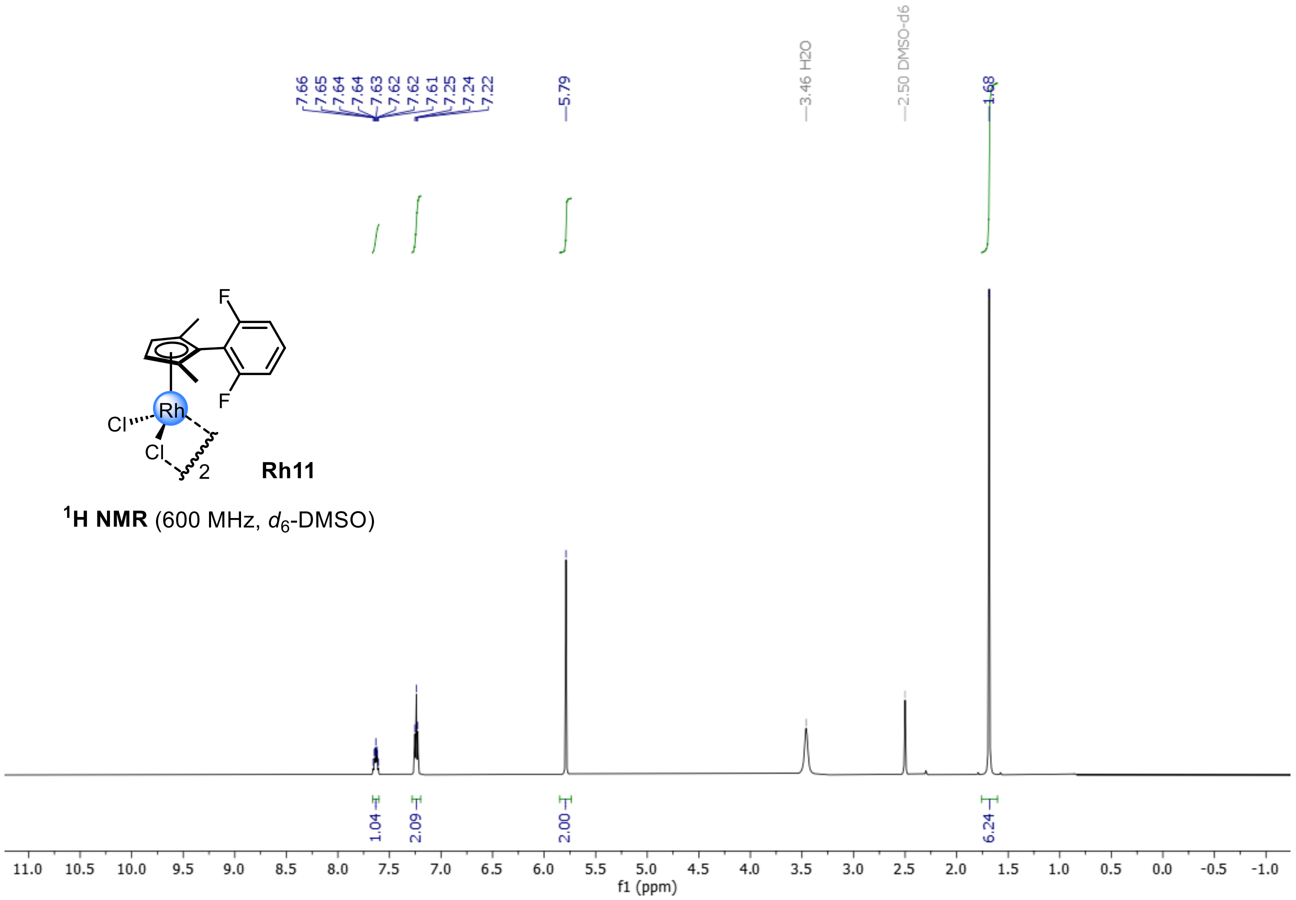
Rh11
^{1}H NMR (600 MHz, d_6-DMSO)
7.66 7.65 7.64 7.63 7.62 7.62 7.61 7.25 7.24 7.22
5.79
3.46 H2O
2.50 DMSO-d6
1.68
1.04
2.09
2.00
6.24
f1 (ppm)

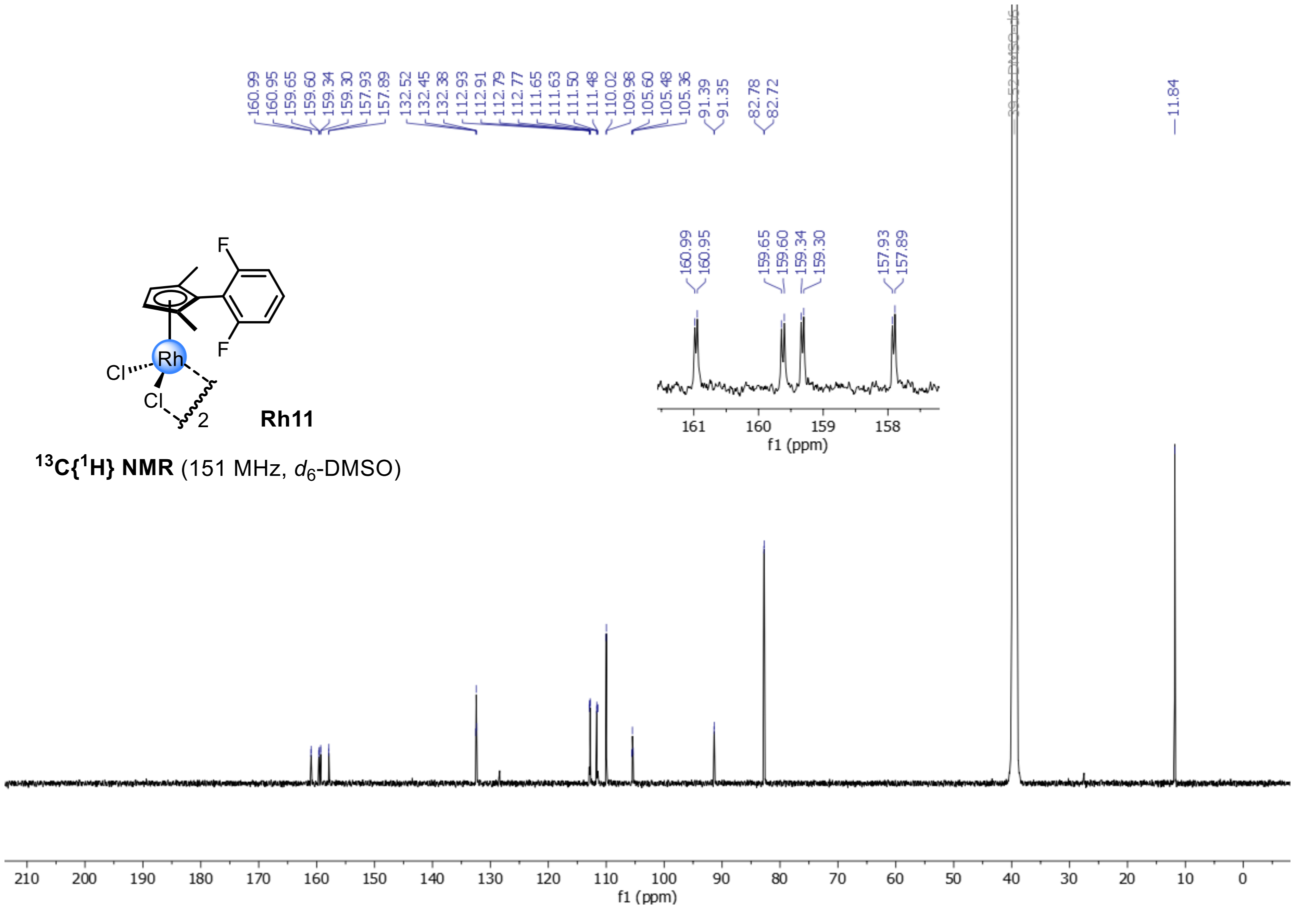
Rh11
^{13}C{^{1}H} NMR (151 MHz, d_6-DMSO)
160.99 160.95 159.65 159.60 159.34 159.30 157.93 157.89 132.52 132.45 132.38 112.93 112.91 112.79 112.77 111.65 111.63 111.50 111.48 110.02 109.98 105.60 105.48 105.36 91.39 91.35 82.78 82.72
39.52 DMSO-d6
11.84
f1 (ppm)

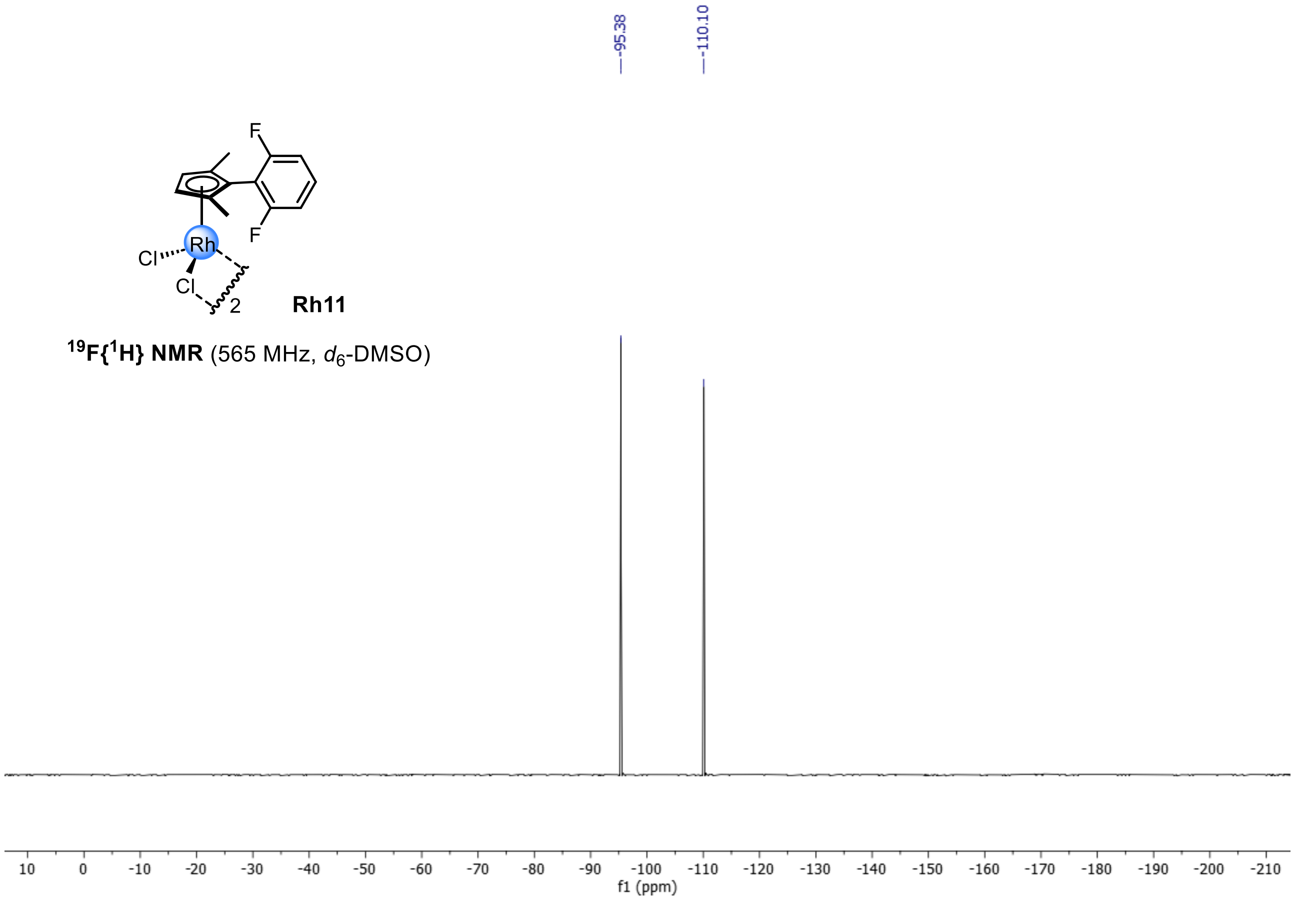
-95.38
-110.10
F
F
Rh
Cl
Cl
2
Rh11
^{19}F{^{1}H} NMR (565 MHz, d_6-DMSO)
10 0 -10 -20 -30 -40 -50 -60 -70 -80 -90 -100 -110 -120 -130 -140 -150 -160 -170 -180 -190 -200 -210
f1 (ppm)

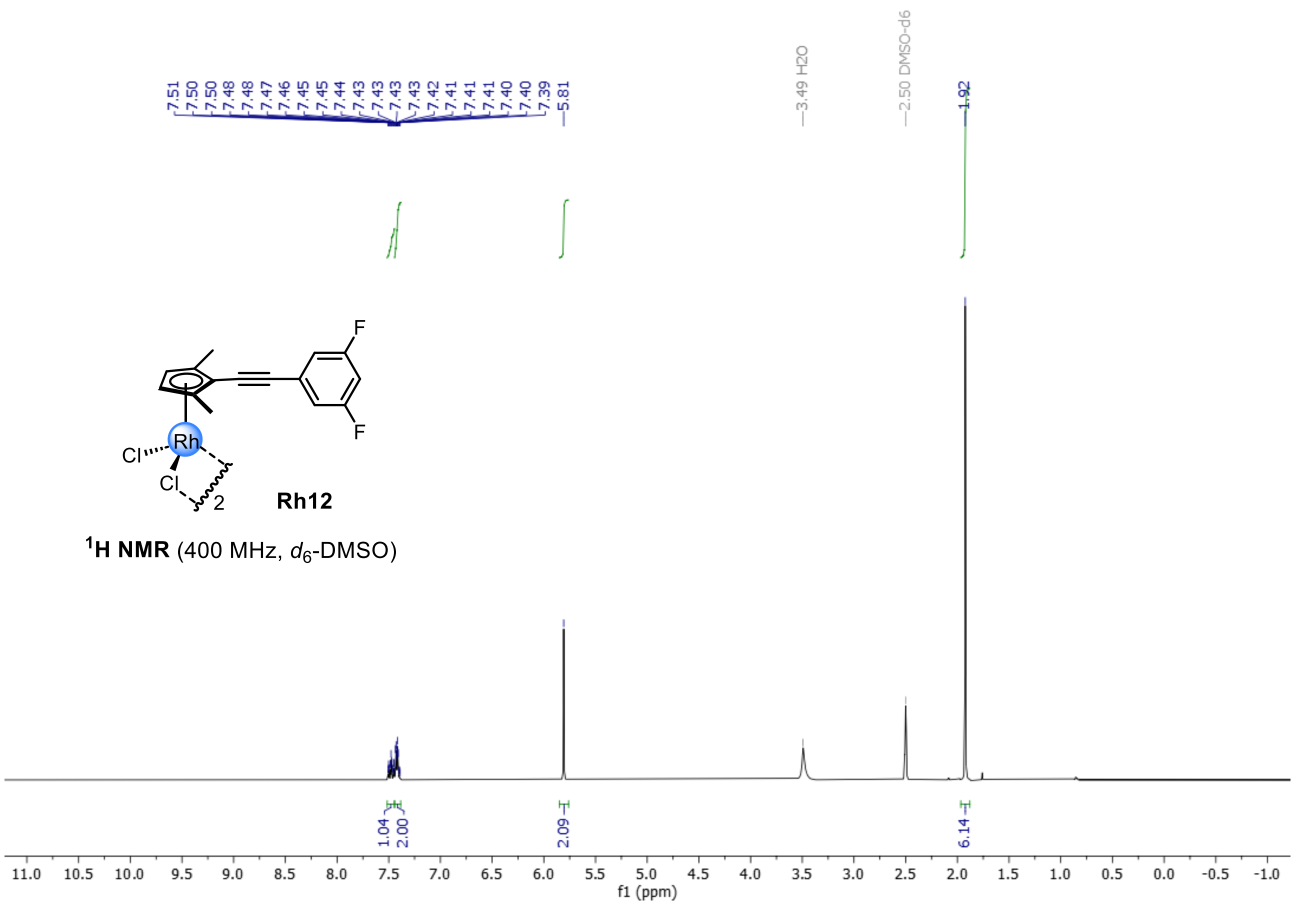
7.51
7.50
7.50
7.48
7.47
7.46
7.45
7.45
7.44
7.43
7.43
7.43
7.43
7.42
7.41
7.41
7.41
7.40
7.40
7.39
5.81
3.49 H2O
2.50 DMSO-d6
1.92
F
F
Rh
Cl
Cl
2
Rh12
1H NMR (400 MHz, d6-DMSO)
1.04
2.00
2.09
6.14
f1 (ppm)

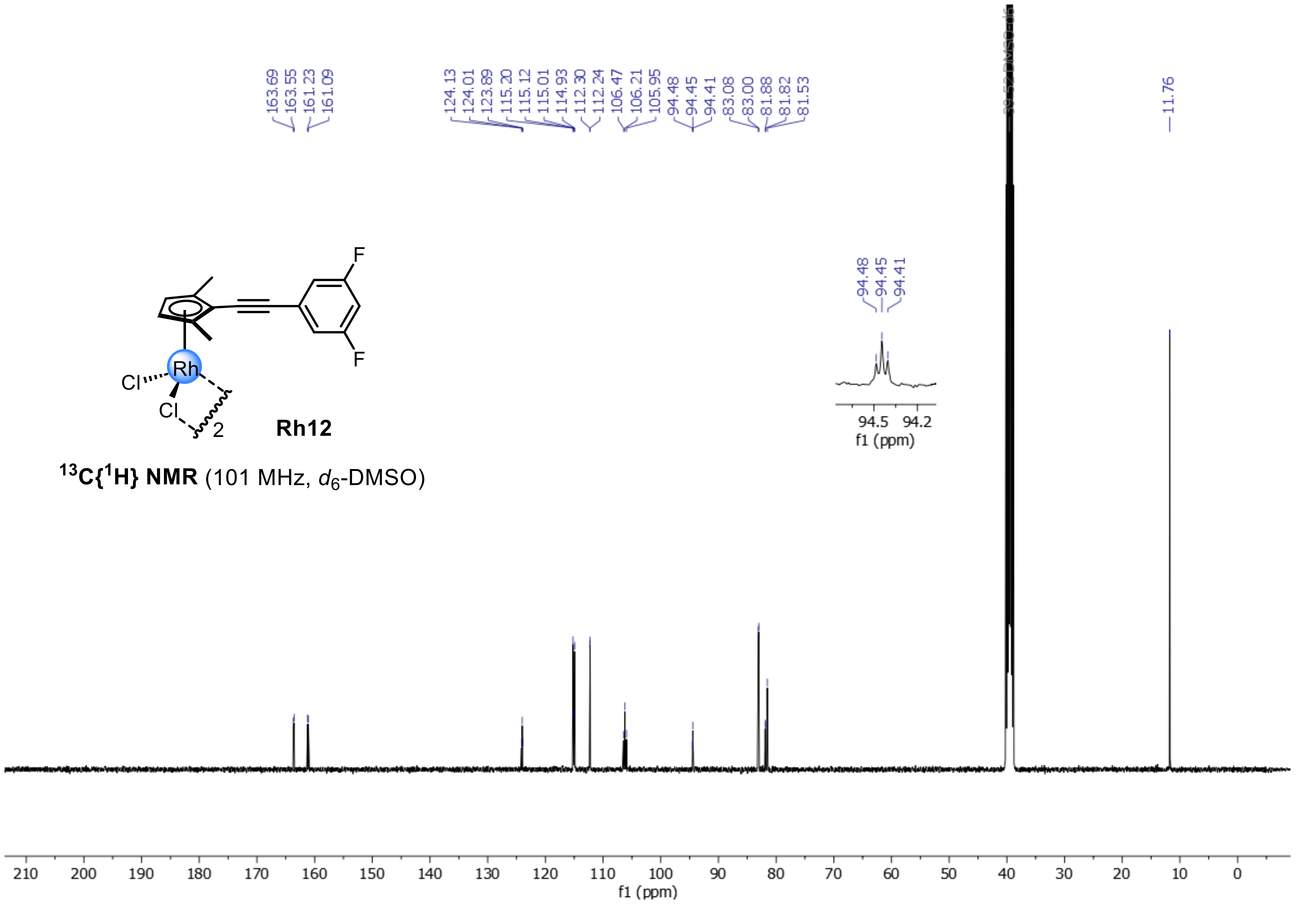
163.69
163.55
161.23
161.09
124.13
124.01
123.89
115.20
115.12
115.01
114.93
112.30
112.24
106.47
106.21
105.95
94.48
94.45
94.41
83.08
83.00
81.88
81.82
81.53
11.76
F
F
Rh
Cl
Cl
2
Rh12
13C{1H} NMR (101 MHz, d6-DMSO)
94.48
94.45
94.41
94.5 94.2
f1 (ppm)
f1 (ppm)

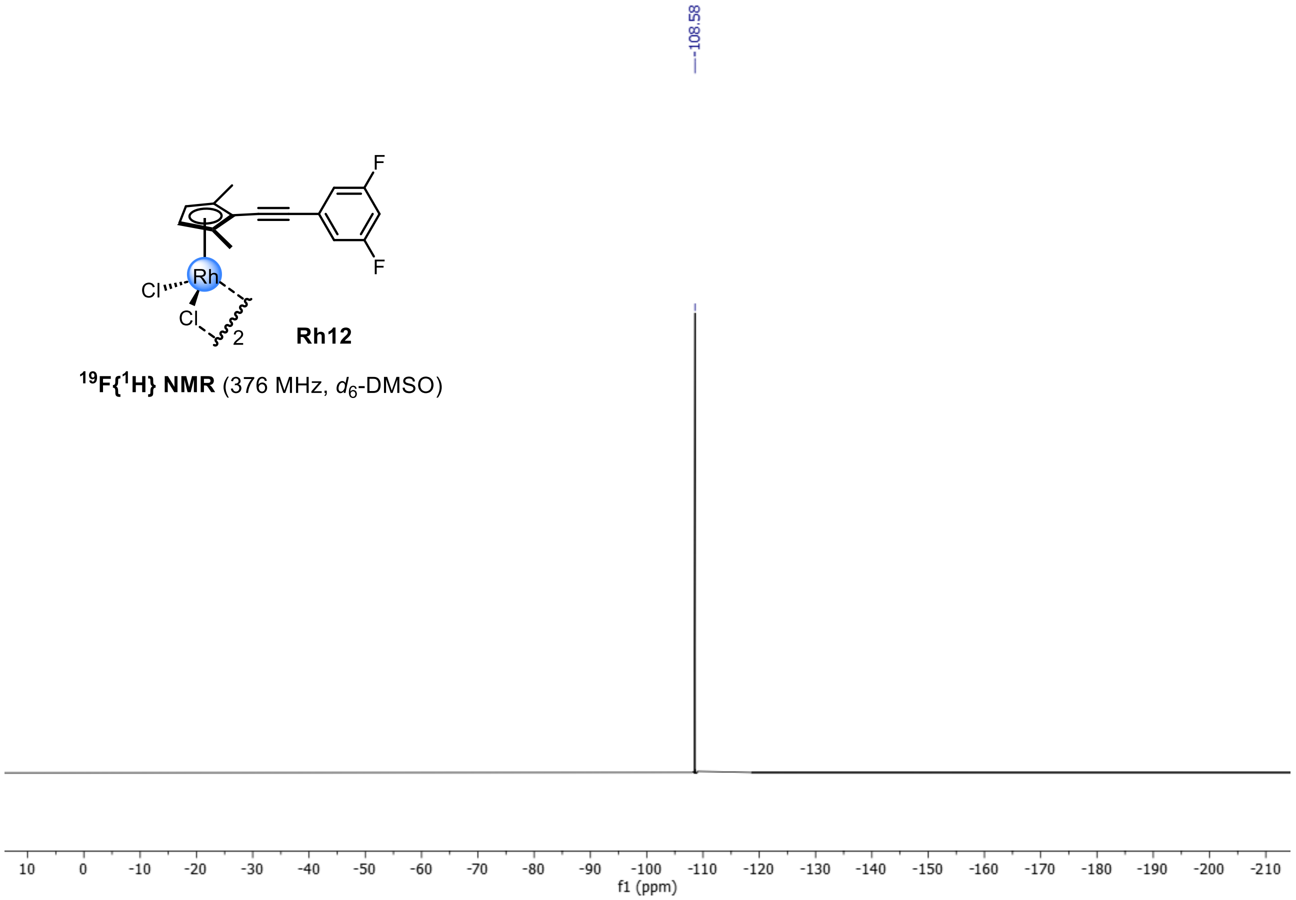

^{19}F{^{1}H} NMR (376 MHz, d_6-DMSO)

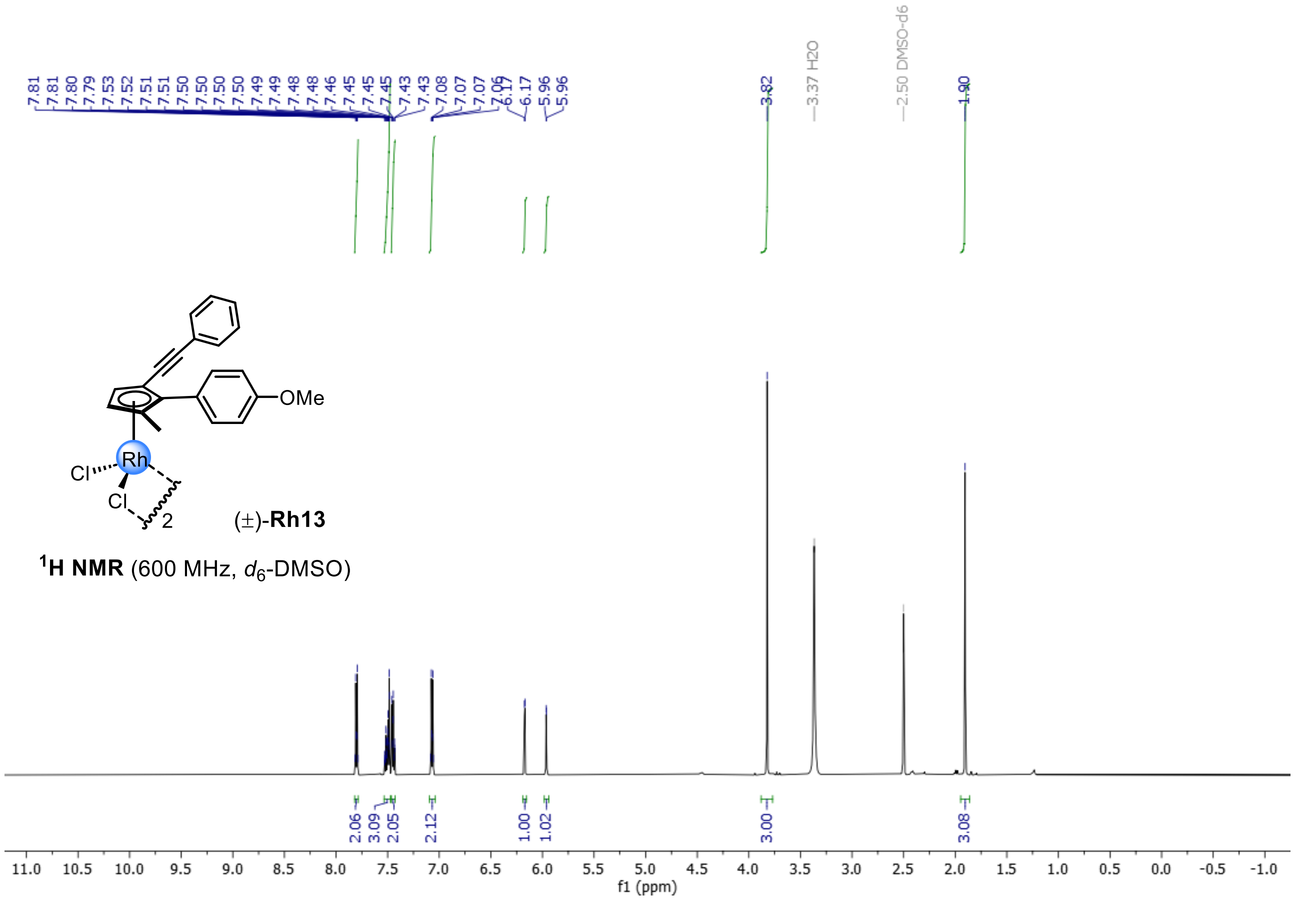
7.81 7.81 7.80 7.79 7.53 7.52 7.51 7.51 7.50 7.50 7.50 7.50 7.49 7.49 7.48 7.46 7.45 7.45 7.45 7.43 7.43 7.08 7.07 7.07 7.06 6.17 6.17 5.96 5.96
3.82
3.37 H2O
2.50 DMSO-d6
1.90
OMe
Rh
Cl
Cl
2
(±)-Rh13
1H NMR (600 MHz, d6-DMSO)
2.06 3.09 2.05 2.12 1.00 1.02 3.00 3.08
f1 (ppm)

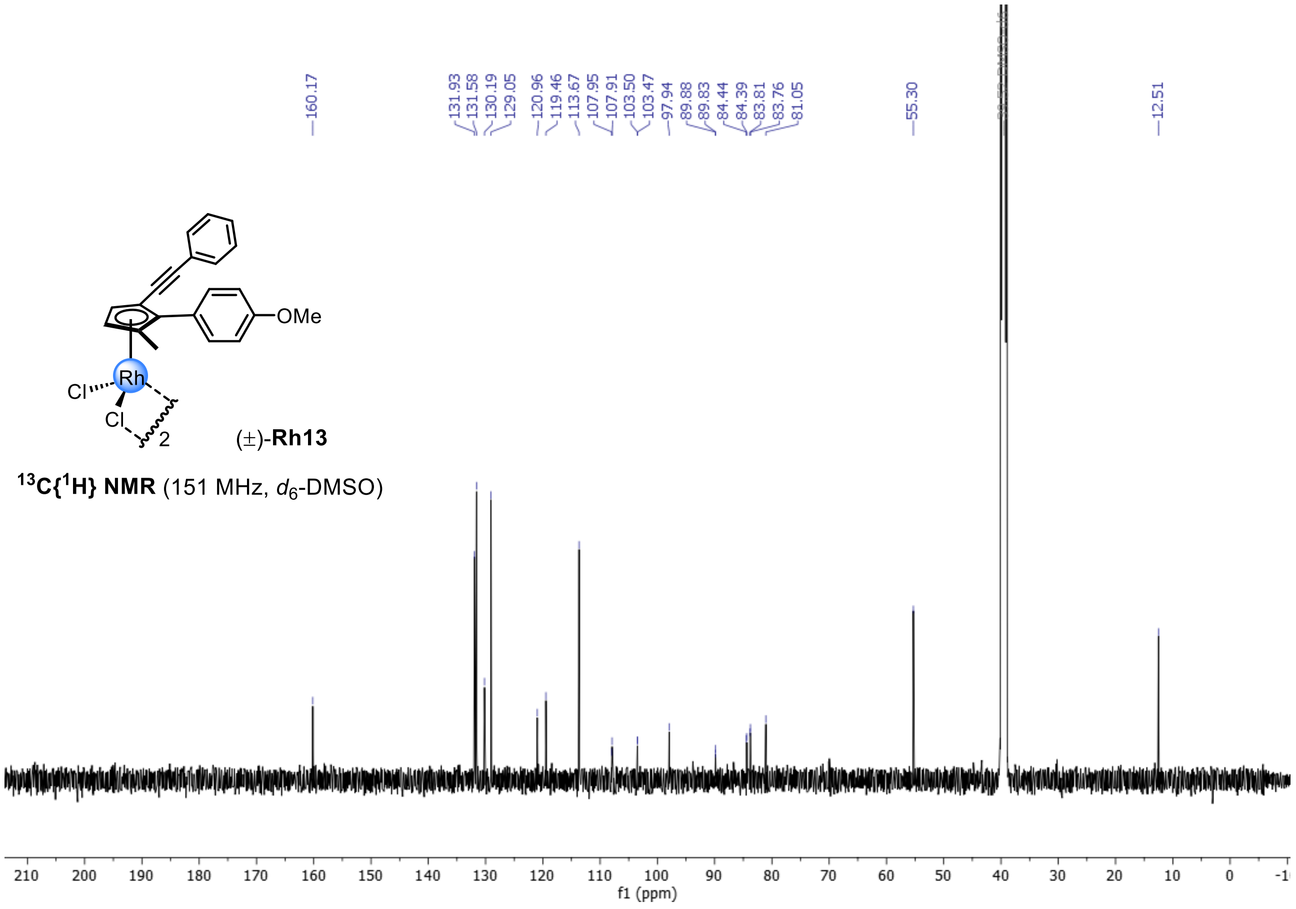
160.17
131.93 131.58 130.19 129.05 120.96 119.46 113.67 107.95 107.91 103.50 103.47 97.94 89.88 89.83 84.44 84.39 83.81 83.76 81.05
55.30
12.51
OMe
Rh
Cl
Cl
2
(±)-Rh13
13C{1H} NMR (151 MHz, d6-DMSO)
f1 (ppm)

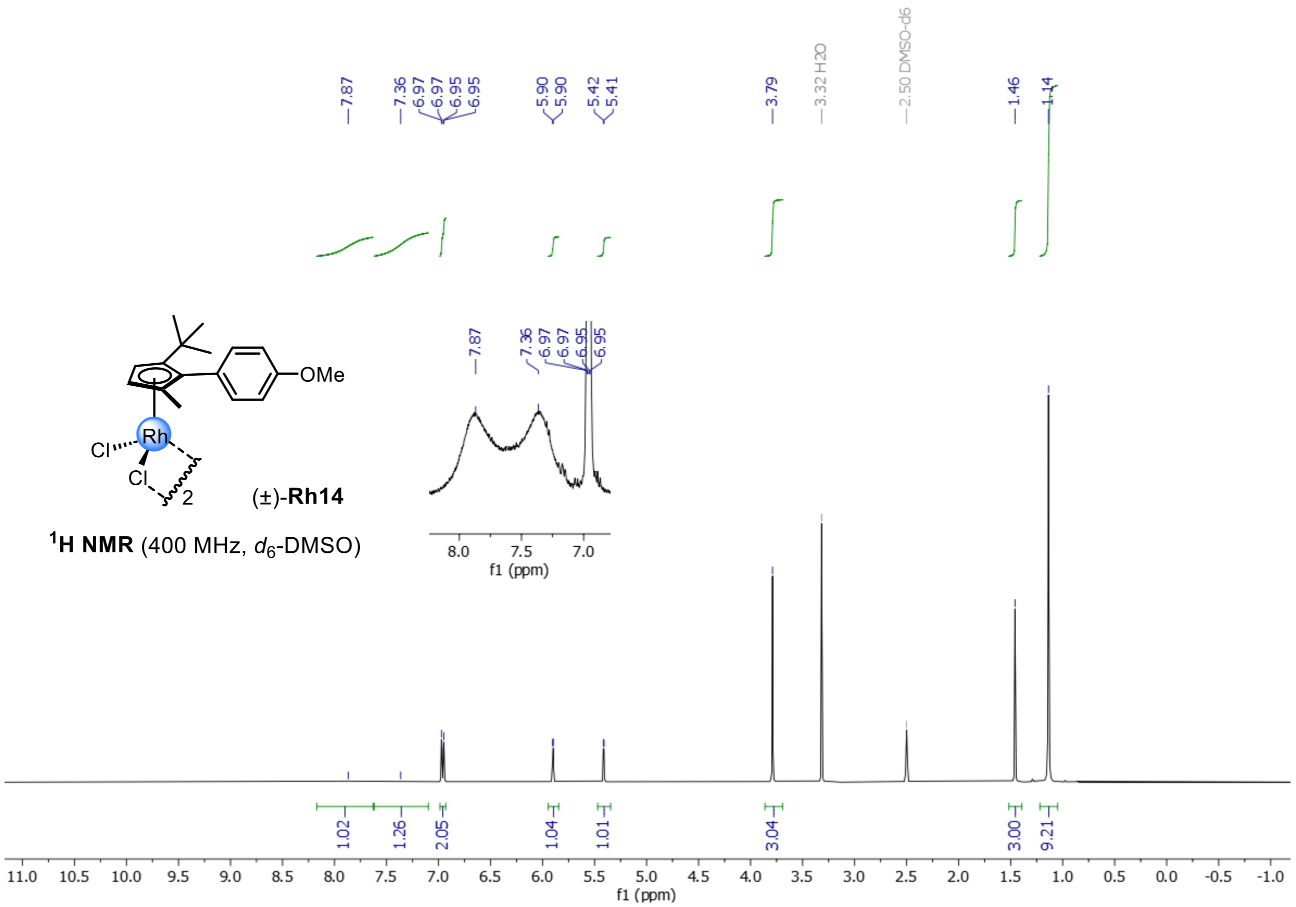
7.87
7.36
6.97
6.97
6.95
6.95
5.90
5.90
5.42
5.41
3.79
3.32 H2O
2.50 DMSO-d6
1.46
1.14
OMe
Rh
Cl
Cl
2
(±)-Rh14
^{1}H NMR (400 MHz, d_6-DMSO)
8.0
7.5
7.0
f1 (ppm)
1.02
1.26
2.05
1.04
1.01
3.04
3.00
9.21
f1 (ppm)

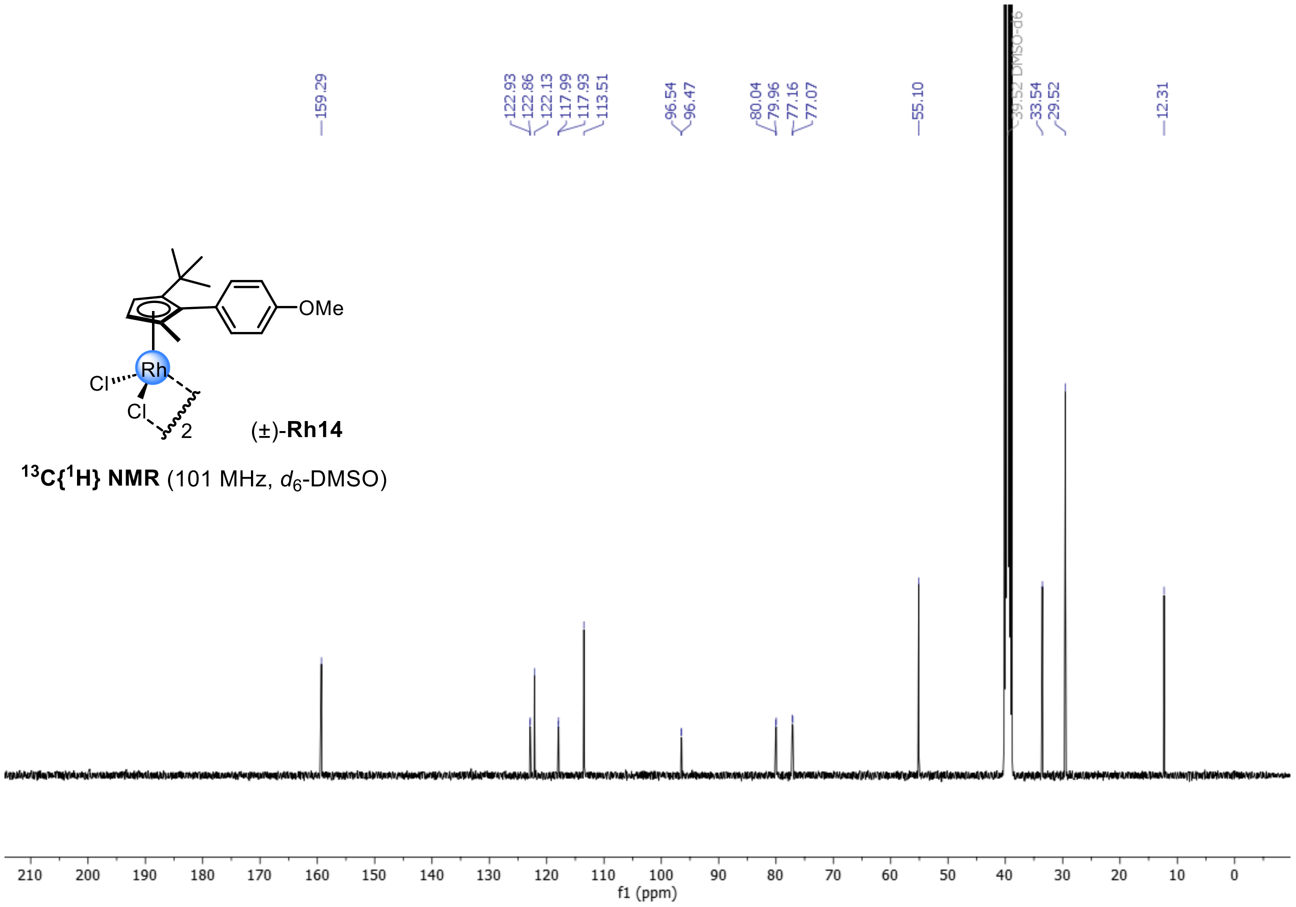
159.29
122.93
122.86
122.13
117.99
117.93
113.51
96.54
96.47
80.04
79.96
77.16
77.07
55.10
39.52 DMSO-d6
33.54
29.52
12.31
OMe
Rh
Cl
Cl
2
(±)-Rh14
^{13}C{^{1}H} NMR (101 MHz, d_6-DMSO)
f1 (ppm)

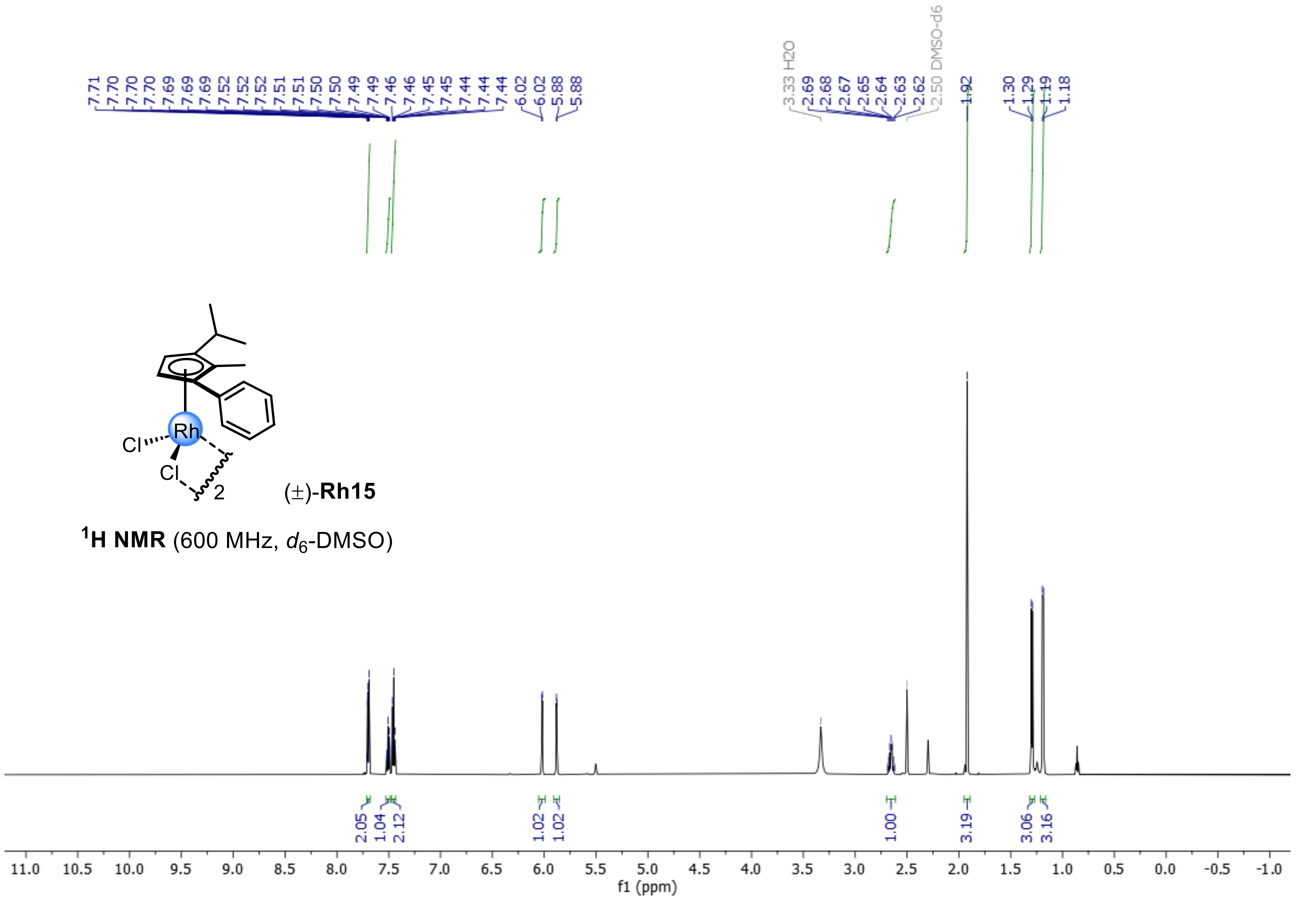

(±)-**Rh15**

^{1}H NMR (600 MHz, d_6-DMSO)

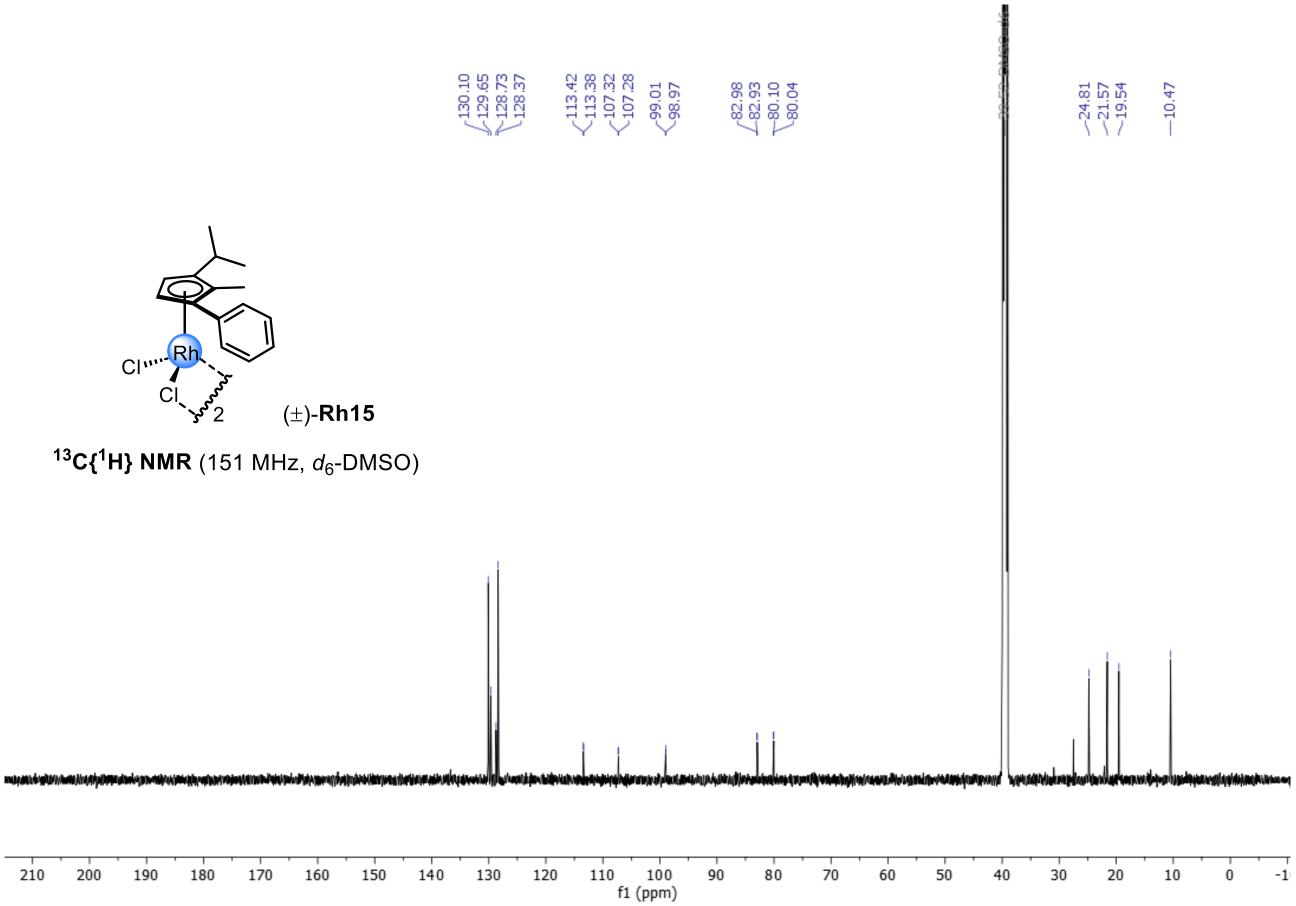

(±)-**Rh15**

^{13}C{^{1}H} NMR (151 MHz, d_6-DMSO)

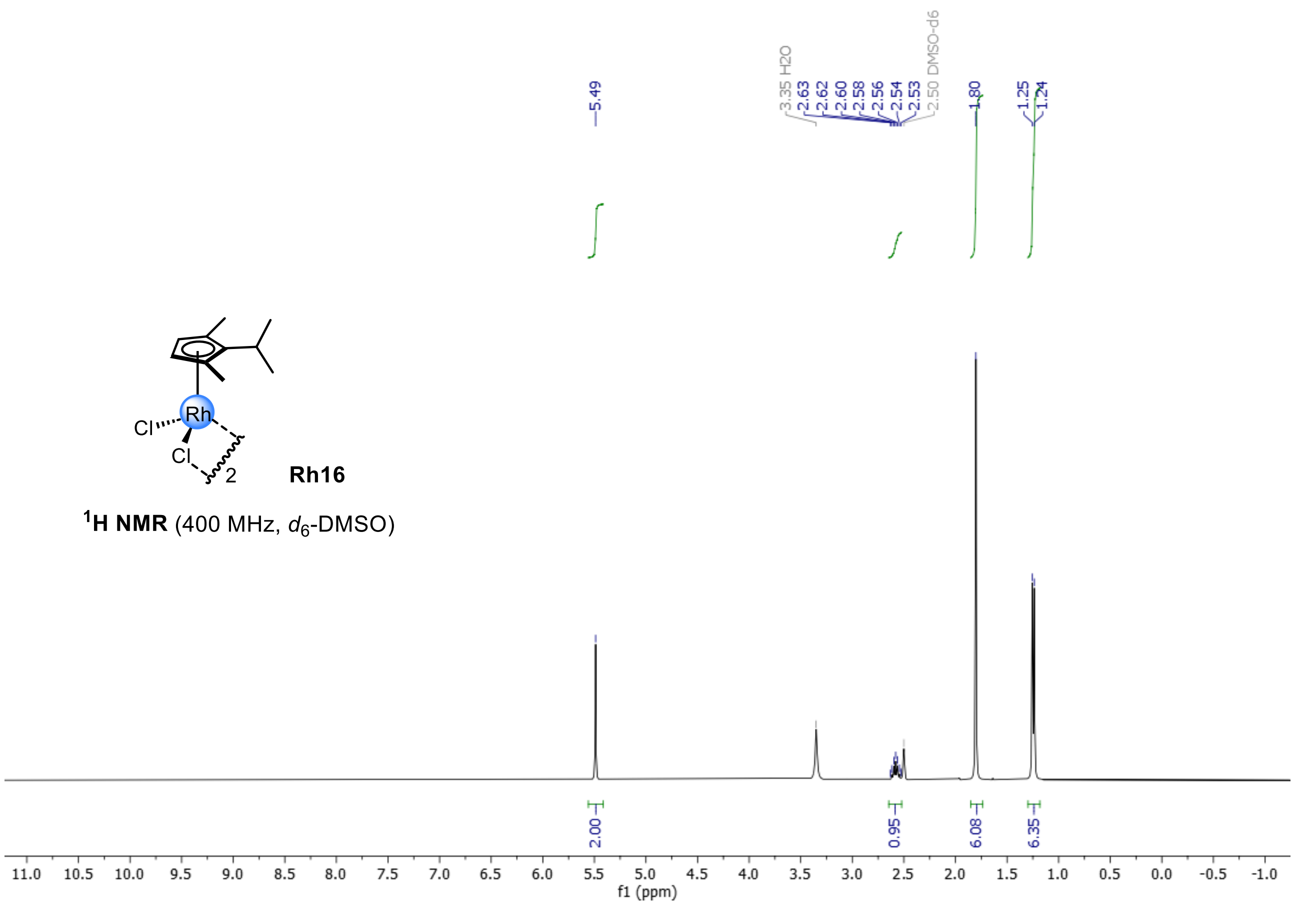

Rh16

^{1}H NMR (400 MHz, d_6-DMSO)

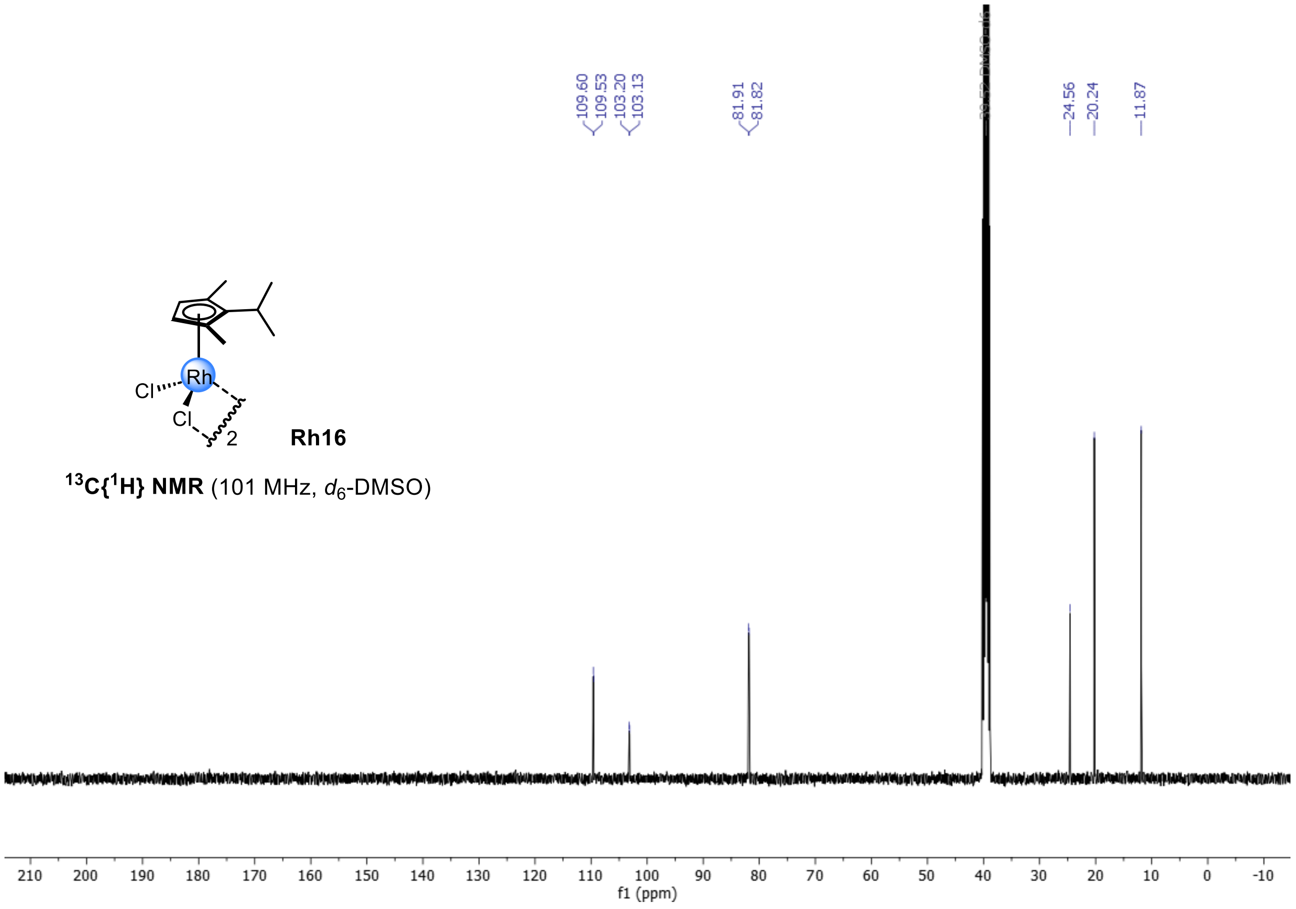

Rh16

^{13}C{^{1}H} NMR (101 MHz, d_6-DMSO)

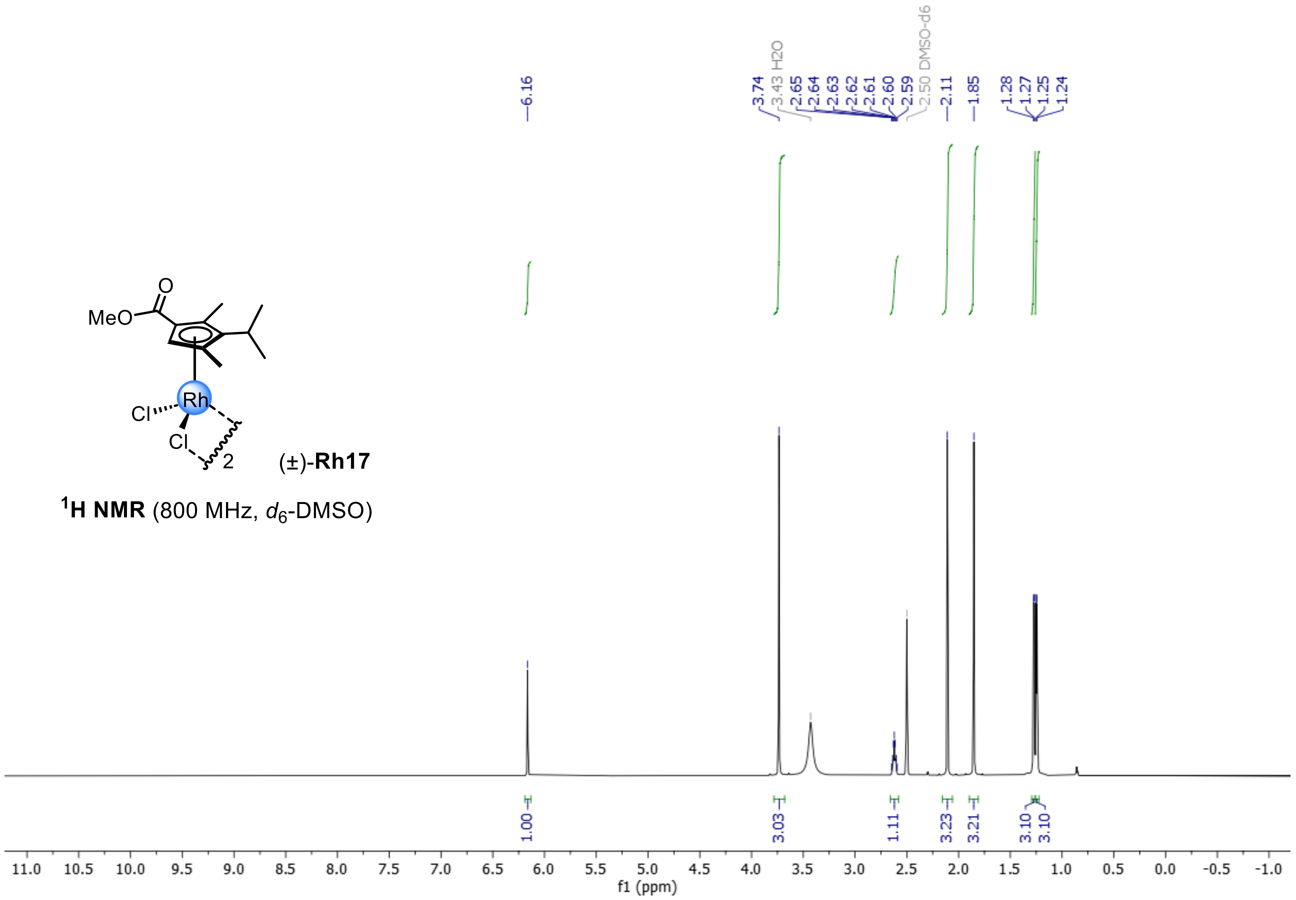

(±)-**Rh17**

^{1}H NMR (800 MHz, d_6-DMSO)

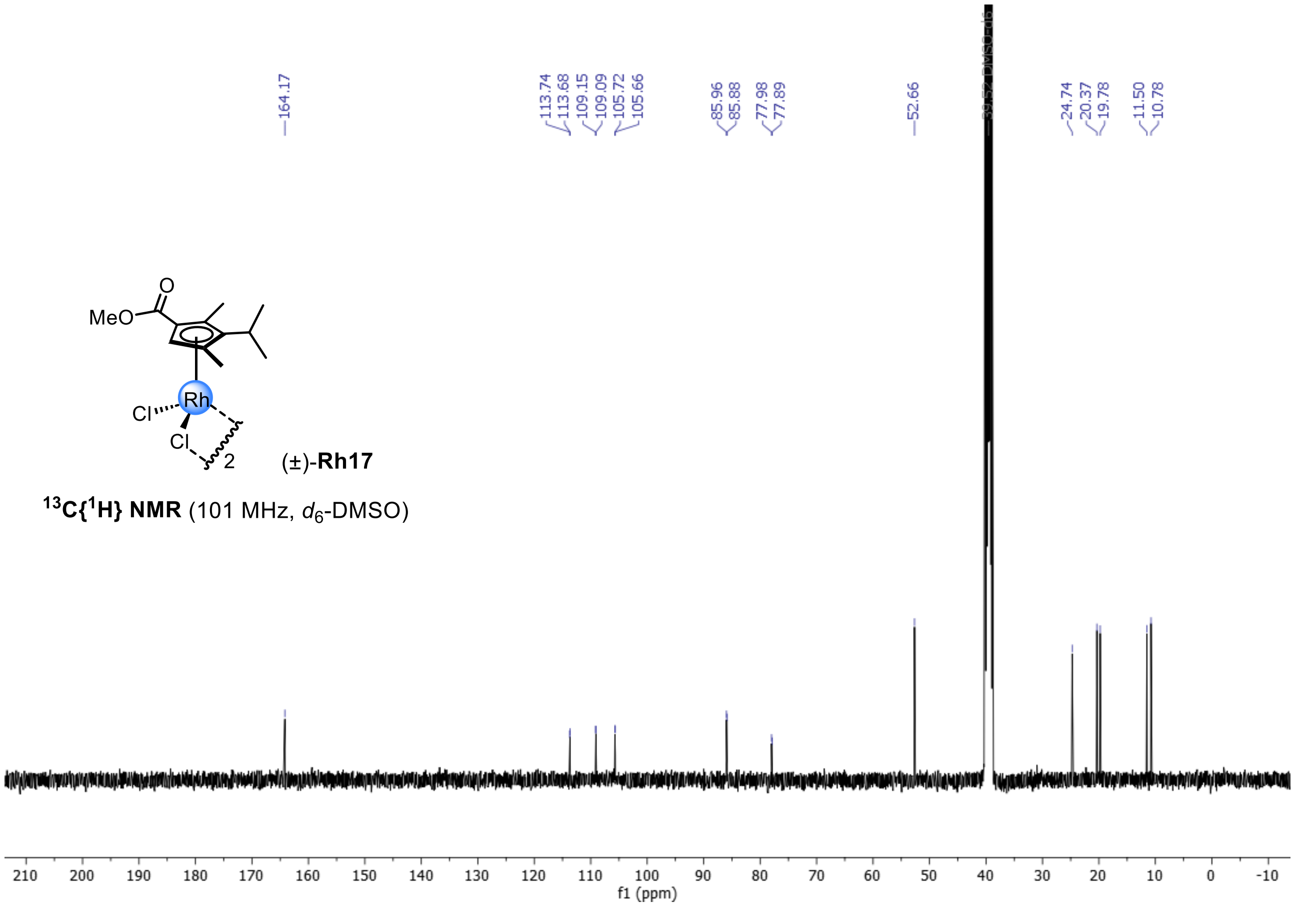

(±)-**Rh17**

^{13}C{^{1}H} NMR (101 MHz, d_6-DMSO)

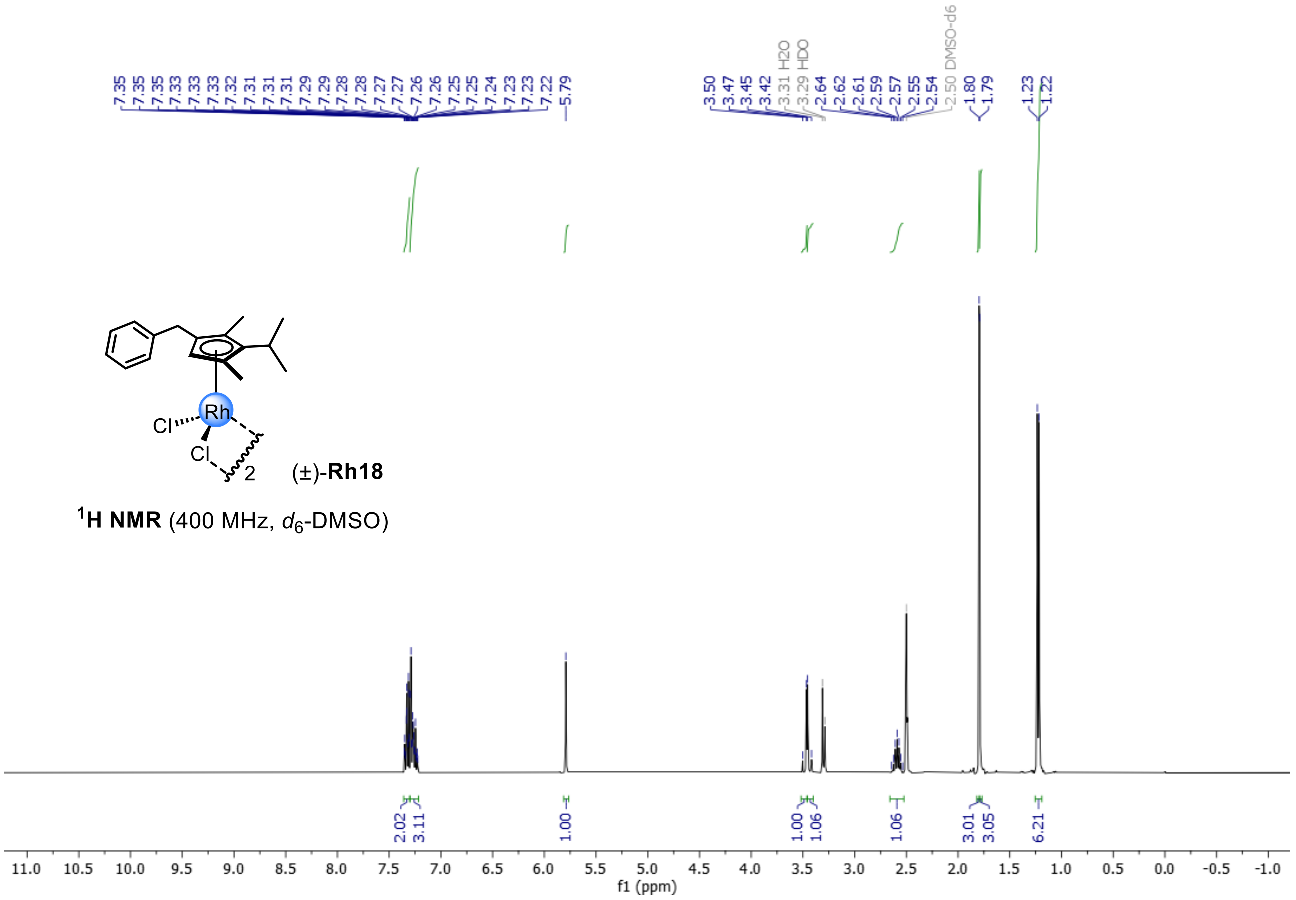
(±)-Rh18
¹H NMR (400 MHz, d_6-DMSO)
f1 (ppm)

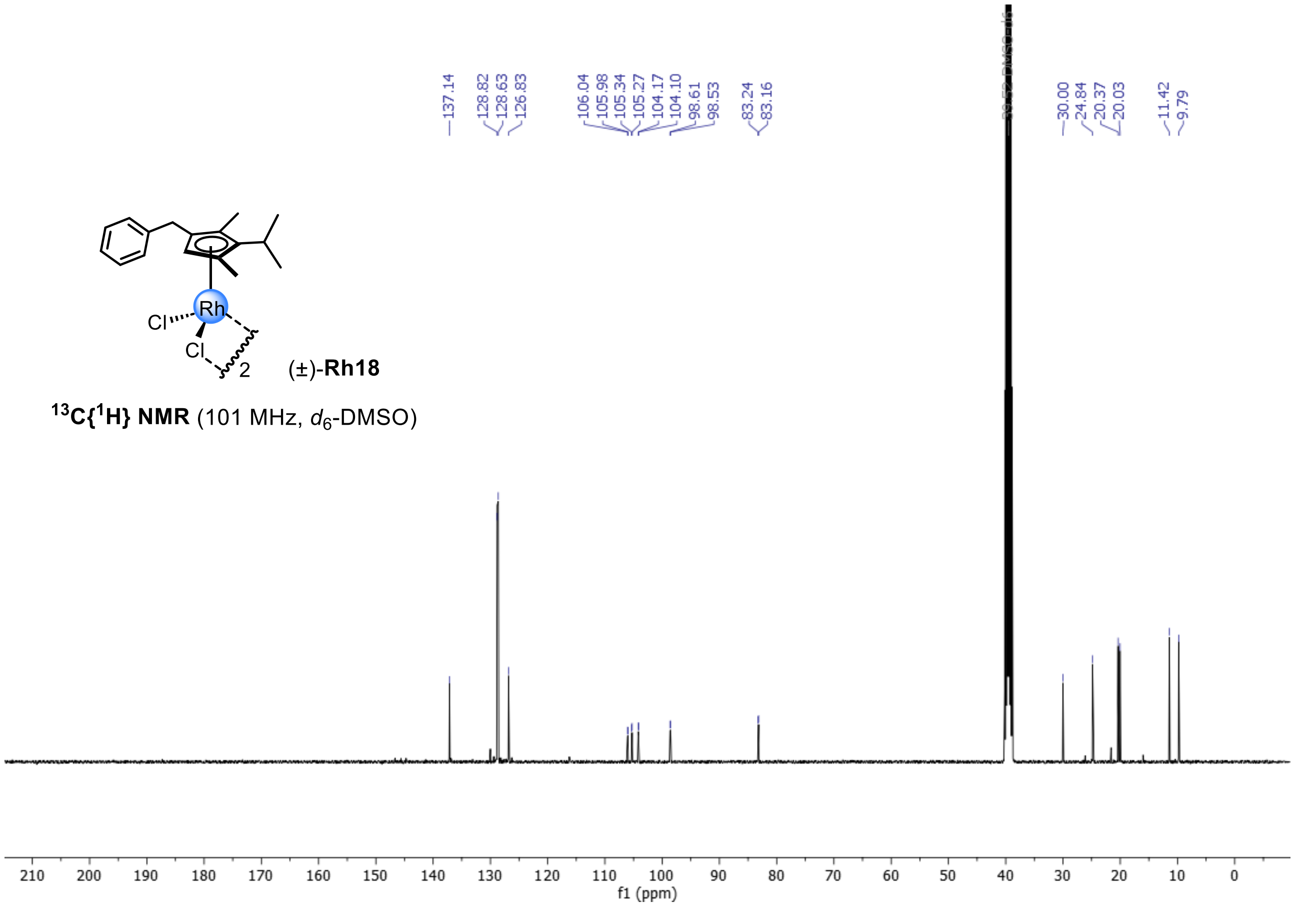
(±)-Rh18
¹³C{¹H} NMR (101 MHz, d_6-DMSO)
f1 (ppm)

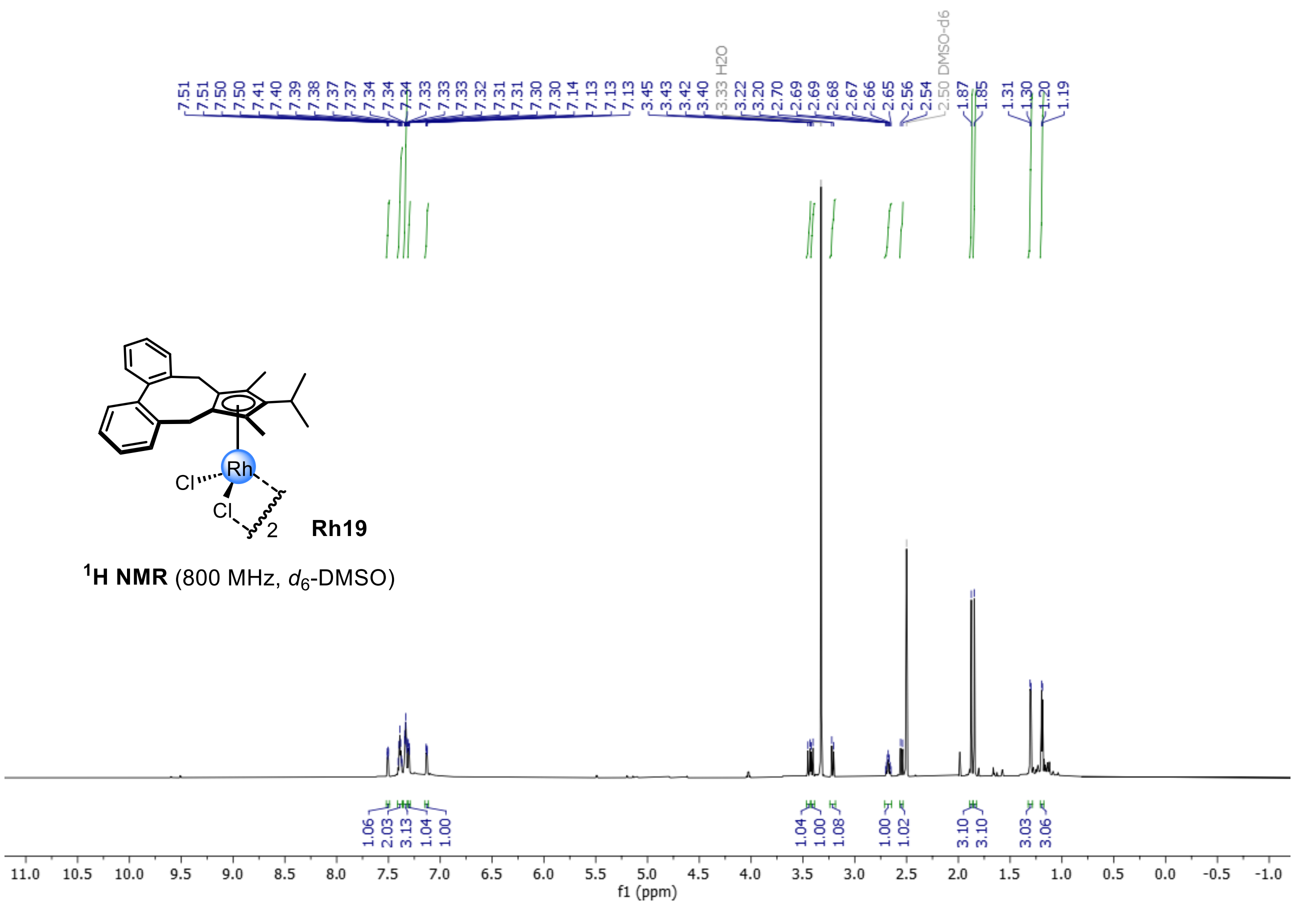
Rh
Cl
Cl
2
Rh19
1H NMR (800 MHz, d6-DMSO)
3.33 H2O
2.50 DMSO-d6
f1 (ppm)

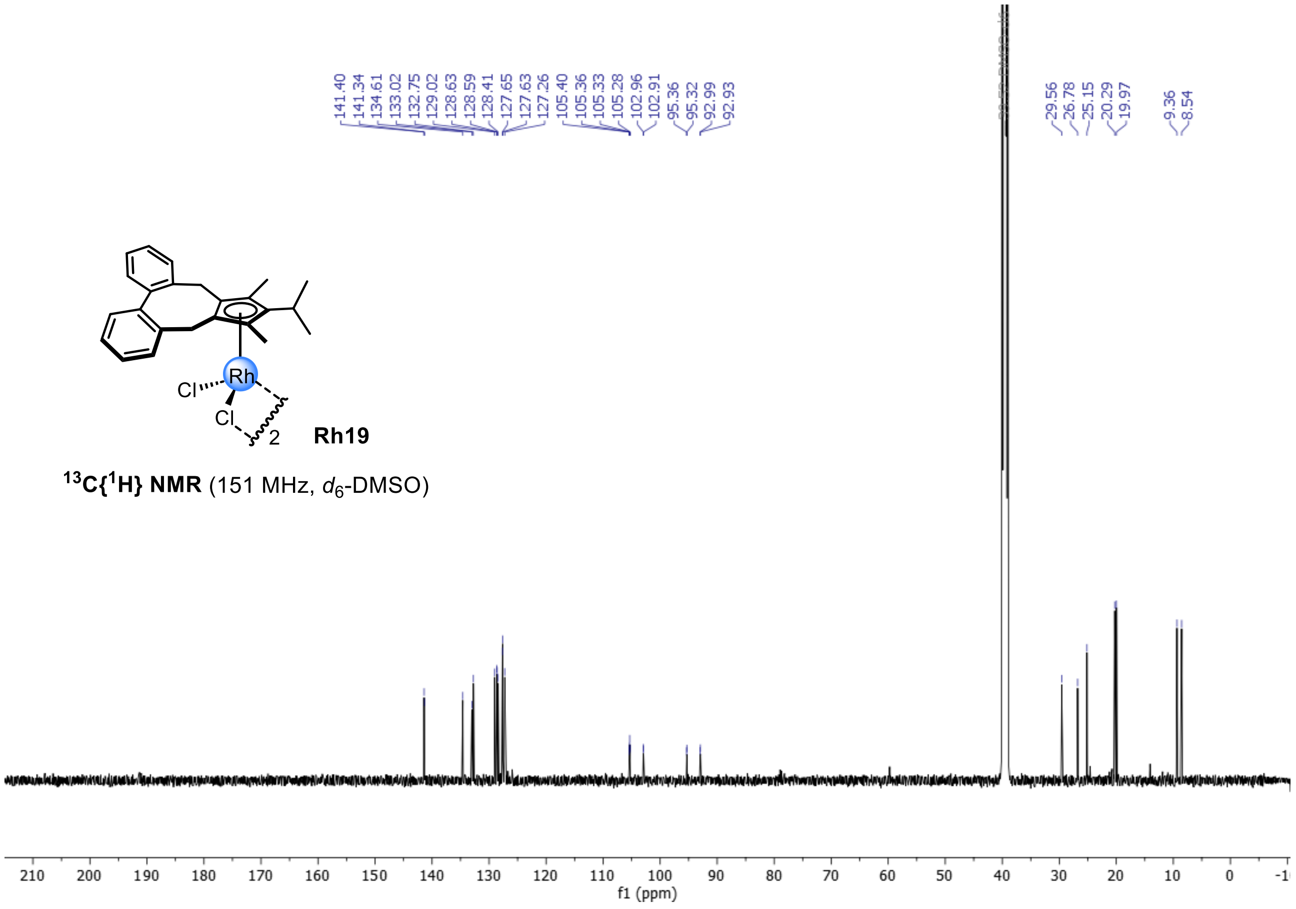
141.40
141.34
134.61
133.02
132.75
129.02
128.63
128.59
128.41
127.65
127.63
127.26
105.40
105.36
105.33
105.28
102.96
102.91
95.36
95.32
92.99
92.93
29.56
26.78
25.15
20.29
19.97
9.36
8.54
Rh
Cl
Cl
2
Rh19
13C{1H} NMR (151 MHz, d6-DMSO)
f1 (ppm)

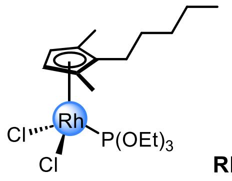

Rh1'

^{31}P{^{1}H} NMR (162 MHz, CD_2Cl_2)

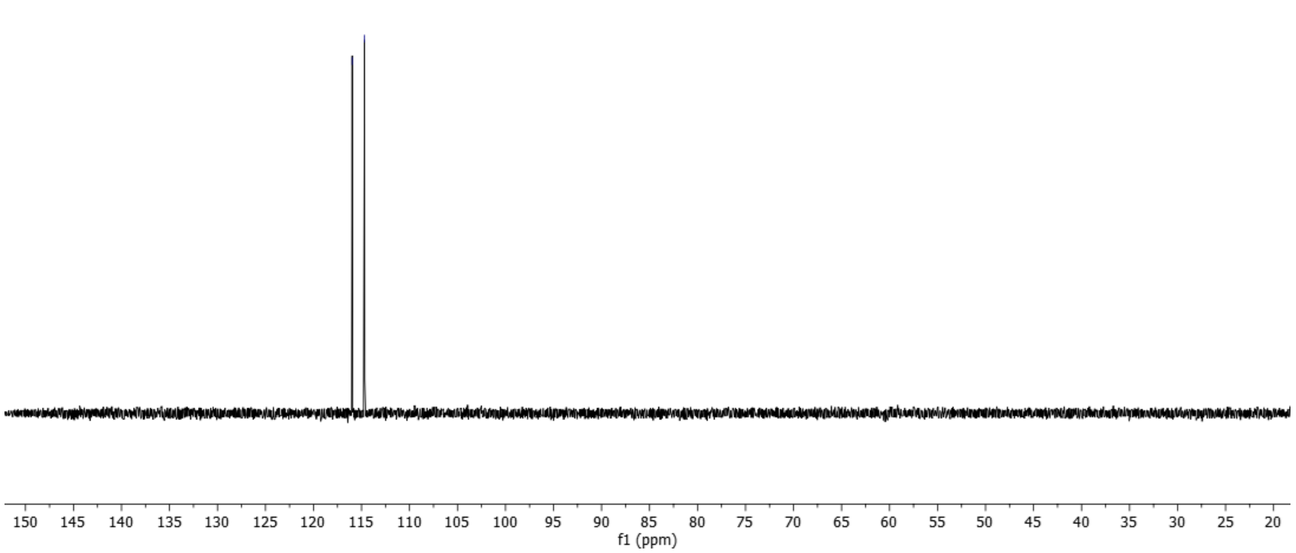

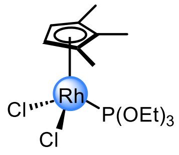

Rh2'

^{31}P{^{1}H} NMR (162 MHz, CD_2Cl_2)

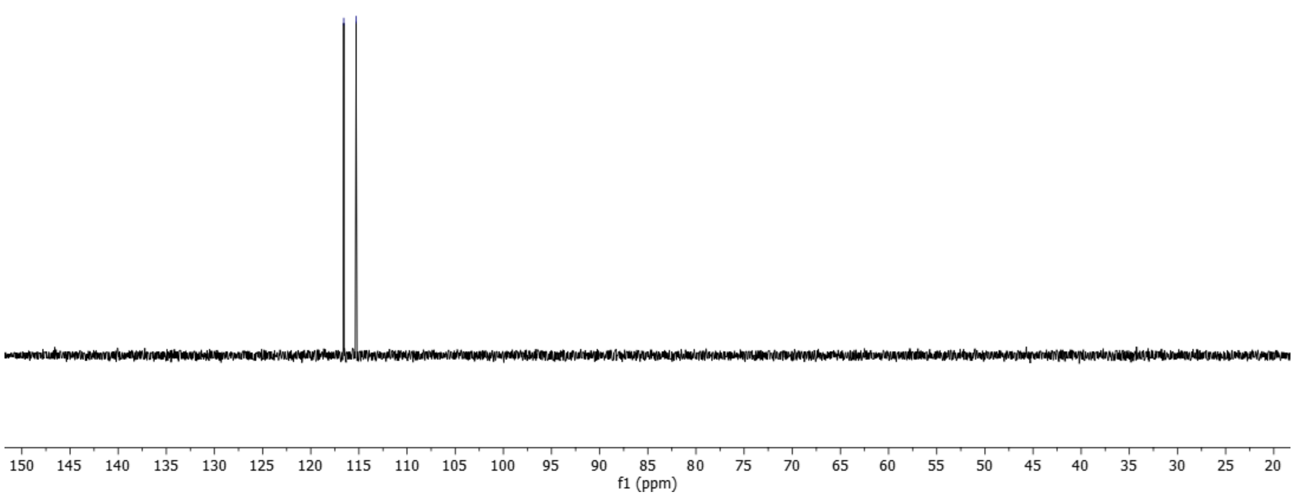

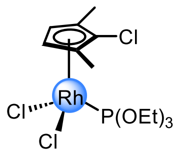

Rh3'

$^{31}P\{^1H\}$ NMR (162 MHz, CD_2Cl_2)

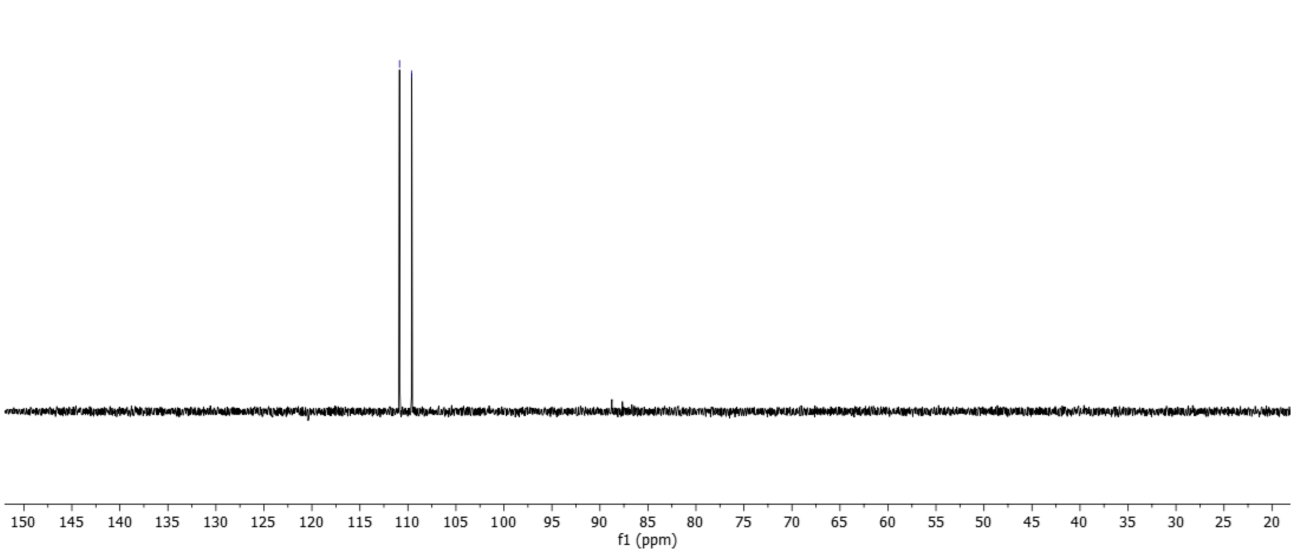

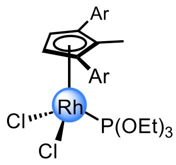

(Ar = 3,5-CF_3-C_6H_3) **Rh5'**

$^{31}P\{^1H\}$ NMR (162 MHz, CD_2Cl_2)

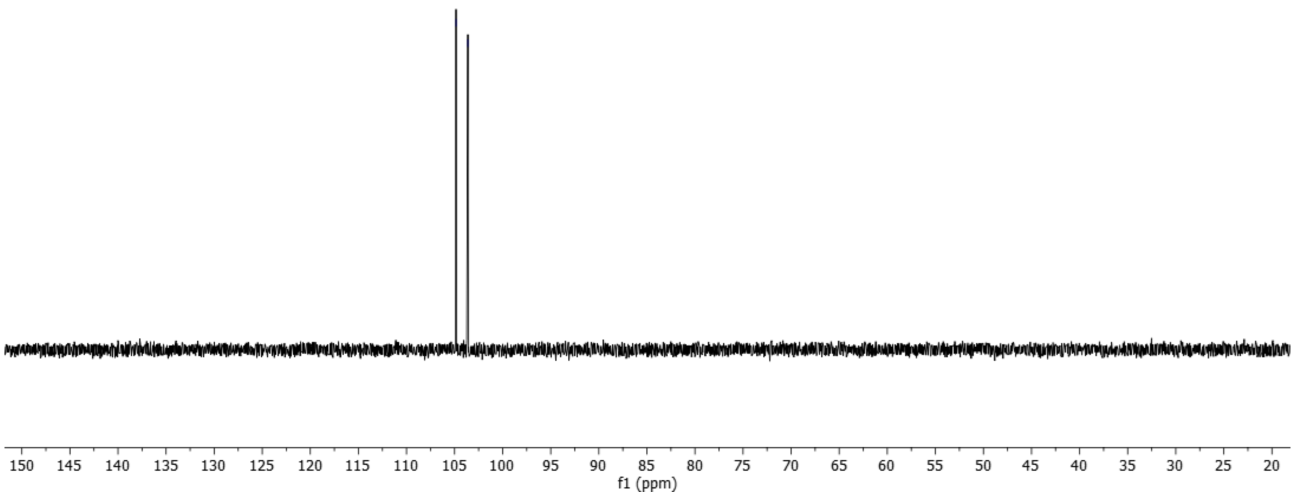

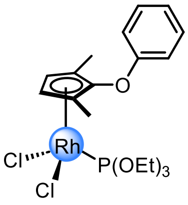

Rh6'

$^{31}P\{^{1}H\}$ NMR (162 MHz, CD_2Cl_2)

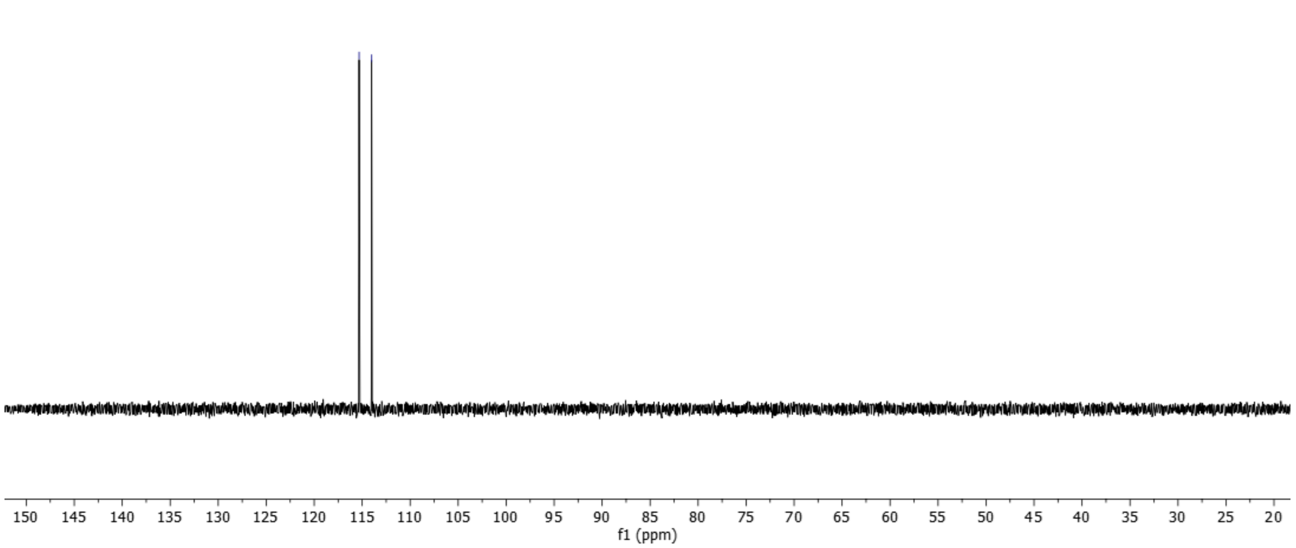

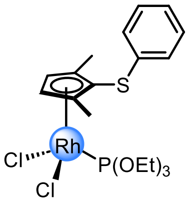

Rh7'

$^{31}P\{^{1}H\}$ NMR (162 MHz, CD_2Cl_2)

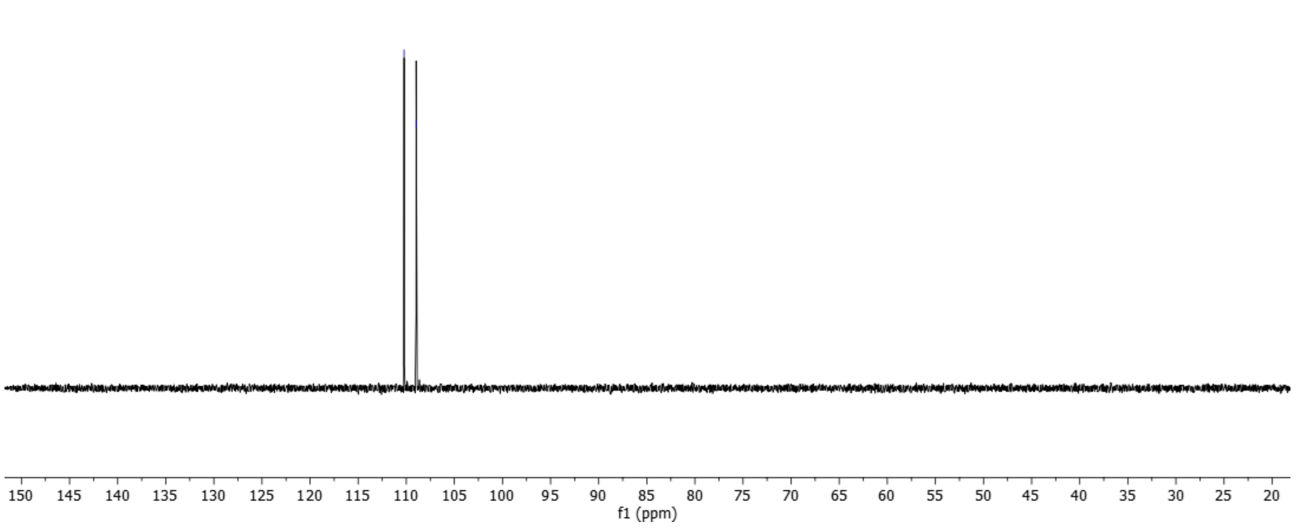

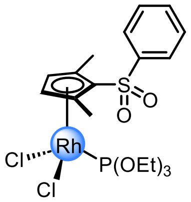

Rh8'

$^{31}P\{^1H\}$ NMR (162 MHz, CD_2Cl_2)

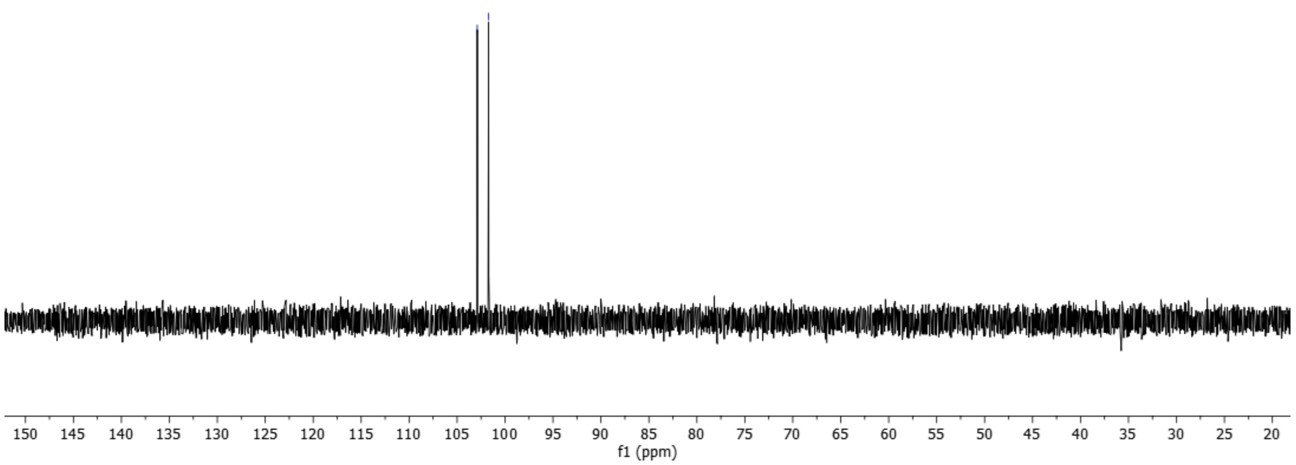

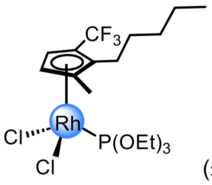

(±)-**Rh9'**

$^{31}P\{^1H\}$ NMR (162 MHz, CD_2Cl_2)

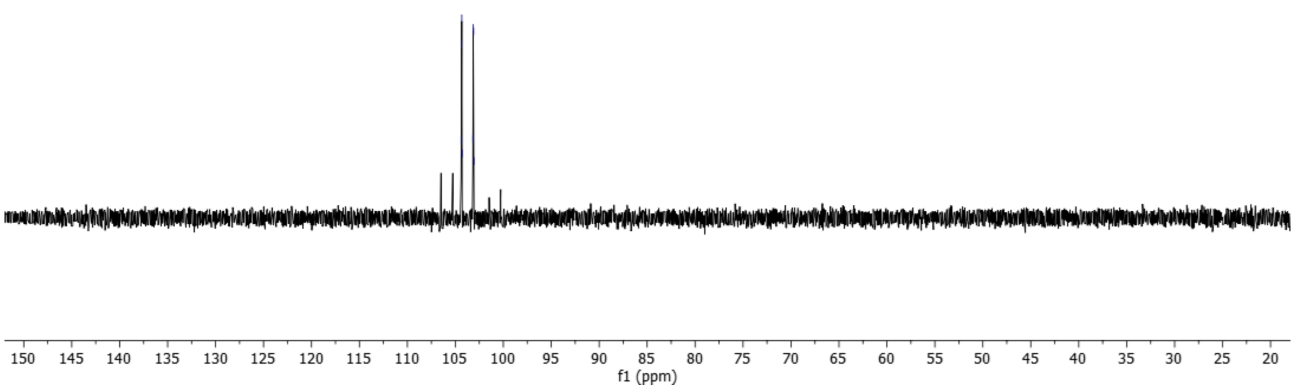

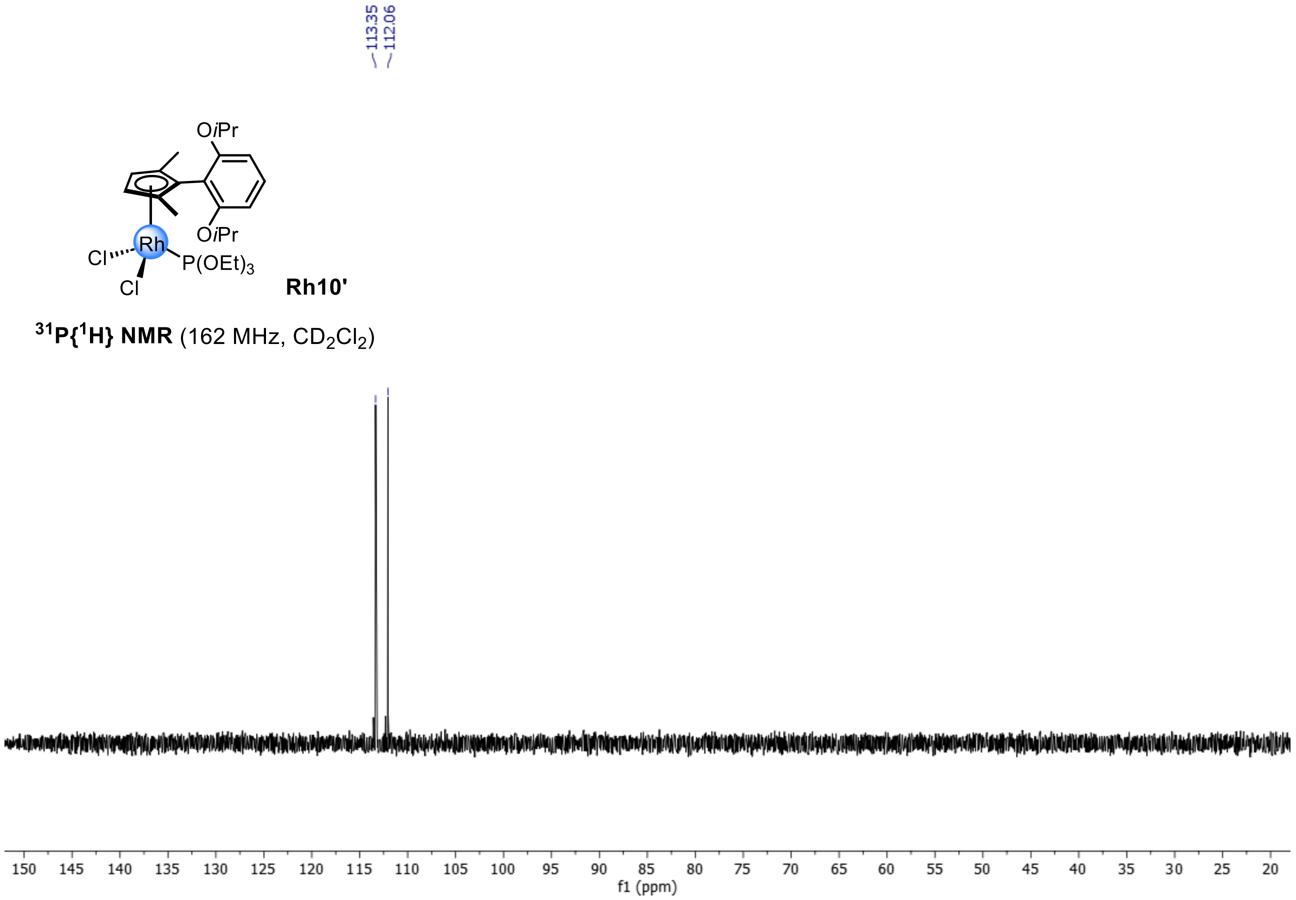

Rh10'

^{31}P{^{1}H} NMR (162 MHz, CD_2Cl_2)

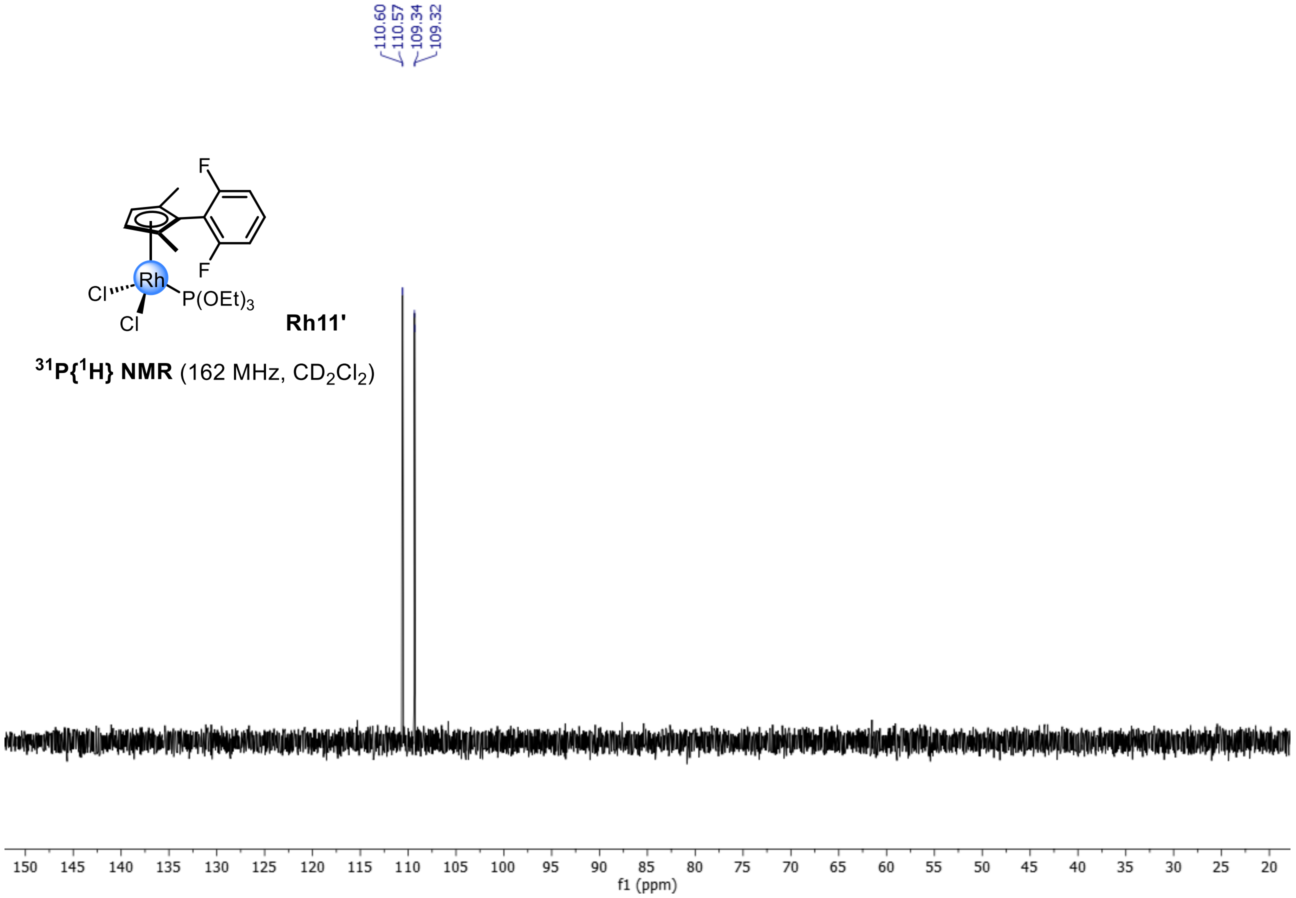

Rh11'

^{31}P{^{1}H} NMR (162 MHz, CD_2Cl_2)

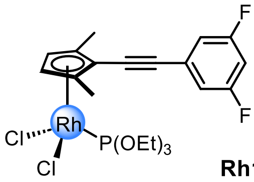

Rh12'

^{31}**P{^{1}H} NMR** (162 MHz, CD_2Cl_2)

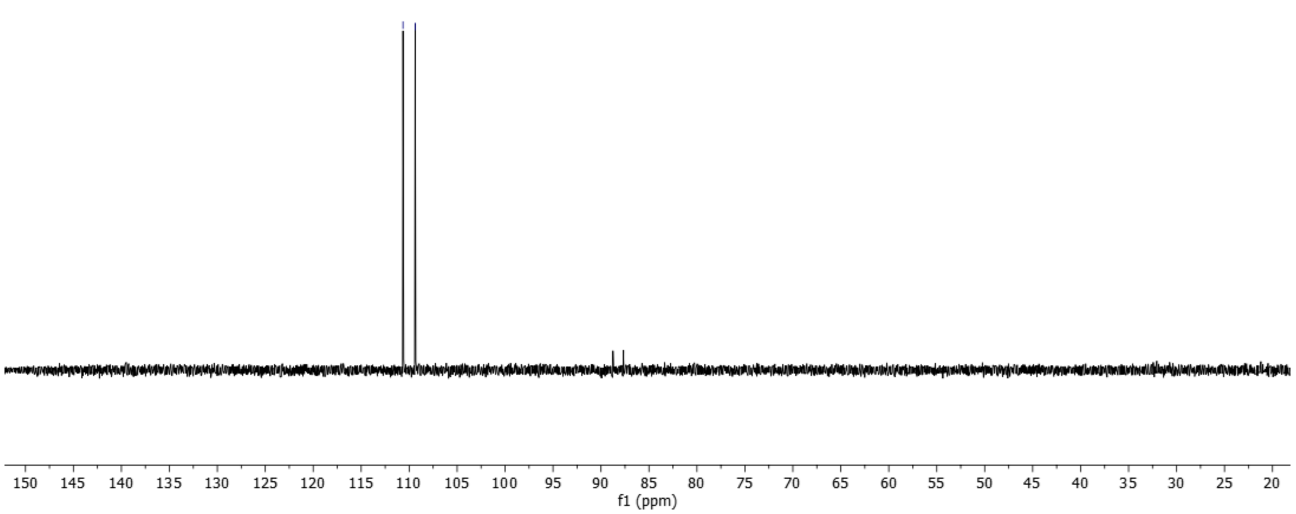

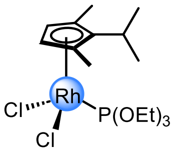

Rh16'

^{31}**P{^{1}H} NMR** (162 MHz, CD_2Cl_2)

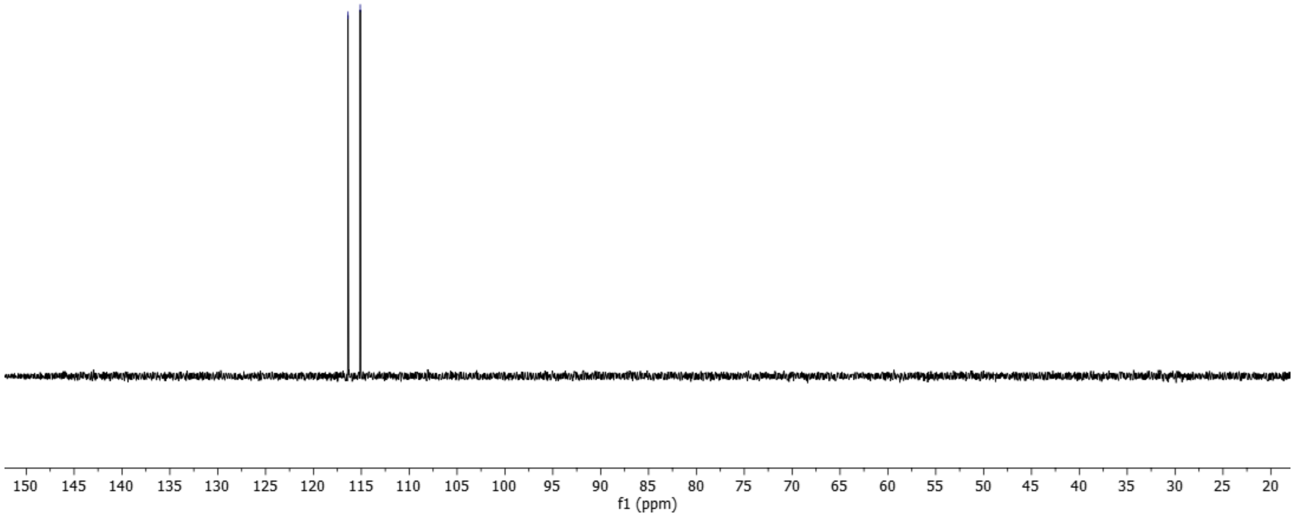

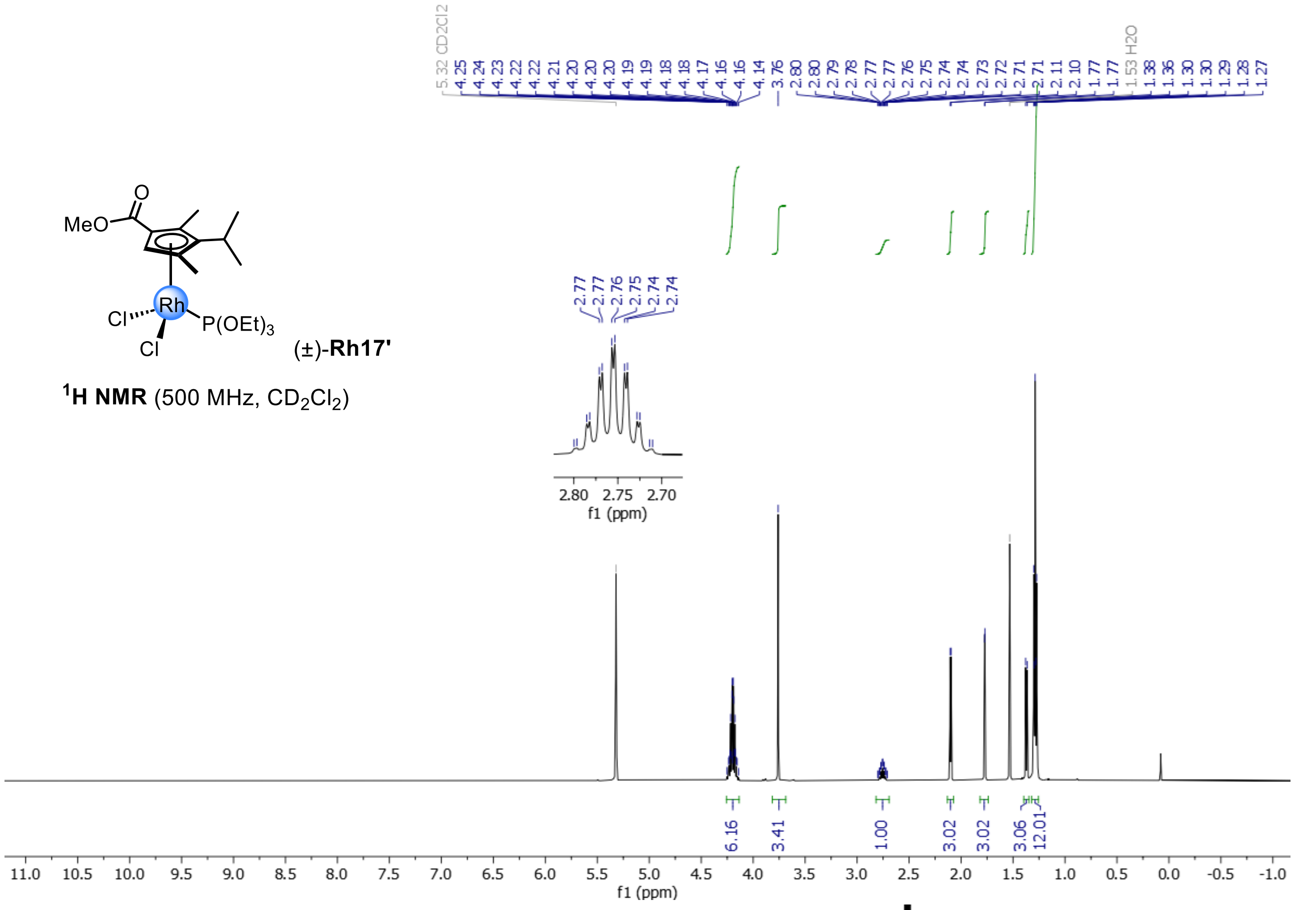

(±)-**Rh17'**

^{1}H NMR (500 MHz, CD_2Cl_2)

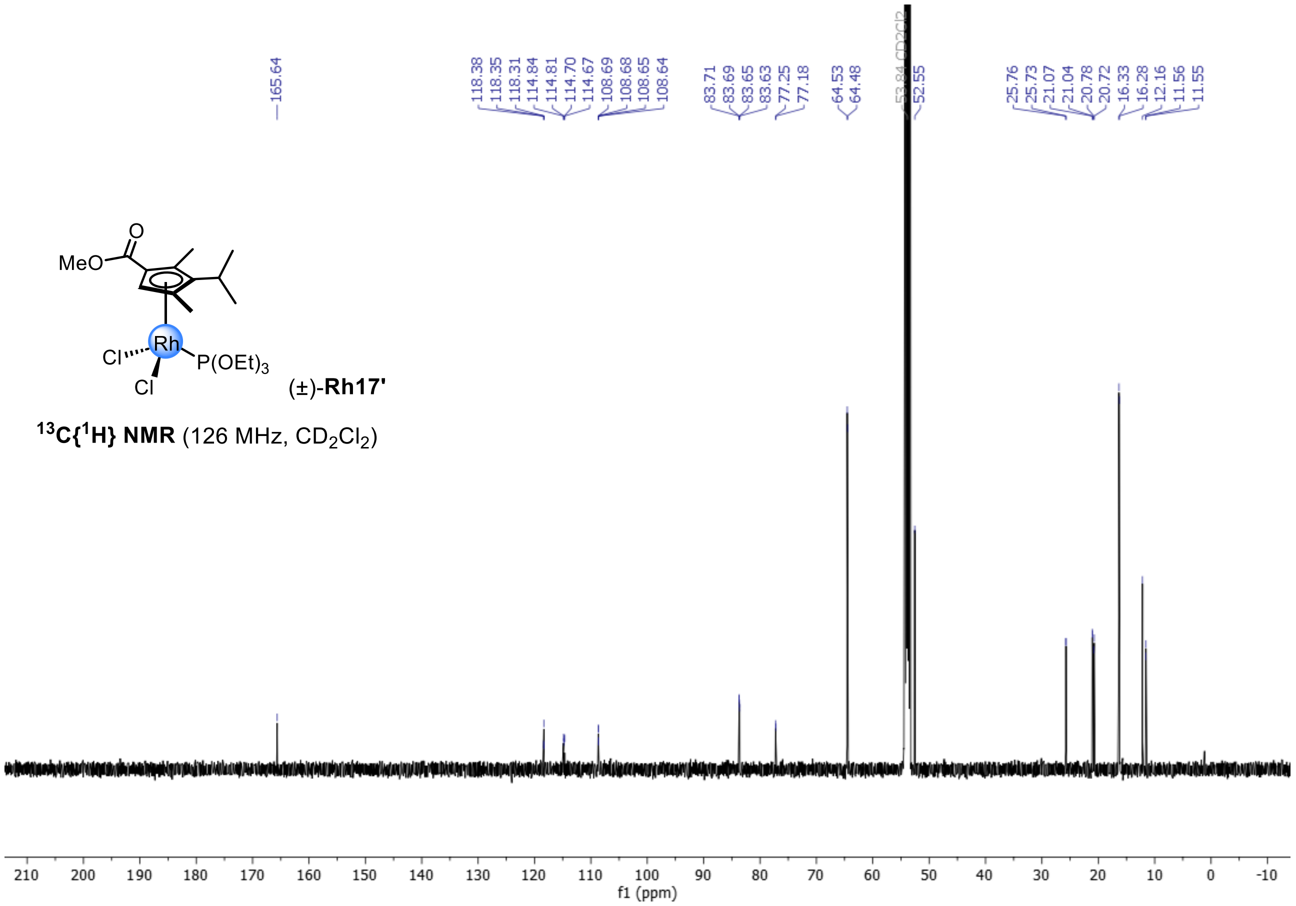

(±)-**Rh17'**

^{13}C{^{1}H} NMR (126 MHz, CD_2Cl_2)

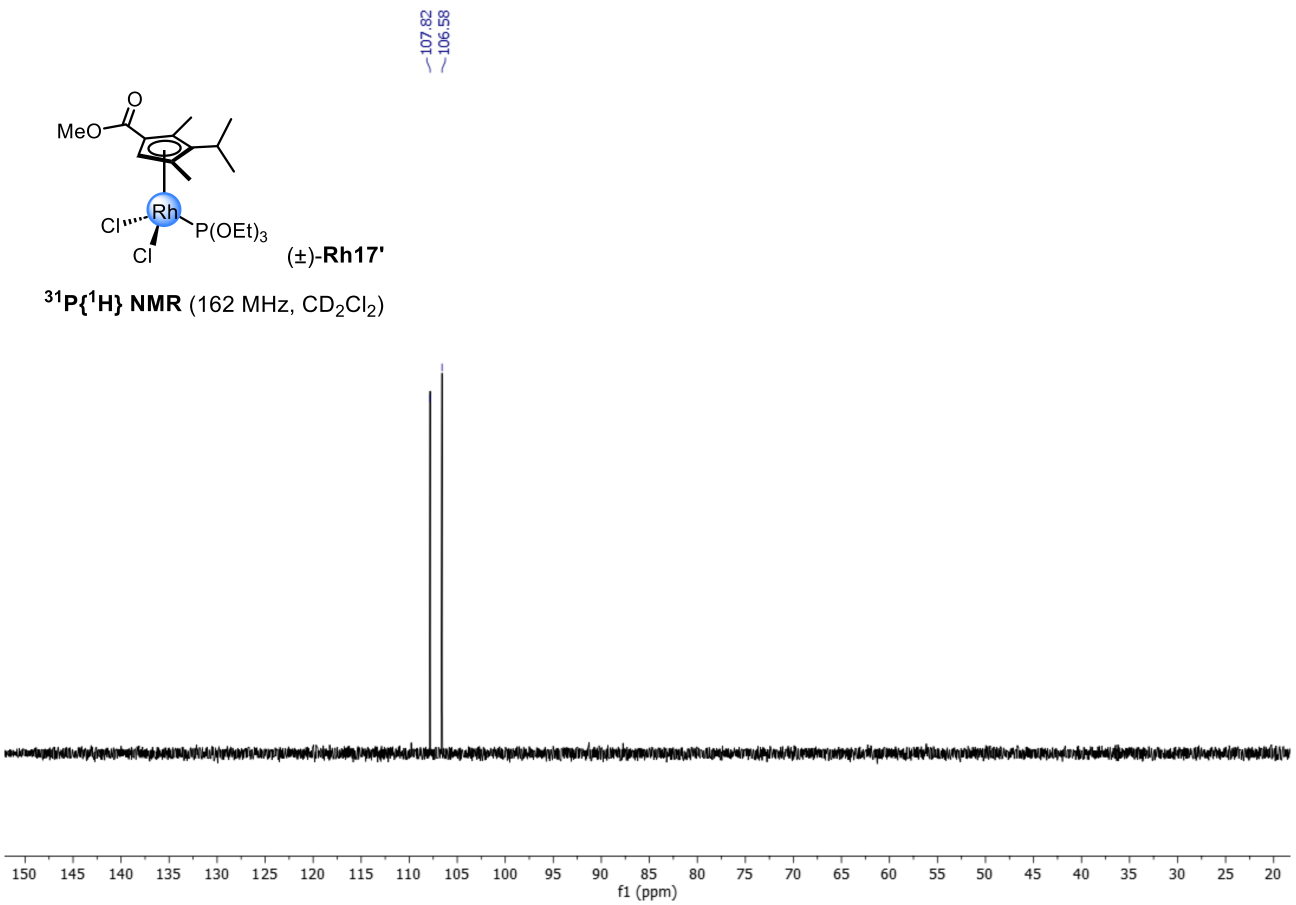

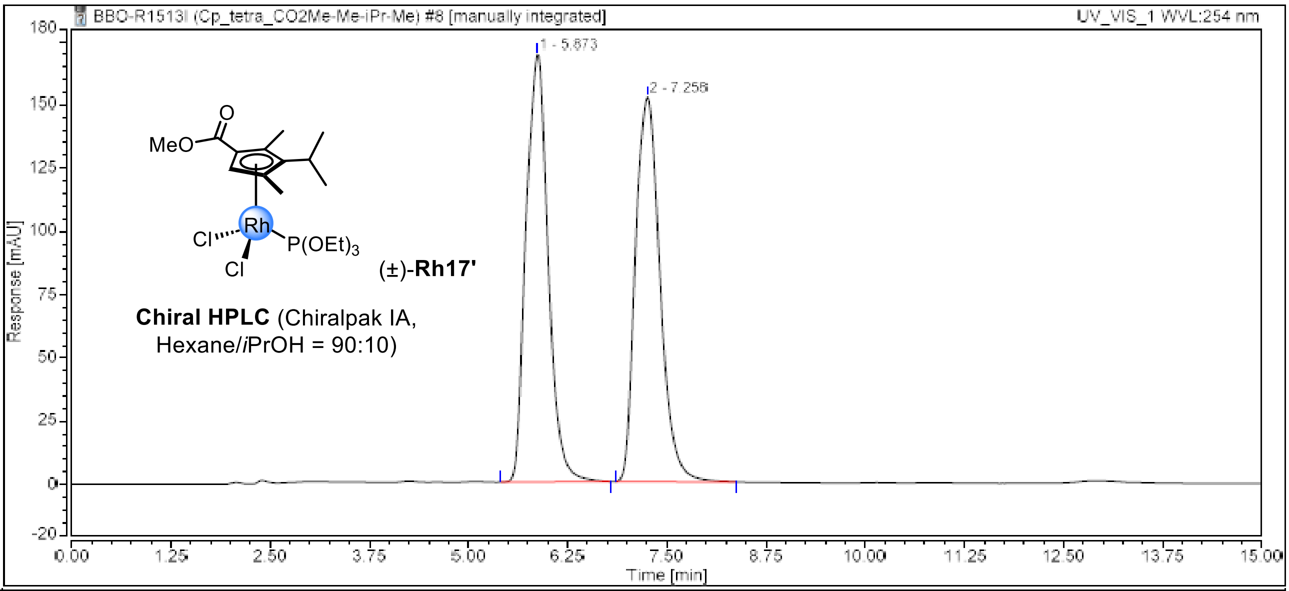

Integration Results

No.	Peak Name	Retention Time<br>min	Area<br>mAU*min	Height<br>mAU	Relative Area<br>%	Relative Height<br>%	Amount<br>n.a.
1		5.873	54.656	169.047	50.09	52.67	n.a.
2		7.258	54.453	151.916	49.91	47.33	n.a.

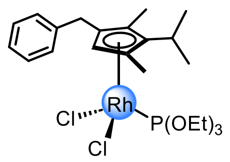

Rh18'

$^{31}P\{^1H\}$ NMR (162 MHz, CD_2Cl_2)

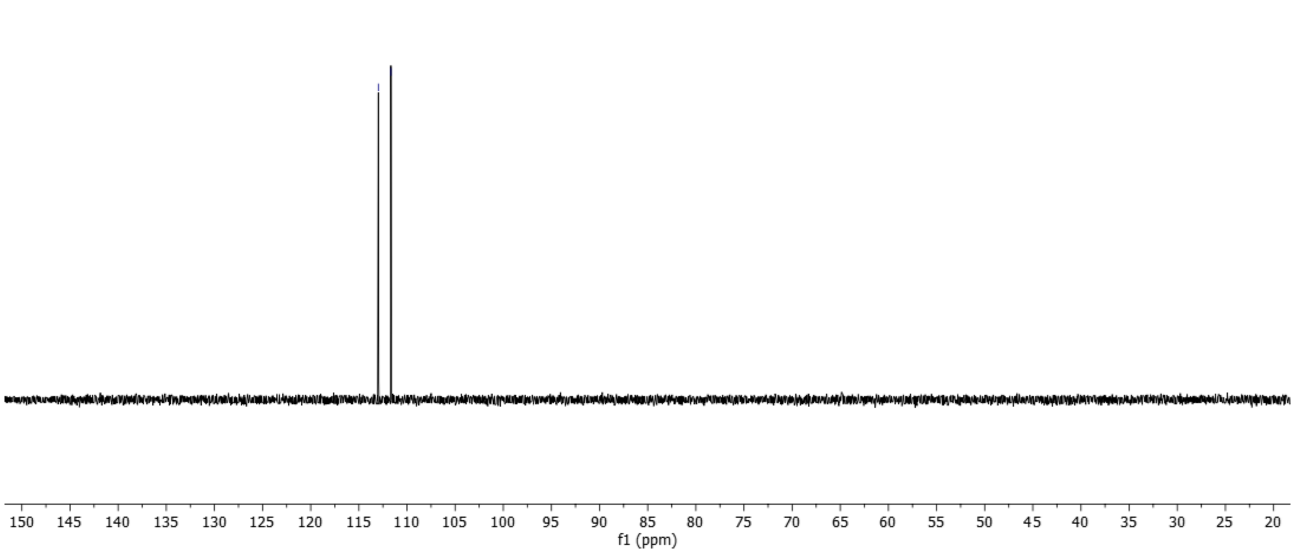

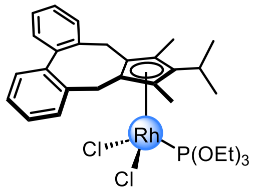

Rh19'

$^{31}P\{^1H\}$ NMR (162 MHz, CD_2Cl_2)

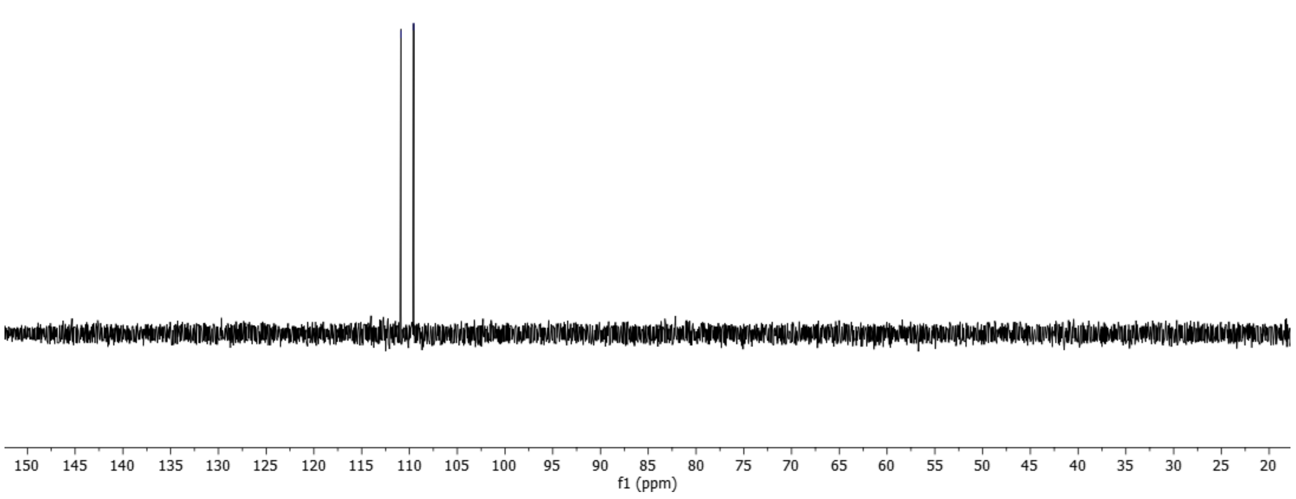

Supplement: Supplementary file 1 [file ja5c20631_si_001.pdf]
